# Supplementary material for: Heterochromatin formation and remodeling by IRTKS condensates counteract cellular senescence
Source: EMBO J. 2024 Aug 27;43(20):7. doi: 10.1038/s44318-024-00212-3 (PMC11480336; doi:10.1038/s44318-024-00212-3)
Supplement: Supplementary file 1 — Appendix [file 44318_2024_212_MOESM1_ESM.pdf]

Appendix

Heterochromatin formation and remodeling by IRTKS condensates counteract cellular senescence

Jia Xie *et al*

*Corresponding Author: Ze-Guang Han, hanzg@sjtu.edu.cn

Table of contents

Page no.

Appendix Figure S1 2

Appendix Figure S2 6

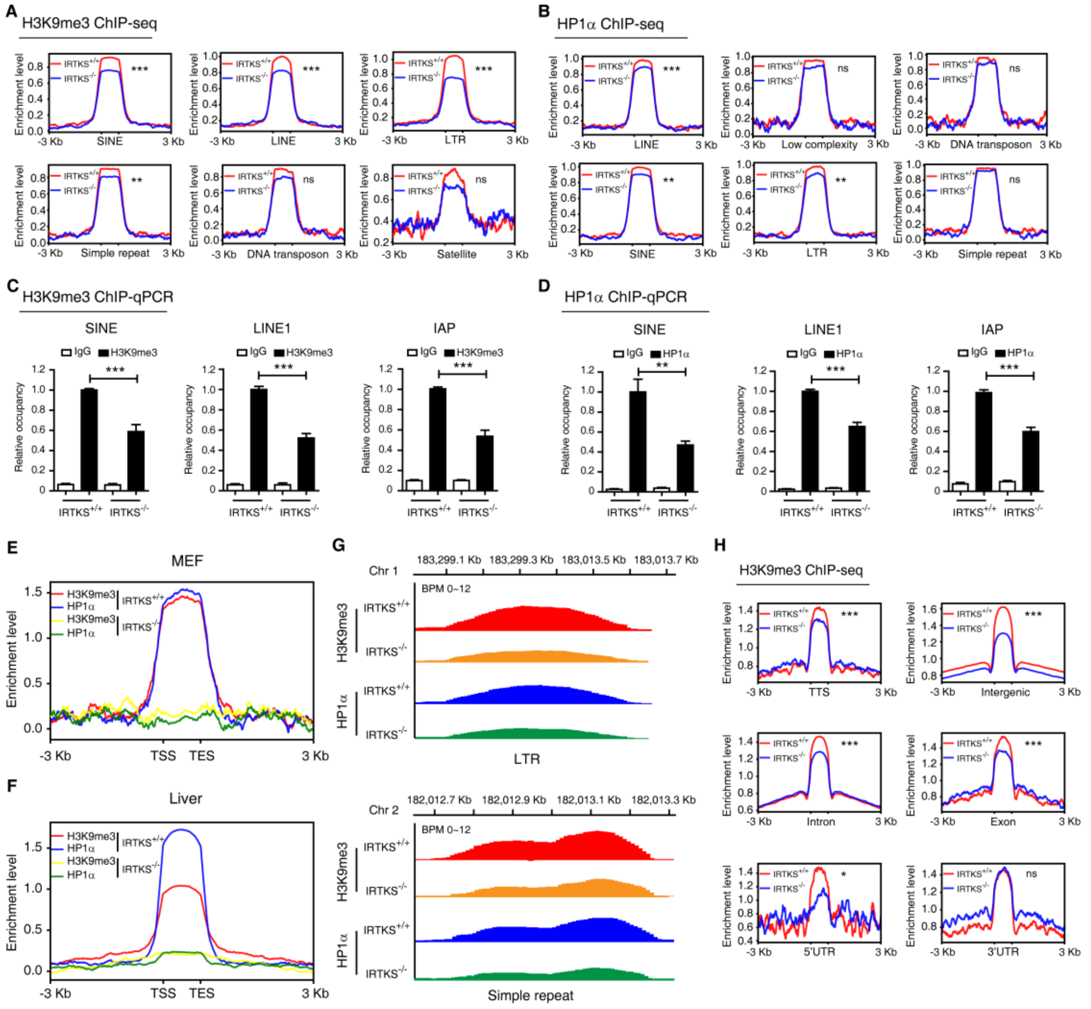
A
H3K9me3 ChIP-seq
B
HP1α ChIP-seq
C
H3K9me3 ChIP-qPCR
D
HP1α ChIP-qPCR
E
MEF
F
Liver
G
LTR
Simple repeat
H
H3K9me3 ChIP-seq

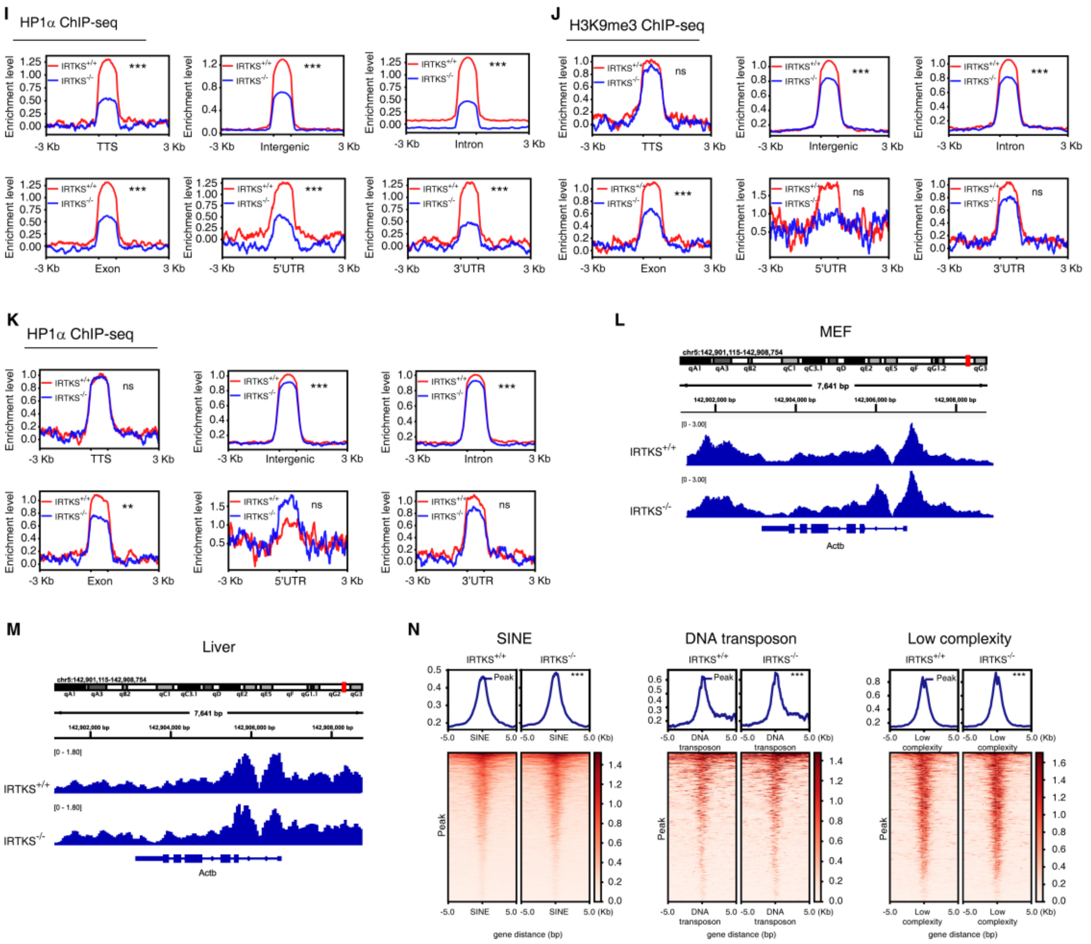
I
HP1α ChIP-seq
J
H3K9me3 ChIP-seq
Enrichment level
IRTKS+/+
IRTKS-/-
***
ns
**
-3 Kb
3 Kb
TTS
Intergenic
Intron
Exon
5'UTR
3'UTR
K
HP1α ChIP-seq
L
MEF
chr5:142,901,115-142,908,754
7,641 bp
142,902,000 bp
142,904,000 bp
142,906,000 bp
142,908,000 bp
[0 - 3.00]
Actb
M
Liver
[0 - 1.80]
N
SINE
DNA transposon
Low complexity
Peak
gene distance (bp)
(Kb)

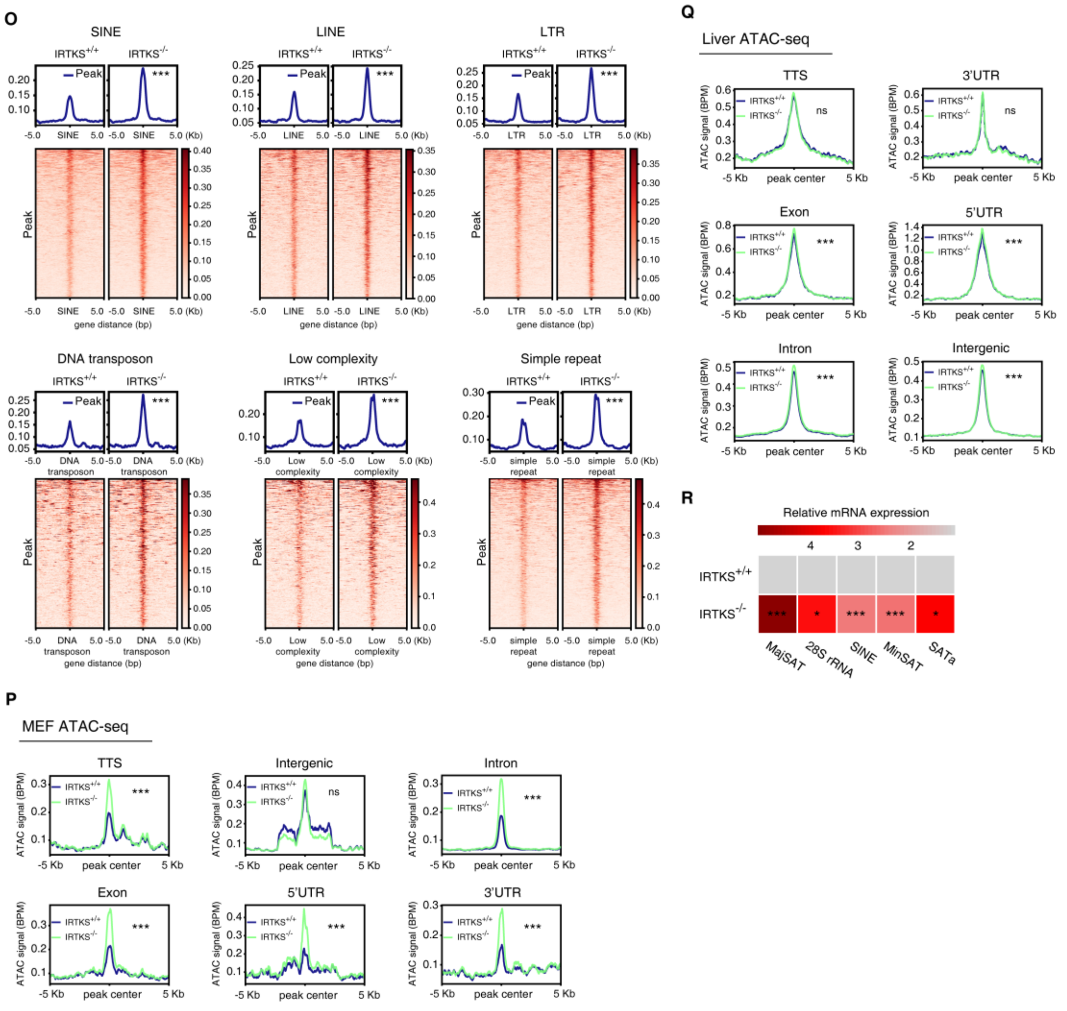

Appendix Figure S1. IRTKS suppresses the expression of repetitive sequences.

(A, B) ChIP-seq enrichment profiles of H3K9me3 (**A**) and HP1α (**B**) peaks showing the loss of H3K9me3 and HP1α signals at repetitive sequence regions in *Irtks* KO MEFs. **(C, D)** Enrichment of H3K9me3 (**C**) and HP1α (**D**) within regions of repetitive sequences (LINE1, SINE and IAP) in MEFs from WT and *Irtks* KO mice as measured by ChIP–qPCR. n = 3. **(E, F)** ChIP-seq enrichment profiles of H3K9me3 and HP1α peaks on the gene body and flanking regions, including the transcription start site (TSS), transcription end sites (TES), and 3 kb up- and downstream regions, in MEFs (**E**) and livers (**F**) from WT and *Irtks* KO mice. **(G)** Visualization of the co-localization of H3K9me3 and HP1α on representative genomic regions corresponding to the indicated repetitive sequences in MEFs from WT and Irtks-KO mice. **(H-K)** ChIP-seq enrichment profiles of H3K9me3 and HP1α peaks showing a significant reduction on some non-repetitive sequence regions (TTS, intergenic, intron, exon, 5'UTR and 3'UTR) in the livers (**H, I**) and MEFs (**J, K**) of *Irtks* KO mice, as compared to those of WT mice. **(L, M)** Visualization of chromatin accessibility at the control locus (*Actb*) of MEF cells (**L**) and livers (**M**) from WT and *Irtks* KO mice**.** **(N)** Heatmaps showing ATAC signals ranging from 5 kb upstream to 5 kb downstream of ATAC-seq peaks of repetitive sequence regions (SINE, DNA transposon and low complexity) in livers from WT and *Irtks* KO mice. **(O)** Heatmaps showing the read signals of ATAC-seq peaks ranging from 5 kb upstream to 5 kb downstream of repetitive sequence regions (SINE, LINE,

LTR, DNA transposon, low complexity and simple repeat) in MEFs from WT and *Irtks* KO mice. **(P, Q)** Heatmaps showing the ATAC signals ranging from 5 kb upstream to 5 kb downstream of non-repetitive sequence regions (TTS, intergenic, intron, exon, 5'UTR and 3'UTR) in MEFs (**P**) and livers (**Q**) from WT and *Irtks* KO mice. **(R)** Heatmap showing the mRNA expression levels determined by RT–qPCR of repetitive sequences in MEFs from WT and *Irtks* KO mice. n = 3. Data are presented as the mean ± SD or mean ± SEM. Appendix Figs.1C and 1D were tested by one-way ANOVA followed by Tukey's post hoc test. The remaining plots were tested by Student's t test.

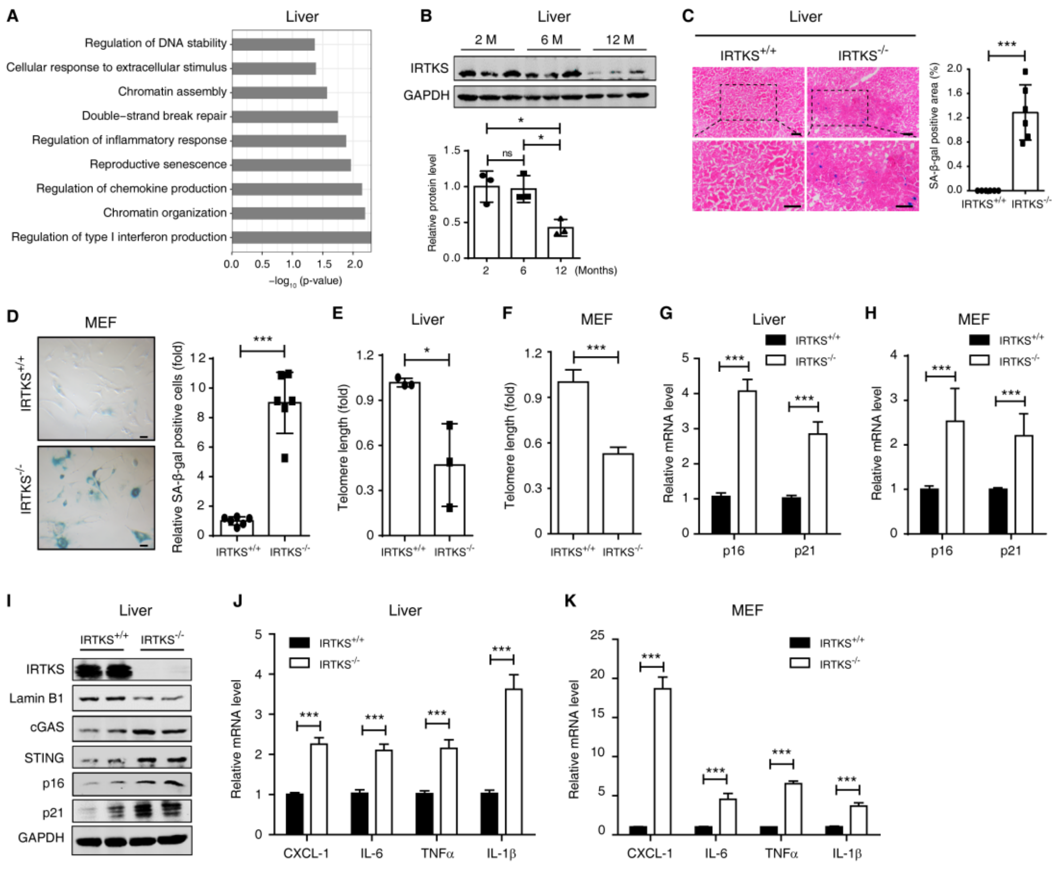

Appendix Figure S2. IRTKS deficiency promotes cellular senescence.

(A) GO analysis of DEGs in the livers of *Irtks* KO mice. **(B)** Western blotting images and quantification showing that the IRTKS protein was downregulated in the livers of 12-month-old mice (n = 3). Fold change represents the normalized IRTKS signal (IRTKS/GAPDH). **(C)** Representative images and quantification of SA-β-gal staining in the livers of 12-month-old WT and *Irtks* KO mice. Scale bars, 100 µm. n = 6. **(D)** Representative images and quantification of SA-β-gal staining in MEFs from WT and *Irtks* KO mice. Scale bars, 50 µm. n = 6. **(E, F)** Telomere length analysis in livers (**E**) and MEFs (**F**) from WT and *Irtks* KO mice by qPCR. n = 3. **(G, H)** p21 and p16 transcriptional expression in livers (**G**) and MEFs (**H**) from *Irtks* KO mice, as assessed by qPCR. n = 3. **I,** Western blotting analyses of cellular senescence-related molecules in livers of WT and *Irtks* KO mice. GAPDH was used as the loading control. **(J, K)** SASP-associated genes are significantly upregulated in livers (**J**) and MEFs (**K**) from *Irtks* KO mice compared to in those from WT mice. n = 3. Data are presented as the mean ± SD or mean ± SEM. Appendix Figs.2C-2F were tested by two-tailed Student’s t test. The remaining plots were tested by one-way ANOVA followed by Tukey’s post hoc test.
