# Supplementary figures and images for: Heterochromatin formation and remodeling by IRTKS condensates counteract cellular senescence (part 1 of 4)
Source: EMBO J. 2024 Aug 27;43(20):7. doi: 10.1038/s44318-024-00212-3 (PMC11480336; doi:10.1038/s44318-024-00212-3)

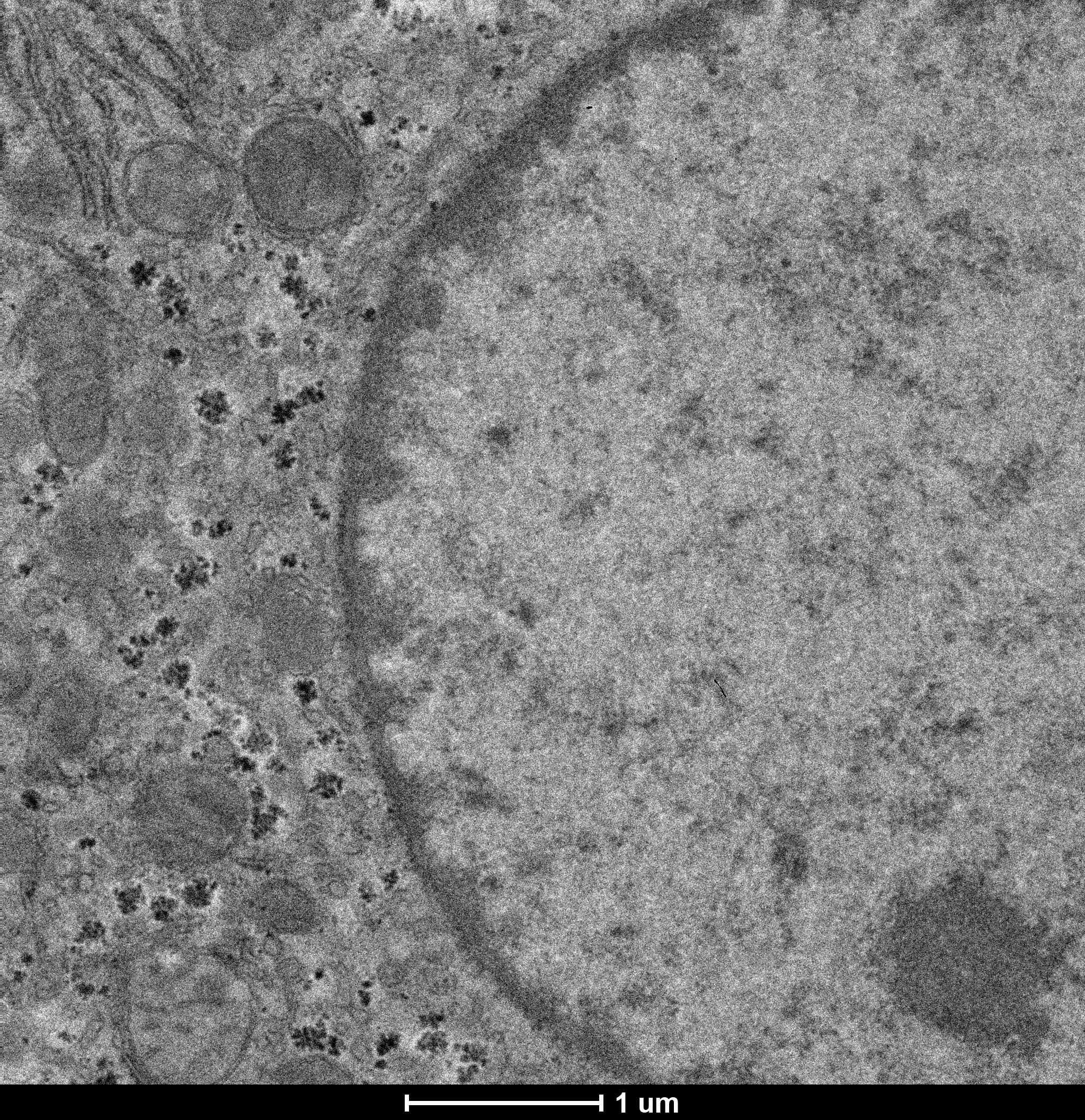

Supplement: Supplementary file 19 — Source data Fig. 1 [file 44318_2024_212_MOESM19_ESM.zip › Source Data For Figure1/1A/KO-enlarged.tif]

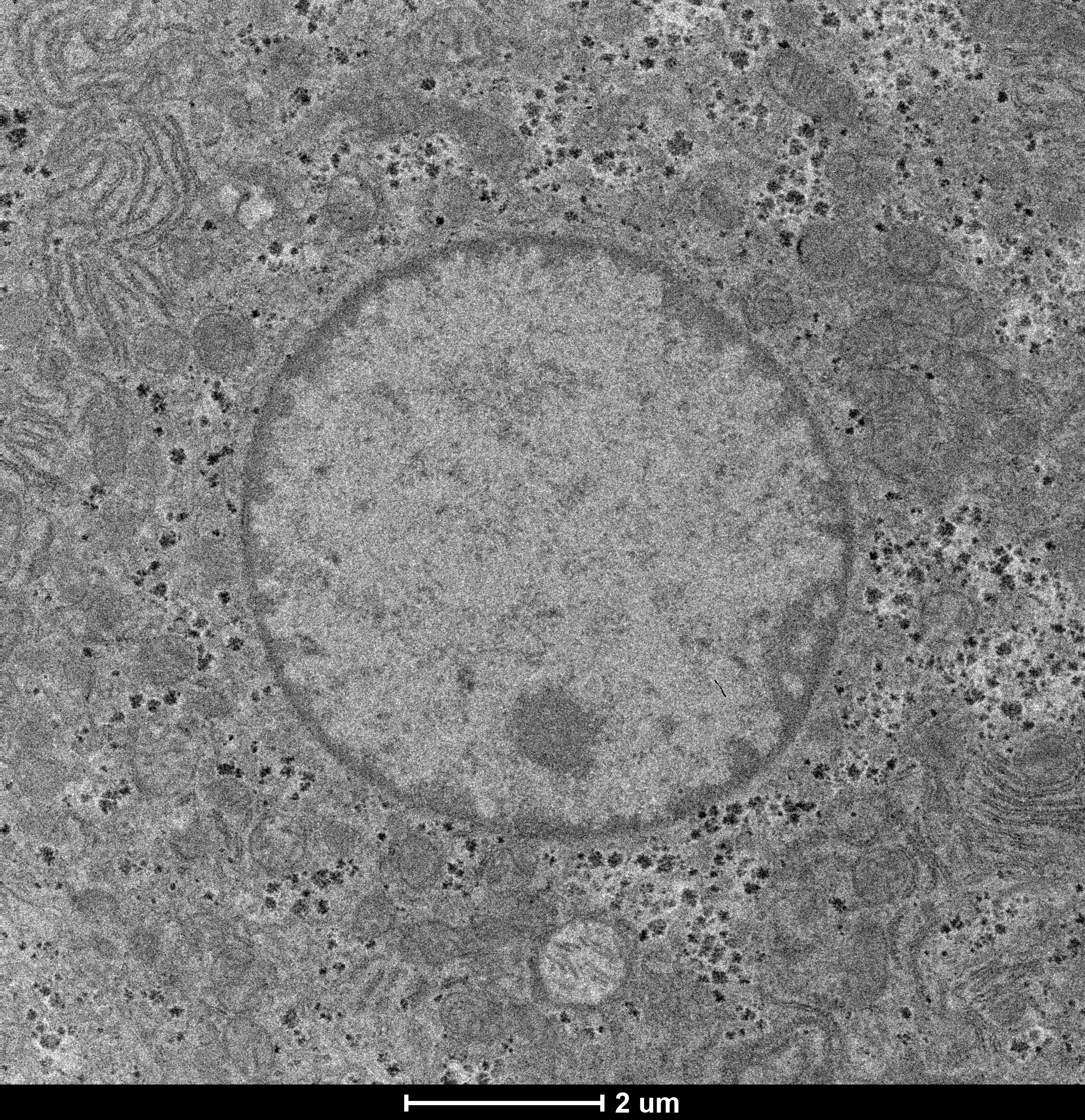

Supplement: Supplementary file 19 — Source data Fig. 1 [file 44318_2024_212_MOESM19_ESM.zip › Source Data For Figure1/1A/KO.tif]

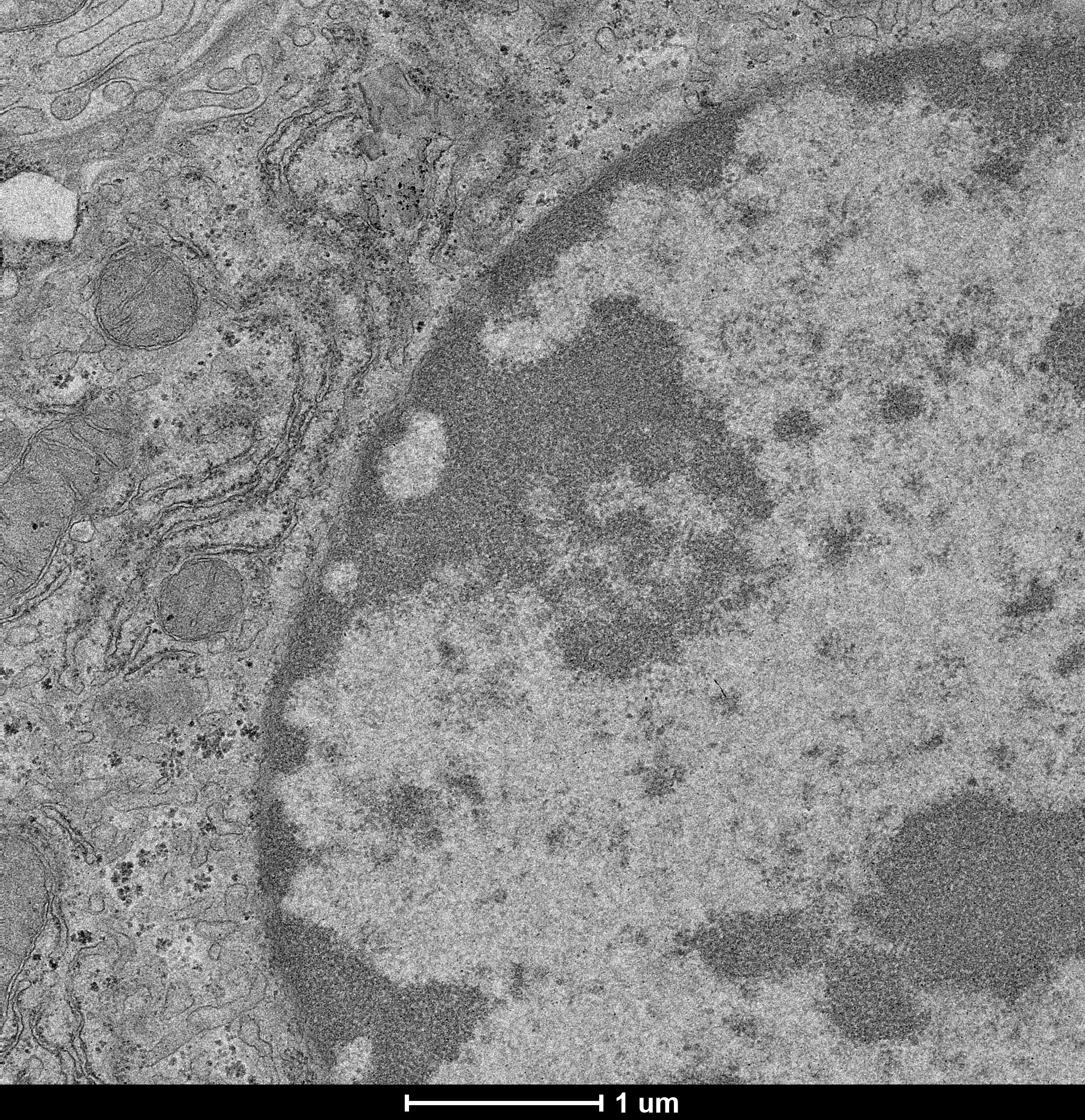

Supplement: Supplementary file 19 — Source data Fig. 1 [file 44318_2024_212_MOESM19_ESM.zip › Source Data For Figure1/1A/WT-enlarged.tif]

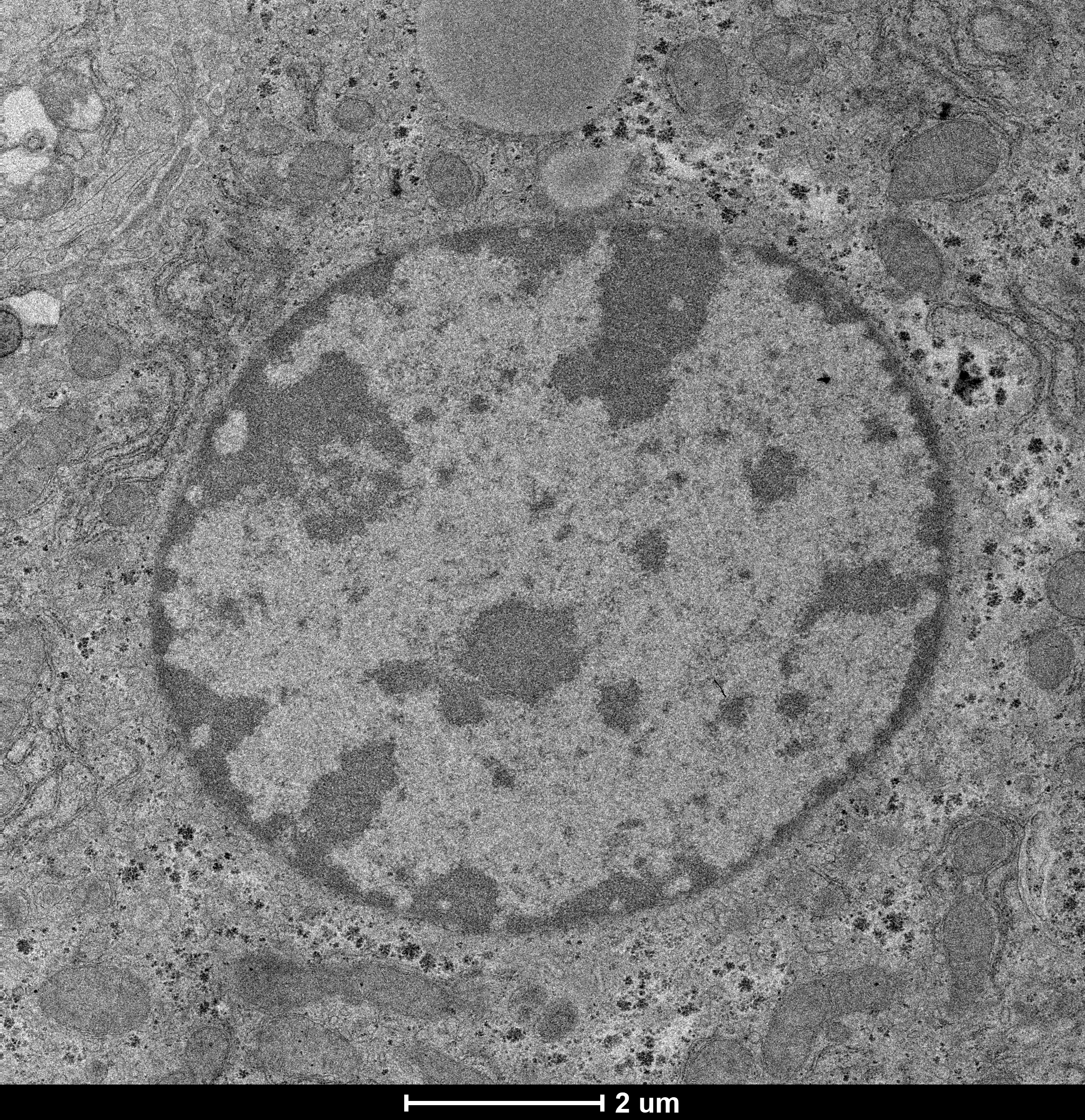

Supplement: Supplementary file 19 — Source data Fig. 1 [file 44318_2024_212_MOESM19_ESM.zip › Source Data For Figure1/1A/WT.tif]

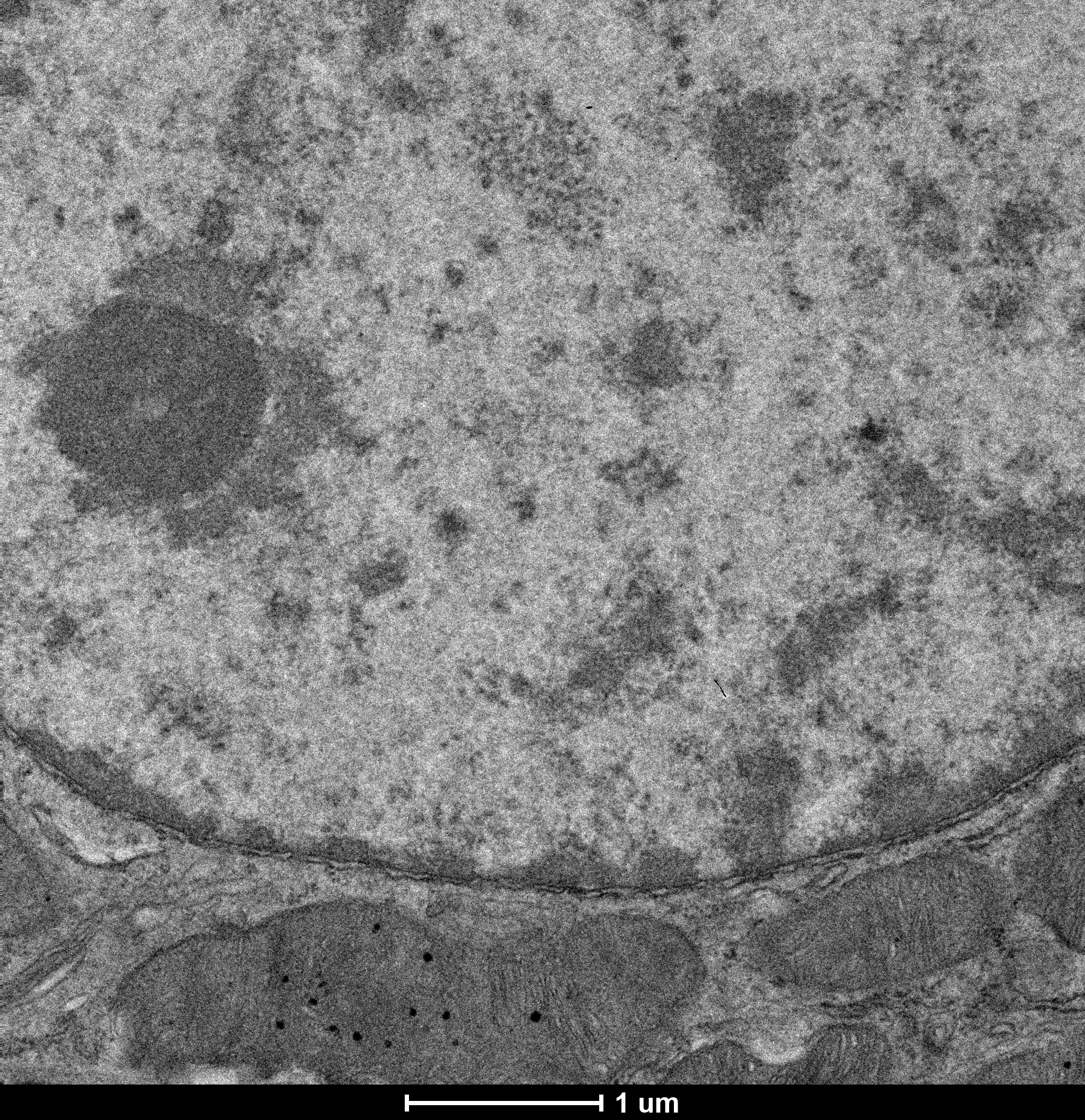

Supplement: Supplementary file 19 — Source data Fig. 1 [file 44318_2024_212_MOESM19_ESM.zip › Source Data For Figure1/1C/KO-enlarged.tif]

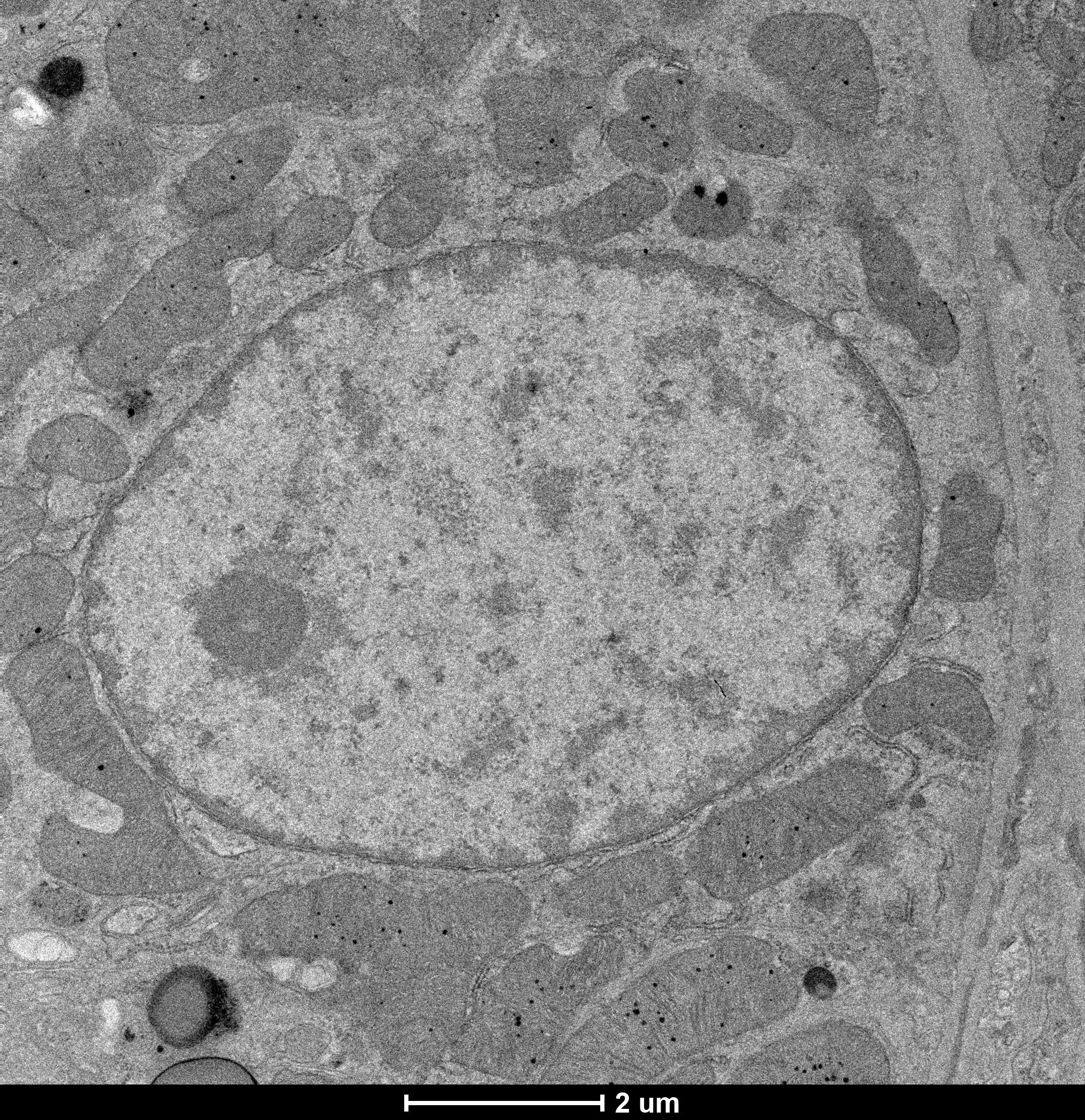

Supplement: Supplementary file 19 — Source data Fig. 1 [file 44318_2024_212_MOESM19_ESM.zip › Source Data For Figure1/1C/KO.tif]

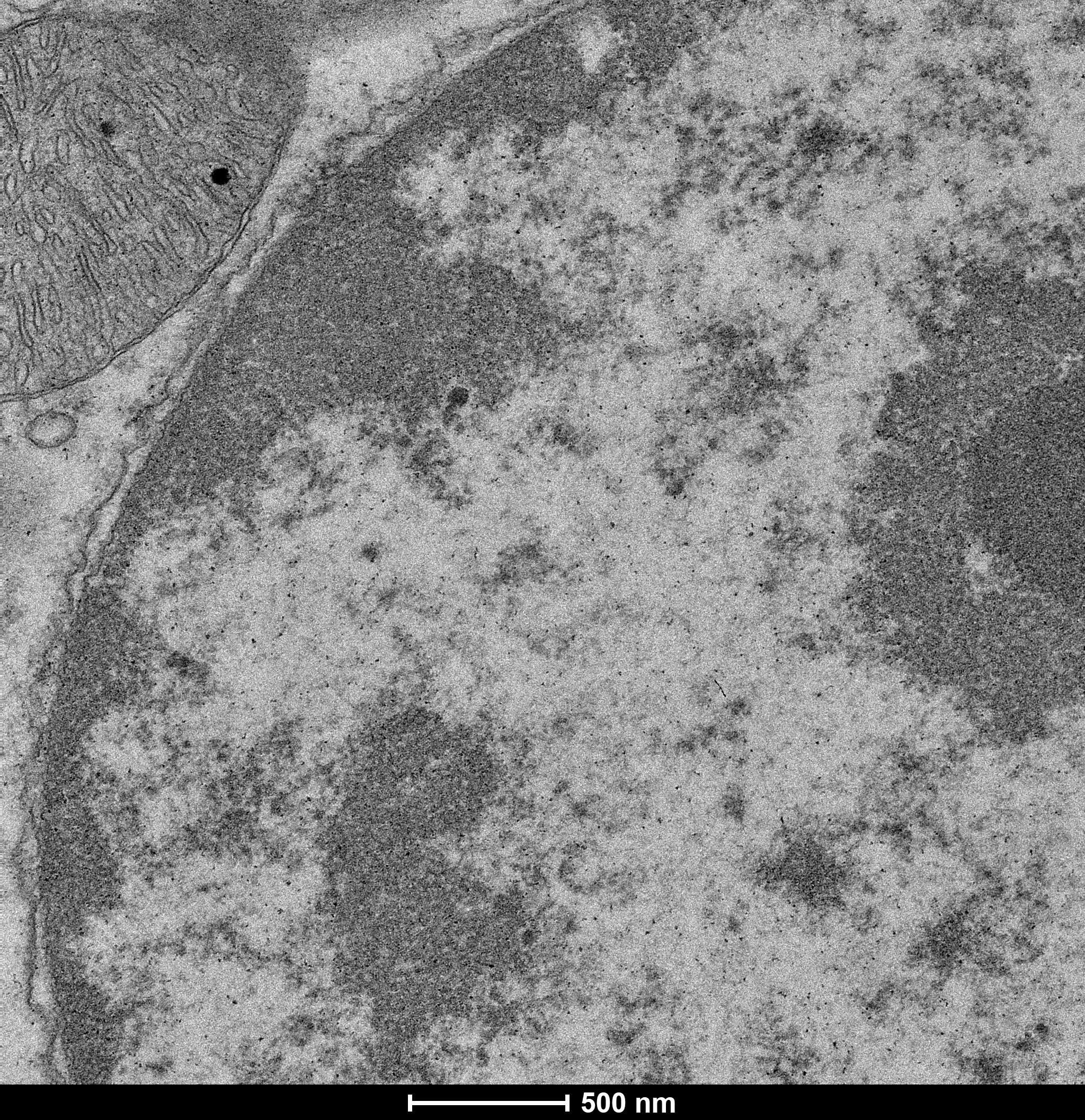

Supplement: Supplementary file 19 — Source data Fig. 1 [file 44318_2024_212_MOESM19_ESM.zip › Source Data For Figure1/1C/WT-enlarged.tif]

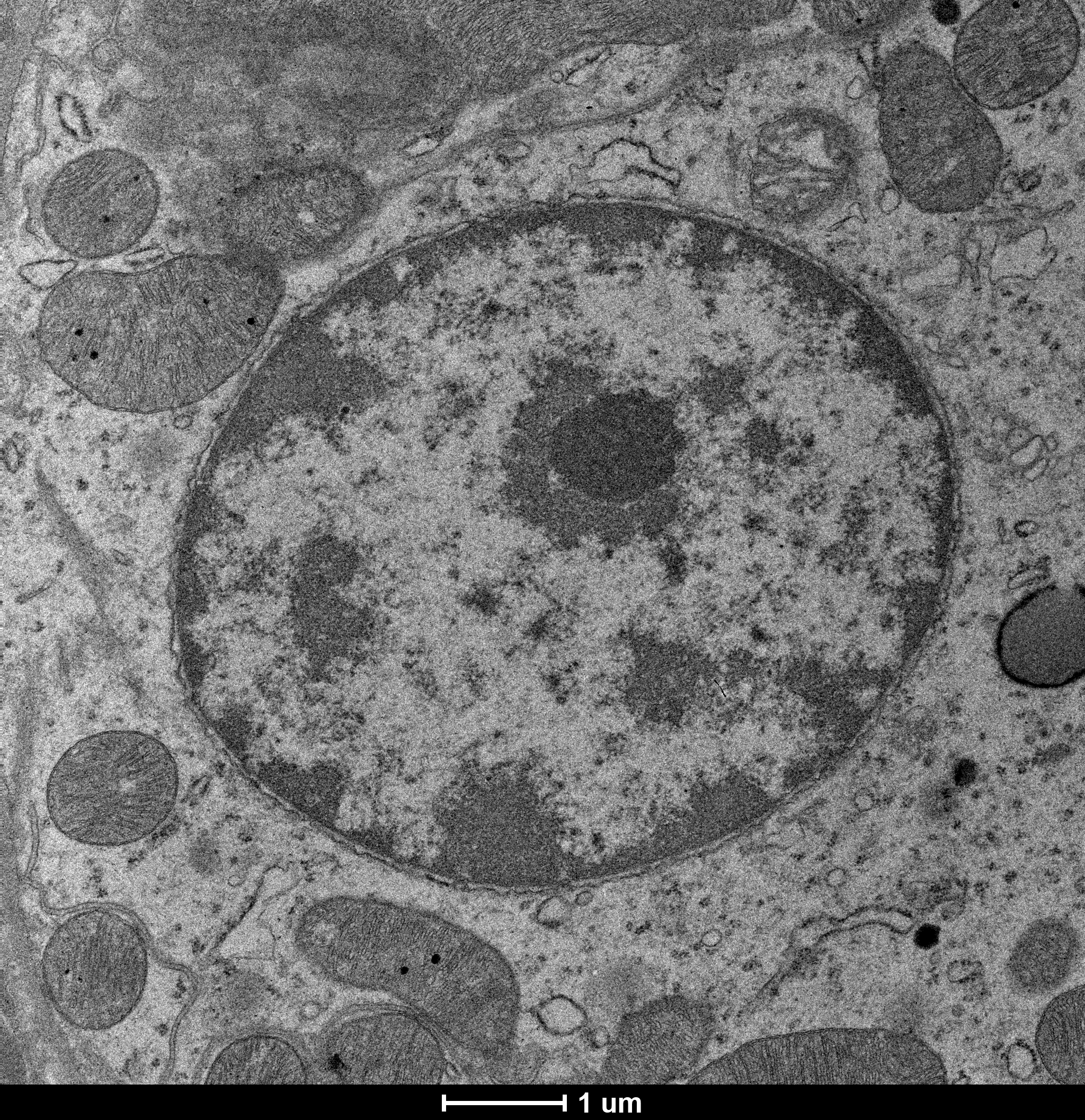

Supplement: Supplementary file 19 — Source data Fig. 1 [file 44318_2024_212_MOESM19_ESM.zip › Source Data For Figure1/1C/WT.tif]

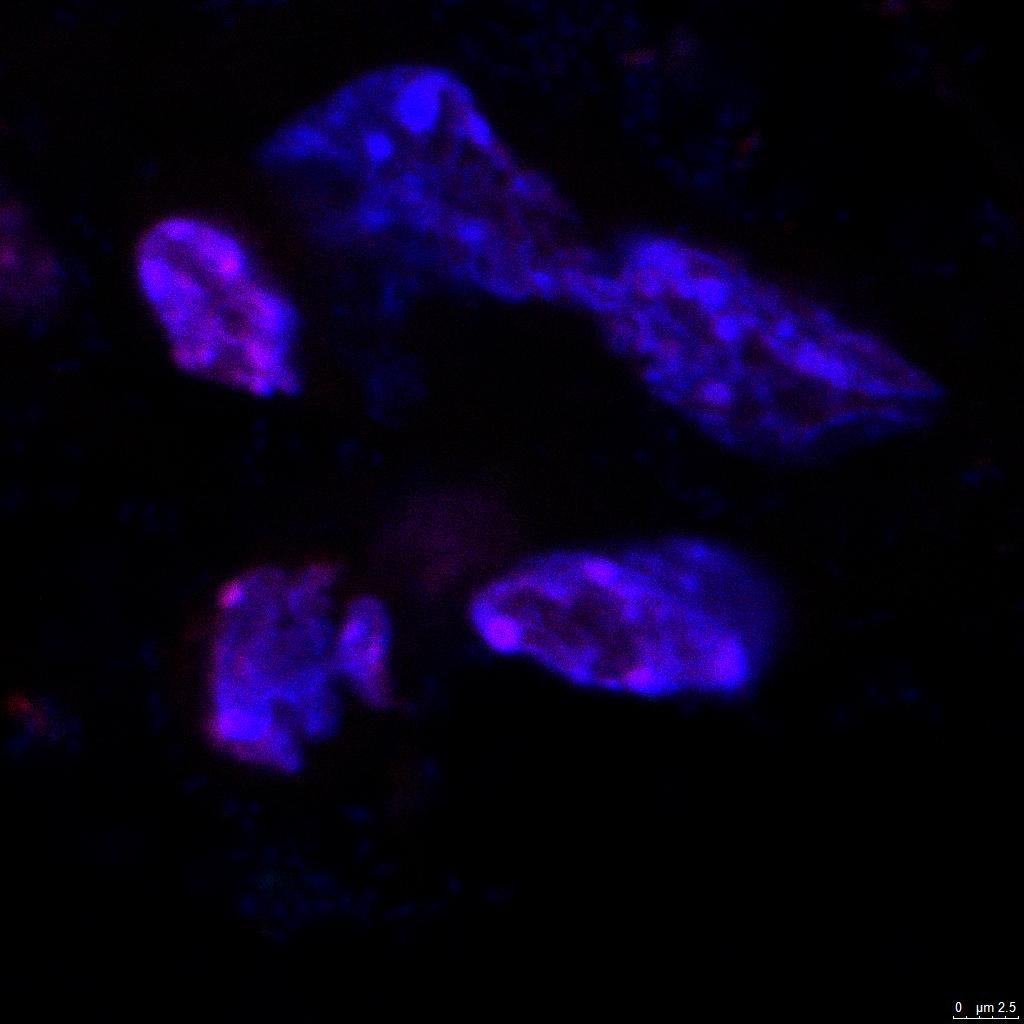

Supplement: Supplementary file 19 — Source data Fig. 1 [file 44318_2024_212_MOESM19_ESM.zip › Source Data For Figure1/1G/KO-DAPI.tif]

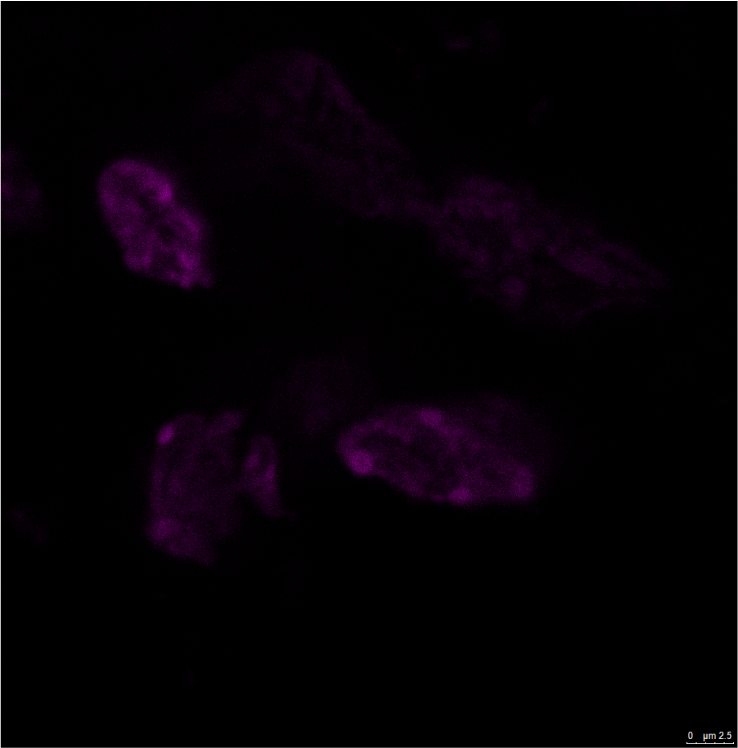

Supplement: Supplementary file 19 — Source data Fig. 1 [file 44318_2024_212_MOESM19_ESM.zip › Source Data For Figure1/1G/KO-H3K9me3.tif]

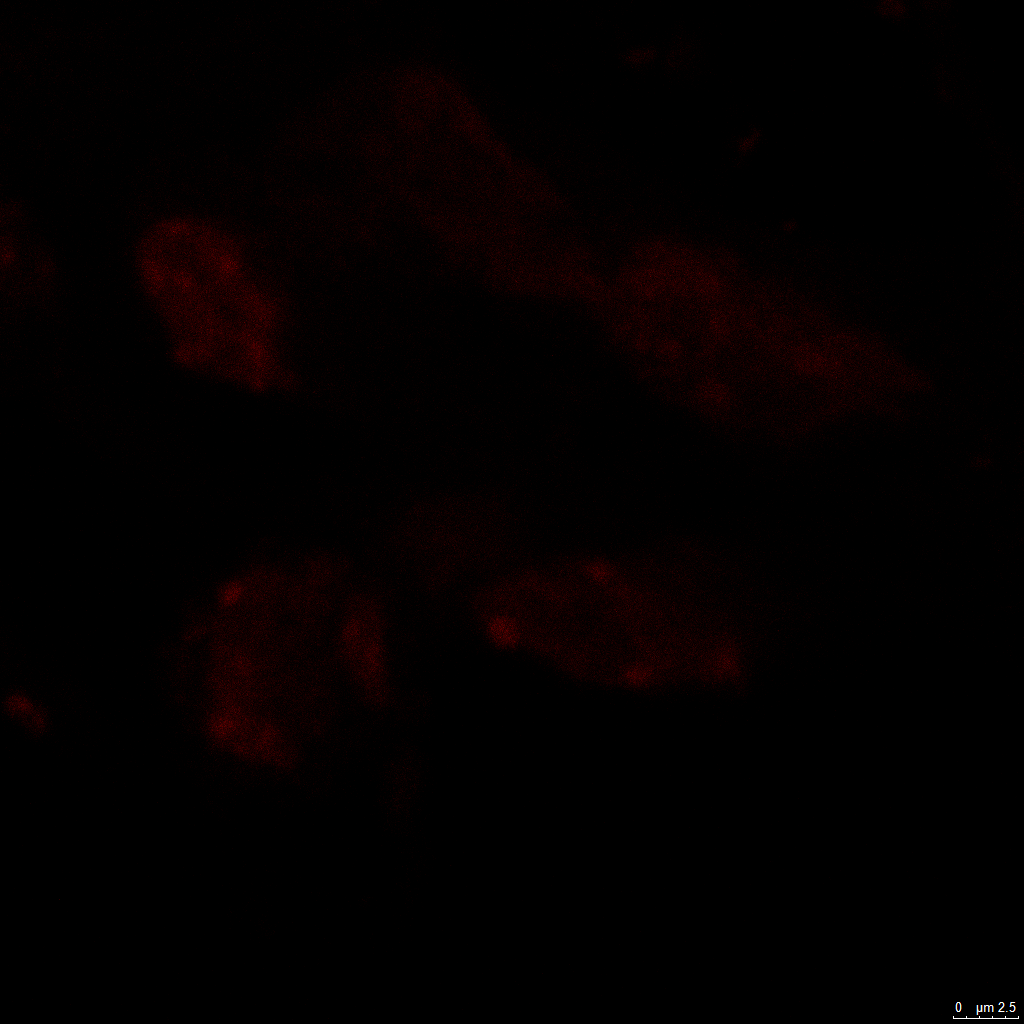

Supplement: Supplementary file 19 — Source data Fig. 1 [file 44318_2024_212_MOESM19_ESM.zip › Source Data For Figure1/1G/KO-HP1α.tif]

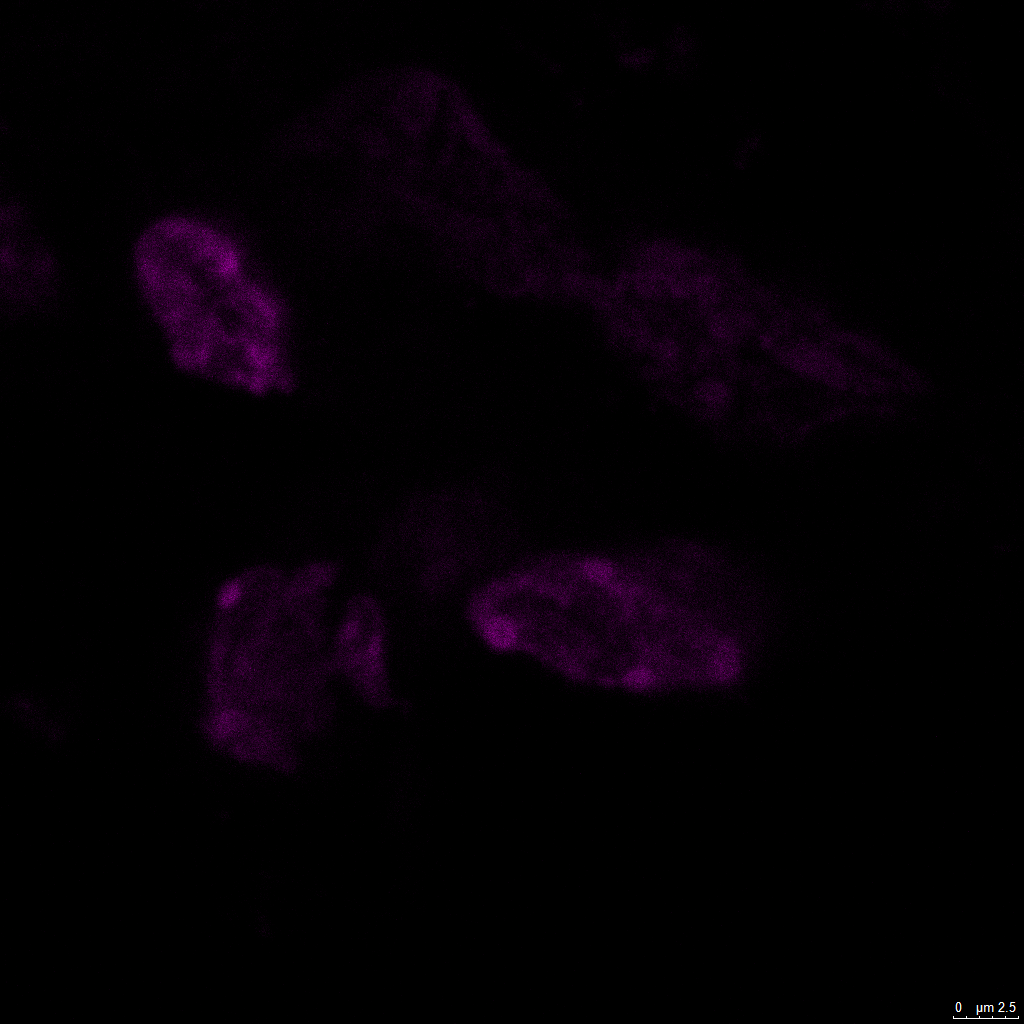

Supplement: Supplementary file 19 — Source data Fig. 1 [file 44318_2024_212_MOESM19_ESM.zip › Source Data For Figure1/1G/KO-IRTKS.tif]

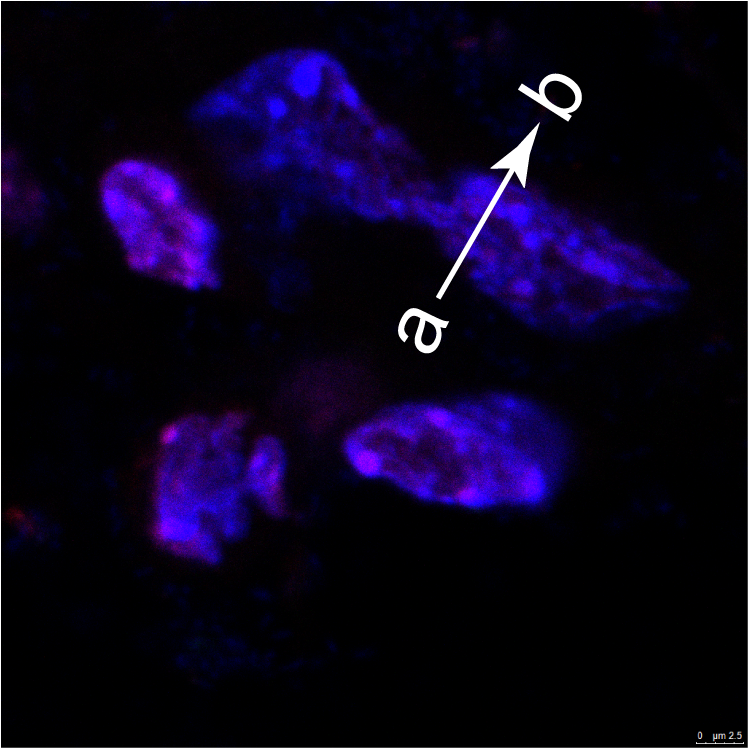

Supplement: Supplementary file 19 — Source data Fig. 1 [file 44318_2024_212_MOESM19_ESM.zip › Source Data For Figure1/1G/KO-Merge-01.tif]

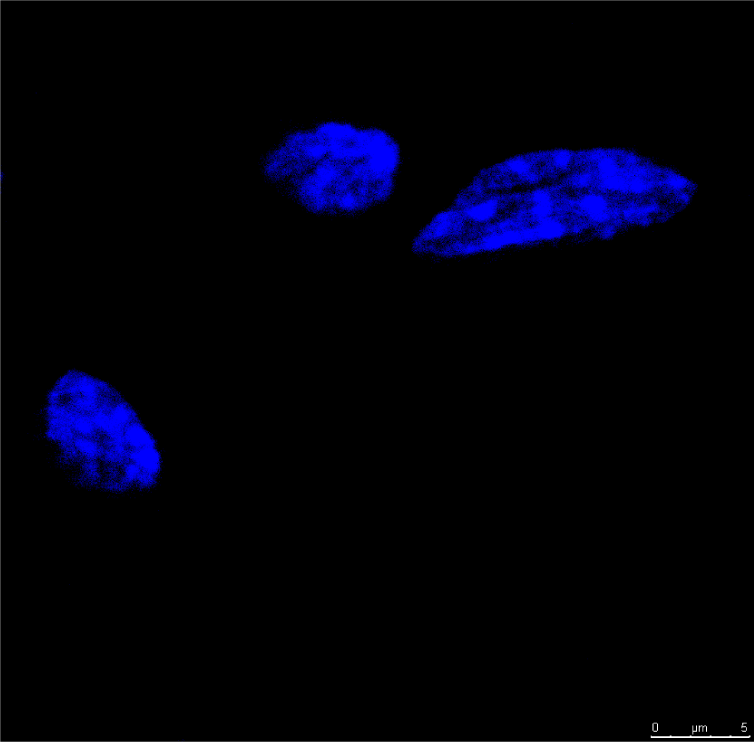

Supplement: Supplementary file 19 — Source data Fig. 1 [file 44318_2024_212_MOESM19_ESM.zip › Source Data For Figure1/1G/WT-DAPI.tif]

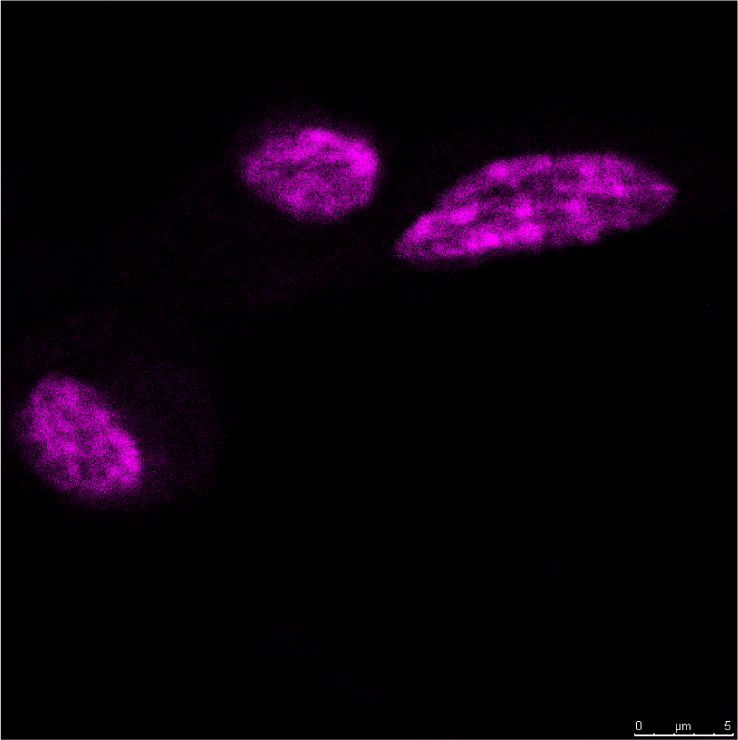

Supplement: Supplementary file 19 — Source data Fig. 1 [file 44318_2024_212_MOESM19_ESM.zip › Source Data For Figure1/1G/WT-H3K9me3.tif]

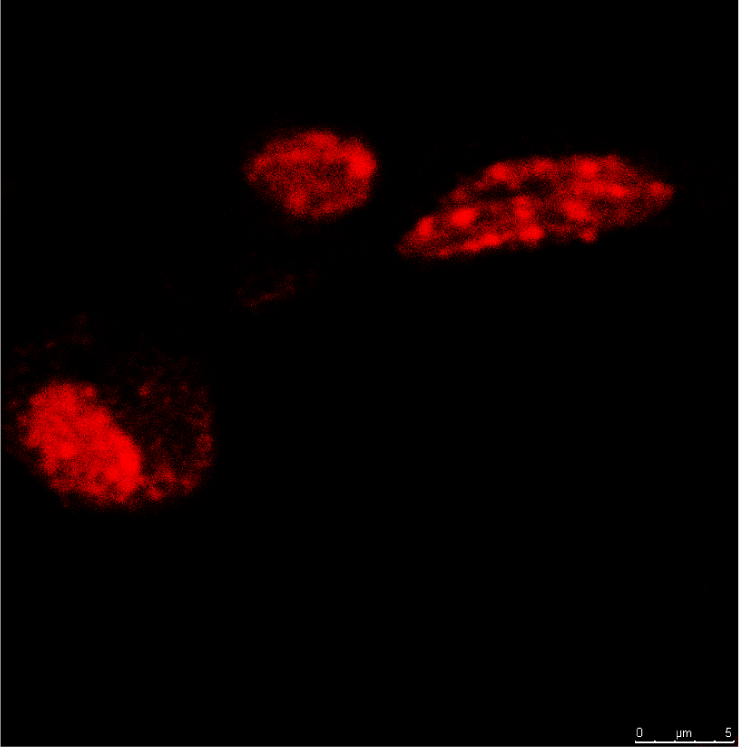

Supplement: Supplementary file 19 — Source data Fig. 1 [file 44318_2024_212_MOESM19_ESM.zip › Source Data For Figure1/1G/WT-HP1α.tif]

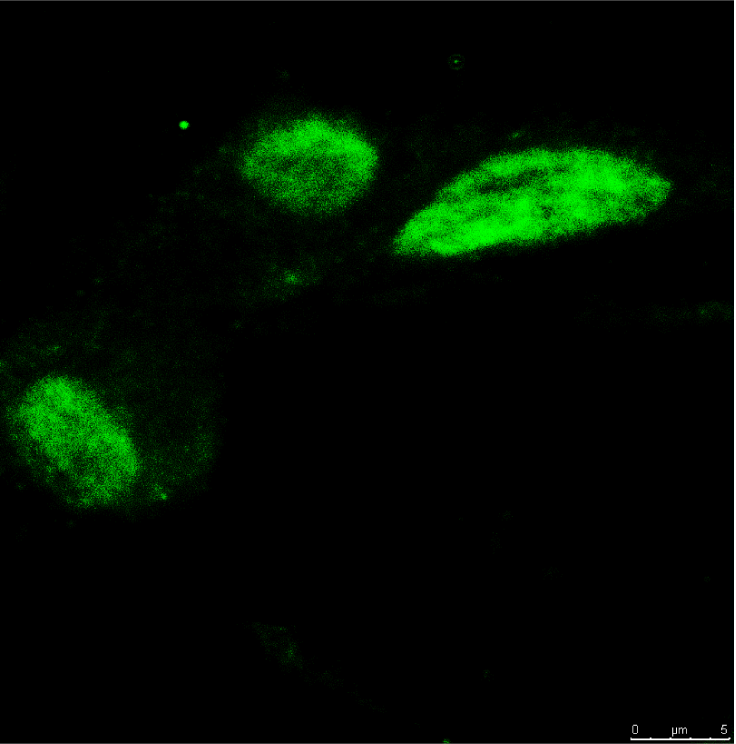

Supplement: Supplementary file 19 — Source data Fig. 1 [file 44318_2024_212_MOESM19_ESM.zip › Source Data For Figure1/1G/WT-IRTKS.tif]

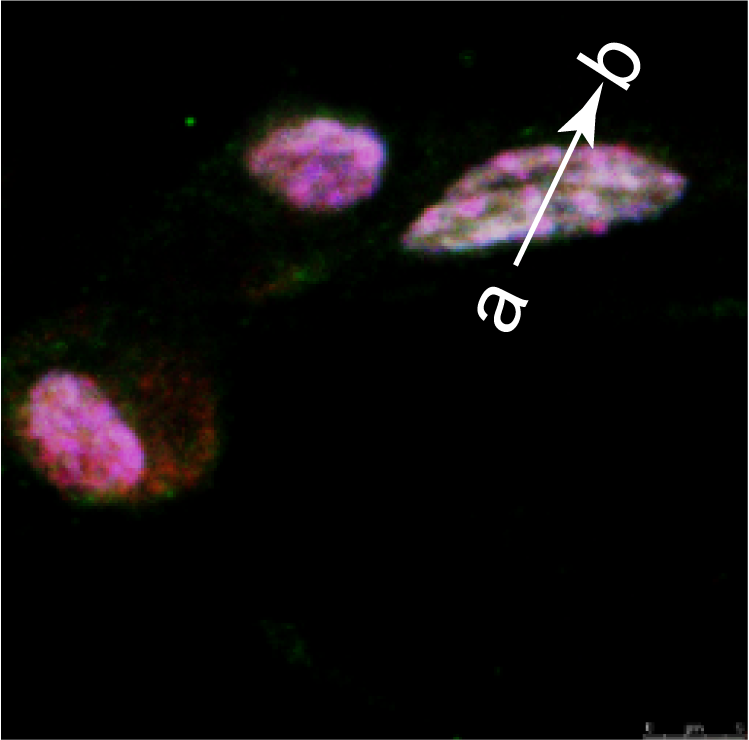

Supplement: Supplementary file 19 — Source data Fig. 1 [file 44318_2024_212_MOESM19_ESM.zip › Source Data For Figure1/1G/WT-Merge.tif]

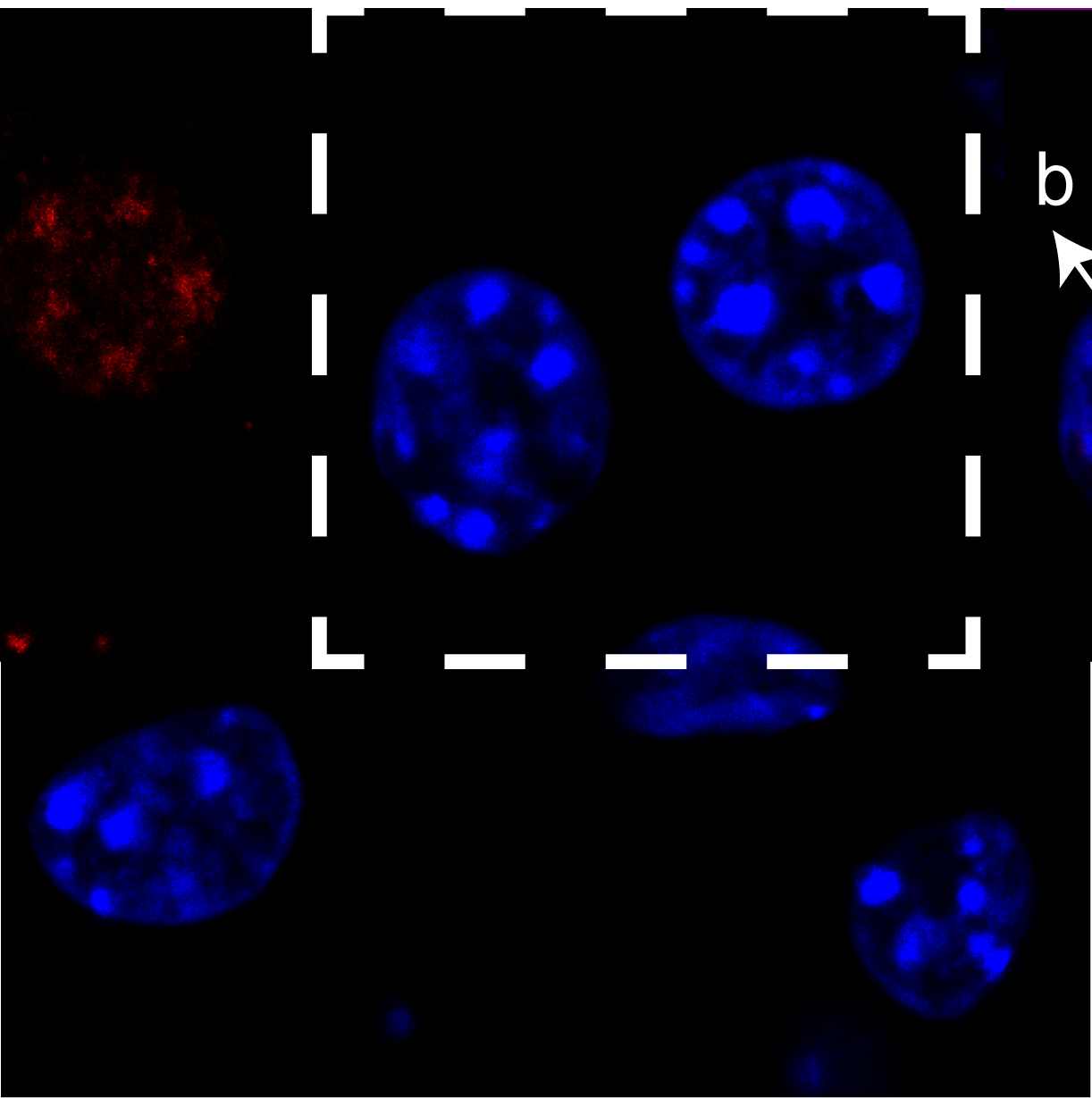

Supplement: Supplementary file 19 — Source data Fig. 1 [file 44318_2024_212_MOESM19_ESM.zip › Source Data For Figure1/1I/KO-DAPI.tif]

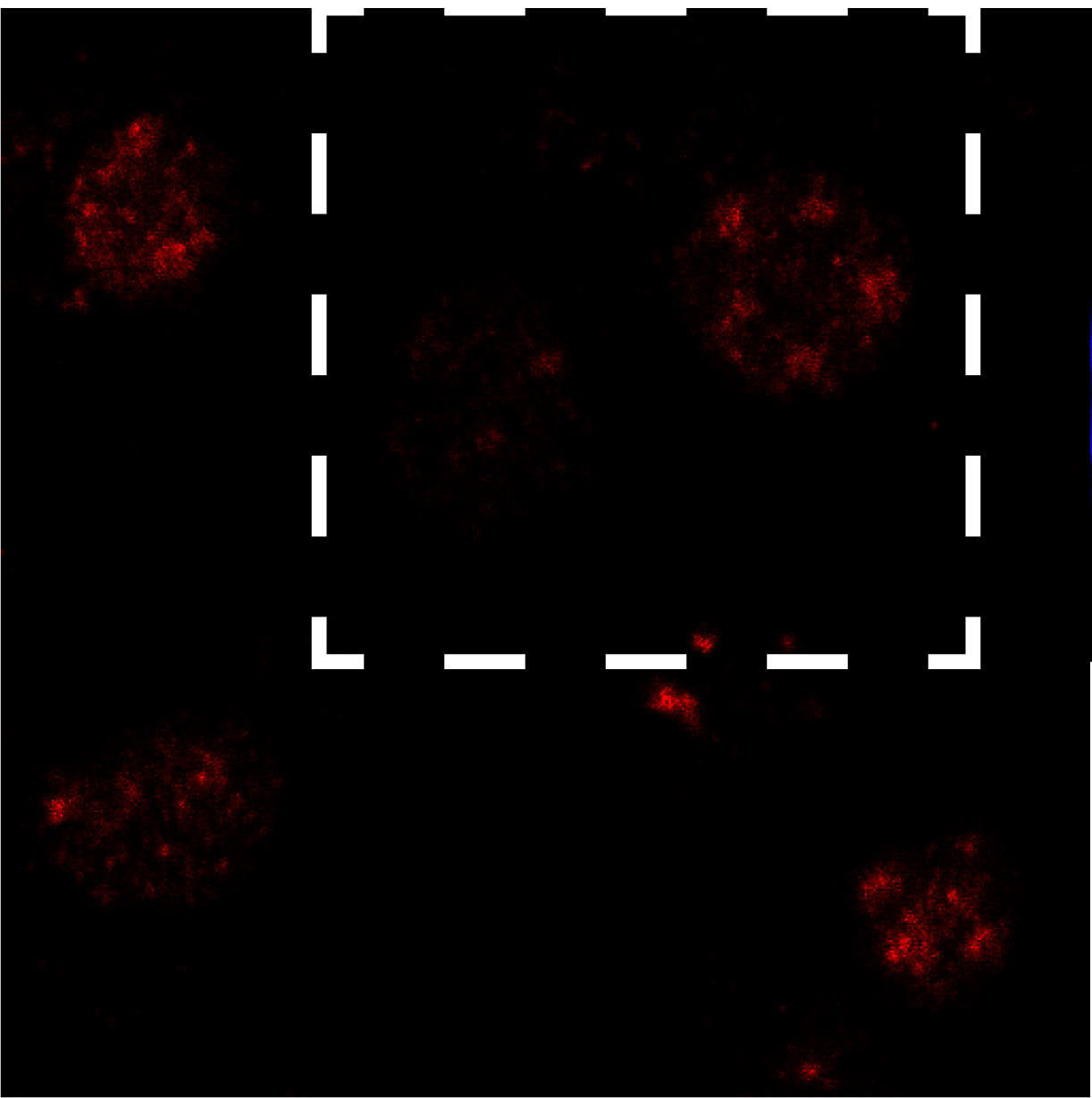

Supplement: Supplementary file 19 — Source data Fig. 1 [file 44318_2024_212_MOESM19_ESM.zip › Source Data For Figure1/1I/KO-HP1α.tif]

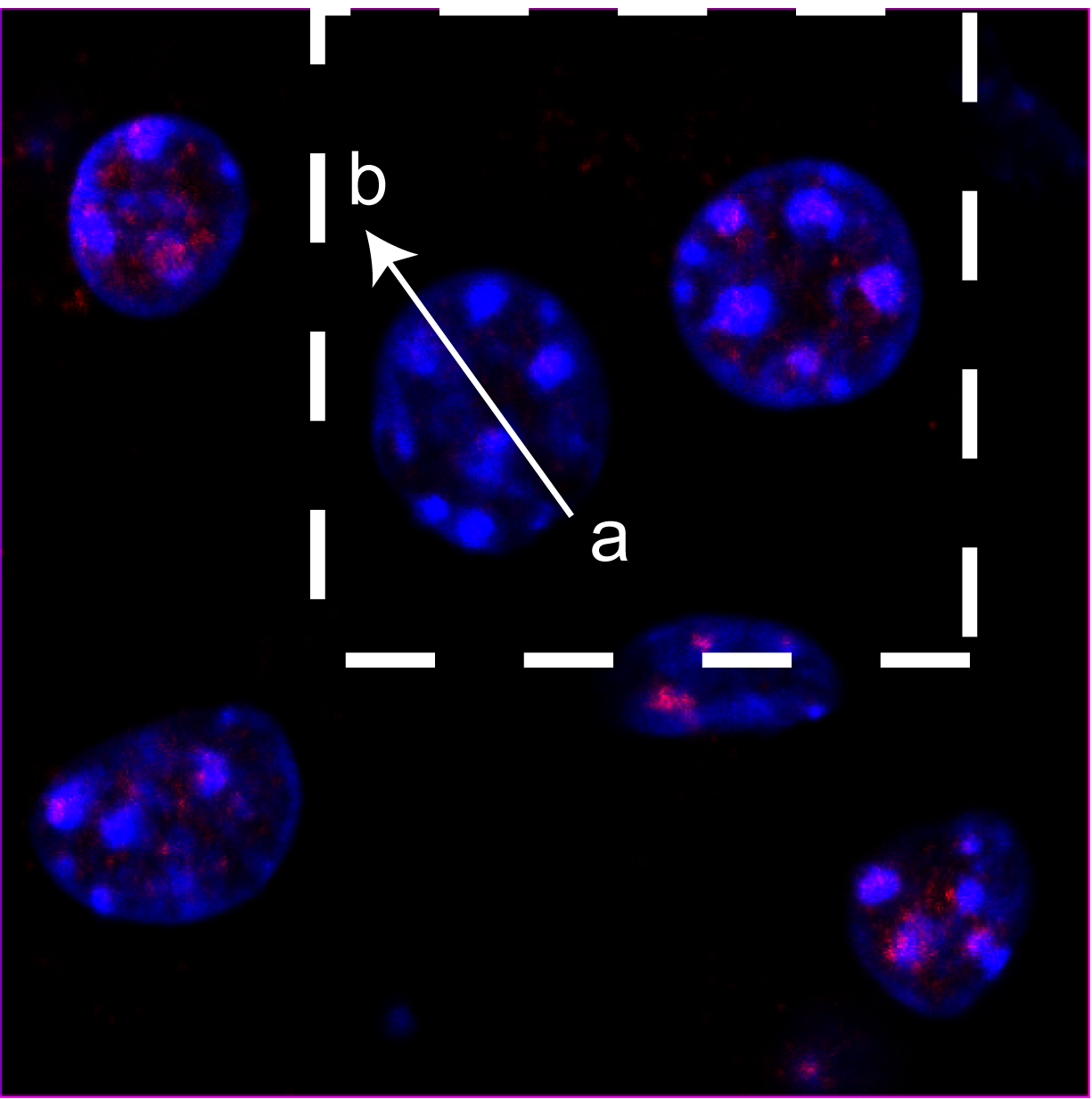

Supplement: Supplementary file 19 — Source data Fig. 1 [file 44318_2024_212_MOESM19_ESM.zip › Source Data For Figure1/1I/KO-Merge.tif]

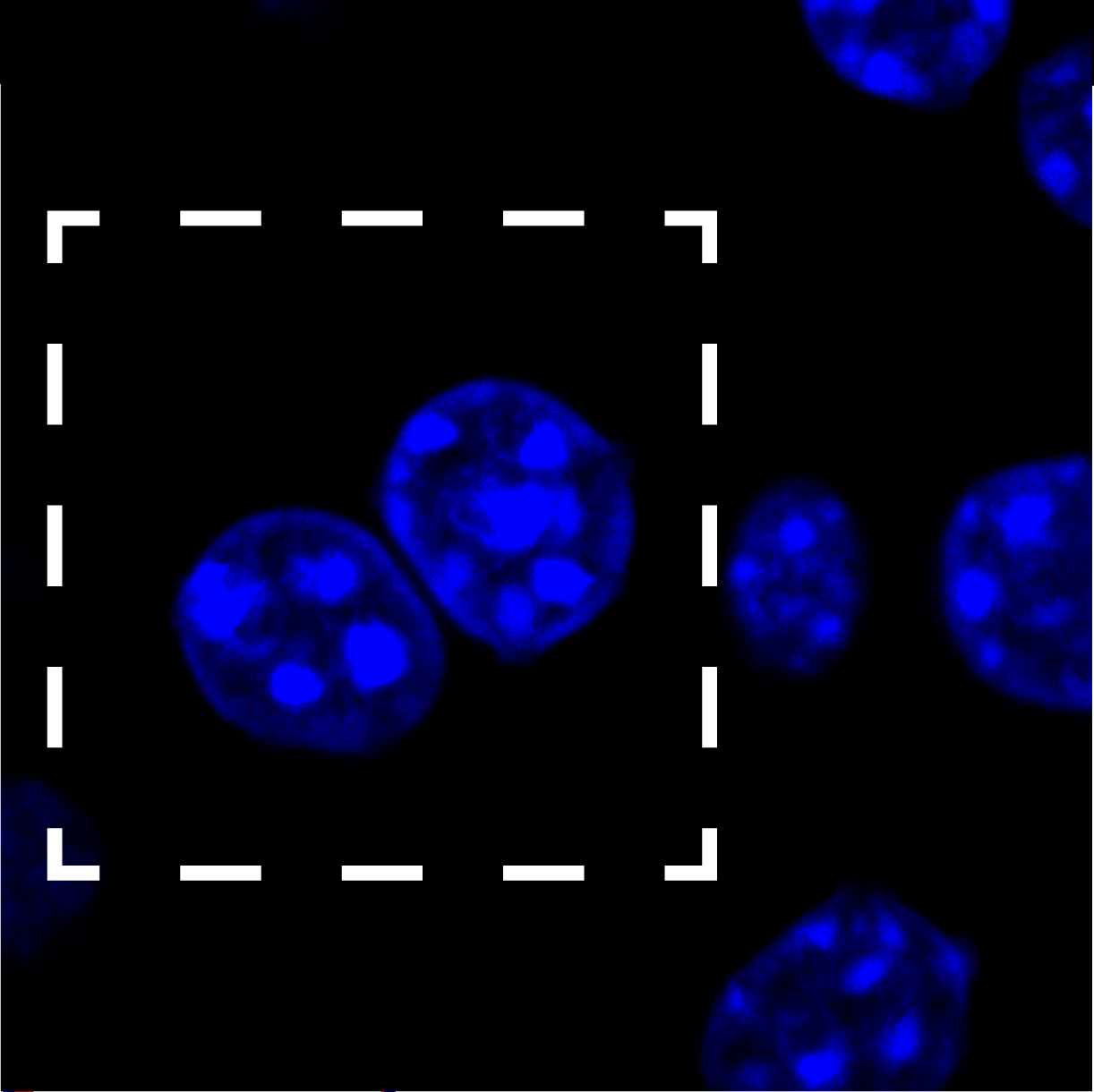

Supplement: Supplementary file 19 — Source data Fig. 1 [file 44318_2024_212_MOESM19_ESM.zip › Source Data For Figure1/1I/WT-DAPI.tif]

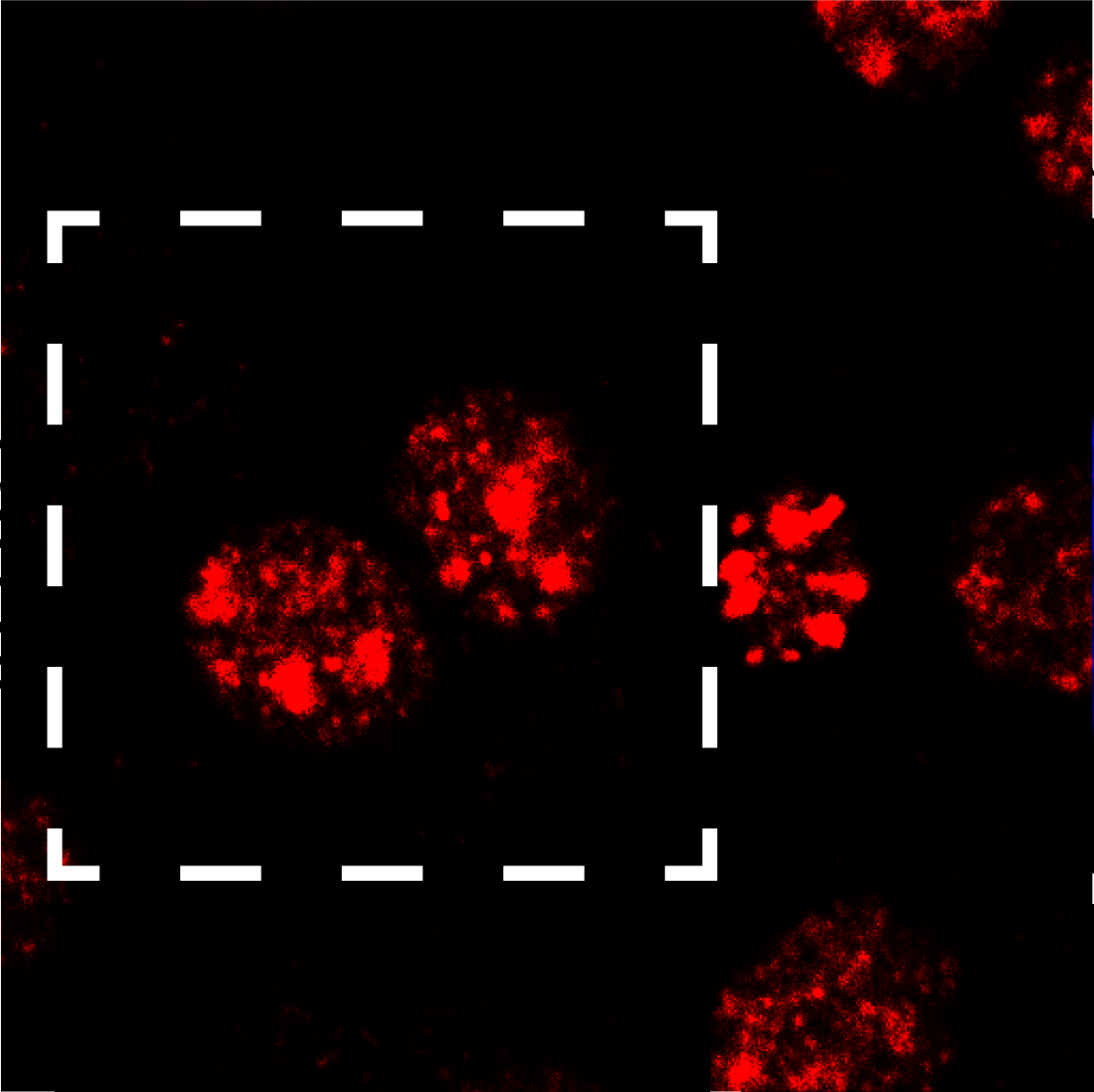

Supplement: Supplementary file 19 — Source data Fig. 1 [file 44318_2024_212_MOESM19_ESM.zip › Source Data For Figure1/1I/WT-HP1α.tif]

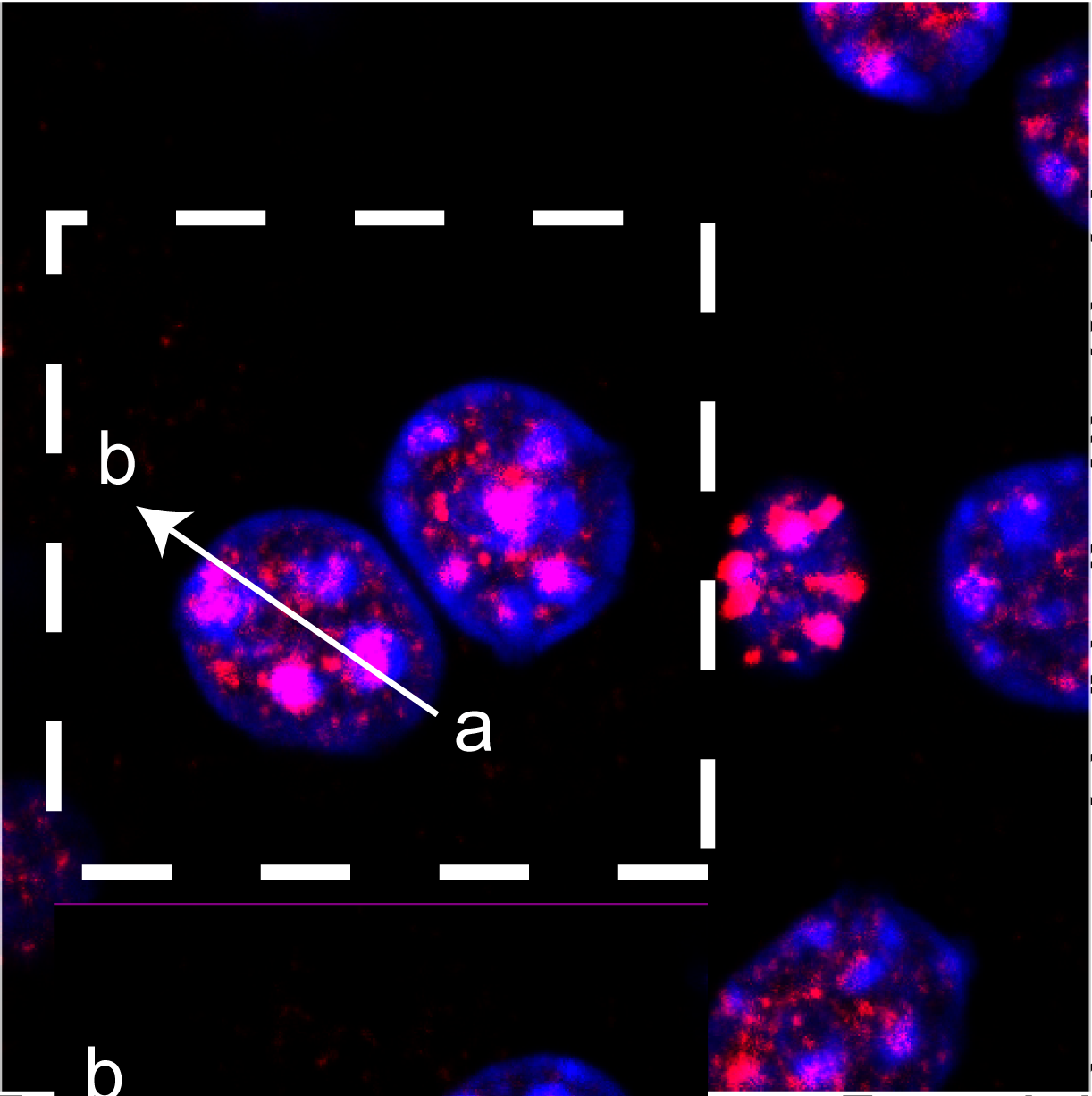

Supplement: Supplementary file 19 — Source data Fig. 1 [file 44318_2024_212_MOESM19_ESM.zip › Source Data For Figure1/1I/WT-Merge.tif]

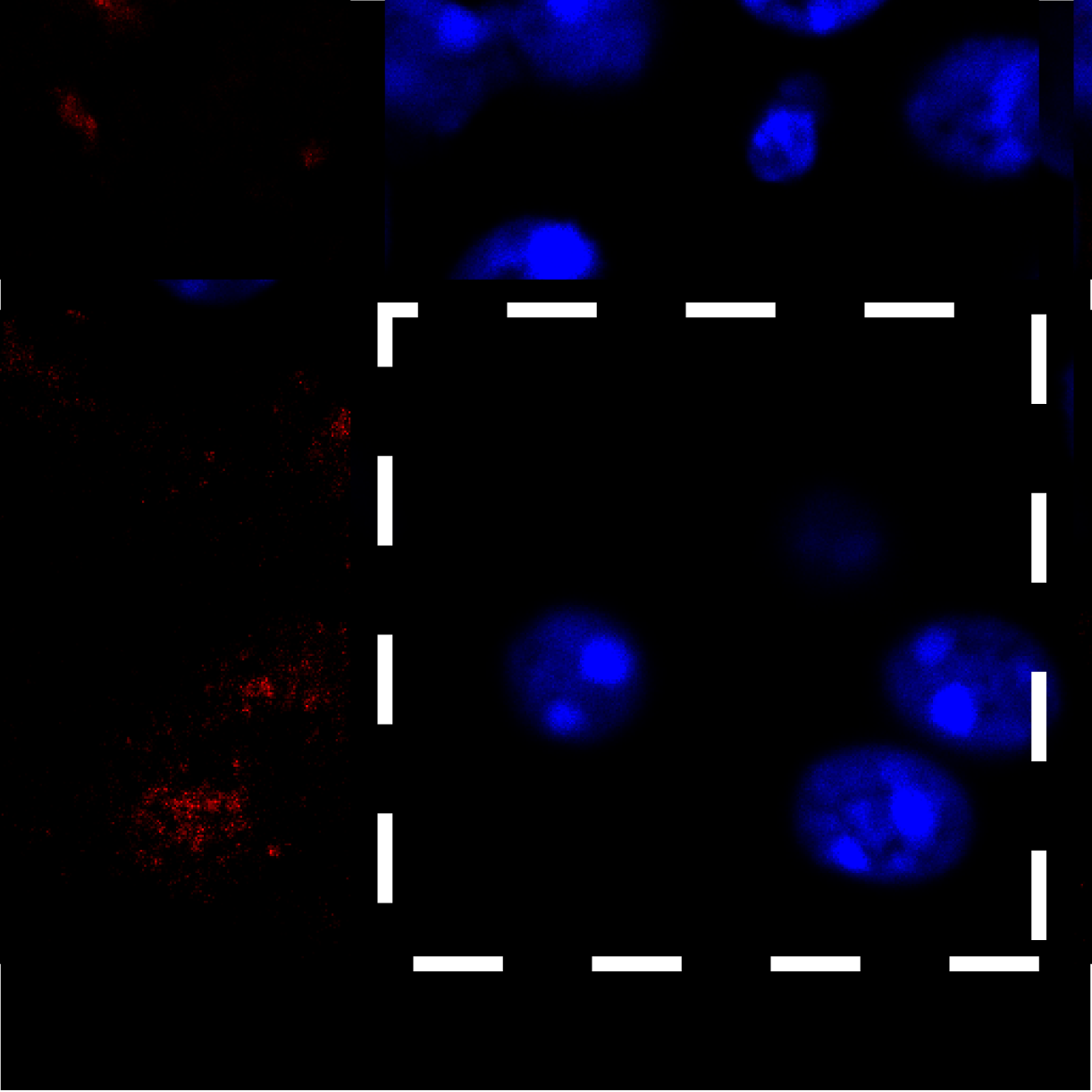

Supplement: Supplementary file 19 — Source data Fig. 1 [file 44318_2024_212_MOESM19_ESM.zip › Source Data For Figure1/1K/KO-DAPI.tif]

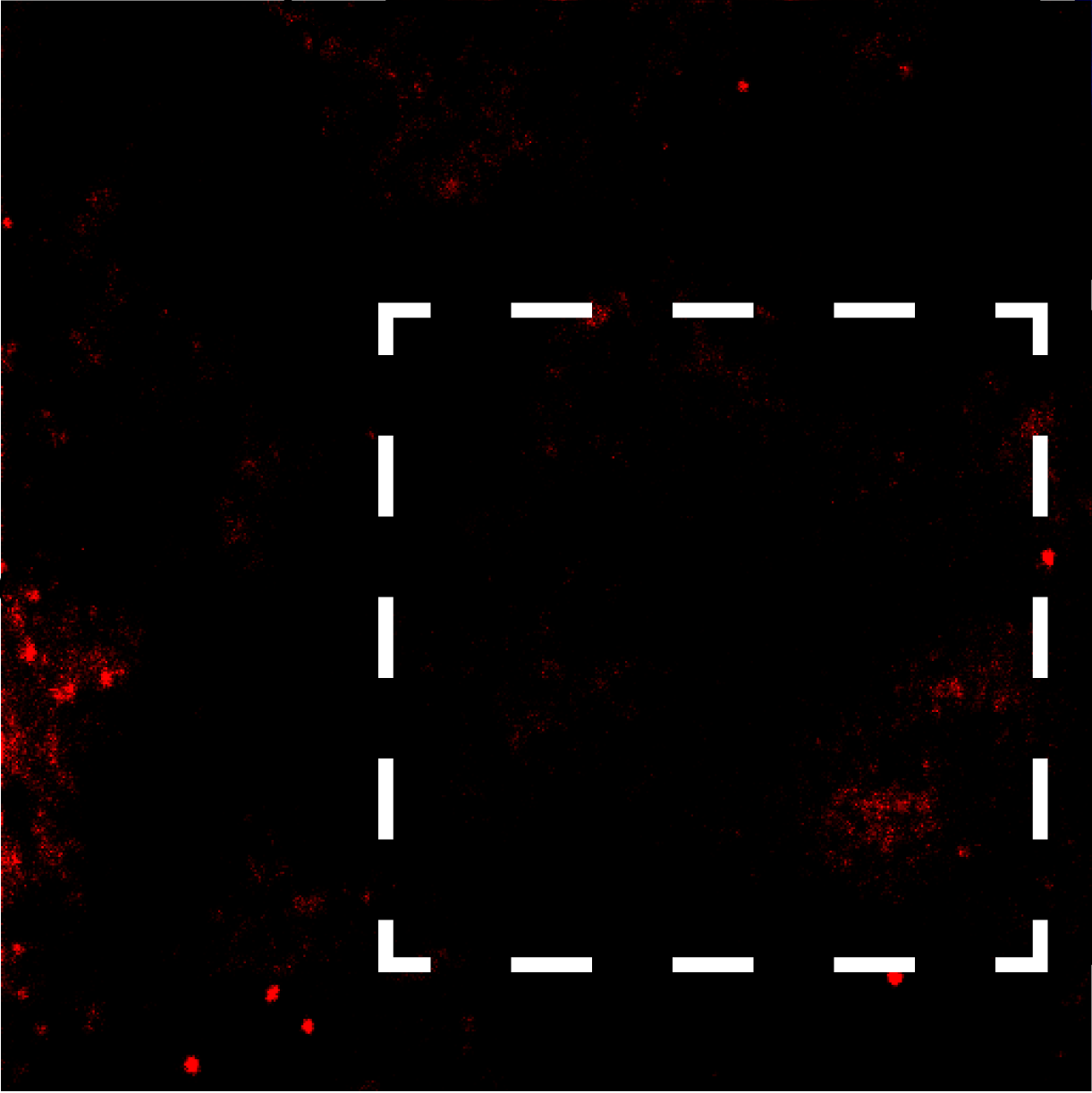

Supplement: Supplementary file 19 — Source data Fig. 1 [file 44318_2024_212_MOESM19_ESM.zip › Source Data For Figure1/1K/KO-HP1α.tif]

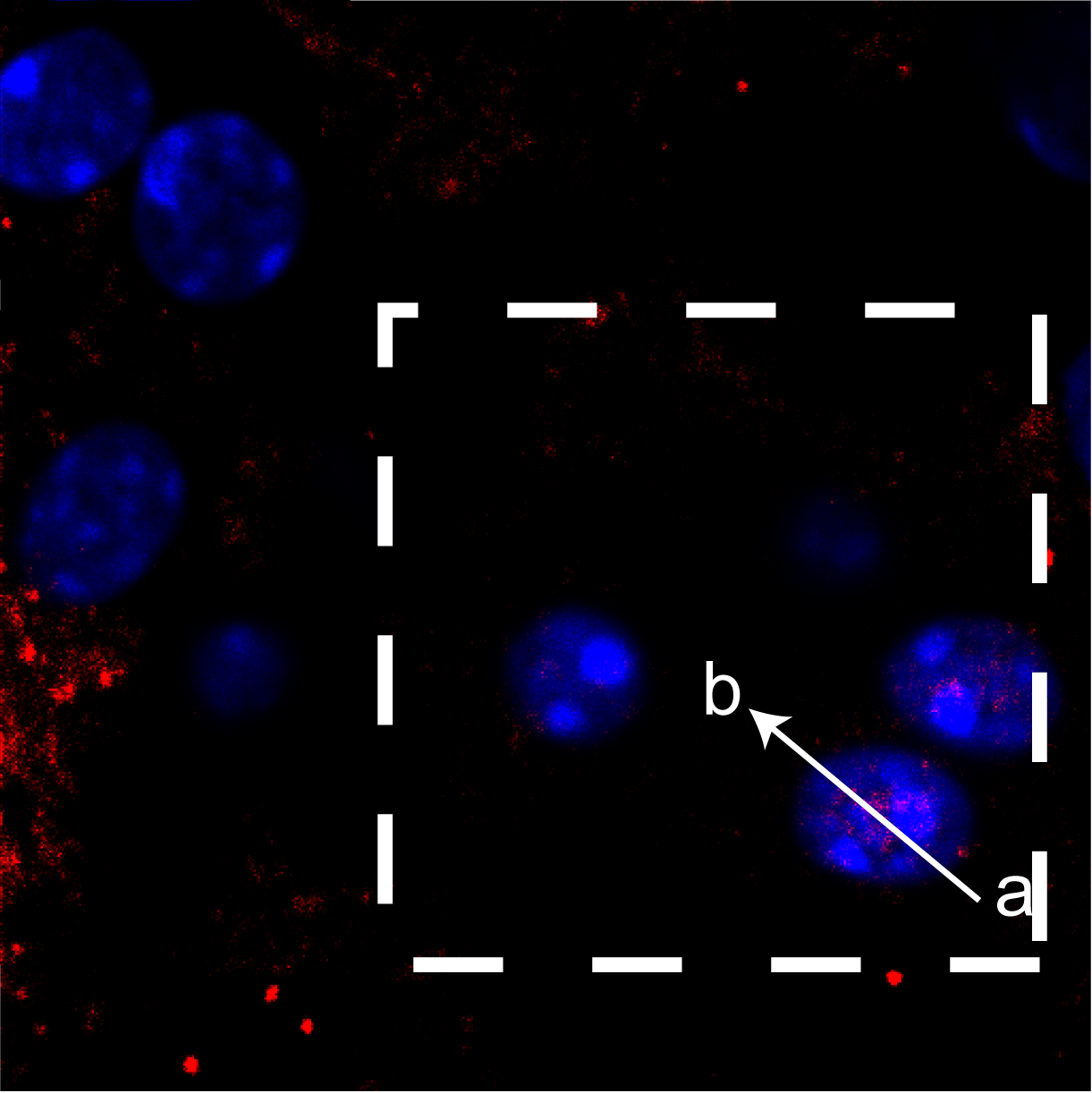

Supplement: Supplementary file 19 — Source data Fig. 1 [file 44318_2024_212_MOESM19_ESM.zip › Source Data For Figure1/1K/KO-Merge-01.tif]

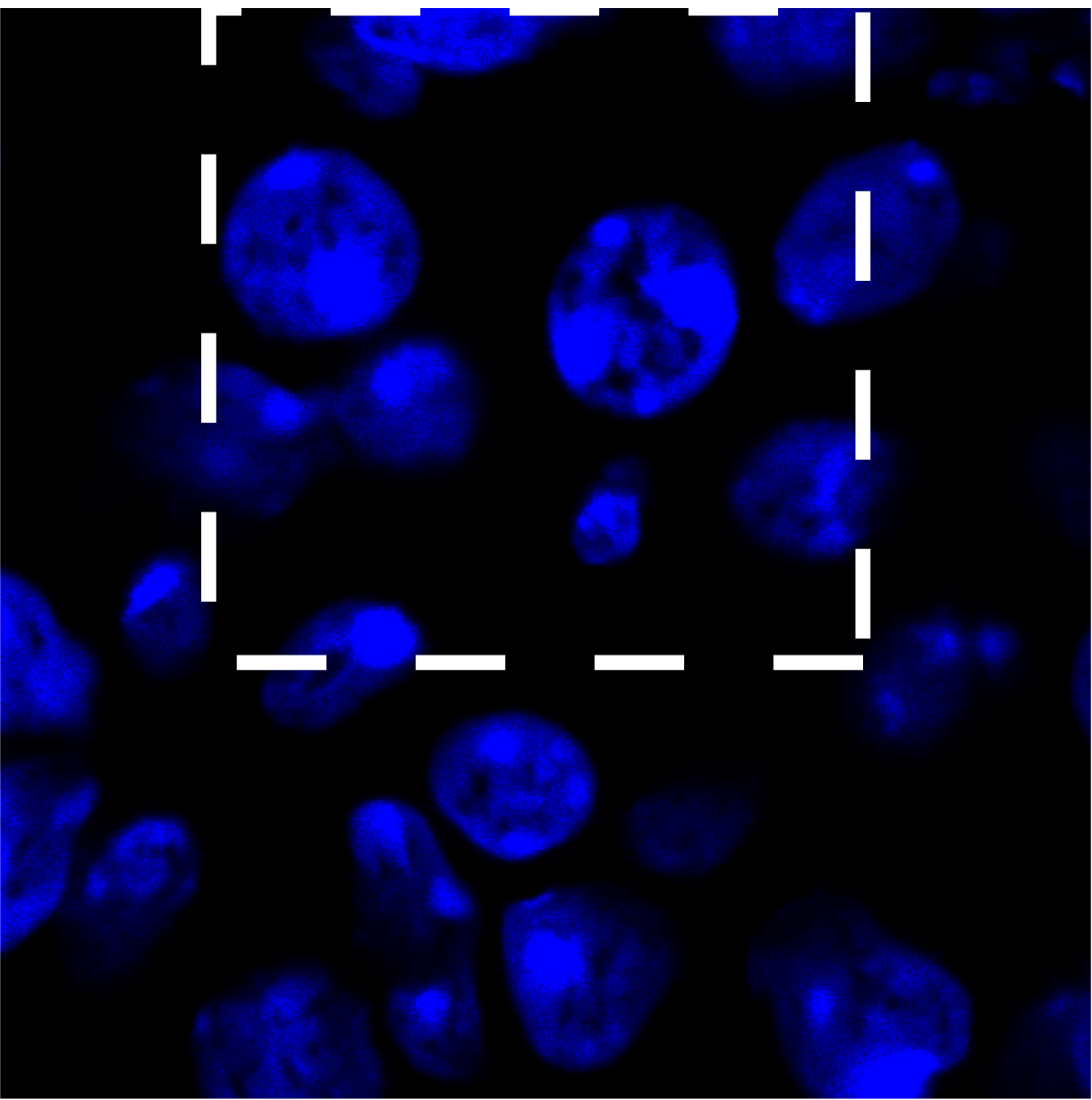

Supplement: Supplementary file 19 — Source data Fig. 1 [file 44318_2024_212_MOESM19_ESM.zip › Source Data For Figure1/1K/WT-DAPI-01.tif]

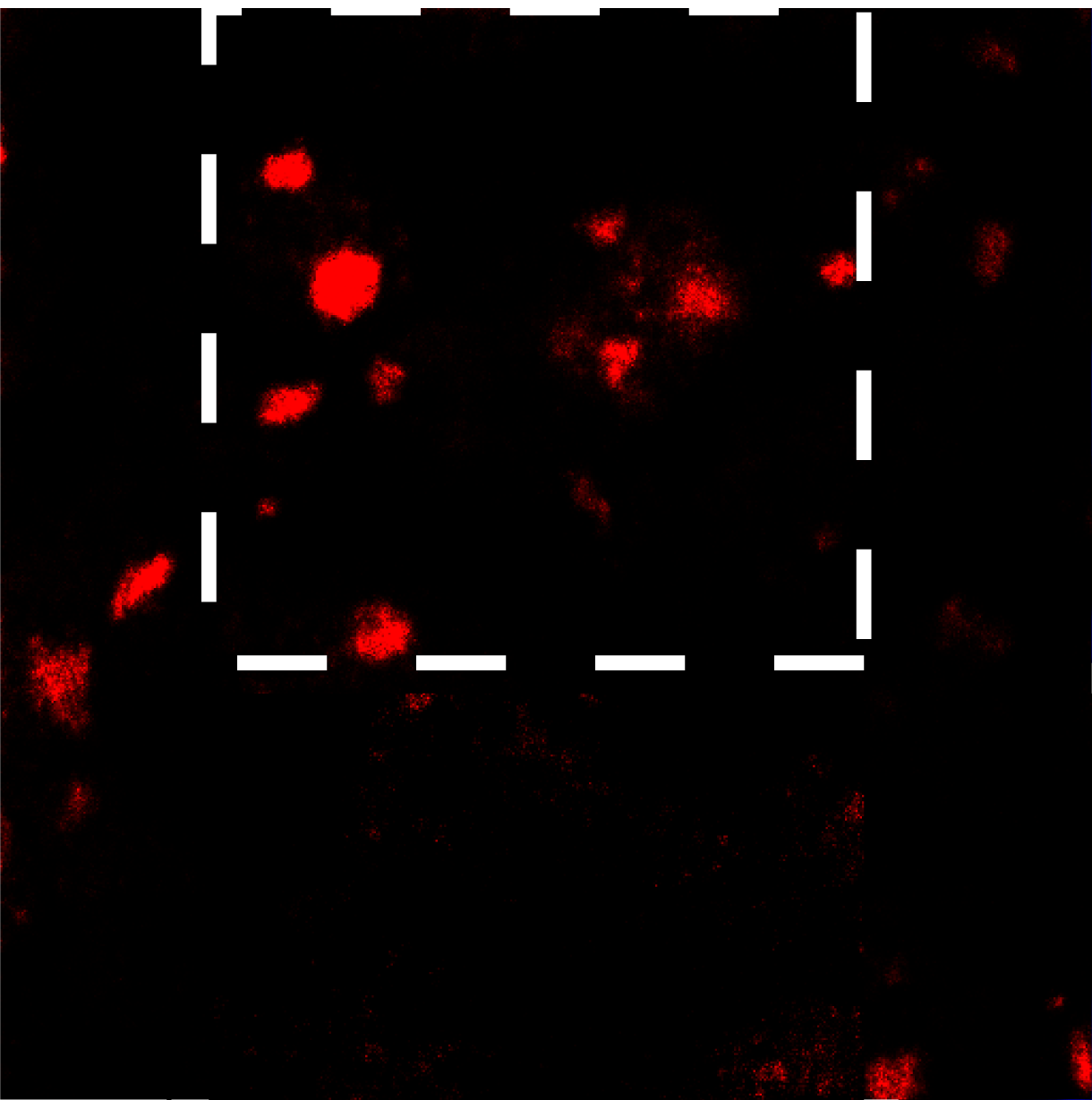

Supplement: Supplementary file 19 — Source data Fig. 1 [file 44318_2024_212_MOESM19_ESM.zip › Source Data For Figure1/1K/WT-HP1α.tif]

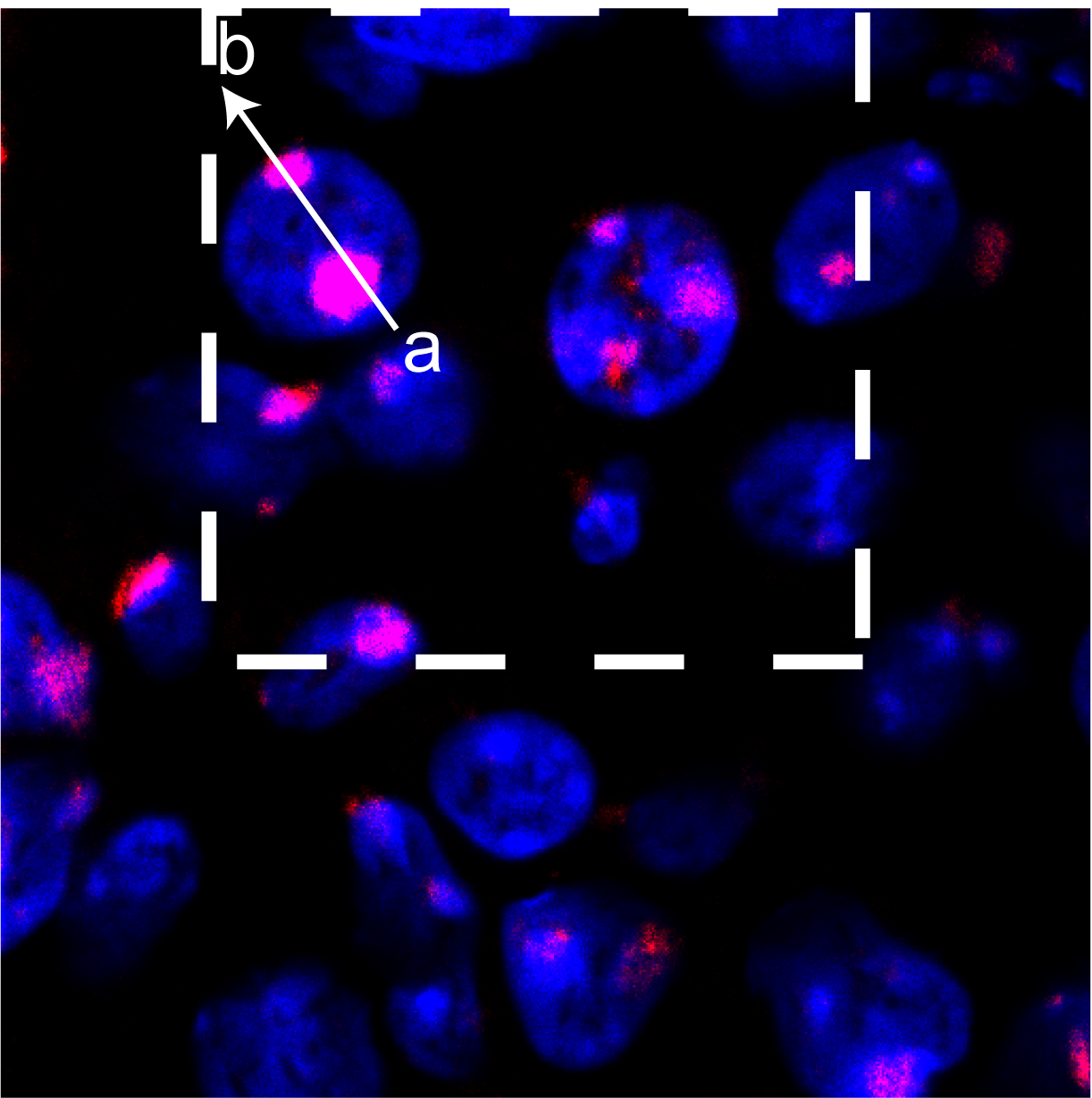

Supplement: Supplementary file 19 — Source data Fig. 1 [file 44318_2024_212_MOESM19_ESM.zip › Source Data For Figure1/1K/WT-Merge-01.tif]

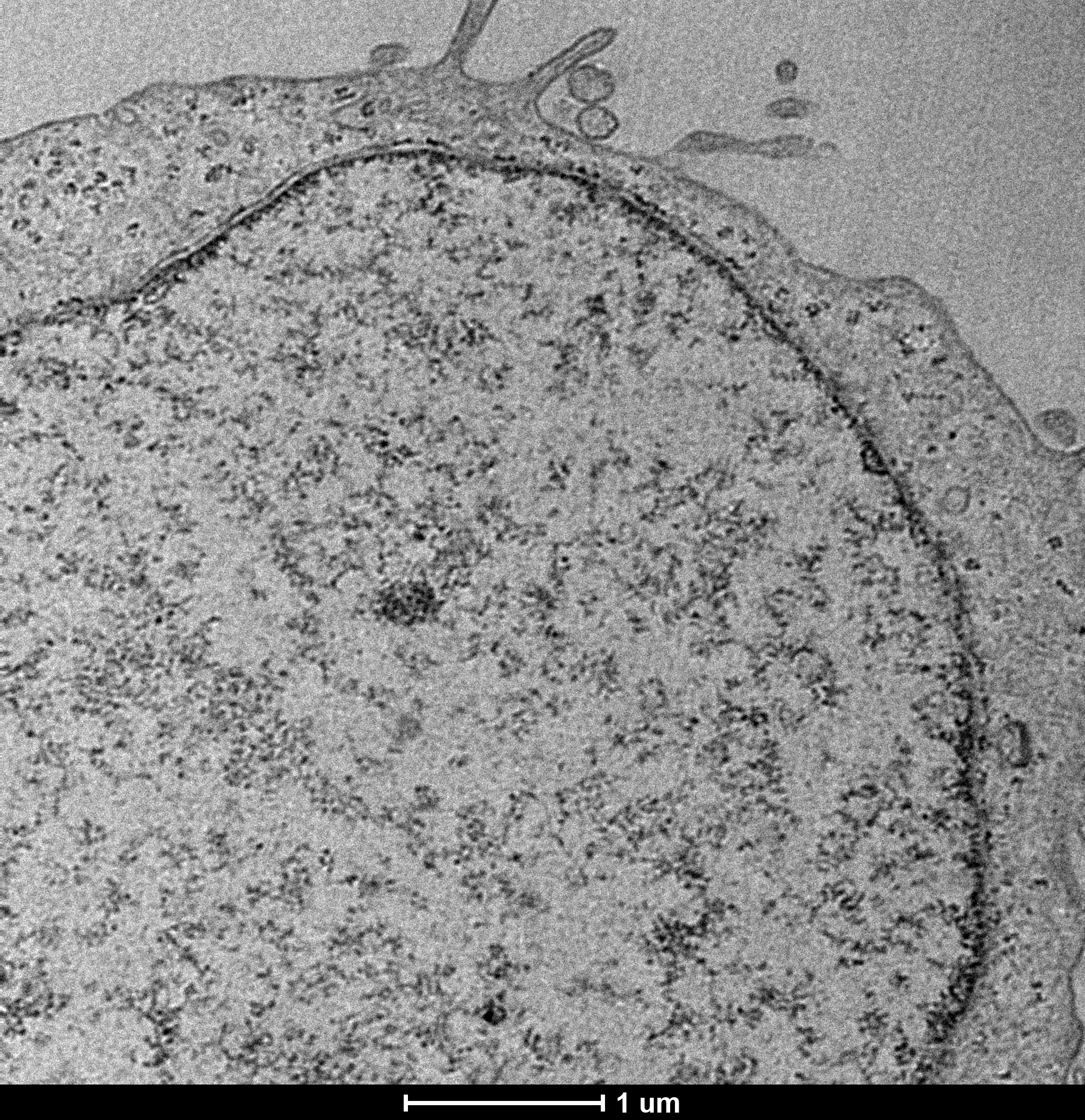

Supplement: Supplementary file 20 — Source data Fig. 2 [file 44318_2024_212_MOESM20_ESM.zip › Source Data For Figure2/2A/sgHP1α-enlarged.tif]

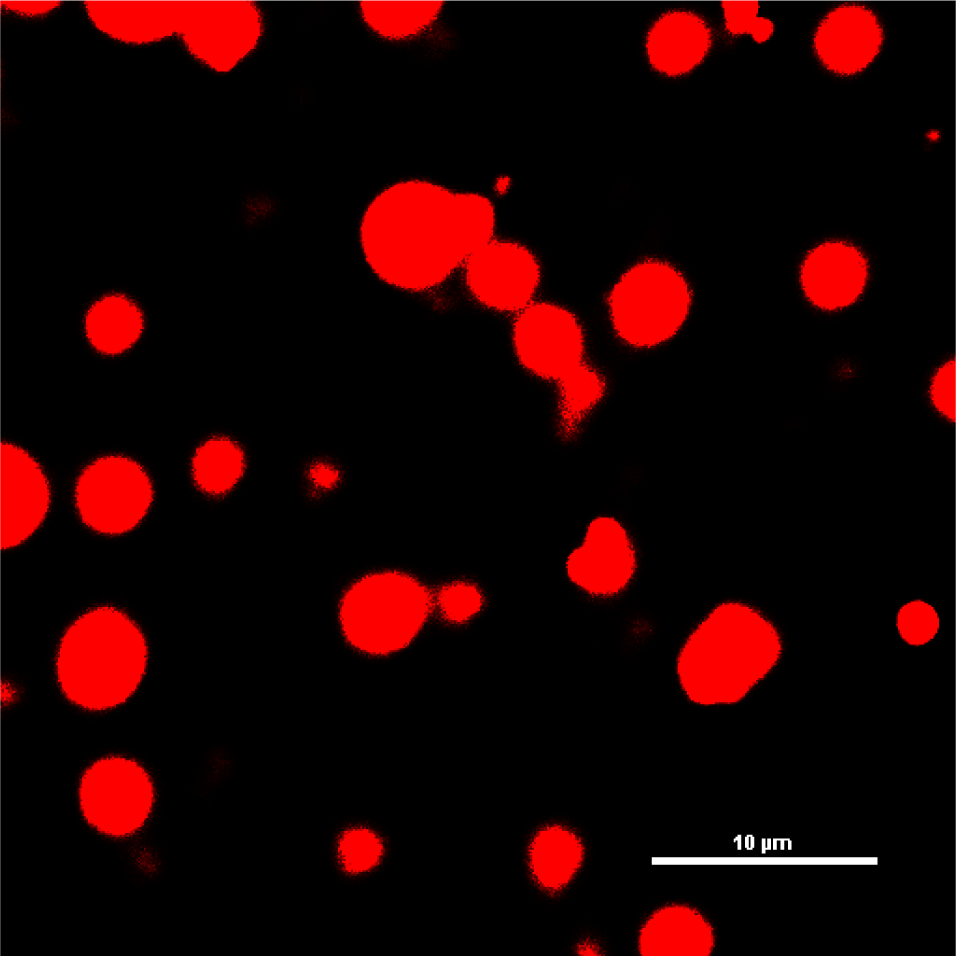

Supplement: Supplementary file 20 — Source data Fig. 2 [file 44318_2024_212_MOESM20_ESM.zip › Source Data For Figure2/2D/mCherry-HP1α-enlarged.tif]

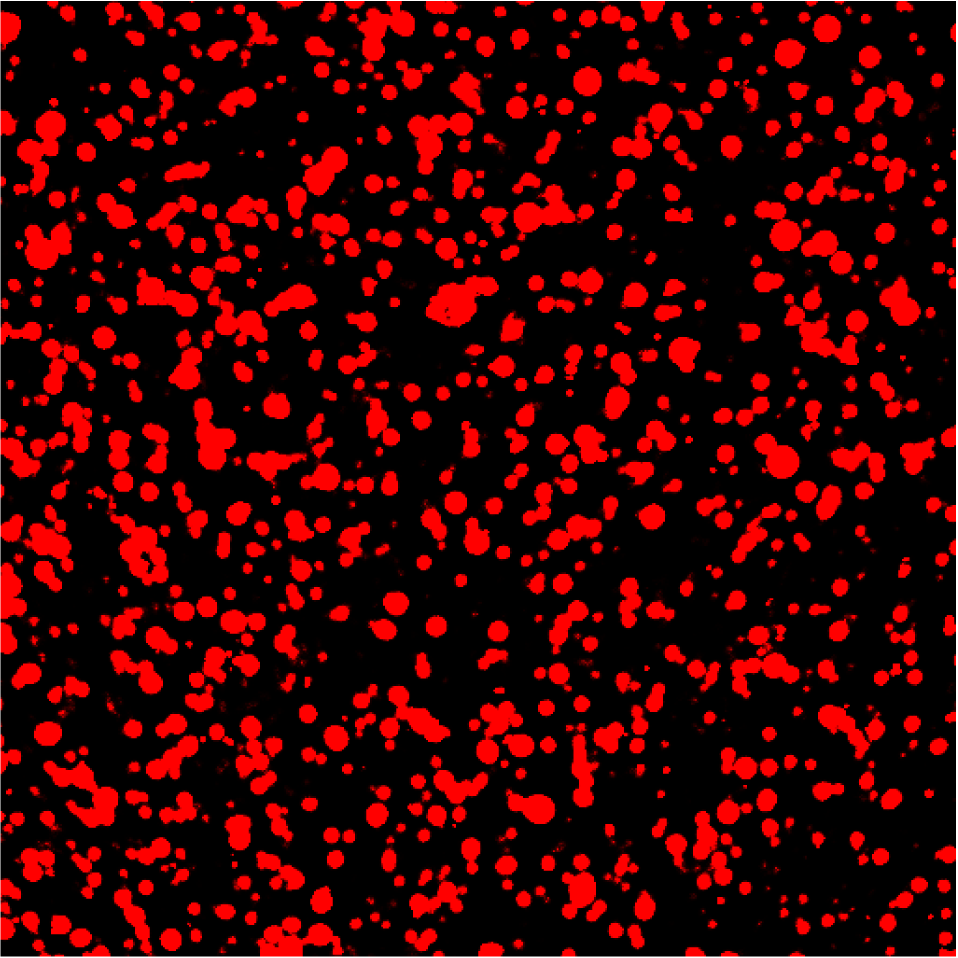

Supplement: Supplementary file 20 — Source data Fig. 2 [file 44318_2024_212_MOESM20_ESM.zip › Source Data For Figure2/2D/mCherry-HP1α.tif]

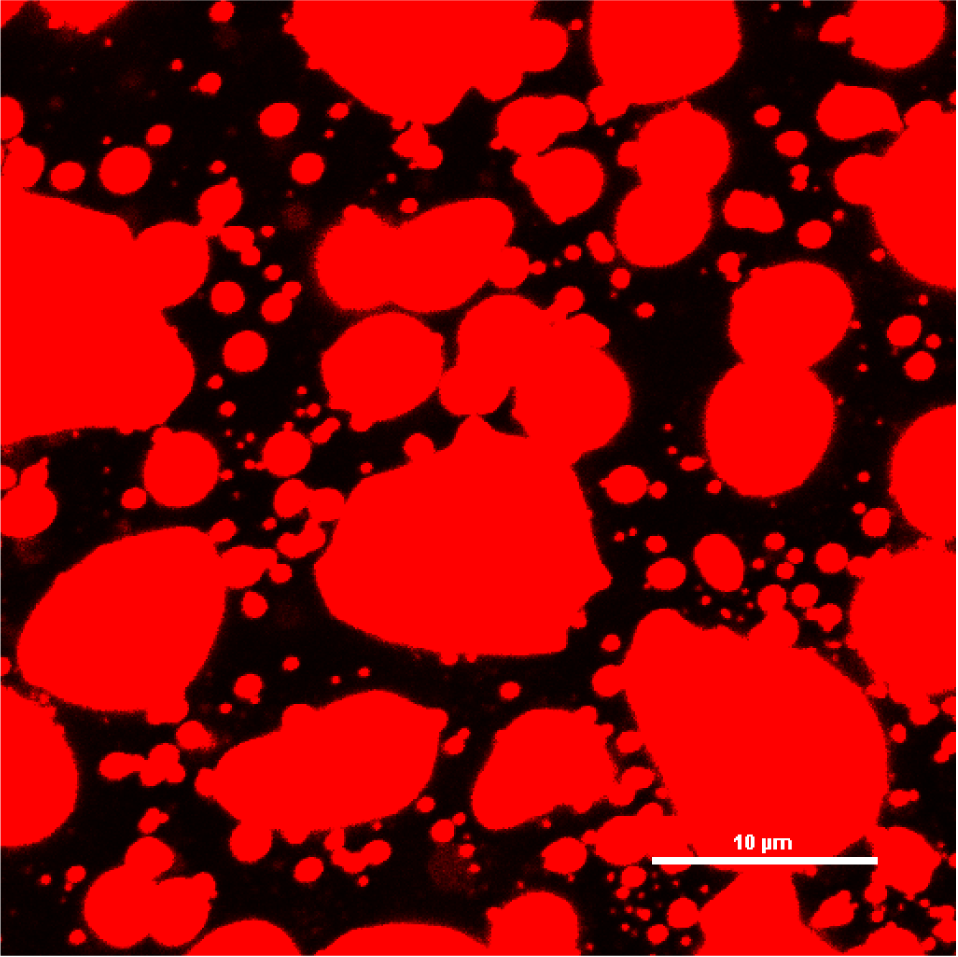

Supplement: Supplementary file 20 — Source data Fig. 2 [file 44318_2024_212_MOESM20_ESM.zip › Source Data For Figure2/2D/SUMO-mCherry-HP1α-enlarged.tif]

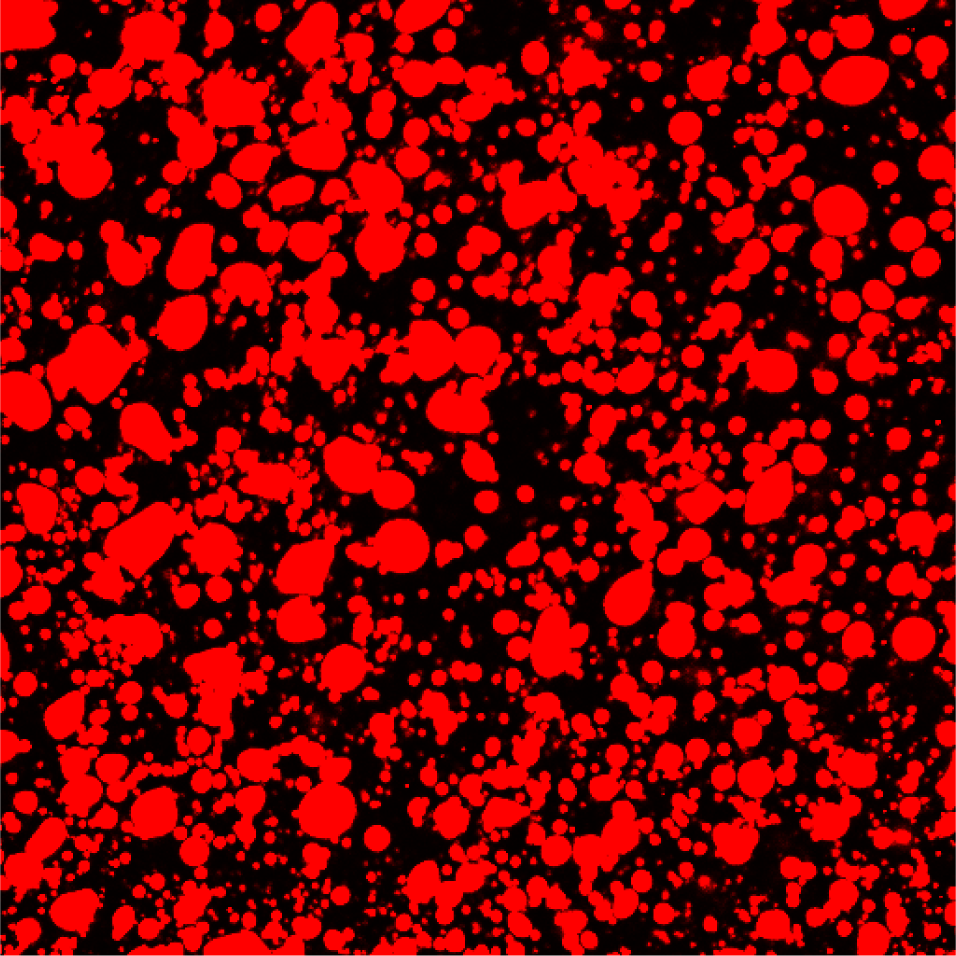

Supplement: Supplementary file 20 — Source data Fig. 2 [file 44318_2024_212_MOESM20_ESM.zip › Source Data For Figure2/2D/SUMO-mCherry-HP1α.tif]

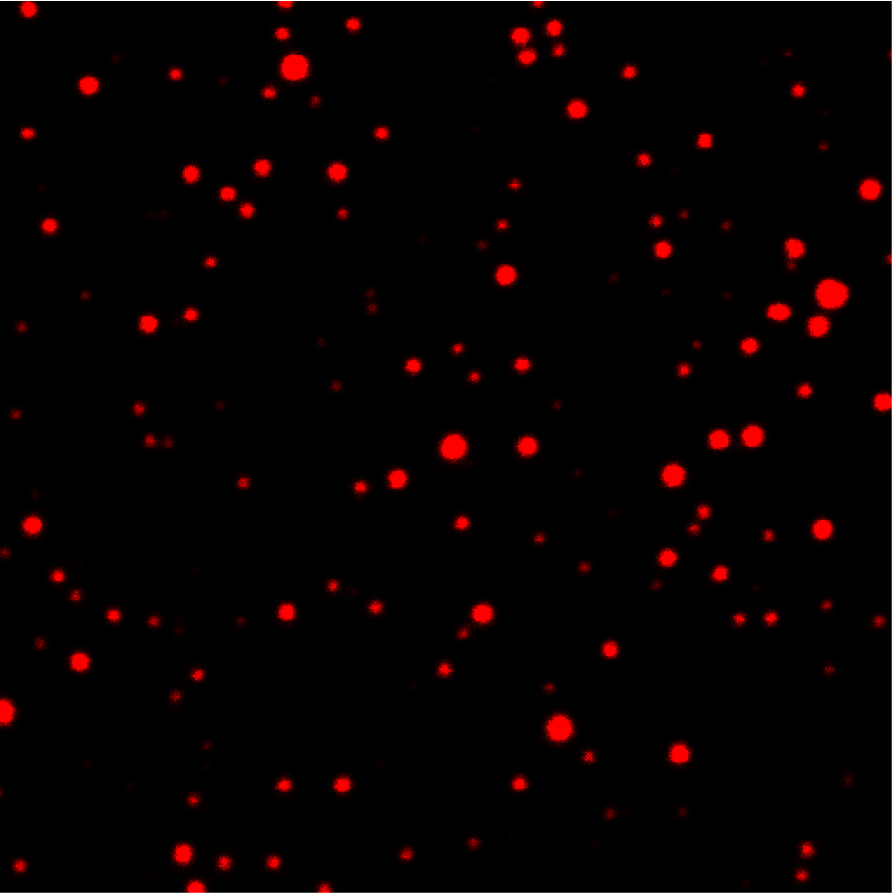

Supplement: Supplementary file 20 — Source data Fig. 2 [file 44318_2024_212_MOESM20_ESM.zip › Source Data For Figure2/2E/10 μM.tif]

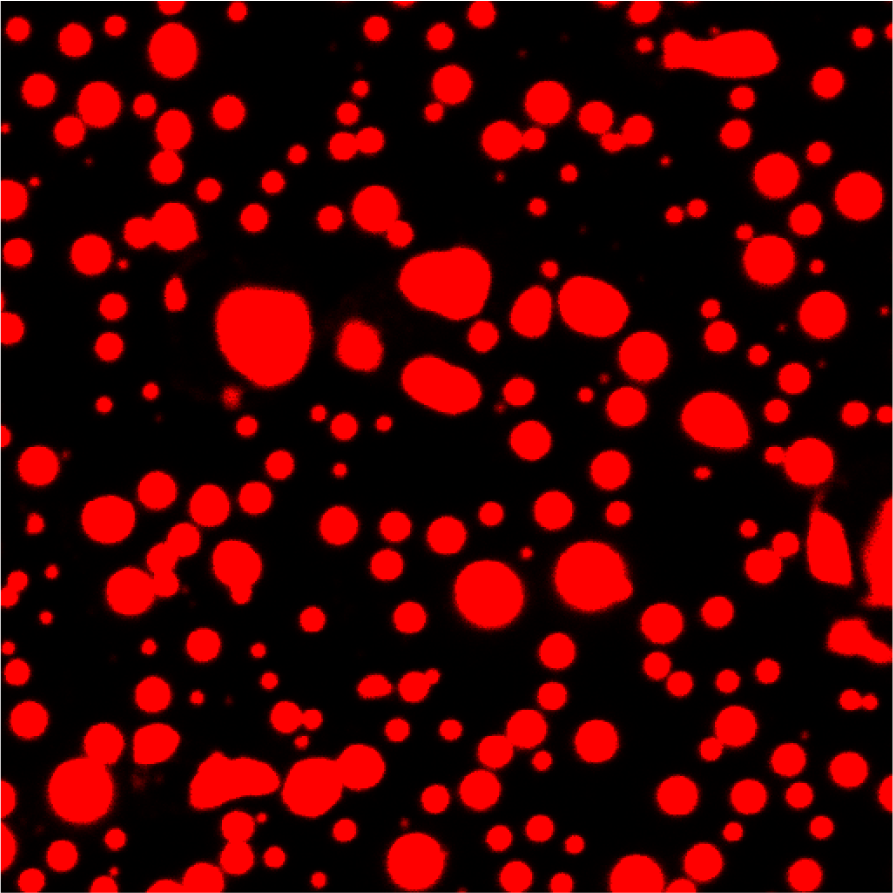

Supplement: Supplementary file 20 — Source data Fig. 2 [file 44318_2024_212_MOESM20_ESM.zip › Source Data For Figure2/2E/20 μM.tif]

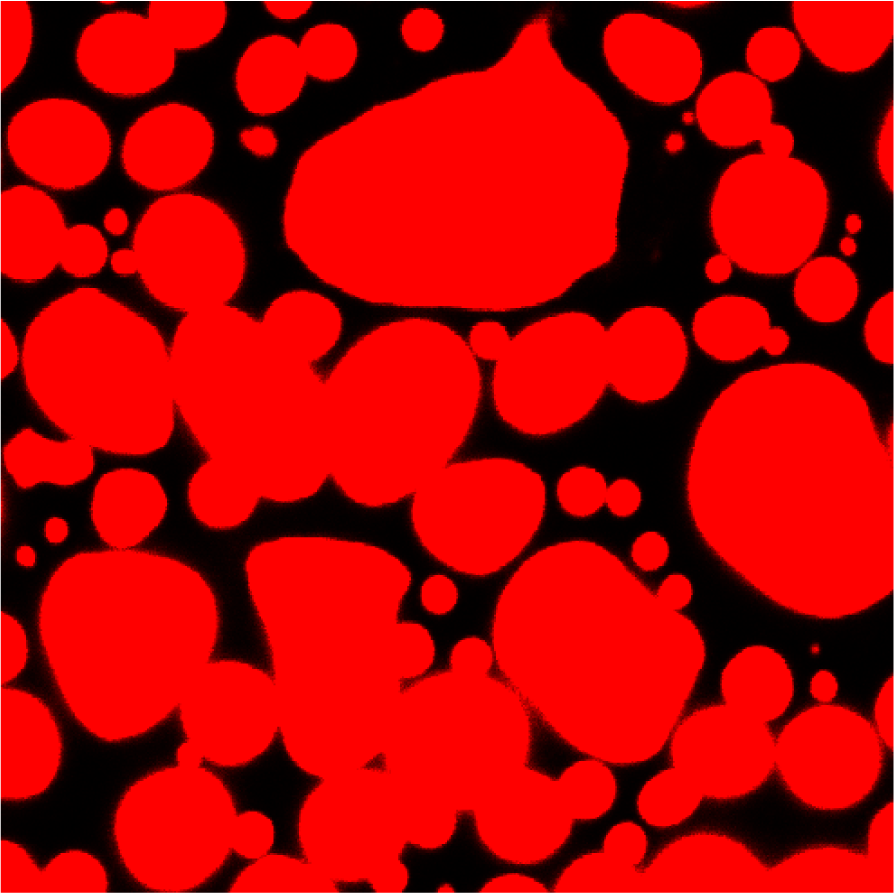

Supplement: Supplementary file 20 — Source data Fig. 2 [file 44318_2024_212_MOESM20_ESM.zip › Source Data For Figure2/2E/40 μM.tif]

**Fig. 2B**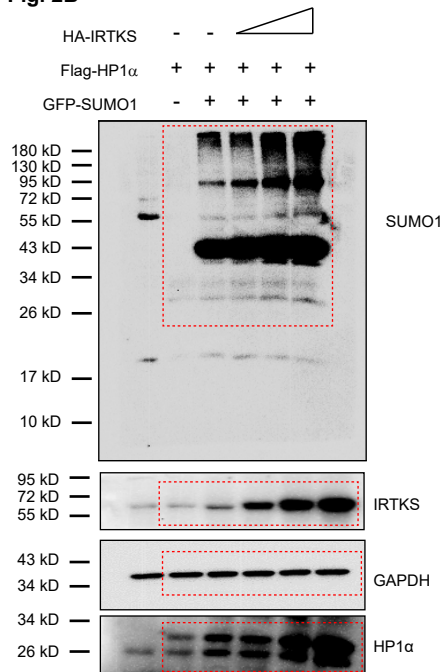**Fig. 2C**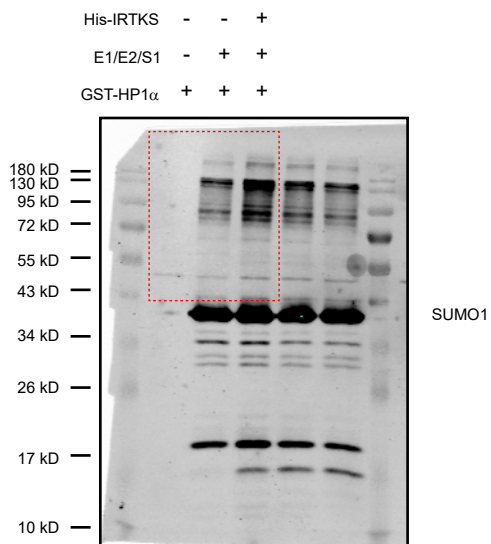

Supplement: Supplementary file 20 — Source data Fig. 2 [file 44318_2024_212_MOESM20_ESM.zip › Source Data For Figure2/Source Data Fig. 2.pdf]

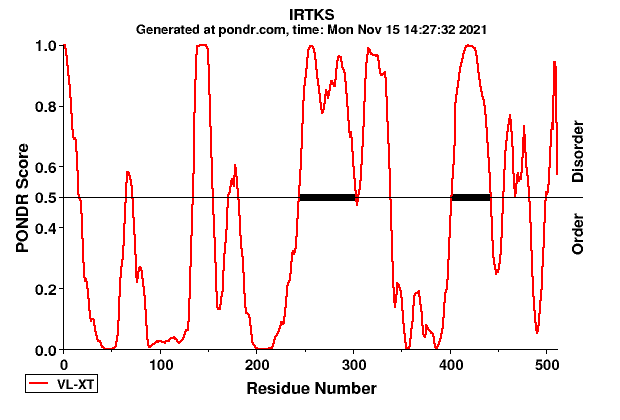

Supplement: Supplementary file 21 — Source data Fig. 3 [file 44318_2024_212_MOESM21_ESM.zip › Source Data For Figure3/3A/IRTKS prediction.gif]

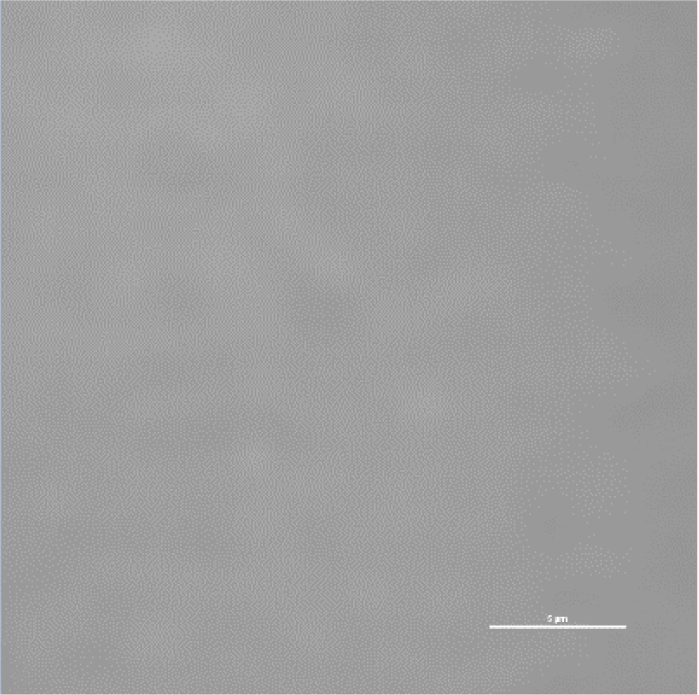

Supplement: Supplementary file 21 — Source data Fig. 3 [file 44318_2024_212_MOESM21_ESM.zip › Source Data For Figure3/3B/EGFP-BF.tif]

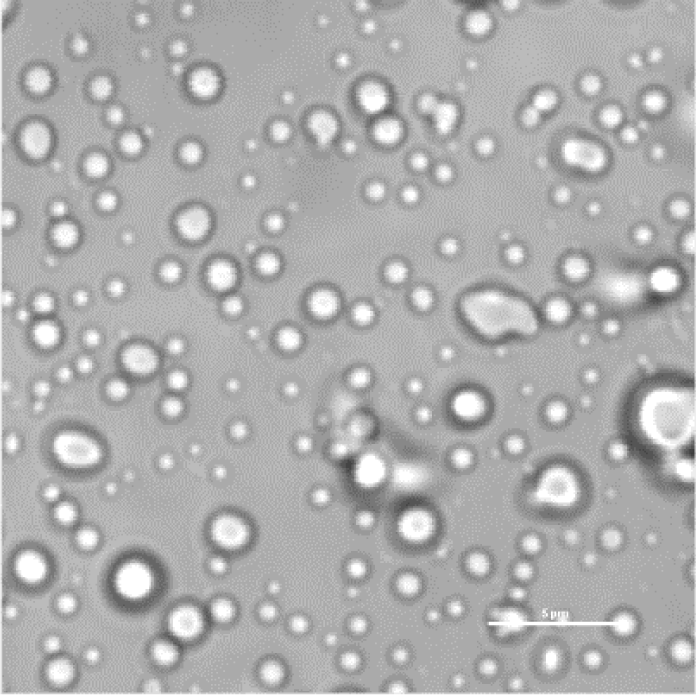

Supplement: Supplementary file 21 — Source data Fig. 3 [file 44318_2024_212_MOESM21_ESM.zip › Source Data For Figure3/3B/EGFP-IRTKS-BF.tif]

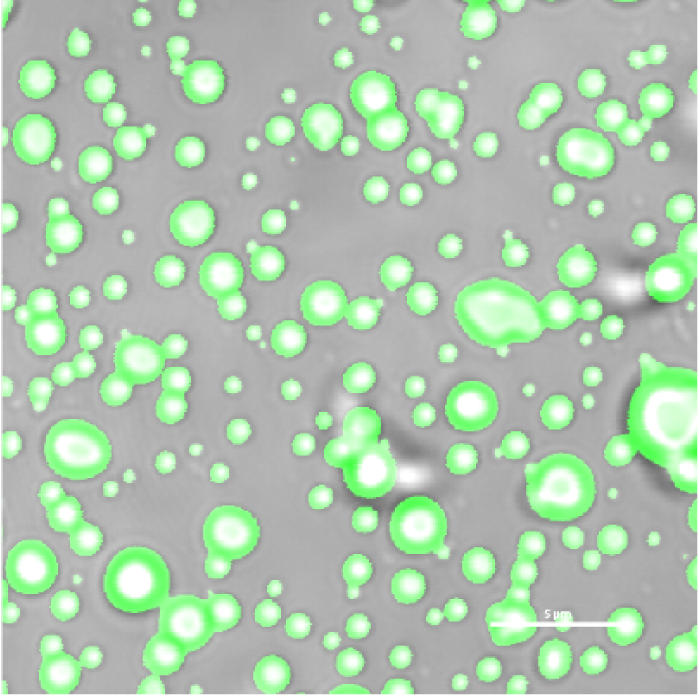

Supplement: Supplementary file 21 — Source data Fig. 3 [file 44318_2024_212_MOESM21_ESM.zip › Source Data For Figure3/3B/EGFP-IRTKS-Merge.tif]

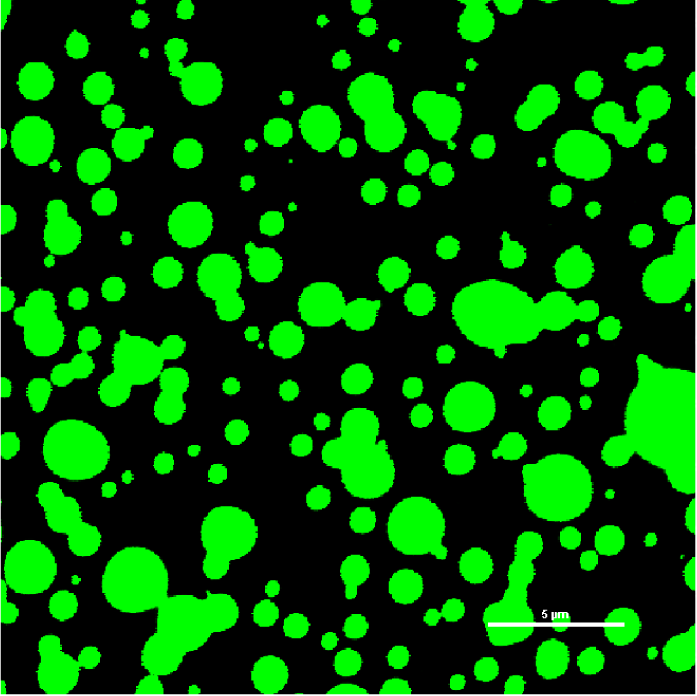

Supplement: Supplementary file 21 — Source data Fig. 3 [file 44318_2024_212_MOESM21_ESM.zip › Source Data For Figure3/3B/EGFP-IRTKS.tif]

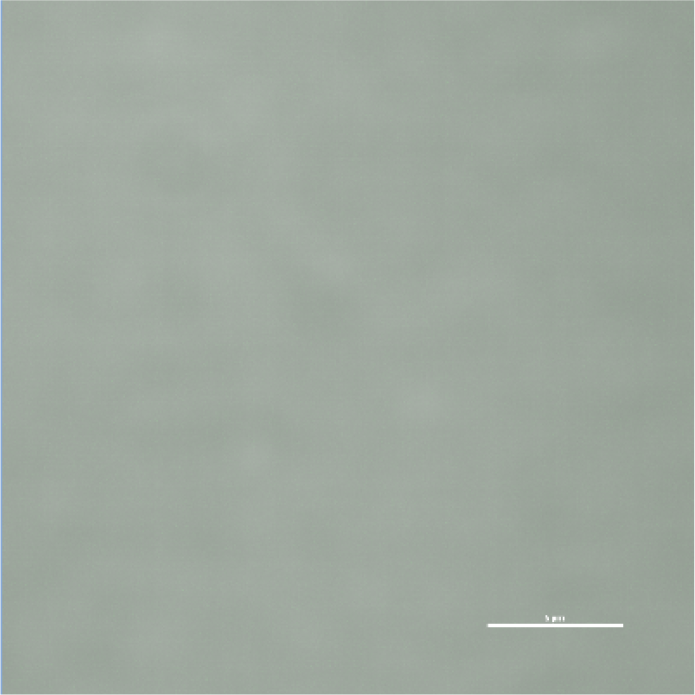

Supplement: Supplementary file 21 — Source data Fig. 3 [file 44318_2024_212_MOESM21_ESM.zip › Source Data For Figure3/3B/EGFP-Merge.tif]

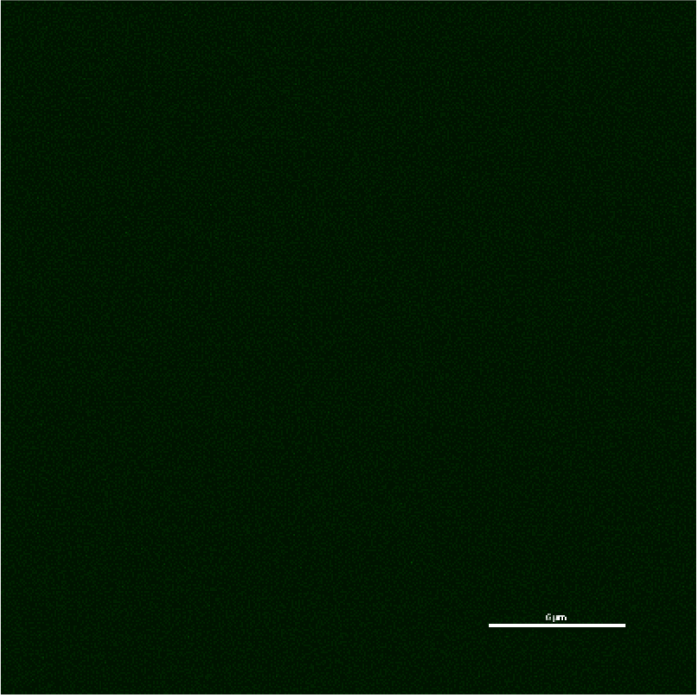

Supplement: Supplementary file 21 — Source data Fig. 3 [file 44318_2024_212_MOESM21_ESM.zip › Source Data For Figure3/3B/EGFP.tif]

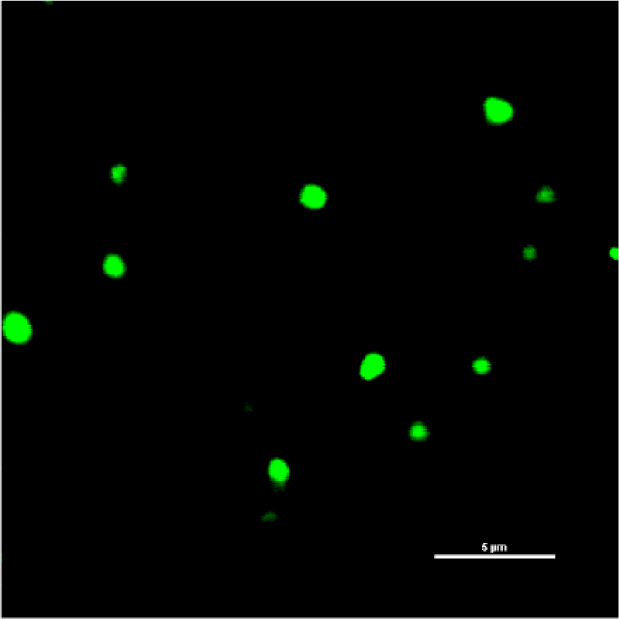

Supplement: Supplementary file 21 — Source data Fig. 3 [file 44318_2024_212_MOESM21_ESM.zip › Source Data For Figure3/3D/1,6-hex.tif]

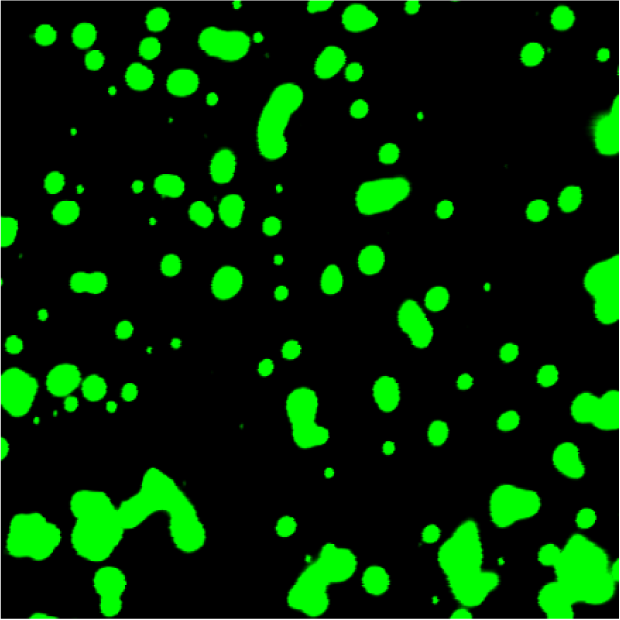

Supplement: Supplementary file 21 — Source data Fig. 3 [file 44318_2024_212_MOESM21_ESM.zip › Source Data For Figure3/3D/Control.tif]

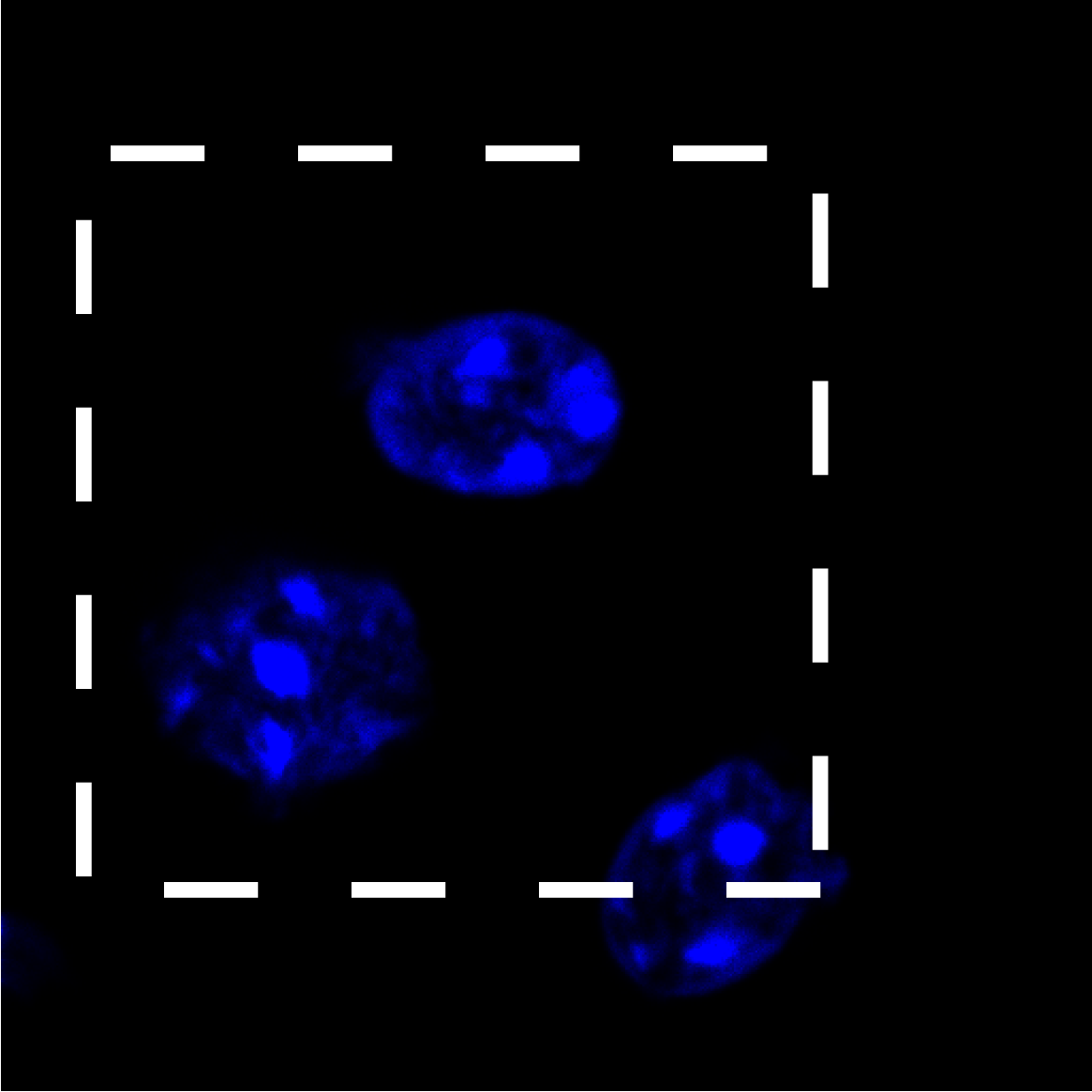

Supplement: Supplementary file 21 — Source data Fig. 3 [file 44318_2024_212_MOESM21_ESM.zip › Source Data For Figure3/3H/KO-DAPI.tif]

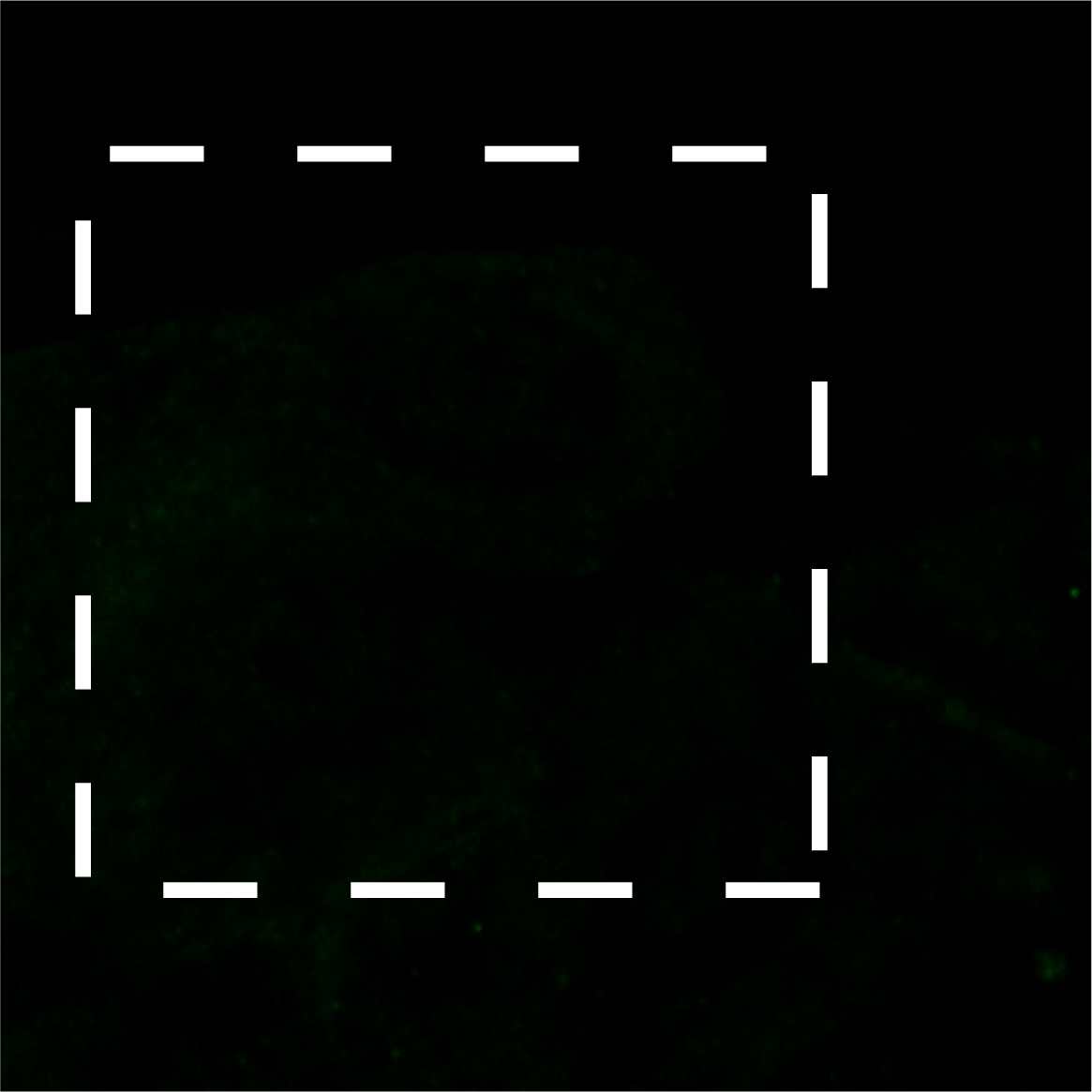

Supplement: Supplementary file 21 — Source data Fig. 3 [file 44318_2024_212_MOESM21_ESM.zip › Source Data For Figure3/3H/KO-IRTKS.tif]

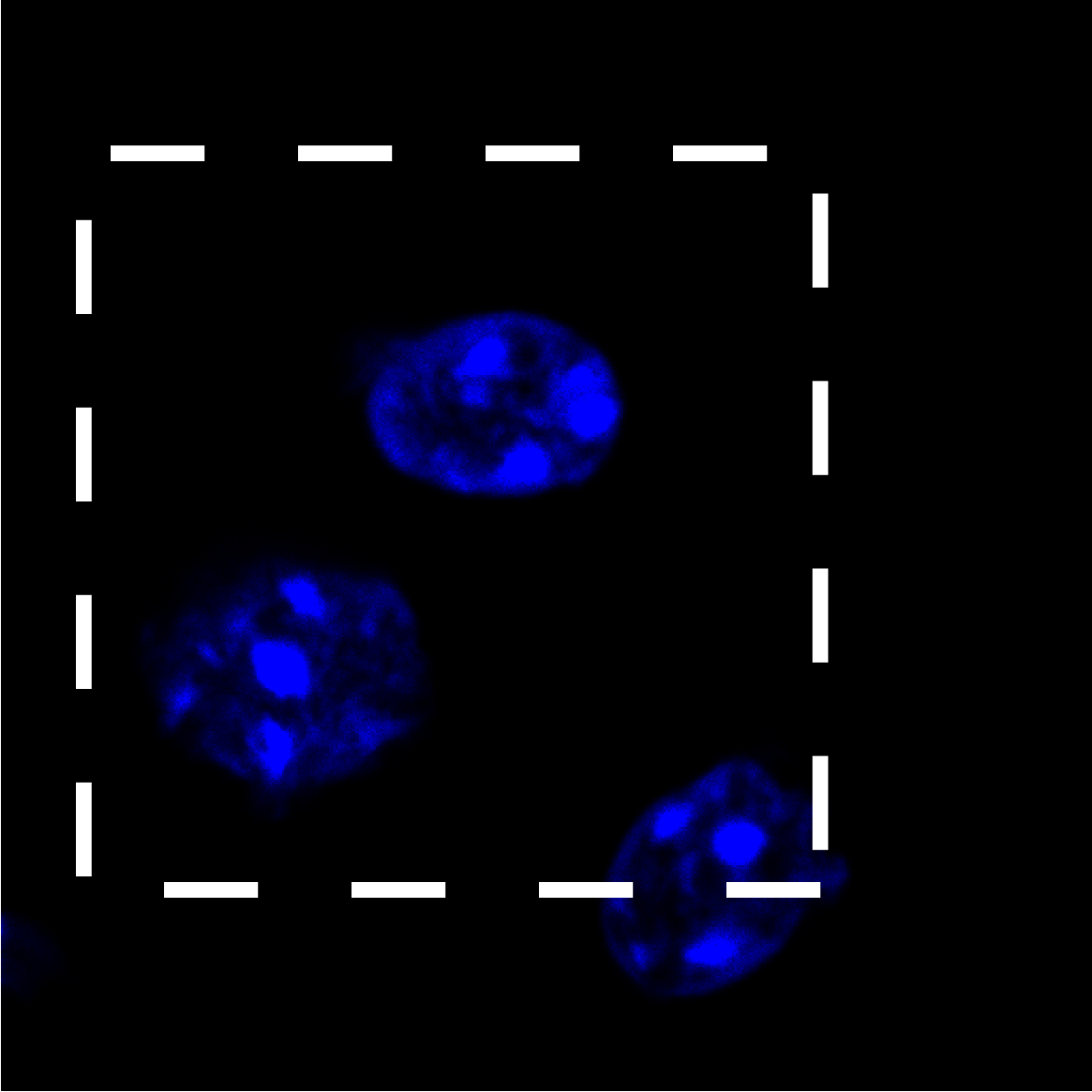

Supplement: Supplementary file 21 — Source data Fig. 3 [file 44318_2024_212_MOESM21_ESM.zip › Source Data For Figure3/3H/KO-Merge.tif]

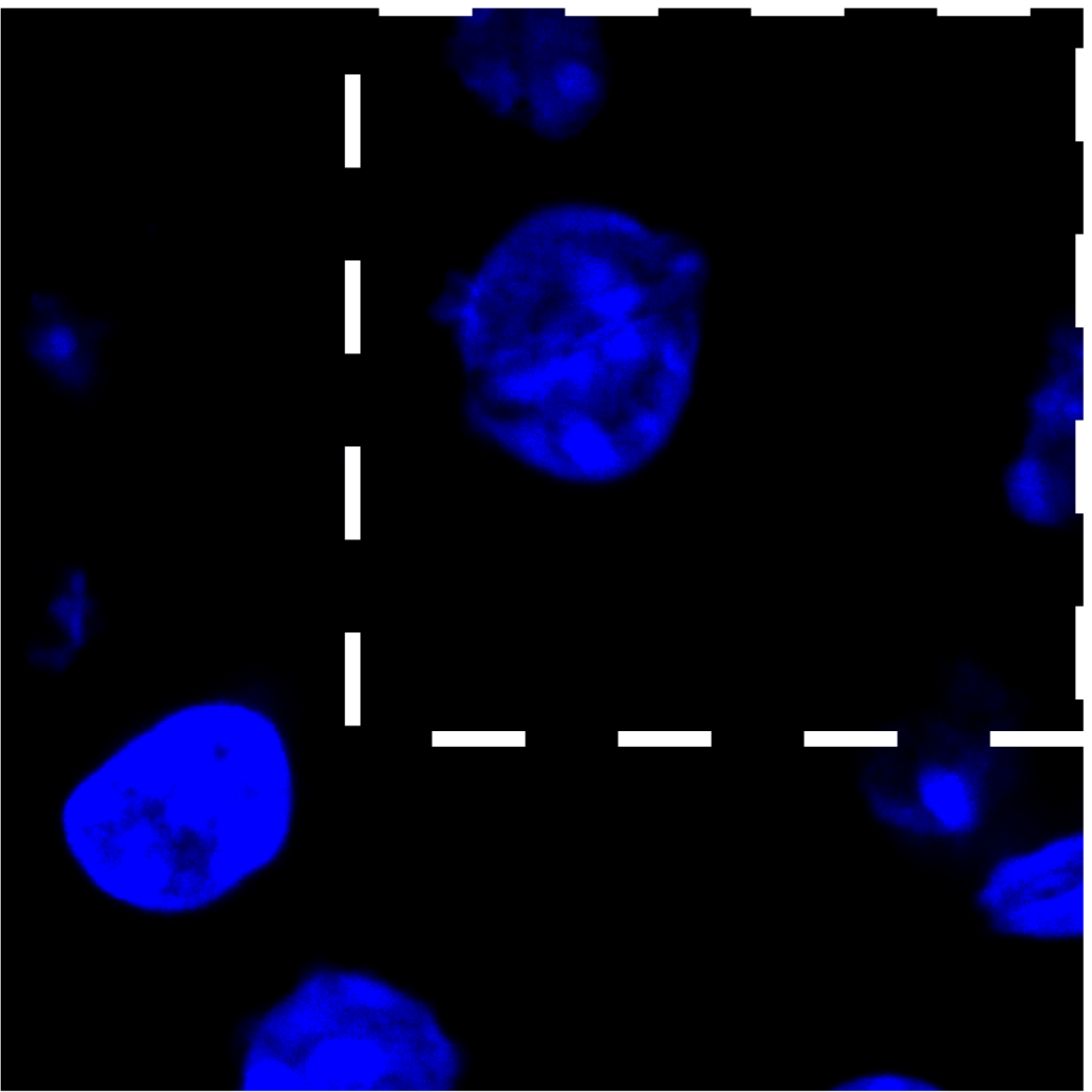

Supplement: Supplementary file 21 — Source data Fig. 3 [file 44318_2024_212_MOESM21_ESM.zip › Source Data For Figure3/3H/WT-DAPI.tif]

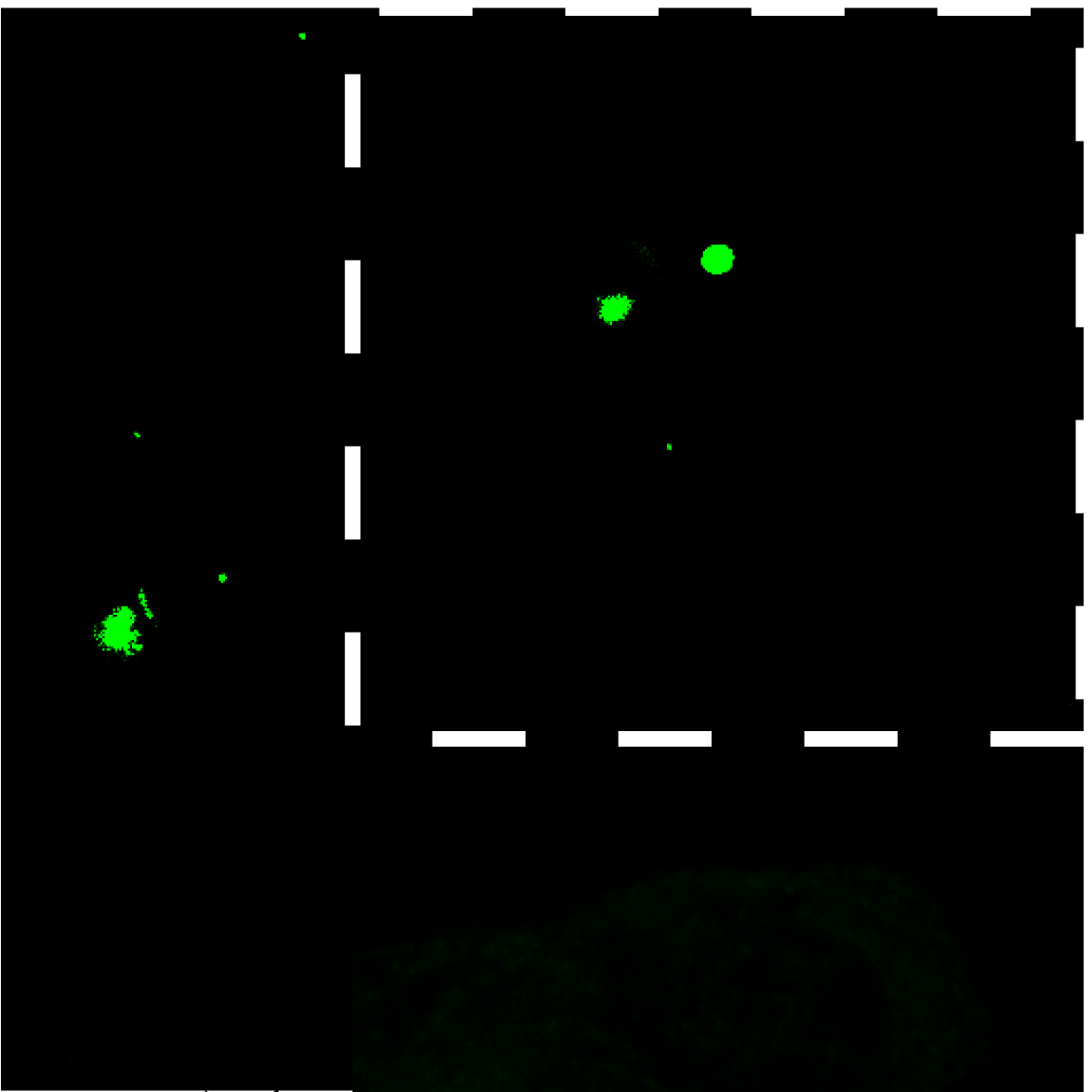

Supplement: Supplementary file 21 — Source data Fig. 3 [file 44318_2024_212_MOESM21_ESM.zip › Source Data For Figure3/3H/WT-IRTKS.tif]

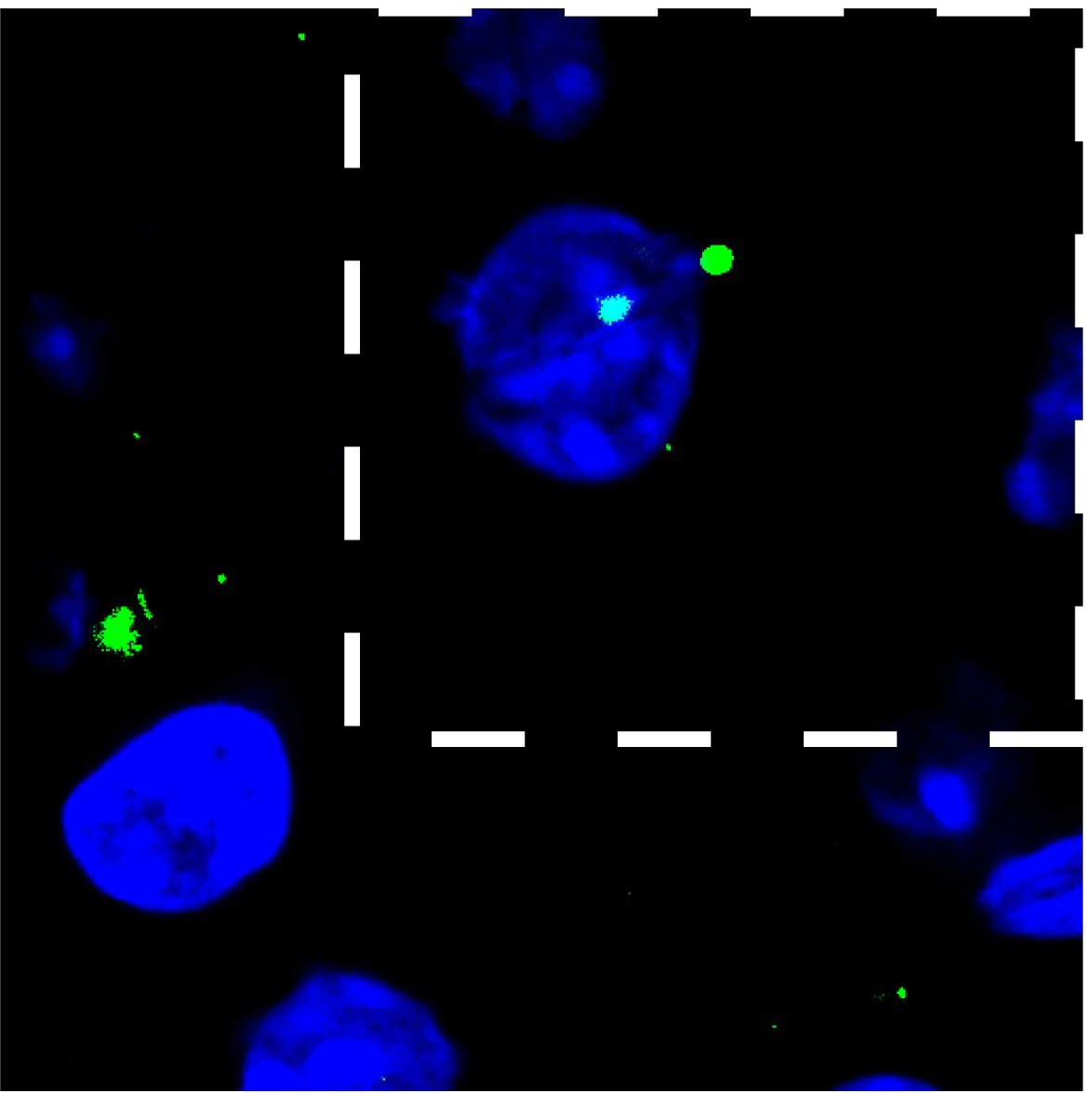

Supplement: Supplementary file 21 — Source data Fig. 3 [file 44318_2024_212_MOESM21_ESM.zip › Source Data For Figure3/3H/WT-Merge.tif]

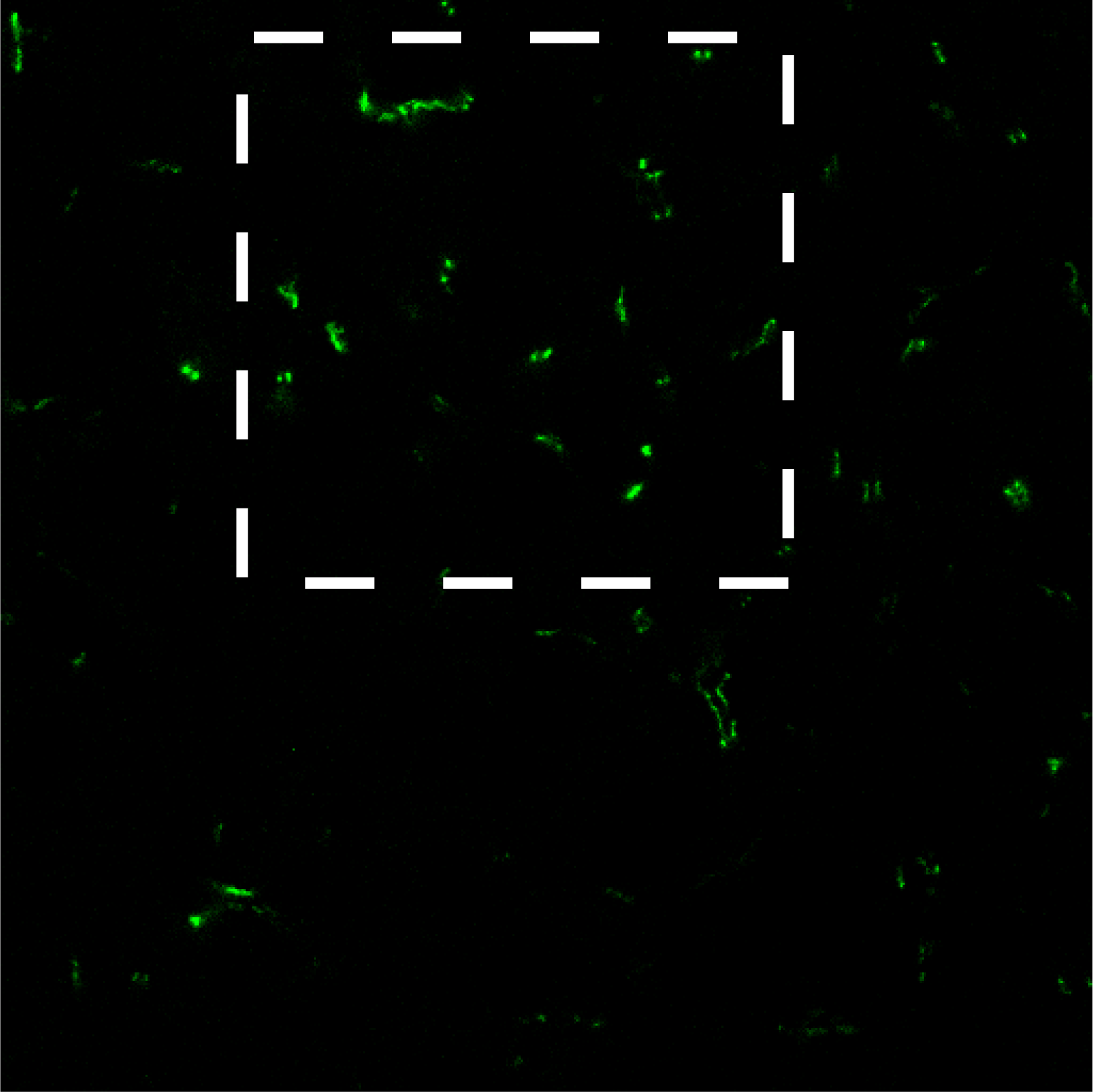

Supplement: Supplementary file 21 — Source data Fig. 3 [file 44318_2024_212_MOESM21_ESM.zip › Source Data For Figure3/3I/E.V.-DAPI.tif]

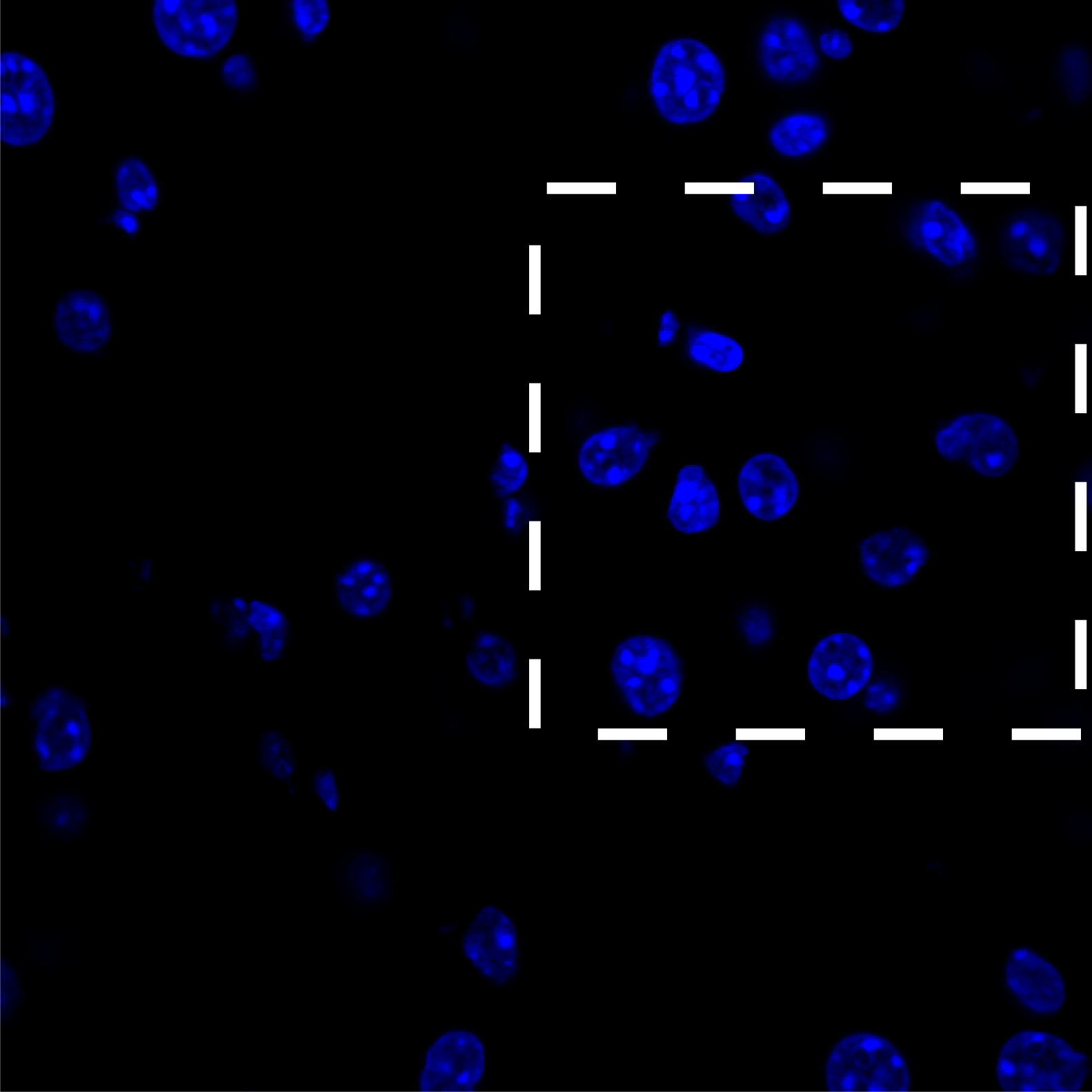

Supplement: Supplementary file 21 — Source data Fig. 3 [file 44318_2024_212_MOESM21_ESM.zip › Source Data For Figure3/3I/E.V.-IRTKS-DAPI.tif]

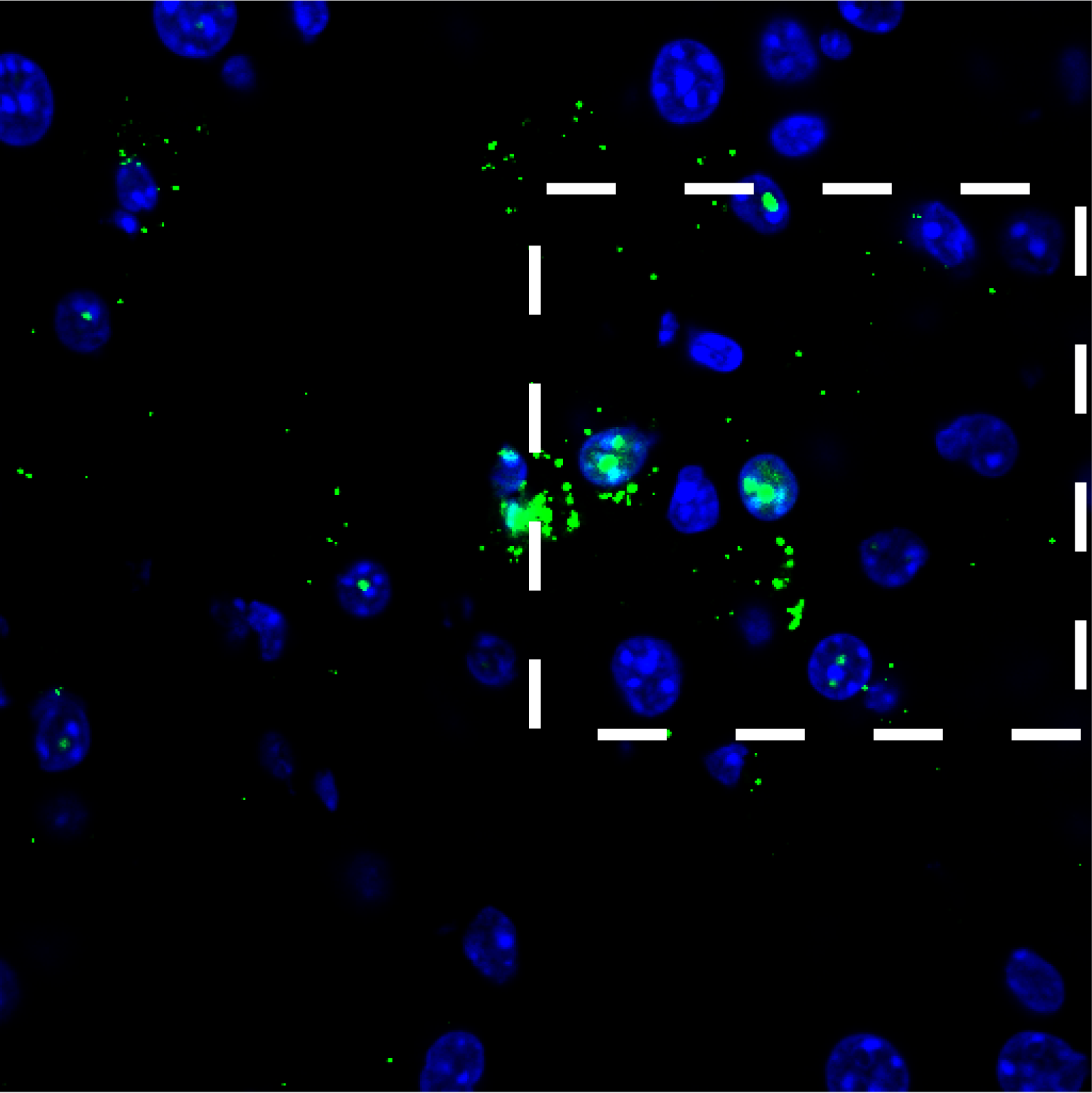

Supplement: Supplementary file 21 — Source data Fig. 3 [file 44318_2024_212_MOESM21_ESM.zip › Source Data For Figure3/3I/E.V.-IRTKS-Merge.tif]

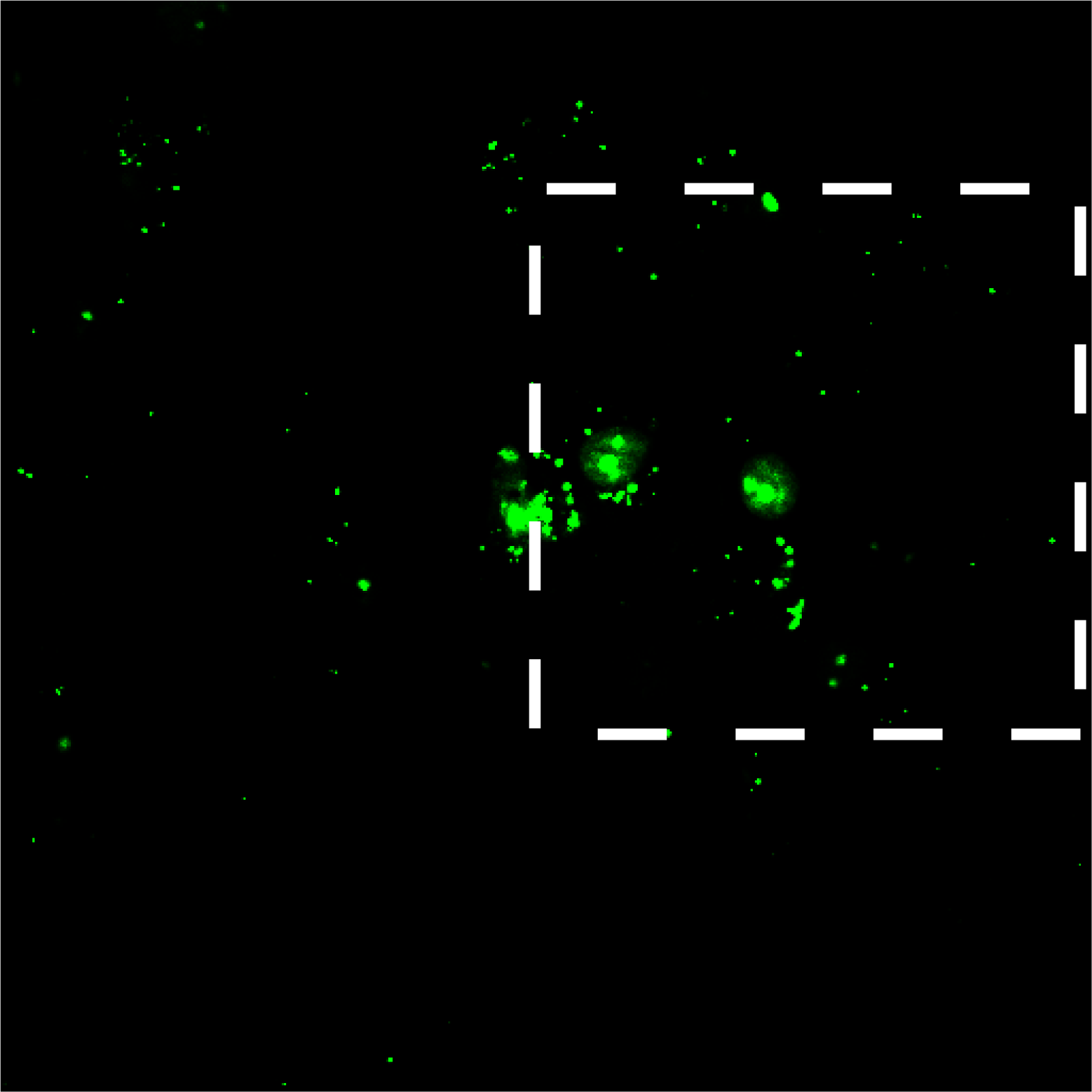

Supplement: Supplementary file 21 — Source data Fig. 3 [file 44318_2024_212_MOESM21_ESM.zip › Source Data For Figure3/3I/E.V.-IRTKS.tif]

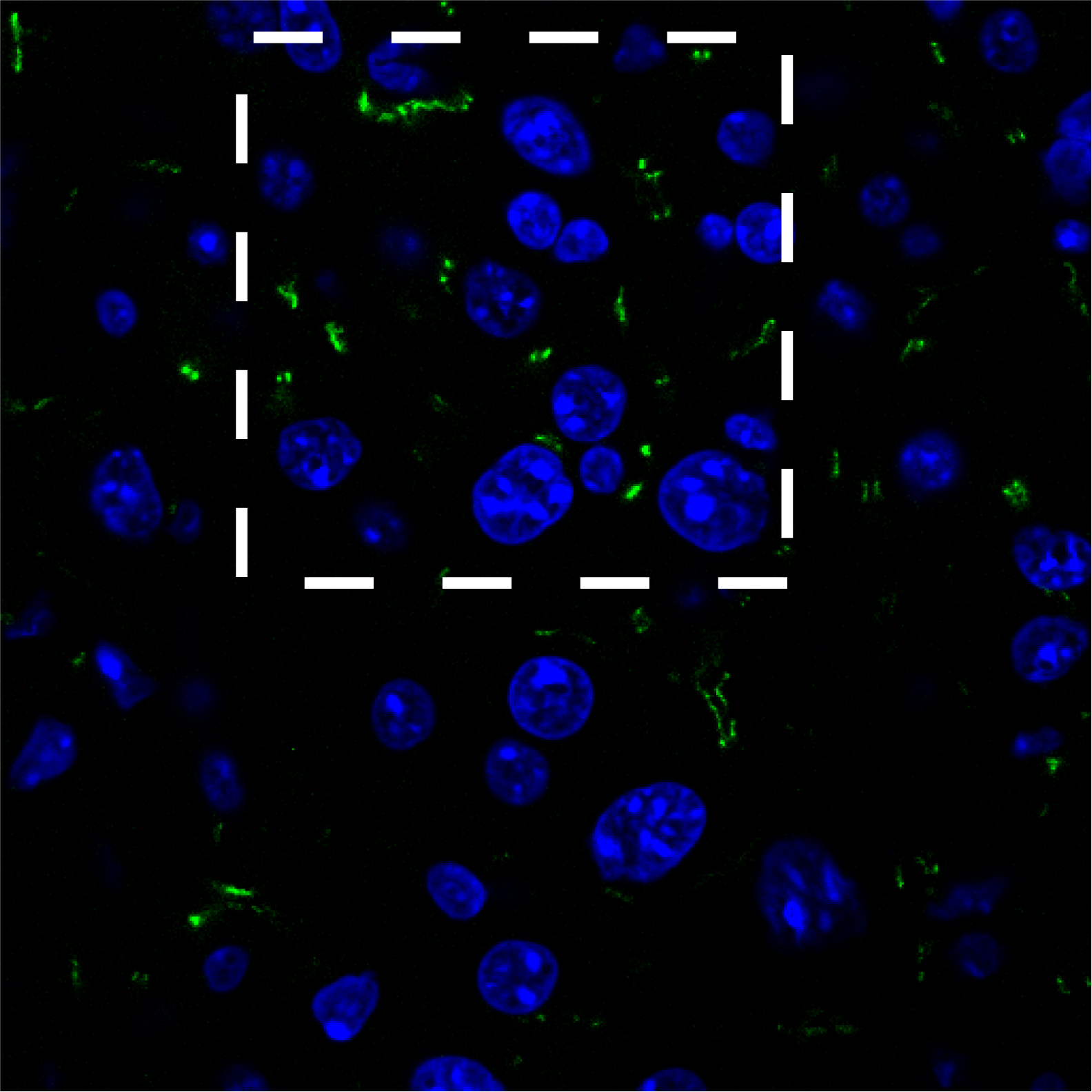

Supplement: Supplementary file 21 — Source data Fig. 3 [file 44318_2024_212_MOESM21_ESM.zip › Source Data For Figure3/3I/E.V.-Merge.tif]

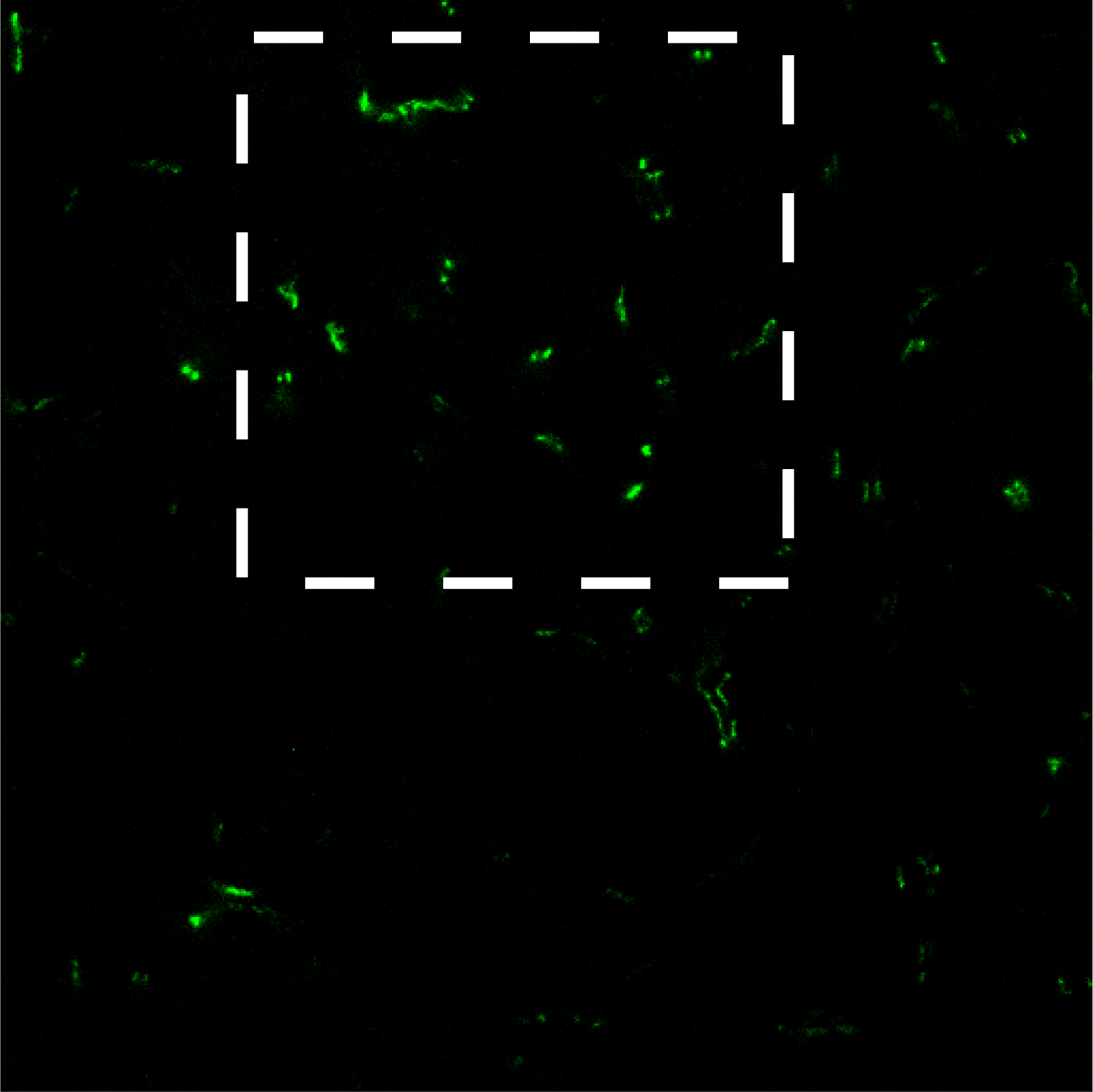

Supplement: Supplementary file 21 — Source data Fig. 3 [file 44318_2024_212_MOESM21_ESM.zip › Source Data For Figure3/3I/E.V.-ZsGreen.tif]

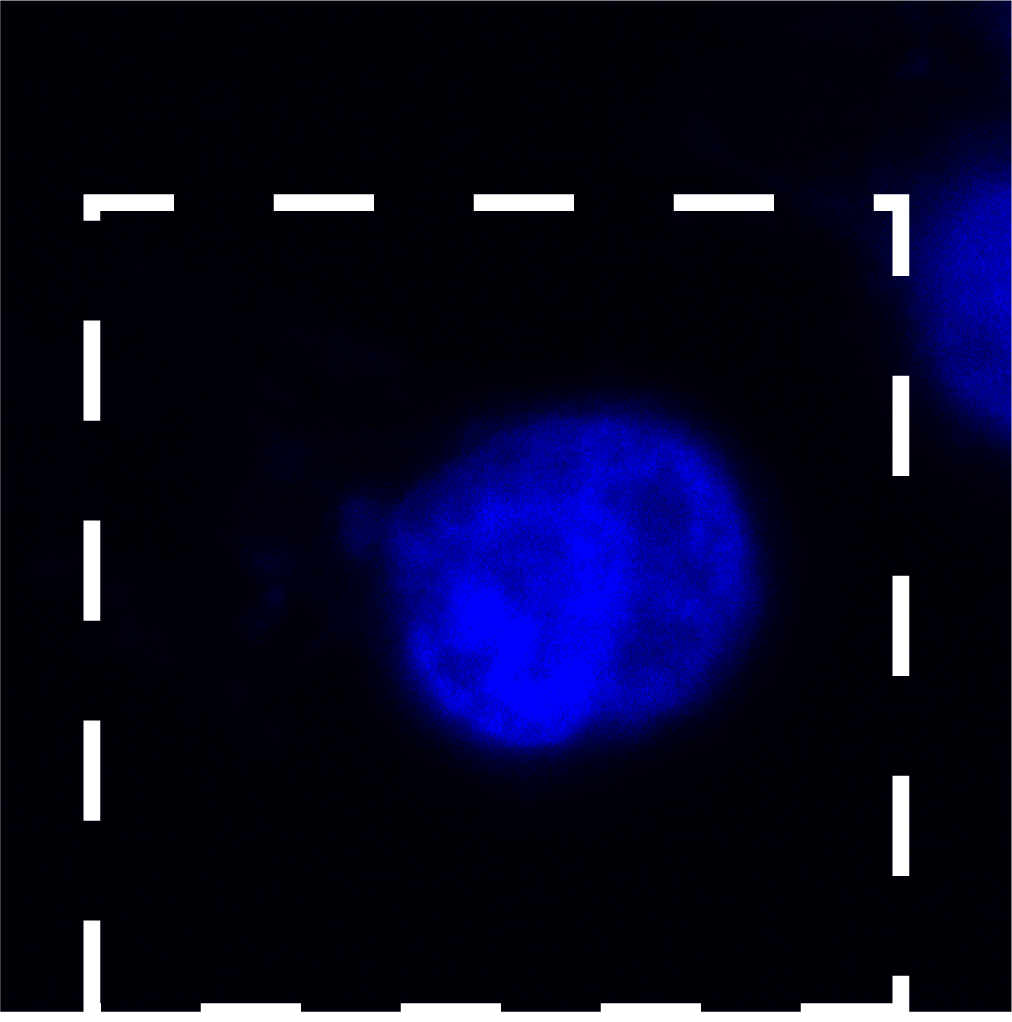

Supplement: Supplementary file 21 — Source data Fig. 3 [file 44318_2024_212_MOESM21_ESM.zip › Source Data For Figure3/3J/DAPI.tif]

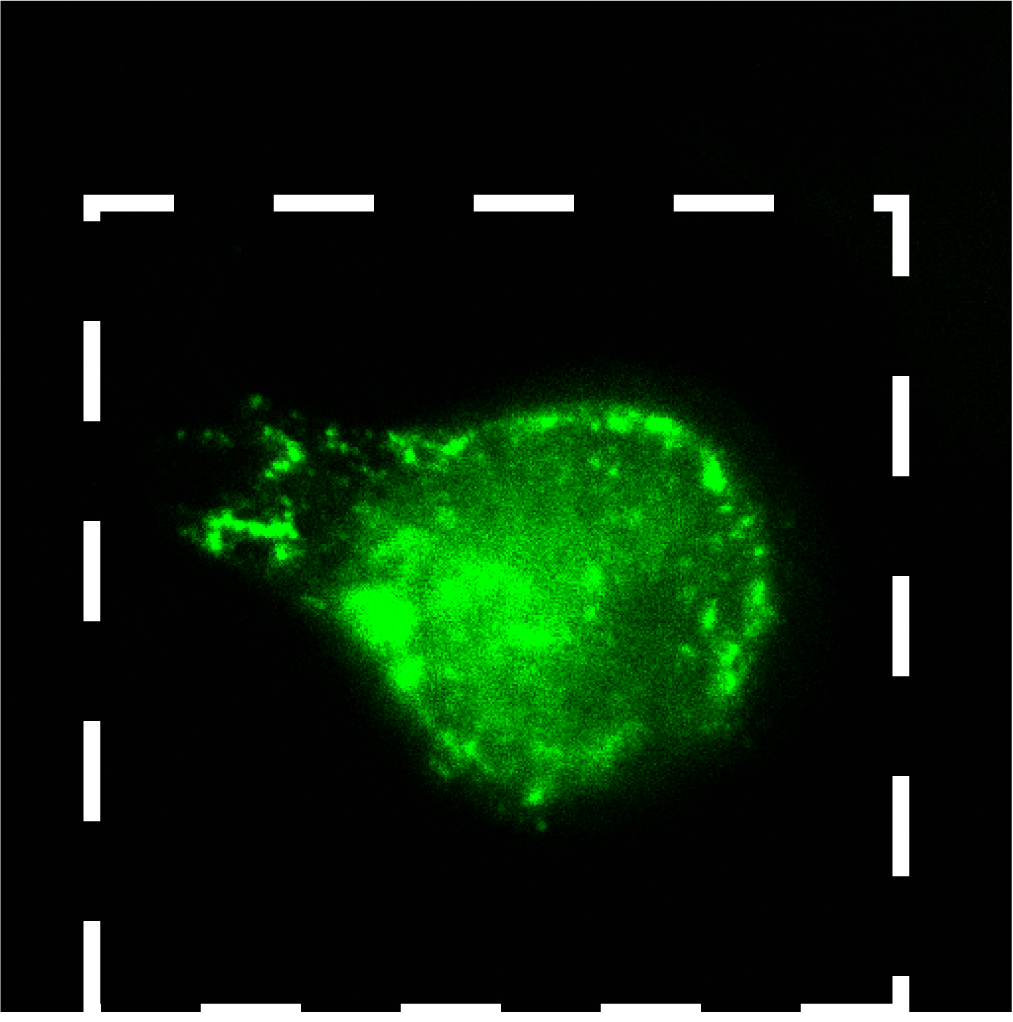

Supplement: Supplementary file 21 — Source data Fig. 3 [file 44318_2024_212_MOESM21_ESM.zip › Source Data For Figure3/3J/IRTKS.tif]

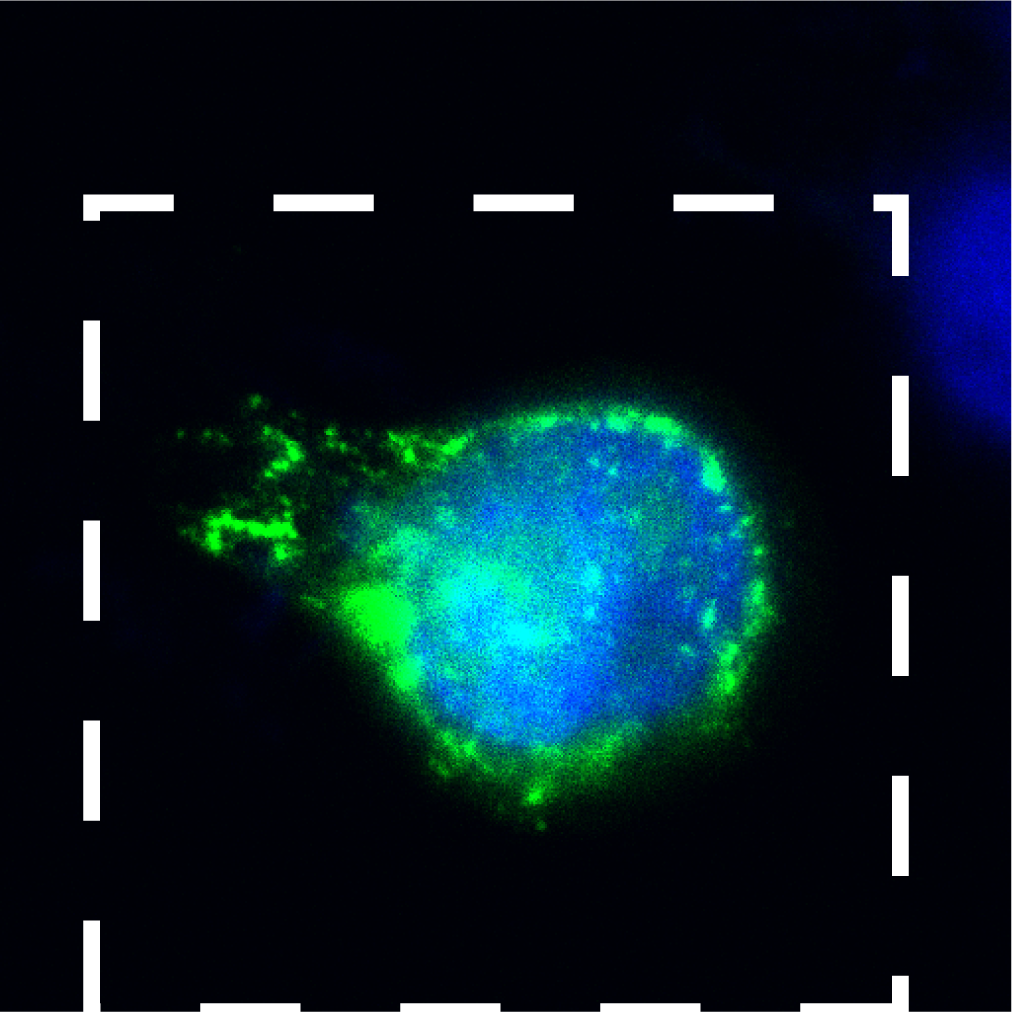

Supplement: Supplementary file 21 — Source data Fig. 3 [file 44318_2024_212_MOESM21_ESM.zip › Source Data For Figure3/3J/Merge.tif]

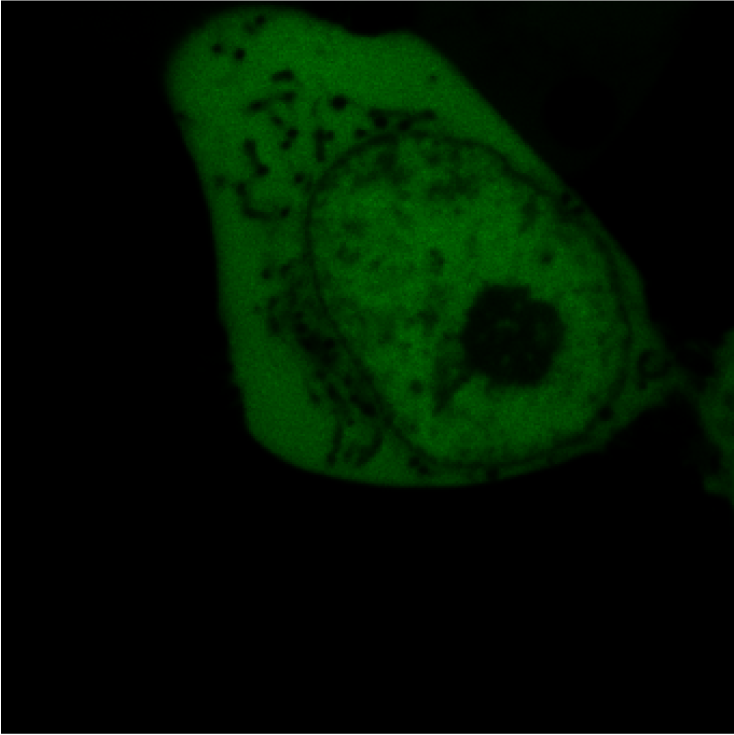

Supplement: Supplementary file 21 — Source data Fig. 3 [file 44318_2024_212_MOESM21_ESM.zip › Source Data For Figure3/3L/EGFP.tif]

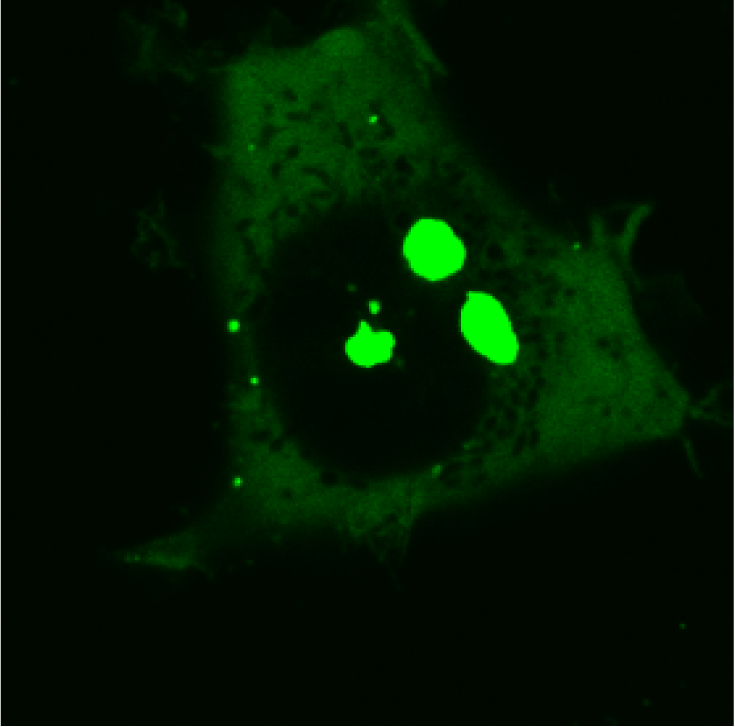

Supplement: Supplementary file 21 — Source data Fig. 3 [file 44318_2024_212_MOESM21_ESM.zip › Source Data For Figure3/3L/IRTKS.tif]

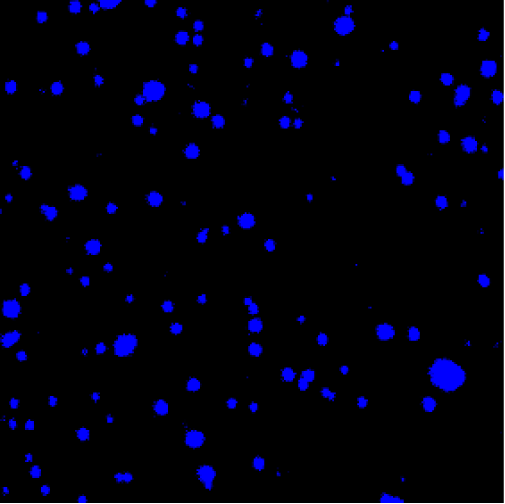

Supplement: Supplementary file 22 — Source data Fig. 4 [file 44318_2024_212_MOESM22_ESM.zip › Source Data For Figure4/4A/10 μm DAPI.tif]

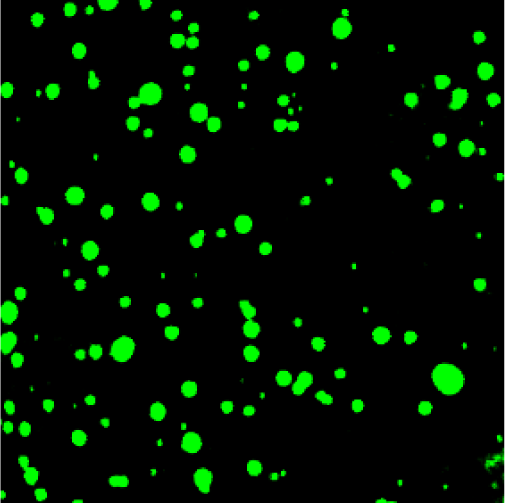

Supplement: Supplementary file 22 — Source data Fig. 4 [file 44318_2024_212_MOESM22_ESM.zip › Source Data For Figure4/4A/10 μm IRTKS.tif]

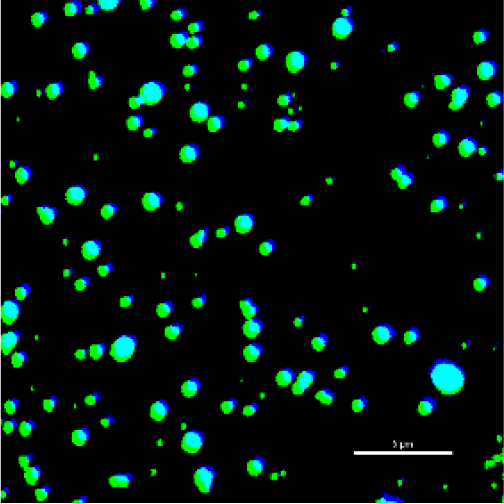

Supplement: Supplementary file 22 — Source data Fig. 4 [file 44318_2024_212_MOESM22_ESM.zip › Source Data For Figure4/4A/10 μm Merge.tif]

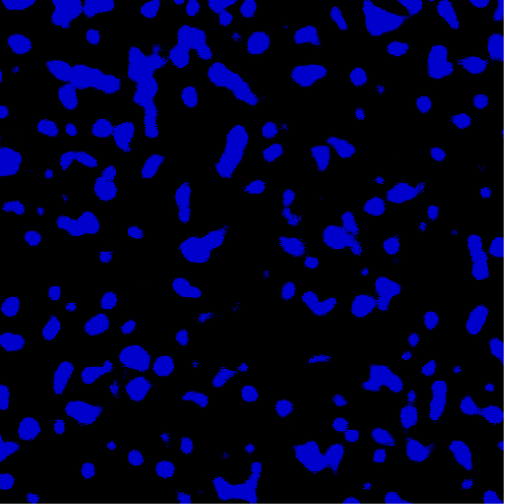

Supplement: Supplementary file 22 — Source data Fig. 4 [file 44318_2024_212_MOESM22_ESM.zip › Source Data For Figure4/4A/20 μm DAPI.tif]

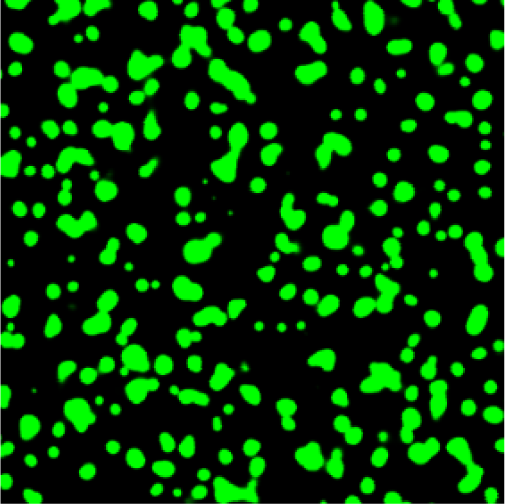

Supplement: Supplementary file 22 — Source data Fig. 4 [file 44318_2024_212_MOESM22_ESM.zip › Source Data For Figure4/4A/20 μm IRTKS.tif]

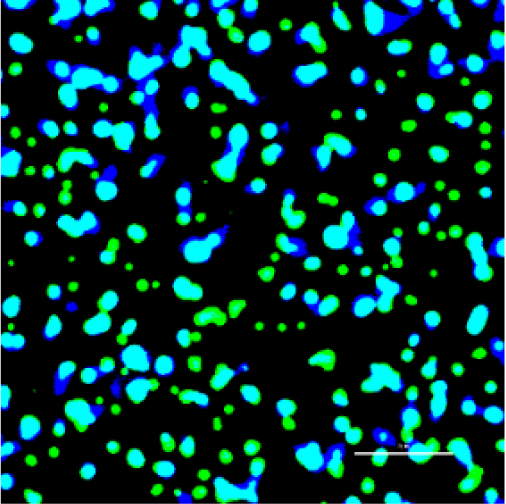

Supplement: Supplementary file 22 — Source data Fig. 4 [file 44318_2024_212_MOESM22_ESM.zip › Source Data For Figure4/4A/20 μm Merge.tif]

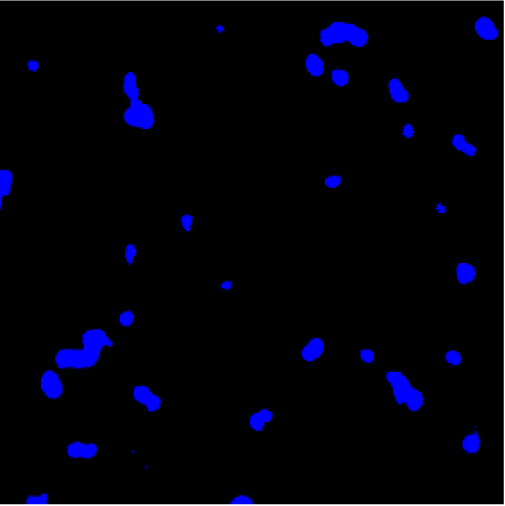

Supplement: Supplementary file 22 — Source data Fig. 4 [file 44318_2024_212_MOESM22_ESM.zip › Source Data For Figure4/4A/5 μm DAPI.tif]

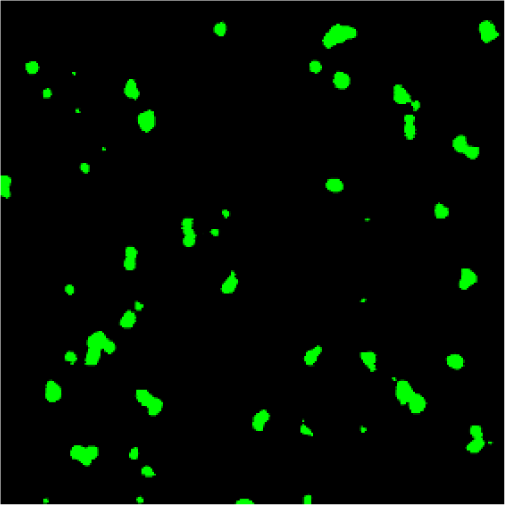

Supplement: Supplementary file 22 — Source data Fig. 4 [file 44318_2024_212_MOESM22_ESM.zip › Source Data For Figure4/4A/5 μm IRTKS.tif]

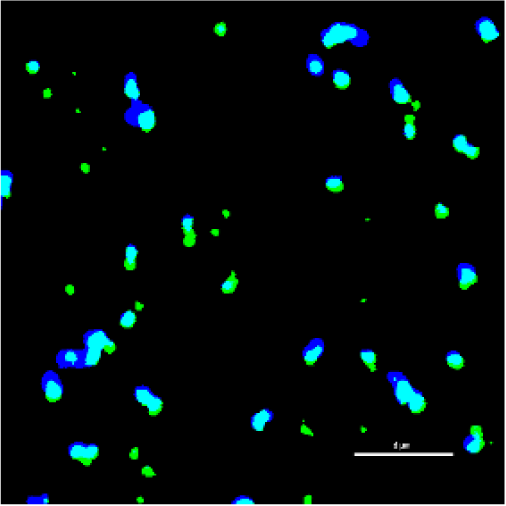

Supplement: Supplementary file 22 — Source data Fig. 4 [file 44318_2024_212_MOESM22_ESM.zip › Source Data For Figure4/4A/5 μm Merge.tif]

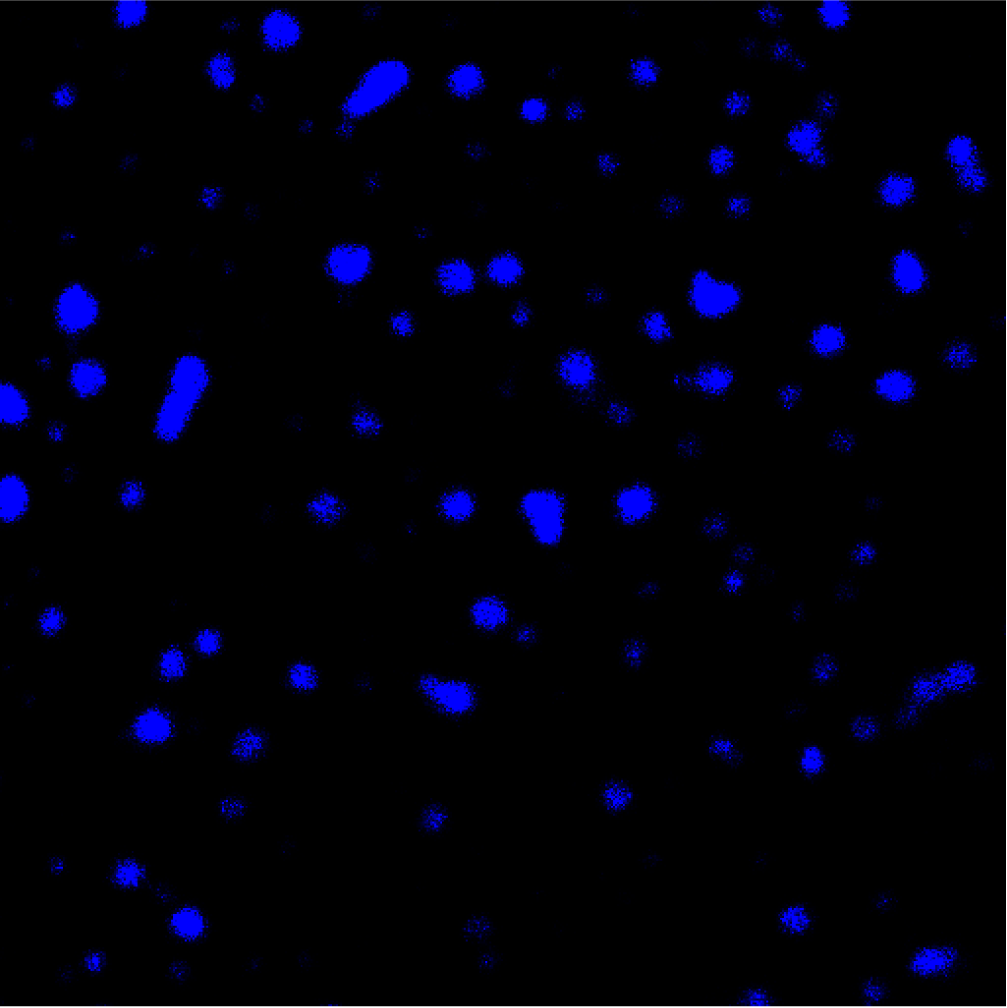

Supplement: Supplementary file 22 — Source data Fig. 4 [file 44318_2024_212_MOESM22_ESM.zip › Source Data For Figure4/4C/10 μm DAPI.tif]

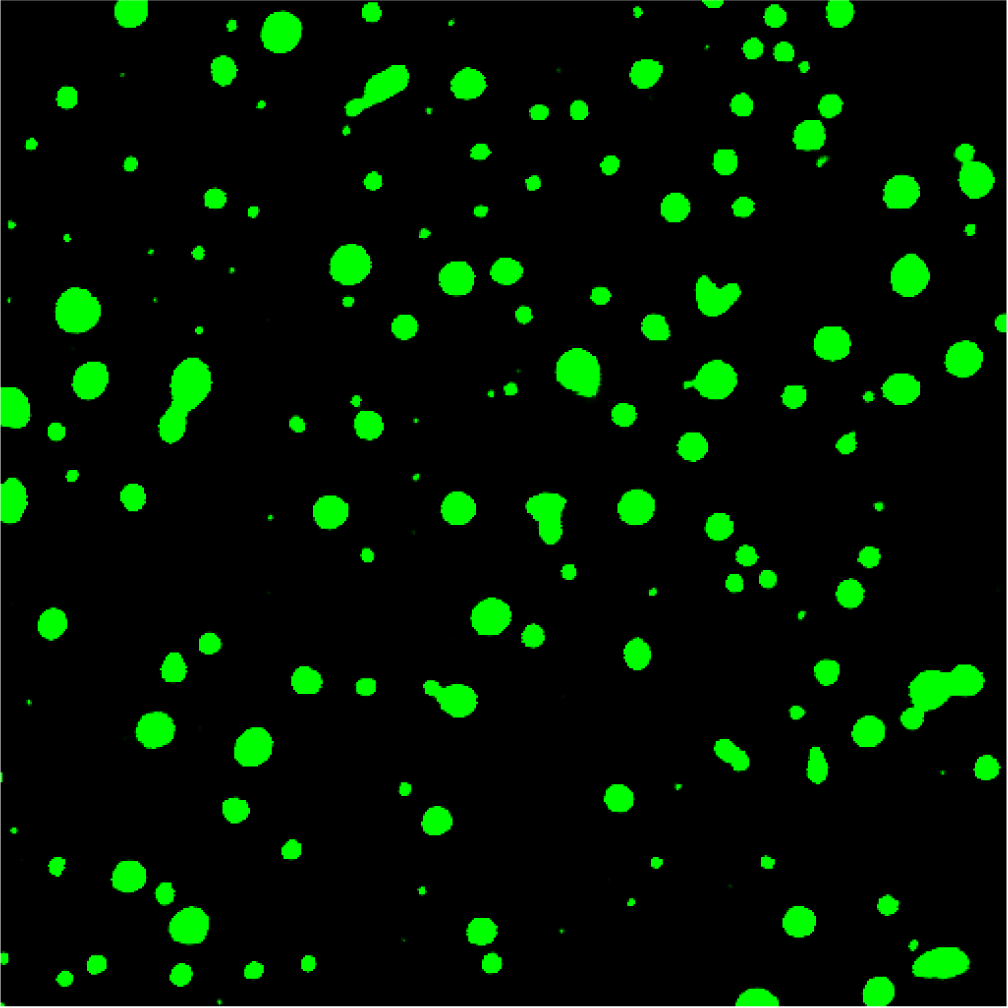

Supplement: Supplementary file 22 — Source data Fig. 4 [file 44318_2024_212_MOESM22_ESM.zip › Source Data For Figure4/4C/10 μm IRTKS.tif]

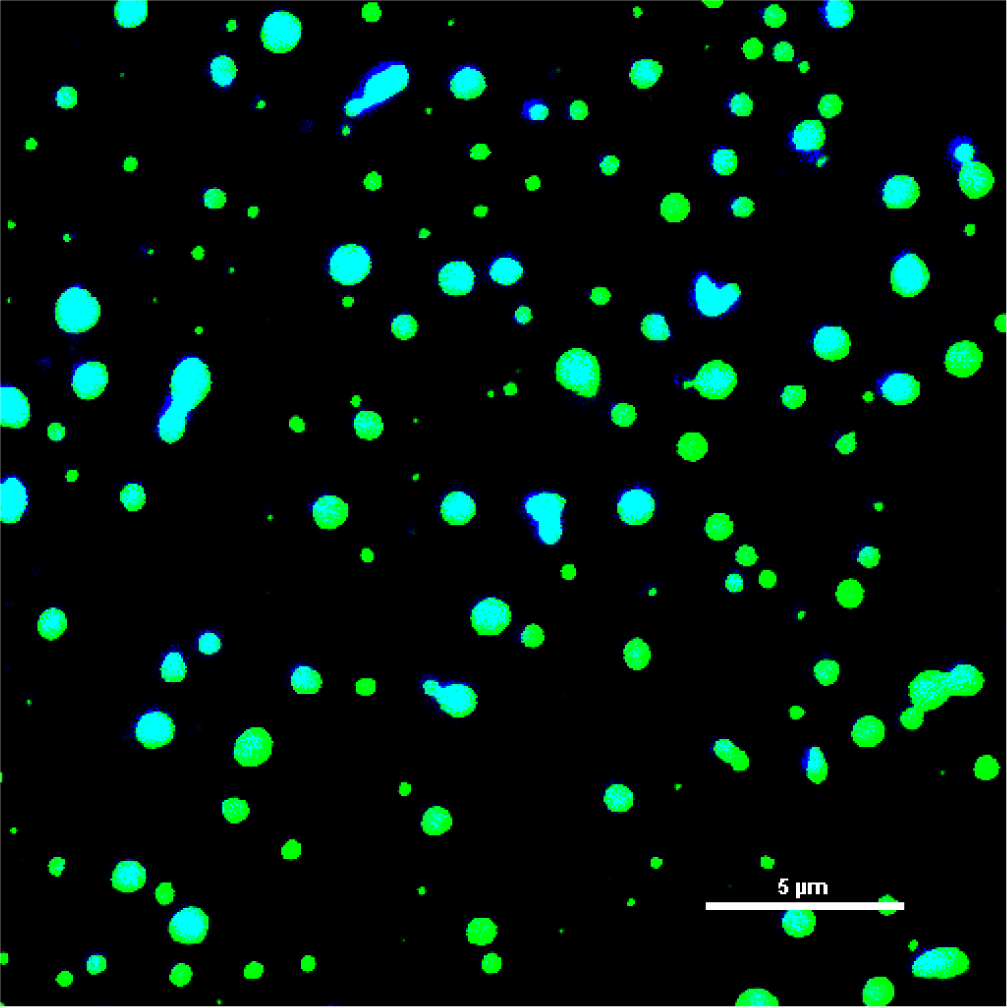

Supplement: Supplementary file 22 — Source data Fig. 4 [file 44318_2024_212_MOESM22_ESM.zip › Source Data For Figure4/4C/10 μm Merge.tif]

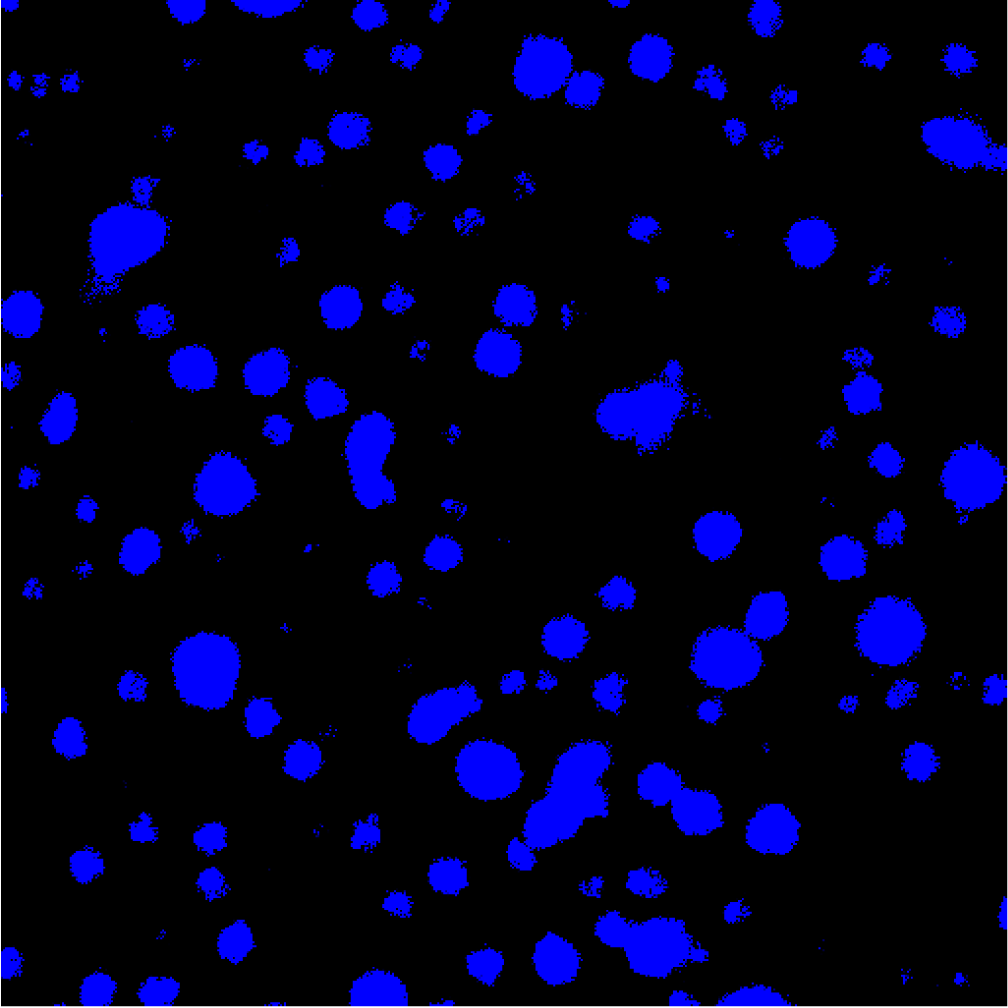

Supplement: Supplementary file 22 — Source data Fig. 4 [file 44318_2024_212_MOESM22_ESM.zip › Source Data For Figure4/4C/20 μm DAPI.tif]

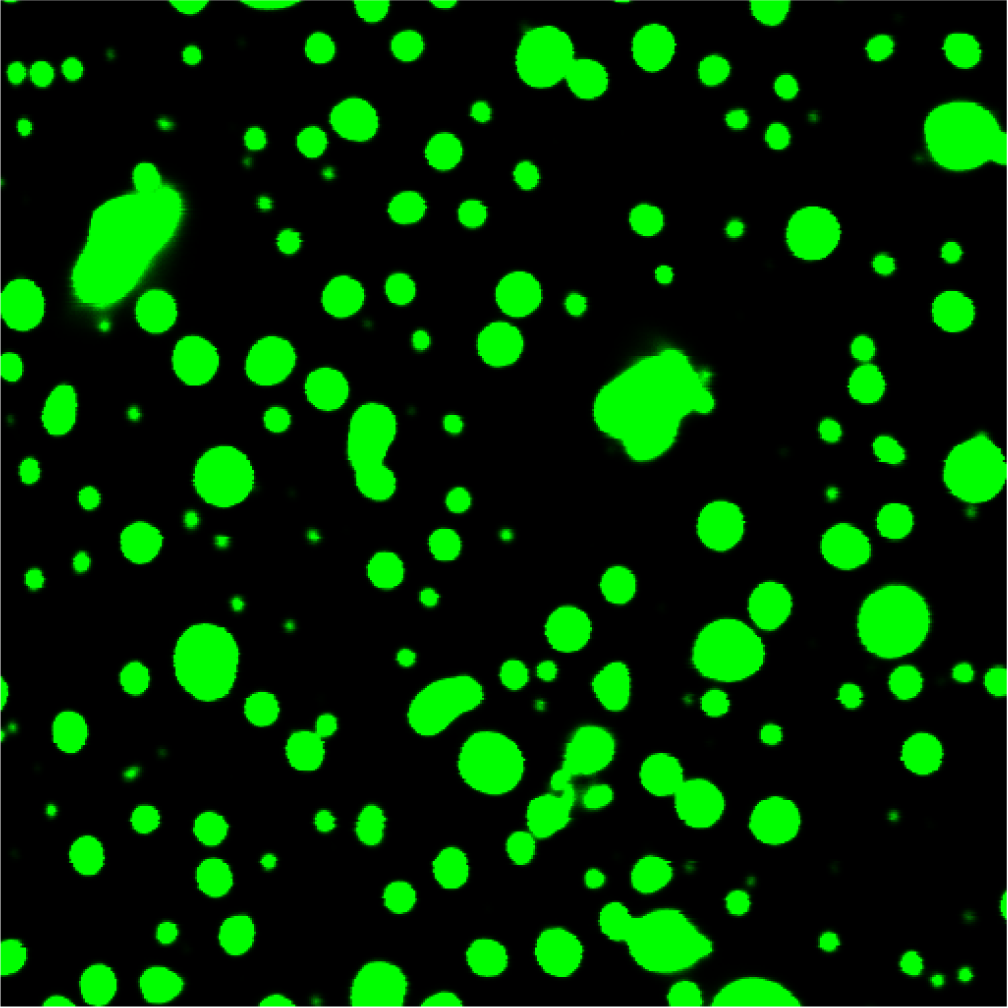

Supplement: Supplementary file 22 — Source data Fig. 4 [file 44318_2024_212_MOESM22_ESM.zip › Source Data For Figure4/4C/20 μm IRTKS.tif]

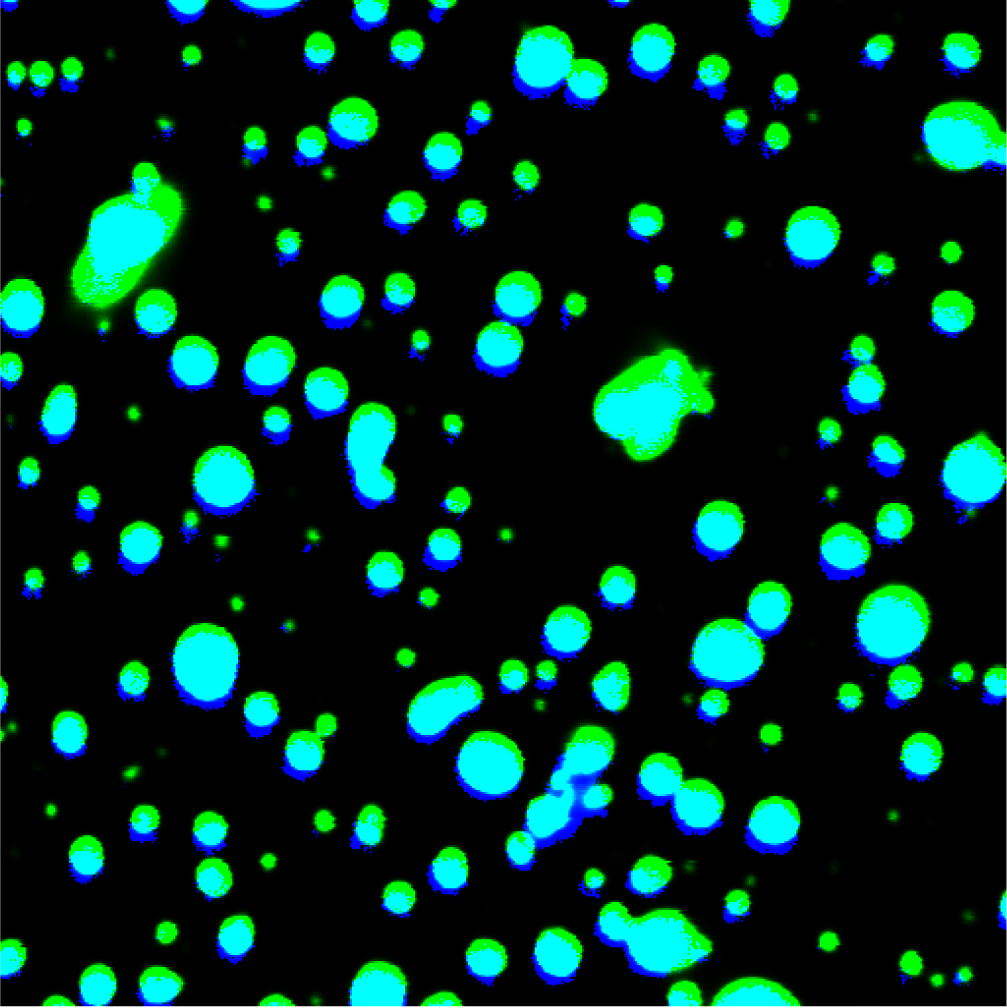

Supplement: Supplementary file 22 — Source data Fig. 4 [file 44318_2024_212_MOESM22_ESM.zip › Source Data For Figure4/4C/20 μm Merge.tif]

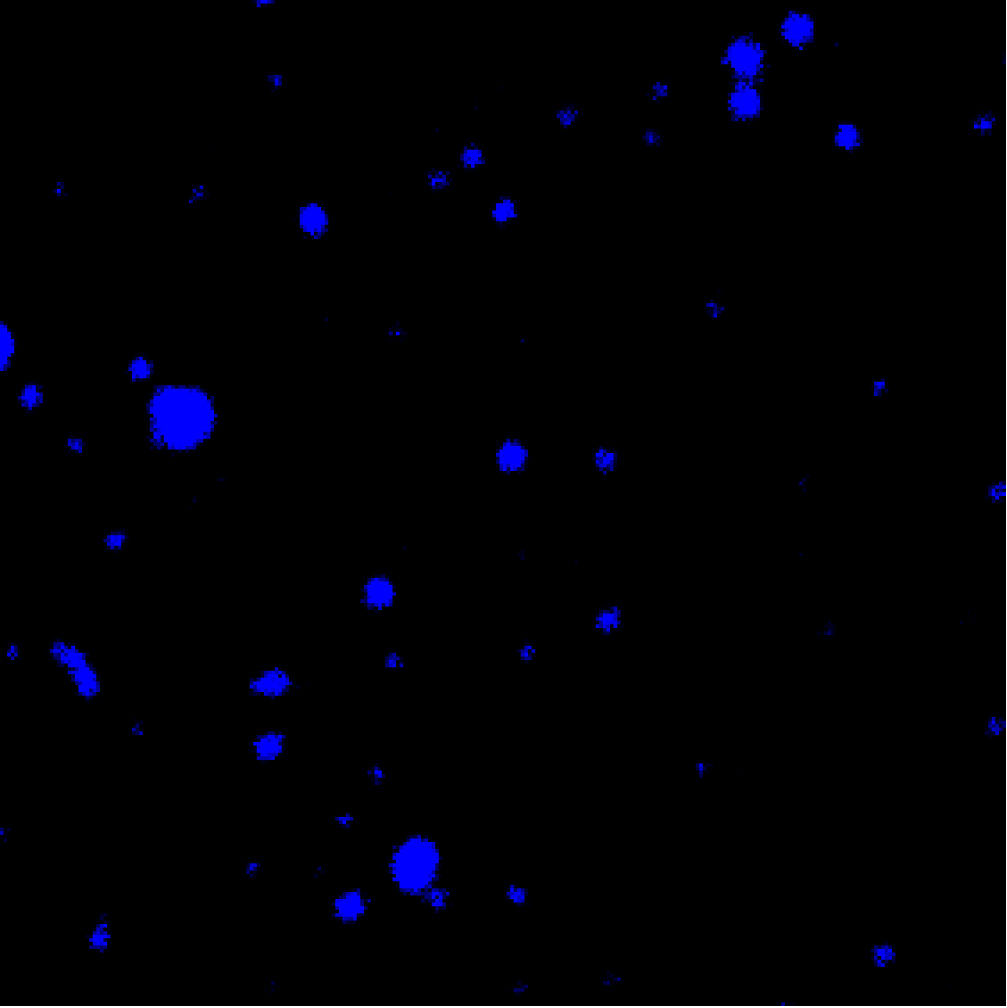

Supplement: Supplementary file 22 — Source data Fig. 4 [file 44318_2024_212_MOESM22_ESM.zip › Source Data For Figure4/4C/5 μm DAPI.tif]

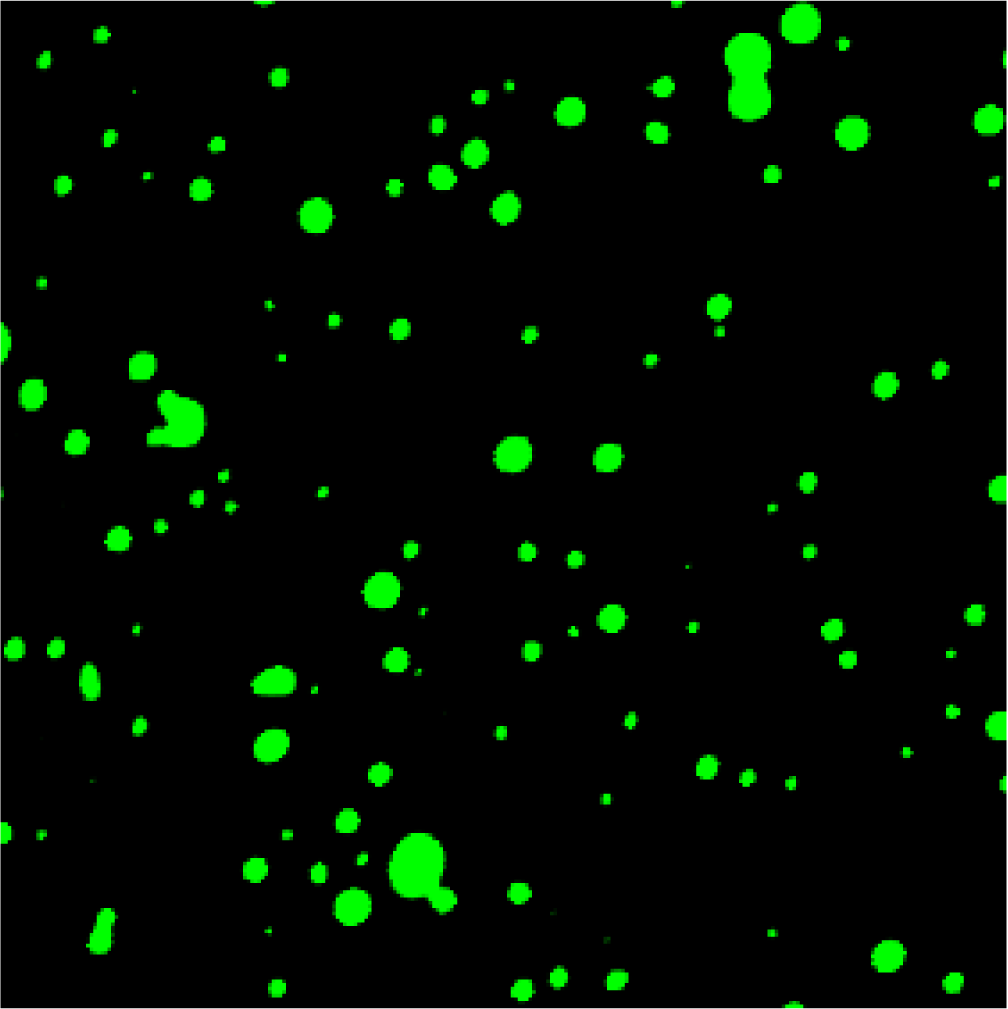

Supplement: Supplementary file 22 — Source data Fig. 4 [file 44318_2024_212_MOESM22_ESM.zip › Source Data For Figure4/4C/5 μm IRTKS.tif]

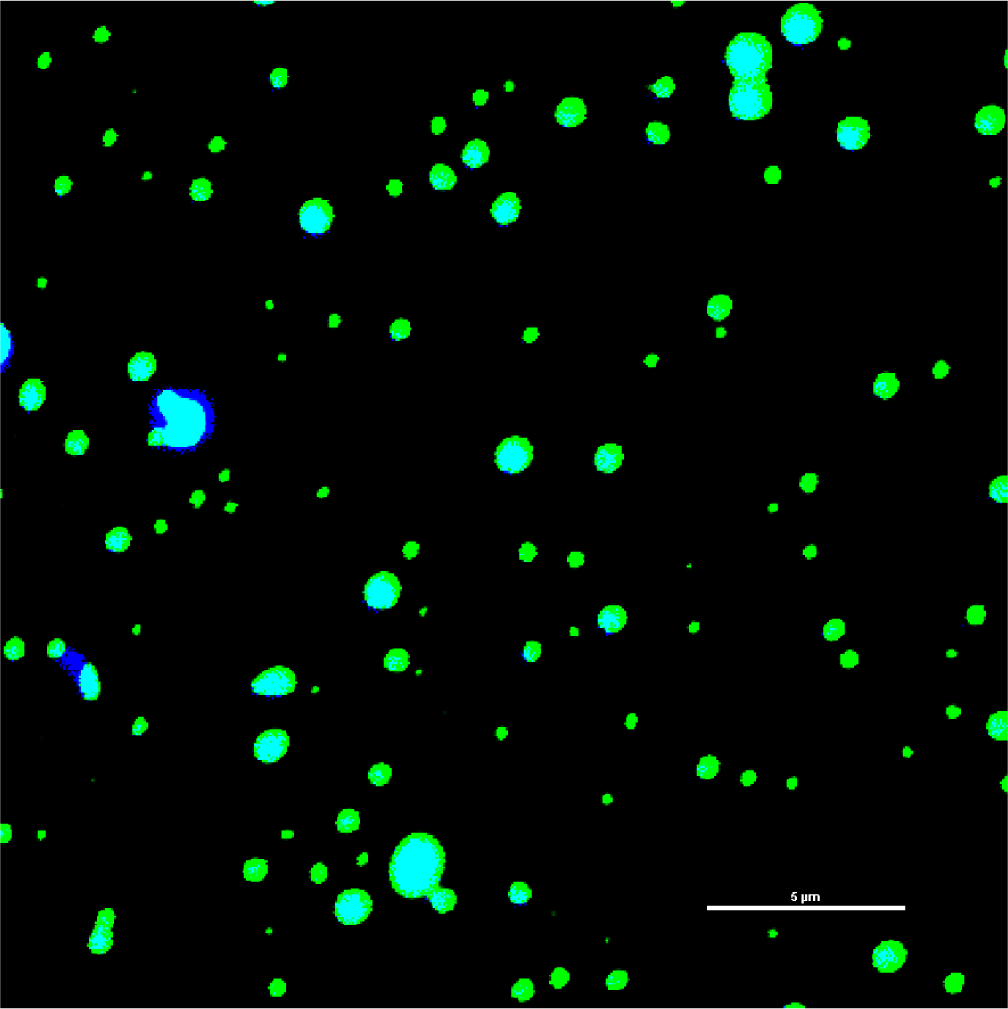

Supplement: Supplementary file 22 — Source data Fig. 4 [file 44318_2024_212_MOESM22_ESM.zip › Source Data For Figure4/4C/5 μm Merge.tif]

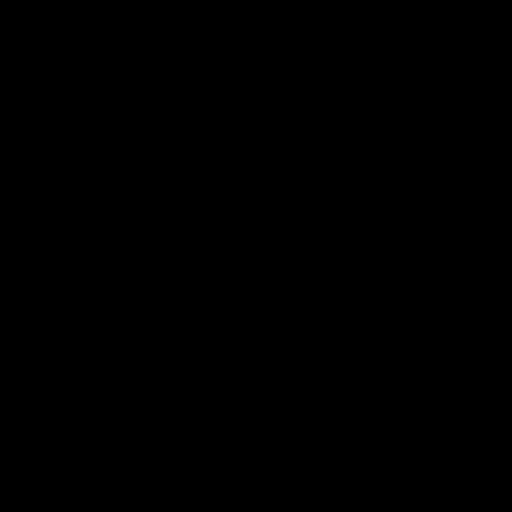

Supplement: Supplementary file 22 — Source data Fig. 4 [file 44318_2024_212_MOESM22_ESM.zip › Source Data For Figure4/4E/0 irtks 0 hp1a 500c1.tif]

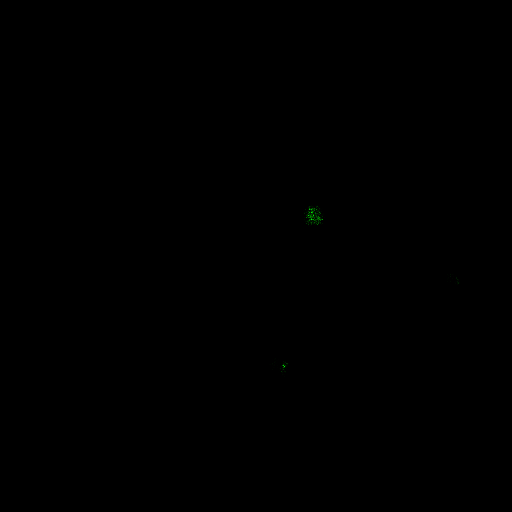

Supplement: Supplementary file 22 — Source data Fig. 4 [file 44318_2024_212_MOESM22_ESM.zip › Source Data For Figure4/4E/0 irtks 20 hp1a 500x c1.tif]

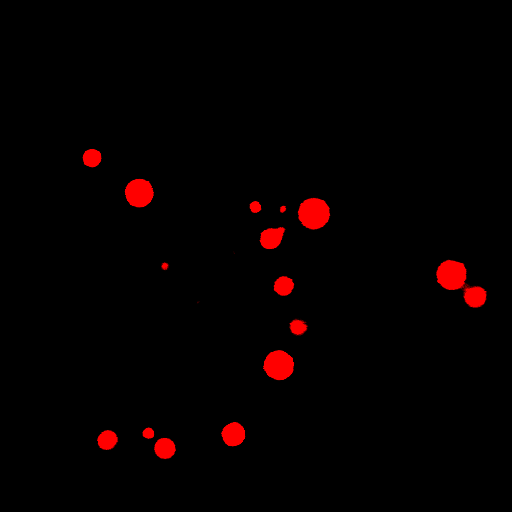

Supplement: Supplementary file 22 — Source data Fig. 4 [file 44318_2024_212_MOESM22_ESM.zip › Source Data For Figure4/4E/0 irtks 20 hp1a 500x c2.tif]

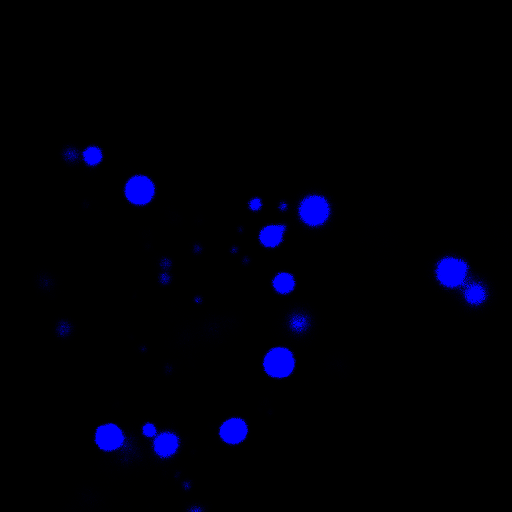

Supplement: Supplementary file 22 — Source data Fig. 4 [file 44318_2024_212_MOESM22_ESM.zip › Source Data For Figure4/4E/0 irtks 20 hp1a 500x c3.tif]

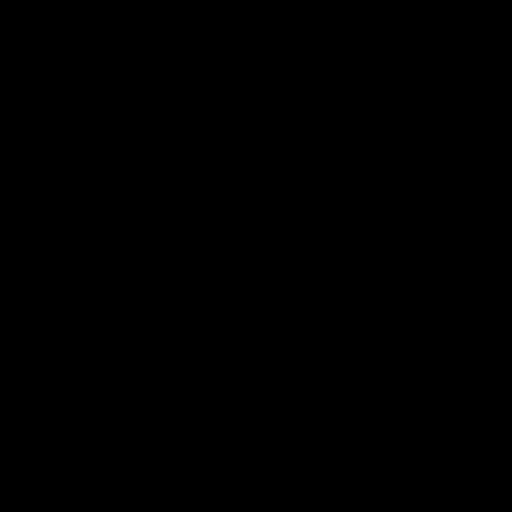

Supplement: Supplementary file 22 — Source data Fig. 4 [file 44318_2024_212_MOESM22_ESM.zip › Source Data For Figure4/4E/0 irtks 40 hp1a c1.tif]

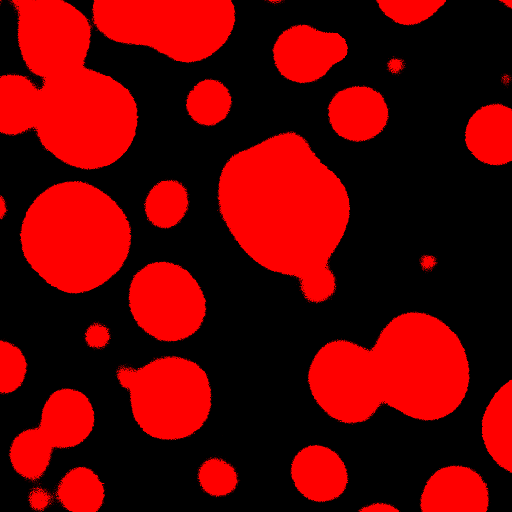

Supplement: Supplementary file 22 — Source data Fig. 4 [file 44318_2024_212_MOESM22_ESM.zip › Source Data For Figure4/4E/0 irtks 40 hp1a c2.tif]

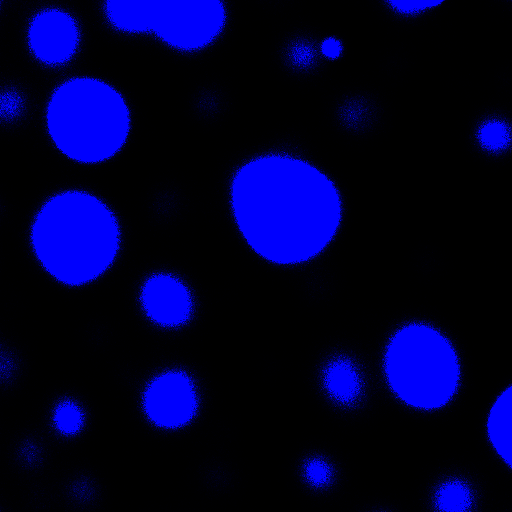

Supplement: Supplementary file 22 — Source data Fig. 4 [file 44318_2024_212_MOESM22_ESM.zip › Source Data For Figure4/4E/0 irtks 40 hp1a c3.tif]

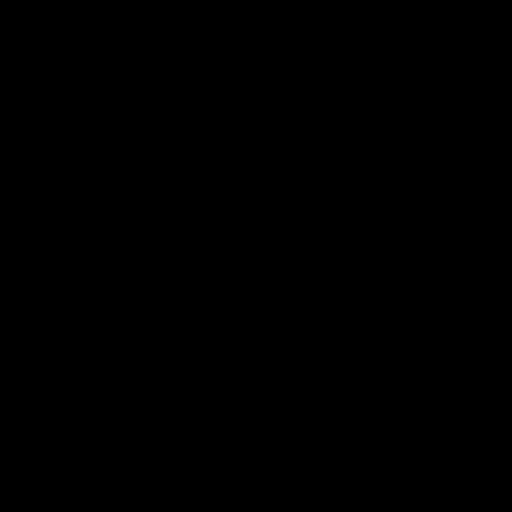

Supplement: Supplementary file 22 — Source data Fig. 4 [file 44318_2024_212_MOESM22_ESM.zip › Source Data For Figure4/4E/0 irtks 80 hp1a c1.tif]

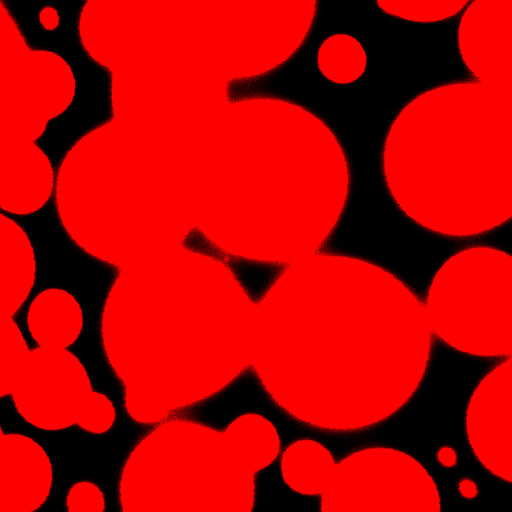

Supplement: Supplementary file 22 — Source data Fig. 4 [file 44318_2024_212_MOESM22_ESM.zip › Source Data For Figure4/4E/0 irtks 80 hp1a c2.tif]

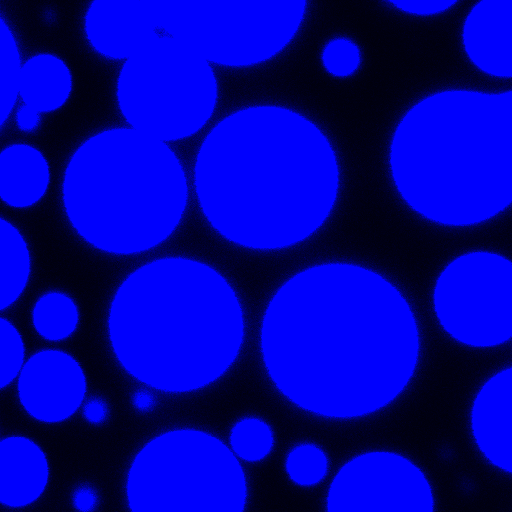

Supplement: Supplementary file 22 — Source data Fig. 4 [file 44318_2024_212_MOESM22_ESM.zip › Source Data For Figure4/4E/0 irtks 80 hp1a c3.tif]

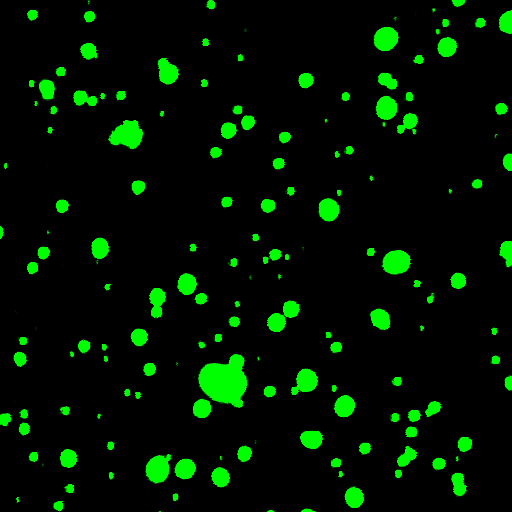

Supplement: Supplementary file 22 — Source data Fig. 4 [file 44318_2024_212_MOESM22_ESM.zip › Source Data For Figure4/4E/10 irtks 0 hp1a 500x c1.tif]

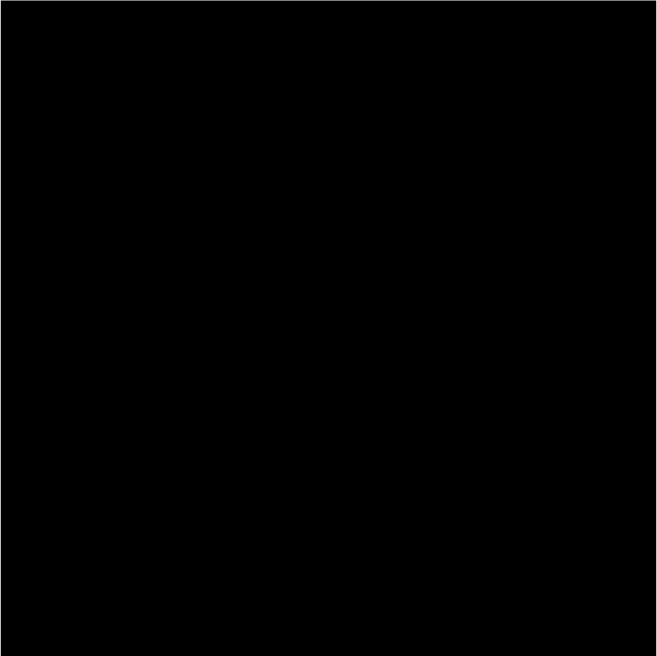

Supplement: Supplementary file 22 — Source data Fig. 4 [file 44318_2024_212_MOESM22_ESM.zip › Source Data For Figure4/4E/10 irtks 0 hp1a 500x c2.tif]

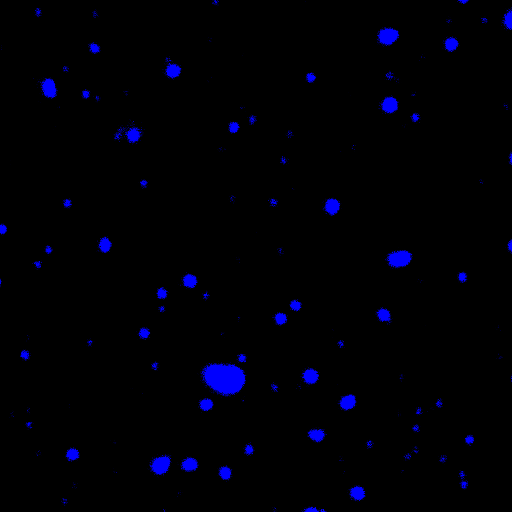

Supplement: Supplementary file 22 — Source data Fig. 4 [file 44318_2024_212_MOESM22_ESM.zip › Source Data For Figure4/4E/10 irtks 0 hp1a 500x c3.tif]

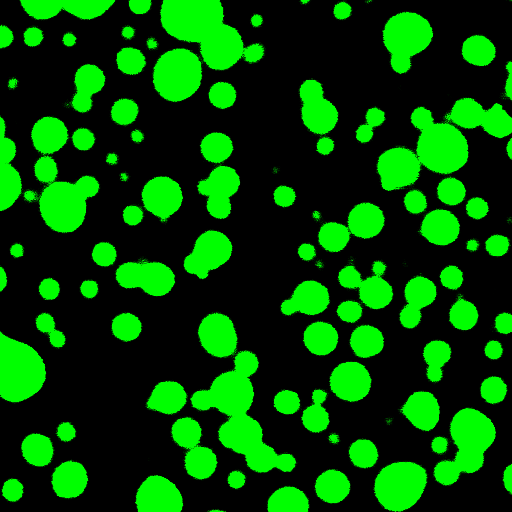

Supplement: Supplementary file 22 — Source data Fig. 4 [file 44318_2024_212_MOESM22_ESM.zip › Source Data For Figure4/4E/10 irtks 20 hp1a 500x 4c1.tif]

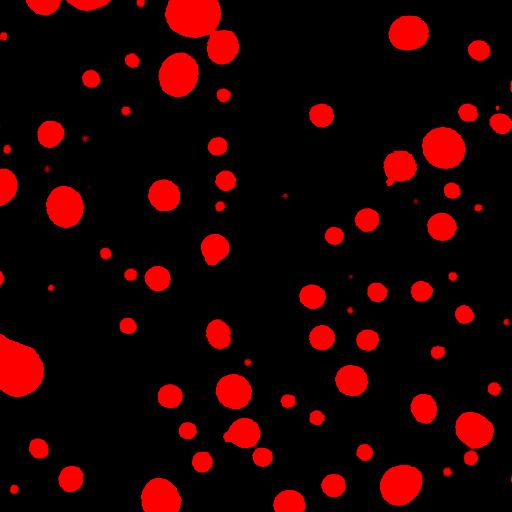

Supplement: Supplementary file 22 — Source data Fig. 4 [file 44318_2024_212_MOESM22_ESM.zip › Source Data For Figure4/4E/10 irtks 20 hp1a 500x 4c2.tif]

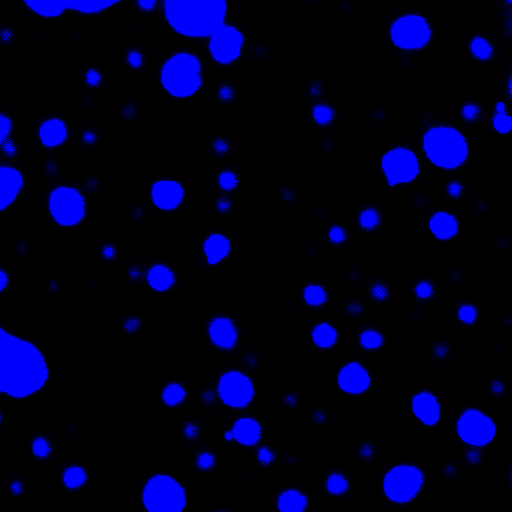

Supplement: Supplementary file 22 — Source data Fig. 4 [file 44318_2024_212_MOESM22_ESM.zip › Source Data For Figure4/4E/10 irtks 20 hp1a 500x 4c3.tif]

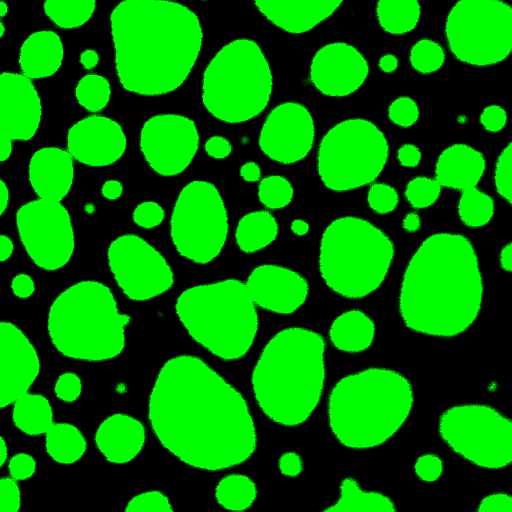

Supplement: Supplementary file 22 — Source data Fig. 4 [file 44318_2024_212_MOESM22_ESM.zip › Source Data For Figure4/4E/10 irtks 40 hp1a 500x 4c1.tif]
