# Supplementary figures and images for: Heterochromatin formation and remodeling by IRTKS condensates counteract cellular senescence (part 2 of 4)
Source: EMBO J. 2024 Aug 27;43(20):7. doi: 10.1038/s44318-024-00212-3 (PMC11480336; doi:10.1038/s44318-024-00212-3)

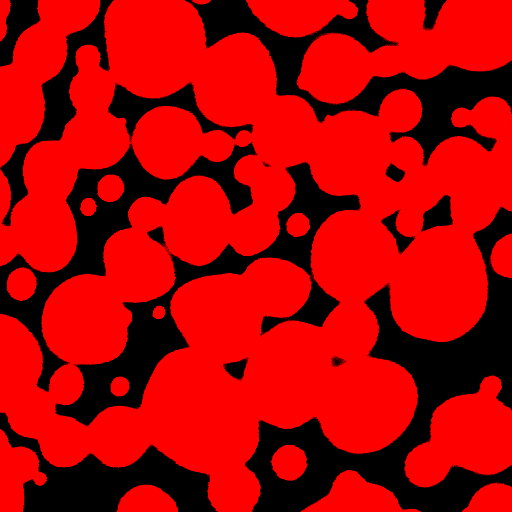

Supplement: Supplementary file 22 — Source data Fig. 4 [file 44318_2024_212_MOESM22_ESM.zip › Source Data For Figure4/4E/10 irtks 40 hp1a 500x 4c2.tif]

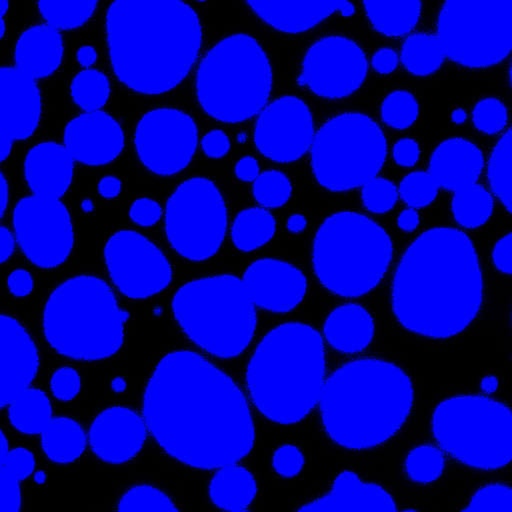

Supplement: Supplementary file 22 — Source data Fig. 4 [file 44318_2024_212_MOESM22_ESM.zip › Source Data For Figure4/4E/10 irtks 40 hp1a 500x 4c3.tif]

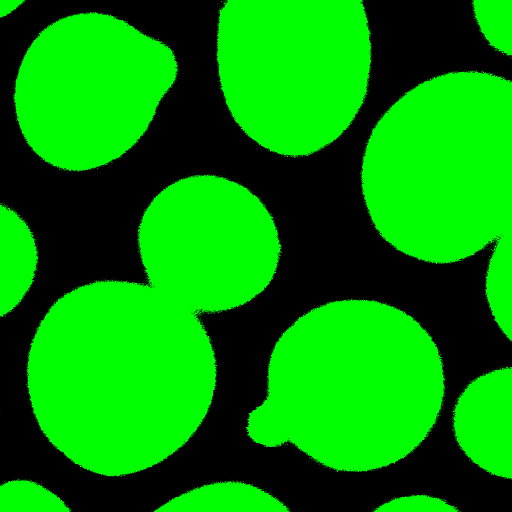

Supplement: Supplementary file 22 — Source data Fig. 4 [file 44318_2024_212_MOESM22_ESM.zip › Source Data For Figure4/4E/10 irtks 80 hp1a 500x 3c1.tif]

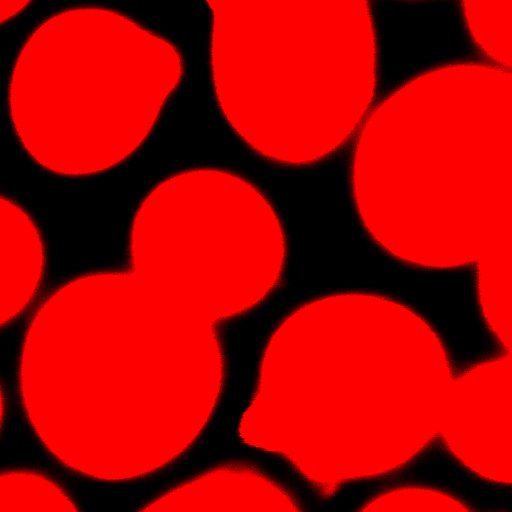

Supplement: Supplementary file 22 — Source data Fig. 4 [file 44318_2024_212_MOESM22_ESM.zip › Source Data For Figure4/4E/10 irtks 80 hp1a 500x 3c2.tif]

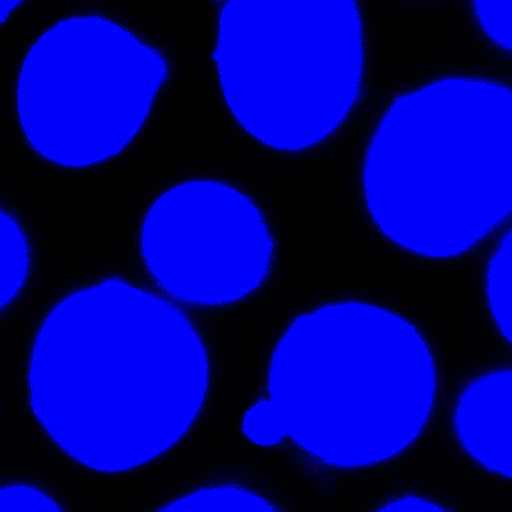

Supplement: Supplementary file 22 — Source data Fig. 4 [file 44318_2024_212_MOESM22_ESM.zip › Source Data For Figure4/4E/10 irtks 80 hp1a 500x 3c3.tif]

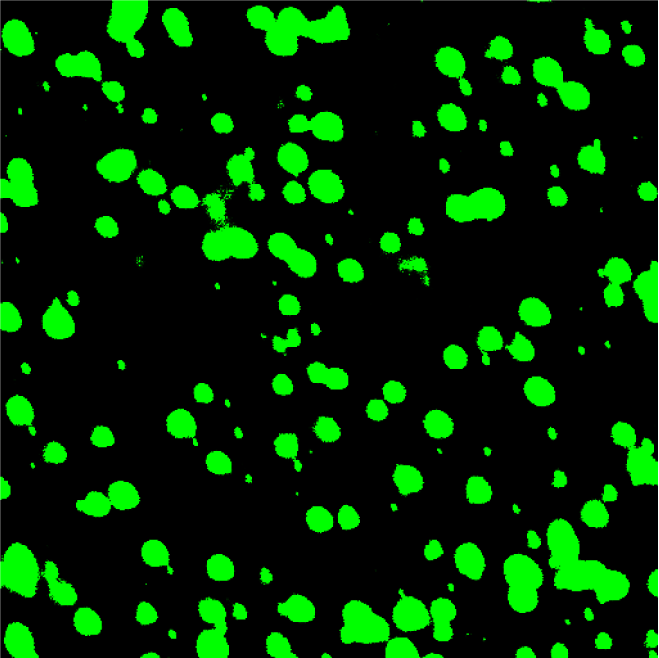

Supplement: Supplementary file 22 — Source data Fig. 4 [file 44318_2024_212_MOESM22_ESM.zip › Source Data For Figure4/4E/20 irtks 0 hp1a 500x c1.tif]

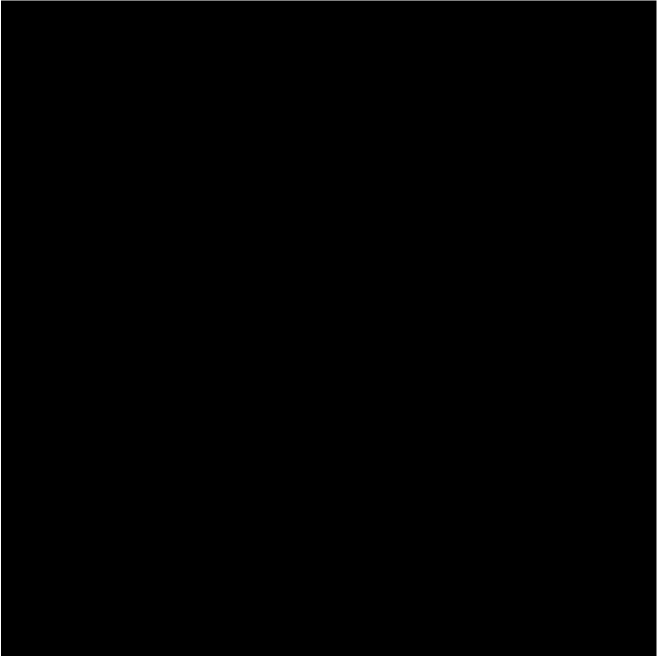

Supplement: Supplementary file 22 — Source data Fig. 4 [file 44318_2024_212_MOESM22_ESM.zip › Source Data For Figure4/4E/20 irtks 0 hp1a 500x c2.tif]

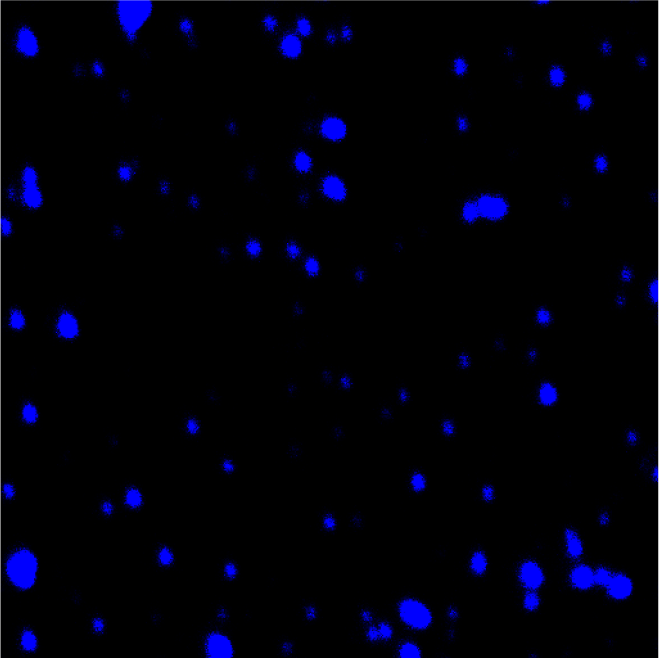

Supplement: Supplementary file 22 — Source data Fig. 4 [file 44318_2024_212_MOESM22_ESM.zip › Source Data For Figure4/4E/20 irtks 0 hp1a 500x c3.tif]

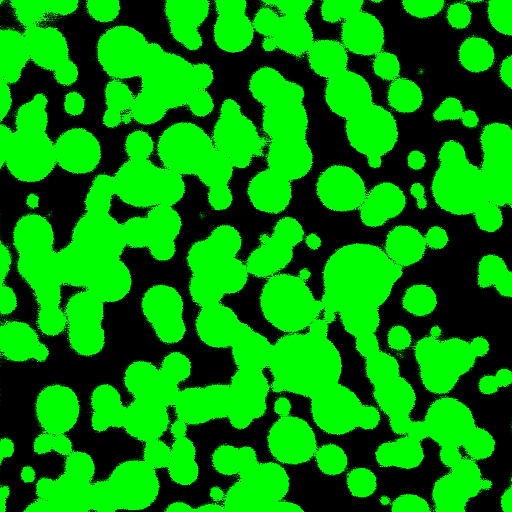

Supplement: Supplementary file 22 — Source data Fig. 4 [file 44318_2024_212_MOESM22_ESM.zip › Source Data For Figure4/4E/20 irtks 20 hp1a 500x 2c1.tif]

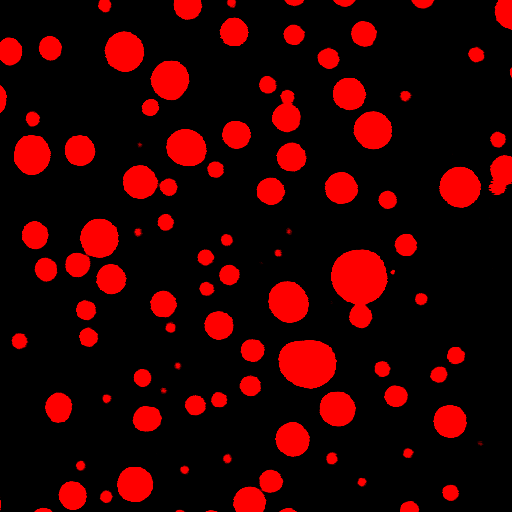

Supplement: Supplementary file 22 — Source data Fig. 4 [file 44318_2024_212_MOESM22_ESM.zip › Source Data For Figure4/4E/20 irtks 20 hp1a 500x 2c2.tif]

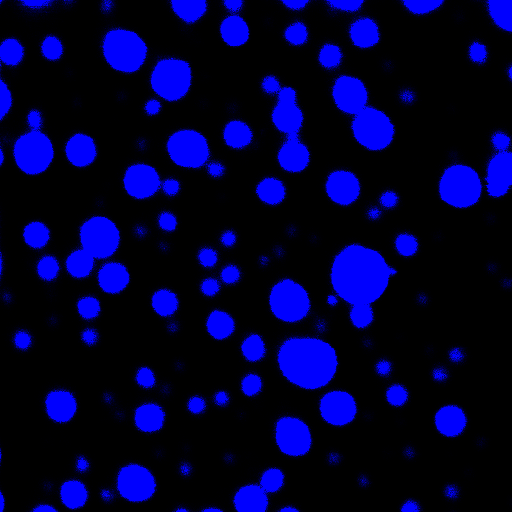

Supplement: Supplementary file 22 — Source data Fig. 4 [file 44318_2024_212_MOESM22_ESM.zip › Source Data For Figure4/4E/20 irtks 20 hp1a 500x 2c3.tif]

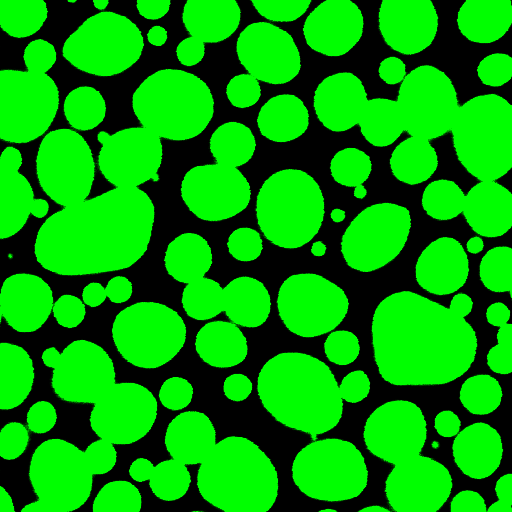

Supplement: Supplementary file 22 — Source data Fig. 4 [file 44318_2024_212_MOESM22_ESM.zip › Source Data For Figure4/4E/20 irtks 40 hp1a 500x 4c1.tif]

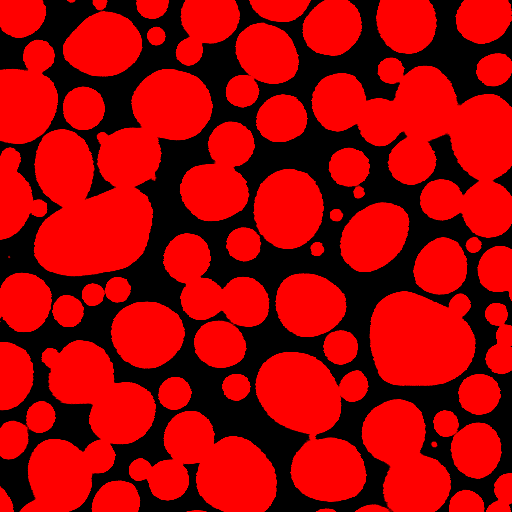

Supplement: Supplementary file 22 — Source data Fig. 4 [file 44318_2024_212_MOESM22_ESM.zip › Source Data For Figure4/4E/20 irtks 40 hp1a 500x 4c2.tif]

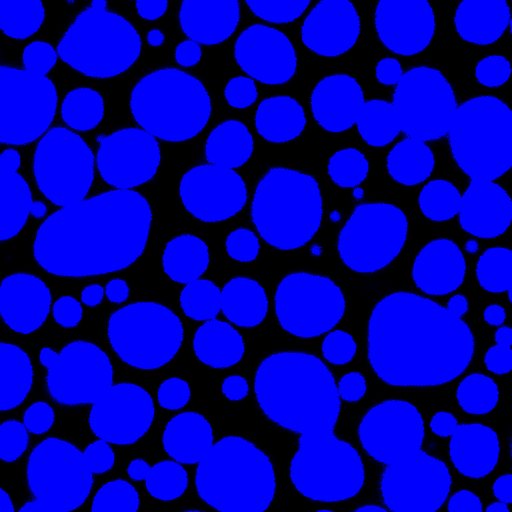

Supplement: Supplementary file 22 — Source data Fig. 4 [file 44318_2024_212_MOESM22_ESM.zip › Source Data For Figure4/4E/20 irtks 40 hp1a 500x 4c3.tif]

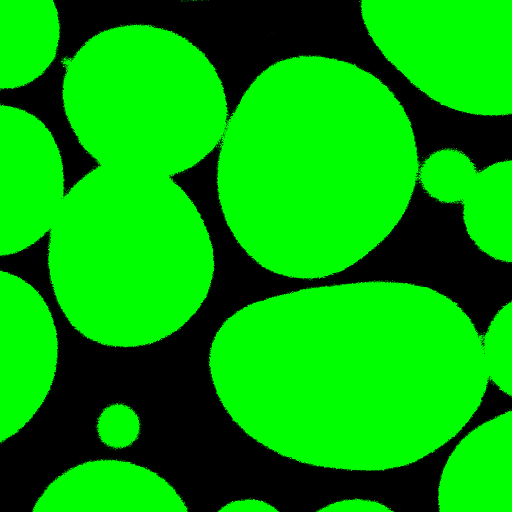

Supplement: Supplementary file 22 — Source data Fig. 4 [file 44318_2024_212_MOESM22_ESM.zip › Source Data For Figure4/4E/20 irtks 80 hp1a 500x 1c1.tif]

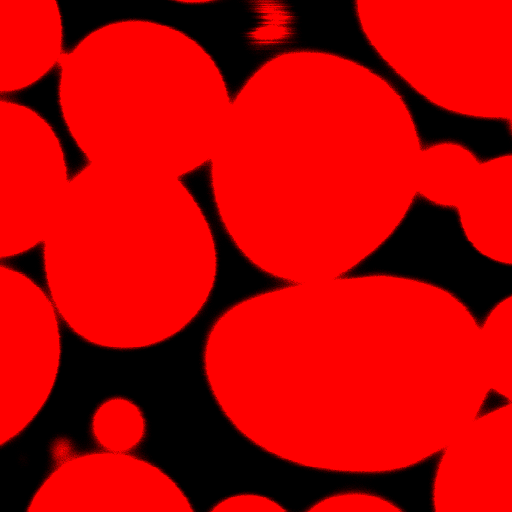

Supplement: Supplementary file 22 — Source data Fig. 4 [file 44318_2024_212_MOESM22_ESM.zip › Source Data For Figure4/4E/20 irtks 80 hp1a 500x 1c2.tif]

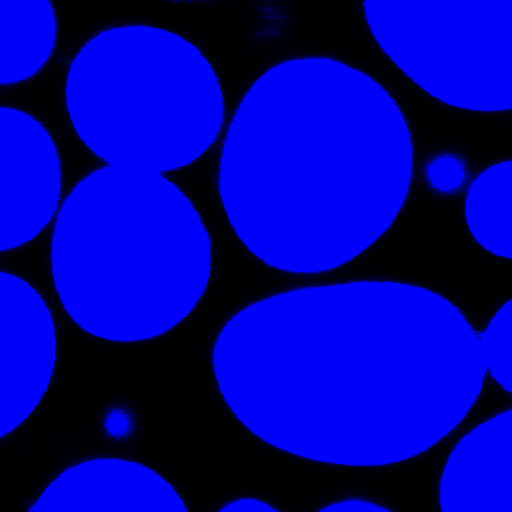

Supplement: Supplementary file 22 — Source data Fig. 4 [file 44318_2024_212_MOESM22_ESM.zip › Source Data For Figure4/4E/20 irtks 80 hp1a 500x 1c3.tif]

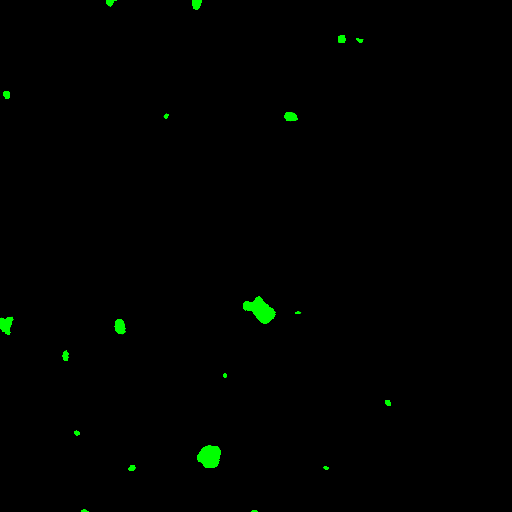

Supplement: Supplementary file 22 — Source data Fig. 4 [file 44318_2024_212_MOESM22_ESM.zip › Source Data For Figure4/4E/5 irtks 0 hp1a 500x 5c1.tif]

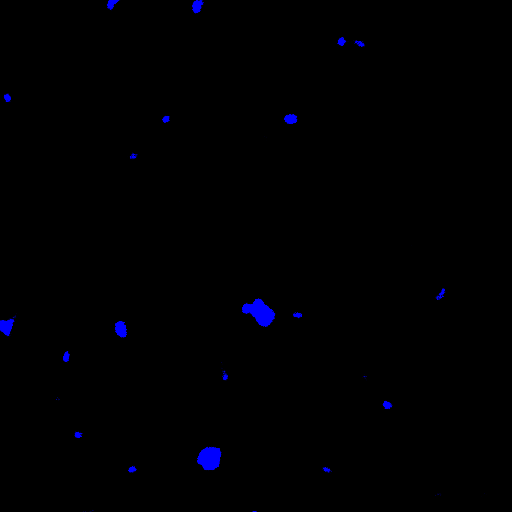

Supplement: Supplementary file 22 — Source data Fig. 4 [file 44318_2024_212_MOESM22_ESM.zip › Source Data For Figure4/4E/5 irtks 0 hp1a 500x 5c3.tif]

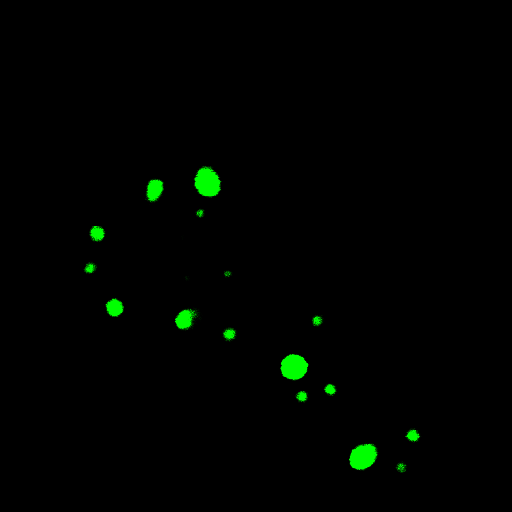

Supplement: Supplementary file 22 — Source data Fig. 4 [file 44318_2024_212_MOESM22_ESM.zip › Source Data For Figure4/4E/5 irtks 20 hp1a 500x 1c1.tif]

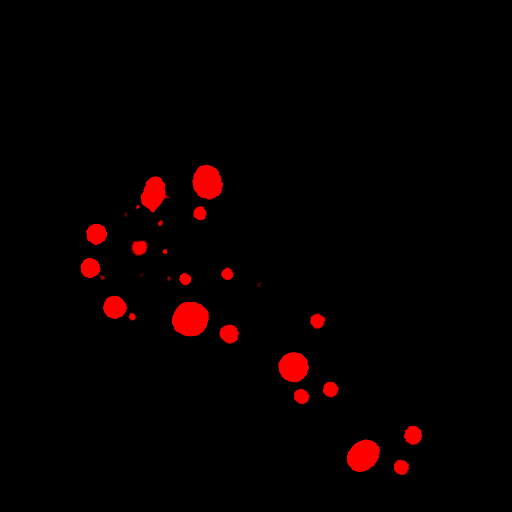

Supplement: Supplementary file 22 — Source data Fig. 4 [file 44318_2024_212_MOESM22_ESM.zip › Source Data For Figure4/4E/5 irtks 20 hp1a 500x 1c2.tif]

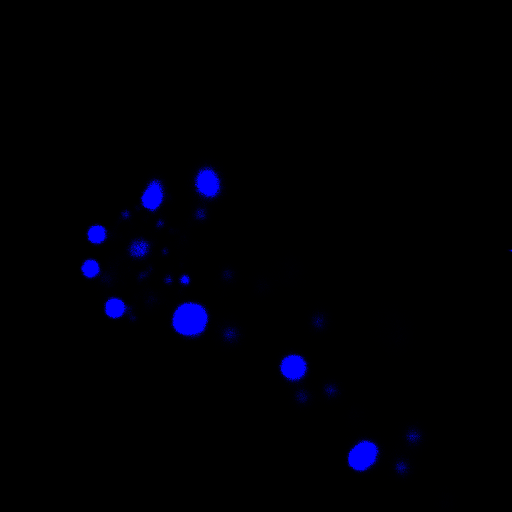

Supplement: Supplementary file 22 — Source data Fig. 4 [file 44318_2024_212_MOESM22_ESM.zip › Source Data For Figure4/4E/5 irtks 20 hp1a 500x 1c3.tif]

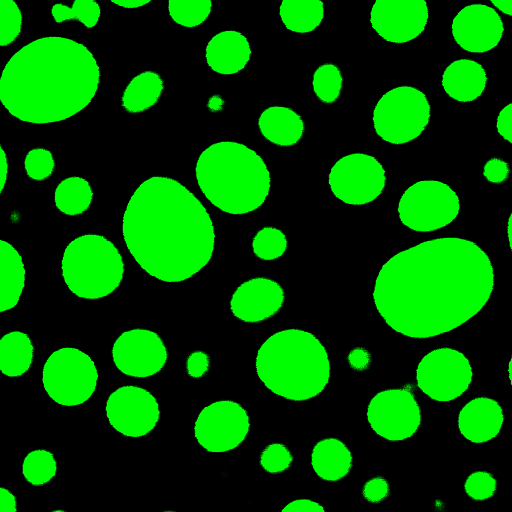

Supplement: Supplementary file 22 — Source data Fig. 4 [file 44318_2024_212_MOESM22_ESM.zip › Source Data For Figure4/4E/5 irtks 40 hp1a 500x 5c1.tif]

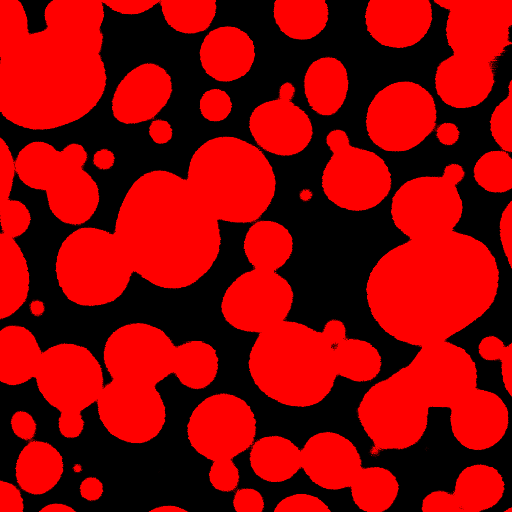

Supplement: Supplementary file 22 — Source data Fig. 4 [file 44318_2024_212_MOESM22_ESM.zip › Source Data For Figure4/4E/5 irtks 40 hp1a 500x 5c2.tif]

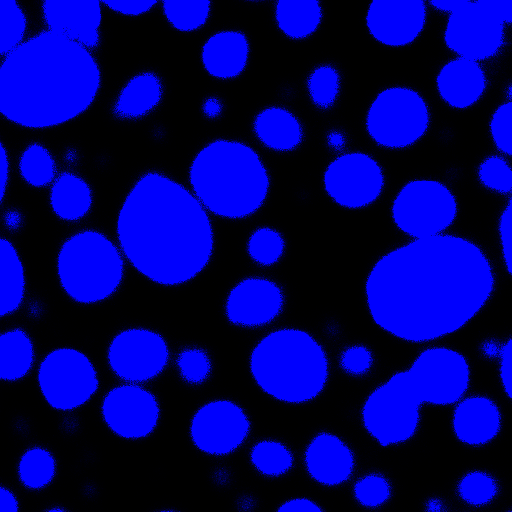

Supplement: Supplementary file 22 — Source data Fig. 4 [file 44318_2024_212_MOESM22_ESM.zip › Source Data For Figure4/4E/5 irtks 40 hp1a 500x 5c3.tif]

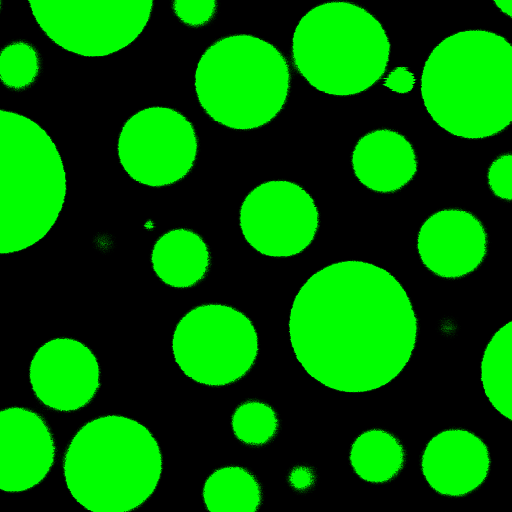

Supplement: Supplementary file 22 — Source data Fig. 4 [file 44318_2024_212_MOESM22_ESM.zip › Source Data For Figure4/4E/5 irtks 80 hp1a 500x 1c1.tif]

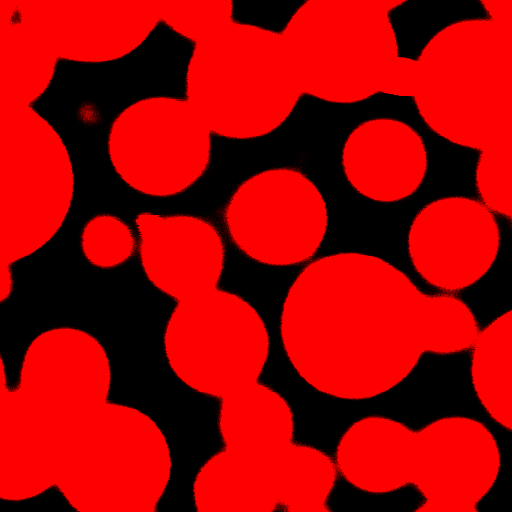

Supplement: Supplementary file 22 — Source data Fig. 4 [file 44318_2024_212_MOESM22_ESM.zip › Source Data For Figure4/4E/5 irtks 80 hp1a 500x 1c2.tif]

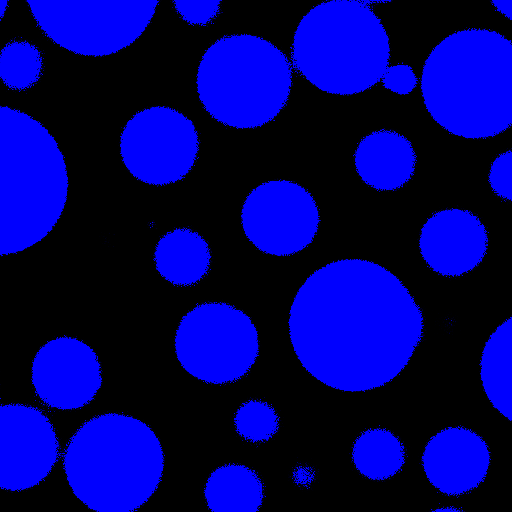

Supplement: Supplementary file 22 — Source data Fig. 4 [file 44318_2024_212_MOESM22_ESM.zip › Source Data For Figure4/4E/5 irtks 80 hp1a 500x 1c3.tif]

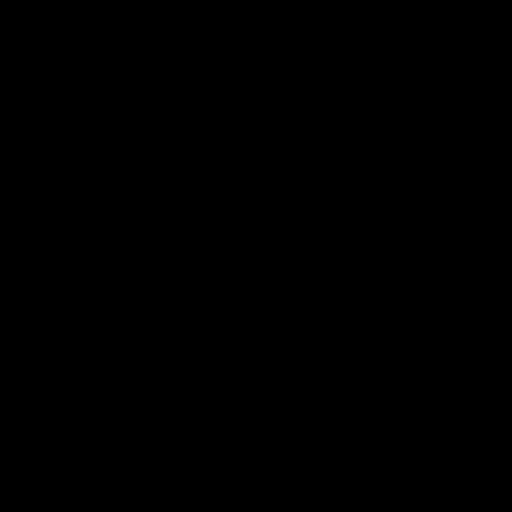

Supplement: Supplementary file 22 — Source data Fig. 4 [file 44318_2024_212_MOESM22_ESM.zip › Source Data For Figure4/4F/0 irtks 0 hp1a 500 c1.tif]

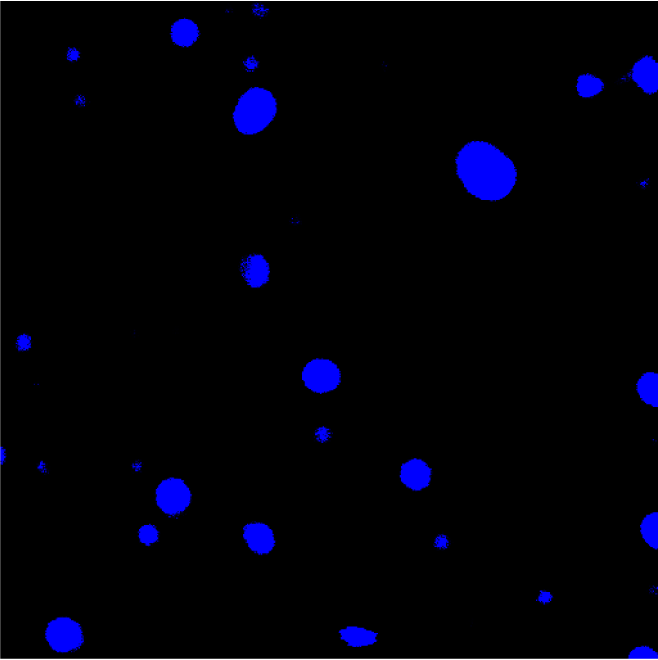

Supplement: Supplementary file 22 — Source data Fig. 4 [file 44318_2024_212_MOESM22_ESM.zip › Source Data For Figure4/4F/0 irtks 20 hp1a 500 c1.tif]

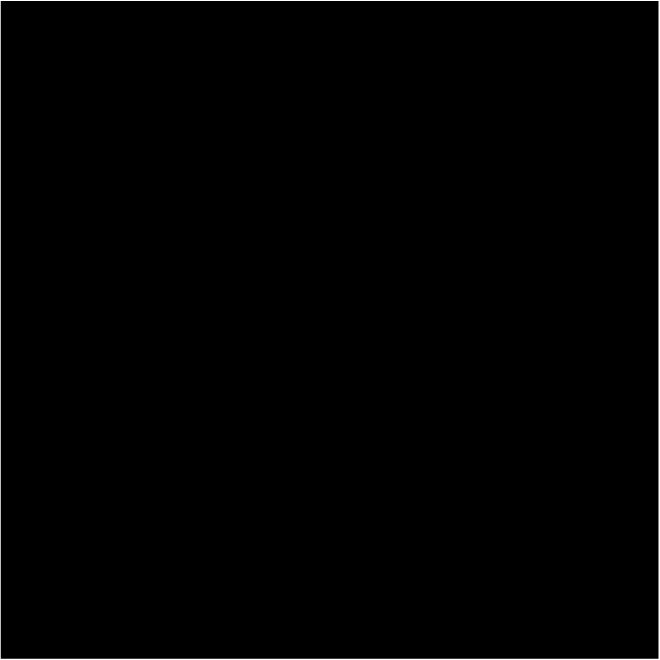

Supplement: Supplementary file 22 — Source data Fig. 4 [file 44318_2024_212_MOESM22_ESM.zip › Source Data For Figure4/4F/0 irtks 20 hp1a 500 c2.tif]

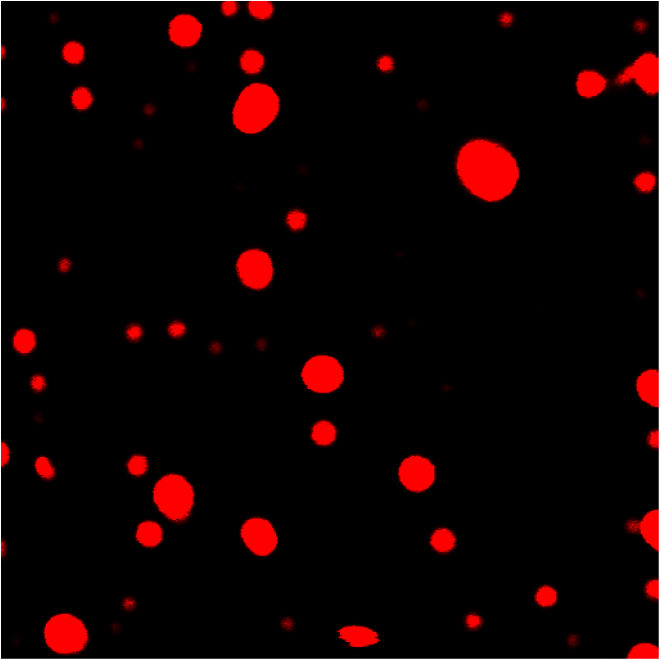

Supplement: Supplementary file 22 — Source data Fig. 4 [file 44318_2024_212_MOESM22_ESM.zip › Source Data For Figure4/4F/0 irtks 20 hp1a 500 c3.tif]

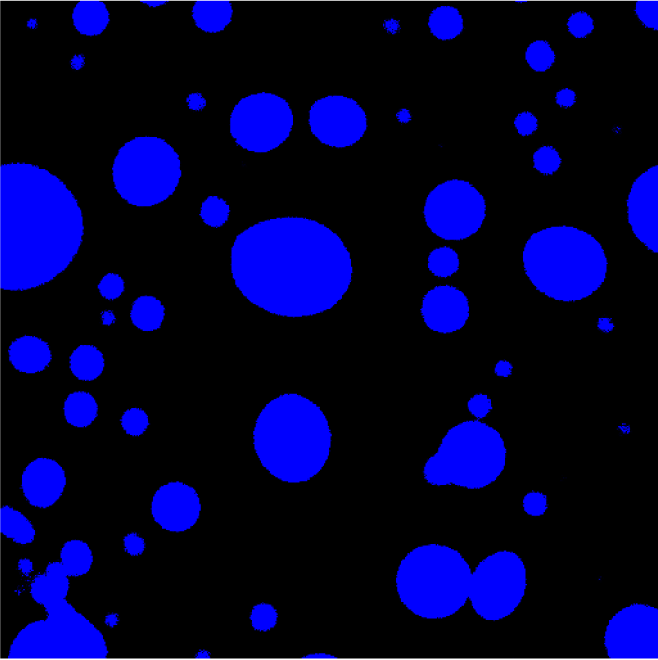

Supplement: Supplementary file 22 — Source data Fig. 4 [file 44318_2024_212_MOESM22_ESM.zip › Source Data For Figure4/4F/0 irtks 40 hp1a 500x 4c1.tif]

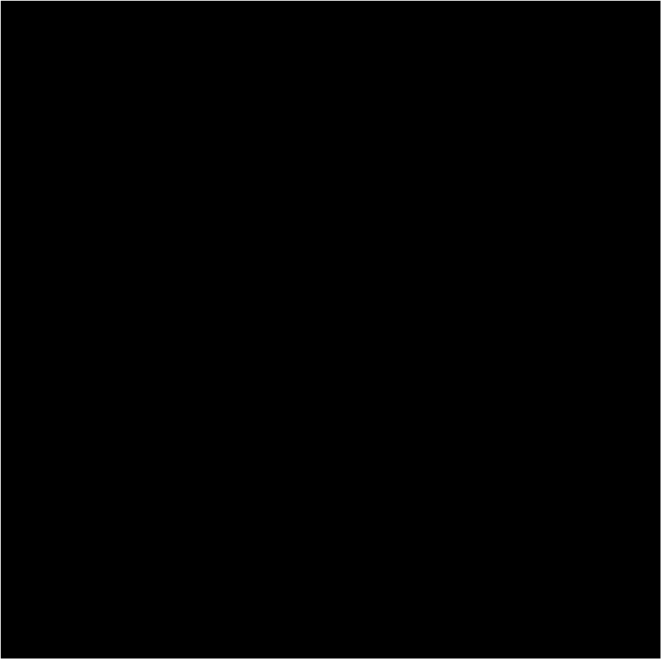

Supplement: Supplementary file 22 — Source data Fig. 4 [file 44318_2024_212_MOESM22_ESM.zip › Source Data For Figure4/4F/0 irtks 40 hp1a 500x 4c2.tif]

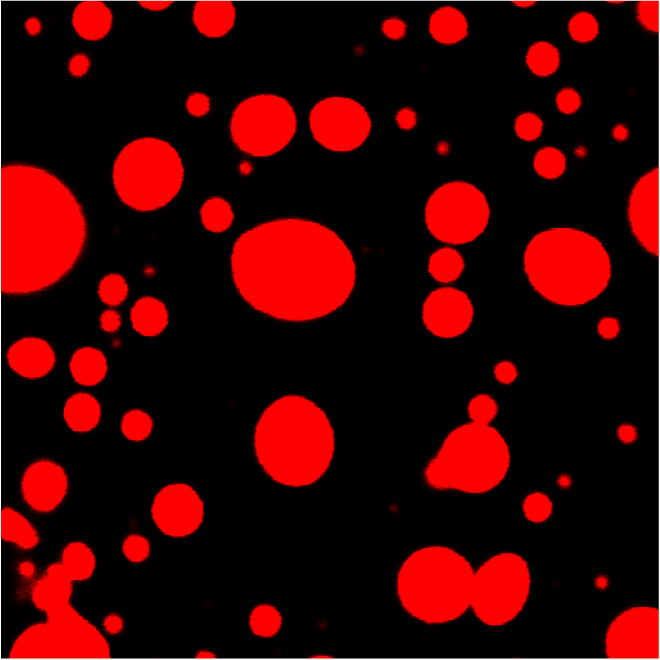

Supplement: Supplementary file 22 — Source data Fig. 4 [file 44318_2024_212_MOESM22_ESM.zip › Source Data For Figure4/4F/0 irtks 40 hp1a 500x 4c3.tif]

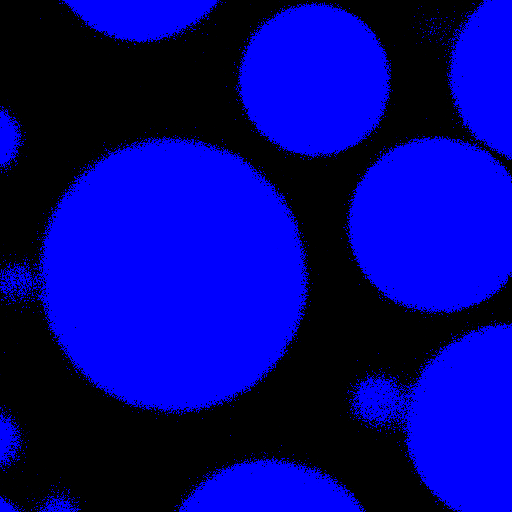

Supplement: Supplementary file 22 — Source data Fig. 4 [file 44318_2024_212_MOESM22_ESM.zip › Source Data For Figure4/4F/0 irtks 80 hp1a 500x 4c1.tif]

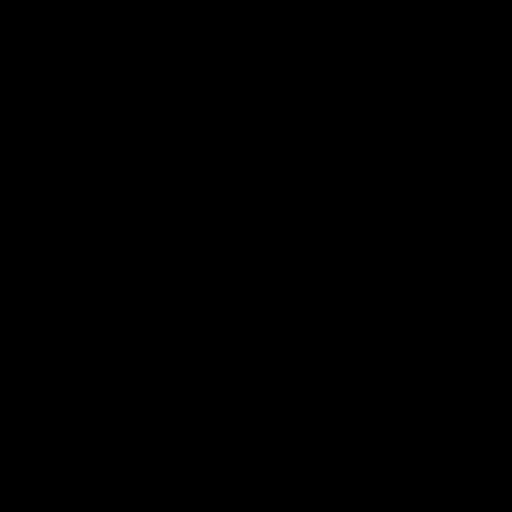

Supplement: Supplementary file 22 — Source data Fig. 4 [file 44318_2024_212_MOESM22_ESM.zip › Source Data For Figure4/4F/0 irtks 80 hp1a 500x 4c2.tif]

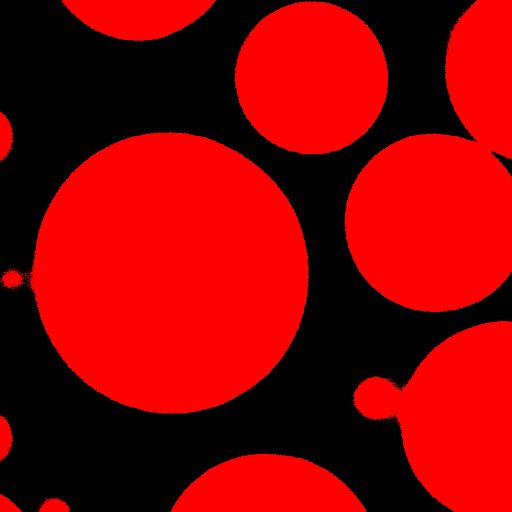

Supplement: Supplementary file 22 — Source data Fig. 4 [file 44318_2024_212_MOESM22_ESM.zip › Source Data For Figure4/4F/0 irtks 80 hp1a 500x 4c3.tif]

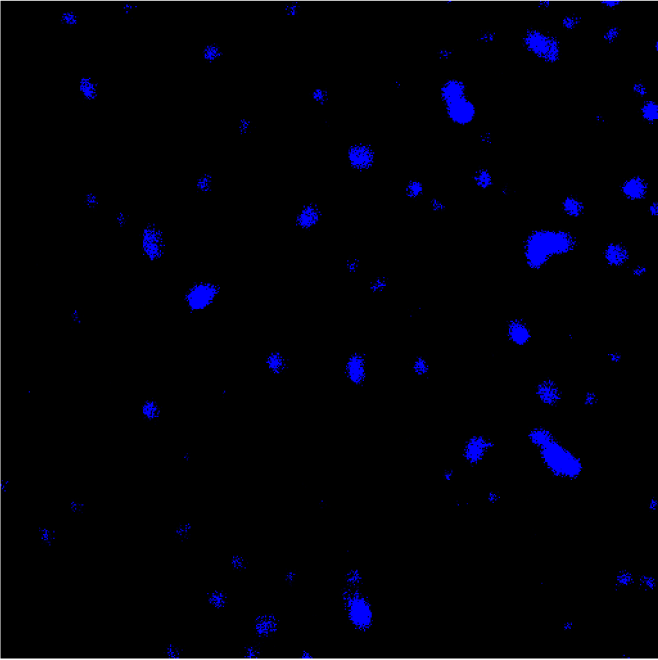

Supplement: Supplementary file 22 — Source data Fig. 4 [file 44318_2024_212_MOESM22_ESM.zip › Source Data For Figure4/4F/10 irtks 0 hp1a 500x4c1.tif]

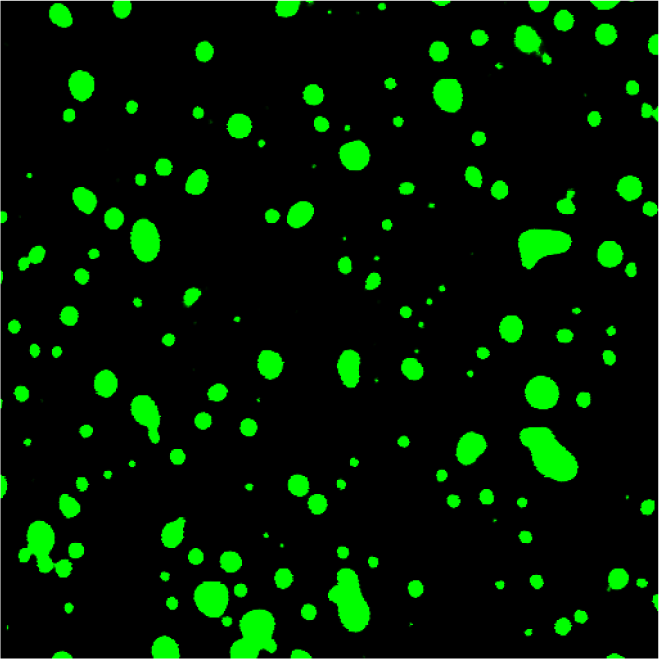

Supplement: Supplementary file 22 — Source data Fig. 4 [file 44318_2024_212_MOESM22_ESM.zip › Source Data For Figure4/4F/10 irtks 0 hp1a 500x4c2.tif]

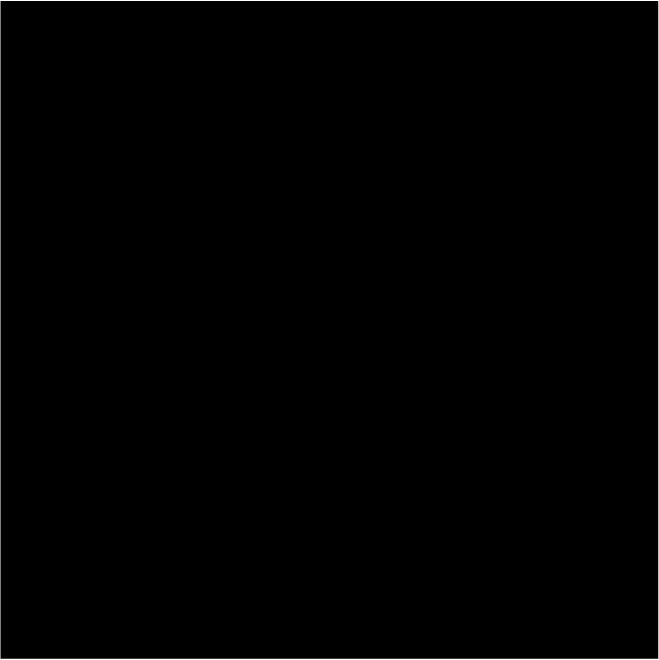

Supplement: Supplementary file 22 — Source data Fig. 4 [file 44318_2024_212_MOESM22_ESM.zip › Source Data For Figure4/4F/10 irtks 0 hp1a 500x4c3.tif]

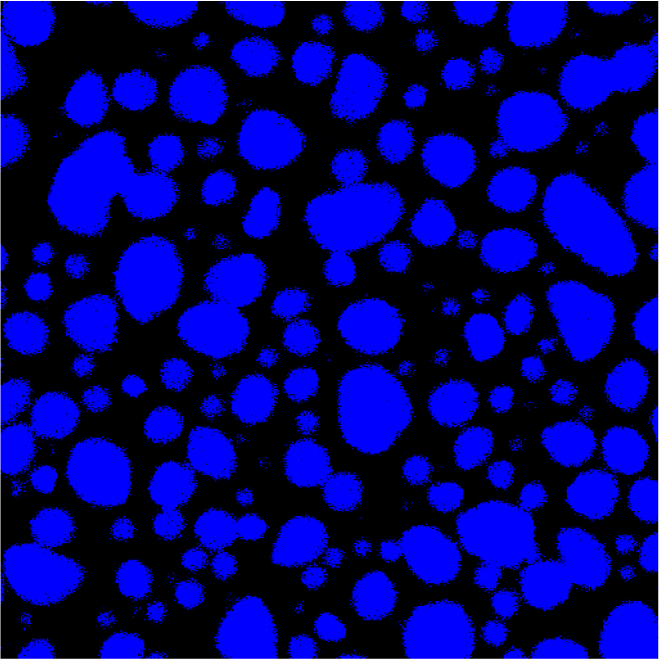

Supplement: Supplementary file 22 — Source data Fig. 4 [file 44318_2024_212_MOESM22_ESM.zip › Source Data For Figure4/4F/10 irtks 20 hp1a 500x4c1.tif]

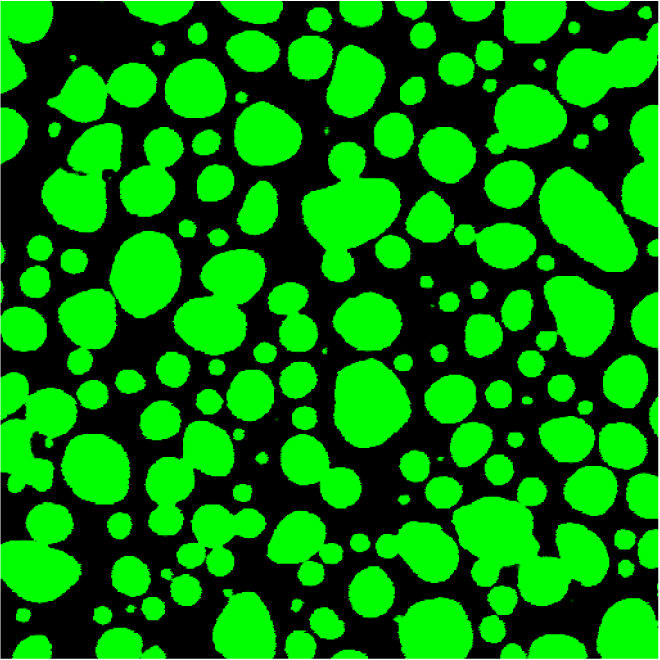

Supplement: Supplementary file 22 — Source data Fig. 4 [file 44318_2024_212_MOESM22_ESM.zip › Source Data For Figure4/4F/10 irtks 20 hp1a 500x4c2.tif]

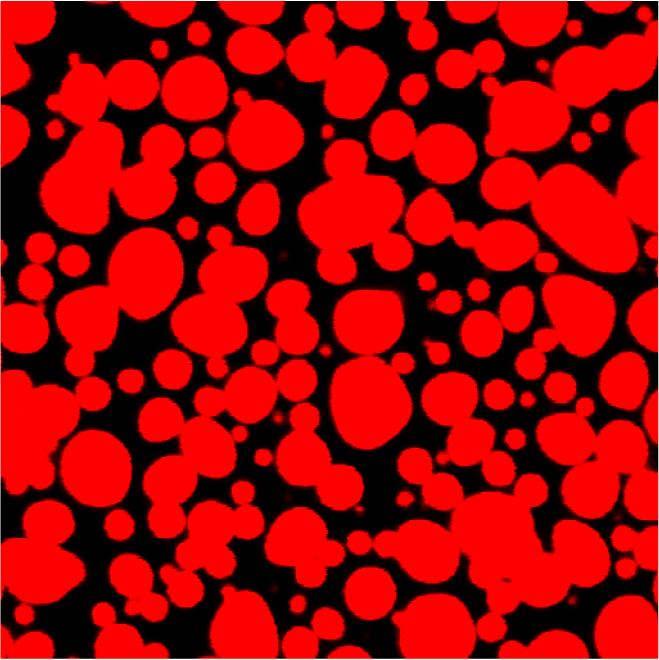

Supplement: Supplementary file 22 — Source data Fig. 4 [file 44318_2024_212_MOESM22_ESM.zip › Source Data For Figure4/4F/10 irtks 20 hp1a 500x4c3.tif]

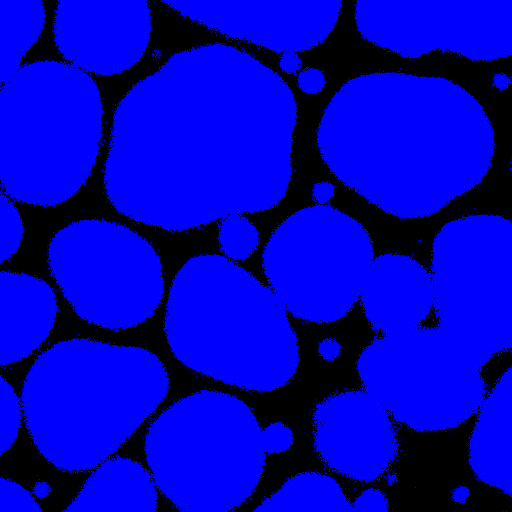

Supplement: Supplementary file 22 — Source data Fig. 4 [file 44318_2024_212_MOESM22_ESM.zip › Source Data For Figure4/4F/10 irtks 40 hp1a 500x4c1.tif]

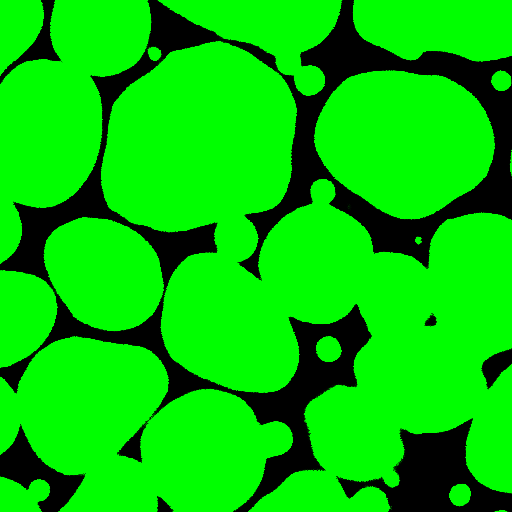

Supplement: Supplementary file 22 — Source data Fig. 4 [file 44318_2024_212_MOESM22_ESM.zip › Source Data For Figure4/4F/10 irtks 40 hp1a 500x4c2.tif]

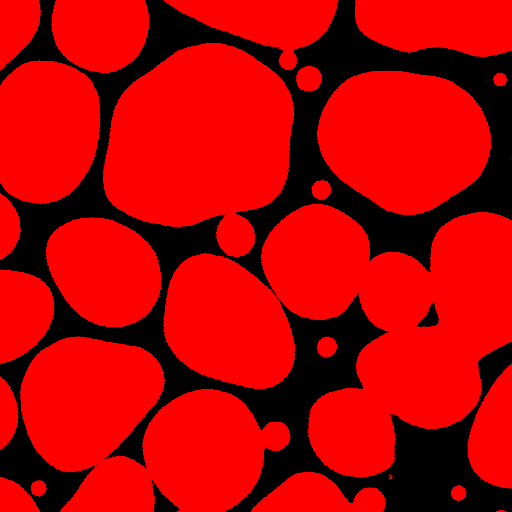

Supplement: Supplementary file 22 — Source data Fig. 4 [file 44318_2024_212_MOESM22_ESM.zip › Source Data For Figure4/4F/10 irtks 40 hp1a 500x4c3.tif]

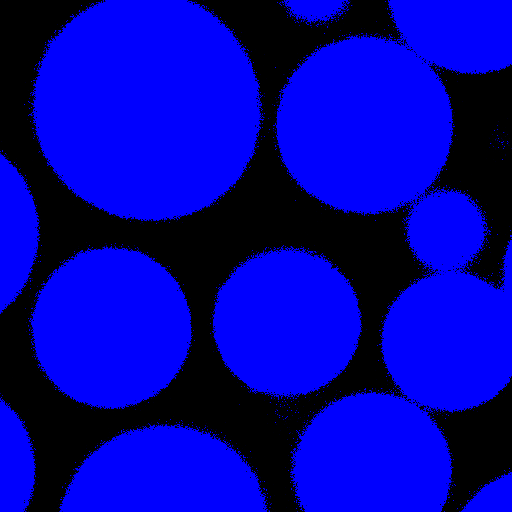

Supplement: Supplementary file 22 — Source data Fig. 4 [file 44318_2024_212_MOESM22_ESM.zip › Source Data For Figure4/4F/10 irtks 80 hp1a 500x 3c1.tif]

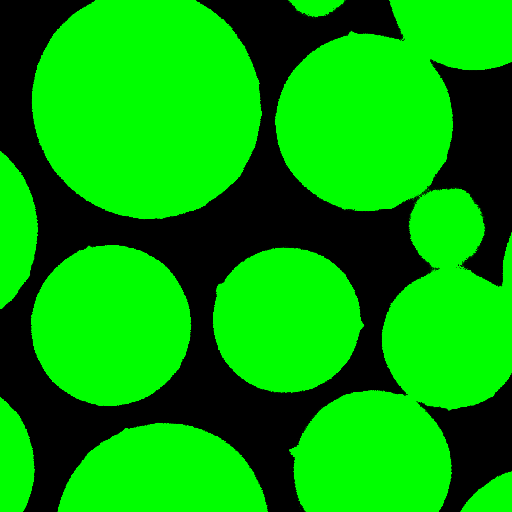

Supplement: Supplementary file 22 — Source data Fig. 4 [file 44318_2024_212_MOESM22_ESM.zip › Source Data For Figure4/4F/10 irtks 80 hp1a 500x 3c2.tif]

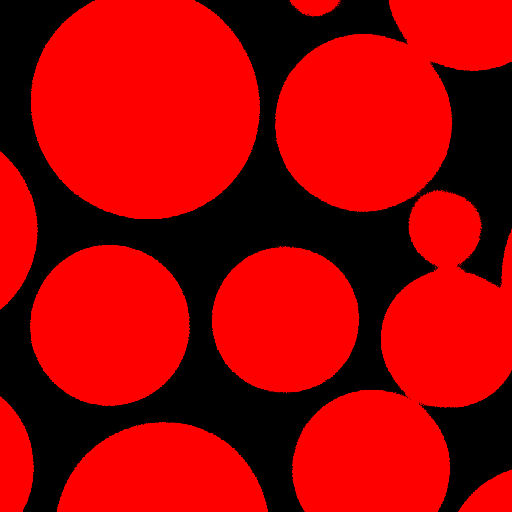

Supplement: Supplementary file 22 — Source data Fig. 4 [file 44318_2024_212_MOESM22_ESM.zip › Source Data For Figure4/4F/10 irtks 80 hp1a 500x 3c3.tif]

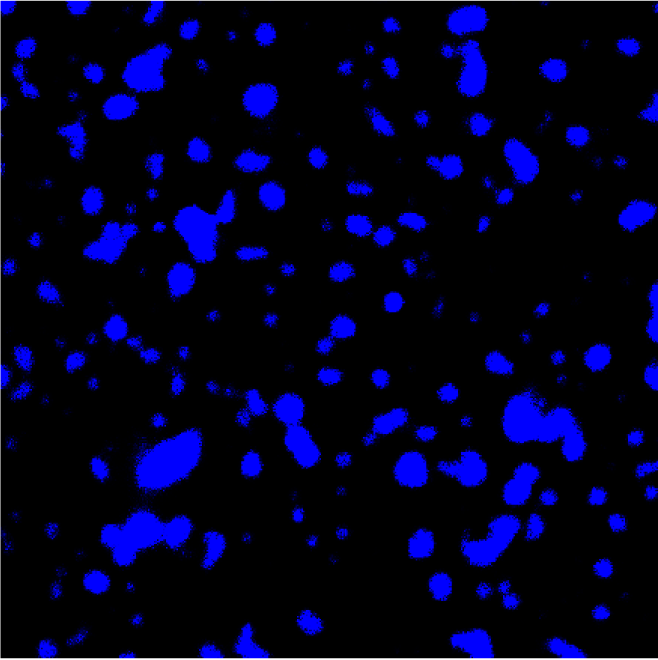

Supplement: Supplementary file 22 — Source data Fig. 4 [file 44318_2024_212_MOESM22_ESM.zip › Source Data For Figure4/4F/20 irtks 0 hp1a 500c1.tif]

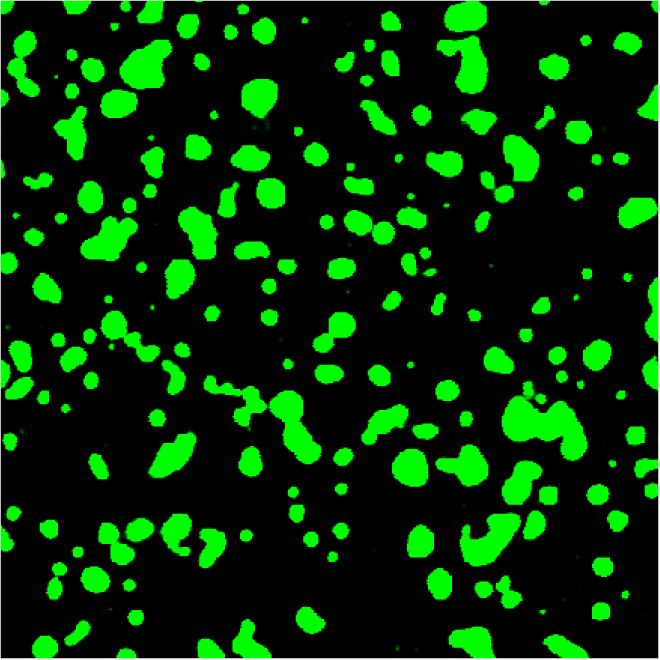

Supplement: Supplementary file 22 — Source data Fig. 4 [file 44318_2024_212_MOESM22_ESM.zip › Source Data For Figure4/4F/20 irtks 0 hp1a 500c2.tif]

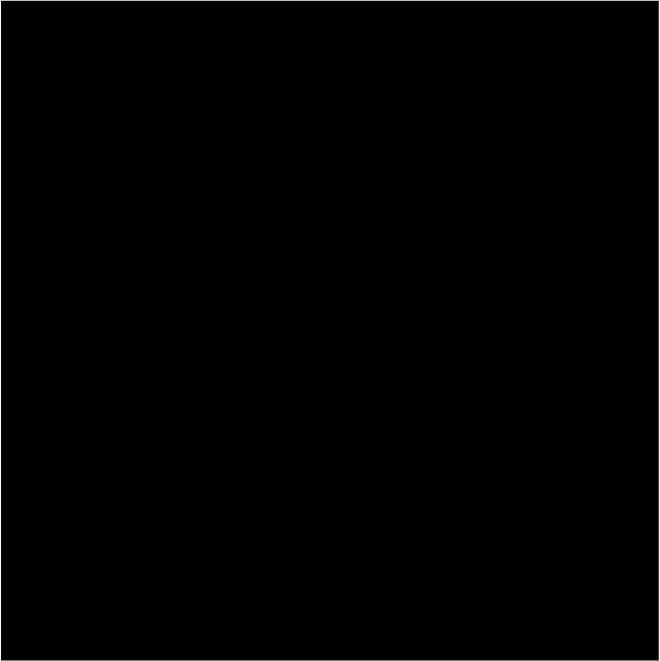

Supplement: Supplementary file 22 — Source data Fig. 4 [file 44318_2024_212_MOESM22_ESM.zip › Source Data For Figure4/4F/20 irtks 0 hp1a 500c3.tif]

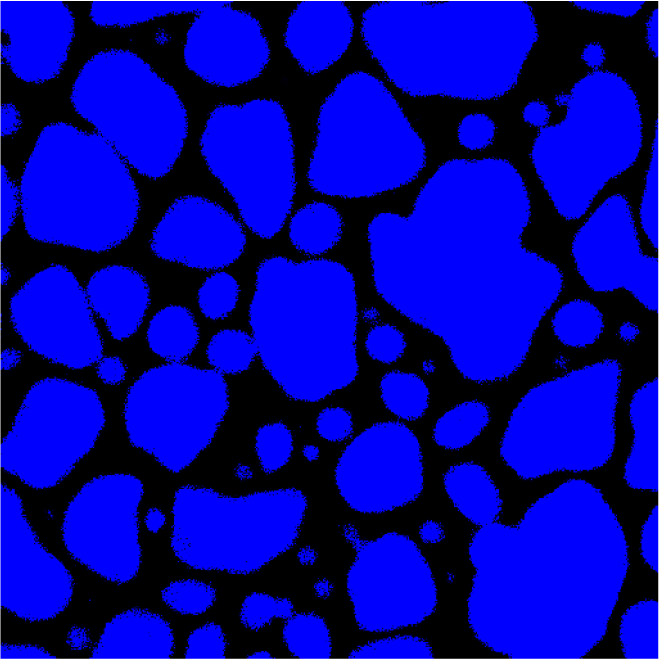

Supplement: Supplementary file 22 — Source data Fig. 4 [file 44318_2024_212_MOESM22_ESM.zip › Source Data For Figure4/4F/20 irtks 20 hp1a 500x6c1.tif]

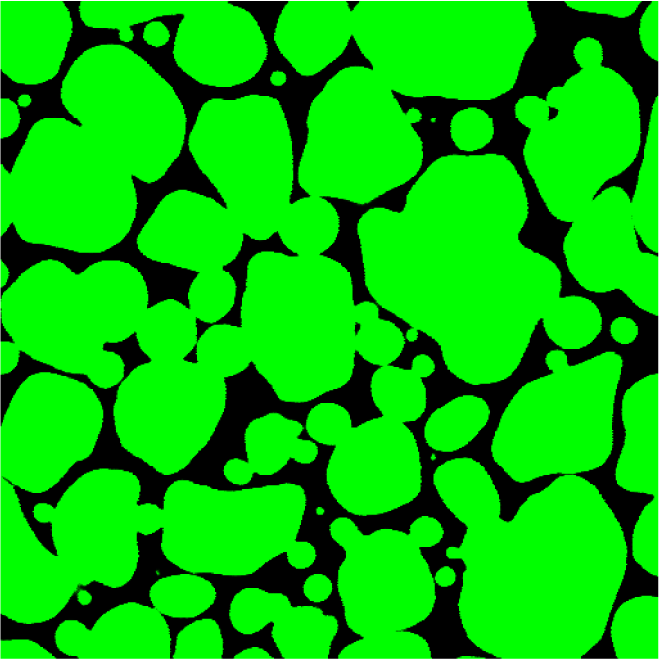

Supplement: Supplementary file 22 — Source data Fig. 4 [file 44318_2024_212_MOESM22_ESM.zip › Source Data For Figure4/4F/20 irtks 20 hp1a 500x6c2.tif]

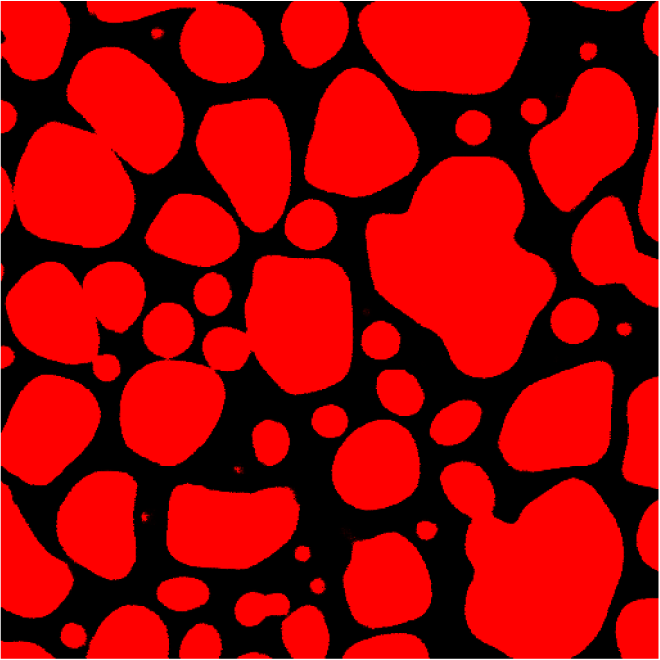

Supplement: Supplementary file 22 — Source data Fig. 4 [file 44318_2024_212_MOESM22_ESM.zip › Source Data For Figure4/4F/20 irtks 20 hp1a 500x6c3.tif]

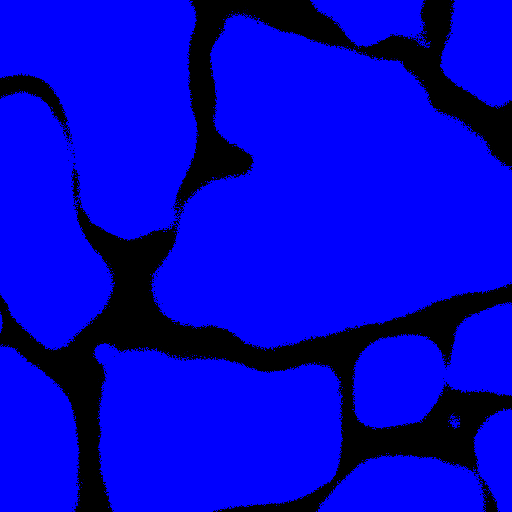

Supplement: Supplementary file 22 — Source data Fig. 4 [file 44318_2024_212_MOESM22_ESM.zip › Source Data For Figure4/4F/20 irtks 40 hp1a 500c1.tif]

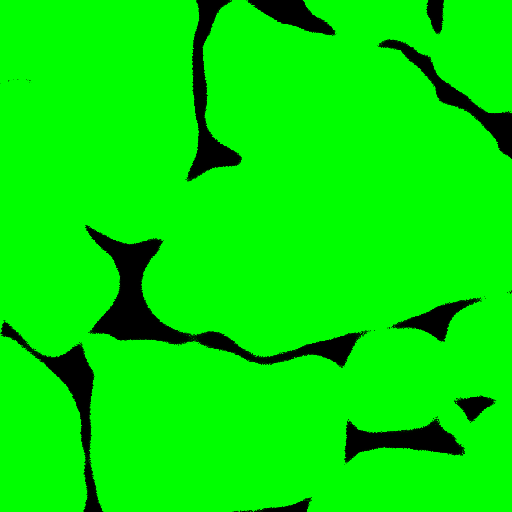

Supplement: Supplementary file 22 — Source data Fig. 4 [file 44318_2024_212_MOESM22_ESM.zip › Source Data For Figure4/4F/20 irtks 40 hp1a 500c2.tif]

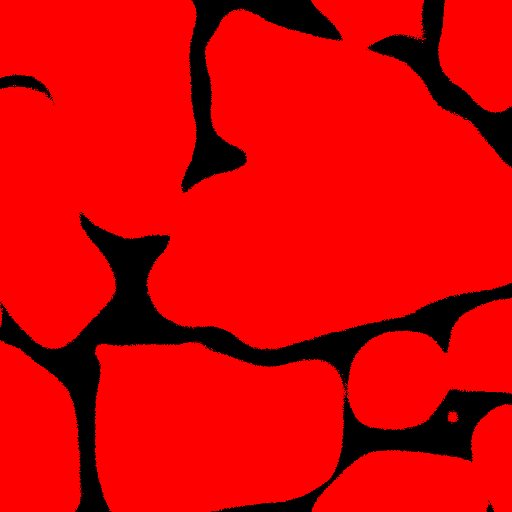

Supplement: Supplementary file 22 — Source data Fig. 4 [file 44318_2024_212_MOESM22_ESM.zip › Source Data For Figure4/4F/20 irtks 40 hp1a 500c3.tif]

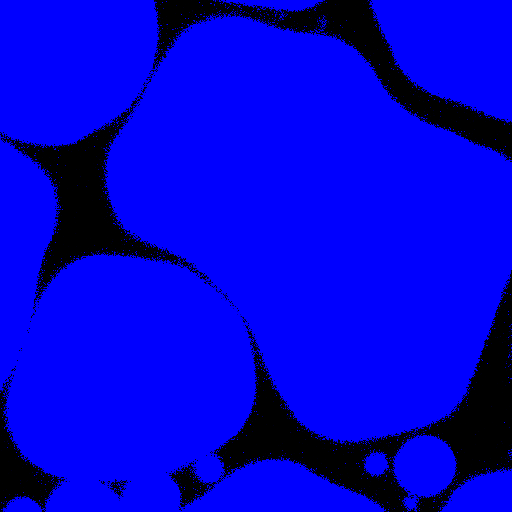

Supplement: Supplementary file 22 — Source data Fig. 4 [file 44318_2024_212_MOESM22_ESM.zip › Source Data For Figure4/4F/20 irtks 80 hp1a 500c1.tif]

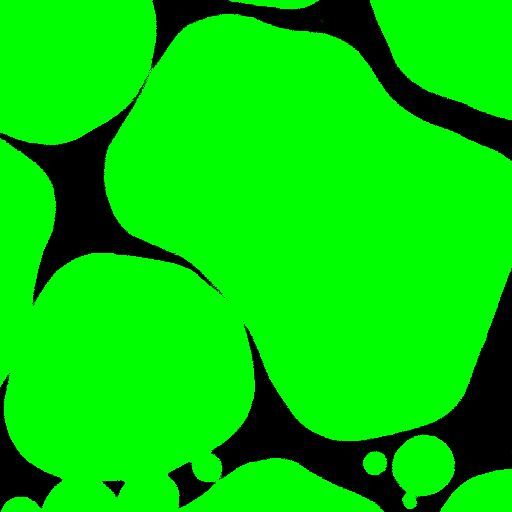

Supplement: Supplementary file 22 — Source data Fig. 4 [file 44318_2024_212_MOESM22_ESM.zip › Source Data For Figure4/4F/20 irtks 80 hp1a 500c2.tif]

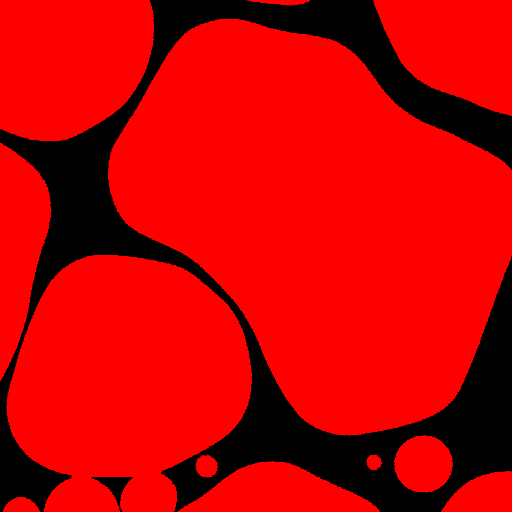

Supplement: Supplementary file 22 — Source data Fig. 4 [file 44318_2024_212_MOESM22_ESM.zip › Source Data For Figure4/4F/20 irtks 80 hp1a 500c3.tif]

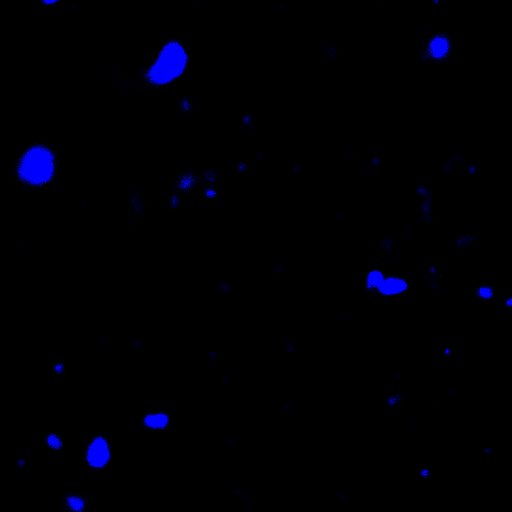

Supplement: Supplementary file 22 — Source data Fig. 4 [file 44318_2024_212_MOESM22_ESM.zip › Source Data For Figure4/4F/5 irtks 0 hp1a 500x 4c1.tif]

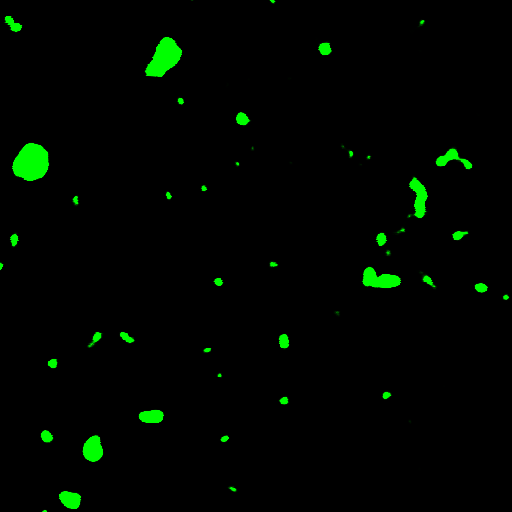

Supplement: Supplementary file 22 — Source data Fig. 4 [file 44318_2024_212_MOESM22_ESM.zip › Source Data For Figure4/4F/5 irtks 0 hp1a 500x 4c2.tif]

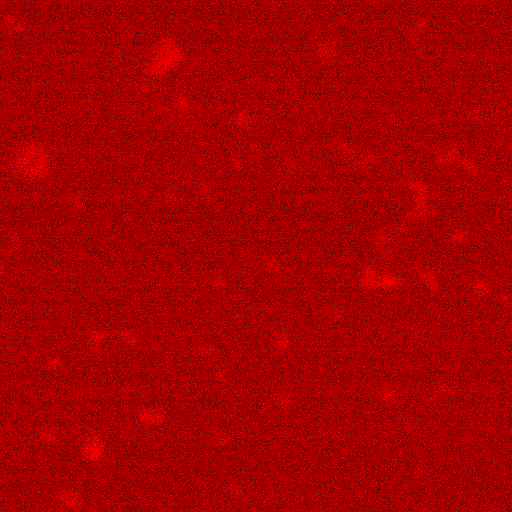

Supplement: Supplementary file 22 — Source data Fig. 4 [file 44318_2024_212_MOESM22_ESM.zip › Source Data For Figure4/4F/5 irtks 0 hp1a 500x 4c3.tif]

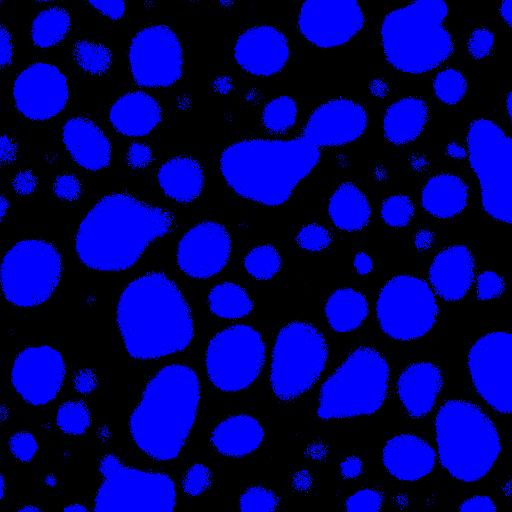

Supplement: Supplementary file 22 — Source data Fig. 4 [file 44318_2024_212_MOESM22_ESM.zip › Source Data For Figure4/4F/5 irtks 20 hp1a 500x6c1.tif]

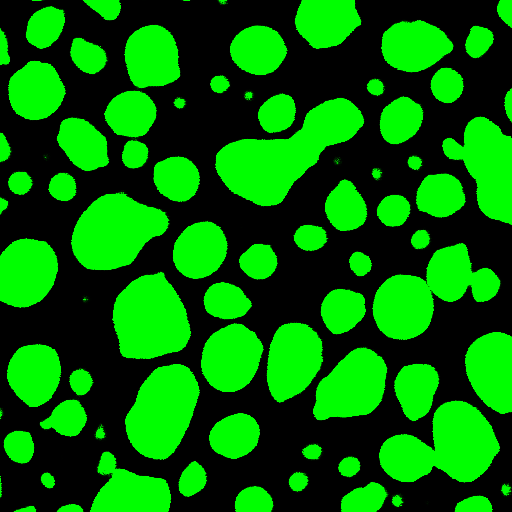

Supplement: Supplementary file 22 — Source data Fig. 4 [file 44318_2024_212_MOESM22_ESM.zip › Source Data For Figure4/4F/5 irtks 20 hp1a 500x6c2.tif]

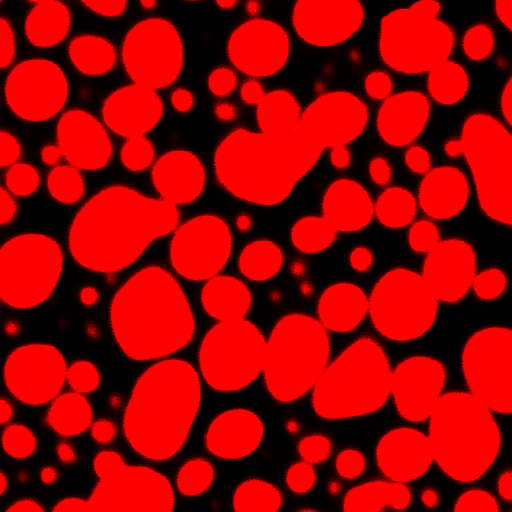

Supplement: Supplementary file 22 — Source data Fig. 4 [file 44318_2024_212_MOESM22_ESM.zip › Source Data For Figure4/4F/5 irtks 20 hp1a 500x6c3.tif]

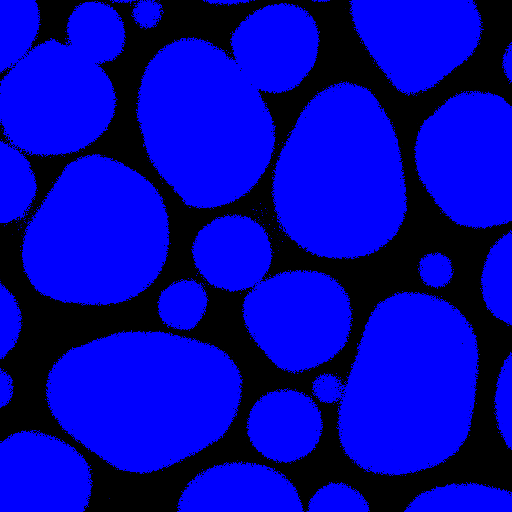

Supplement: Supplementary file 22 — Source data Fig. 4 [file 44318_2024_212_MOESM22_ESM.zip › Source Data For Figure4/4F/5 irtks 40 hp1a 500x2c1.tif]

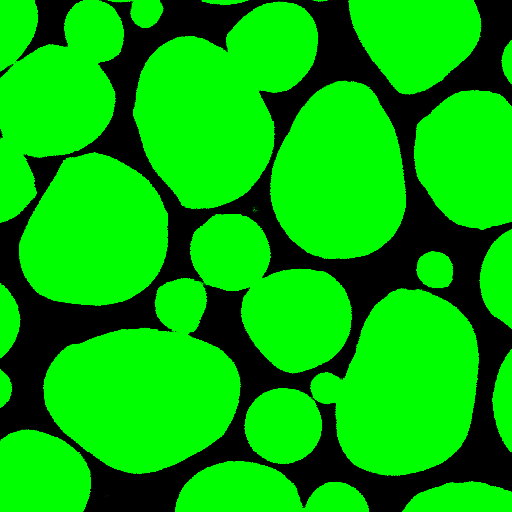

Supplement: Supplementary file 22 — Source data Fig. 4 [file 44318_2024_212_MOESM22_ESM.zip › Source Data For Figure4/4F/5 irtks 40 hp1a 500x2c2.tif]

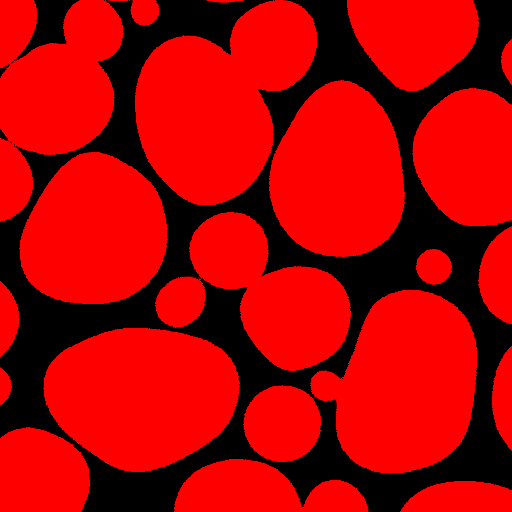

Supplement: Supplementary file 22 — Source data Fig. 4 [file 44318_2024_212_MOESM22_ESM.zip › Source Data For Figure4/4F/5 irtks 40 hp1a 500x2c3.tif]

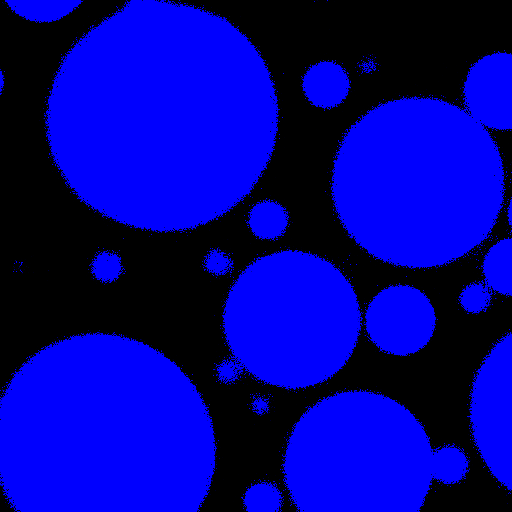

Supplement: Supplementary file 22 — Source data Fig. 4 [file 44318_2024_212_MOESM22_ESM.zip › Source Data For Figure4/4F/5 irtks 80 hp1a 500x 2c1.tif]

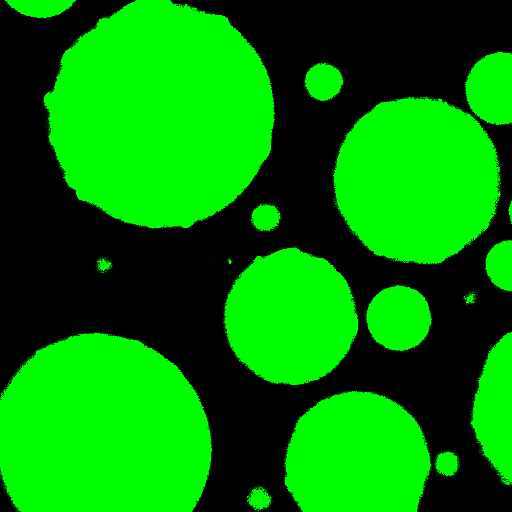

Supplement: Supplementary file 22 — Source data Fig. 4 [file 44318_2024_212_MOESM22_ESM.zip › Source Data For Figure4/4F/5 irtks 80 hp1a 500x 2c2.tif]

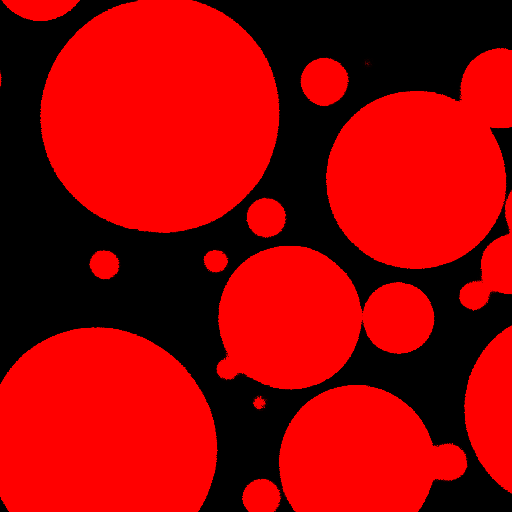

Supplement: Supplementary file 22 — Source data Fig. 4 [file 44318_2024_212_MOESM22_ESM.zip › Source Data For Figure4/4F/5 irtks 80 hp1a 500x 2c3.tif]

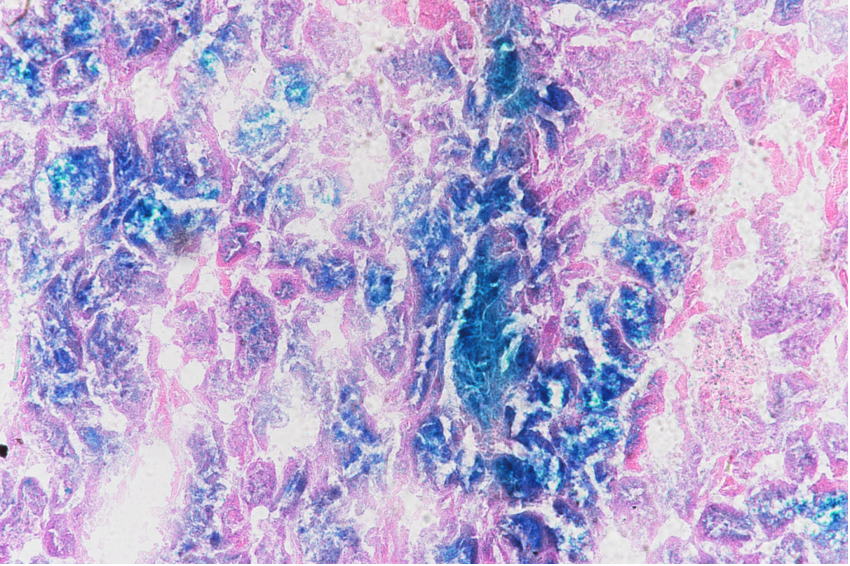

Supplement: Supplementary file 24 — Source data Fig. 6 [file 44318_2024_212_MOESM24_ESM.zip › Source Data For Figure6/6C/12M-KO-enlarged.tif]

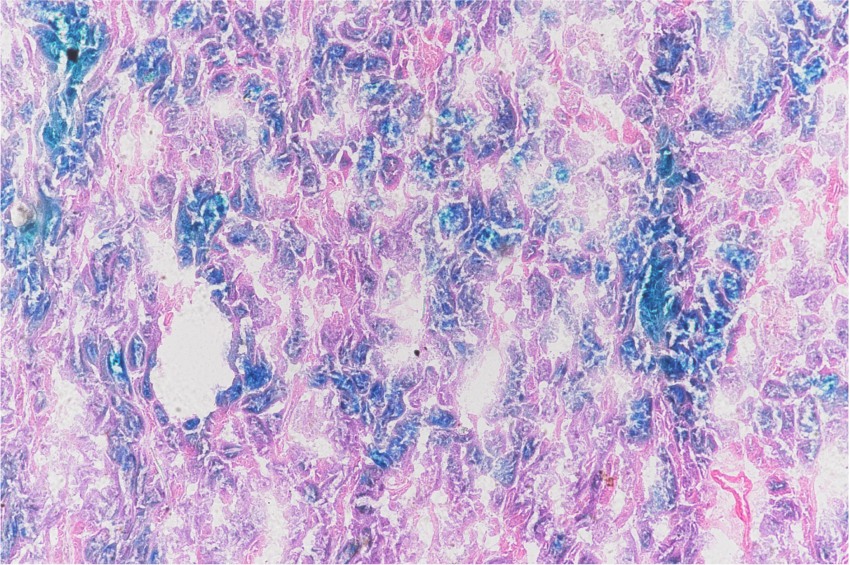

Supplement: Supplementary file 24 — Source data Fig. 6 [file 44318_2024_212_MOESM24_ESM.zip › Source Data For Figure6/6C/12M-KO.tif]

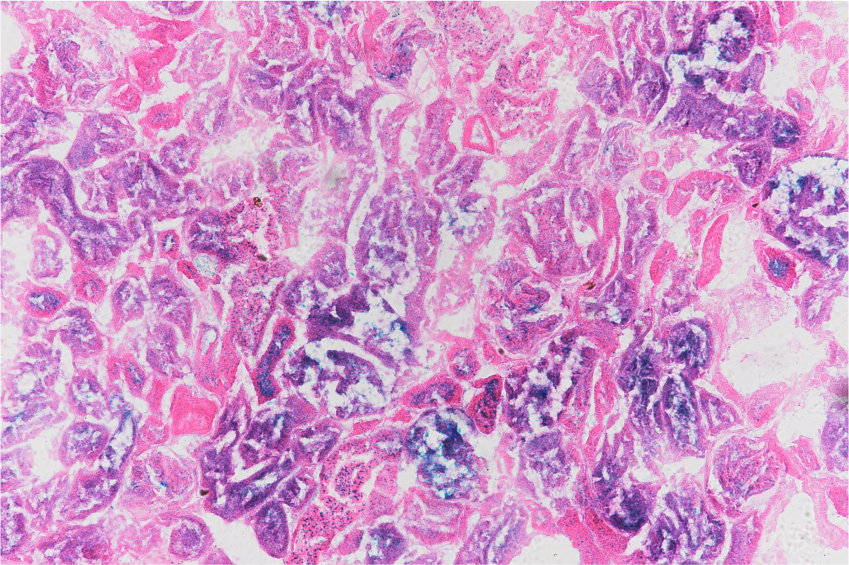

Supplement: Supplementary file 24 — Source data Fig. 6 [file 44318_2024_212_MOESM24_ESM.zip › Source Data For Figure6/6C/12M-WT-enlarged.tif]

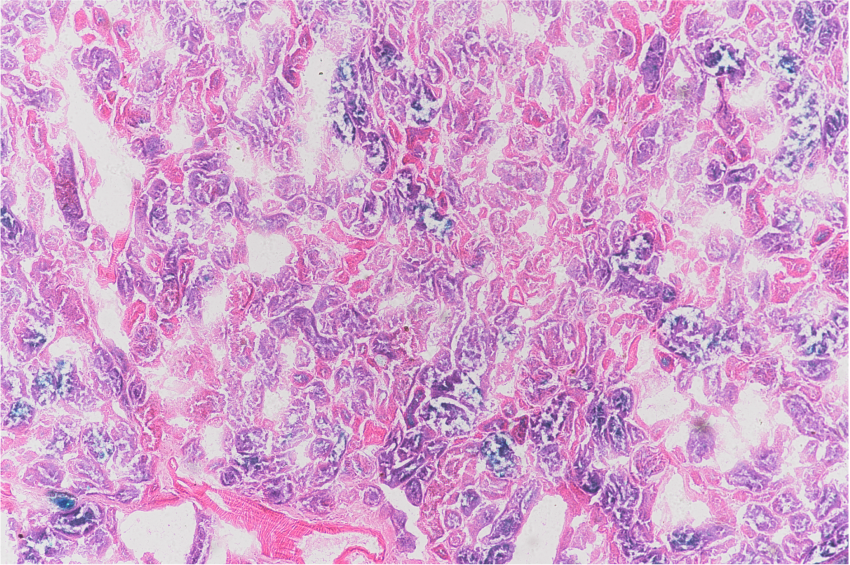

Supplement: Supplementary file 24 — Source data Fig. 6 [file 44318_2024_212_MOESM24_ESM.zip › Source Data For Figure6/6C/12M-WT.tif]

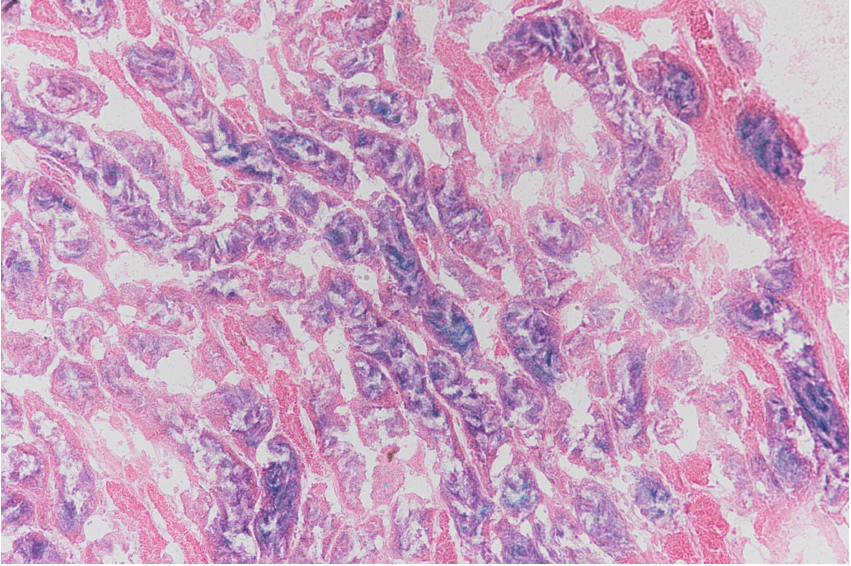

Supplement: Supplementary file 24 — Source data Fig. 6 [file 44318_2024_212_MOESM24_ESM.zip › Source Data For Figure6/6C/6M-KO-enlarged.tif]

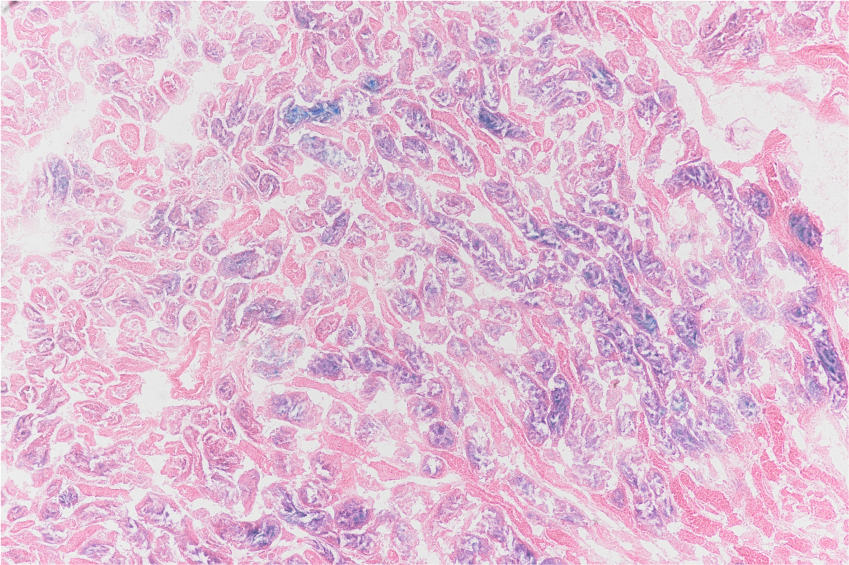

Supplement: Supplementary file 24 — Source data Fig. 6 [file 44318_2024_212_MOESM24_ESM.zip › Source Data For Figure6/6C/6M-KO.tif]

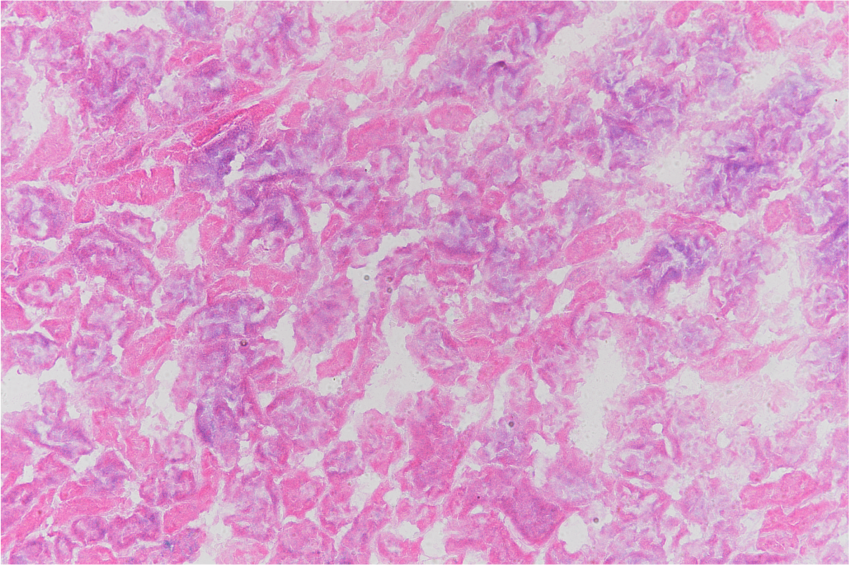

Supplement: Supplementary file 24 — Source data Fig. 6 [file 44318_2024_212_MOESM24_ESM.zip › Source Data For Figure6/6C/6M-WT-enlarged.tif]

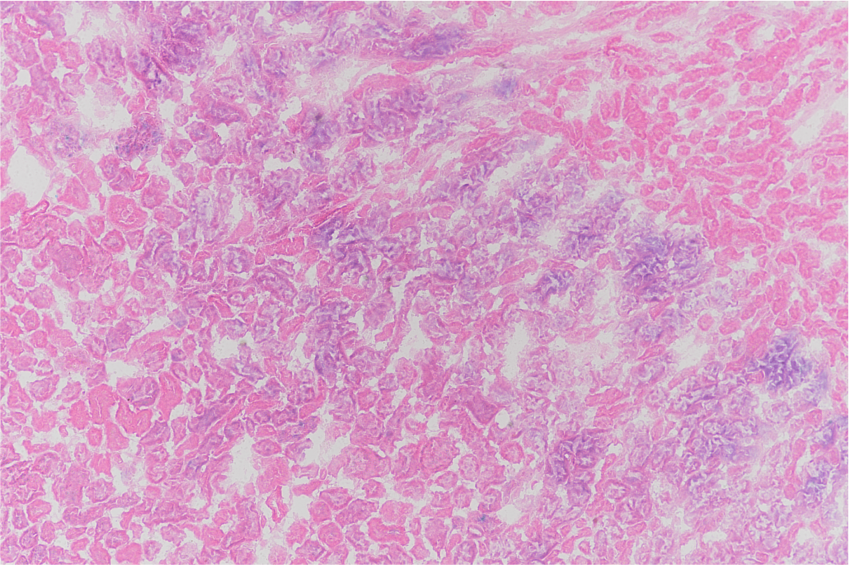

Supplement: Supplementary file 24 — Source data Fig. 6 [file 44318_2024_212_MOESM24_ESM.zip › Source Data For Figure6/6C/6M-WT.tif]

**Fig. 6B**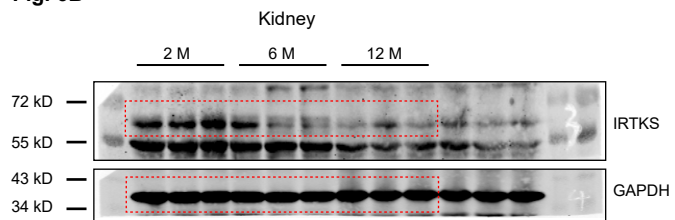**Fig. 6F**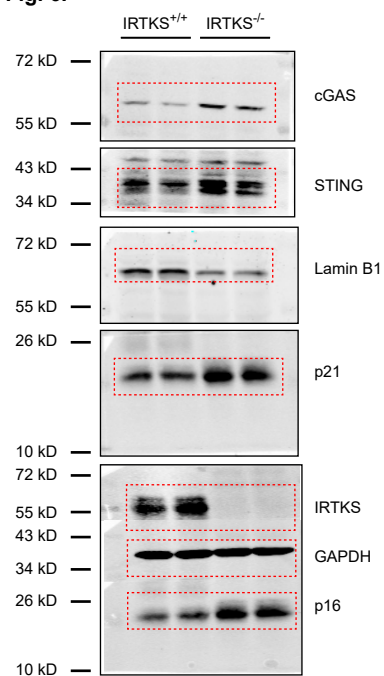

Supplement: Supplementary file 24 — Source data Fig. 6 [file 44318_2024_212_MOESM24_ESM.zip › Source Data For Figure6/Source Data Fig. 6.pdf]

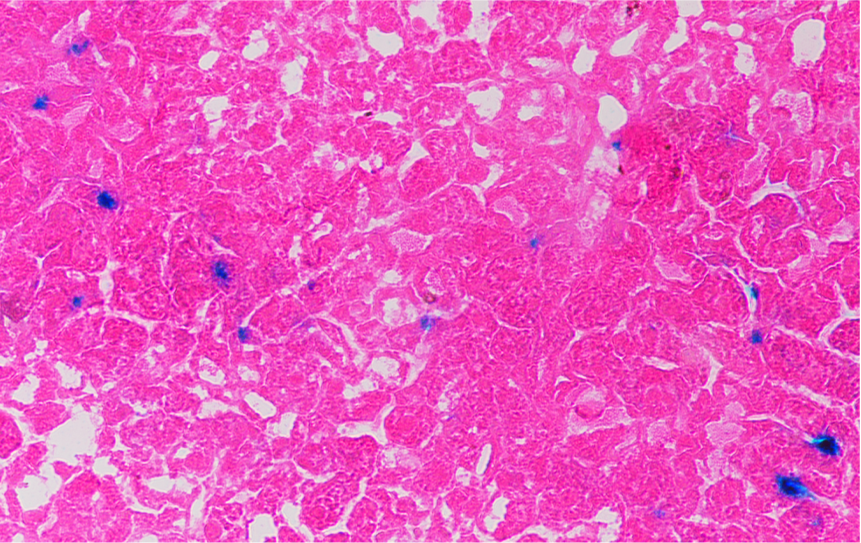

Supplement: Supplementary file 25 — Figure Source Data for Expanded View and Appendix [file 44318_2024_212_MOESM25_ESM.zip › Source Data for Expanded View and Appendix/Appendix Fig S2/2C/KO-enlarged.tif]

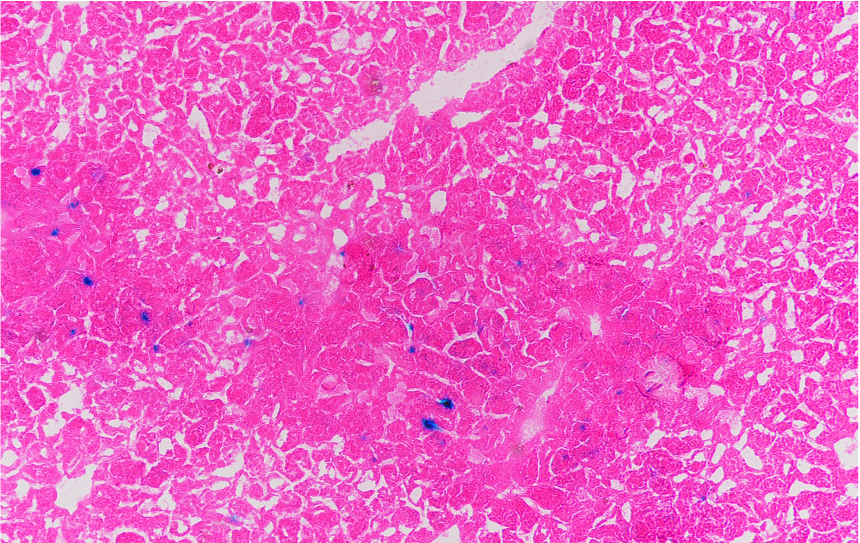

Supplement: Supplementary file 25 — Figure Source Data for Expanded View and Appendix [file 44318_2024_212_MOESM25_ESM.zip › Source Data for Expanded View and Appendix/Appendix Fig S2/2C/KO.tif]

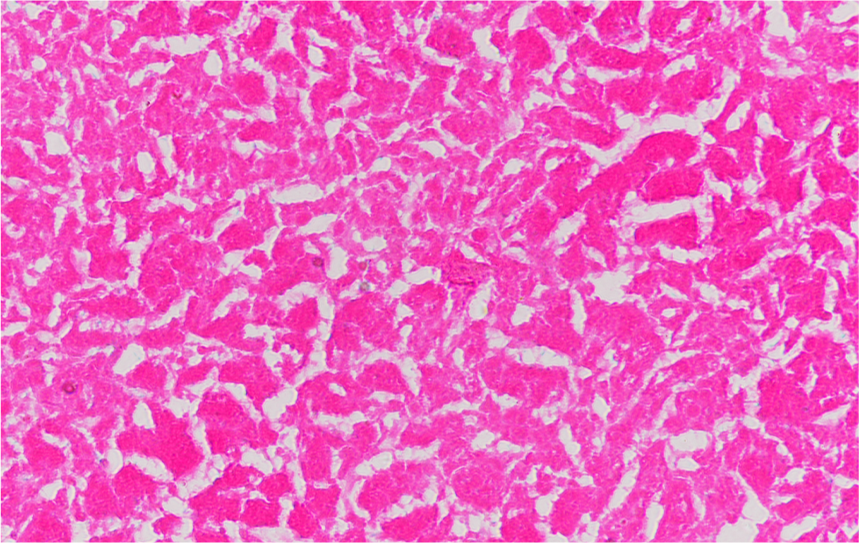

Supplement: Supplementary file 25 — Figure Source Data for Expanded View and Appendix [file 44318_2024_212_MOESM25_ESM.zip › Source Data for Expanded View and Appendix/Appendix Fig S2/2C/WT-enlarged.tif]

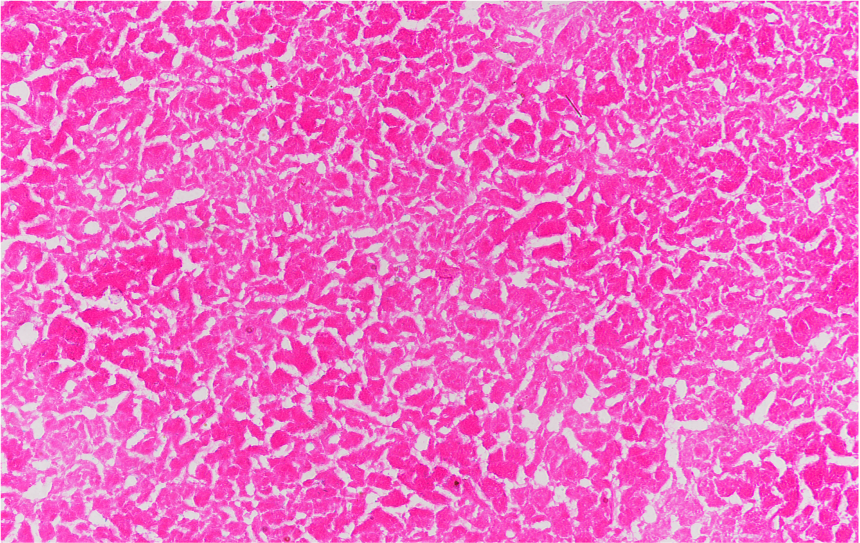

Supplement: Supplementary file 25 — Figure Source Data for Expanded View and Appendix [file 44318_2024_212_MOESM25_ESM.zip › Source Data for Expanded View and Appendix/Appendix Fig S2/2C/WT.tif]

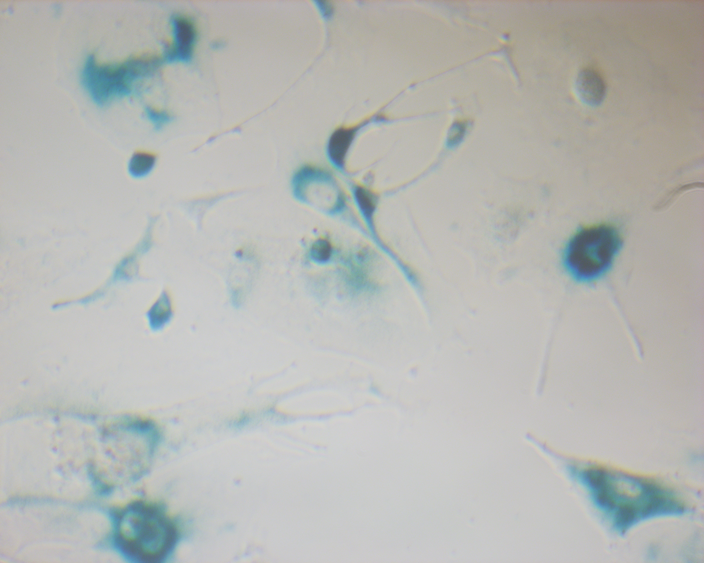

Supplement: Supplementary file 25 — Figure Source Data for Expanded View and Appendix [file 44318_2024_212_MOESM25_ESM.zip › Source Data for Expanded View and Appendix/Appendix Fig S2/2D/KO.tif]

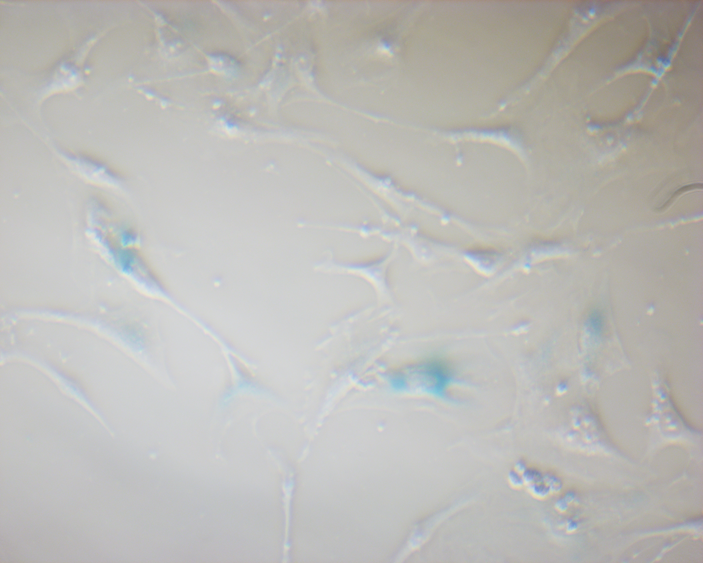

Supplement: Supplementary file 25 — Figure Source Data for Expanded View and Appendix [file 44318_2024_212_MOESM25_ESM.zip › Source Data for Expanded View and Appendix/Appendix Fig S2/2D/WT.tif]

Appendix Fig. S2B

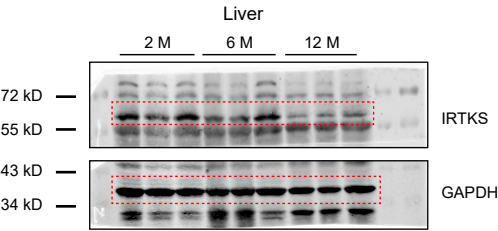

Appendix Fig. S2I

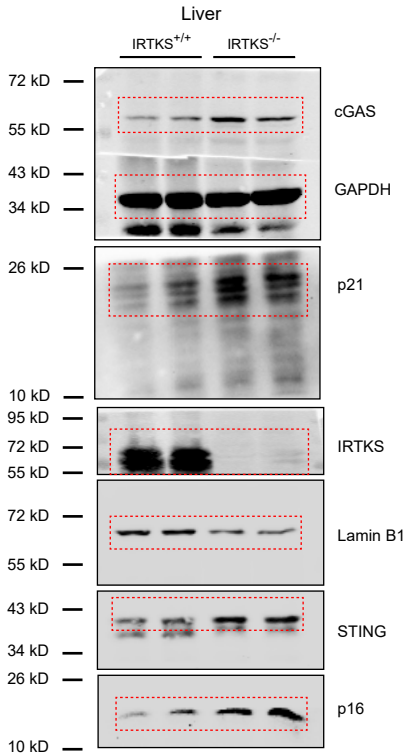

Supplement: Supplementary file 25 — Figure Source Data for Expanded View and Appendix [file 44318_2024_212_MOESM25_ESM.zip › Source Data for Expanded View and Appendix/Appendix Fig S2/Source Data Appendix Fig. S2.pdf]

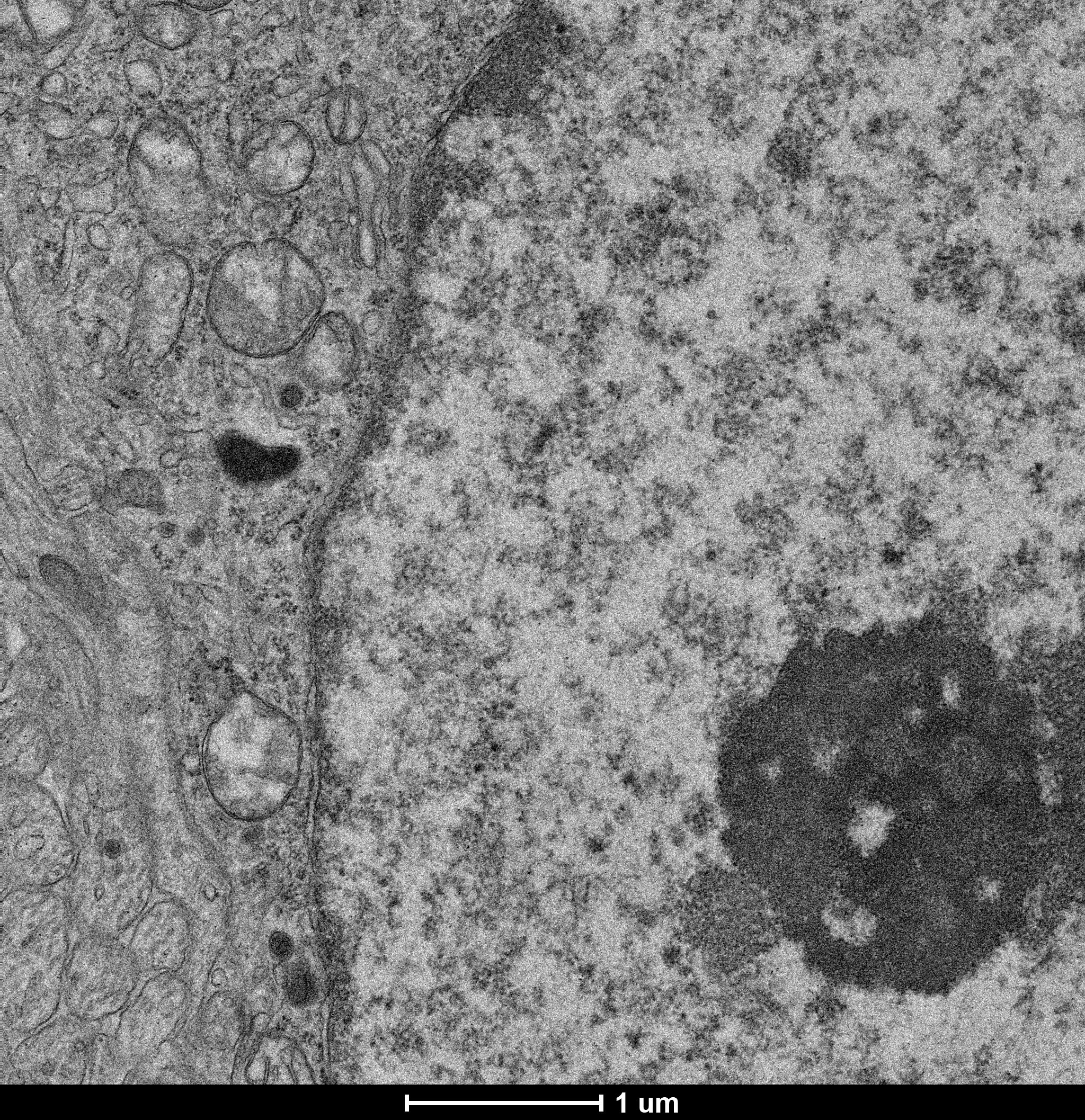

Supplement: Supplementary file 25 — Figure Source Data for Expanded View and Appendix [file 44318_2024_212_MOESM25_ESM.zip › Source Data for Expanded View and Appendix/Figure EV1/1C/KO-enlarged.jpg]

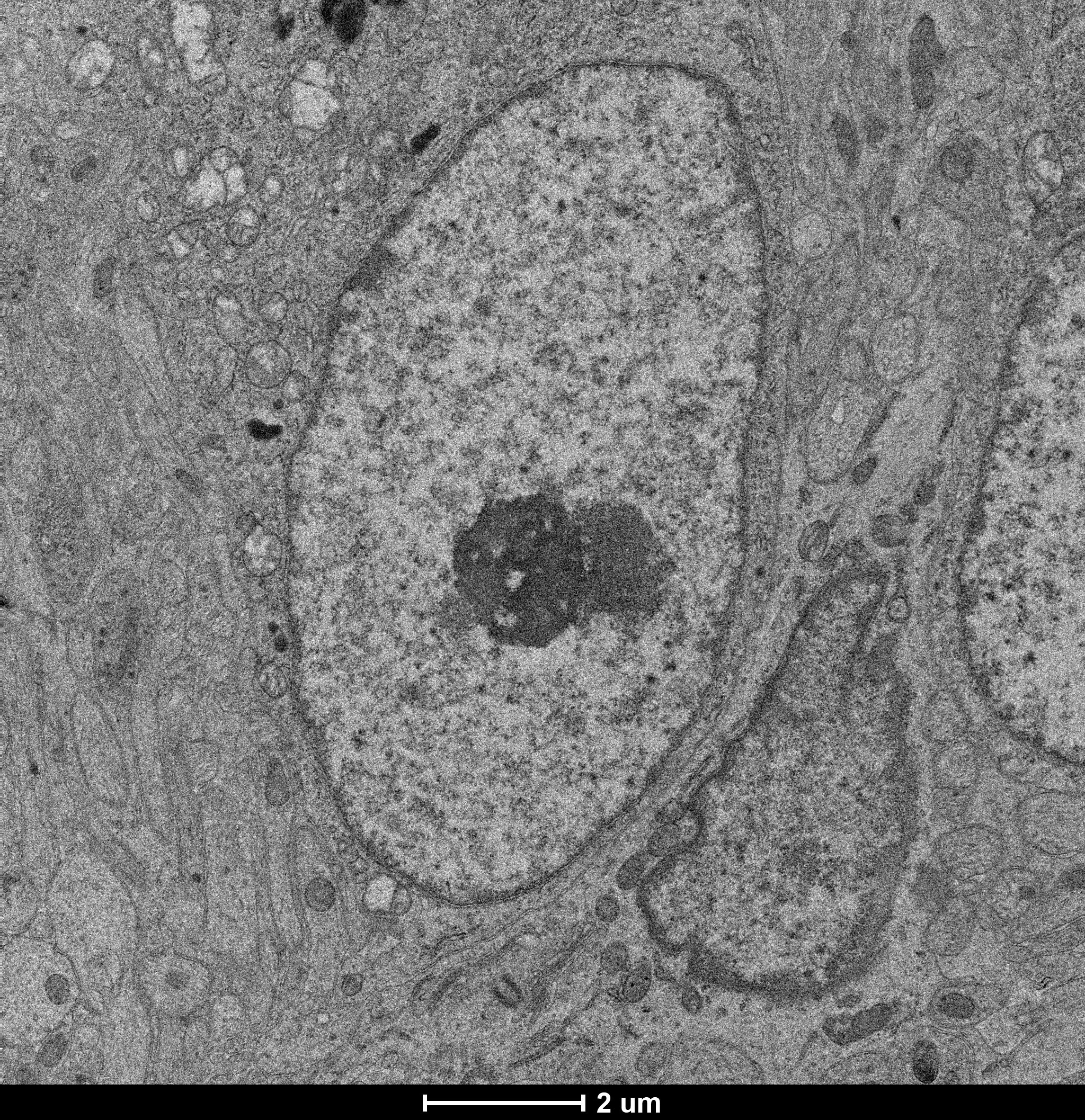

Supplement: Supplementary file 25 — Figure Source Data for Expanded View and Appendix [file 44318_2024_212_MOESM25_ESM.zip › Source Data for Expanded View and Appendix/Figure EV1/1C/KO.jpg]

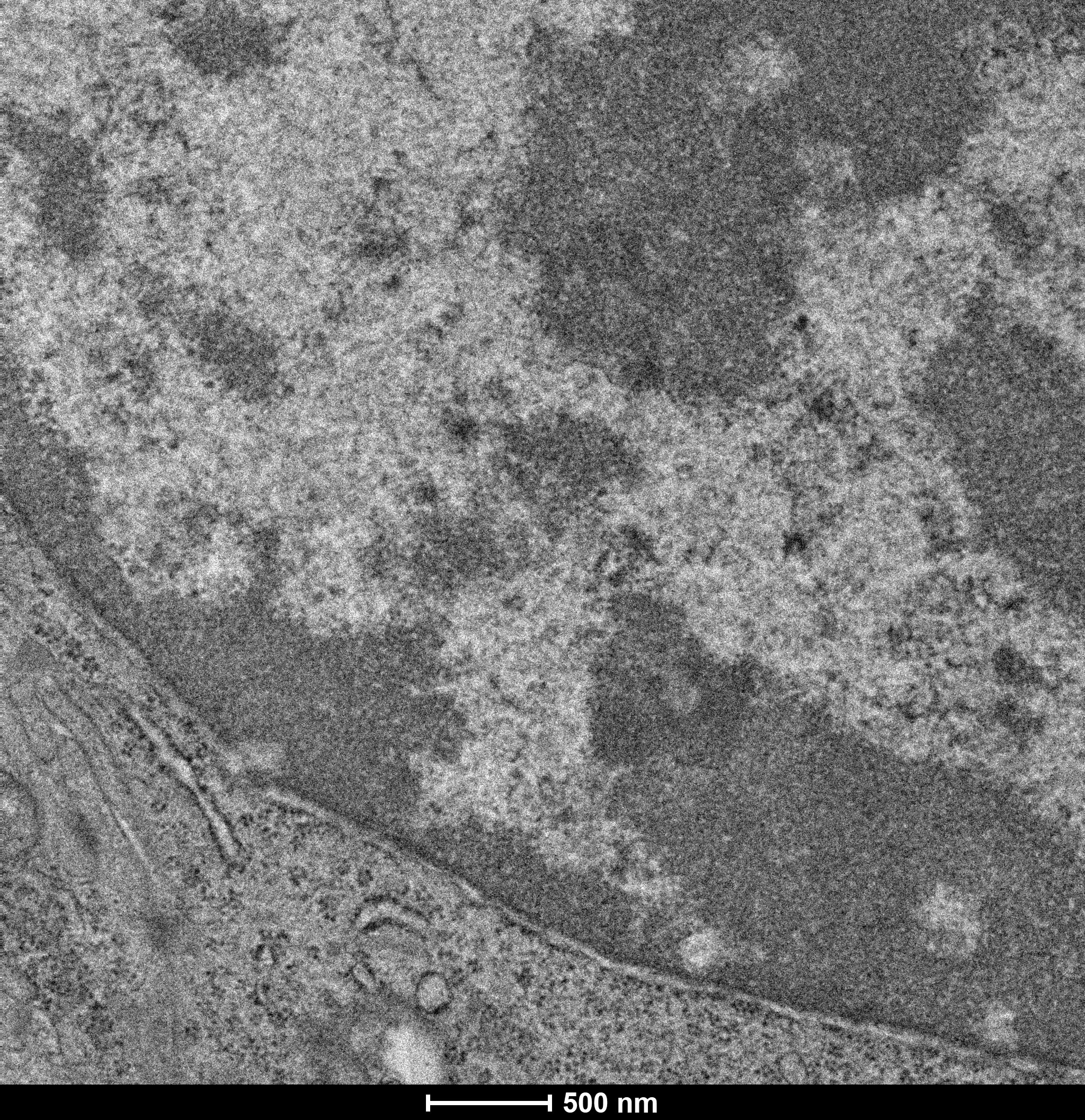

Supplement: Supplementary file 25 — Figure Source Data for Expanded View and Appendix [file 44318_2024_212_MOESM25_ESM.zip › Source Data for Expanded View and Appendix/Figure EV1/1C/WT-enlarged.jpg]

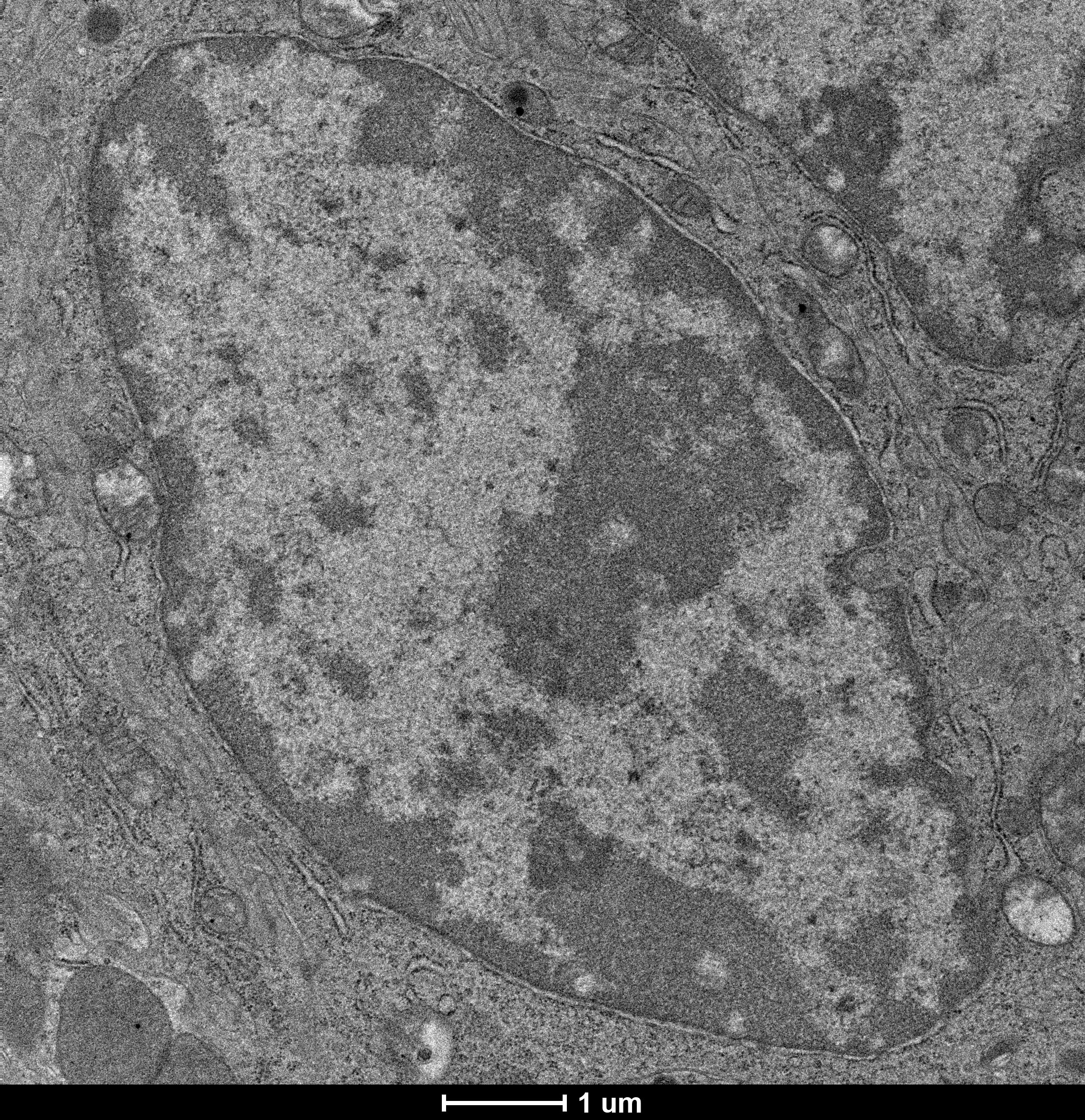

Supplement: Supplementary file 25 — Figure Source Data for Expanded View and Appendix [file 44318_2024_212_MOESM25_ESM.zip › Source Data for Expanded View and Appendix/Figure EV1/1C/WT.jpg]

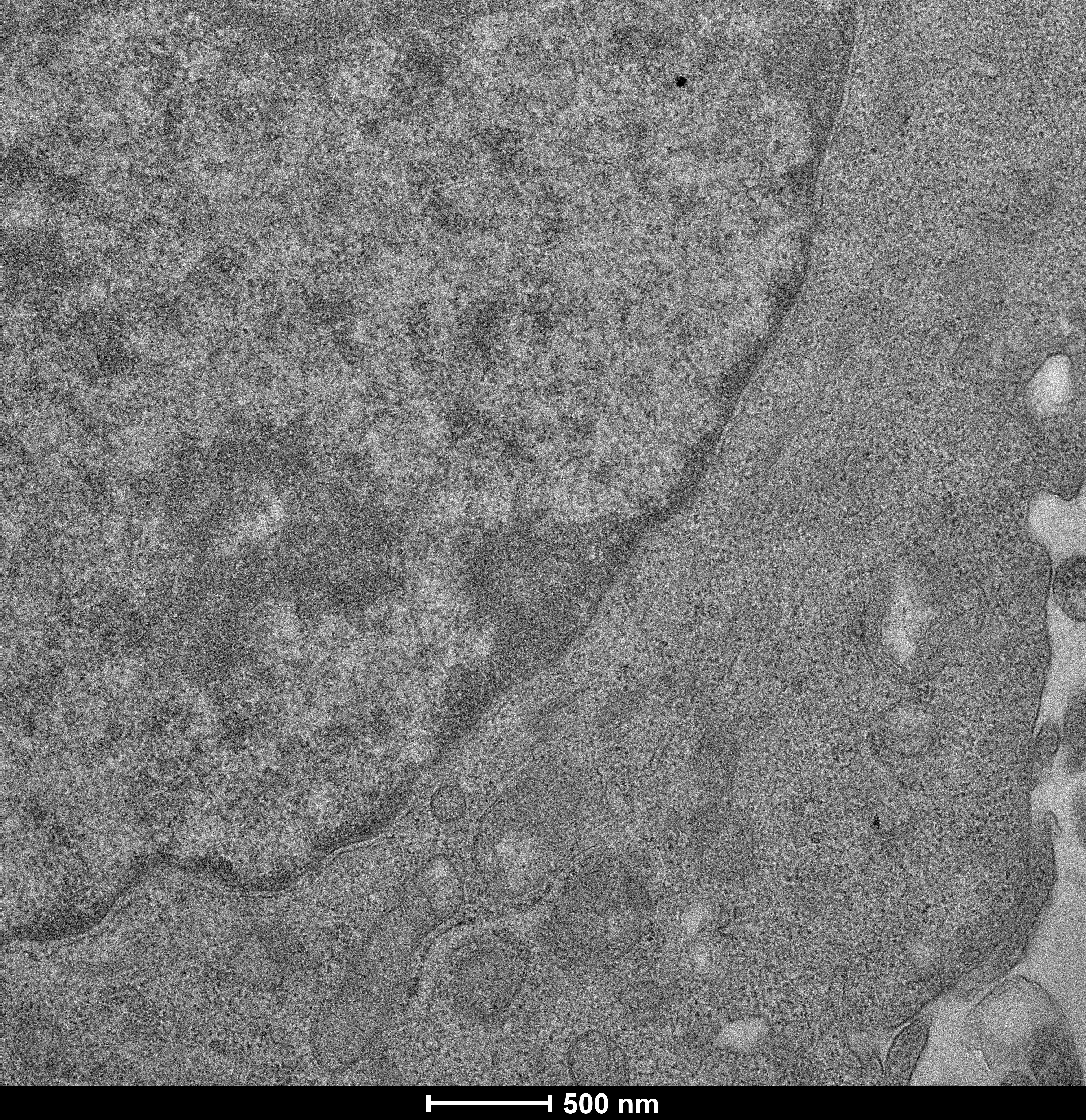

Supplement: Supplementary file 25 — Figure Source Data for Expanded View and Appendix [file 44318_2024_212_MOESM25_ESM.zip › Source Data for Expanded View and Appendix/Figure EV1/1G/sgControl-enlarged.jpg]

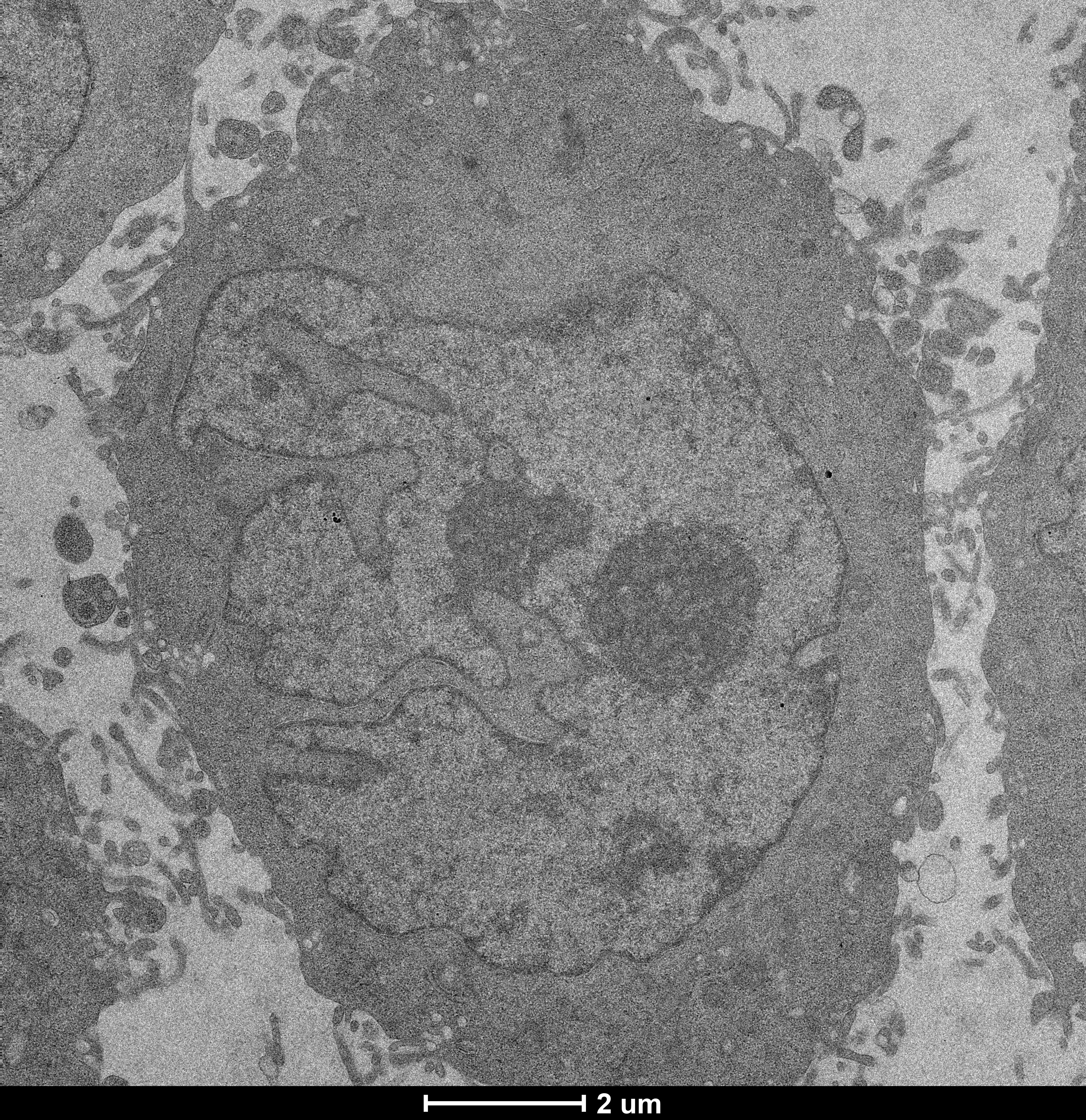

Supplement: Supplementary file 25 — Figure Source Data for Expanded View and Appendix [file 44318_2024_212_MOESM25_ESM.zip › Source Data for Expanded View and Appendix/Figure EV1/1G/sgControl.jpg]

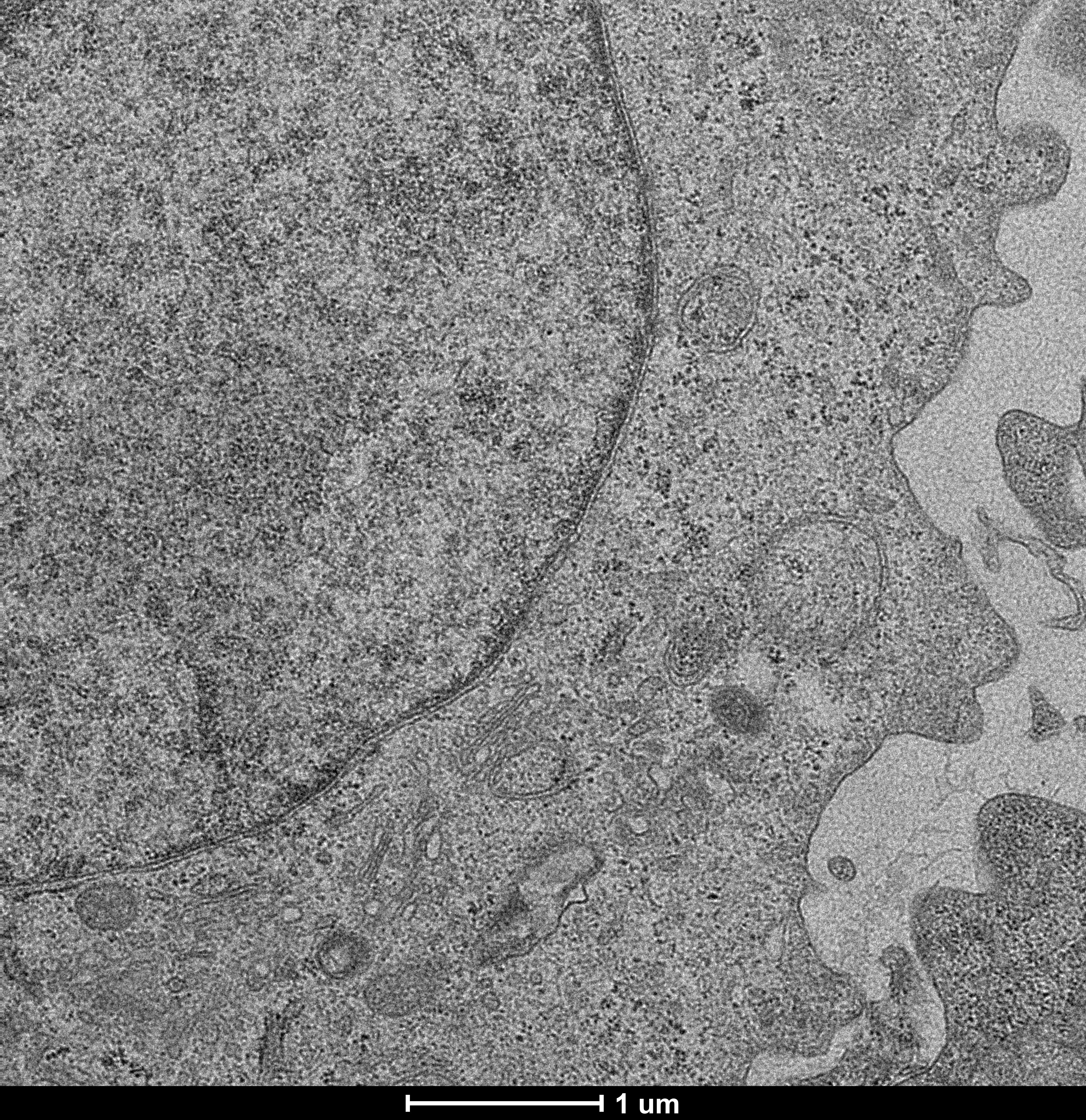

Supplement: Supplementary file 25 — Figure Source Data for Expanded View and Appendix [file 44318_2024_212_MOESM25_ESM.zip › Source Data for Expanded View and Appendix/Figure EV1/1G/sgIRTKS-enlarged.jpg]

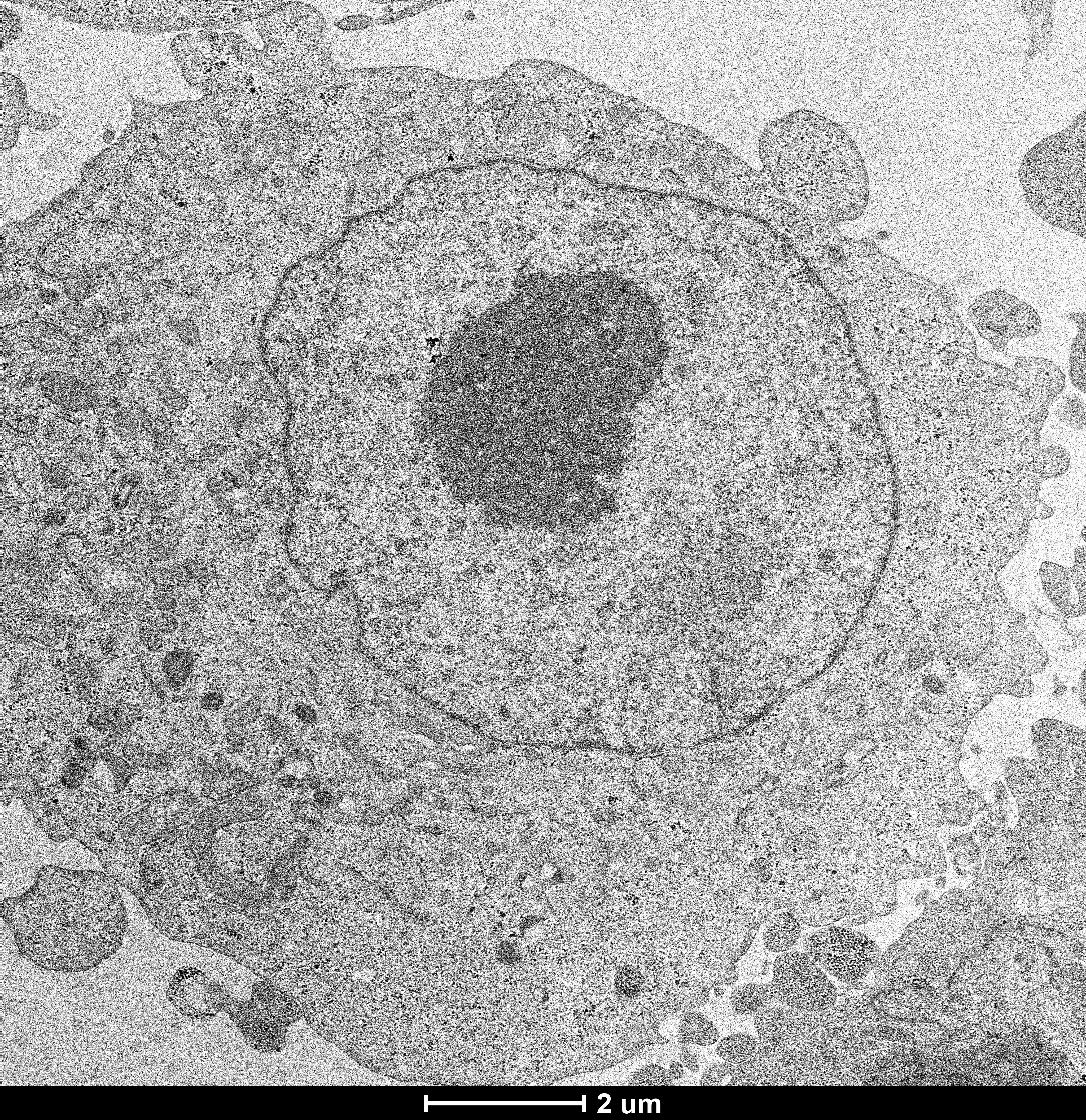

Supplement: Supplementary file 25 — Figure Source Data for Expanded View and Appendix [file 44318_2024_212_MOESM25_ESM.zip › Source Data for Expanded View and Appendix/Figure EV1/1G/sgIRTKS.jpg]

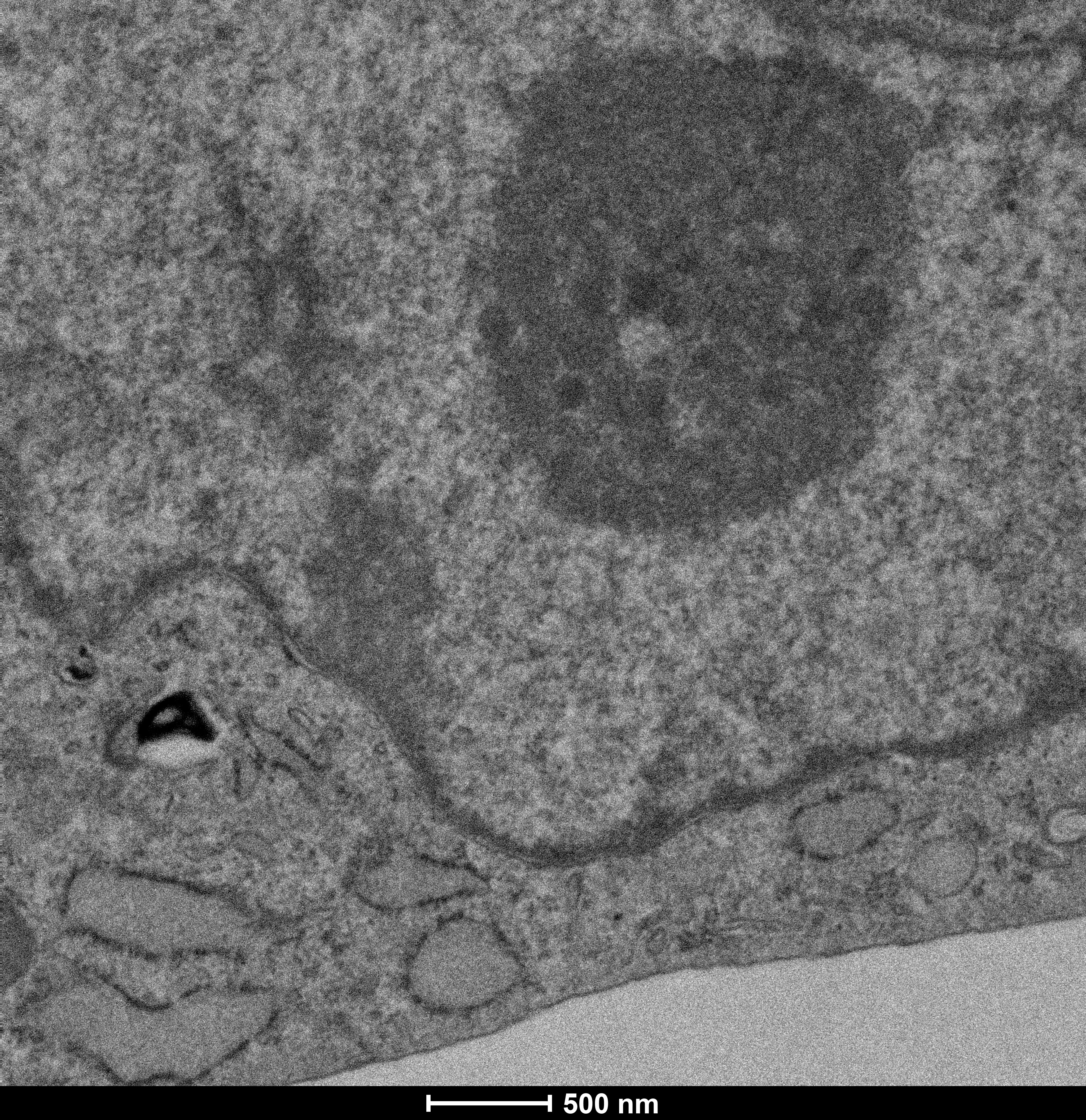

Supplement: Supplementary file 25 — Figure Source Data for Expanded View and Appendix [file 44318_2024_212_MOESM25_ESM.zip › Source Data for Expanded View and Appendix/Figure EV1/1J/E.V.-enlarged.jpg]

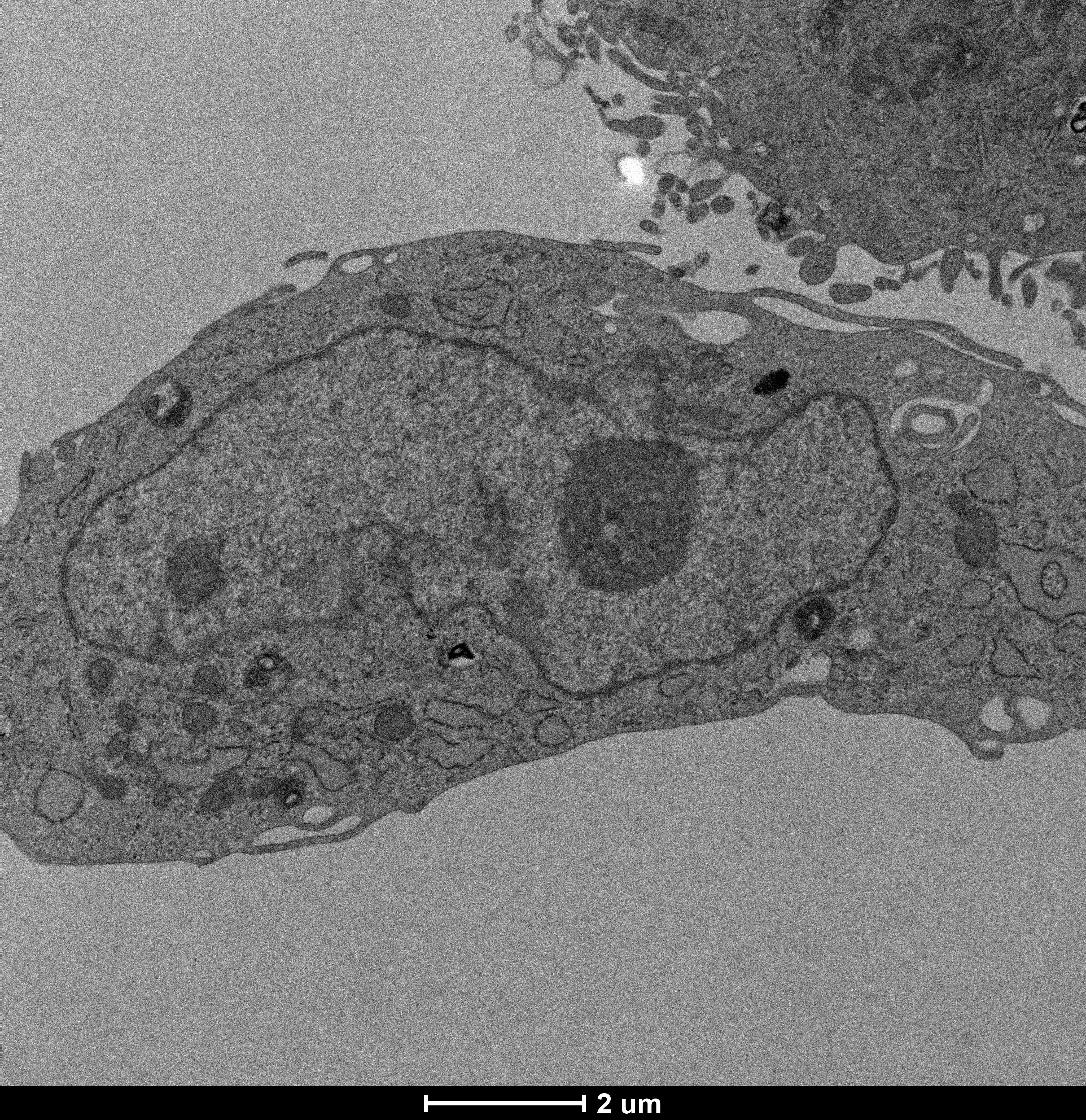

Supplement: Supplementary file 25 — Figure Source Data for Expanded View and Appendix [file 44318_2024_212_MOESM25_ESM.zip › Source Data for Expanded View and Appendix/Figure EV1/1J/E.V..jpg]
