# Supplementary figures and images for: Heterochromatin formation and remodeling by IRTKS condensates counteract cellular senescence (part 4 of 4)
Source: EMBO J. 2024 Aug 27;43(20):7. doi: 10.1038/s44318-024-00212-3 (PMC11480336; doi:10.1038/s44318-024-00212-3)

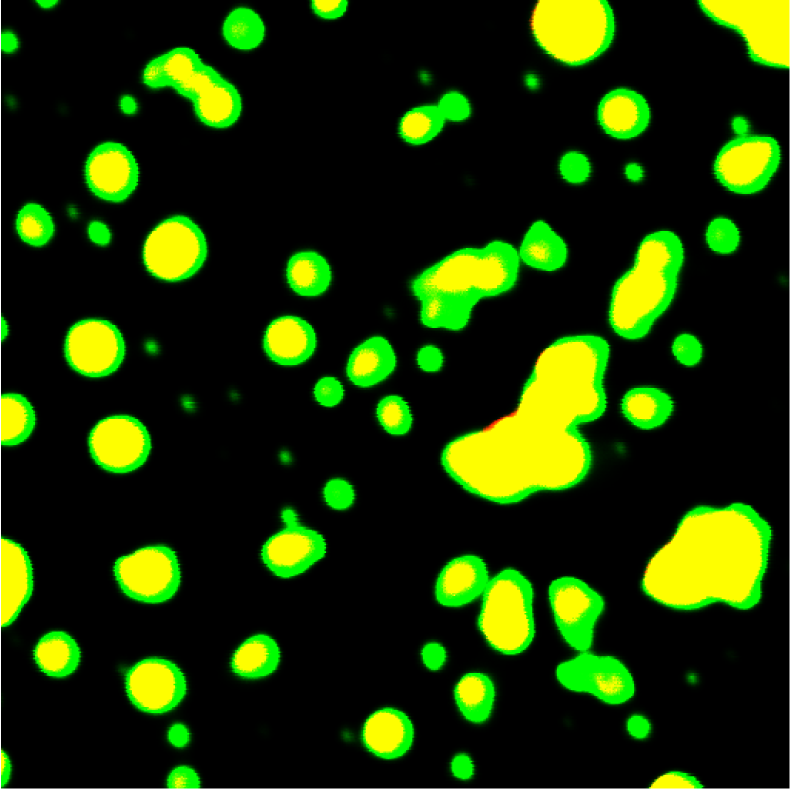

Supplement: Supplementary file 25 — Figure Source Data for Expanded View and Appendix [file 44318_2024_212_MOESM25_ESM.zip › Source Data for Expanded View and Appendix/Figure EV5/5A/20 irtks 20 hp1a 500x 2.tif]

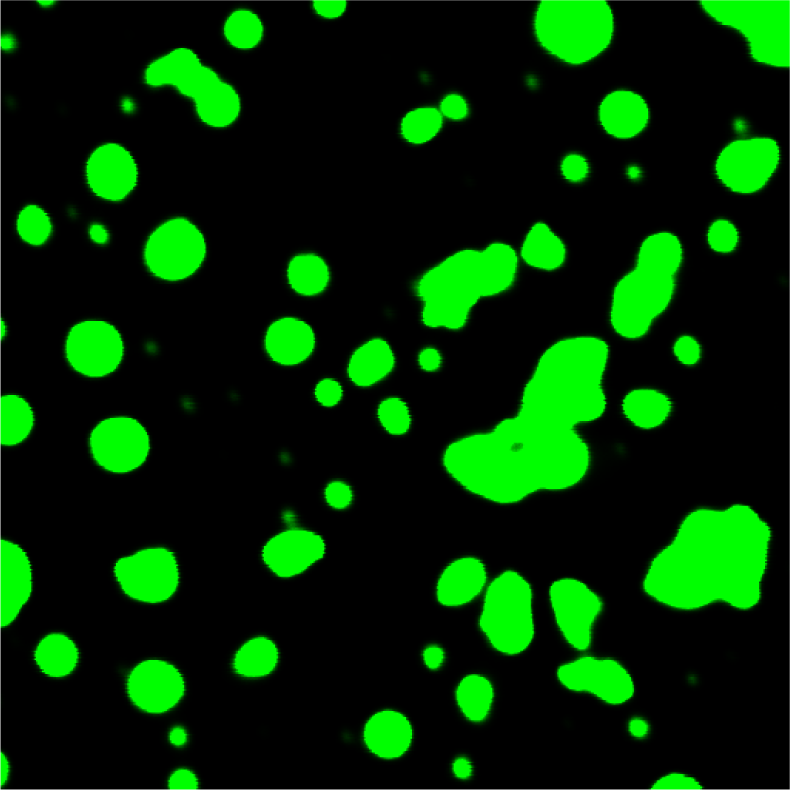

Supplement: Supplementary file 25 — Figure Source Data for Expanded View and Appendix [file 44318_2024_212_MOESM25_ESM.zip › Source Data for Expanded View and Appendix/Figure EV5/5A/20 irtks 20 hp1a 500x 2c1.tif]

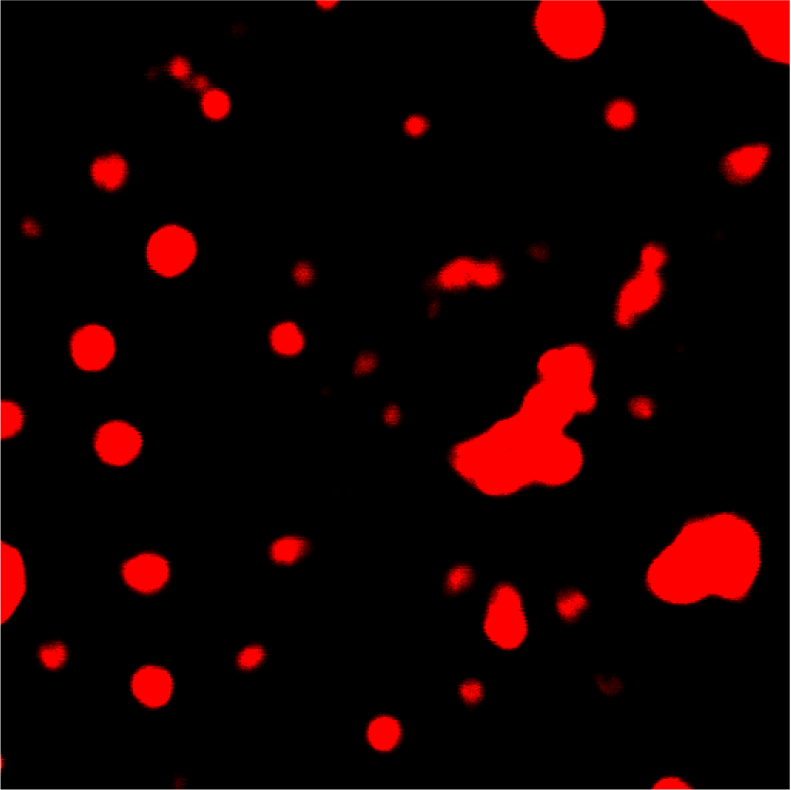

Supplement: Supplementary file 25 — Figure Source Data for Expanded View and Appendix [file 44318_2024_212_MOESM25_ESM.zip › Source Data for Expanded View and Appendix/Figure EV5/5A/20 irtks 20 hp1a 500x 2c2.tif]

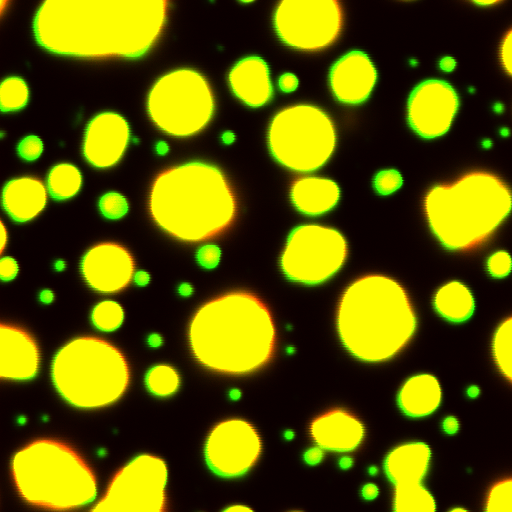

Supplement: Supplementary file 25 — Figure Source Data for Expanded View and Appendix [file 44318_2024_212_MOESM25_ESM.zip › Source Data for Expanded View and Appendix/Figure EV5/5A/20 irtks 40 hp1a 500x 4.tif]

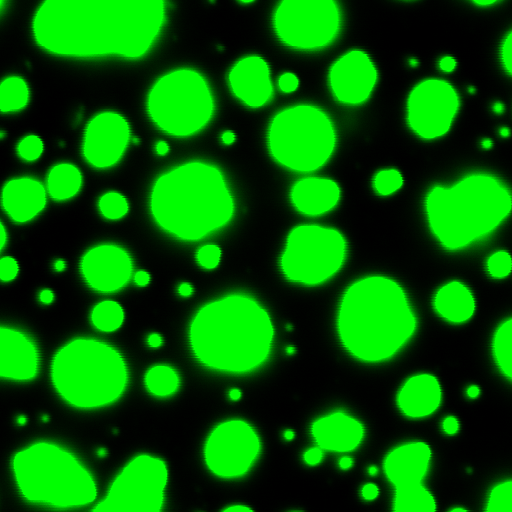

Supplement: Supplementary file 25 — Figure Source Data for Expanded View and Appendix [file 44318_2024_212_MOESM25_ESM.zip › Source Data for Expanded View and Appendix/Figure EV5/5A/20 irtks 40 hp1a 500x 4c1.tif]

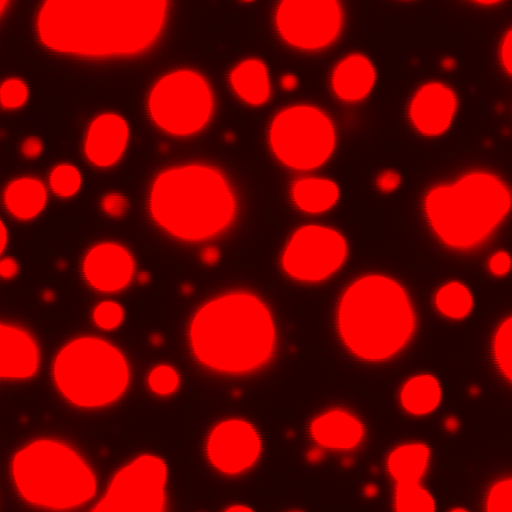

Supplement: Supplementary file 25 — Figure Source Data for Expanded View and Appendix [file 44318_2024_212_MOESM25_ESM.zip › Source Data for Expanded View and Appendix/Figure EV5/5A/20 irtks 40 hp1a 500x 4c2.tif]

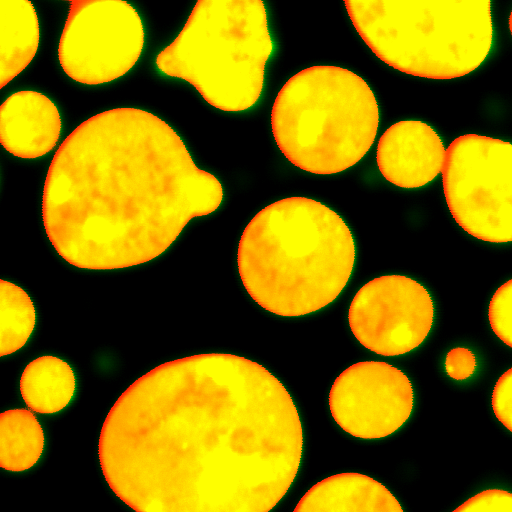

Supplement: Supplementary file 25 — Figure Source Data for Expanded View and Appendix [file 44318_2024_212_MOESM25_ESM.zip › Source Data for Expanded View and Appendix/Figure EV5/5A/20 irtks 80 hp1a 500x 2.tif]

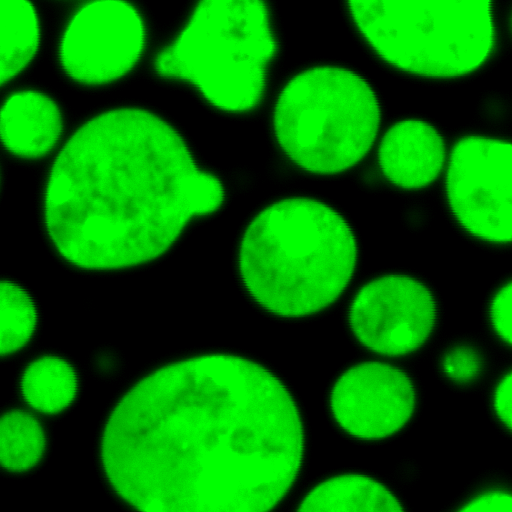

Supplement: Supplementary file 25 — Figure Source Data for Expanded View and Appendix [file 44318_2024_212_MOESM25_ESM.zip › Source Data for Expanded View and Appendix/Figure EV5/5A/20 irtks 80 hp1a 500x 2c1.tif]

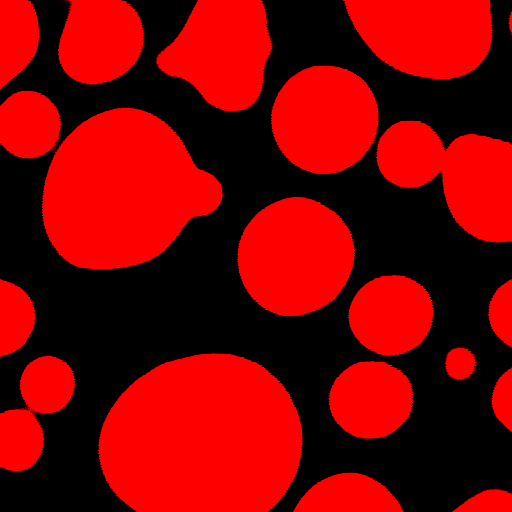

Supplement: Supplementary file 25 — Figure Source Data for Expanded View and Appendix [file 44318_2024_212_MOESM25_ESM.zip › Source Data for Expanded View and Appendix/Figure EV5/5A/20 irtks 80 hp1a 500x 2c2.tif]

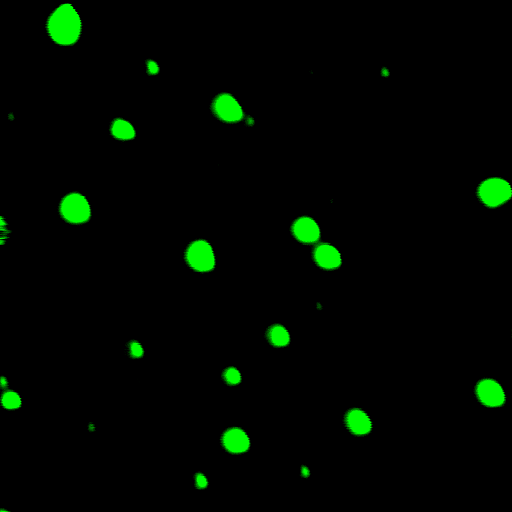

Supplement: Supplementary file 25 — Figure Source Data for Expanded View and Appendix [file 44318_2024_212_MOESM25_ESM.zip › Source Data for Expanded View and Appendix/Figure EV5/5A/5 irtks 0 hp1a 500x 4.tif]

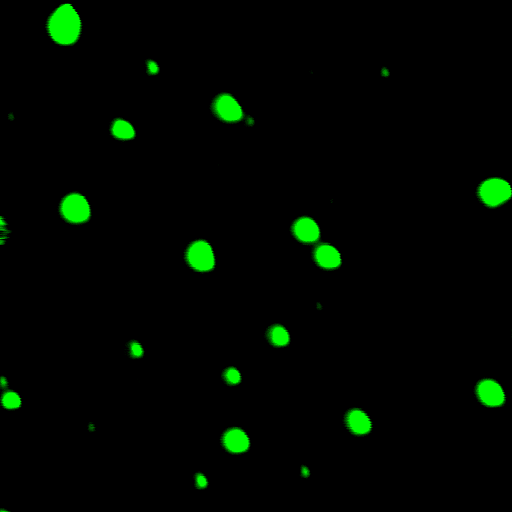

Supplement: Supplementary file 25 — Figure Source Data for Expanded View and Appendix [file 44318_2024_212_MOESM25_ESM.zip › Source Data for Expanded View and Appendix/Figure EV5/5A/5 irtks 0 hp1a 500x 4c1.tif]

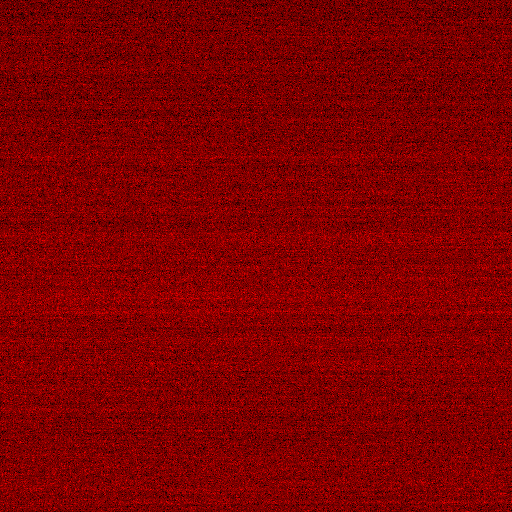

Supplement: Supplementary file 25 — Figure Source Data for Expanded View and Appendix [file 44318_2024_212_MOESM25_ESM.zip › Source Data for Expanded View and Appendix/Figure EV5/5A/5 irtks 0 hp1a 500x 4c2.tif]

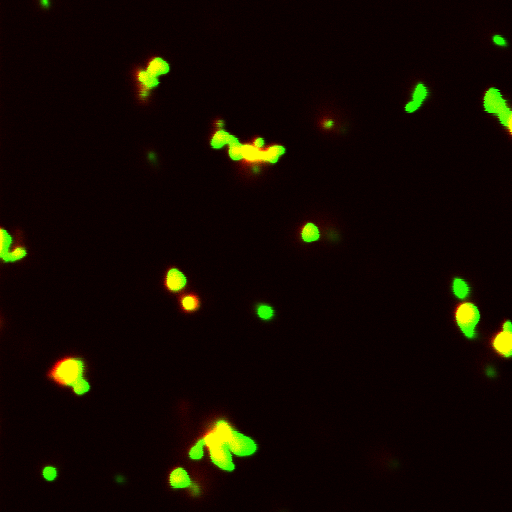

Supplement: Supplementary file 25 — Figure Source Data for Expanded View and Appendix [file 44318_2024_212_MOESM25_ESM.zip › Source Data for Expanded View and Appendix/Figure EV5/5A/5 irtks 20 hp1a 500x.tif]

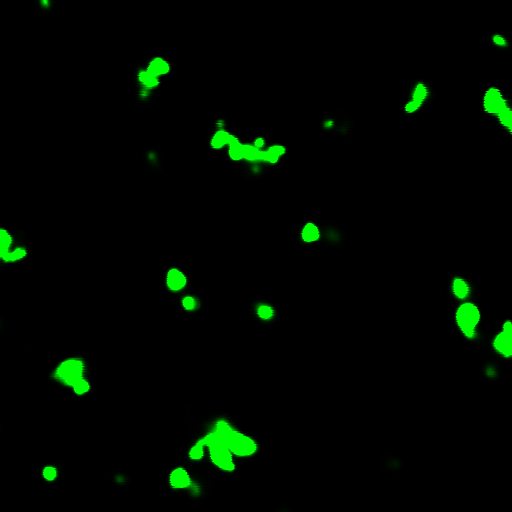

Supplement: Supplementary file 25 — Figure Source Data for Expanded View and Appendix [file 44318_2024_212_MOESM25_ESM.zip › Source Data for Expanded View and Appendix/Figure EV5/5A/5 irtks 20 hp1a 500xc1.tif]

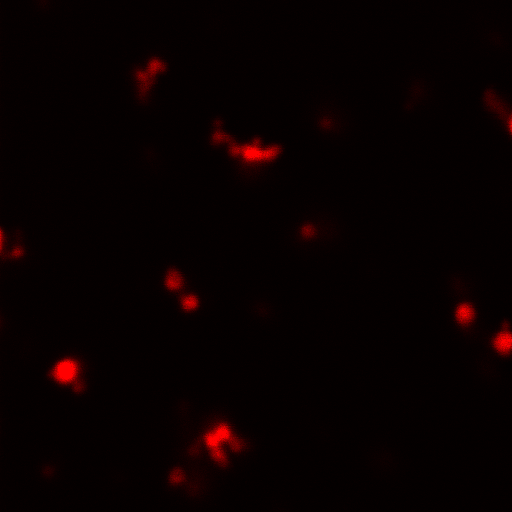

Supplement: Supplementary file 25 — Figure Source Data for Expanded View and Appendix [file 44318_2024_212_MOESM25_ESM.zip › Source Data for Expanded View and Appendix/Figure EV5/5A/5 irtks 20 hp1a 500xc2.tif]

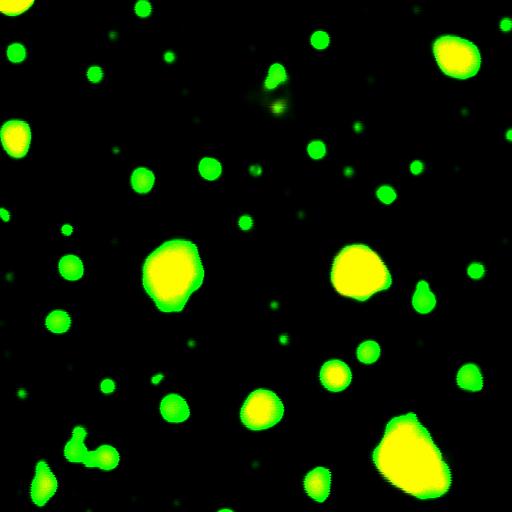

Supplement: Supplementary file 25 — Figure Source Data for Expanded View and Appendix [file 44318_2024_212_MOESM25_ESM.zip › Source Data for Expanded View and Appendix/Figure EV5/5A/5 irtks 40 hp1a 500x 2.tif]

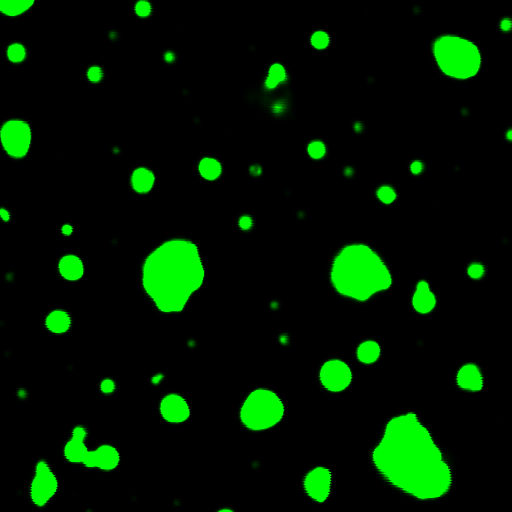

Supplement: Supplementary file 25 — Figure Source Data for Expanded View and Appendix [file 44318_2024_212_MOESM25_ESM.zip › Source Data for Expanded View and Appendix/Figure EV5/5A/5 irtks 40 hp1a 500x 2c1.tif]

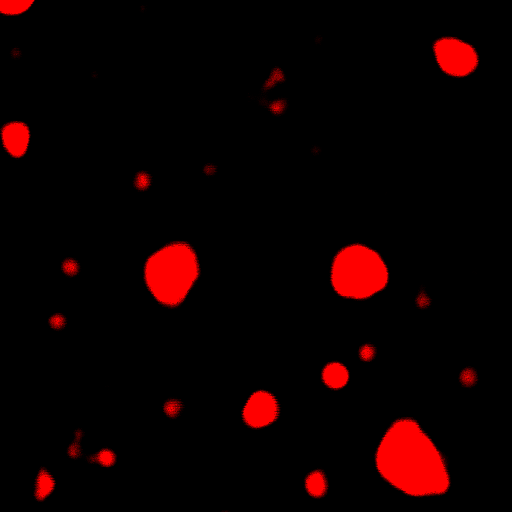

Supplement: Supplementary file 25 — Figure Source Data for Expanded View and Appendix [file 44318_2024_212_MOESM25_ESM.zip › Source Data for Expanded View and Appendix/Figure EV5/5A/5 irtks 40 hp1a 500x 2c2.tif]

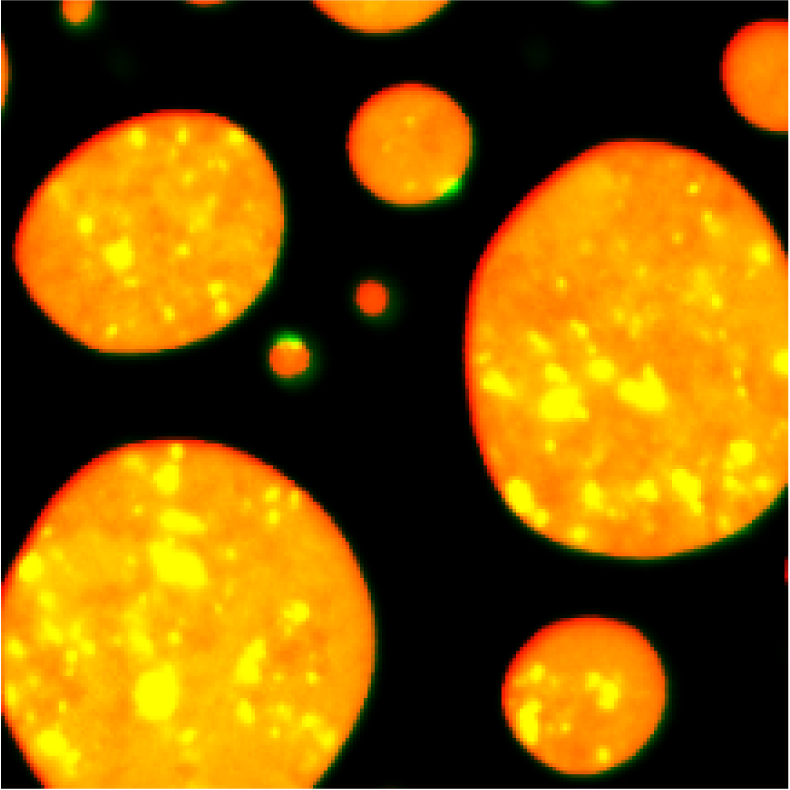

Supplement: Supplementary file 25 — Figure Source Data for Expanded View and Appendix [file 44318_2024_212_MOESM25_ESM.zip › Source Data for Expanded View and Appendix/Figure EV5/5A/5 irtks 80 hp1a 500xc.tif]

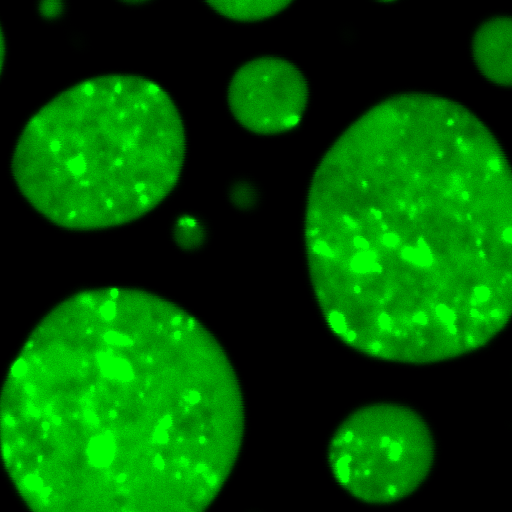

Supplement: Supplementary file 25 — Figure Source Data for Expanded View and Appendix [file 44318_2024_212_MOESM25_ESM.zip › Source Data for Expanded View and Appendix/Figure EV5/5A/5 irtks 80 hp1a 500xc1.tif]

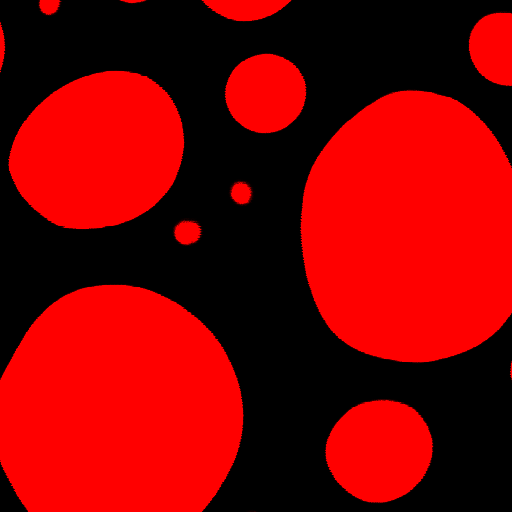

Supplement: Supplementary file 25 — Figure Source Data for Expanded View and Appendix [file 44318_2024_212_MOESM25_ESM.zip › Source Data for Expanded View and Appendix/Figure EV5/5A/5 irtks 80 hp1a 500xc2.tif]

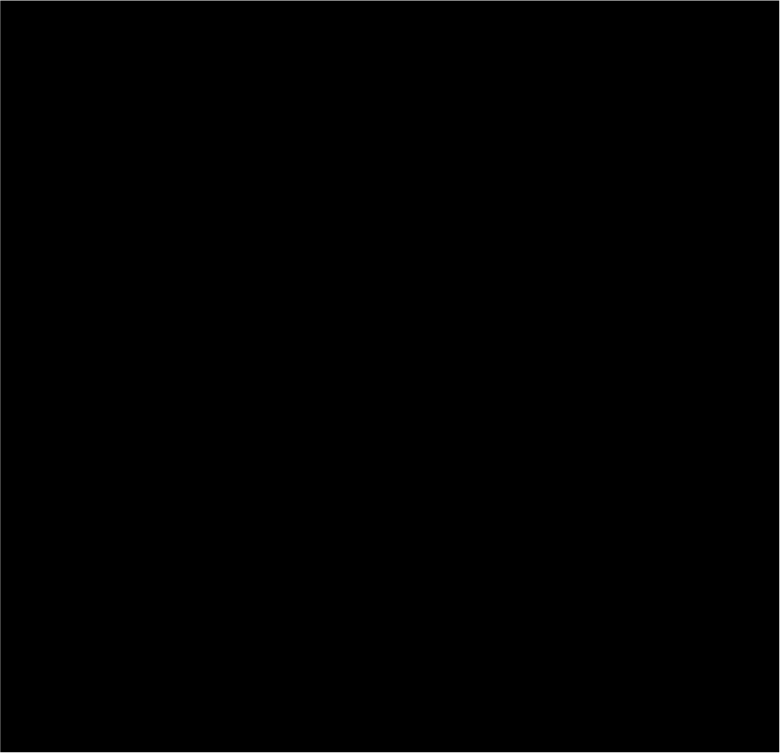

Supplement: Supplementary file 25 — Figure Source Data for Expanded View and Appendix [file 44318_2024_212_MOESM25_ESM.zip › Source Data for Expanded View and Appendix/Figure EV5/5B/0 IRTKS 0 SUMO-HP1 2-01.tif]

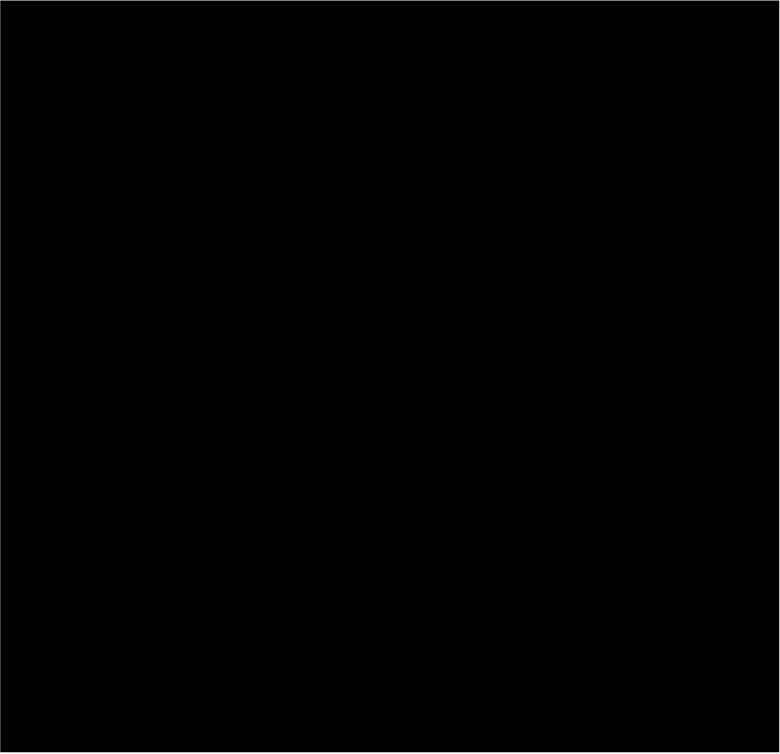

Supplement: Supplementary file 25 — Figure Source Data for Expanded View and Appendix [file 44318_2024_212_MOESM25_ESM.zip › Source Data for Expanded View and Appendix/Figure EV5/5B/0 IRTKS 0 SUMO-HP1 2c1-01.tif]

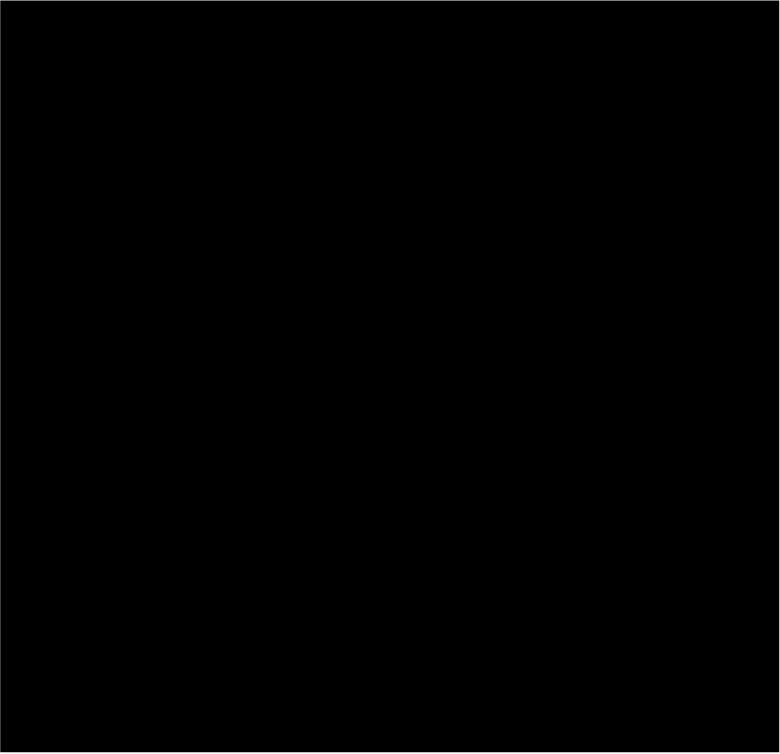

Supplement: Supplementary file 25 — Figure Source Data for Expanded View and Appendix [file 44318_2024_212_MOESM25_ESM.zip › Source Data for Expanded View and Appendix/Figure EV5/5B/0 IRTKS 0 SUMO-HP1 2c2-01.tif]

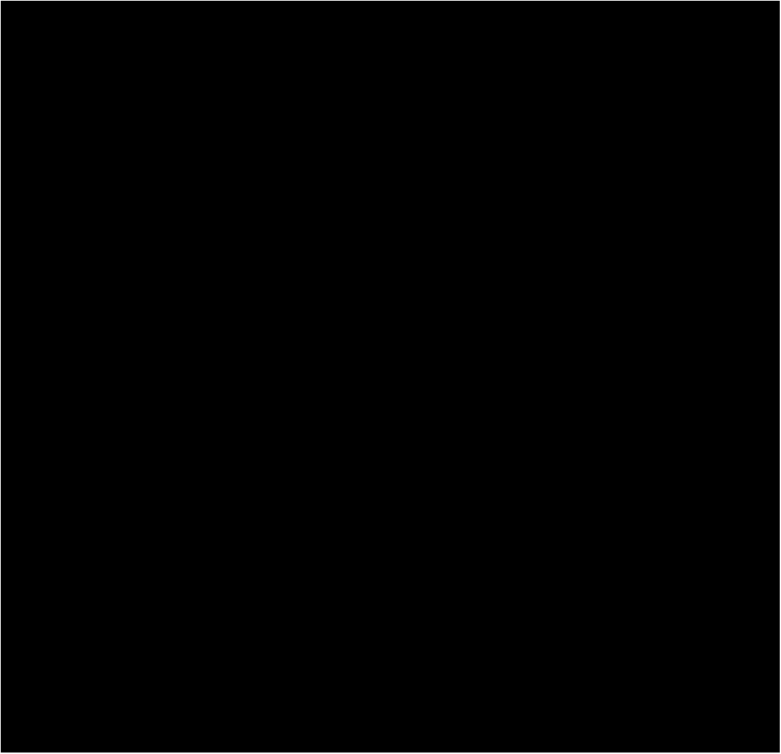

Supplement: Supplementary file 25 — Figure Source Data for Expanded View and Appendix [file 44318_2024_212_MOESM25_ESM.zip › Source Data for Expanded View and Appendix/Figure EV5/5B/0 IRTKS 20 SUMO-HP1 c1-01.tif]

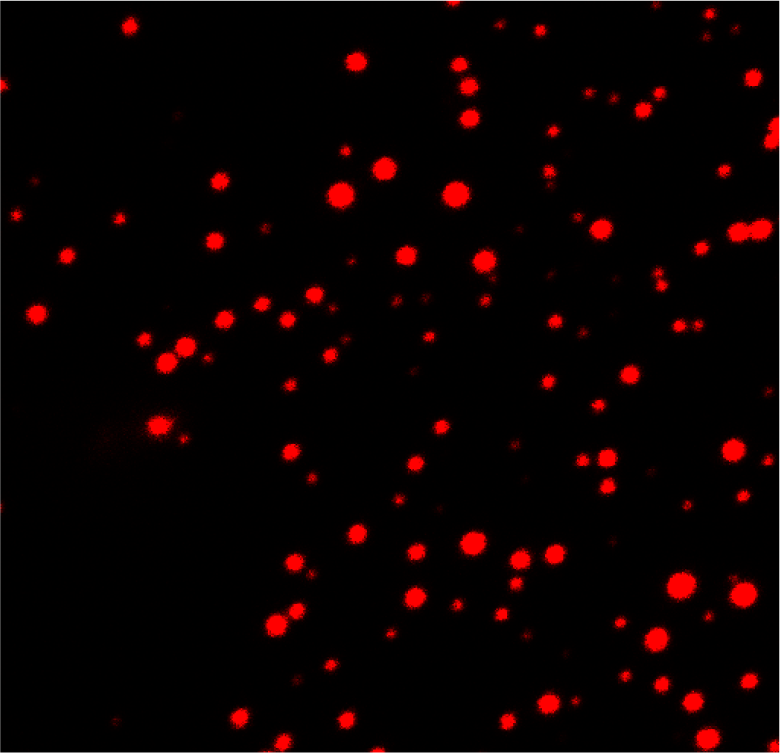

Supplement: Supplementary file 25 — Figure Source Data for Expanded View and Appendix [file 44318_2024_212_MOESM25_ESM.zip › Source Data for Expanded View and Appendix/Figure EV5/5B/0 IRTKS 20 SUMO-HP1 c2-01.tif]

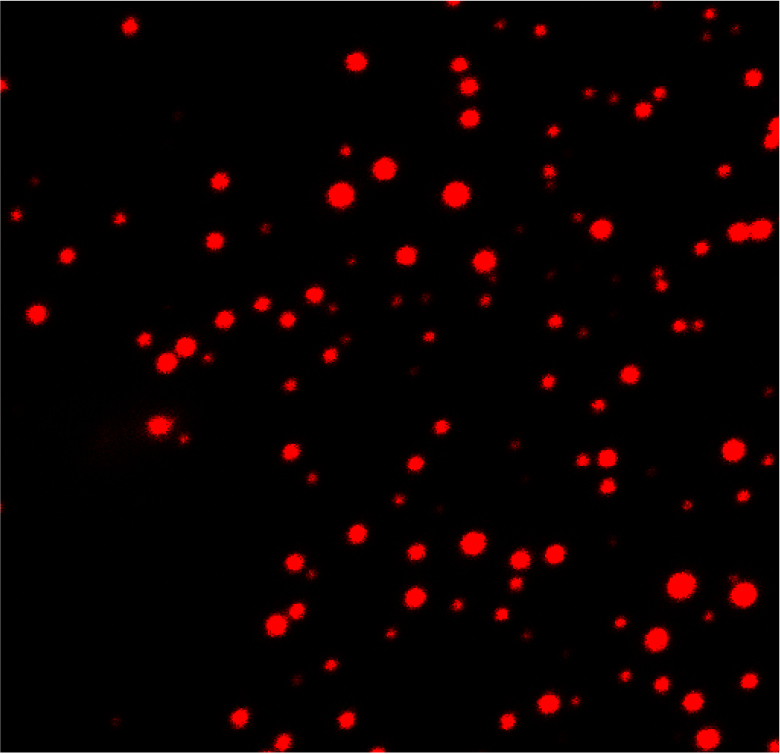

Supplement: Supplementary file 25 — Figure Source Data for Expanded View and Appendix [file 44318_2024_212_MOESM25_ESM.zip › Source Data for Expanded View and Appendix/Figure EV5/5B/0 IRTKS 20 SUMO-HP1-01.tif]

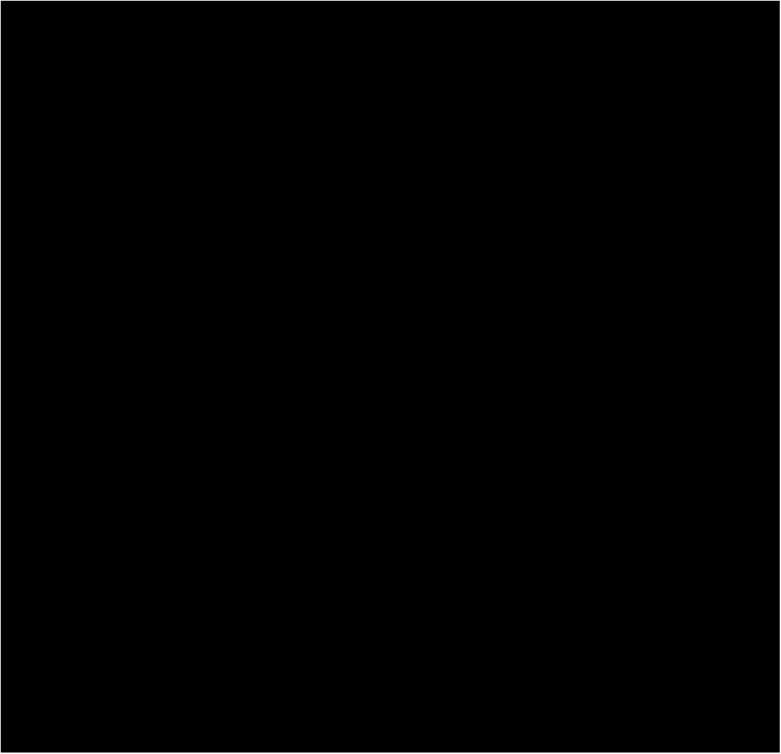

Supplement: Supplementary file 25 — Figure Source Data for Expanded View and Appendix [file 44318_2024_212_MOESM25_ESM.zip › Source Data for Expanded View and Appendix/Figure EV5/5B/0 IRTKS 40 SUMO-HP1 c1-01.tif]

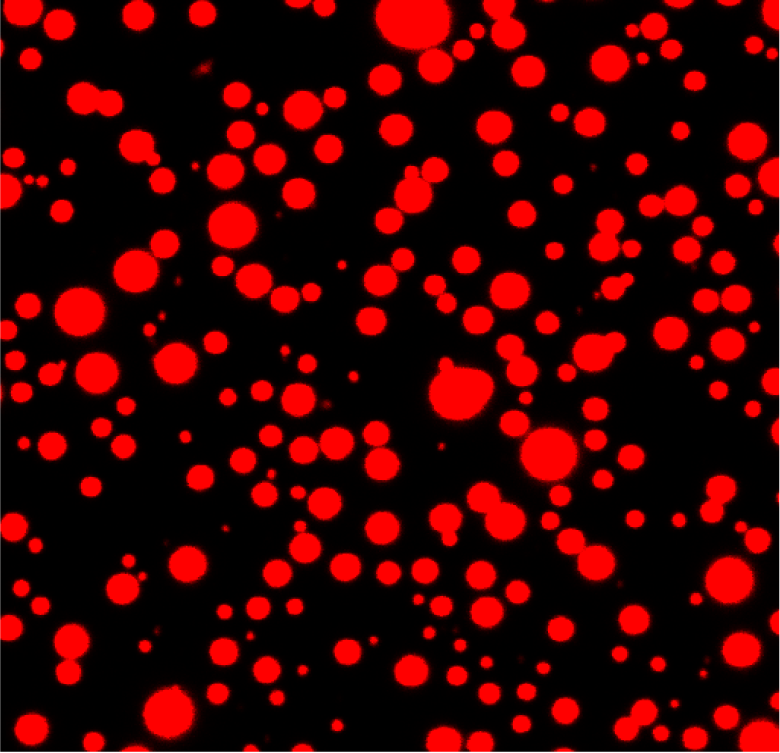

Supplement: Supplementary file 25 — Figure Source Data for Expanded View and Appendix [file 44318_2024_212_MOESM25_ESM.zip › Source Data for Expanded View and Appendix/Figure EV5/5B/0 IRTKS 40 SUMO-HP1-01.tif]

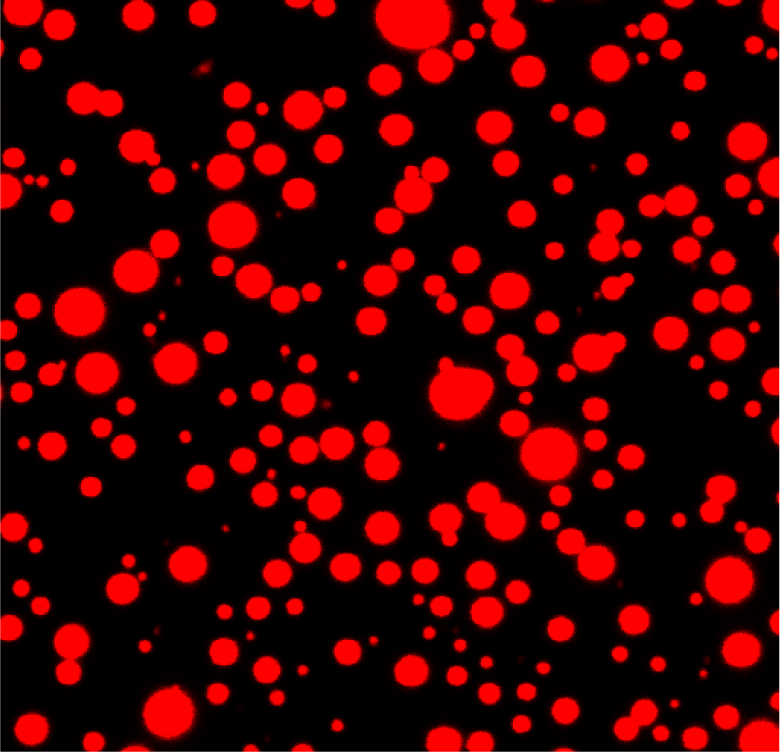

Supplement: Supplementary file 25 — Figure Source Data for Expanded View and Appendix [file 44318_2024_212_MOESM25_ESM.zip › Source Data for Expanded View and Appendix/Figure EV5/5B/0 IRTKS 40 SUMO-HP1c2-01.tif]

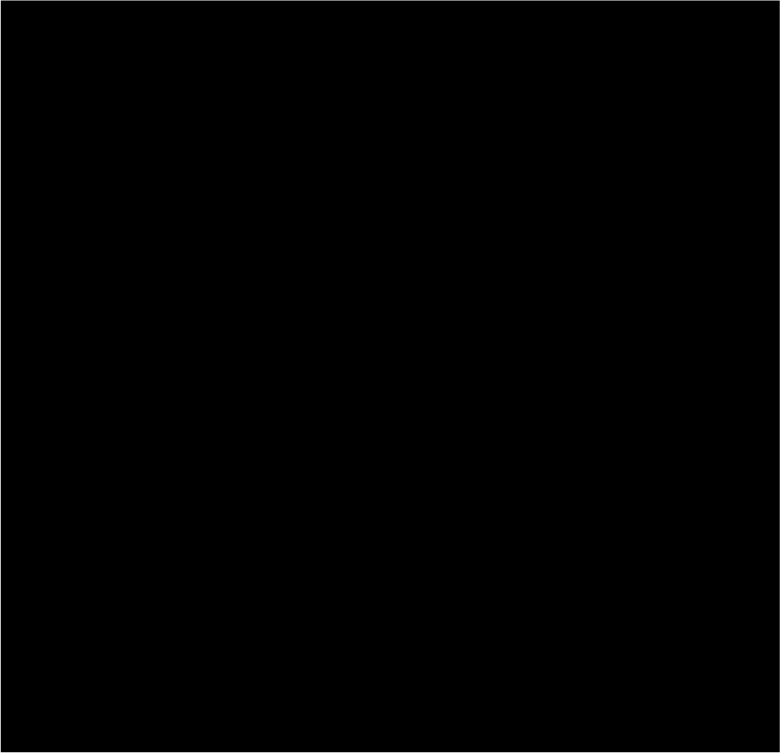

Supplement: Supplementary file 25 — Figure Source Data for Expanded View and Appendix [file 44318_2024_212_MOESM25_ESM.zip › Source Data for Expanded View and Appendix/Figure EV5/5B/0 IRTKS 80 SUMO-HP1 c1-01.tif]

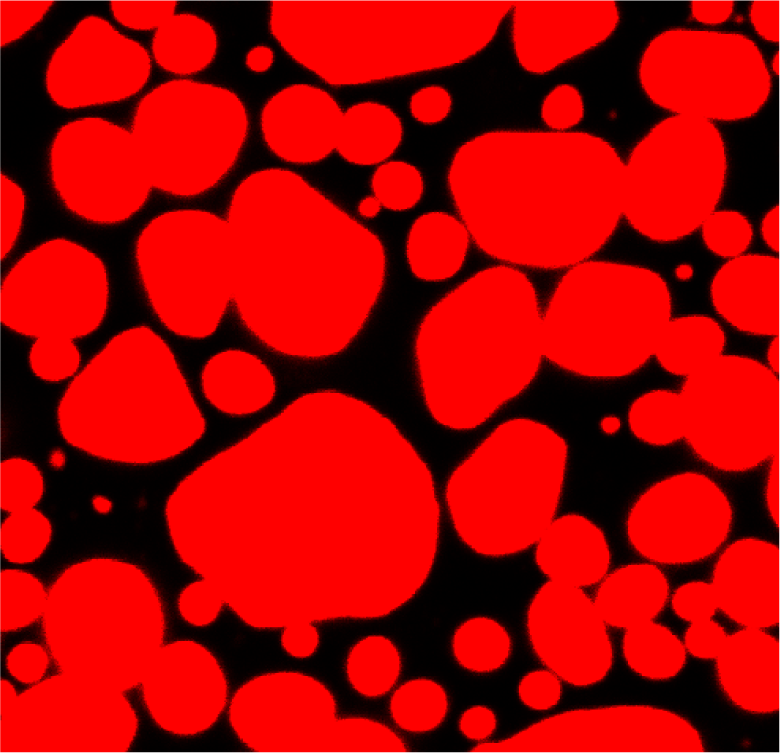

Supplement: Supplementary file 25 — Figure Source Data for Expanded View and Appendix [file 44318_2024_212_MOESM25_ESM.zip › Source Data for Expanded View and Appendix/Figure EV5/5B/0 IRTKS 80 SUMO-HP1 c2-01.tif]

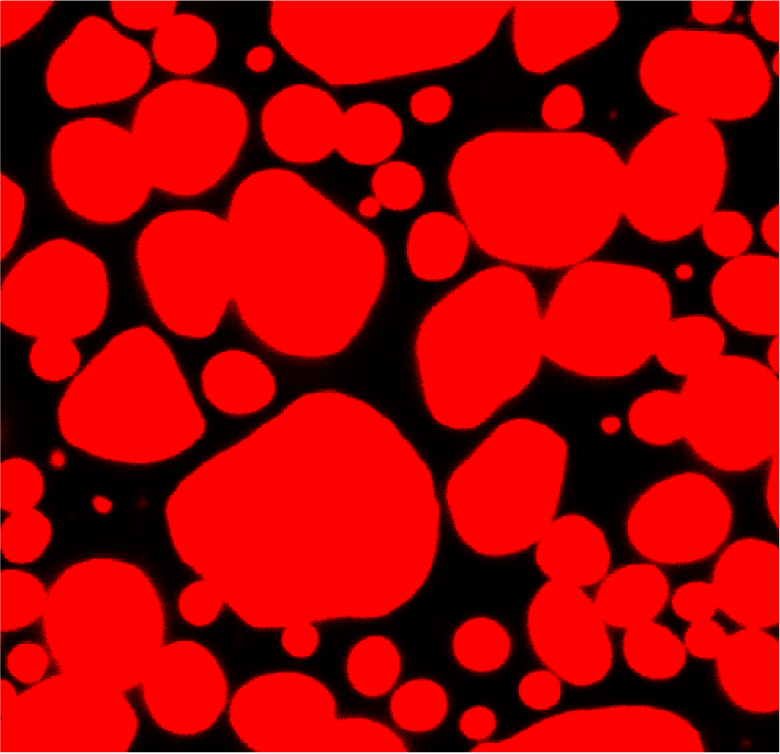

Supplement: Supplementary file 25 — Figure Source Data for Expanded View and Appendix [file 44318_2024_212_MOESM25_ESM.zip › Source Data for Expanded View and Appendix/Figure EV5/5B/0 IRTKS 80 SUMO-HP1-01.tif]

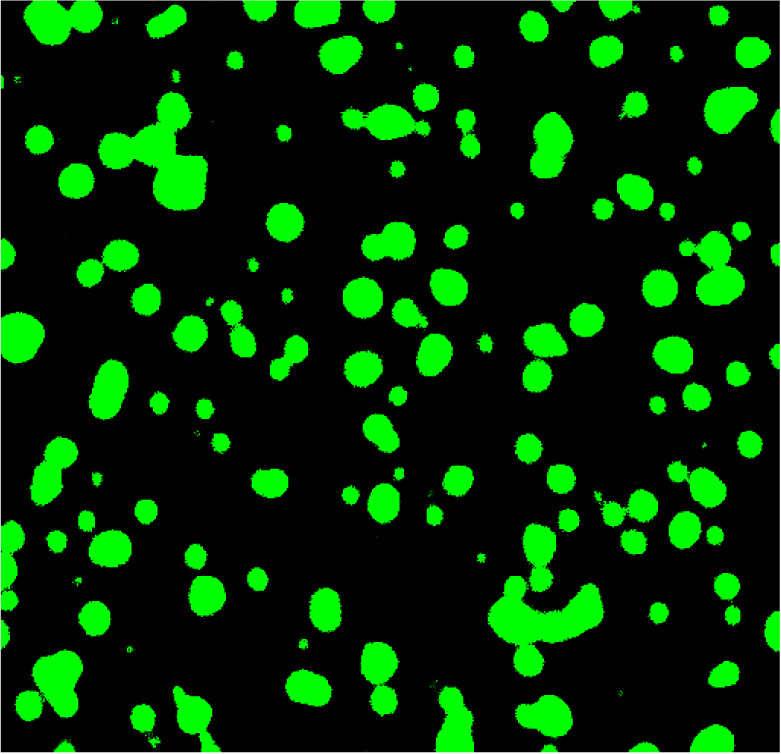

Supplement: Supplementary file 25 — Figure Source Data for Expanded View and Appendix [file 44318_2024_212_MOESM25_ESM.zip › Source Data for Expanded View and Appendix/Figure EV5/5B/10 IRTKS 0 SUMO-HP1 c1-01.tif]

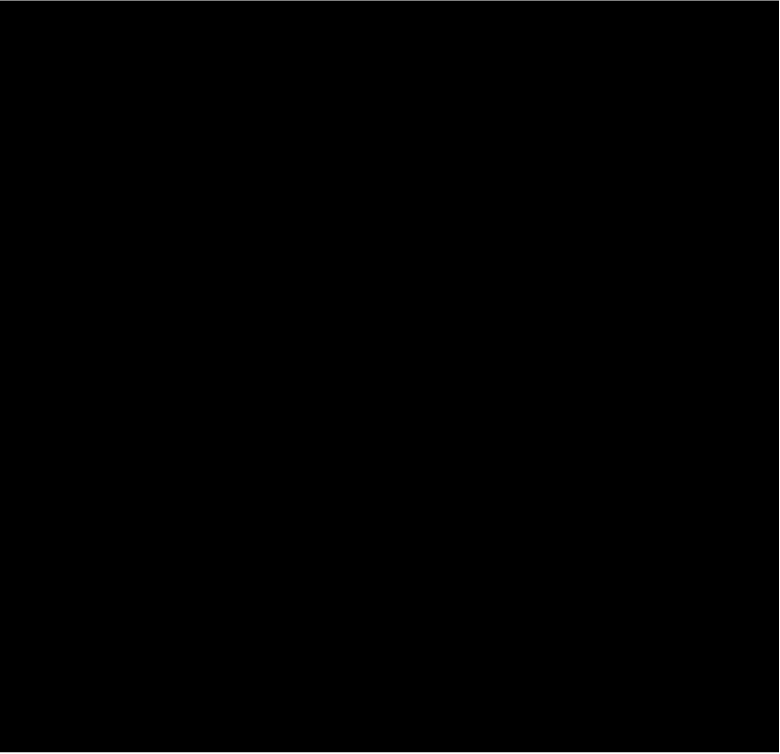

Supplement: Supplementary file 25 — Figure Source Data for Expanded View and Appendix [file 44318_2024_212_MOESM25_ESM.zip › Source Data for Expanded View and Appendix/Figure EV5/5B/10 IRTKS 0 SUMO-HP1 c2-01.tif]

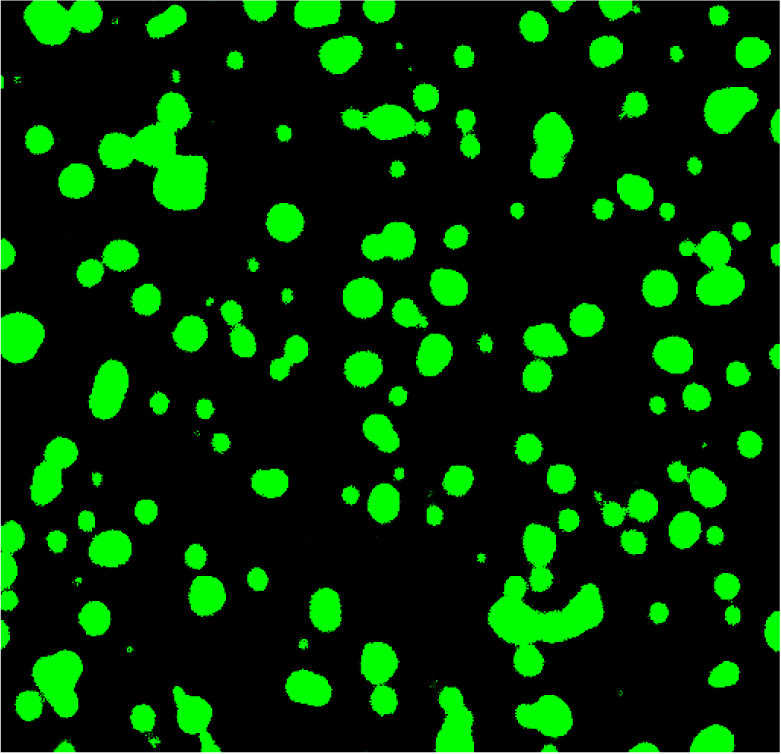

Supplement: Supplementary file 25 — Figure Source Data for Expanded View and Appendix [file 44318_2024_212_MOESM25_ESM.zip › Source Data for Expanded View and Appendix/Figure EV5/5B/10 IRTKS 0 SUMO-HP1-01.tif]

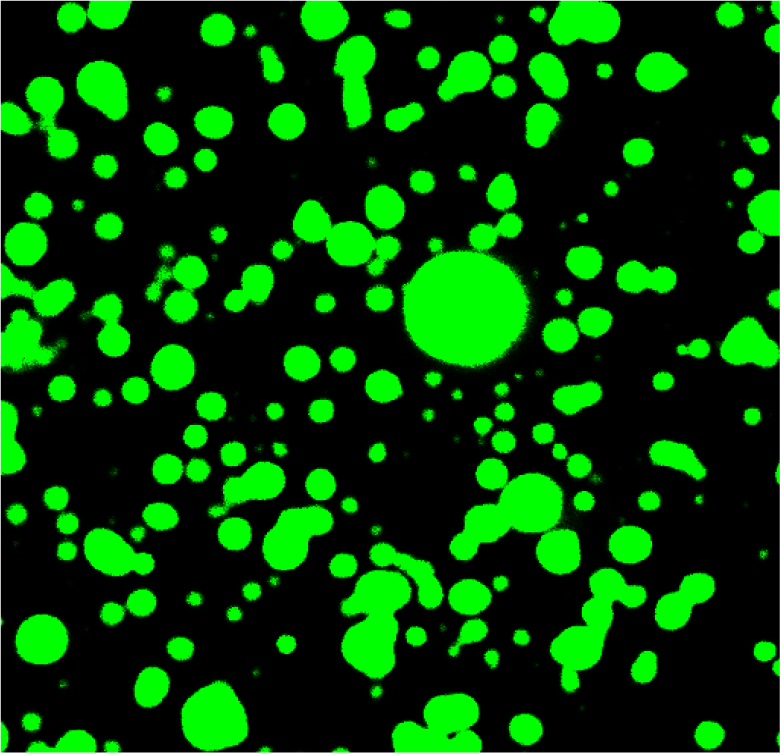

Supplement: Supplementary file 25 — Figure Source Data for Expanded View and Appendix [file 44318_2024_212_MOESM25_ESM.zip › Source Data for Expanded View and Appendix/Figure EV5/5B/10 IRTKS 20 SUMO-HP1 c1-01.tif]

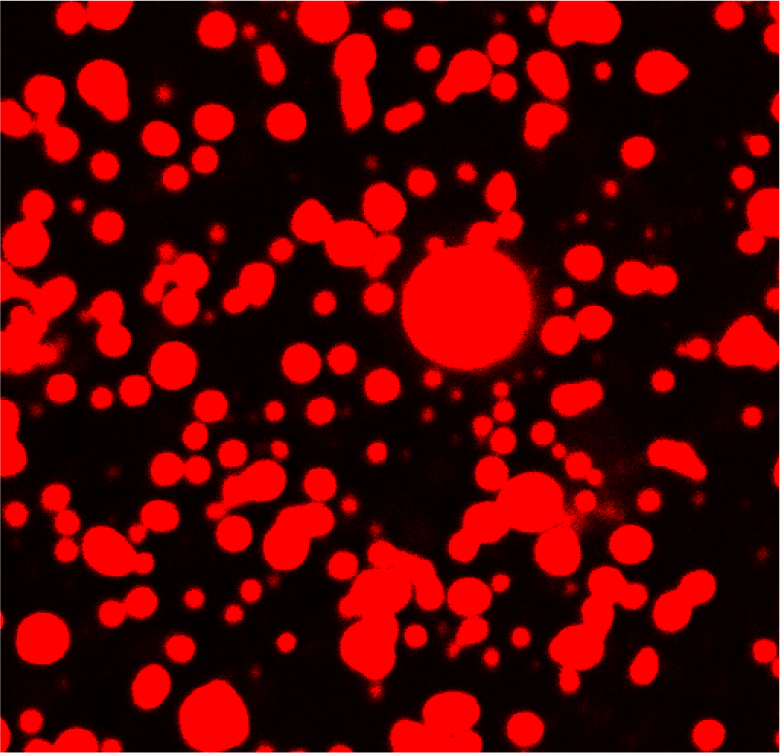

Supplement: Supplementary file 25 — Figure Source Data for Expanded View and Appendix [file 44318_2024_212_MOESM25_ESM.zip › Source Data for Expanded View and Appendix/Figure EV5/5B/10 IRTKS 20 SUMO-HP1 c2-01.tif]

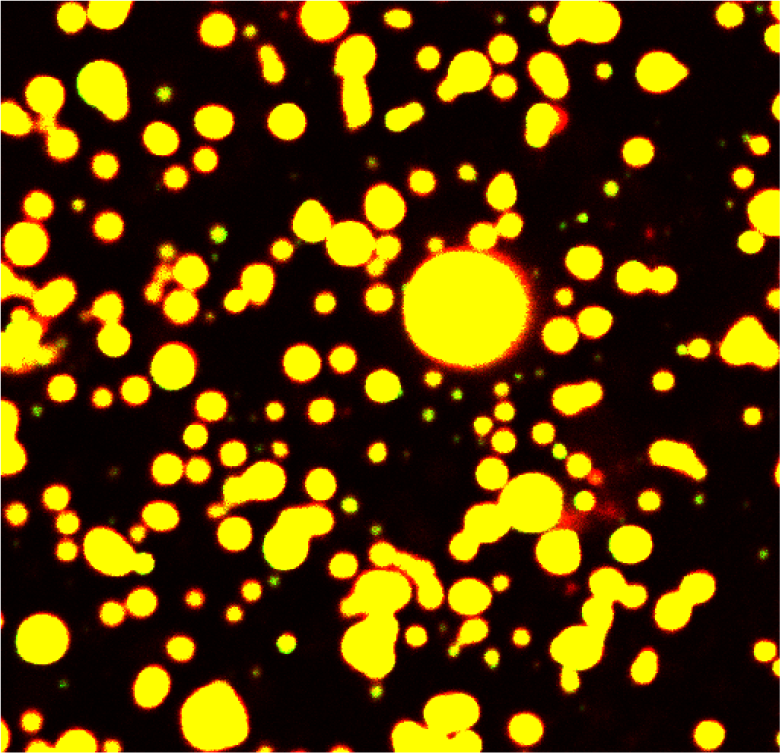

Supplement: Supplementary file 25 — Figure Source Data for Expanded View and Appendix [file 44318_2024_212_MOESM25_ESM.zip › Source Data for Expanded View and Appendix/Figure EV5/5B/10 IRTKS 20 SUMO-HP1-01.tif]

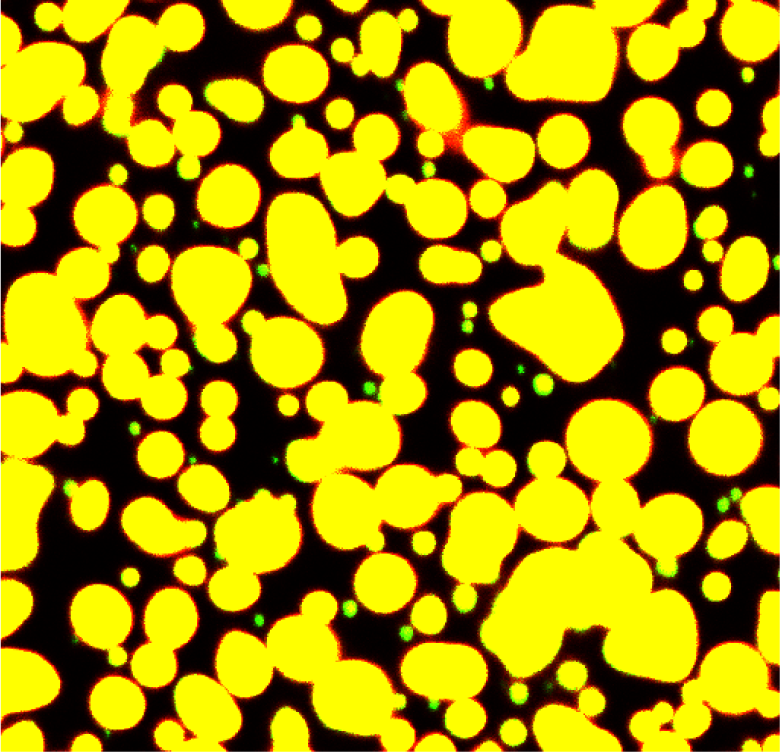

Supplement: Supplementary file 25 — Figure Source Data for Expanded View and Appendix [file 44318_2024_212_MOESM25_ESM.zip › Source Data for Expanded View and Appendix/Figure EV5/5B/10 IRTKS 40 SUMO-HP1-01.tif]

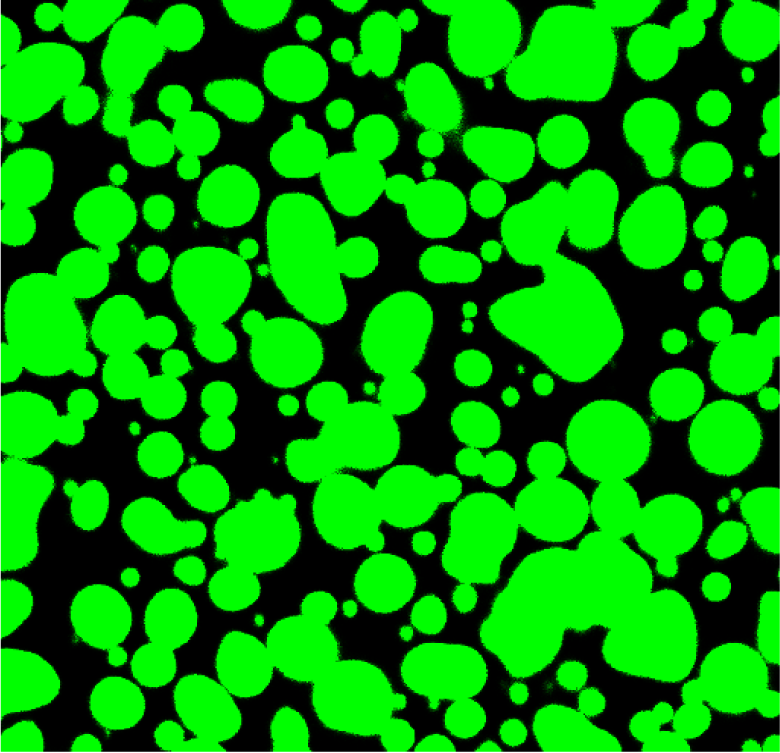

Supplement: Supplementary file 25 — Figure Source Data for Expanded View and Appendix [file 44318_2024_212_MOESM25_ESM.zip › Source Data for Expanded View and Appendix/Figure EV5/5B/10 IRTKS 40 SUMO-HP1c1-01.tif]

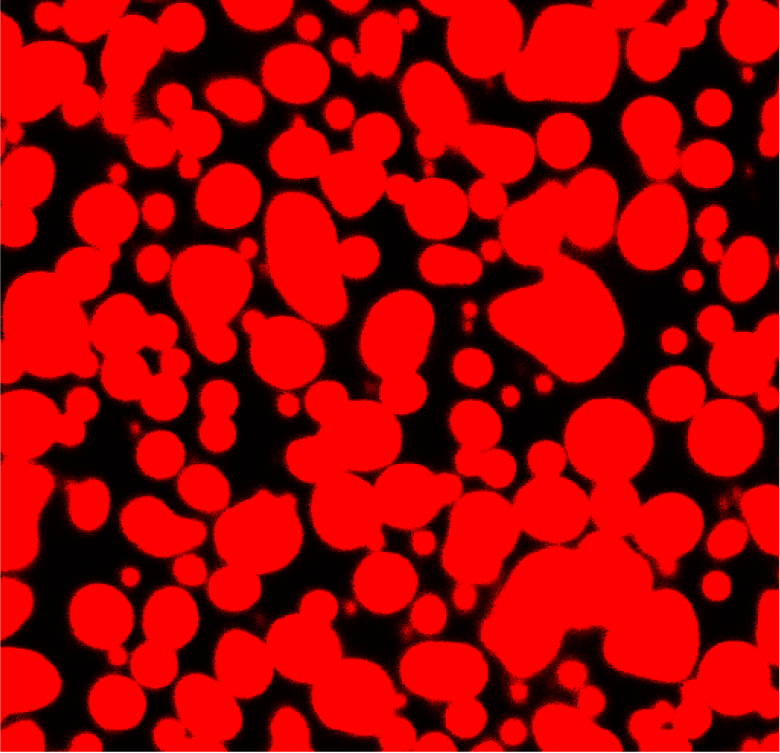

Supplement: Supplementary file 25 — Figure Source Data for Expanded View and Appendix [file 44318_2024_212_MOESM25_ESM.zip › Source Data for Expanded View and Appendix/Figure EV5/5B/10 IRTKS 40 SUMO-HP1c2-01.tif]

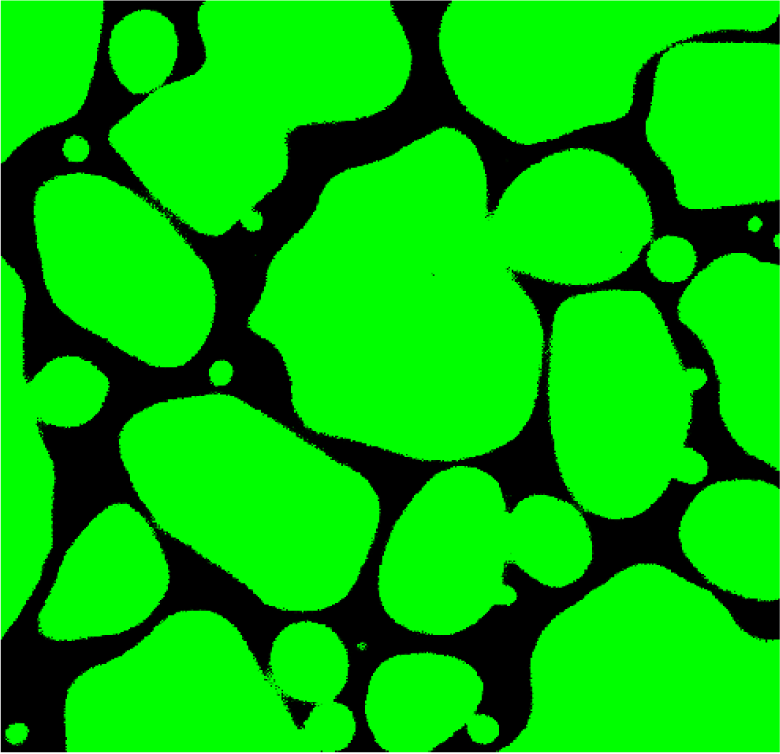

Supplement: Supplementary file 25 — Figure Source Data for Expanded View and Appendix [file 44318_2024_212_MOESM25_ESM.zip › Source Data for Expanded View and Appendix/Figure EV5/5B/10 IRTKS 80 SUMO-HP1 c1-01.tif]

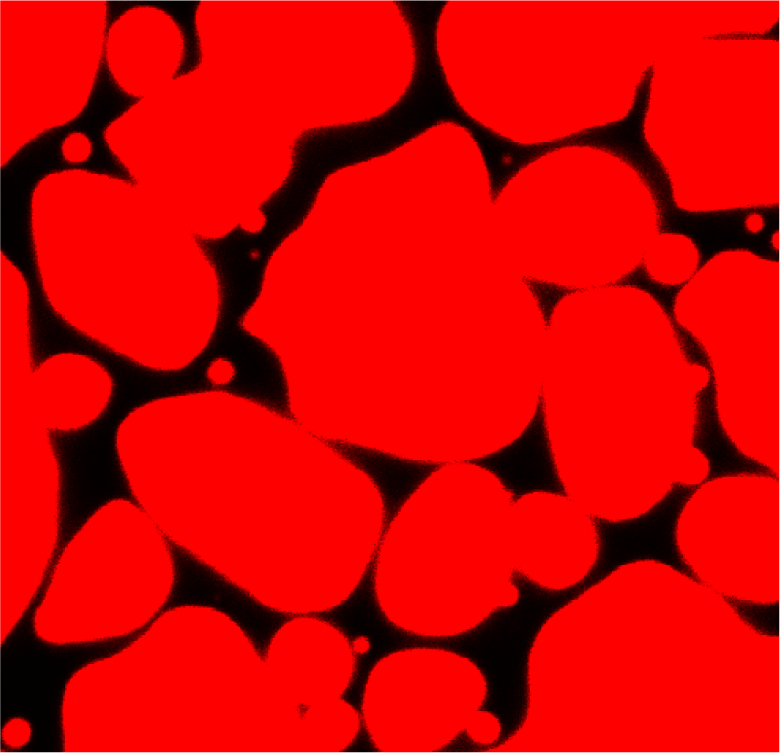

Supplement: Supplementary file 25 — Figure Source Data for Expanded View and Appendix [file 44318_2024_212_MOESM25_ESM.zip › Source Data for Expanded View and Appendix/Figure EV5/5B/10 IRTKS 80 SUMO-HP1 c2-01.tif]

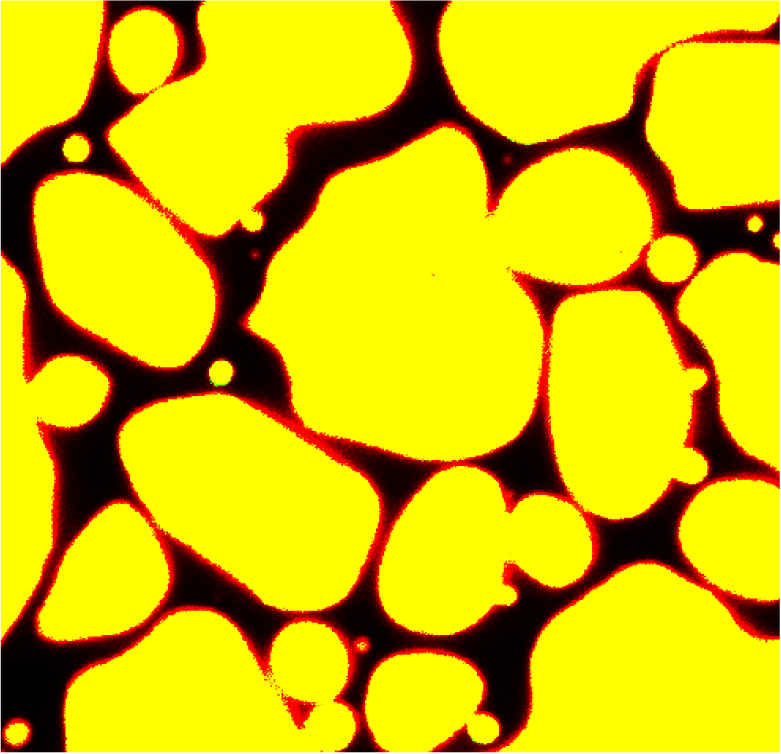

Supplement: Supplementary file 25 — Figure Source Data for Expanded View and Appendix [file 44318_2024_212_MOESM25_ESM.zip › Source Data for Expanded View and Appendix/Figure EV5/5B/10 IRTKS 80 SUMO-HP1-01.tif]

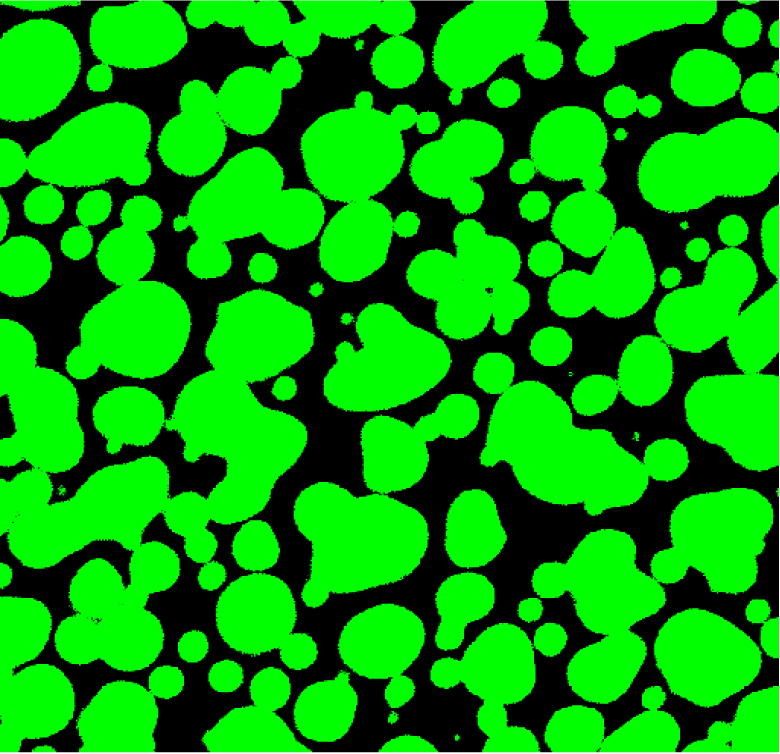

Supplement: Supplementary file 25 — Figure Source Data for Expanded View and Appendix [file 44318_2024_212_MOESM25_ESM.zip › Source Data for Expanded View and Appendix/Figure EV5/5B/20 IRTKS 0 SUMO-HP1 c1-01.tif]

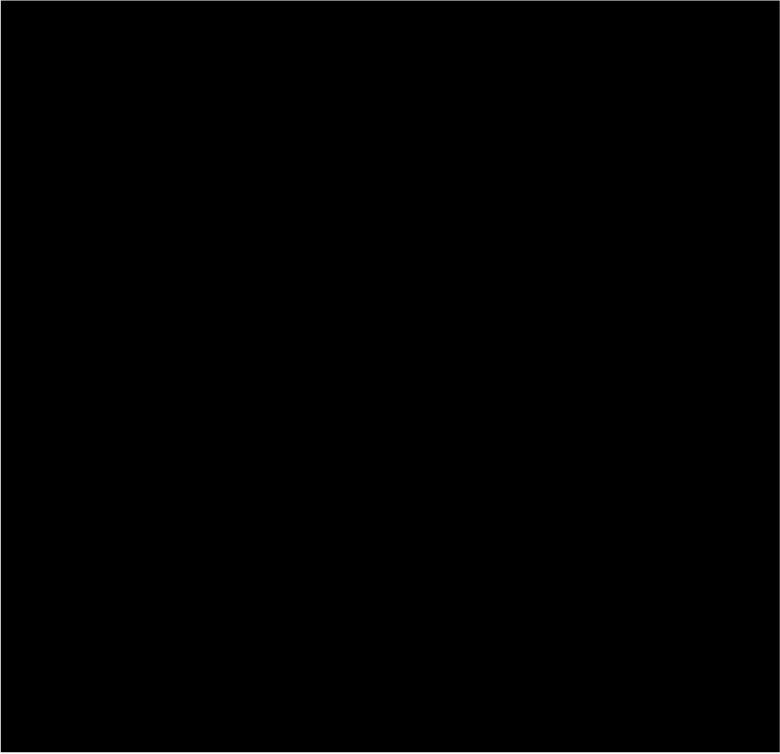

Supplement: Supplementary file 25 — Figure Source Data for Expanded View and Appendix [file 44318_2024_212_MOESM25_ESM.zip › Source Data for Expanded View and Appendix/Figure EV5/5B/20 IRTKS 0 SUMO-HP1 c2-01.tif]

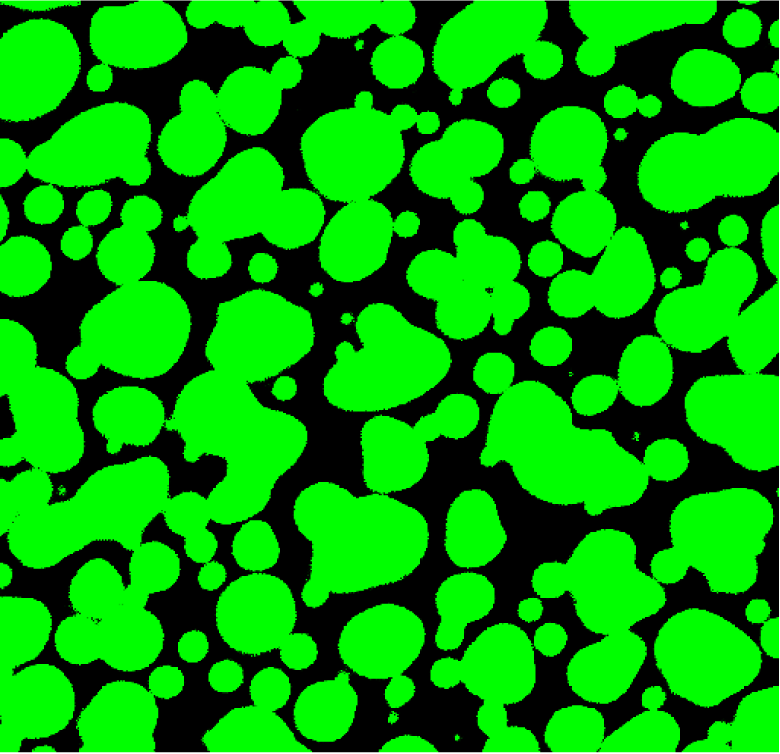

Supplement: Supplementary file 25 — Figure Source Data for Expanded View and Appendix [file 44318_2024_212_MOESM25_ESM.zip › Source Data for Expanded View and Appendix/Figure EV5/5B/20 IRTKS 0 SUMO-HP1-01.tif]

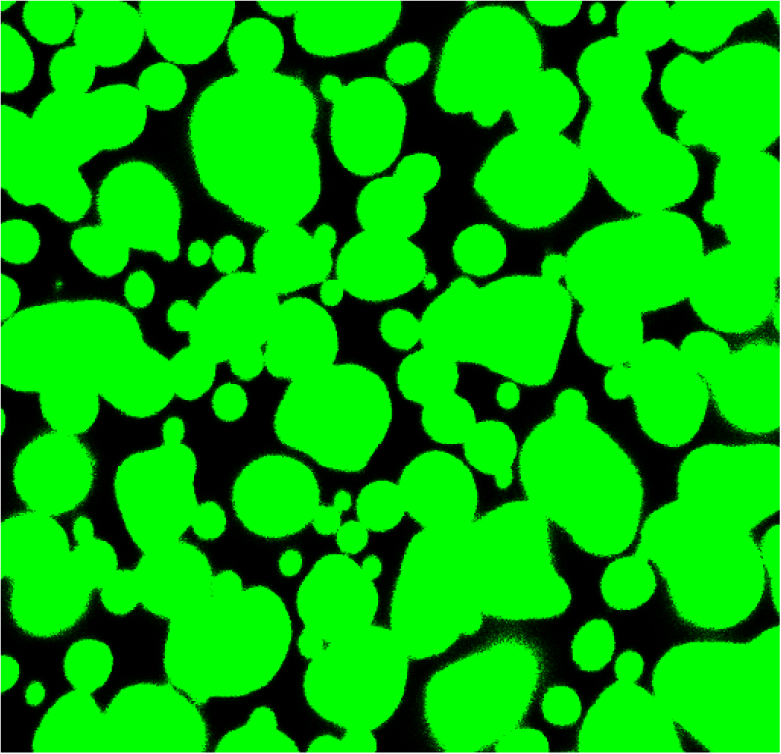

Supplement: Supplementary file 25 — Figure Source Data for Expanded View and Appendix [file 44318_2024_212_MOESM25_ESM.zip › Source Data for Expanded View and Appendix/Figure EV5/5B/20 IRTKS 20 SUMO-HP1 c1-01.tif]

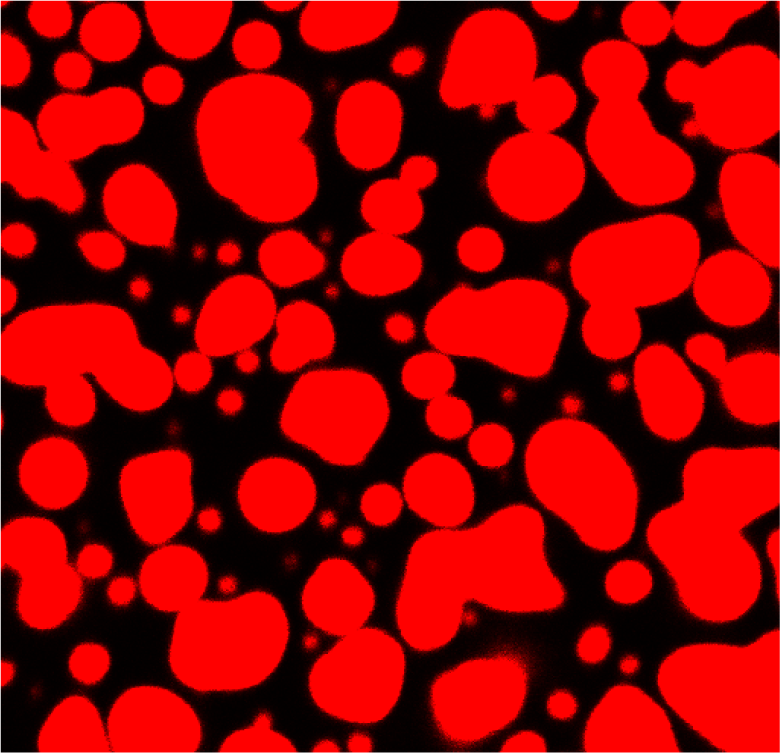

Supplement: Supplementary file 25 — Figure Source Data for Expanded View and Appendix [file 44318_2024_212_MOESM25_ESM.zip › Source Data for Expanded View and Appendix/Figure EV5/5B/20 IRTKS 20 SUMO-HP1 c2-01.tif]

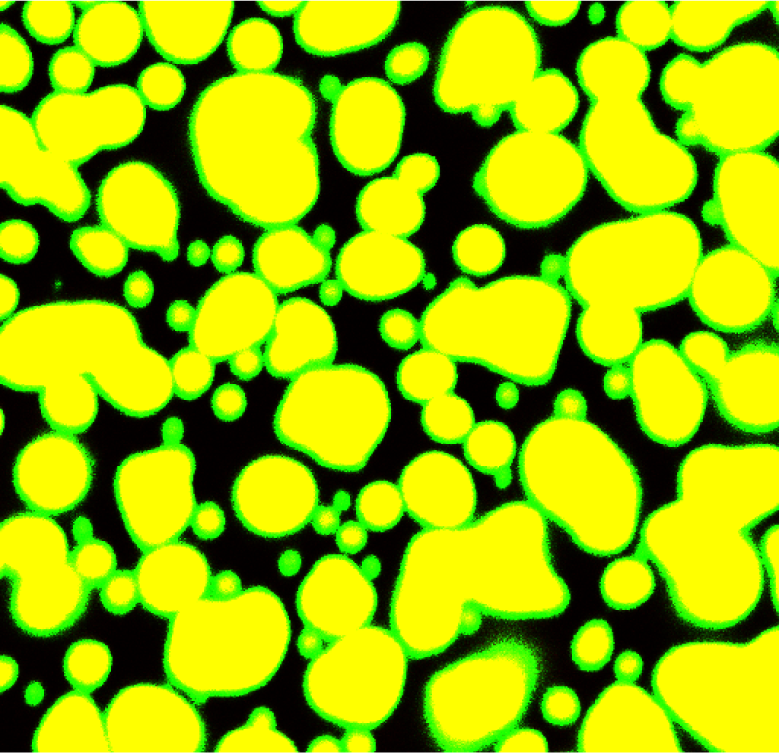

Supplement: Supplementary file 25 — Figure Source Data for Expanded View and Appendix [file 44318_2024_212_MOESM25_ESM.zip › Source Data for Expanded View and Appendix/Figure EV5/5B/20 IRTKS 20 SUMO-HP1-01.tif]

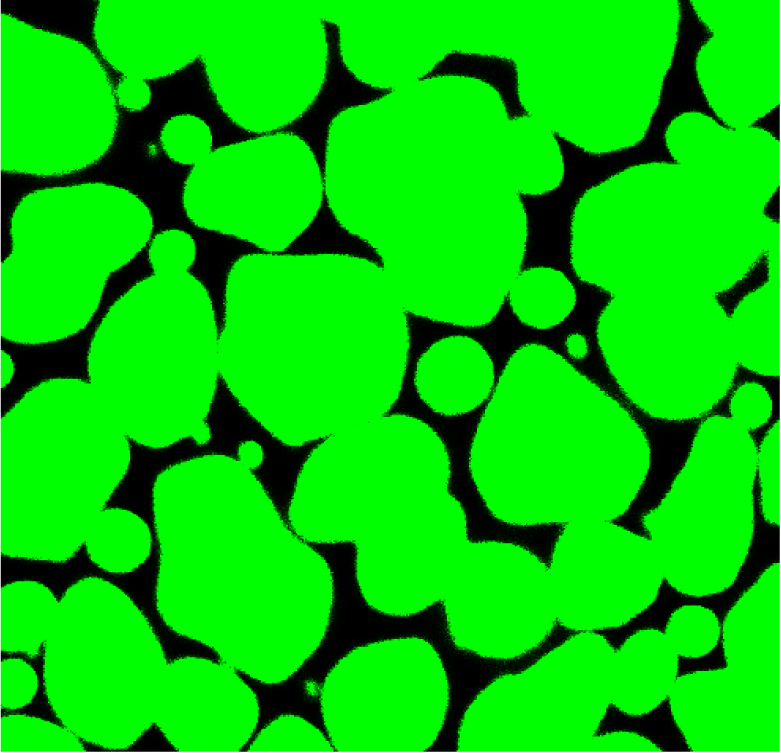

Supplement: Supplementary file 25 — Figure Source Data for Expanded View and Appendix [file 44318_2024_212_MOESM25_ESM.zip › Source Data for Expanded View and Appendix/Figure EV5/5B/20 IRTKS 40 SUMO-HP1 c1-01.tif]

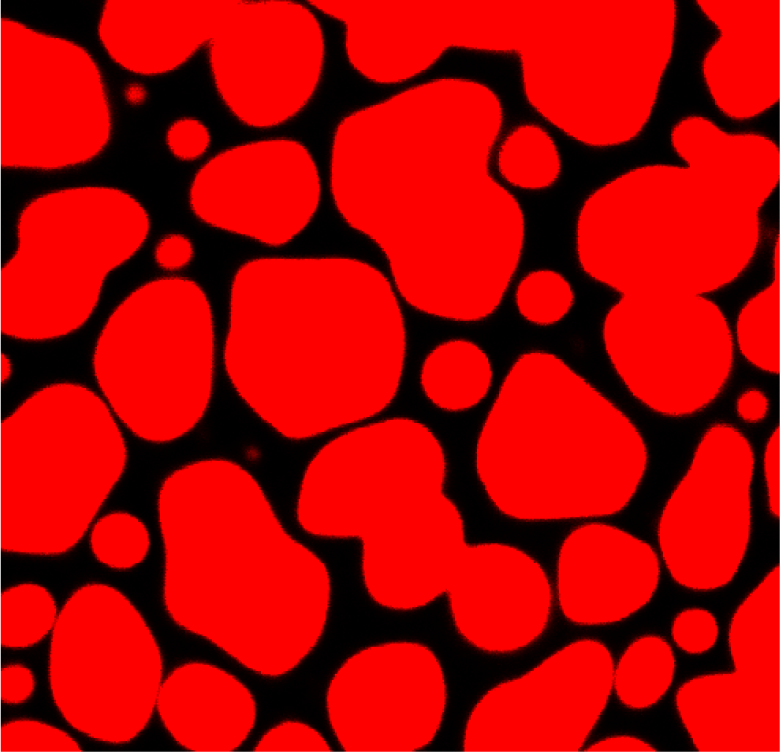

Supplement: Supplementary file 25 — Figure Source Data for Expanded View and Appendix [file 44318_2024_212_MOESM25_ESM.zip › Source Data for Expanded View and Appendix/Figure EV5/5B/20 IRTKS 40 SUMO-HP1 c2-01.tif]

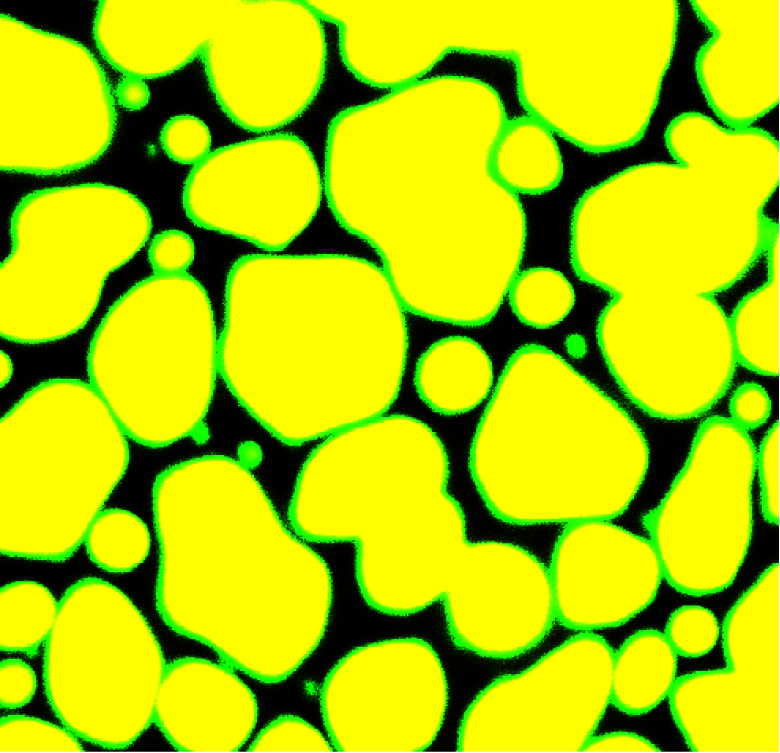

Supplement: Supplementary file 25 — Figure Source Data for Expanded View and Appendix [file 44318_2024_212_MOESM25_ESM.zip › Source Data for Expanded View and Appendix/Figure EV5/5B/20 IRTKS 40 SUMO-HP1-01.tif]

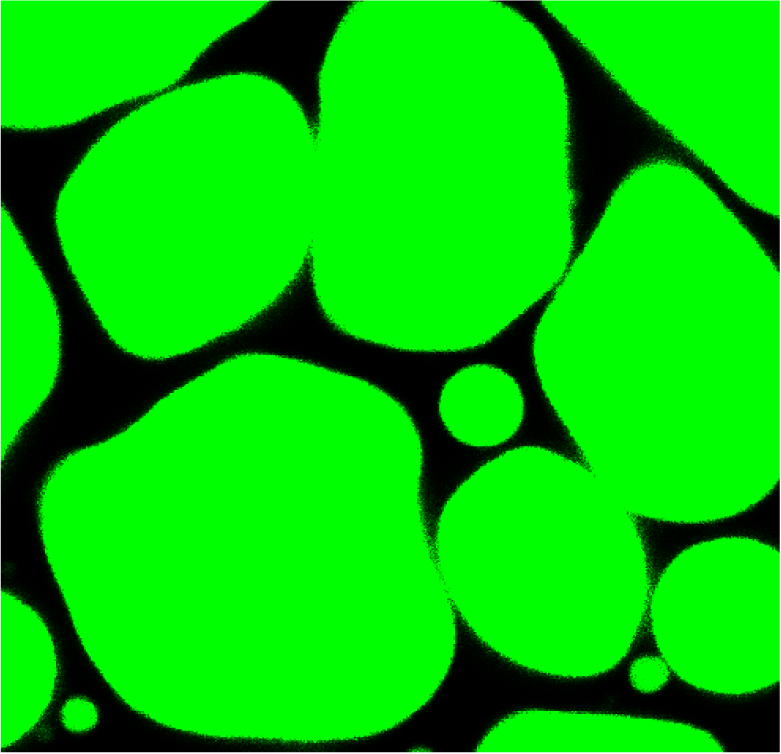

Supplement: Supplementary file 25 — Figure Source Data for Expanded View and Appendix [file 44318_2024_212_MOESM25_ESM.zip › Source Data for Expanded View and Appendix/Figure EV5/5B/20 IRTKS 80 SUMO-HP1 c1-01.tif]

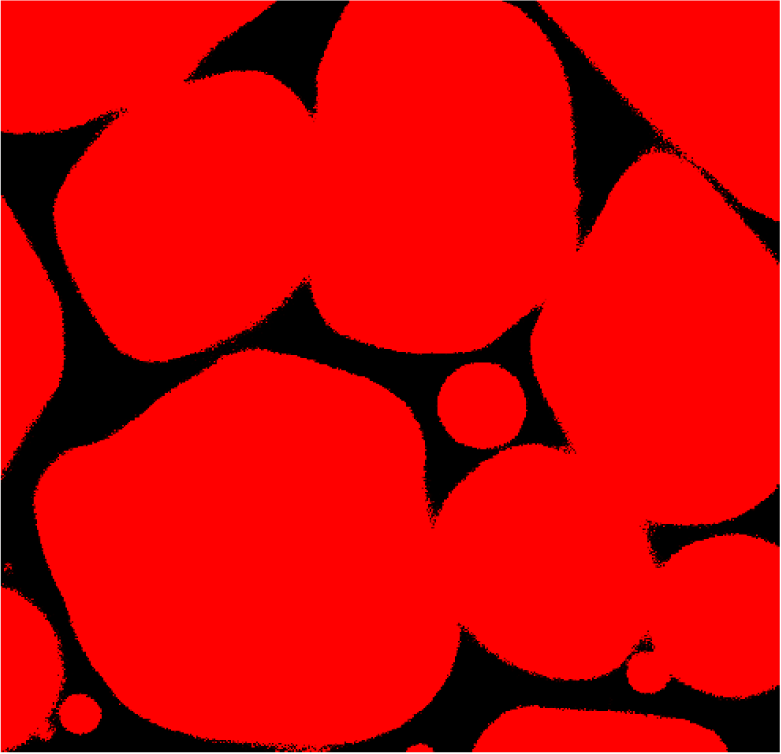

Supplement: Supplementary file 25 — Figure Source Data for Expanded View and Appendix [file 44318_2024_212_MOESM25_ESM.zip › Source Data for Expanded View and Appendix/Figure EV5/5B/20 IRTKS 80 SUMO-HP1 c2-01.tif]

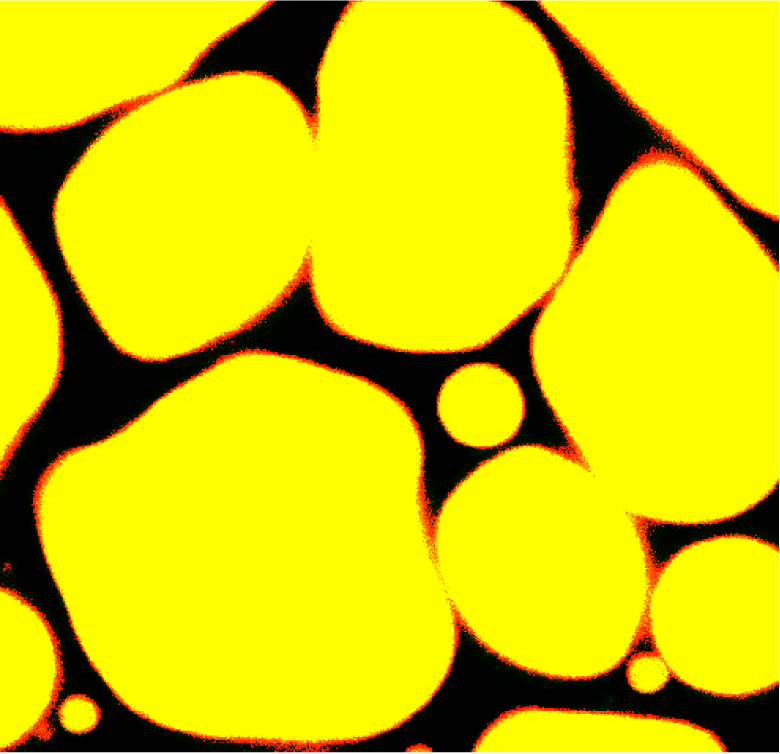

Supplement: Supplementary file 25 — Figure Source Data for Expanded View and Appendix [file 44318_2024_212_MOESM25_ESM.zip › Source Data for Expanded View and Appendix/Figure EV5/5B/20 IRTKS 80 SUMO-HP1-01.tif]

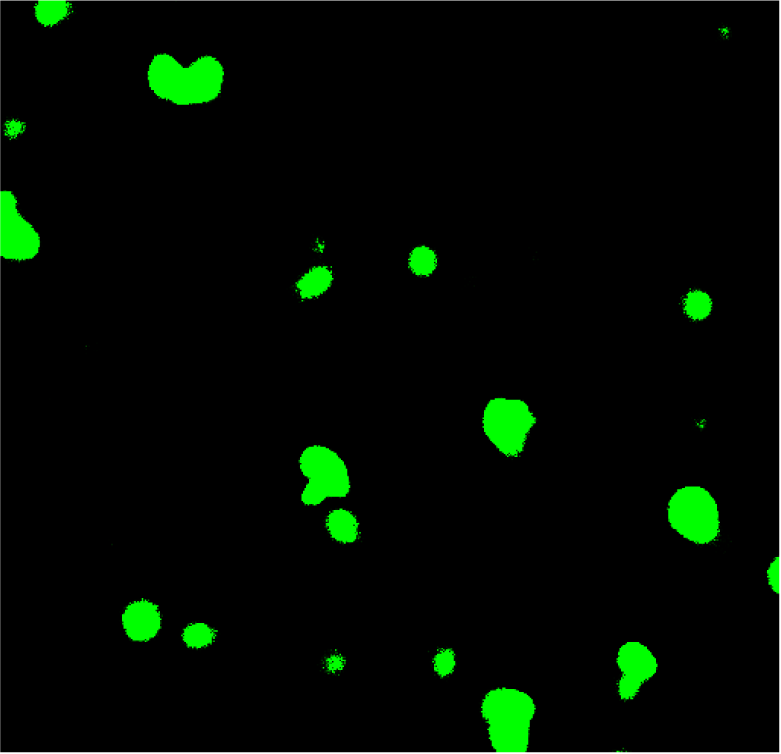

Supplement: Supplementary file 25 — Figure Source Data for Expanded View and Appendix [file 44318_2024_212_MOESM25_ESM.zip › Source Data for Expanded View and Appendix/Figure EV5/5B/5 IRTKS 0 SUMO-HP1 c1-01.tif]

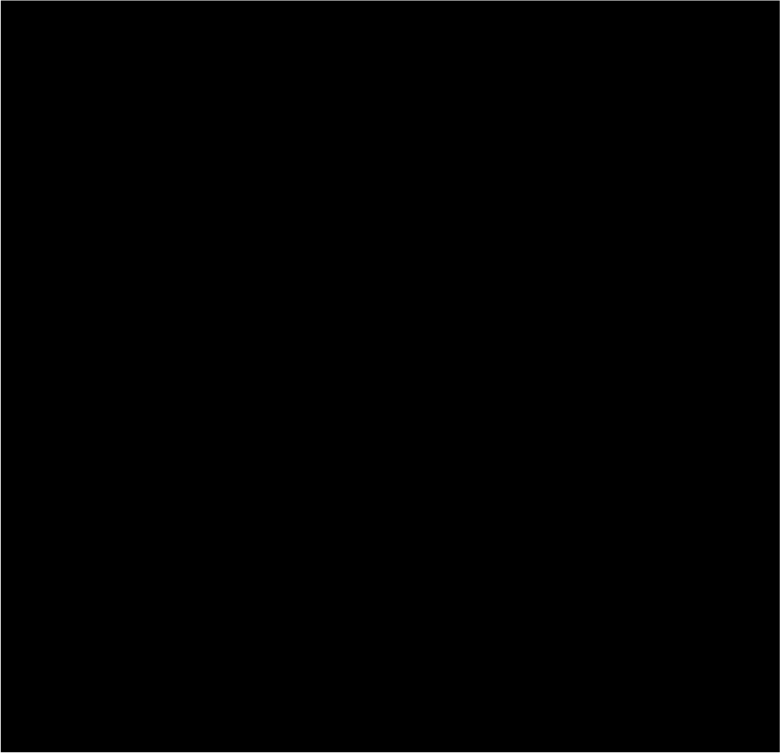

Supplement: Supplementary file 25 — Figure Source Data for Expanded View and Appendix [file 44318_2024_212_MOESM25_ESM.zip › Source Data for Expanded View and Appendix/Figure EV5/5B/5 IRTKS 0 SUMO-HP1 c2-01.tif]

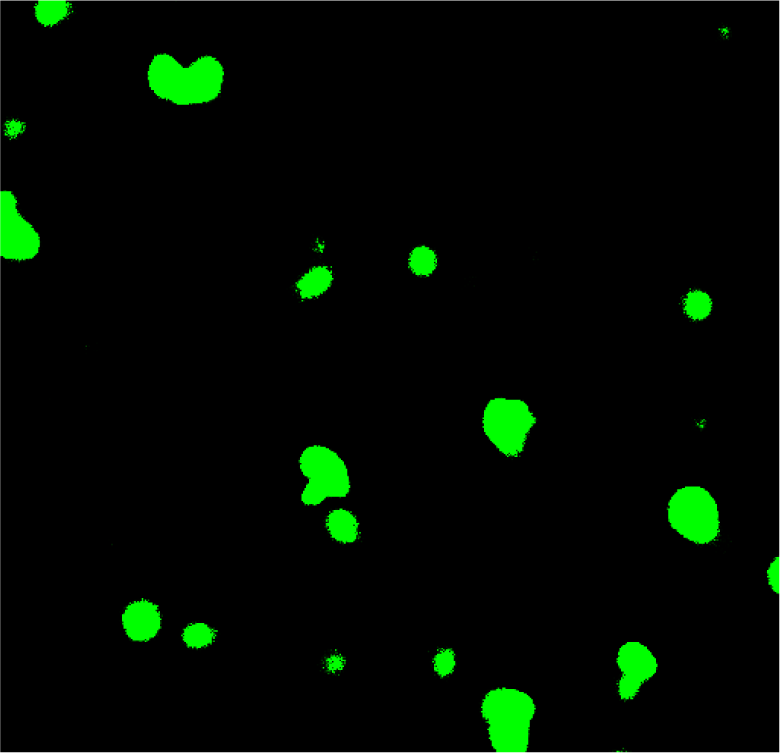

Supplement: Supplementary file 25 — Figure Source Data for Expanded View and Appendix [file 44318_2024_212_MOESM25_ESM.zip › Source Data for Expanded View and Appendix/Figure EV5/5B/5 IRTKS 0 SUMO-HP1-01.tif]

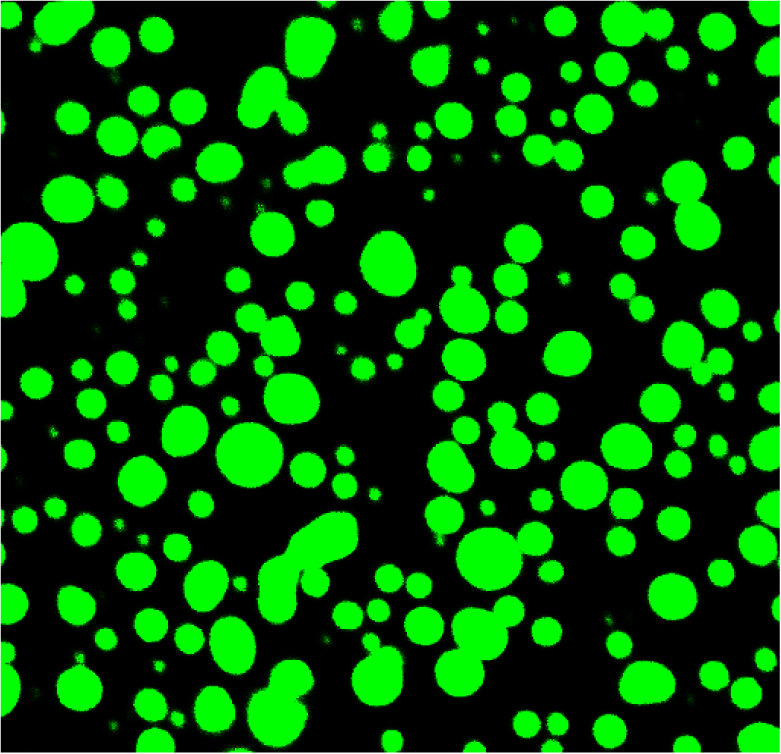

Supplement: Supplementary file 25 — Figure Source Data for Expanded View and Appendix [file 44318_2024_212_MOESM25_ESM.zip › Source Data for Expanded View and Appendix/Figure EV5/5B/5 IRTKS 20 SUMO-HP1 c1-01.tif]

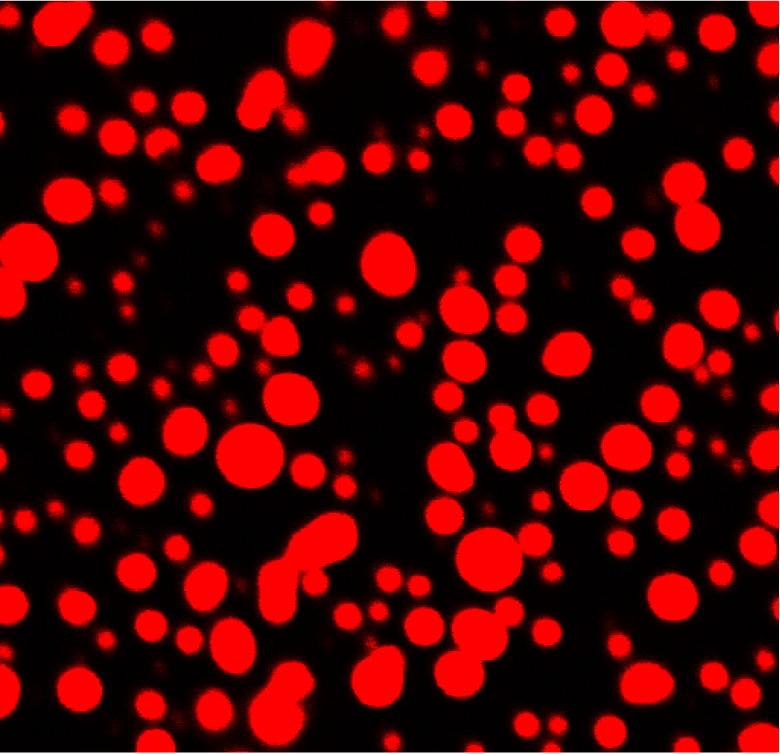

Supplement: Supplementary file 25 — Figure Source Data for Expanded View and Appendix [file 44318_2024_212_MOESM25_ESM.zip › Source Data for Expanded View and Appendix/Figure EV5/5B/5 IRTKS 20 SUMO-HP1 c2-01.tif]

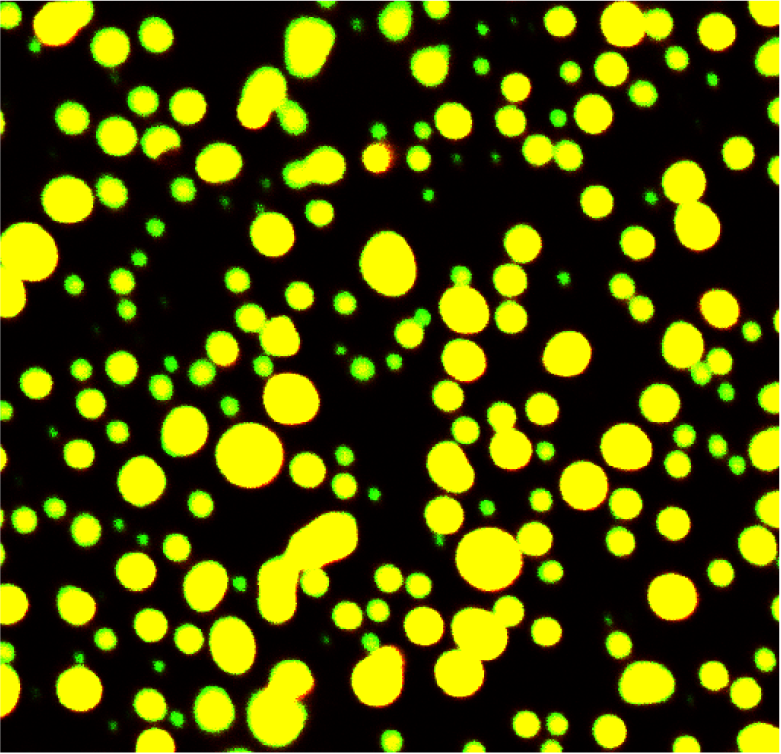

Supplement: Supplementary file 25 — Figure Source Data for Expanded View and Appendix [file 44318_2024_212_MOESM25_ESM.zip › Source Data for Expanded View and Appendix/Figure EV5/5B/5 IRTKS 20 SUMO-HP1-01.tif]

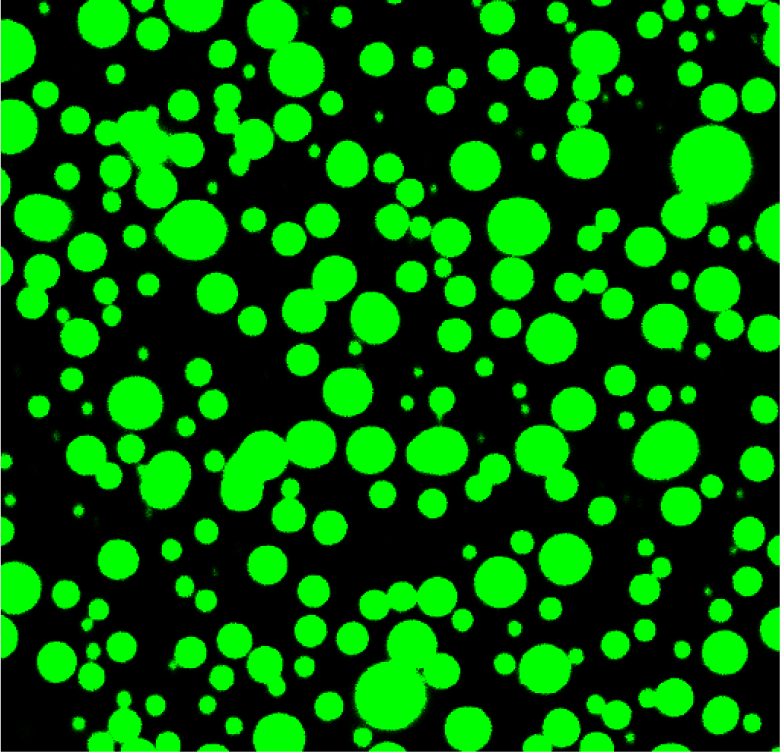

Supplement: Supplementary file 25 — Figure Source Data for Expanded View and Appendix [file 44318_2024_212_MOESM25_ESM.zip › Source Data for Expanded View and Appendix/Figure EV5/5B/5 IRTKS 40 SUMO-HP1 c1-01.tif]

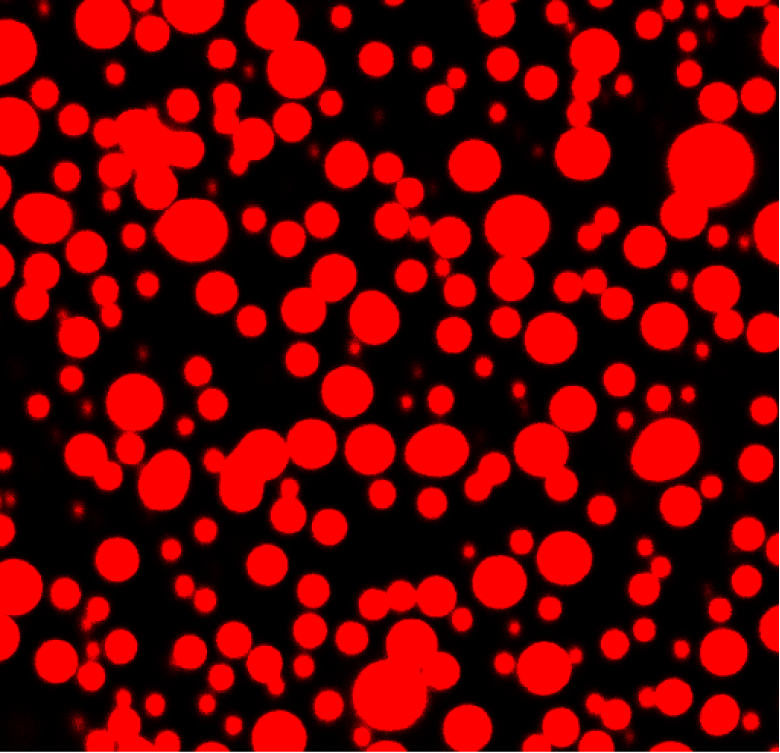

Supplement: Supplementary file 25 — Figure Source Data for Expanded View and Appendix [file 44318_2024_212_MOESM25_ESM.zip › Source Data for Expanded View and Appendix/Figure EV5/5B/5 IRTKS 40 SUMO-HP1 c2-01.tif]

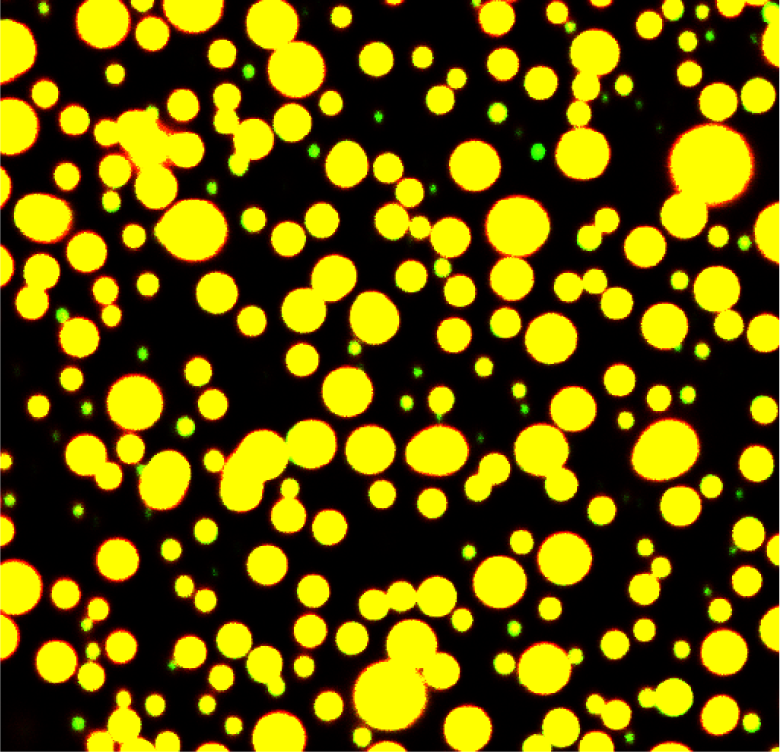

Supplement: Supplementary file 25 — Figure Source Data for Expanded View and Appendix [file 44318_2024_212_MOESM25_ESM.zip › Source Data for Expanded View and Appendix/Figure EV5/5B/5 IRTKS 40 SUMO-HP1-01.tif]

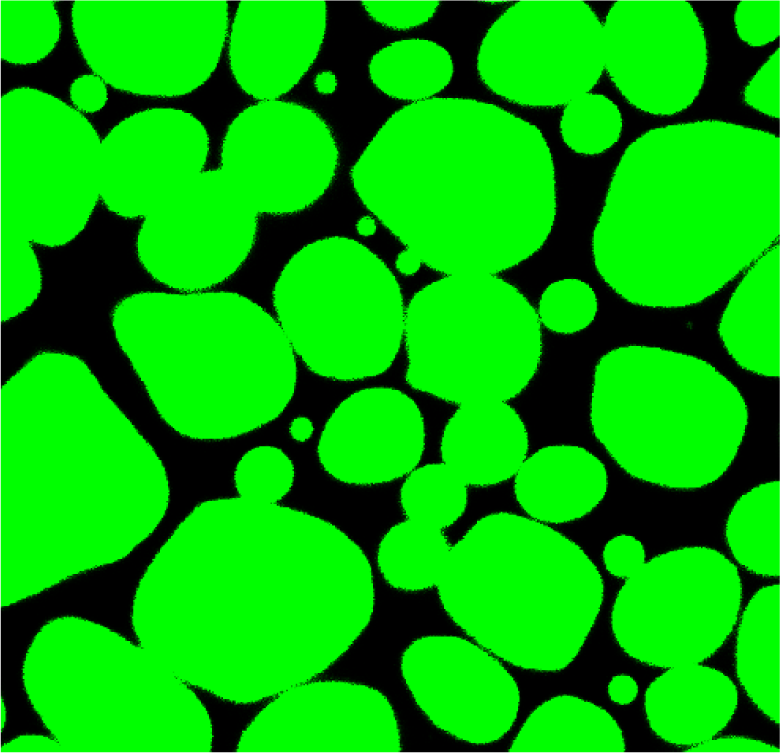

Supplement: Supplementary file 25 — Figure Source Data for Expanded View and Appendix [file 44318_2024_212_MOESM25_ESM.zip › Source Data for Expanded View and Appendix/Figure EV5/5B/5 IRTKS 80 SUMO-HP1 c1-01.tif]
