# Supplementary figures and images for: Heterochromatin formation and remodeling by IRTKS condensates counteract cellular senescence (part 3 of 4)
Source: EMBO J. 2024 Aug 27;43(20):7. doi: 10.1038/s44318-024-00212-3 (PMC11480336; doi:10.1038/s44318-024-00212-3)

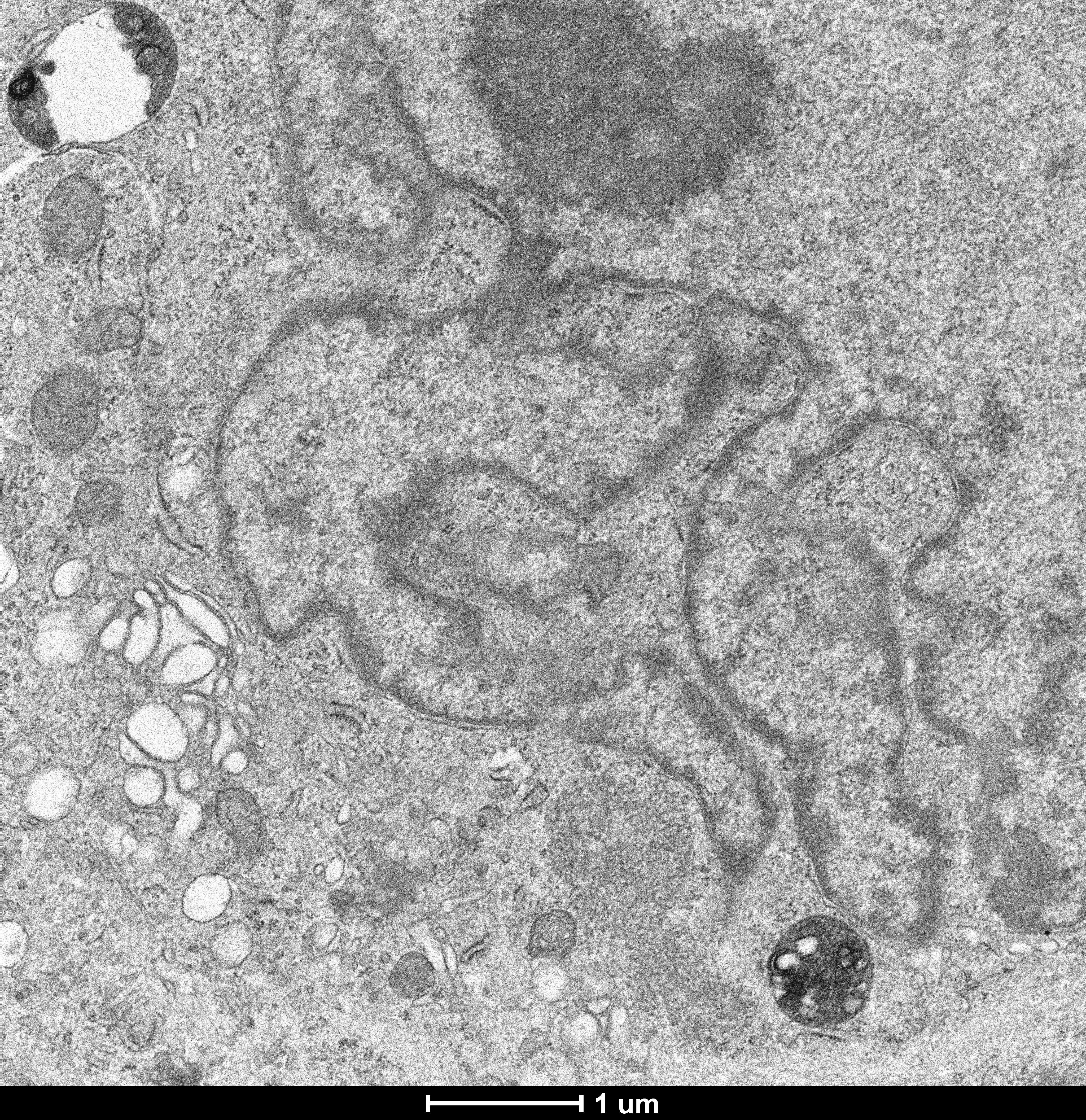

Supplement: Supplementary file 25 — Figure Source Data for Expanded View and Appendix [file 44318_2024_212_MOESM25_ESM.zip › Source Data for Expanded View and Appendix/Figure EV1/1J/Flag-IRTKS-enlarged.jpg]

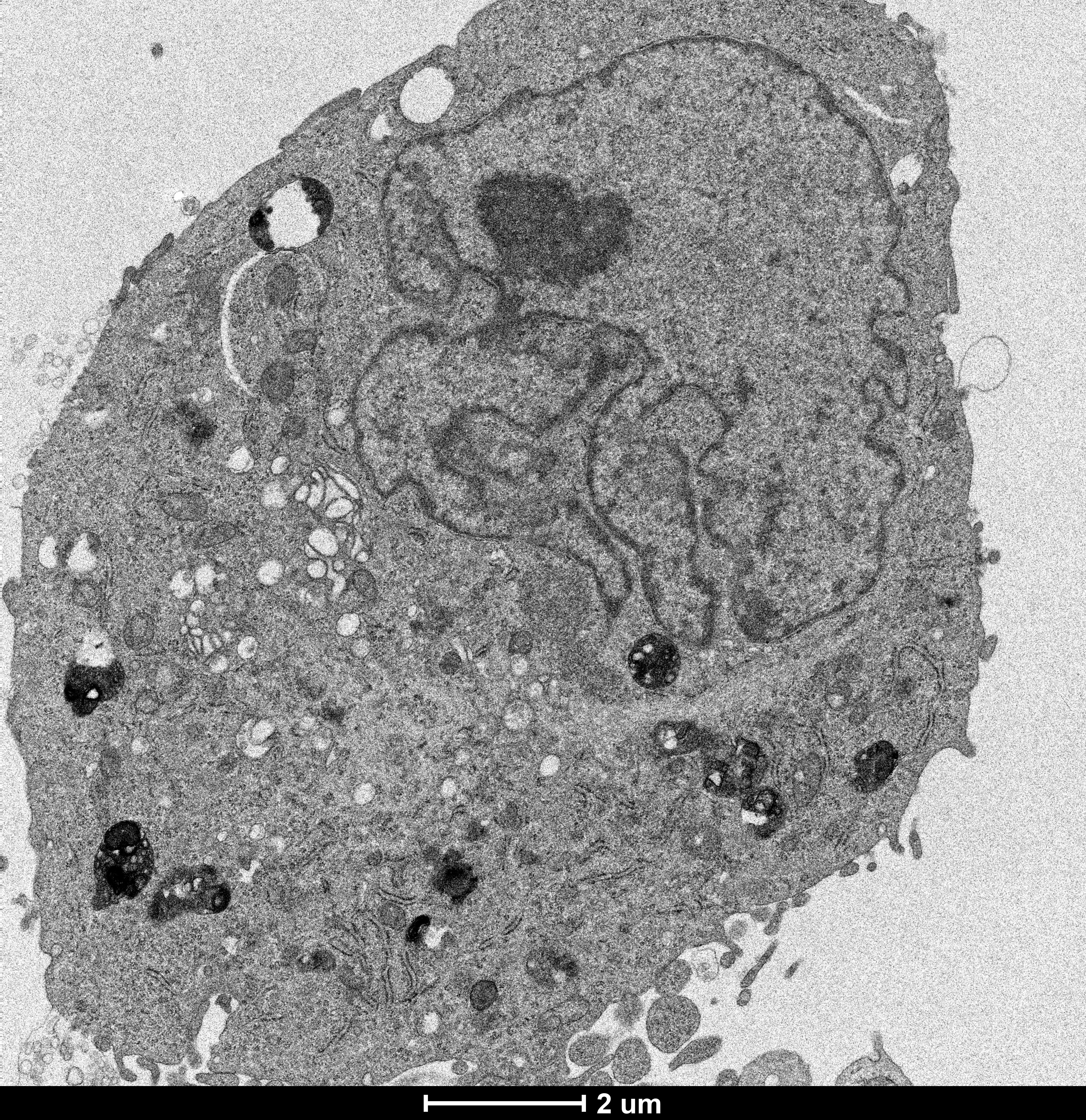

Supplement: Supplementary file 25 — Figure Source Data for Expanded View and Appendix [file 44318_2024_212_MOESM25_ESM.zip › Source Data for Expanded View and Appendix/Figure EV1/1J/Flag-IRTKS.jpg]

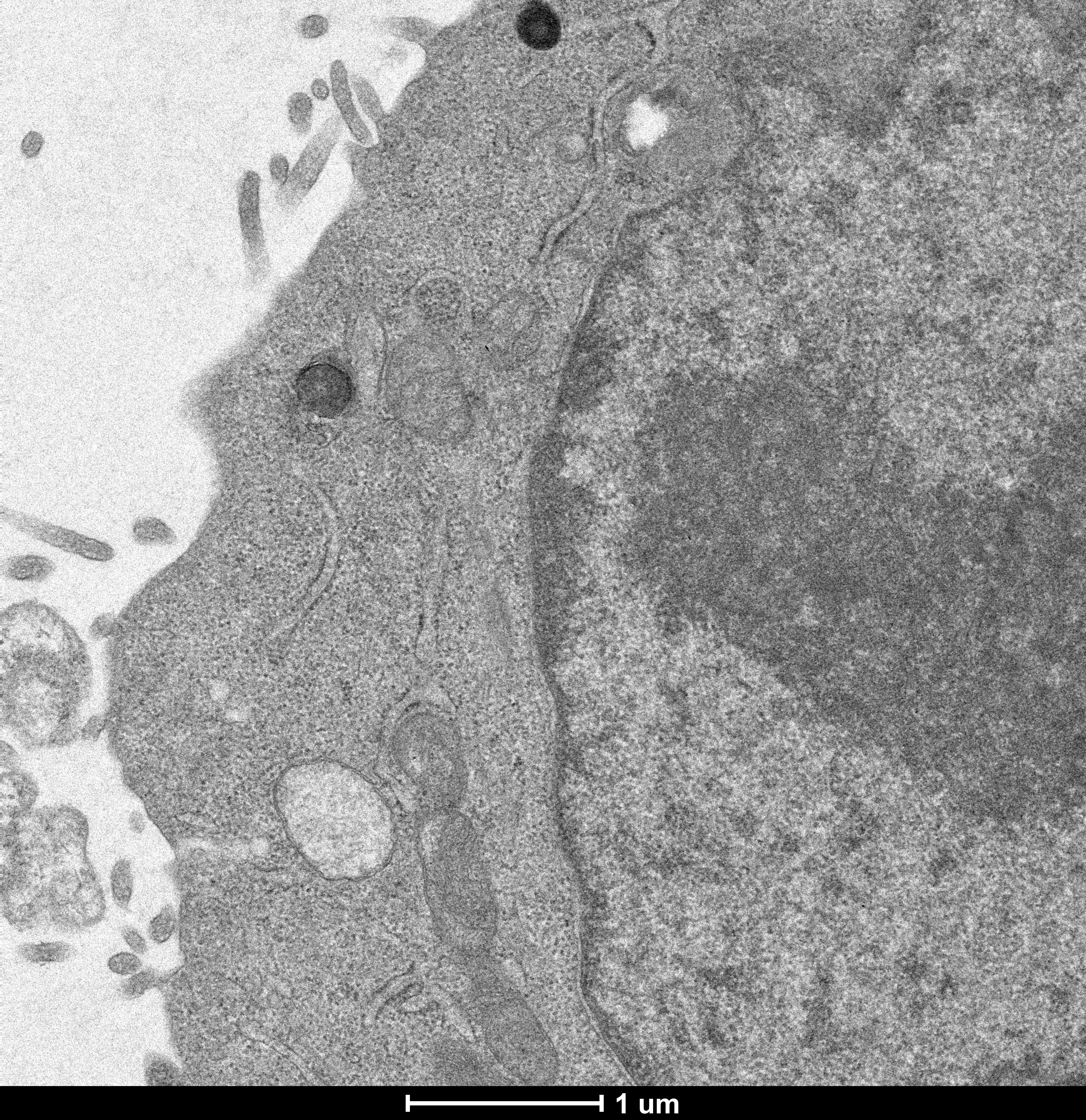

Supplement: Supplementary file 25 — Figure Source Data for Expanded View and Appendix [file 44318_2024_212_MOESM25_ESM.zip › Source Data for Expanded View and Appendix/Figure EV1/1M/E.V.-enlarged.jpg]

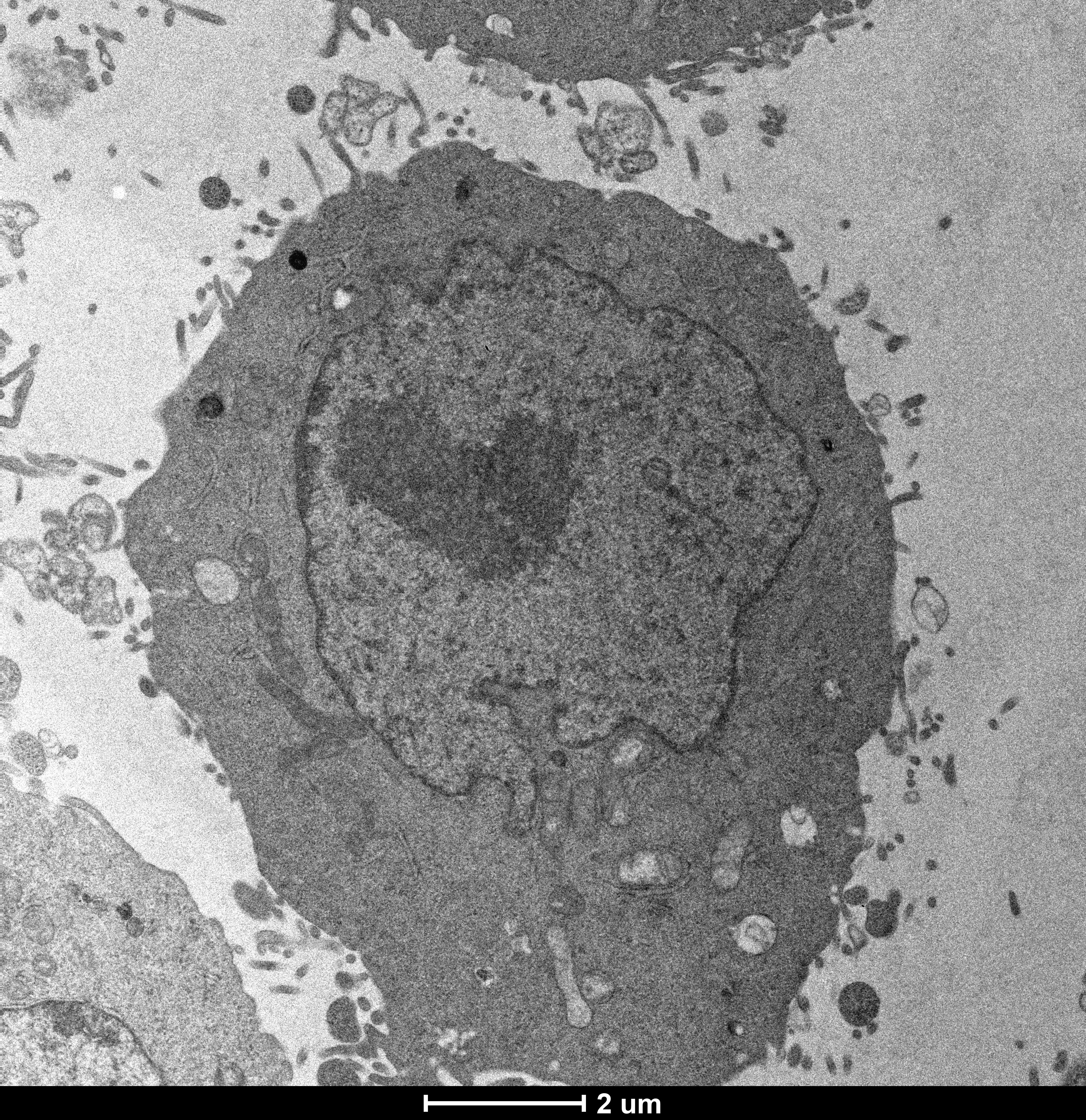

Supplement: Supplementary file 25 — Figure Source Data for Expanded View and Appendix [file 44318_2024_212_MOESM25_ESM.zip › Source Data for Expanded View and Appendix/Figure EV1/1M/E.V..jpg]

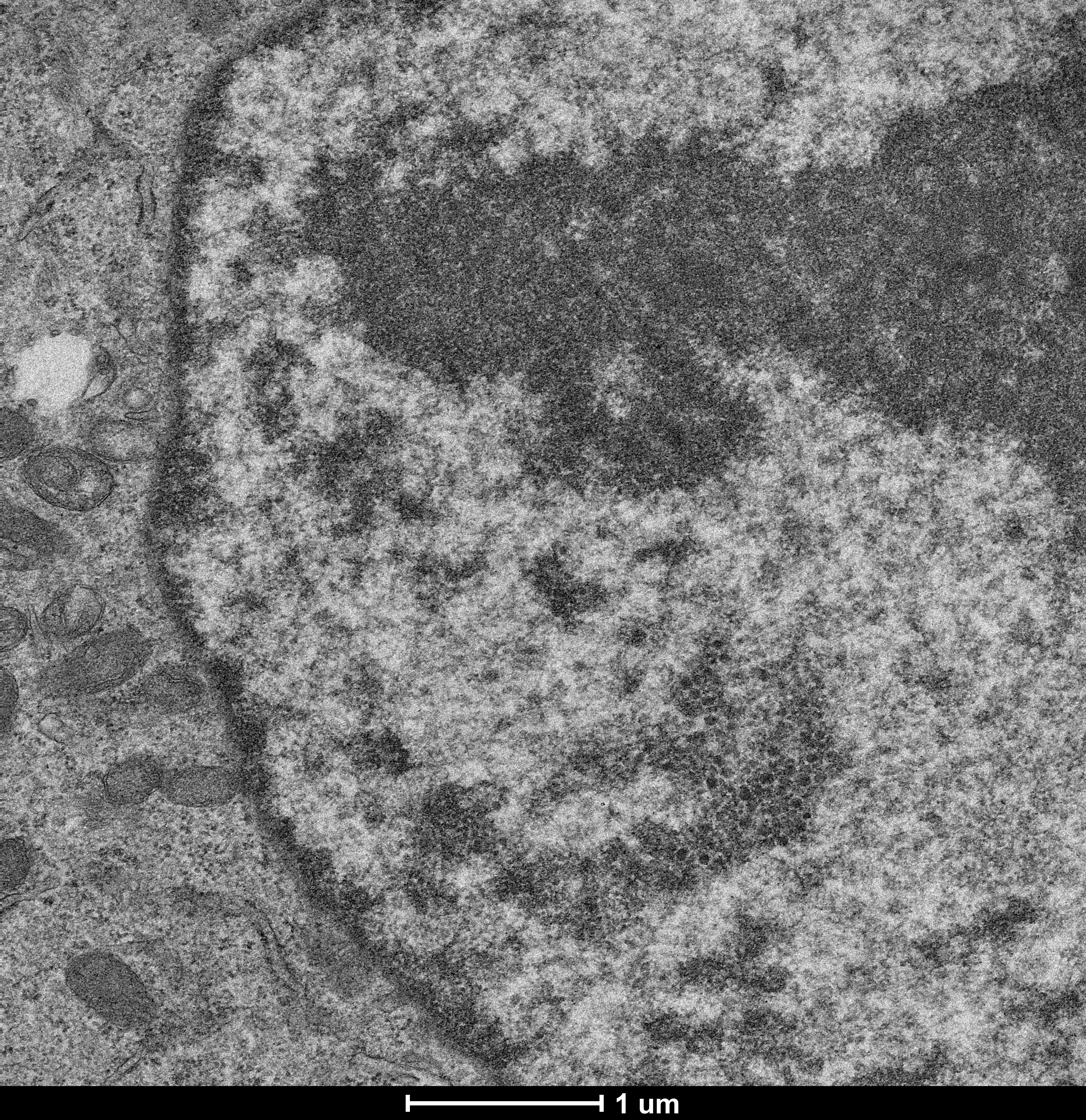

Supplement: Supplementary file 25 — Figure Source Data for Expanded View and Appendix [file 44318_2024_212_MOESM25_ESM.zip › Source Data for Expanded View and Appendix/Figure EV1/1M/Flag-IRTKS-enlarged.jpg]

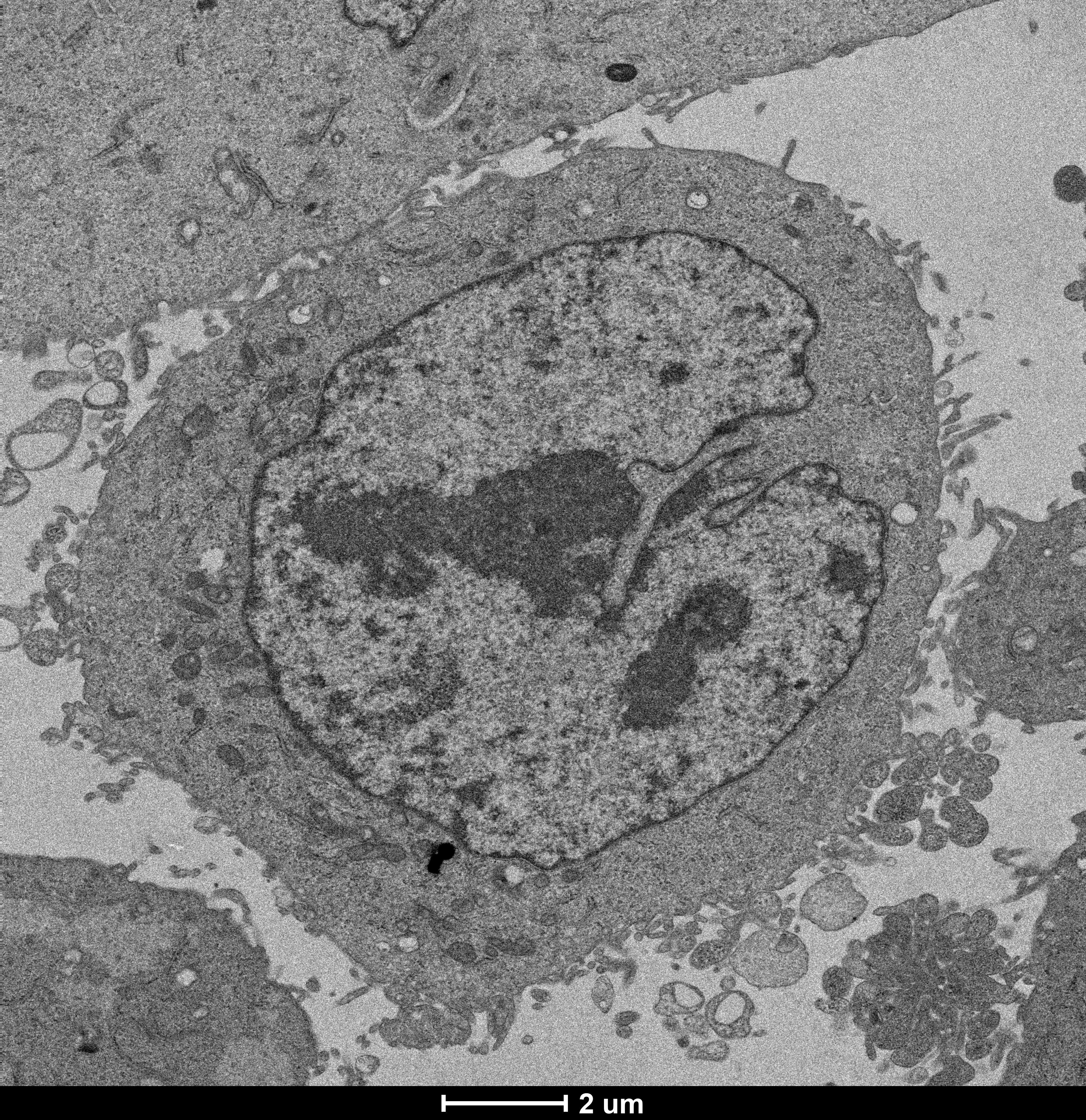

Supplement: Supplementary file 25 — Figure Source Data for Expanded View and Appendix [file 44318_2024_212_MOESM25_ESM.zip › Source Data for Expanded View and Appendix/Figure EV1/1M/Flag-IRTKS.jpg]

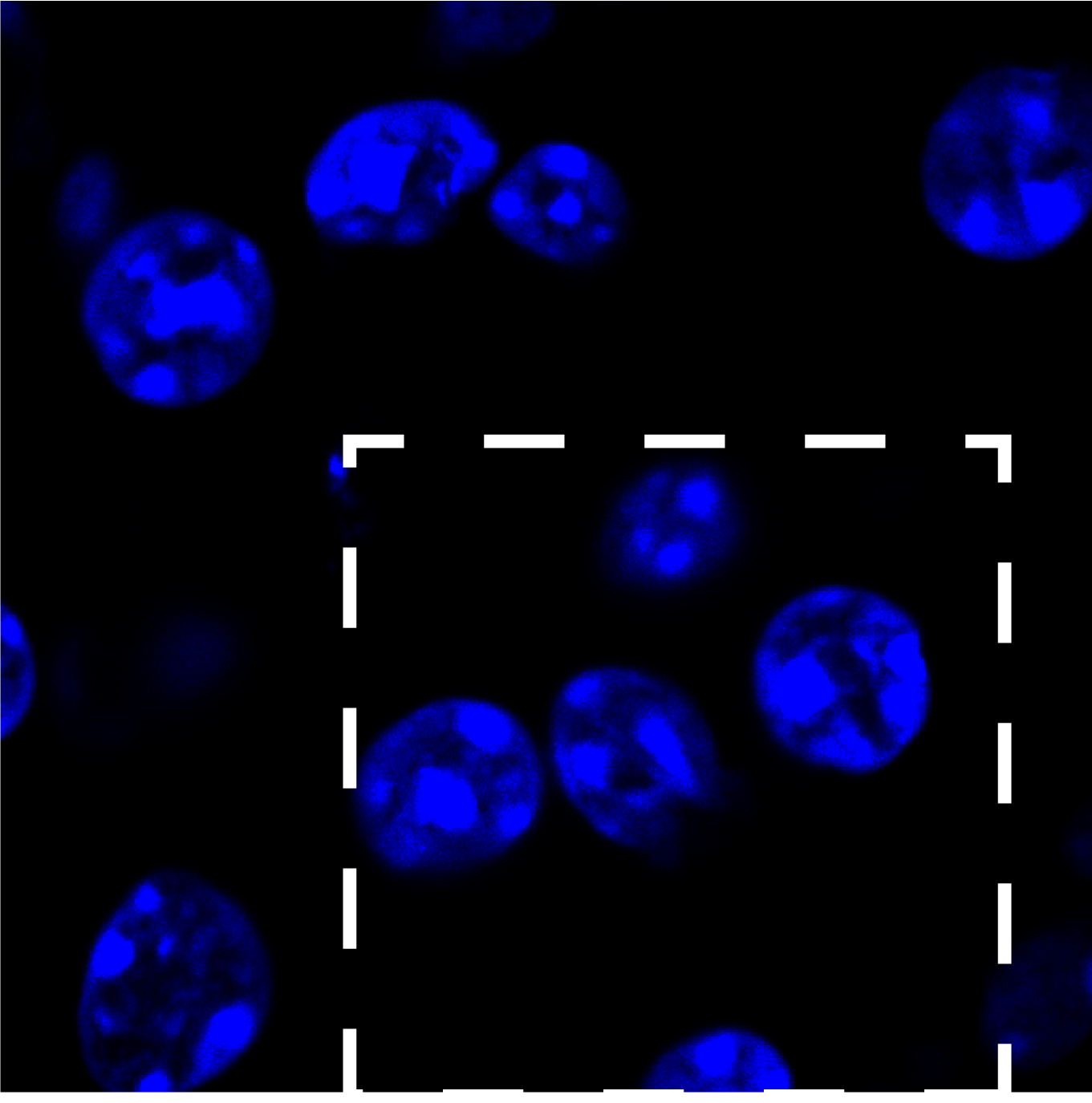

Supplement: Supplementary file 25 — Figure Source Data for Expanded View and Appendix [file 44318_2024_212_MOESM25_ESM.zip › Source Data for Expanded View and Appendix/Figure EV1/1P/KO-DAPI.tif]

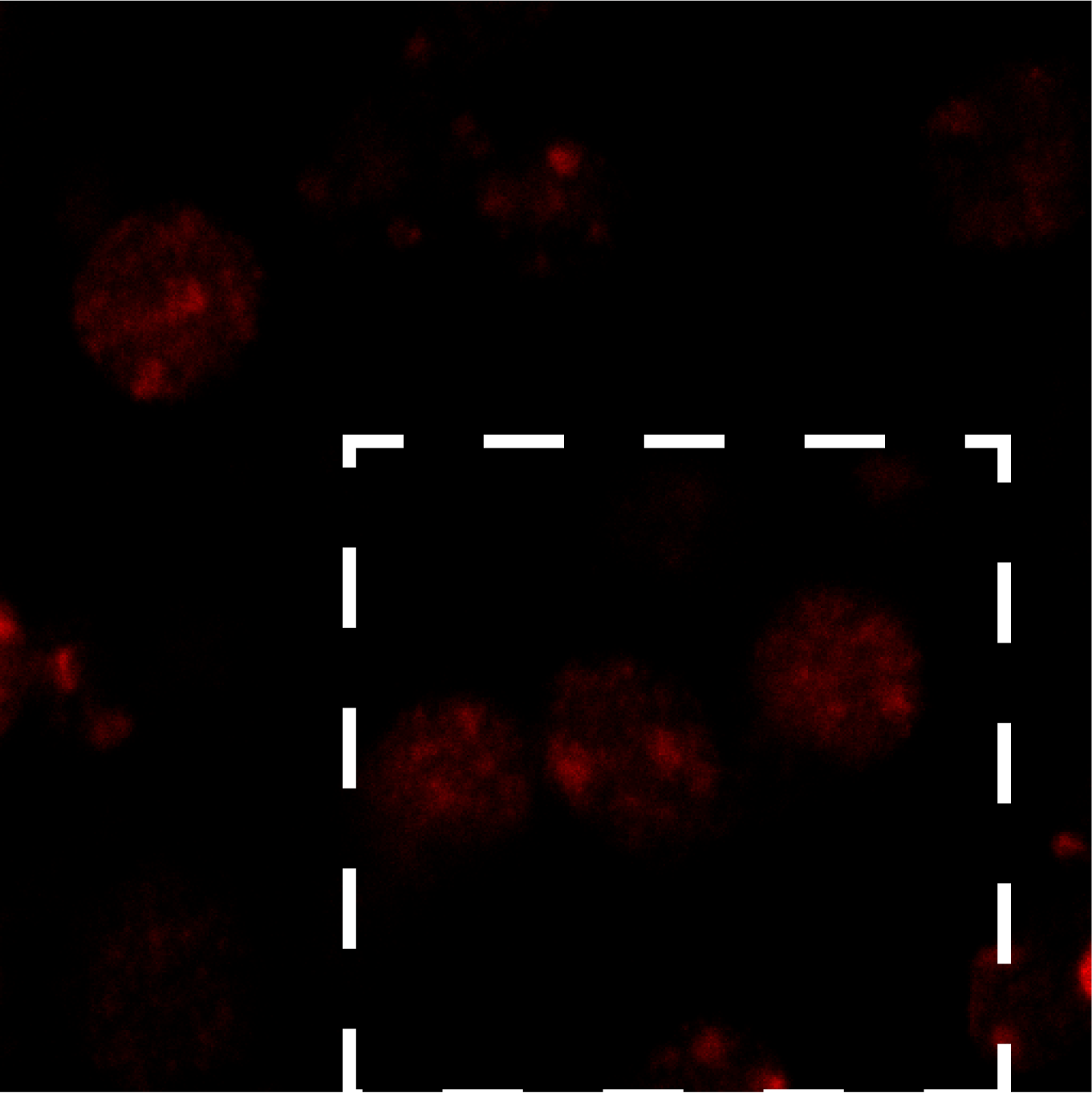

Supplement: Supplementary file 25 — Figure Source Data for Expanded View and Appendix [file 44318_2024_212_MOESM25_ESM.zip › Source Data for Expanded View and Appendix/Figure EV1/1P/KO-HP1α.tif]

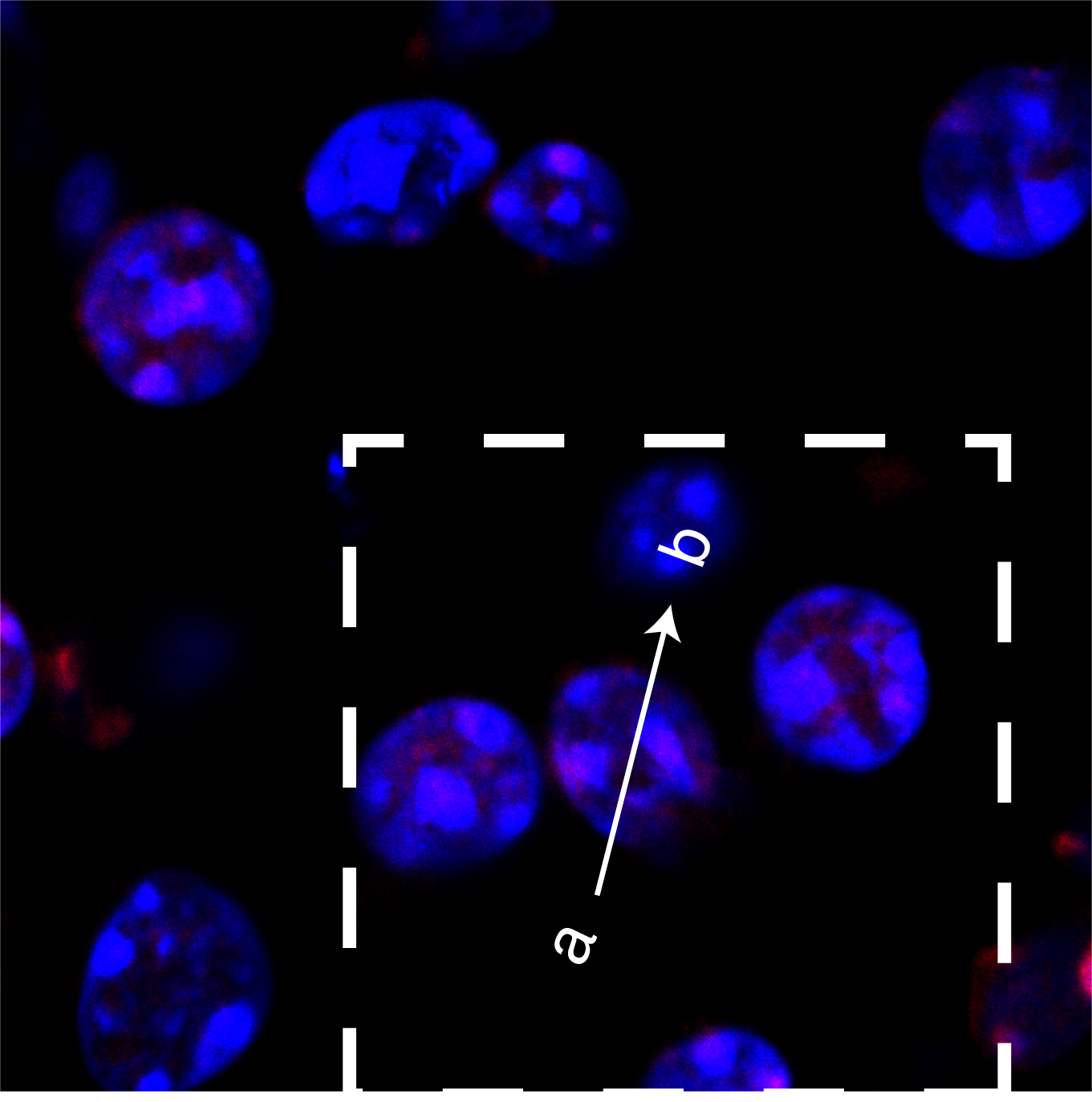

Supplement: Supplementary file 25 — Figure Source Data for Expanded View and Appendix [file 44318_2024_212_MOESM25_ESM.zip › Source Data for Expanded View and Appendix/Figure EV1/1P/KO-Merge.tif]

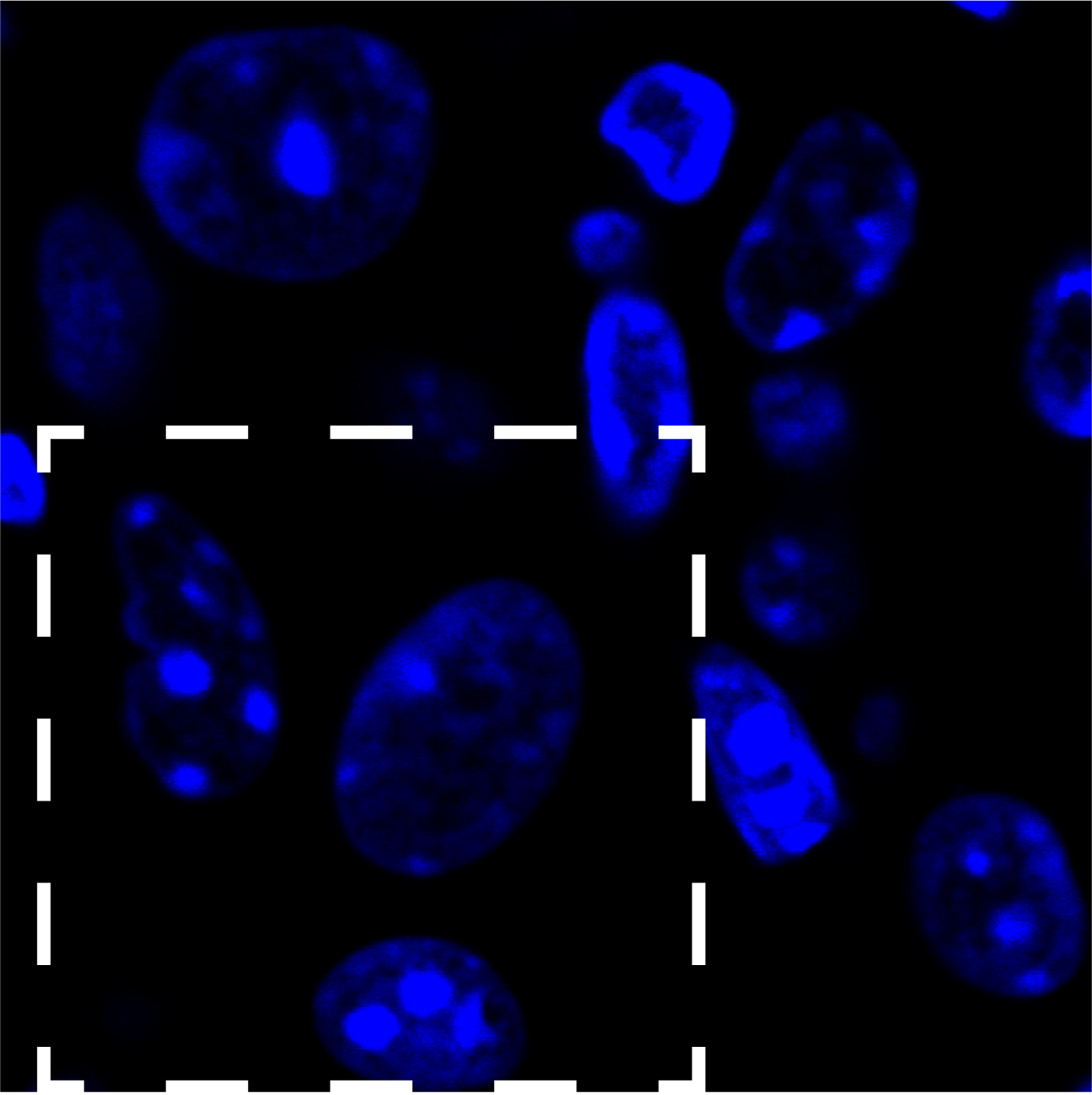

Supplement: Supplementary file 25 — Figure Source Data for Expanded View and Appendix [file 44318_2024_212_MOESM25_ESM.zip › Source Data for Expanded View and Appendix/Figure EV1/1P/WT-DAPI.tif]

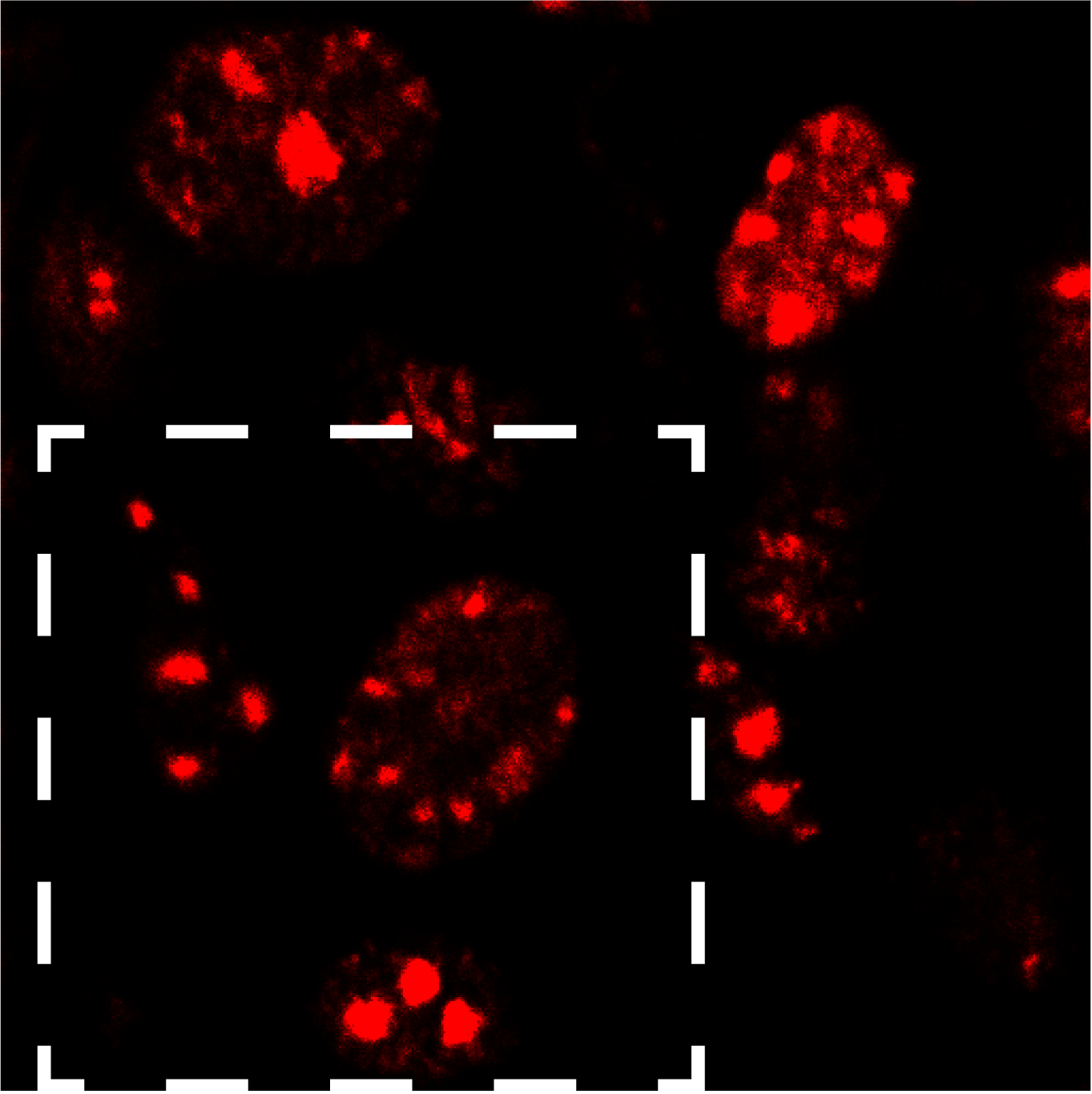

Supplement: Supplementary file 25 — Figure Source Data for Expanded View and Appendix [file 44318_2024_212_MOESM25_ESM.zip › Source Data for Expanded View and Appendix/Figure EV1/1P/WT-HP1α.tif]

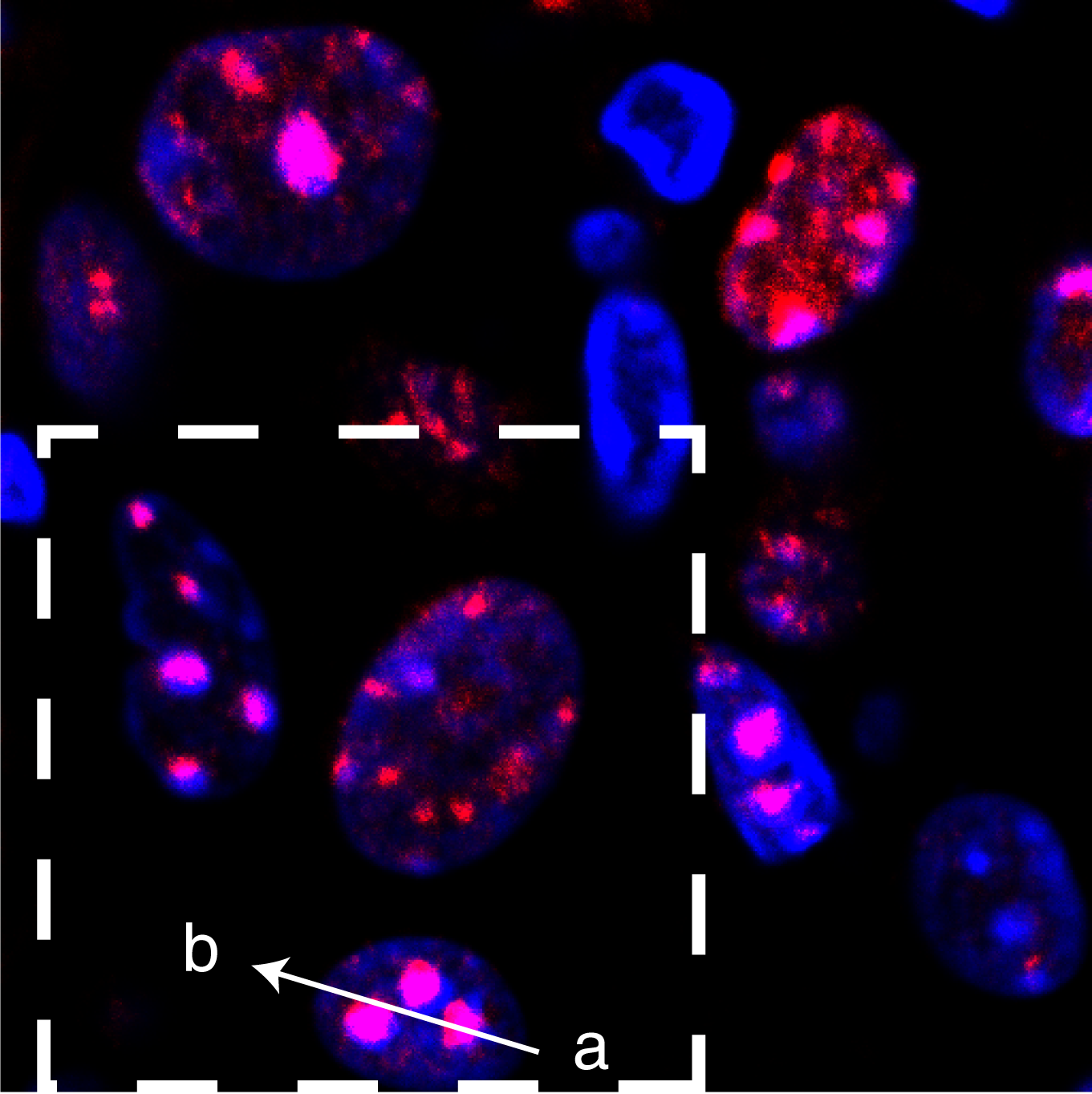

Supplement: Supplementary file 25 — Figure Source Data for Expanded View and Appendix [file 44318_2024_212_MOESM25_ESM.zip › Source Data for Expanded View and Appendix/Figure EV1/1P/WT-Merge.tif]

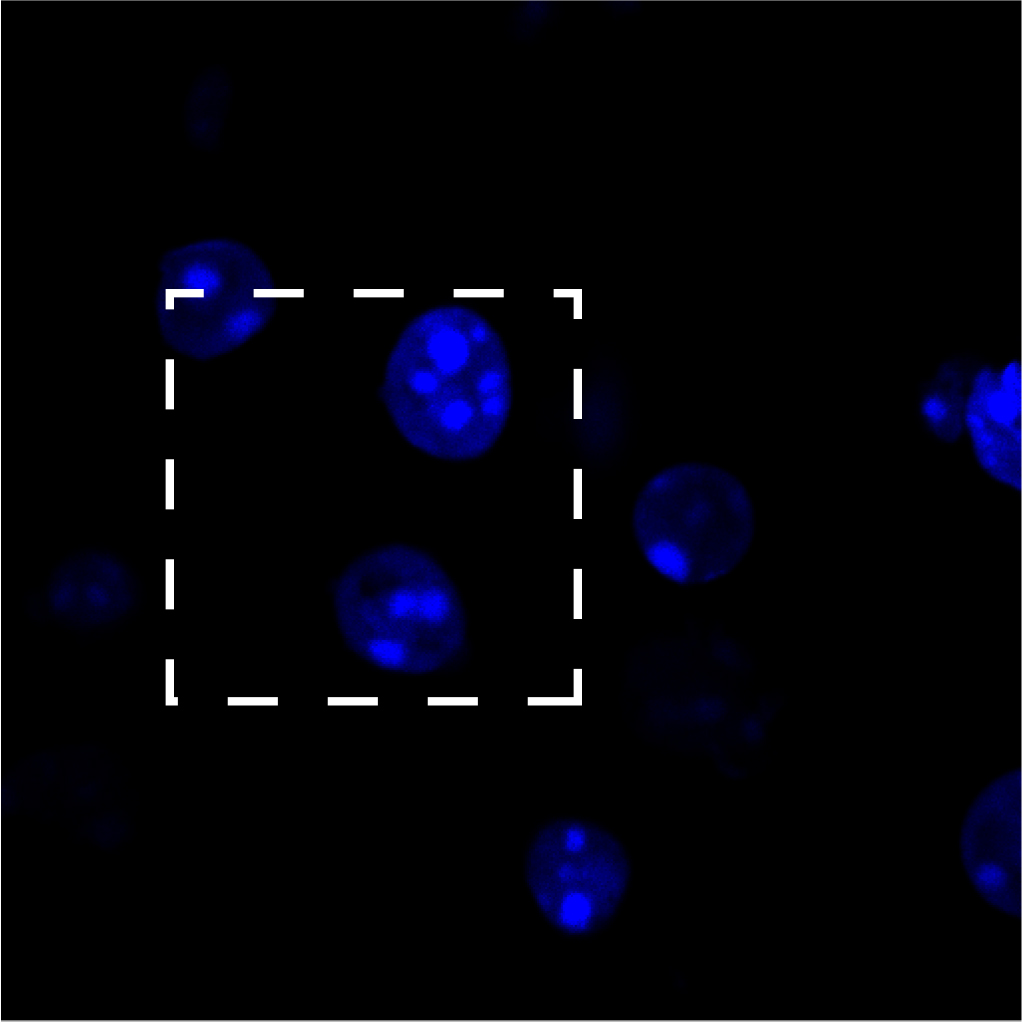

Supplement: Supplementary file 25 — Figure Source Data for Expanded View and Appendix [file 44318_2024_212_MOESM25_ESM.zip › Source Data for Expanded View and Appendix/Figure EV1/1T/KO-DAPI.jpg]

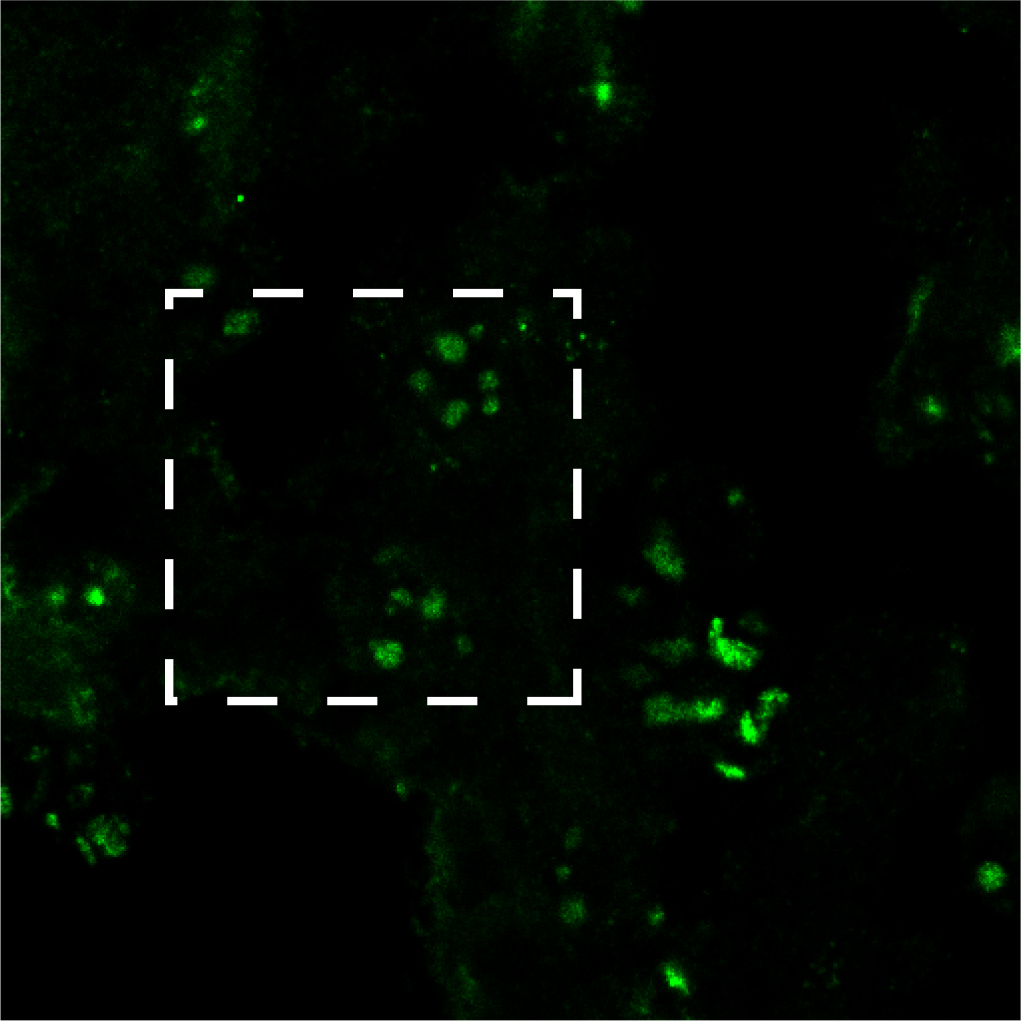

Supplement: Supplementary file 25 — Figure Source Data for Expanded View and Appendix [file 44318_2024_212_MOESM25_ESM.zip › Source Data for Expanded View and Appendix/Figure EV1/1T/KO-H3K9me3.jpg]

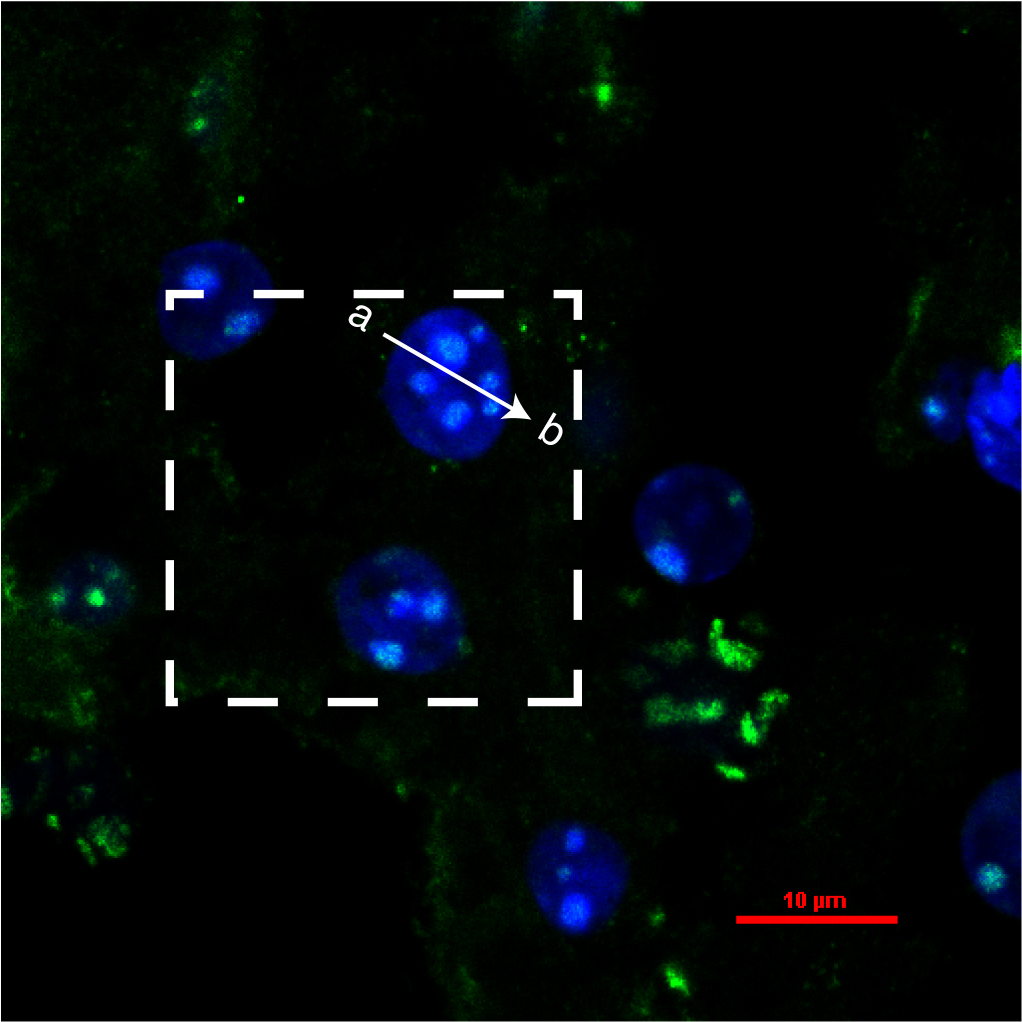

Supplement: Supplementary file 25 — Figure Source Data for Expanded View and Appendix [file 44318_2024_212_MOESM25_ESM.zip › Source Data for Expanded View and Appendix/Figure EV1/1T/KO-Merge.jpg]

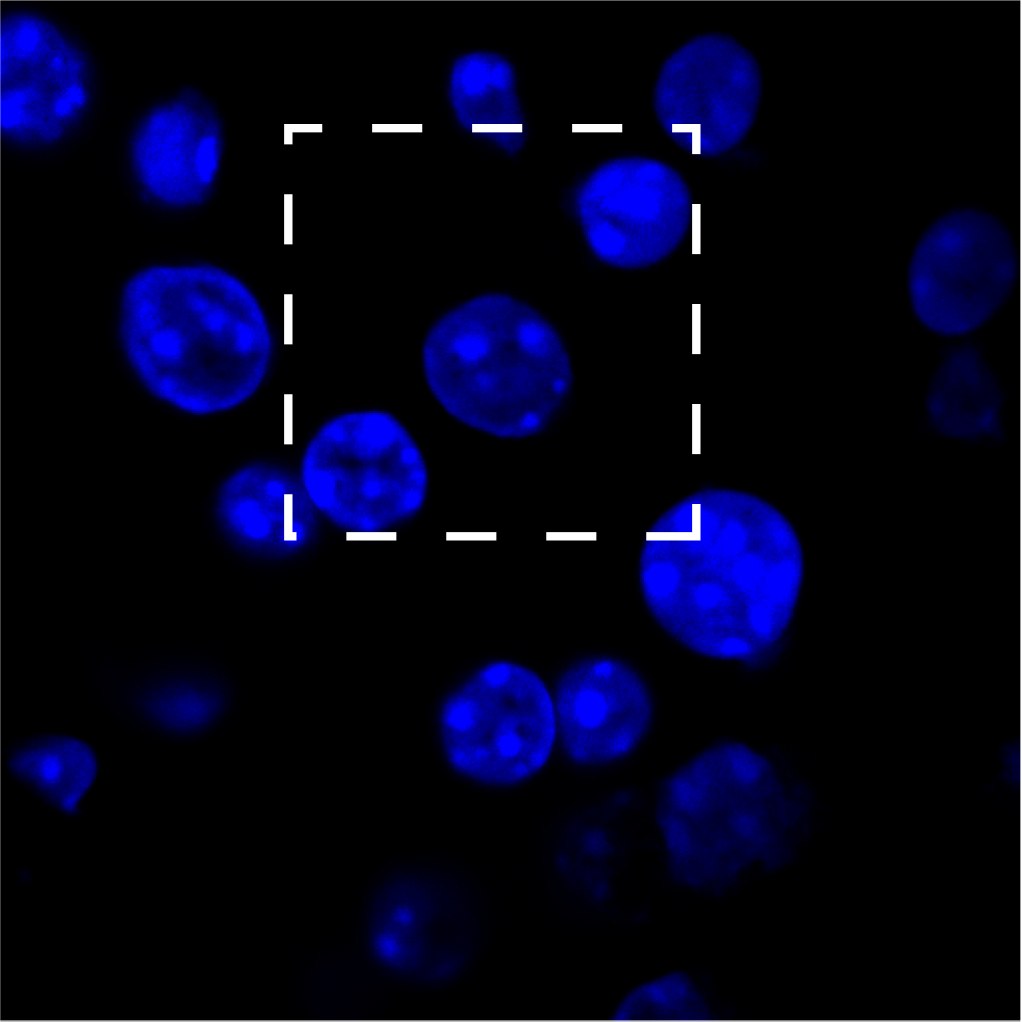

Supplement: Supplementary file 25 — Figure Source Data for Expanded View and Appendix [file 44318_2024_212_MOESM25_ESM.zip › Source Data for Expanded View and Appendix/Figure EV1/1T/WT-DAPI.jpg]

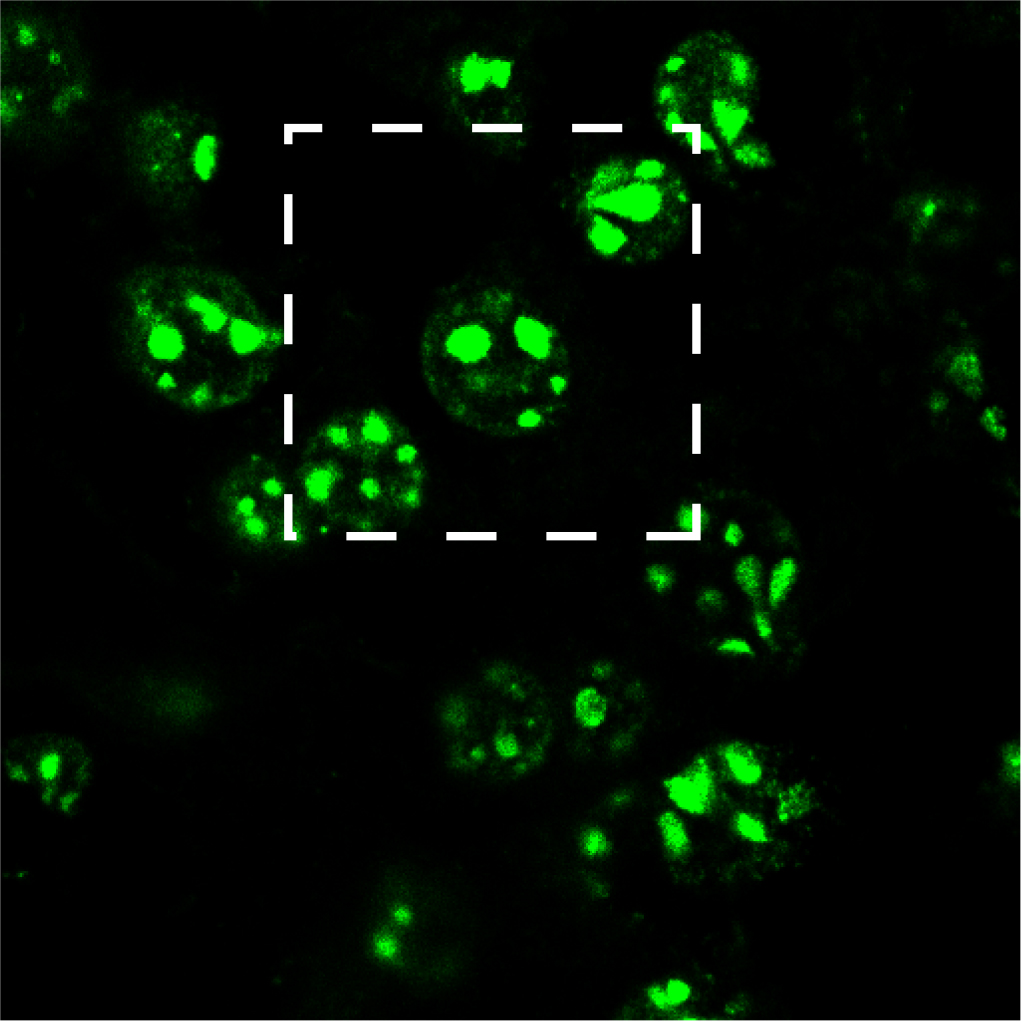

Supplement: Supplementary file 25 — Figure Source Data for Expanded View and Appendix [file 44318_2024_212_MOESM25_ESM.zip › Source Data for Expanded View and Appendix/Figure EV1/1T/WT-H3K9me3.jpg]

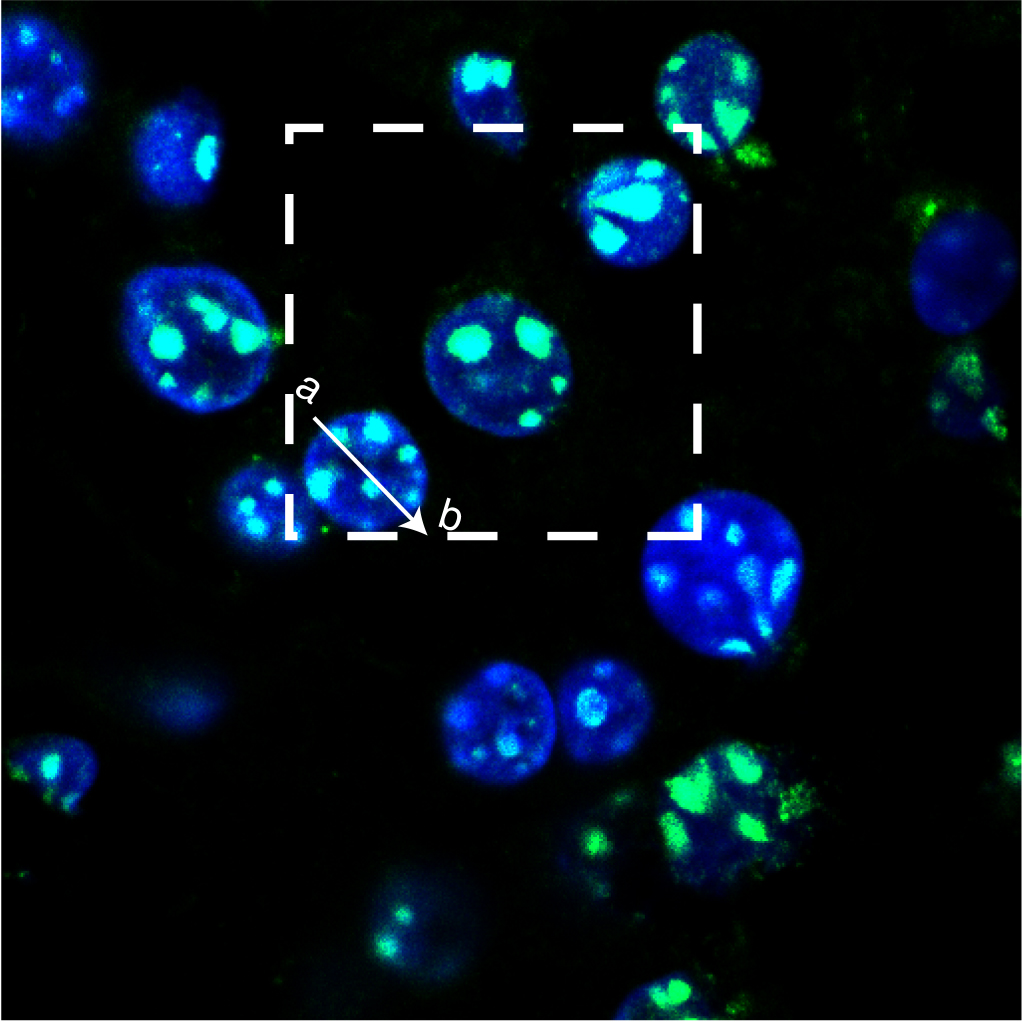

Supplement: Supplementary file 25 — Figure Source Data for Expanded View and Appendix [file 44318_2024_212_MOESM25_ESM.zip › Source Data for Expanded View and Appendix/Figure EV1/1T/WT-Merge.jpg]

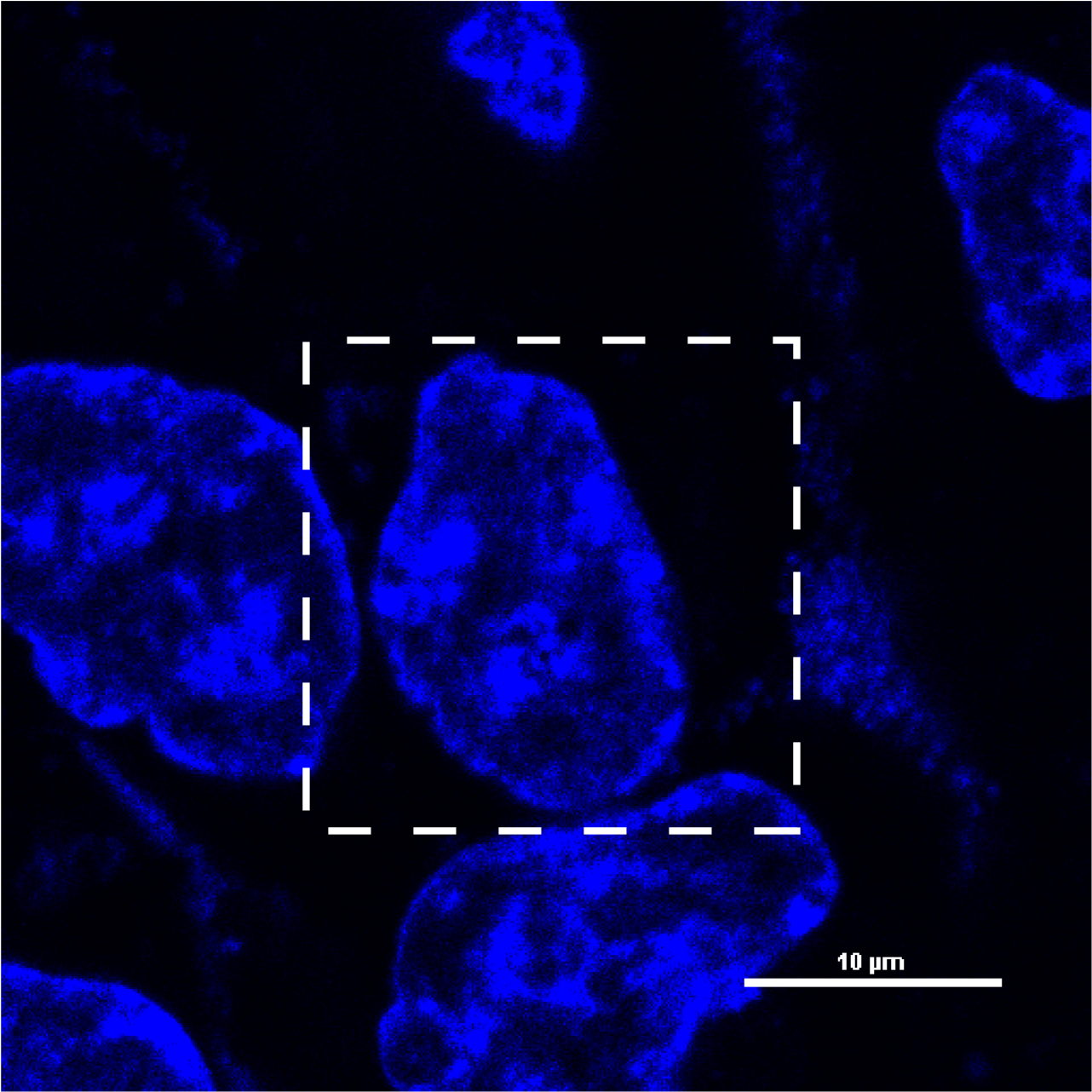

Supplement: Supplementary file 25 — Figure Source Data for Expanded View and Appendix [file 44318_2024_212_MOESM25_ESM.zip › Source Data for Expanded View and Appendix/Figure EV1/1U/Hoechst.jpg]

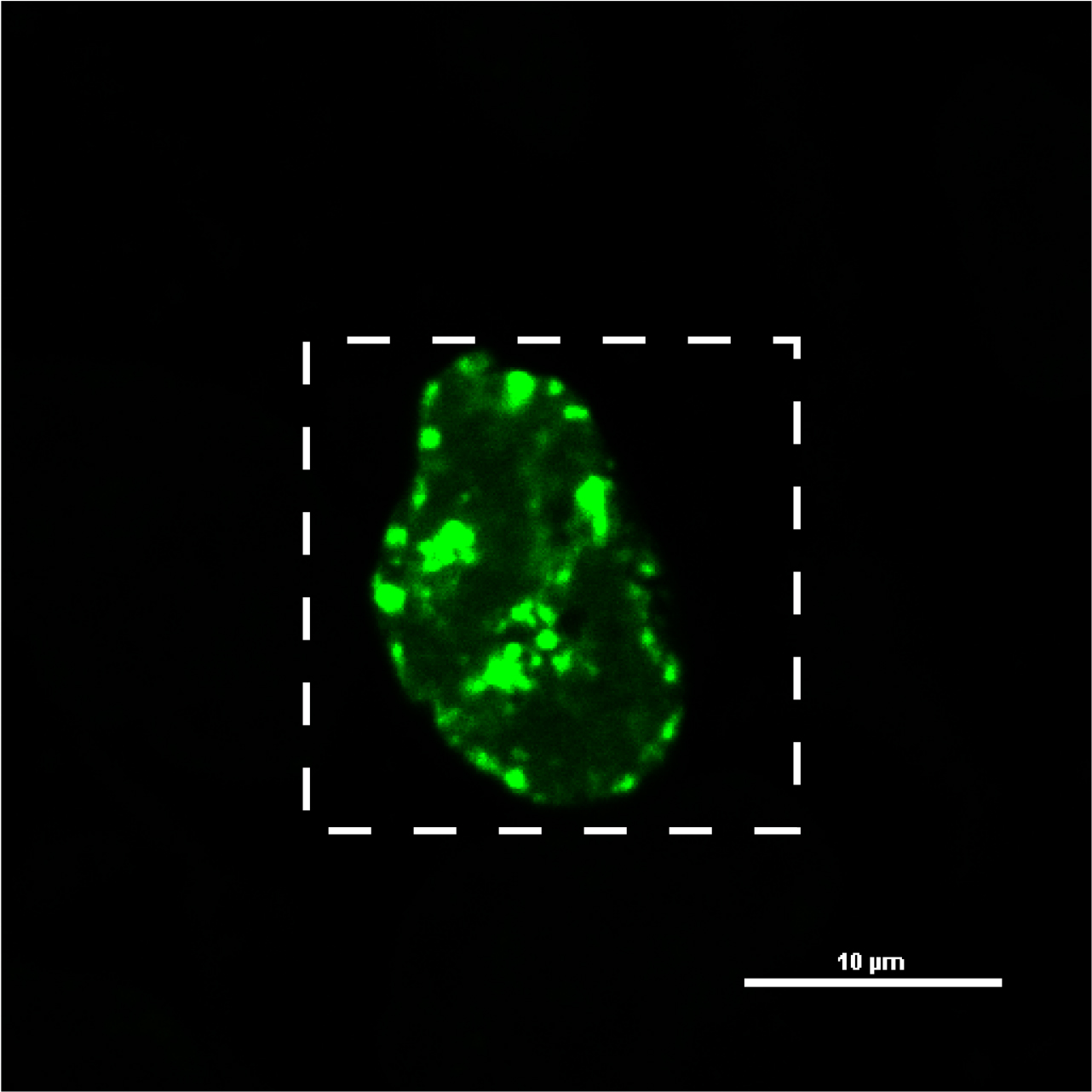

Supplement: Supplementary file 25 — Figure Source Data for Expanded View and Appendix [file 44318_2024_212_MOESM25_ESM.zip › Source Data for Expanded View and Appendix/Figure EV1/1U/HP1α.jpg]

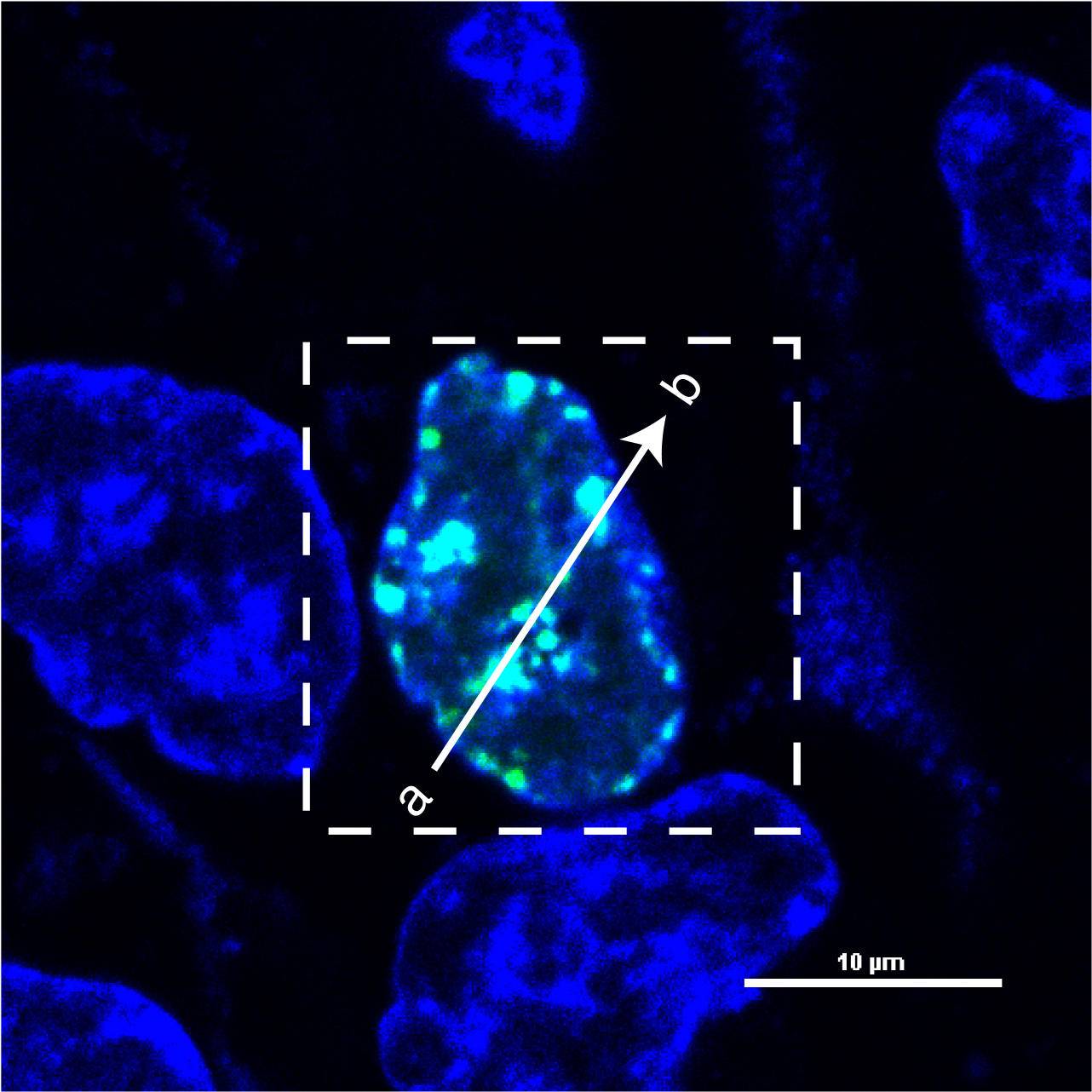

Supplement: Supplementary file 25 — Figure Source Data for Expanded View and Appendix [file 44318_2024_212_MOESM25_ESM.zip › Source Data for Expanded View and Appendix/Figure EV1/1U/Merge.jpg]

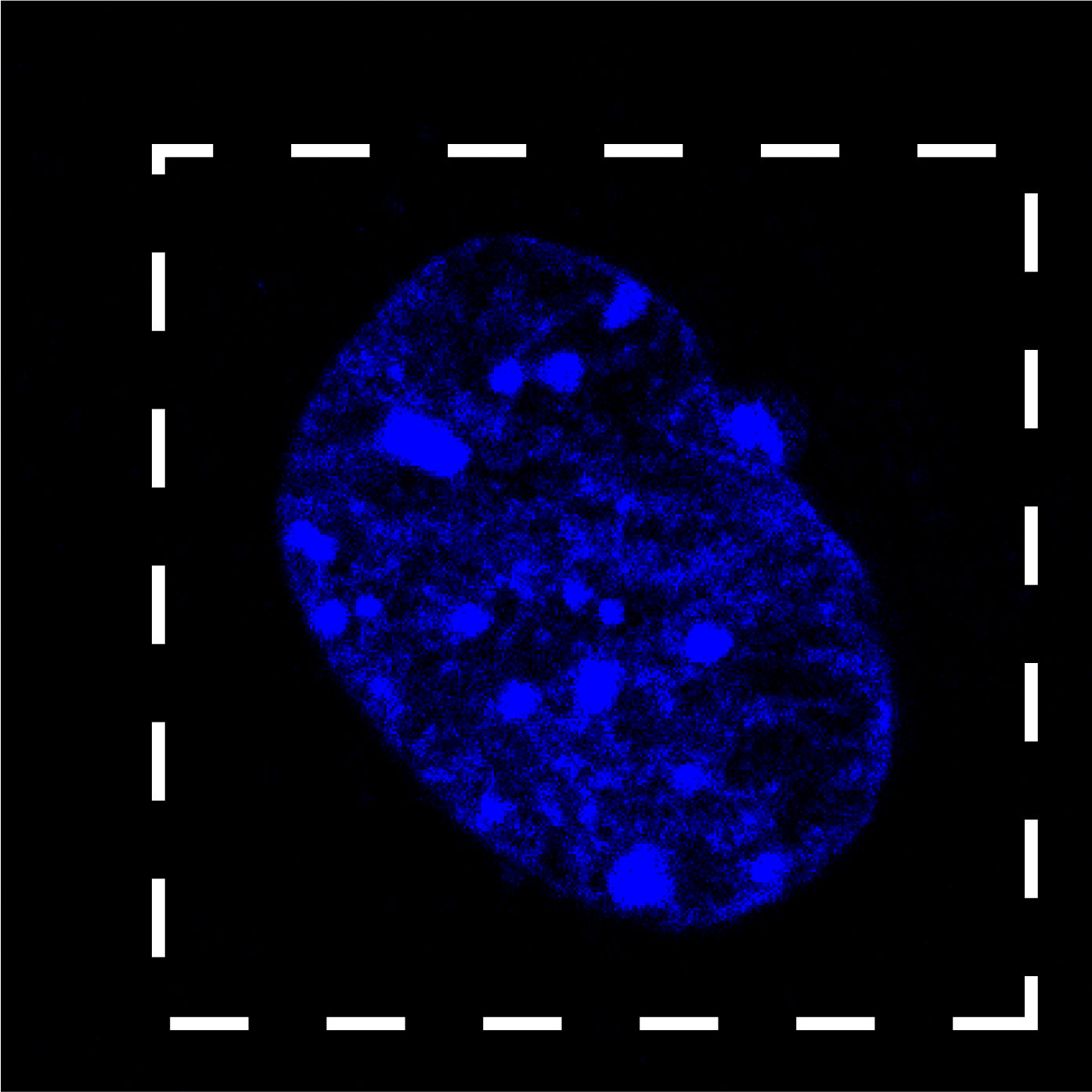

Supplement: Supplementary file 25 — Figure Source Data for Expanded View and Appendix [file 44318_2024_212_MOESM25_ESM.zip › Source Data for Expanded View and Appendix/Figure EV1/1W/DAPI.tif]

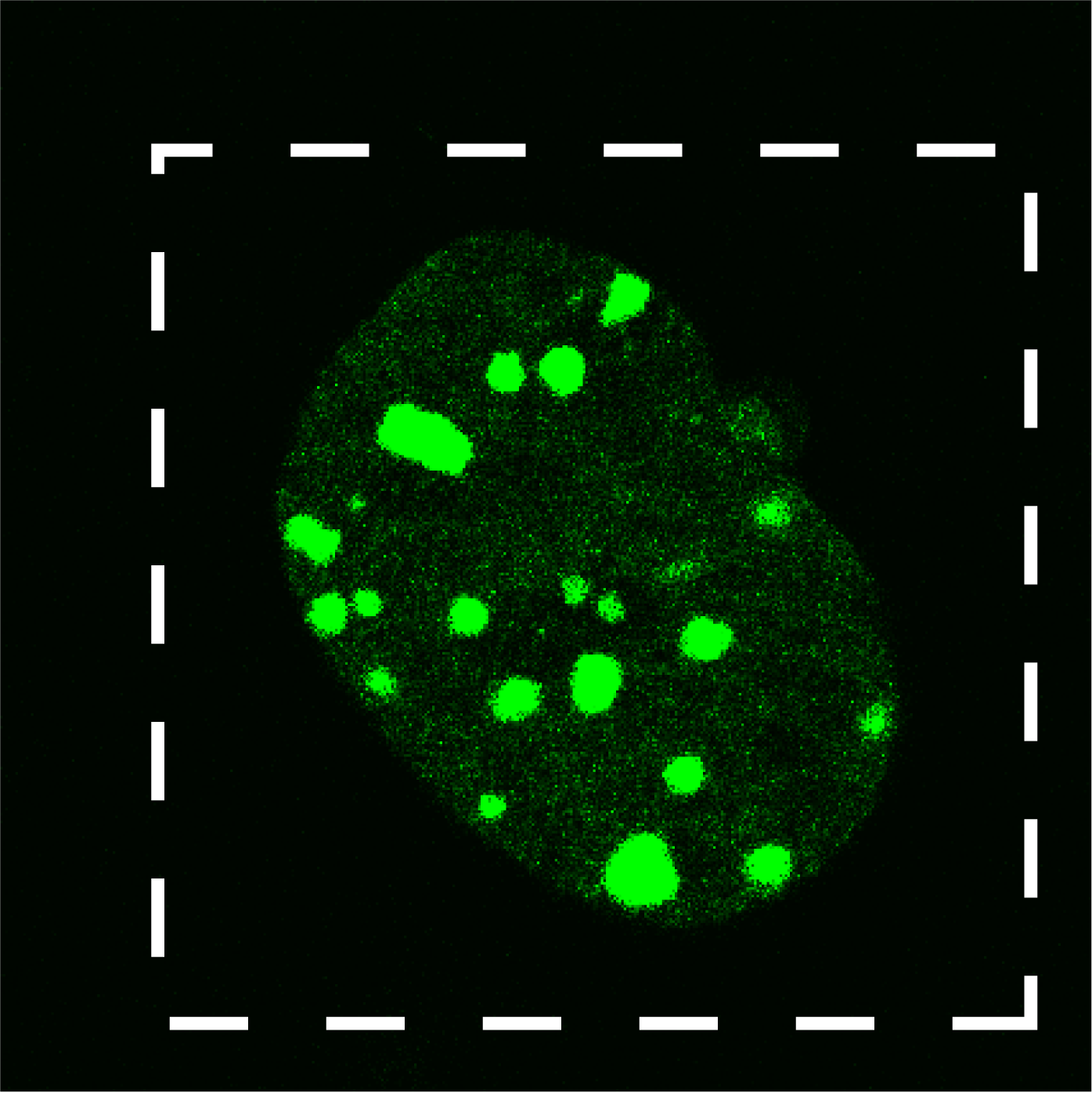

Supplement: Supplementary file 25 — Figure Source Data for Expanded View and Appendix [file 44318_2024_212_MOESM25_ESM.zip › Source Data for Expanded View and Appendix/Figure EV1/1W/HP1α.tif]

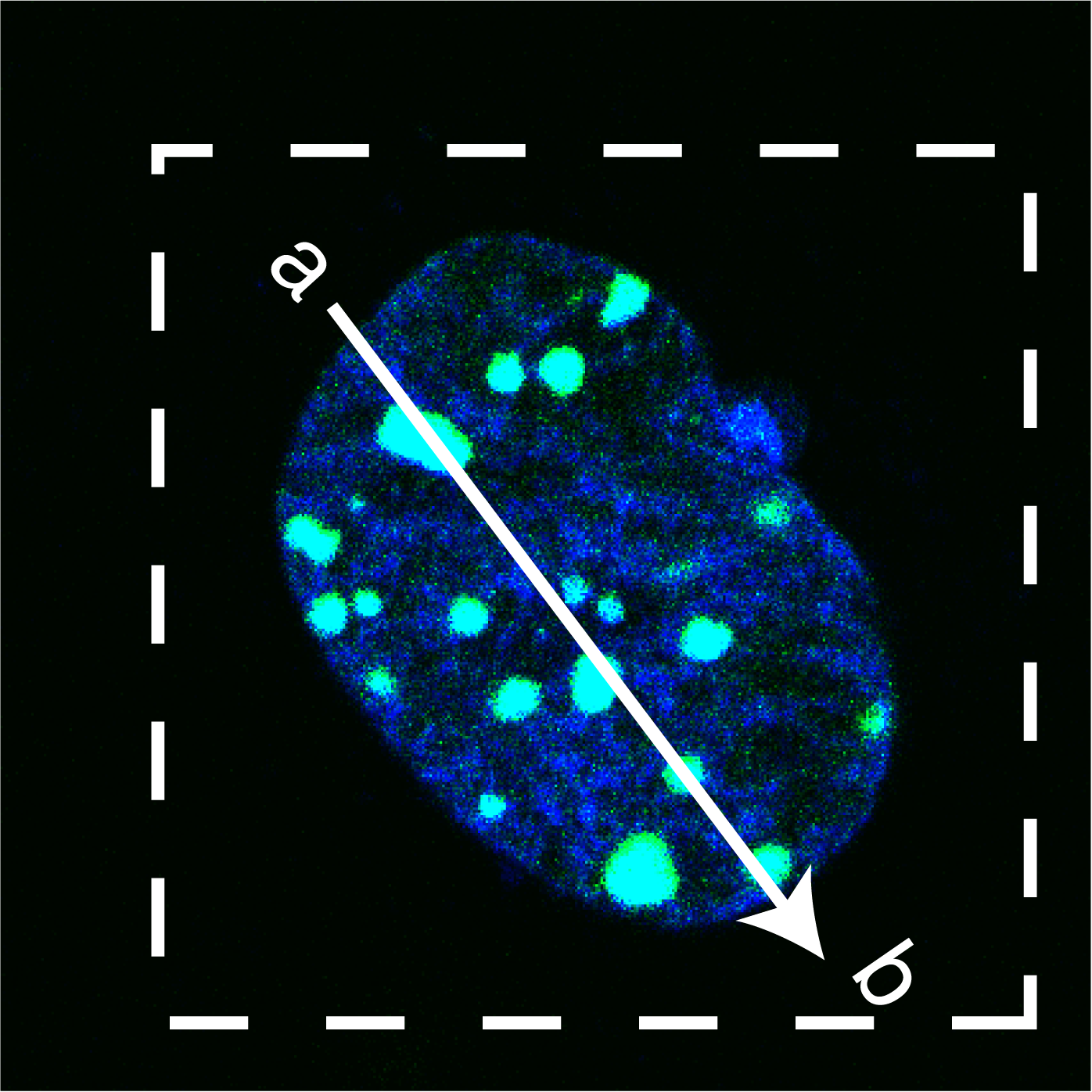

Supplement: Supplementary file 25 — Figure Source Data for Expanded View and Appendix [file 44318_2024_212_MOESM25_ESM.zip › Source Data for Expanded View and Appendix/Figure EV1/1W/Merge.tif]

**Fig. EV1R**

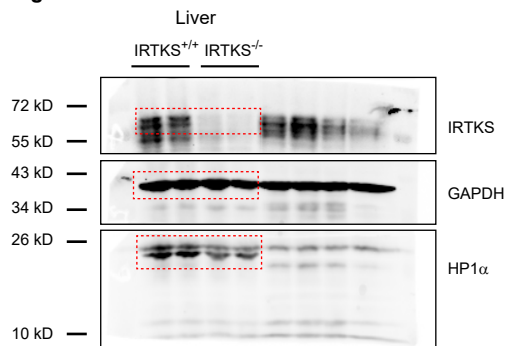

**Fig. EV1S**

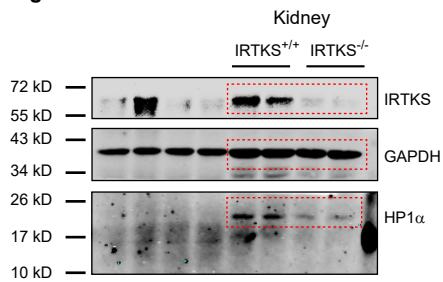

Supplement: Supplementary file 25 — Figure Source Data for Expanded View and Appendix [file 44318_2024_212_MOESM25_ESM.zip › Source Data for Expanded View and Appendix/Figure EV1/Source Data Fig. EV1.pdf]

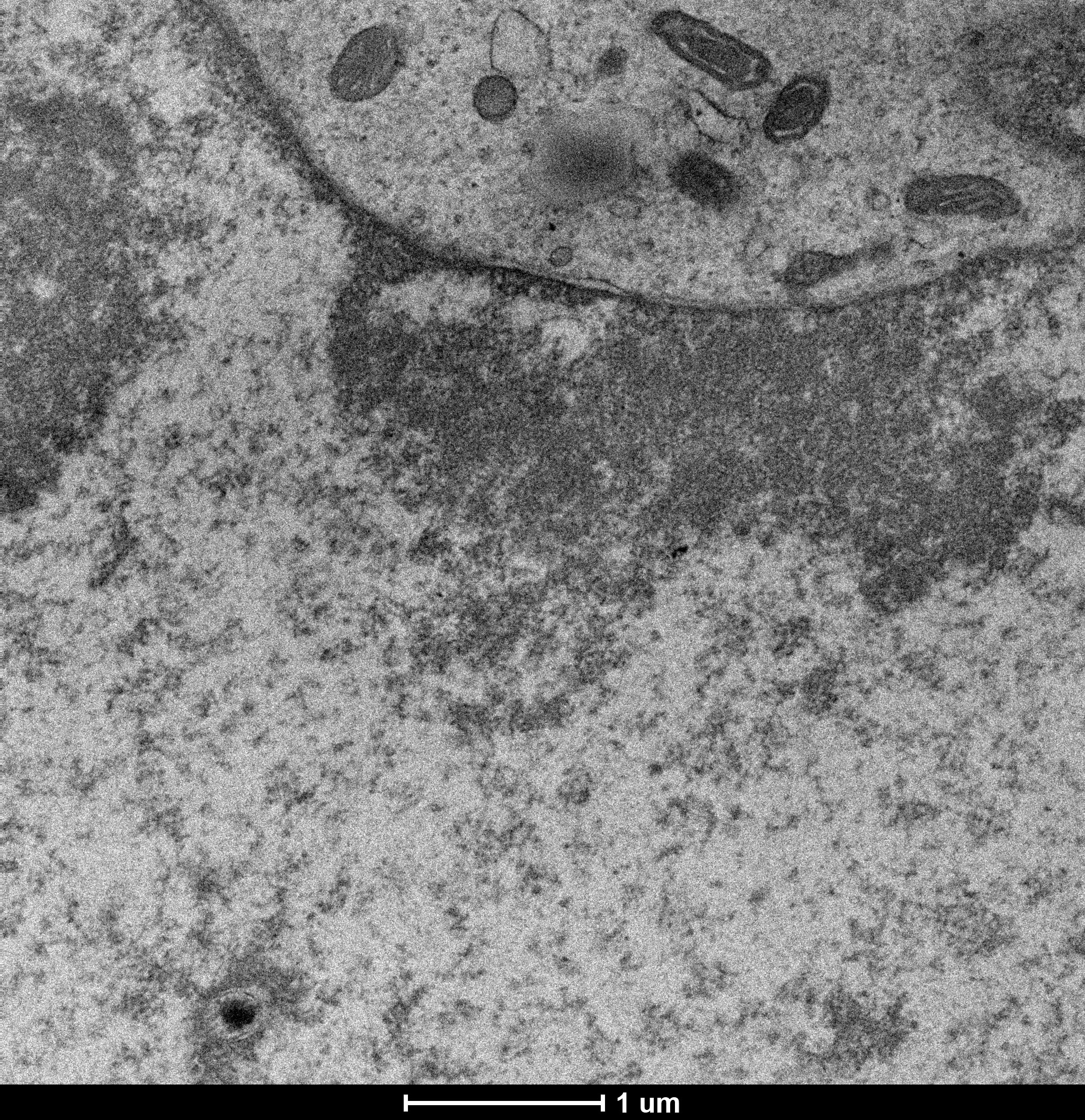

Supplement: Supplementary file 25 — Figure Source Data for Expanded View and Appendix [file 44318_2024_212_MOESM25_ESM.zip › Source Data for Expanded View and Appendix/Figure EV2/2D/sgControl-enlarged.jpg]

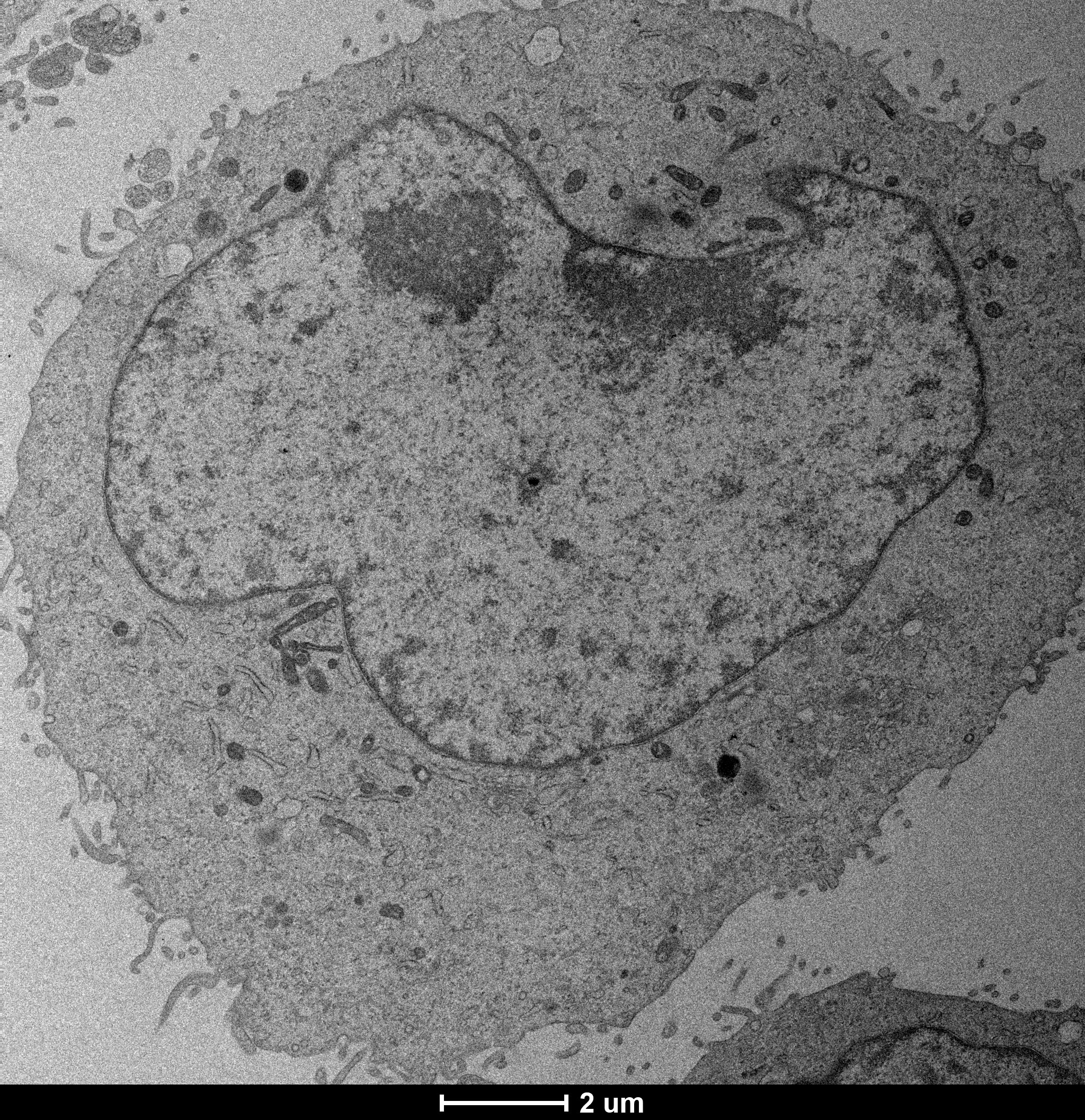

Supplement: Supplementary file 25 — Figure Source Data for Expanded View and Appendix [file 44318_2024_212_MOESM25_ESM.zip › Source Data for Expanded View and Appendix/Figure EV2/2D/sgControl.jpg]

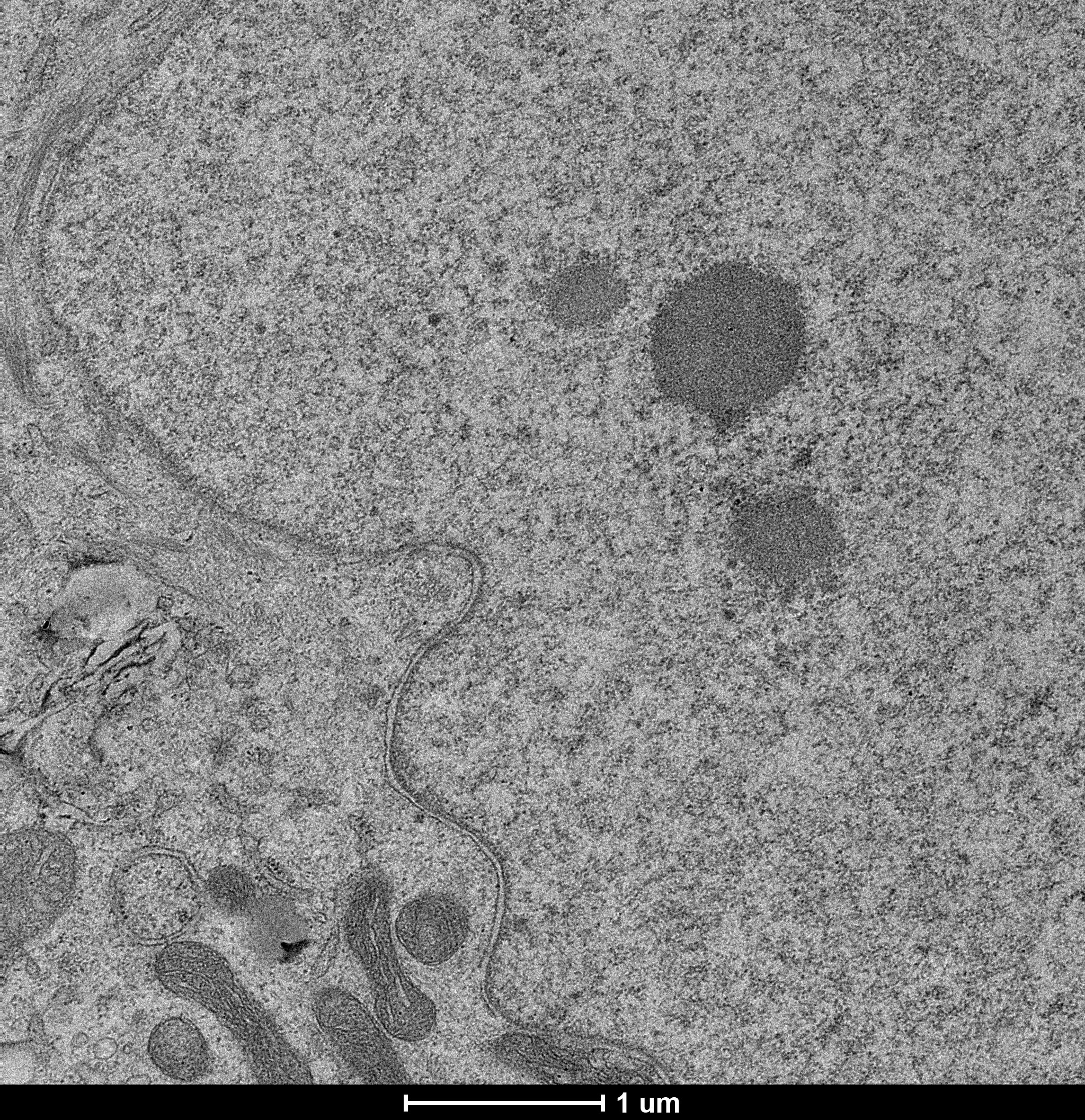

Supplement: Supplementary file 25 — Figure Source Data for Expanded View and Appendix [file 44318_2024_212_MOESM25_ESM.zip › Source Data for Expanded View and Appendix/Figure EV2/2D/sgIRTKS-enlarged.jpg]

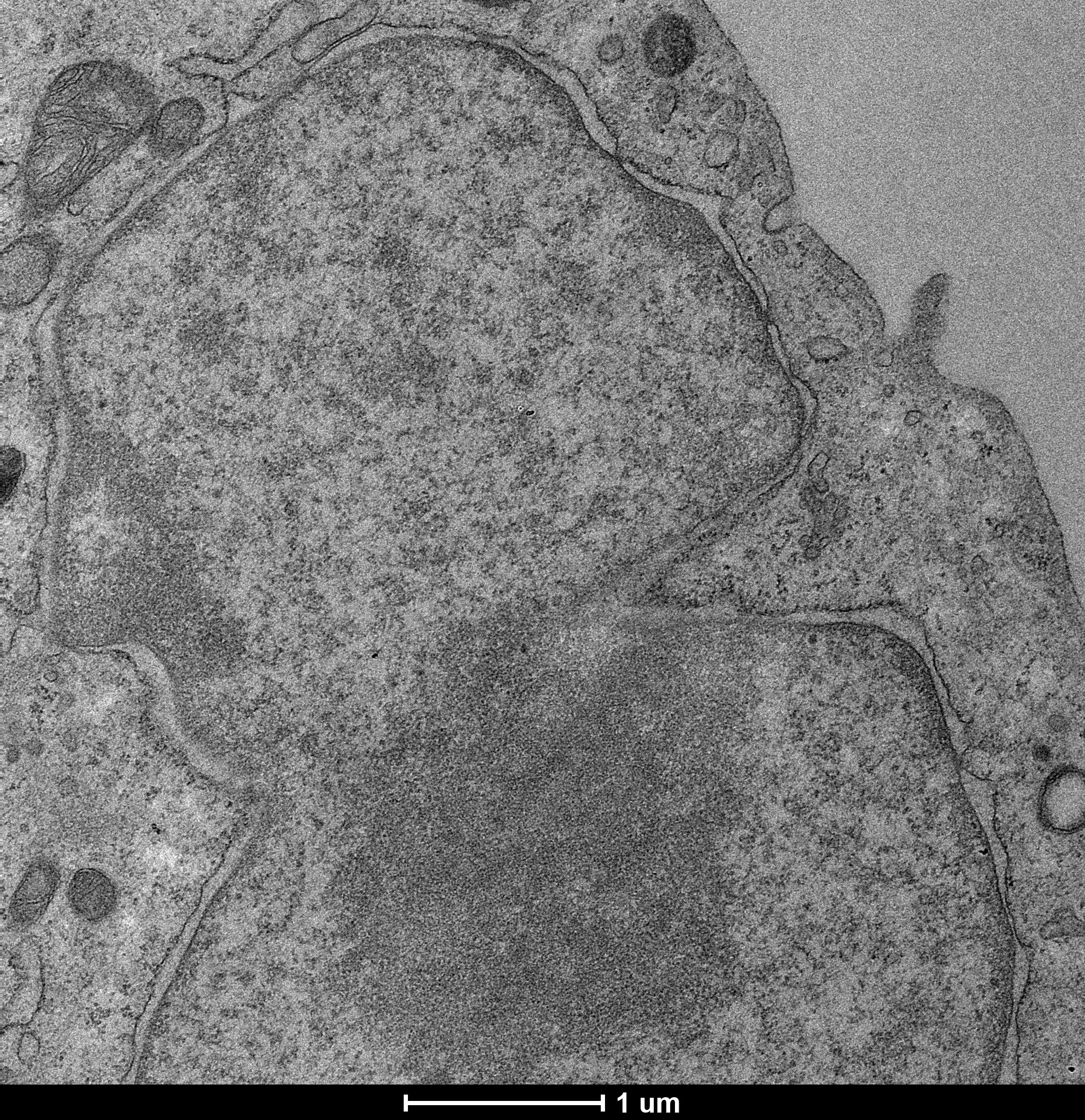

Supplement: Supplementary file 25 — Figure Source Data for Expanded View and Appendix [file 44318_2024_212_MOESM25_ESM.zip › Source Data for Expanded View and Appendix/Figure EV2/2D/sgIRTKS-Flag-IRTKS-enlarged.jpg]

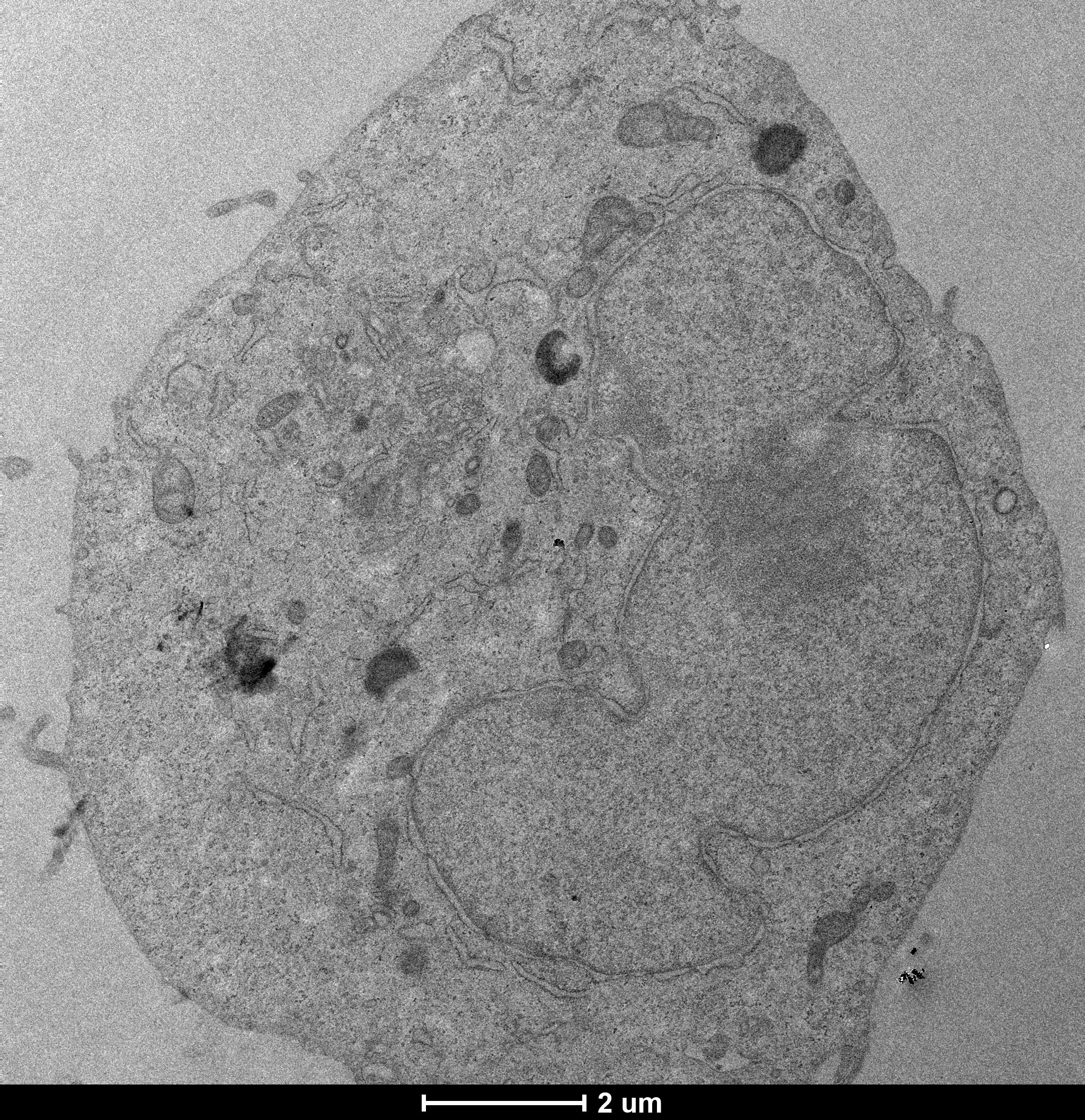

Supplement: Supplementary file 25 — Figure Source Data for Expanded View and Appendix [file 44318_2024_212_MOESM25_ESM.zip › Source Data for Expanded View and Appendix/Figure EV2/2D/sgIRTKS-Flag-IRTKS.jpg]

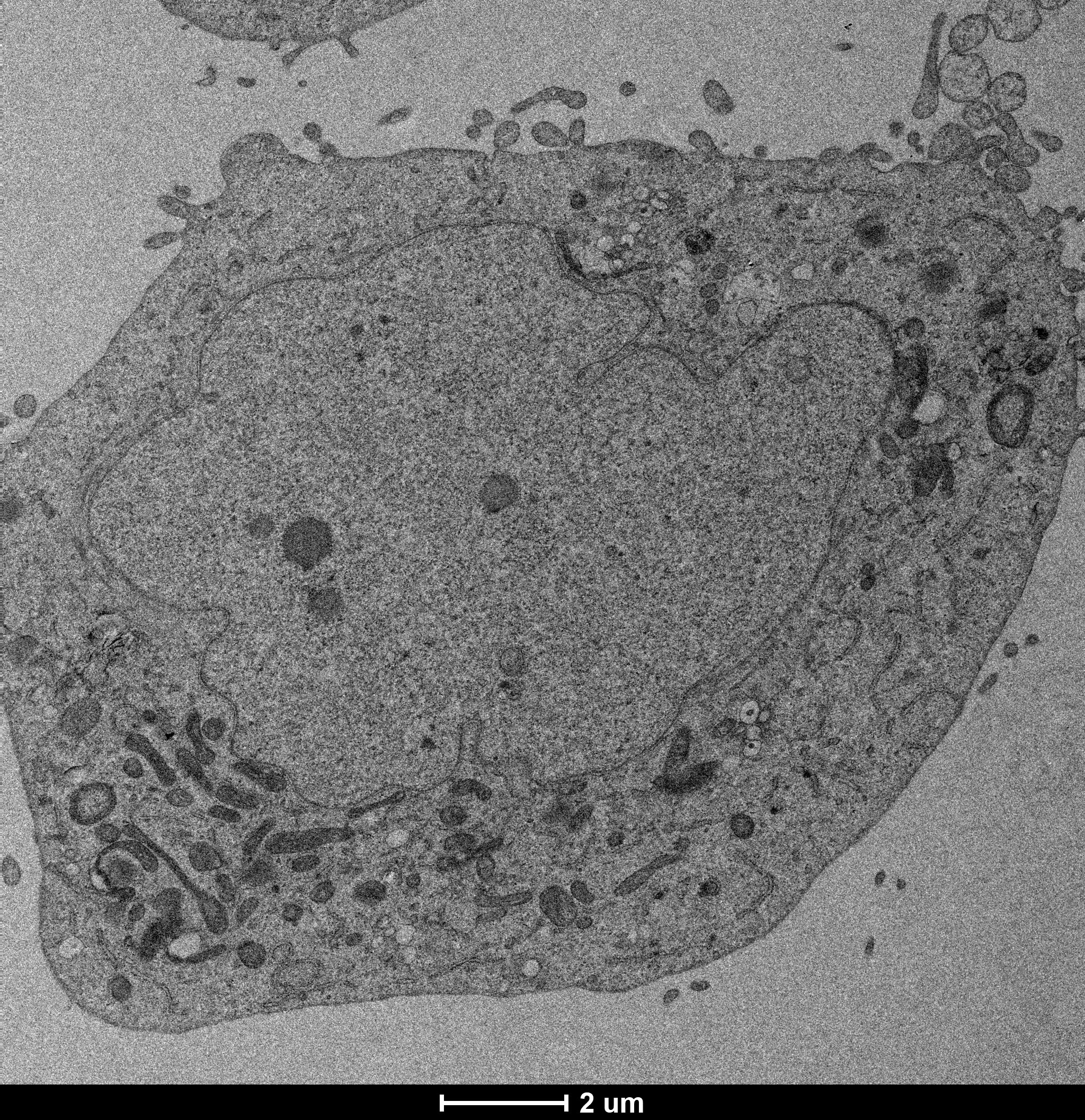

Supplement: Supplementary file 25 — Figure Source Data for Expanded View and Appendix [file 44318_2024_212_MOESM25_ESM.zip › Source Data for Expanded View and Appendix/Figure EV2/2D/sgIRTKS.jpg]

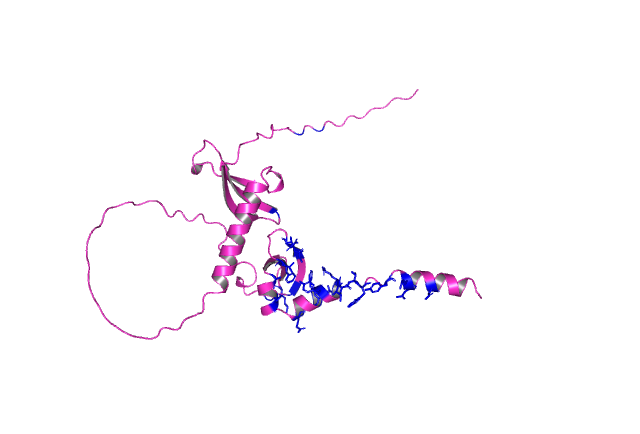

Supplement: Supplementary file 25 — Figure Source Data for Expanded View and Appendix [file 44318_2024_212_MOESM25_ESM.zip › Source Data for Expanded View and Appendix/Figure EV2/2L/HP1α.png]

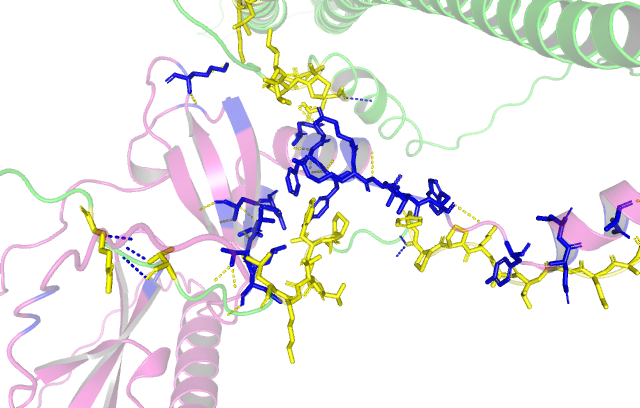

Supplement: Supplementary file 25 — Figure Source Data for Expanded View and Appendix [file 44318_2024_212_MOESM25_ESM.zip › Source Data for Expanded View and Appendix/Figure EV2/2L/IRTKS-HP1a complex enlarged.png]

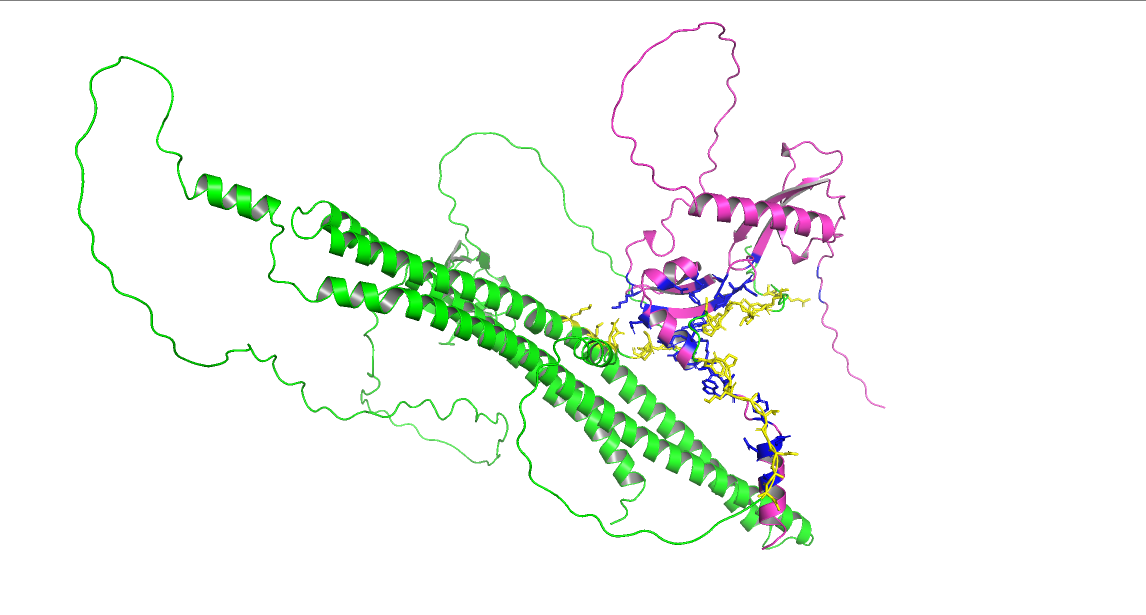

Supplement: Supplementary file 25 — Figure Source Data for Expanded View and Appendix [file 44318_2024_212_MOESM25_ESM.zip › Source Data for Expanded View and Appendix/Figure EV2/2L/IRTKS-HP1a complex.png]

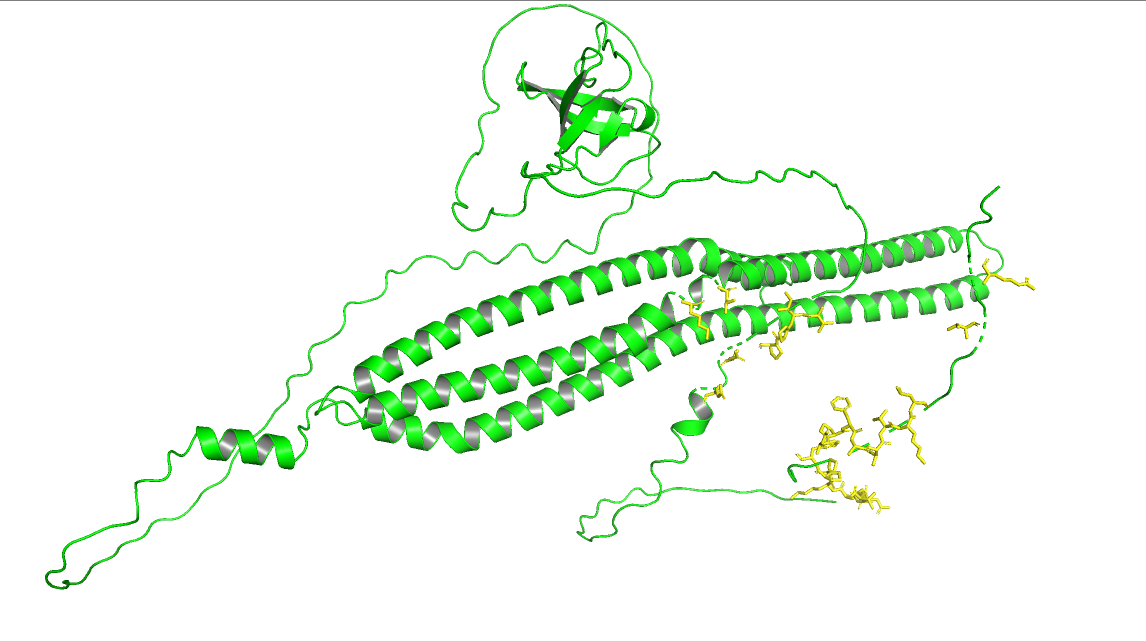

Supplement: Supplementary file 25 — Figure Source Data for Expanded View and Appendix [file 44318_2024_212_MOESM25_ESM.zip › Source Data for Expanded View and Appendix/Figure EV2/2L/IRTKS.png]

**Fig. EV2A**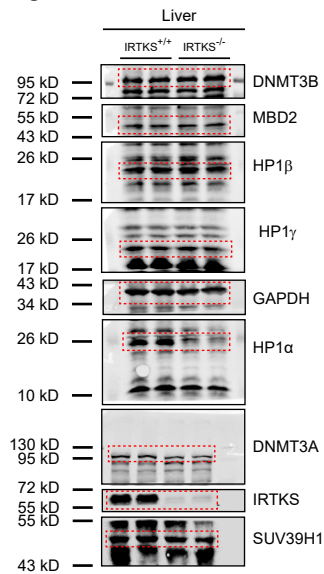**Fig. EV2G**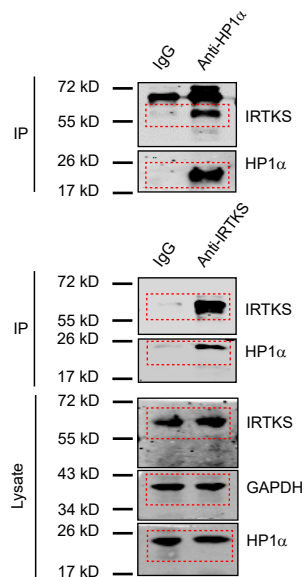**Fig. EV2B**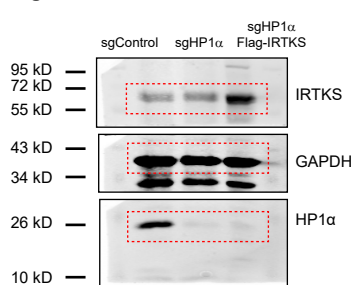**Fig. EV2F**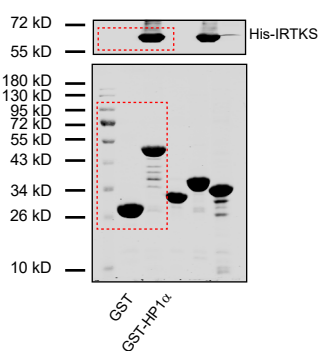**Fig. EV2J**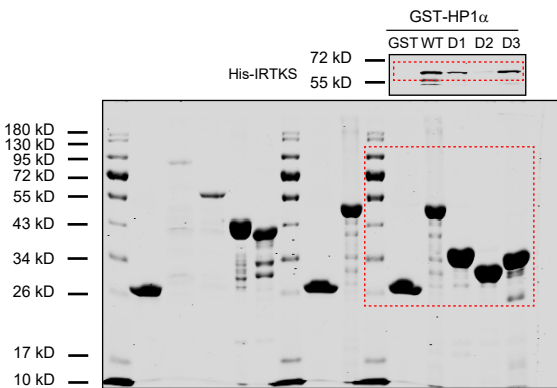**Fig. EV2C**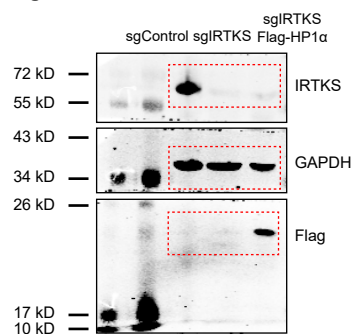**Fig. EV2H**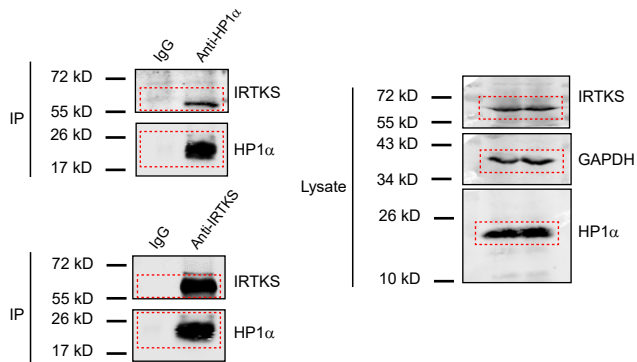**Fig. EV2K**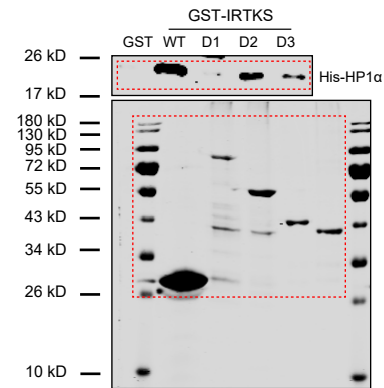**Fig. EV2E**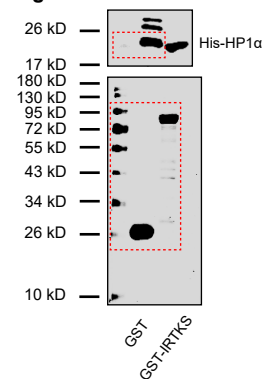

Supplement: Supplementary file 25 — Figure Source Data for Expanded View and Appendix [file 44318_2024_212_MOESM25_ESM.zip › Source Data for Expanded View and Appendix/Figure EV2/Source Data Fig. EV2.pdf]

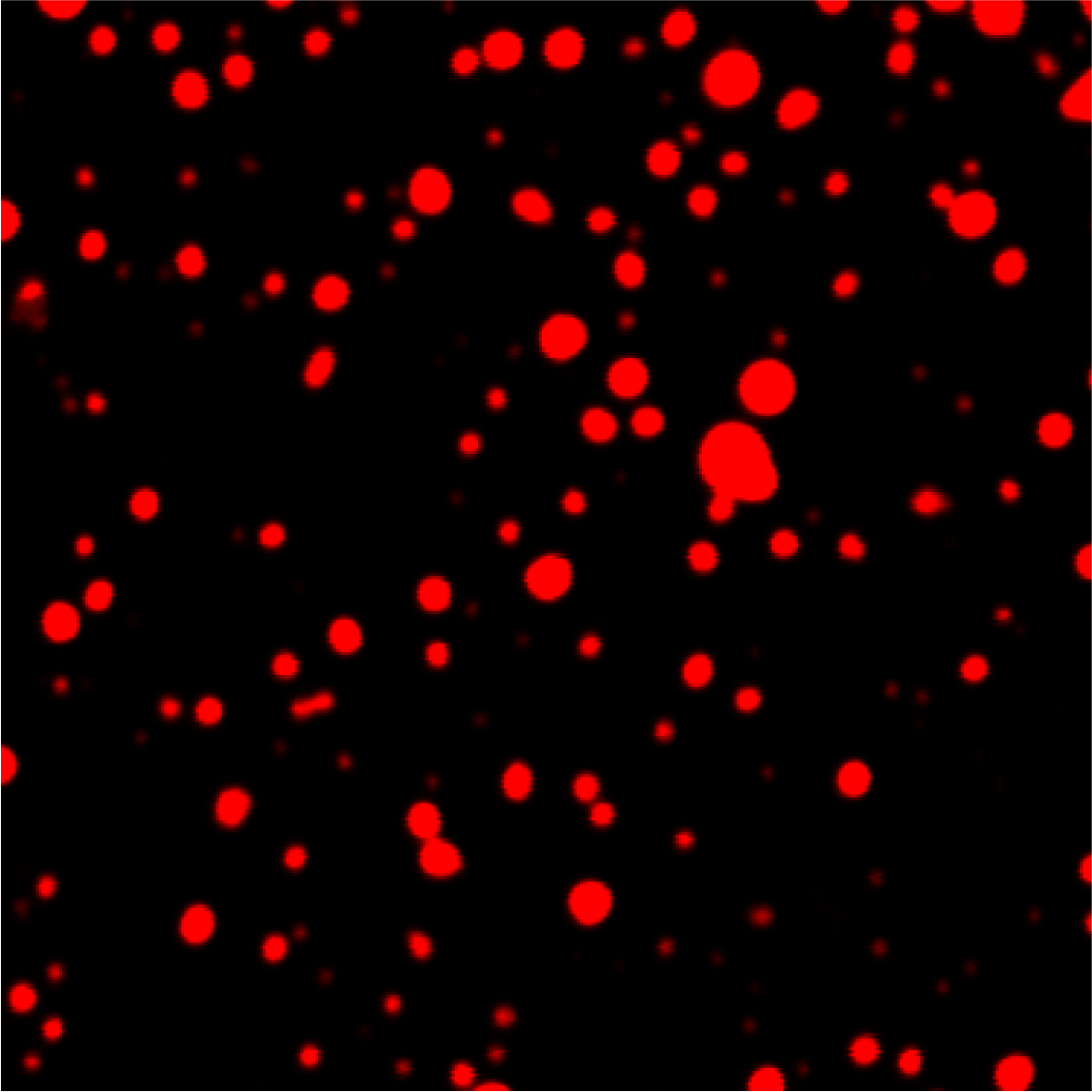

Supplement: Supplementary file 25 — Figure Source Data for Expanded View and Appendix [file 44318_2024_212_MOESM25_ESM.zip › Source Data for Expanded View and Appendix/Figure EV3/3K/150 mM.tif]

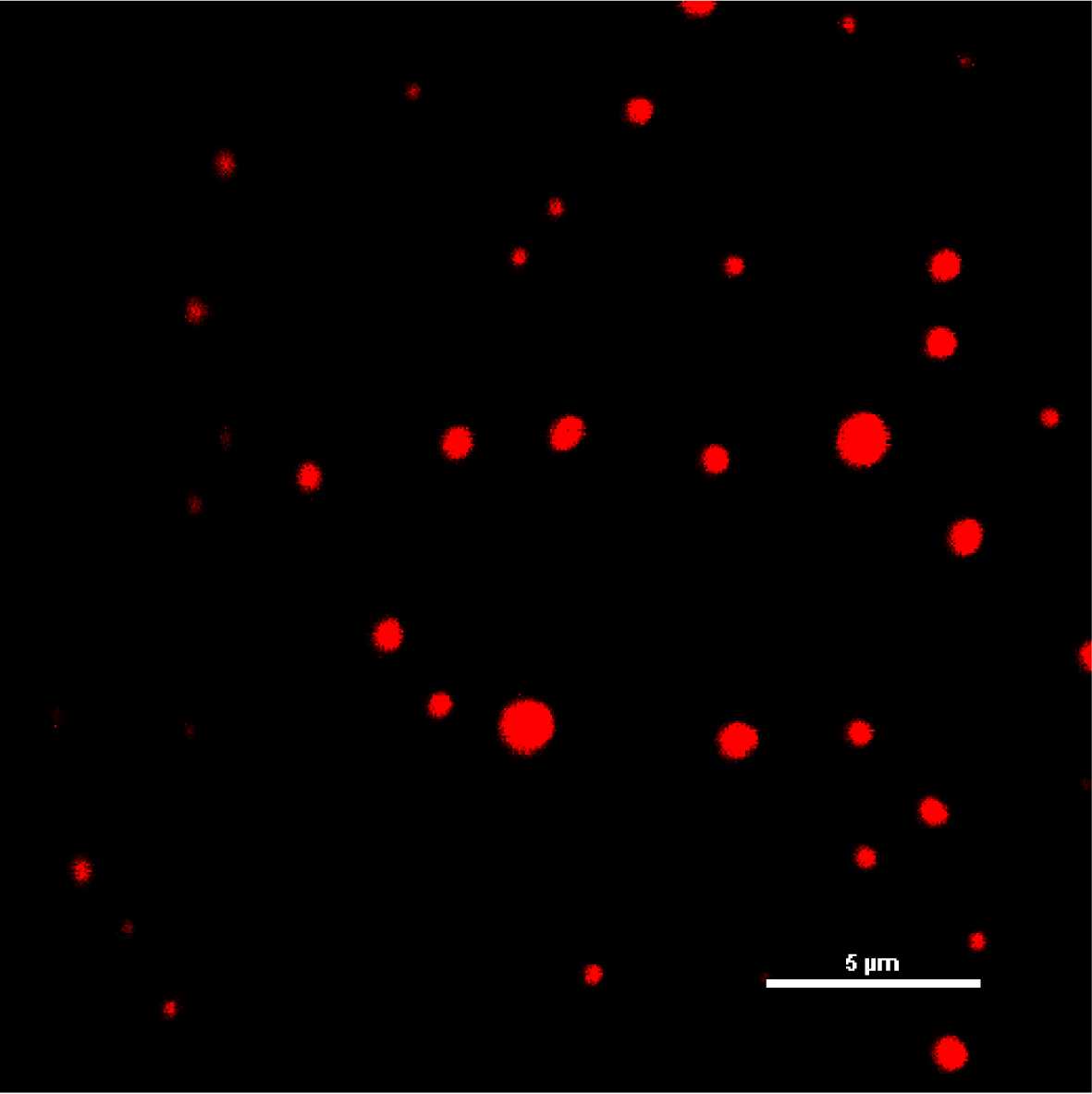

Supplement: Supplementary file 25 — Figure Source Data for Expanded View and Appendix [file 44318_2024_212_MOESM25_ESM.zip › Source Data for Expanded View and Appendix/Figure EV3/3K/300 mM.tif]

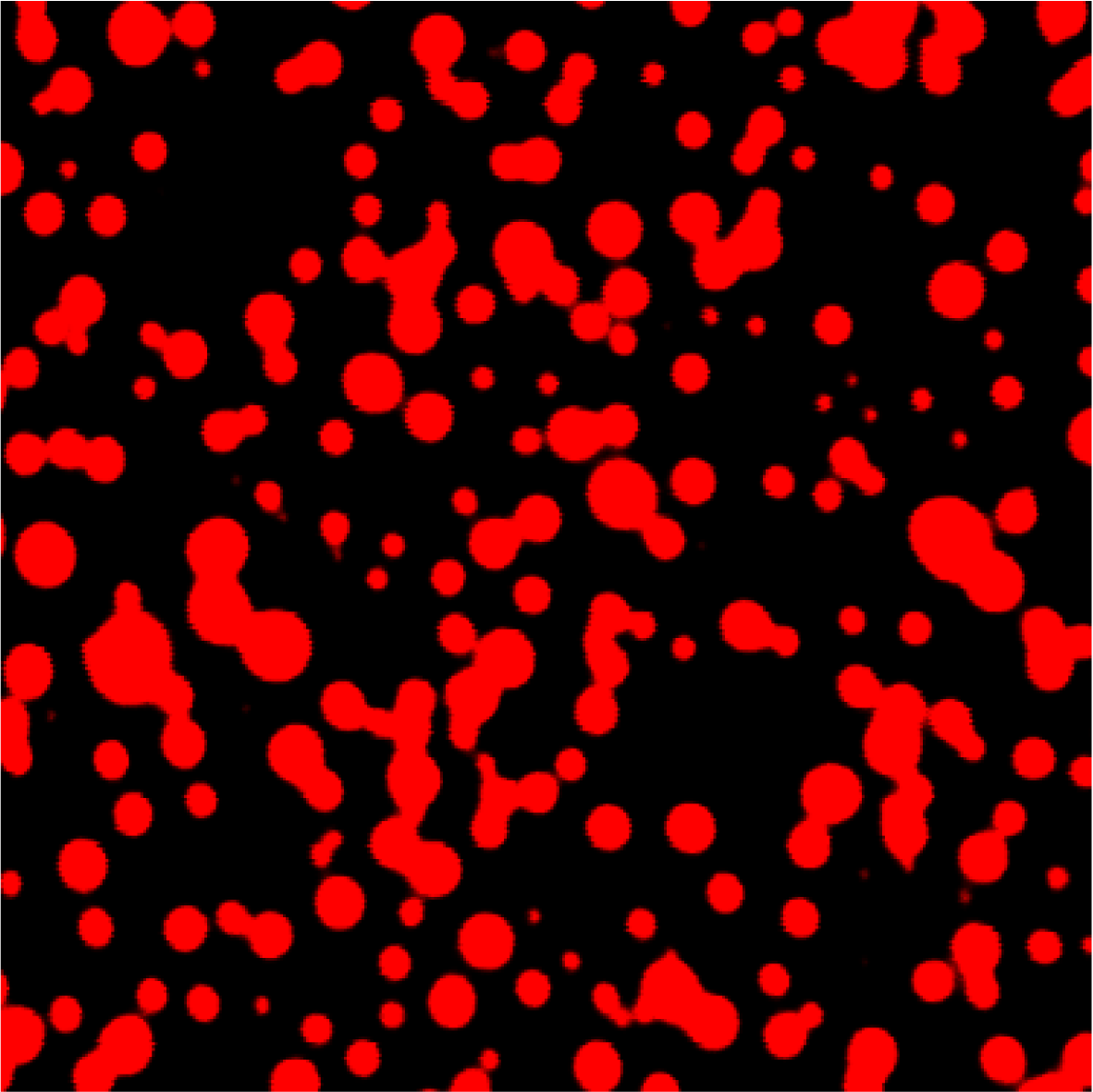

Supplement: Supplementary file 25 — Figure Source Data for Expanded View and Appendix [file 44318_2024_212_MOESM25_ESM.zip › Source Data for Expanded View and Appendix/Figure EV3/3K/50 mM.tif]

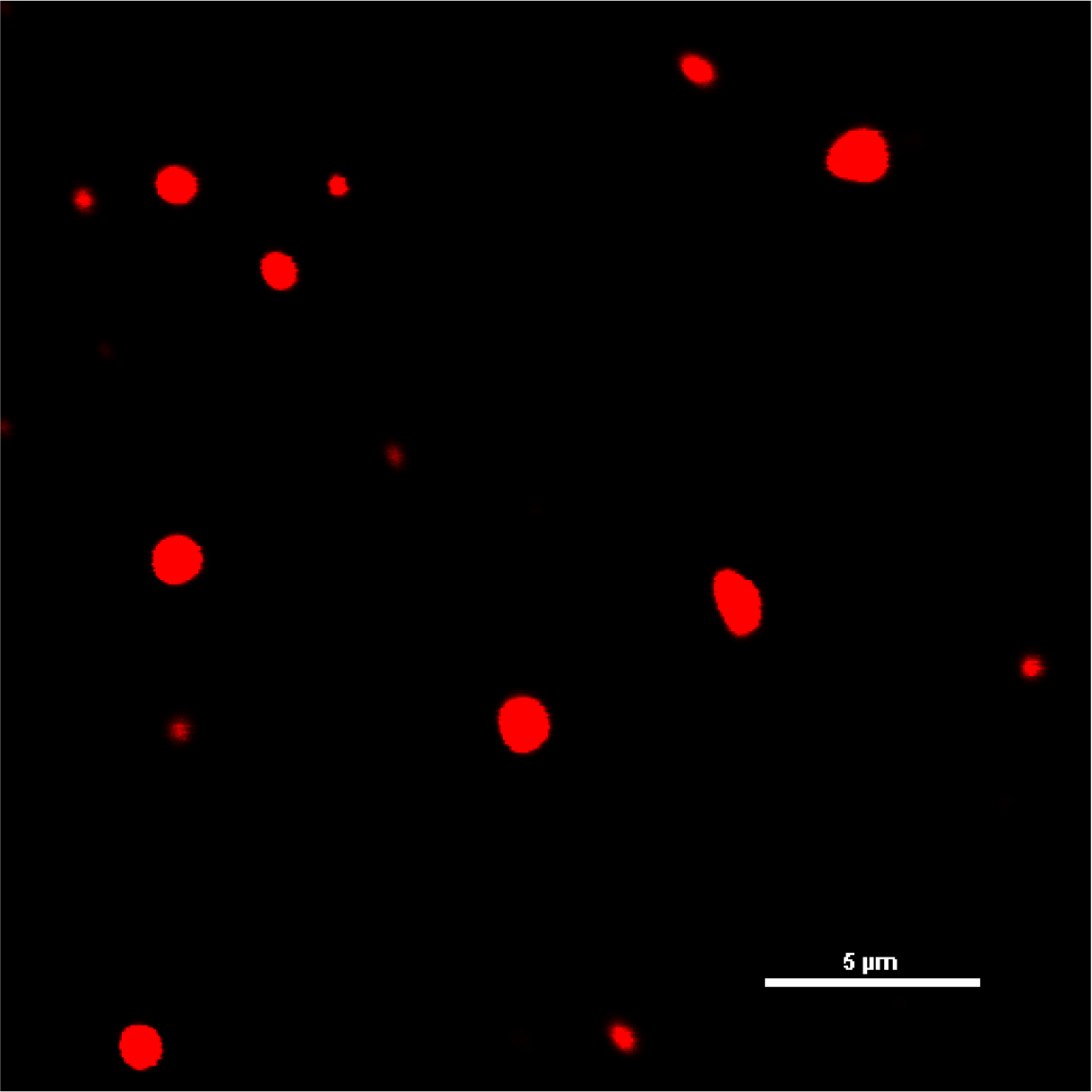

Supplement: Supplementary file 25 — Figure Source Data for Expanded View and Appendix [file 44318_2024_212_MOESM25_ESM.zip › Source Data for Expanded View and Appendix/Figure EV3/3L/1,6-hex.tif]

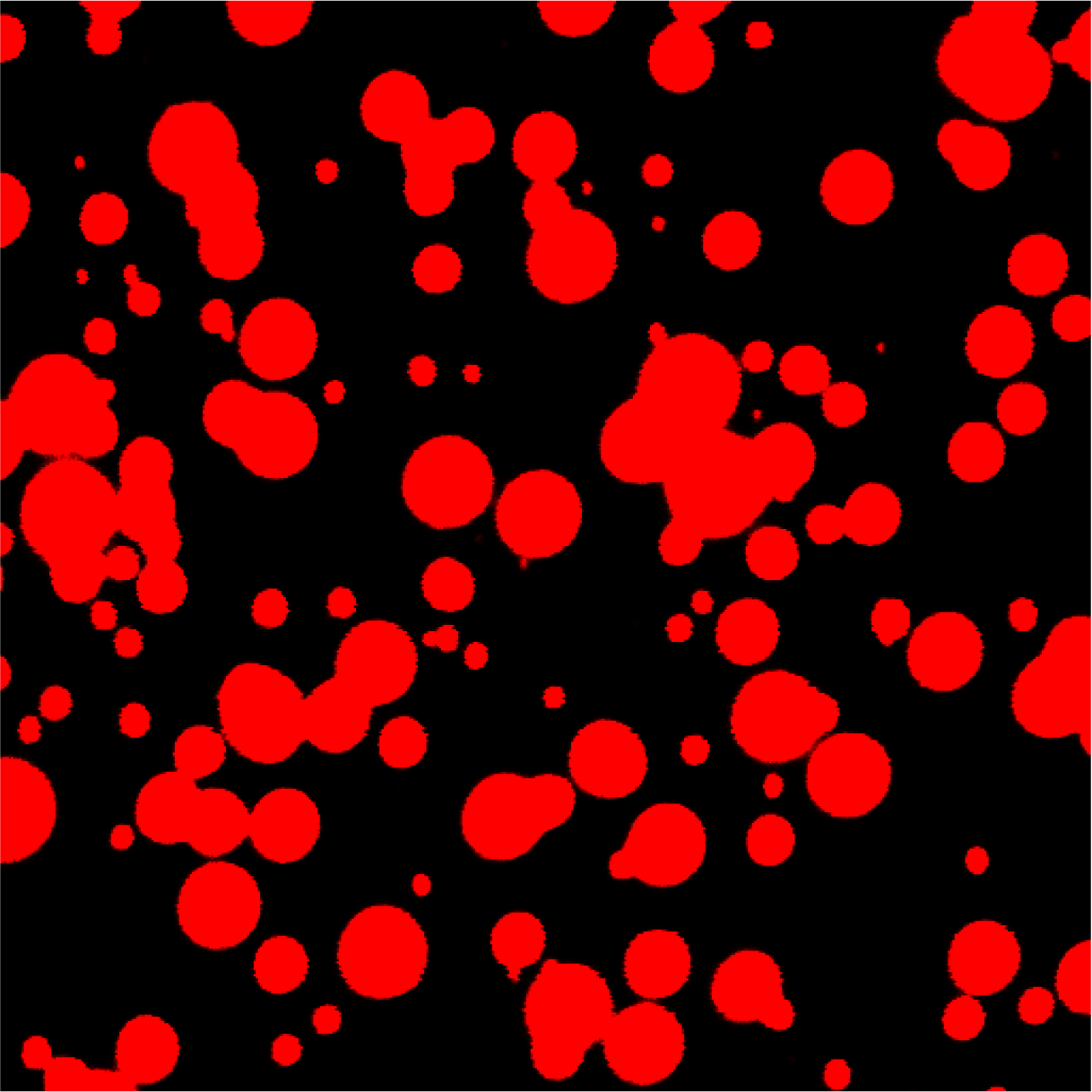

Supplement: Supplementary file 25 — Figure Source Data for Expanded View and Appendix [file 44318_2024_212_MOESM25_ESM.zip › Source Data for Expanded View and Appendix/Figure EV3/3L/Control.tif]

**Fig. EV3B**

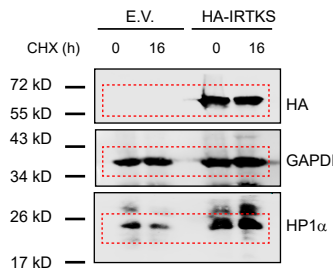

**Fig. EV3E**

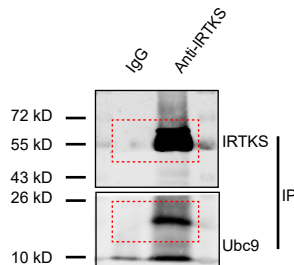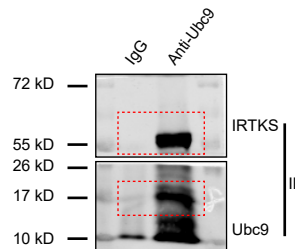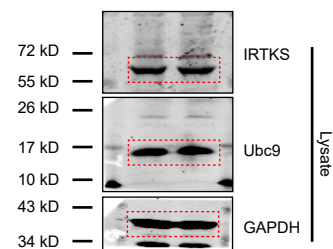

**Fig. EV3C**

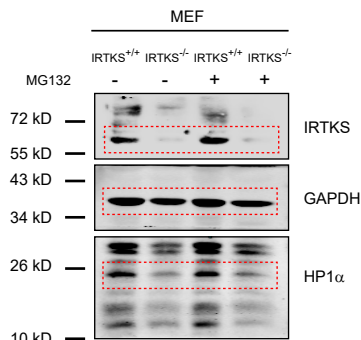

**Fig. EV3F**

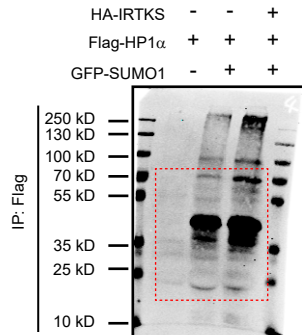

**Fig. EV3H**

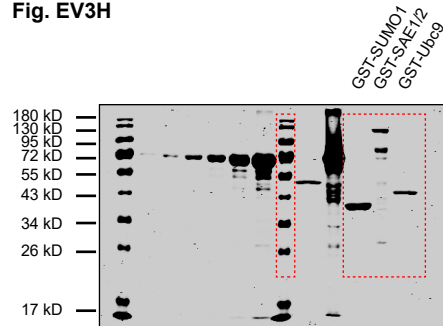

**Fig. EV3D**

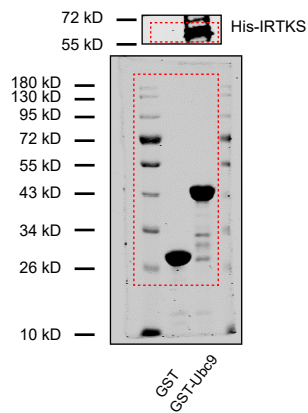

**Fig. EV3G**

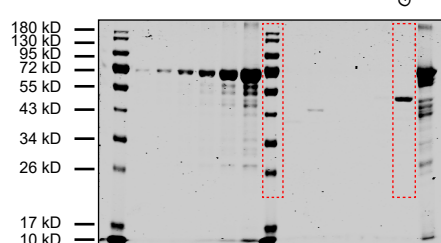

**Fig. EV3I**

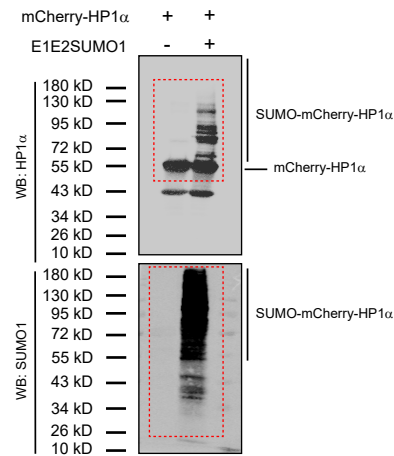

Supplement: Supplementary file 25 — Figure Source Data for Expanded View and Appendix [file 44318_2024_212_MOESM25_ESM.zip › Source Data for Expanded View and Appendix/Figure EV3/Source Data Fig. EV3.pdf]

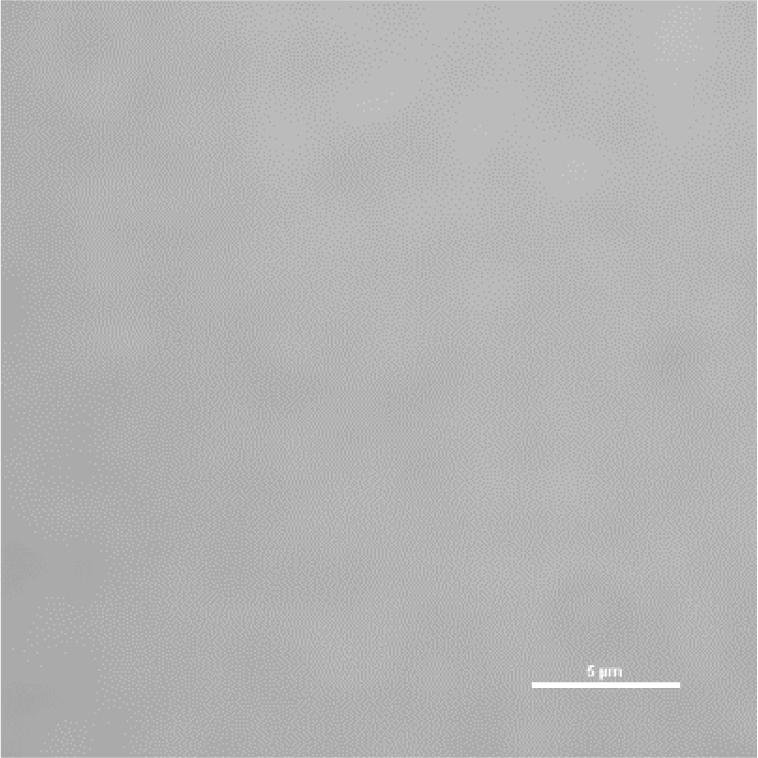

Supplement: Supplementary file 25 — Figure Source Data for Expanded View and Appendix [file 44318_2024_212_MOESM25_ESM.zip › Source Data for Expanded View and Appendix/Figure EV4/4C/mCherry-BF.tif]

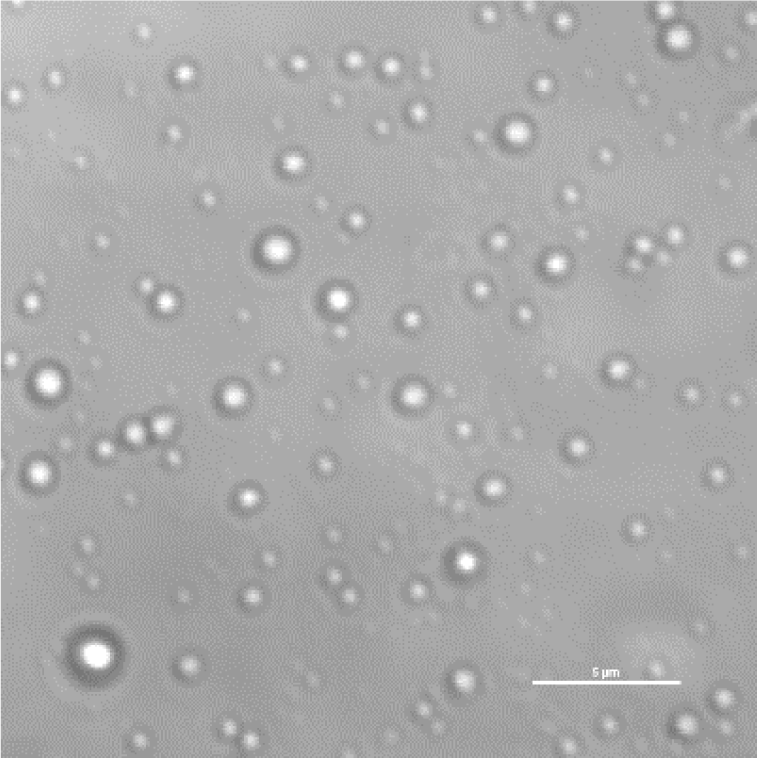

Supplement: Supplementary file 25 — Figure Source Data for Expanded View and Appendix [file 44318_2024_212_MOESM25_ESM.zip › Source Data for Expanded View and Appendix/Figure EV4/4C/mCherry-HP1α-BF.tif]

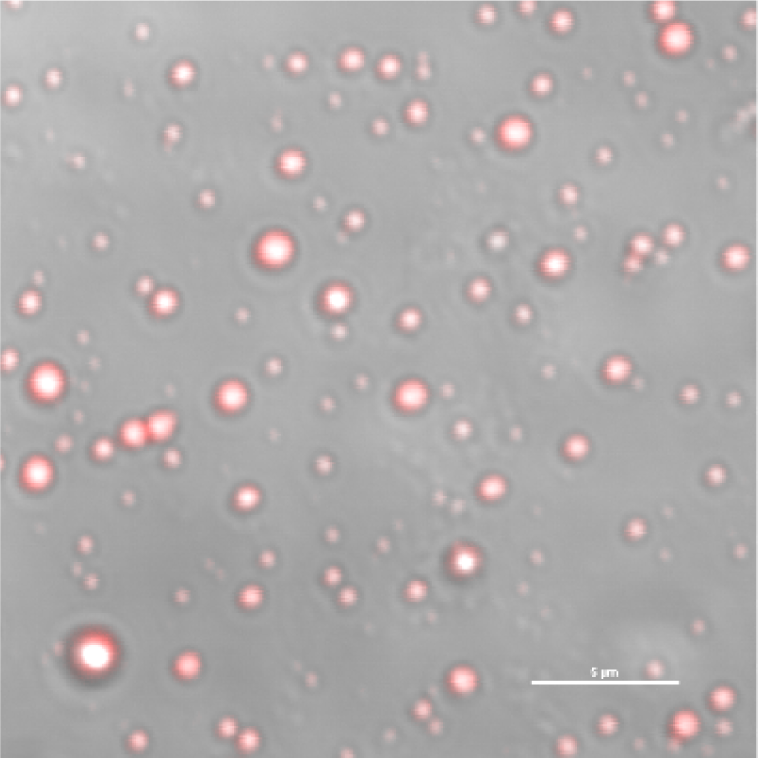

Supplement: Supplementary file 25 — Figure Source Data for Expanded View and Appendix [file 44318_2024_212_MOESM25_ESM.zip › Source Data for Expanded View and Appendix/Figure EV4/4C/mCherry-HP1α-Merge.tif]

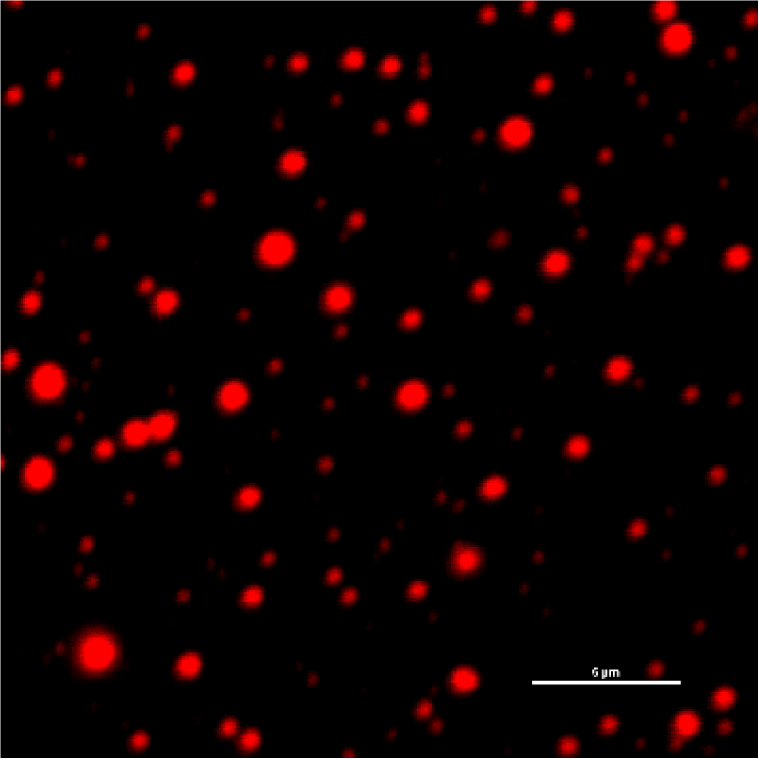

Supplement: Supplementary file 25 — Figure Source Data for Expanded View and Appendix [file 44318_2024_212_MOESM25_ESM.zip › Source Data for Expanded View and Appendix/Figure EV4/4C/mCherry-HP1α.tif]

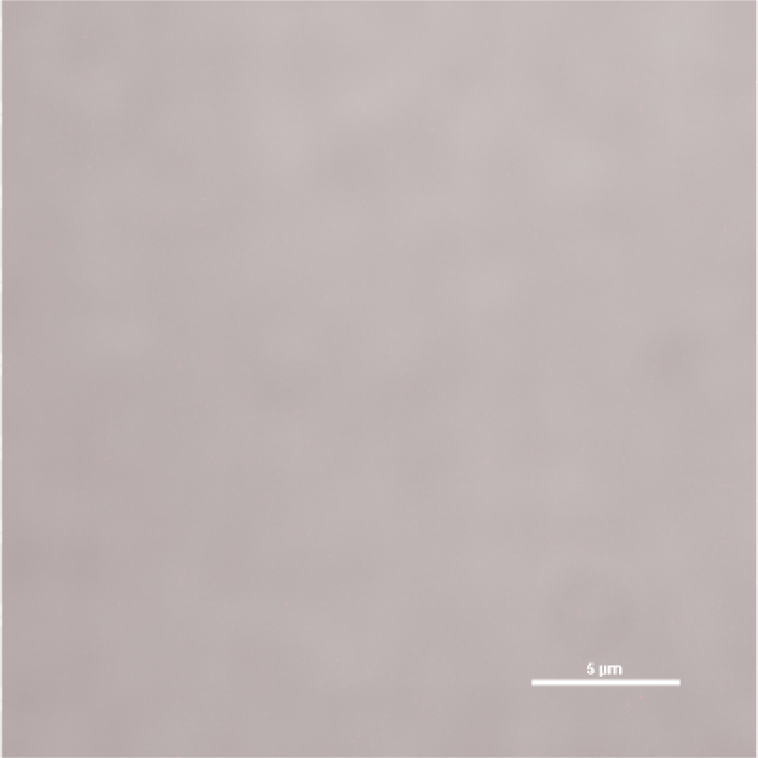

Supplement: Supplementary file 25 — Figure Source Data for Expanded View and Appendix [file 44318_2024_212_MOESM25_ESM.zip › Source Data for Expanded View and Appendix/Figure EV4/4C/mCherry-Merge.tif]

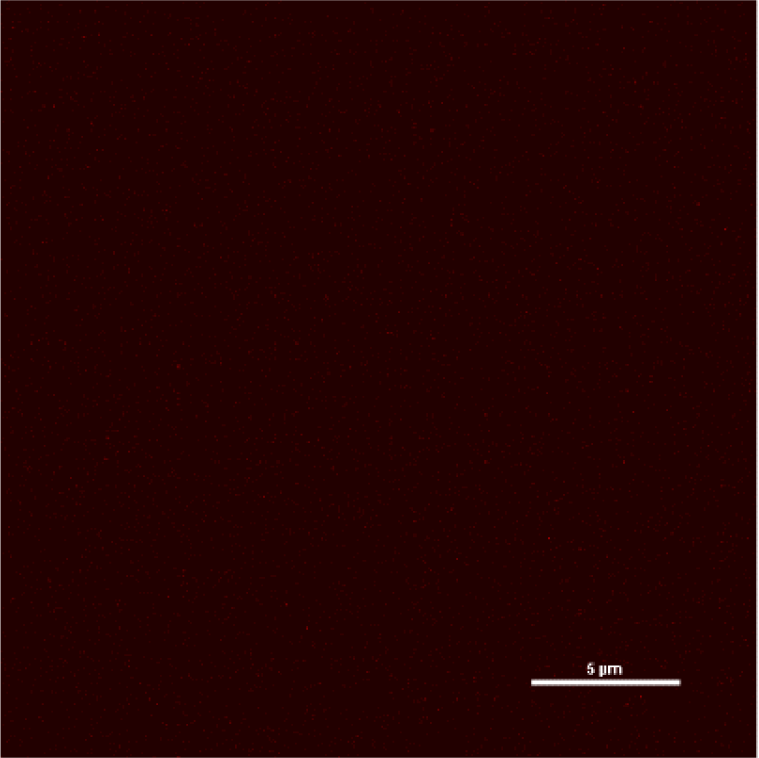

Supplement: Supplementary file 25 — Figure Source Data for Expanded View and Appendix [file 44318_2024_212_MOESM25_ESM.zip › Source Data for Expanded View and Appendix/Figure EV4/4C/mCherry.tif]

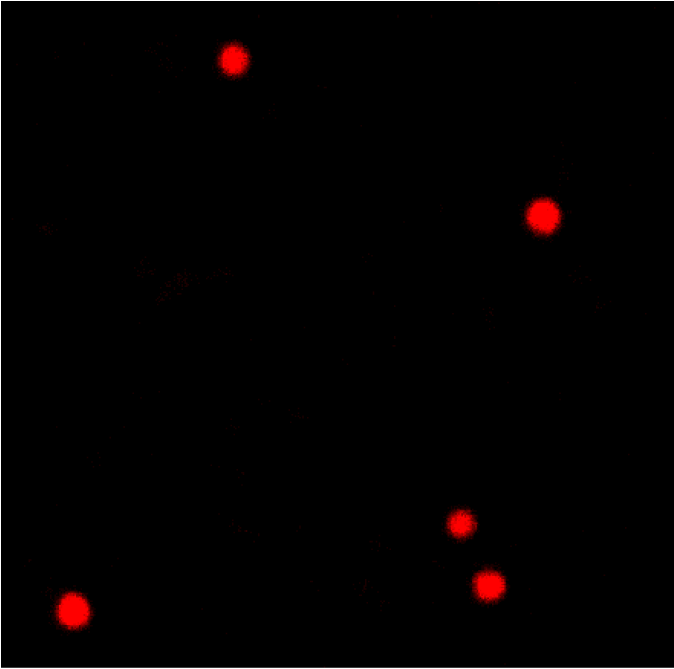

Supplement: Supplementary file 25 — Figure Source Data for Expanded View and Appendix [file 44318_2024_212_MOESM25_ESM.zip › Source Data for Expanded View and Appendix/Figure EV4/4D/20 μM-01.tif]

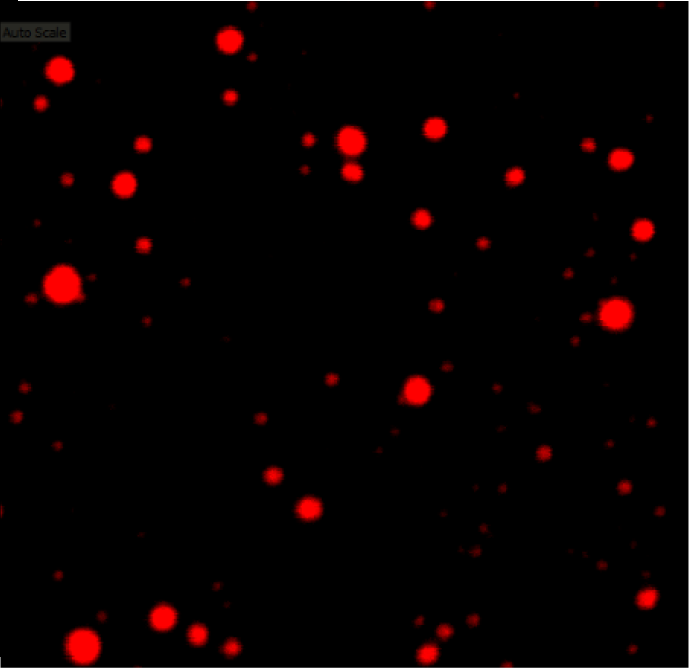

Supplement: Supplementary file 25 — Figure Source Data for Expanded View and Appendix [file 44318_2024_212_MOESM25_ESM.zip › Source Data for Expanded View and Appendix/Figure EV4/4D/40 μM-01.tif]

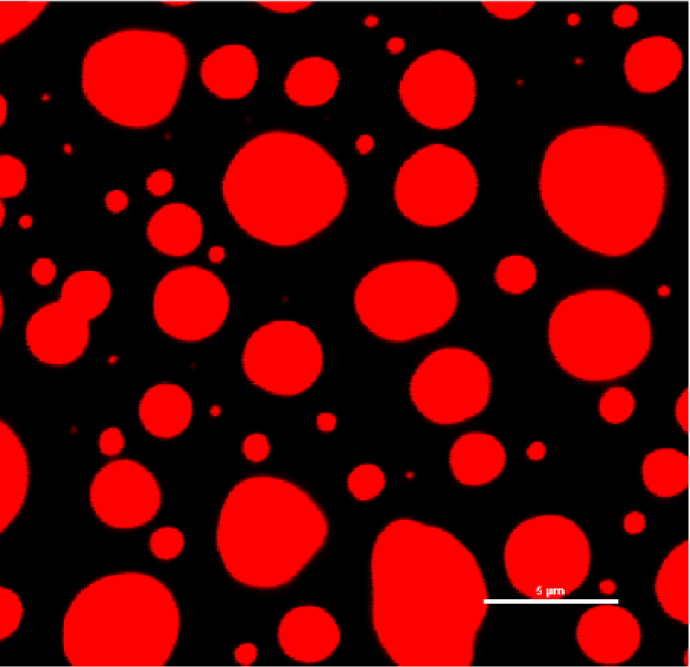

Supplement: Supplementary file 25 — Figure Source Data for Expanded View and Appendix [file 44318_2024_212_MOESM25_ESM.zip › Source Data for Expanded View and Appendix/Figure EV4/4D/80 μM.tif]

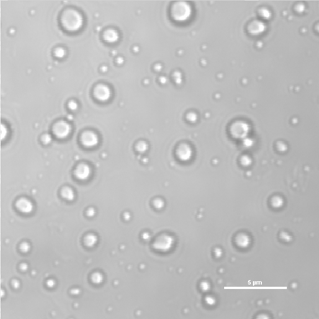

Supplement: Supplementary file 25 — Figure Source Data for Expanded View and Appendix [file 44318_2024_212_MOESM25_ESM.zip › Source Data for Expanded View and Appendix/Figure EV4/4F/His-IRTKS.tif]

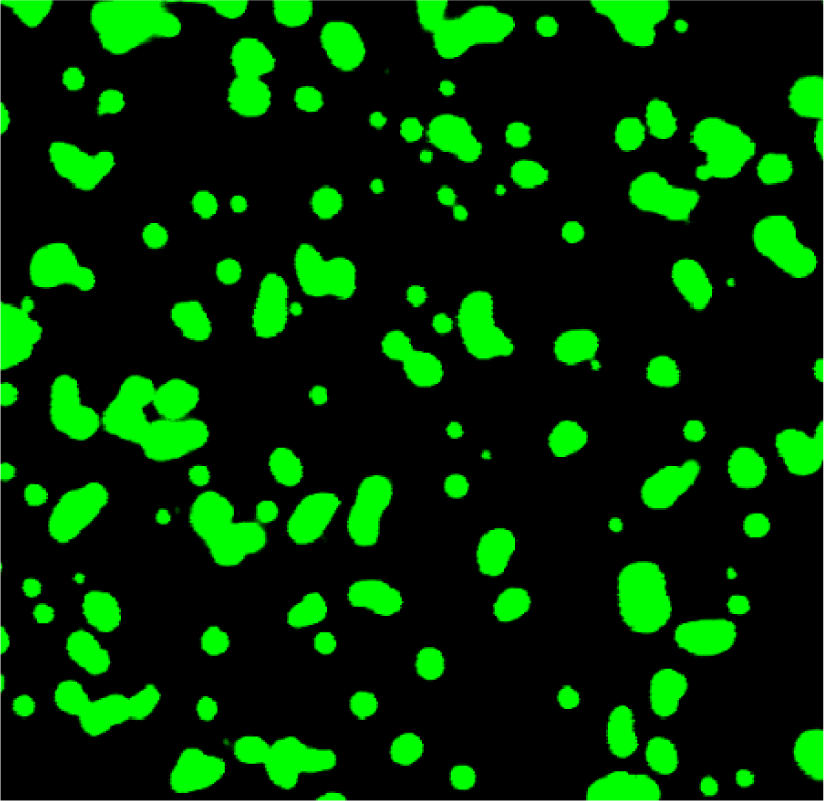

Supplement: Supplementary file 25 — Figure Source Data for Expanded View and Appendix [file 44318_2024_212_MOESM25_ESM.zip › Source Data for Expanded View and Appendix/Figure EV4/4G/10 μM.tif]

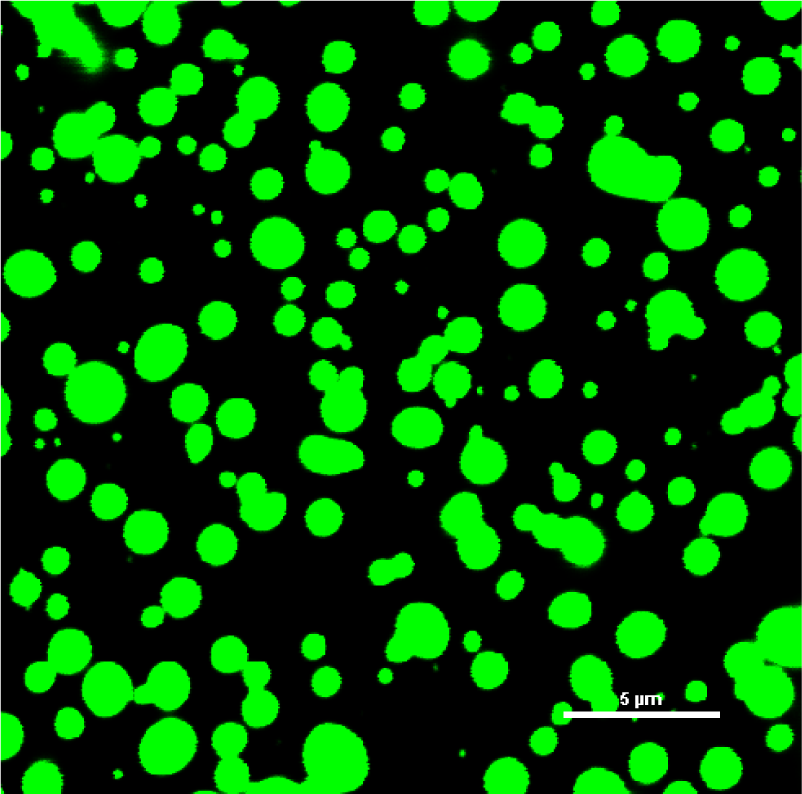

Supplement: Supplementary file 25 — Figure Source Data for Expanded View and Appendix [file 44318_2024_212_MOESM25_ESM.zip › Source Data for Expanded View and Appendix/Figure EV4/4G/20 μM.tif]

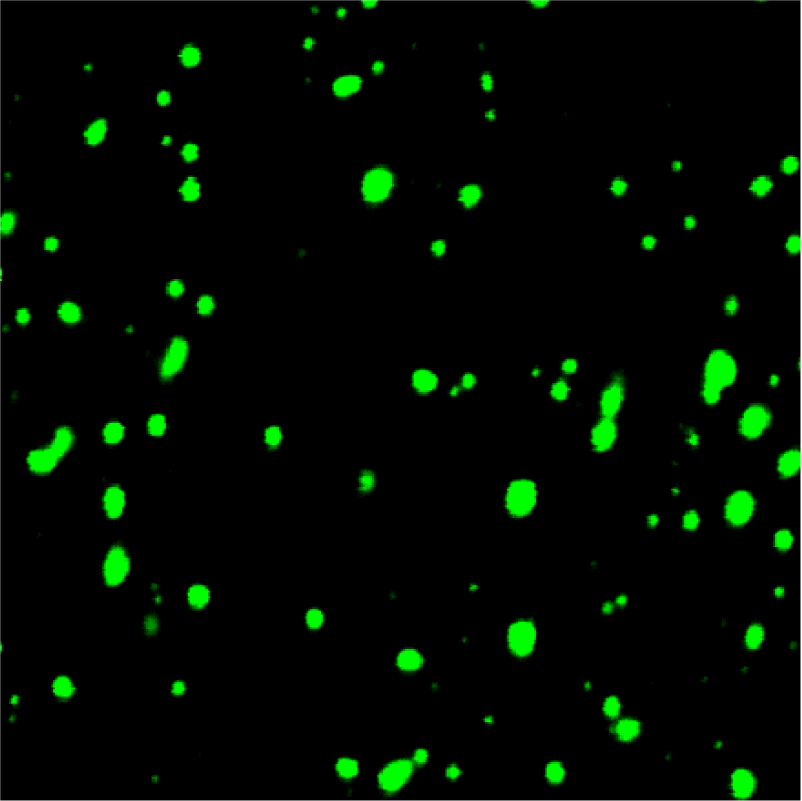

Supplement: Supplementary file 25 — Figure Source Data for Expanded View and Appendix [file 44318_2024_212_MOESM25_ESM.zip › Source Data for Expanded View and Appendix/Figure EV4/4G/5 μM.tif]

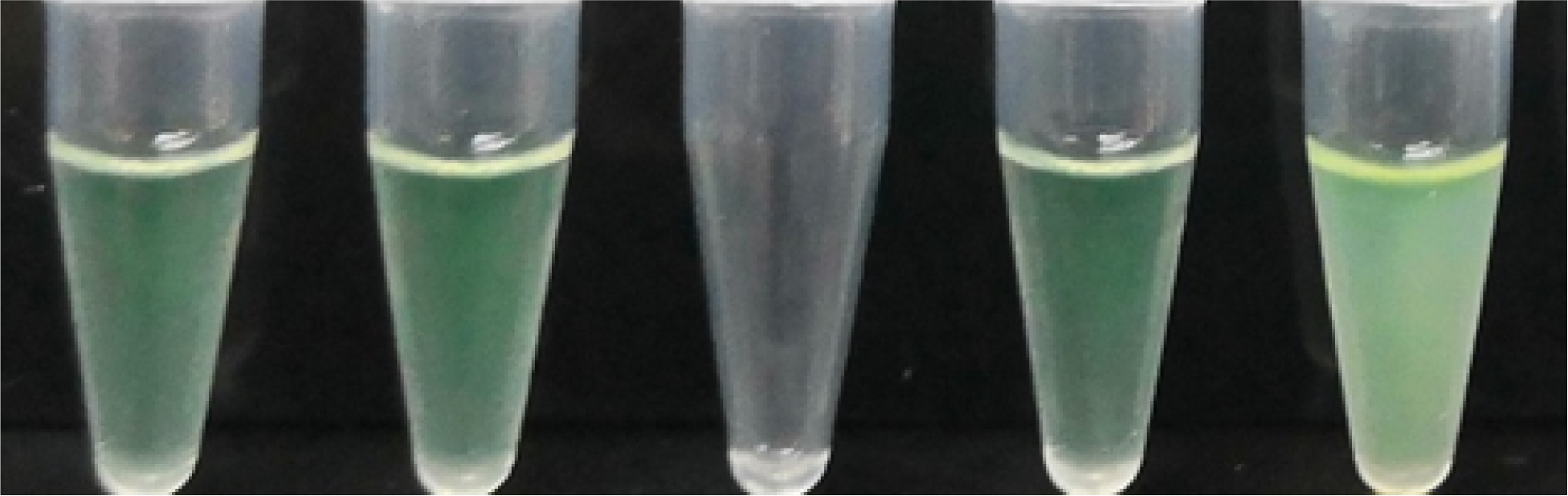

Supplement: Supplementary file 25 — Figure Source Data for Expanded View and Appendix [file 44318_2024_212_MOESM25_ESM.zip › Source Data for Expanded View and Appendix/Figure EV4/4H/EGFP and EGFP-IRTKS.tif]

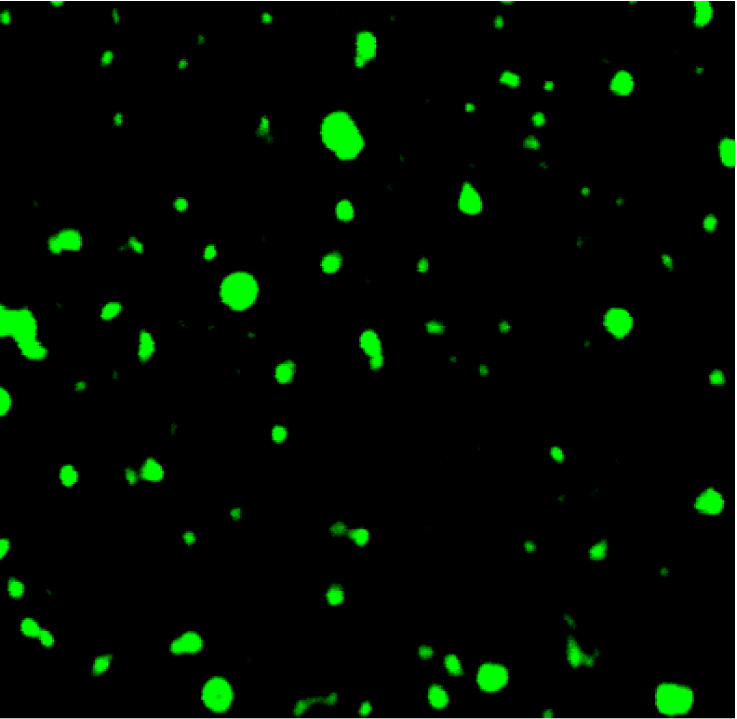

Supplement: Supplementary file 25 — Figure Source Data for Expanded View and Appendix [file 44318_2024_212_MOESM25_ESM.zip › Source Data for Expanded View and Appendix/Figure EV4/4I/150 mM.tif]

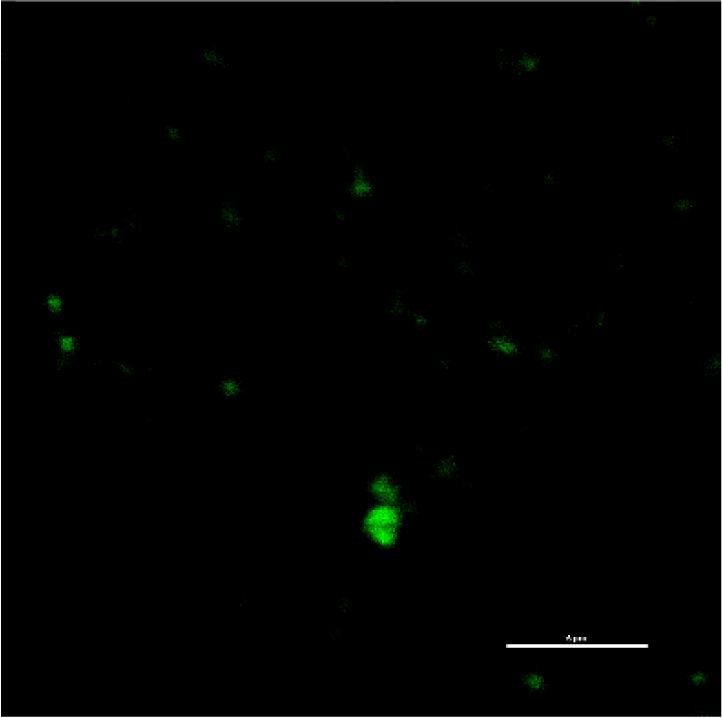

Supplement: Supplementary file 25 — Figure Source Data for Expanded View and Appendix [file 44318_2024_212_MOESM25_ESM.zip › Source Data for Expanded View and Appendix/Figure EV4/4I/300 mM.tif]

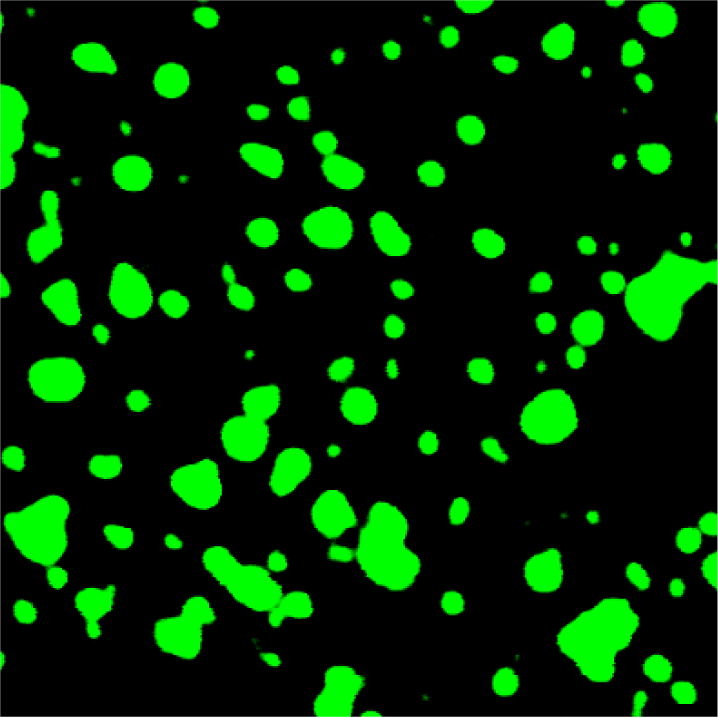

Supplement: Supplementary file 25 — Figure Source Data for Expanded View and Appendix [file 44318_2024_212_MOESM25_ESM.zip › Source Data for Expanded View and Appendix/Figure EV4/4I/50 mM.tif]

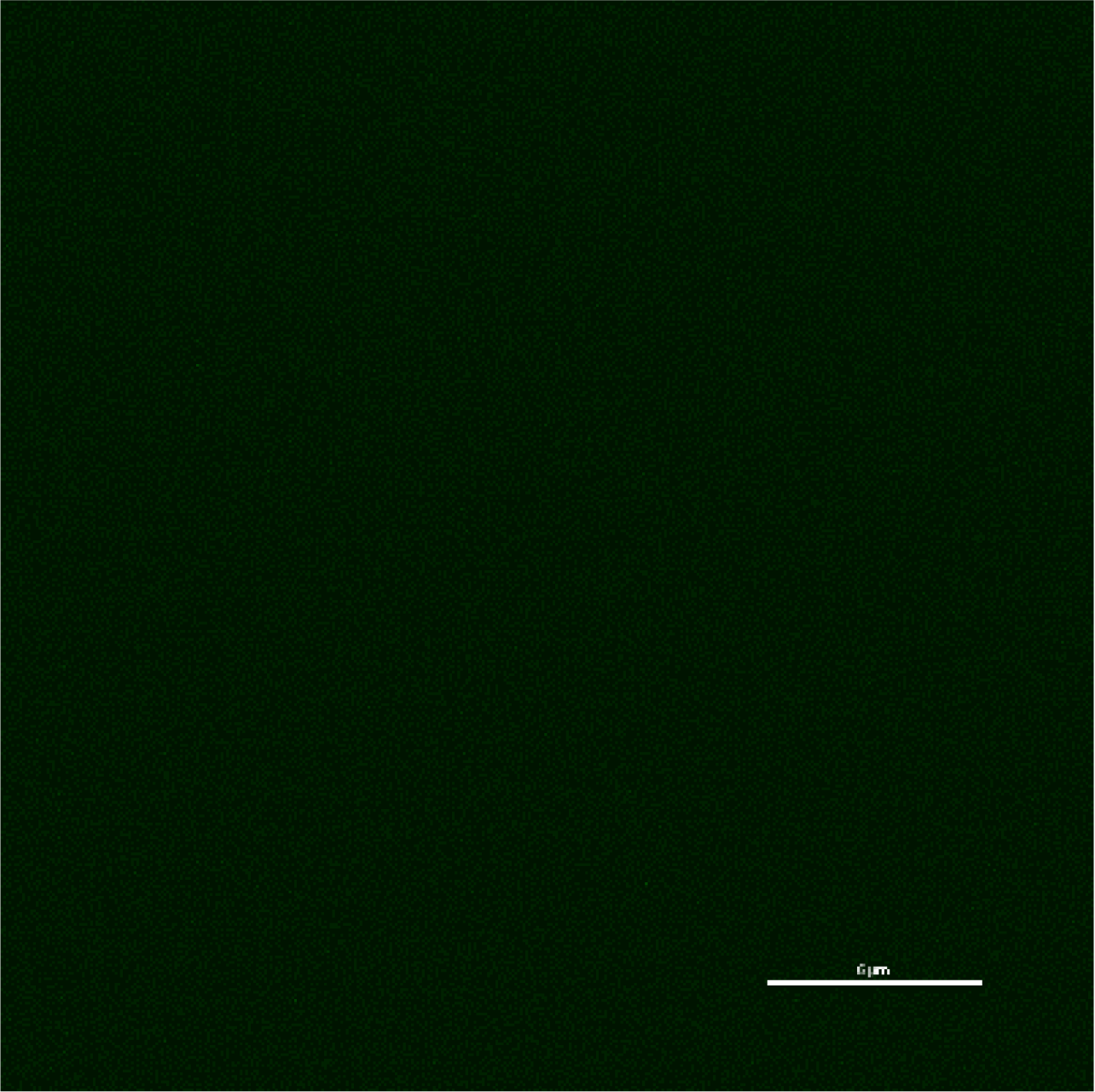

Supplement: Supplementary file 25 — Figure Source Data for Expanded View and Appendix [file 44318_2024_212_MOESM25_ESM.zip › Source Data for Expanded View and Appendix/Figure EV4/4K/D1.tif]

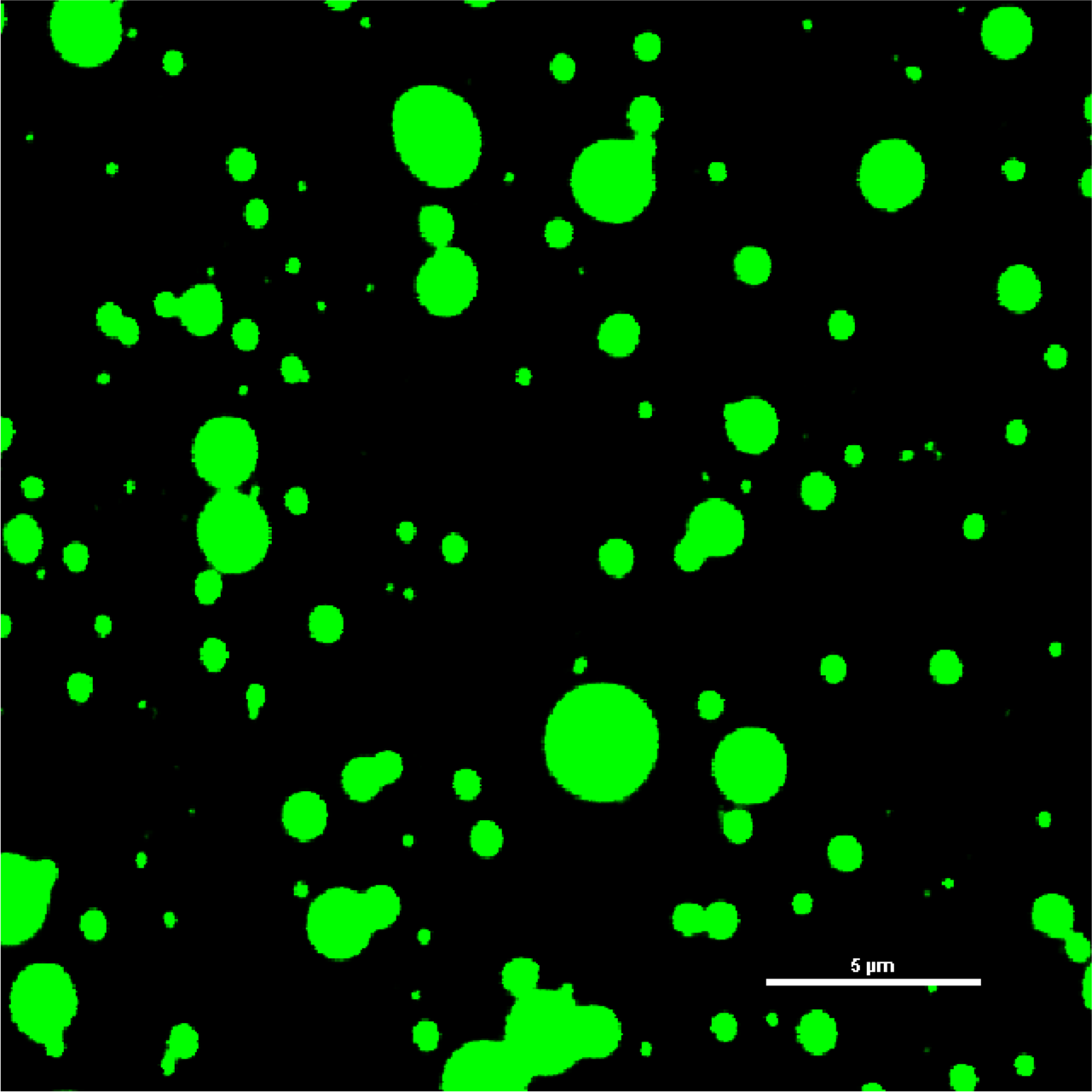

Supplement: Supplementary file 25 — Figure Source Data for Expanded View and Appendix [file 44318_2024_212_MOESM25_ESM.zip › Source Data for Expanded View and Appendix/Figure EV4/4K/D2.tif]

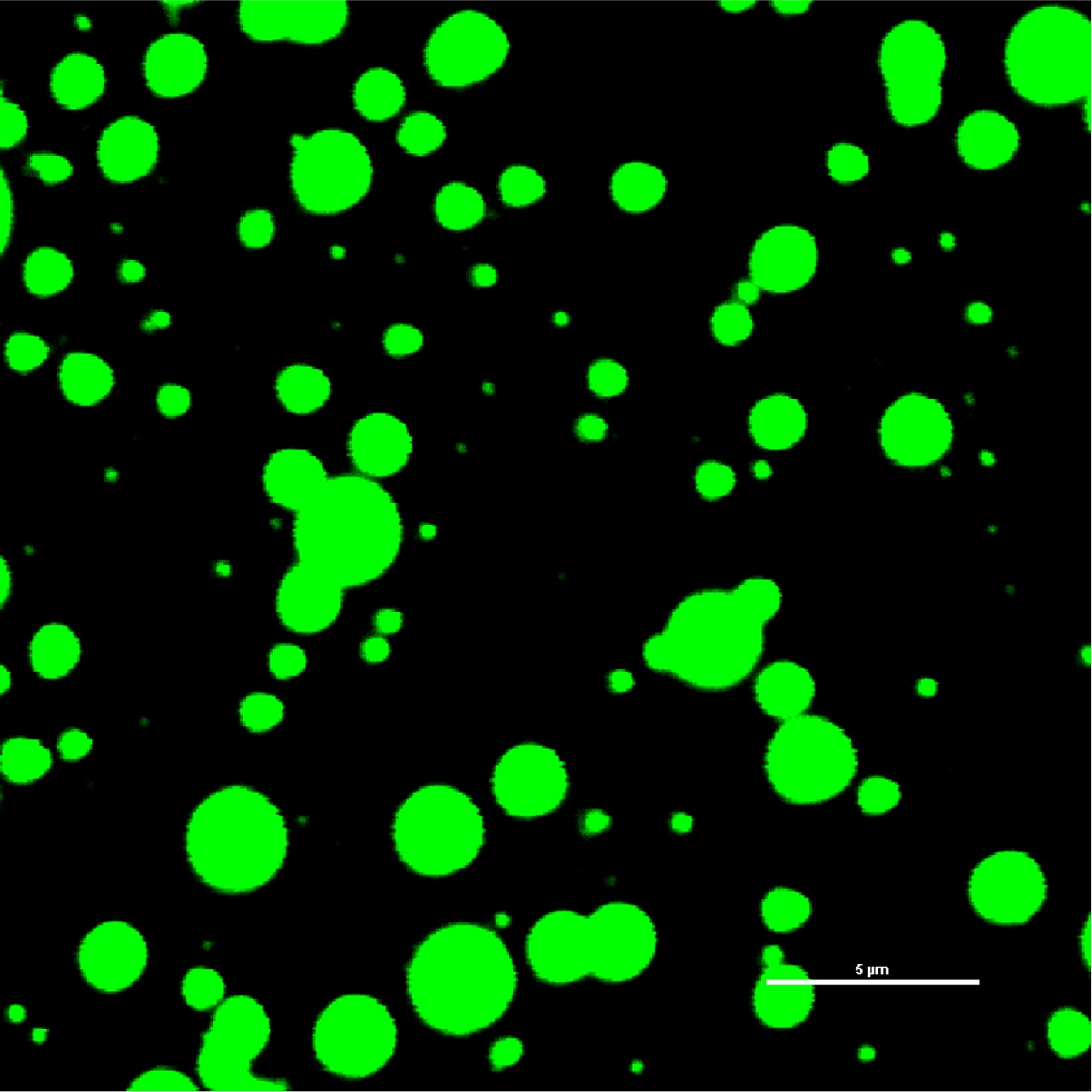

Supplement: Supplementary file 25 — Figure Source Data for Expanded View and Appendix [file 44318_2024_212_MOESM25_ESM.zip › Source Data for Expanded View and Appendix/Figure EV4/4K/D3.tif]

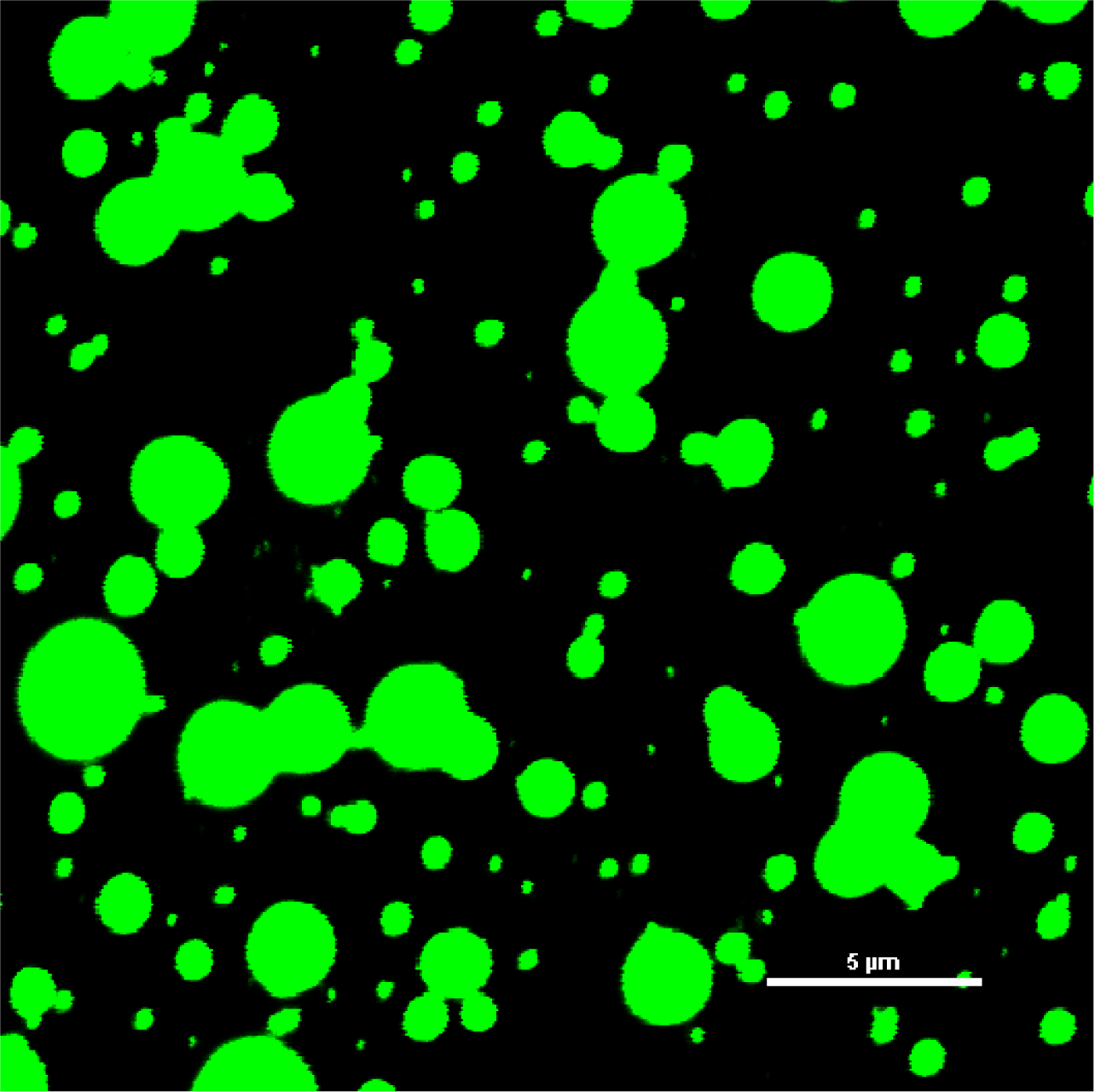

Supplement: Supplementary file 25 — Figure Source Data for Expanded View and Appendix [file 44318_2024_212_MOESM25_ESM.zip › Source Data for Expanded View and Appendix/Figure EV4/4K/WT.tif]

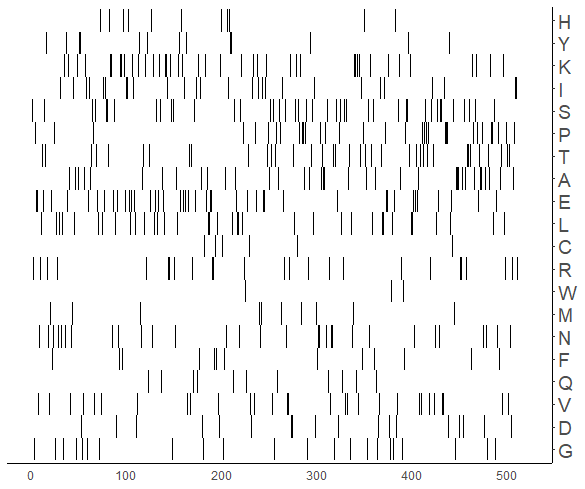

Supplement: Supplementary file 25 — Figure Source Data for Expanded View and Appendix [file 44318_2024_212_MOESM25_ESM.zip › Source Data for Expanded View and Appendix/Figure EV4/4L/IRTKS.tiff]

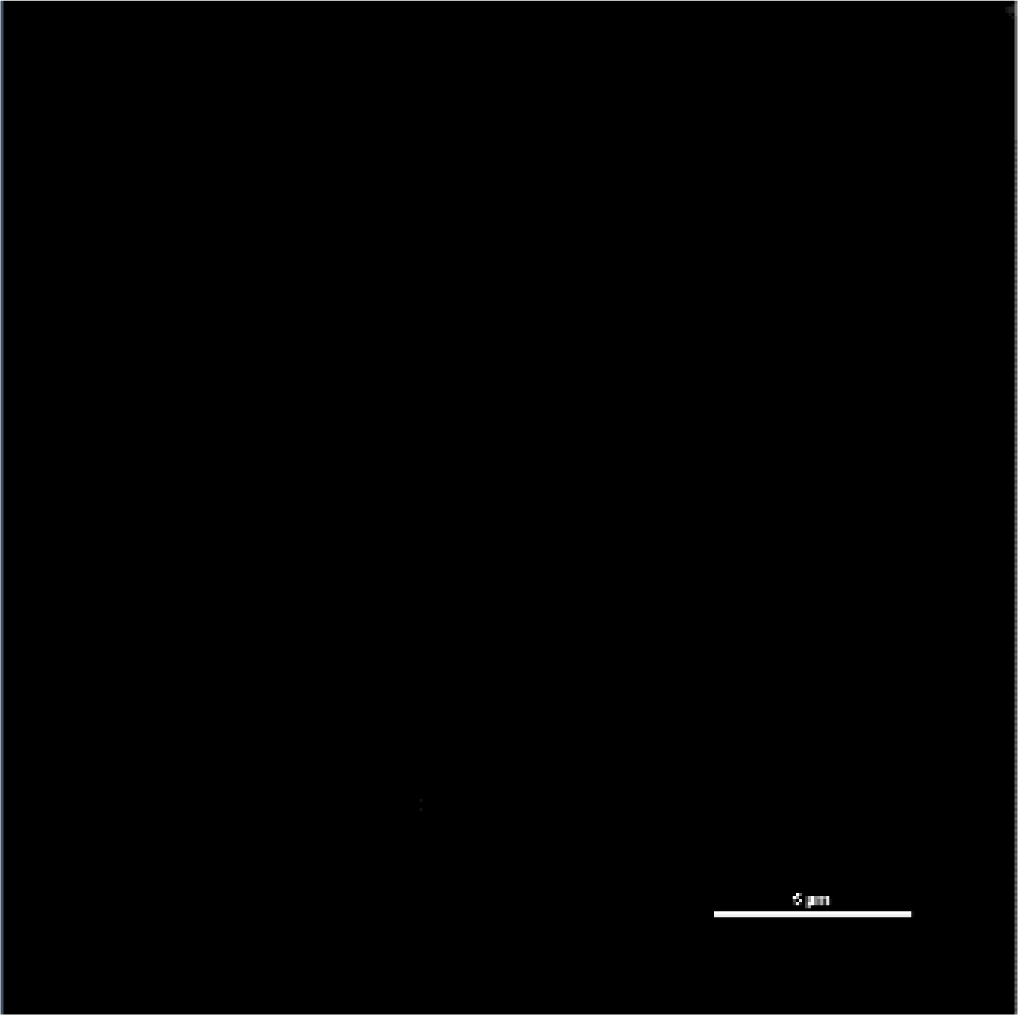

Supplement: Supplementary file 25 — Figure Source Data for Expanded View and Appendix [file 44318_2024_212_MOESM25_ESM.zip › Source Data for Expanded View and Appendix/Figure EV4/4N/IDR-WT-Mut.tif]

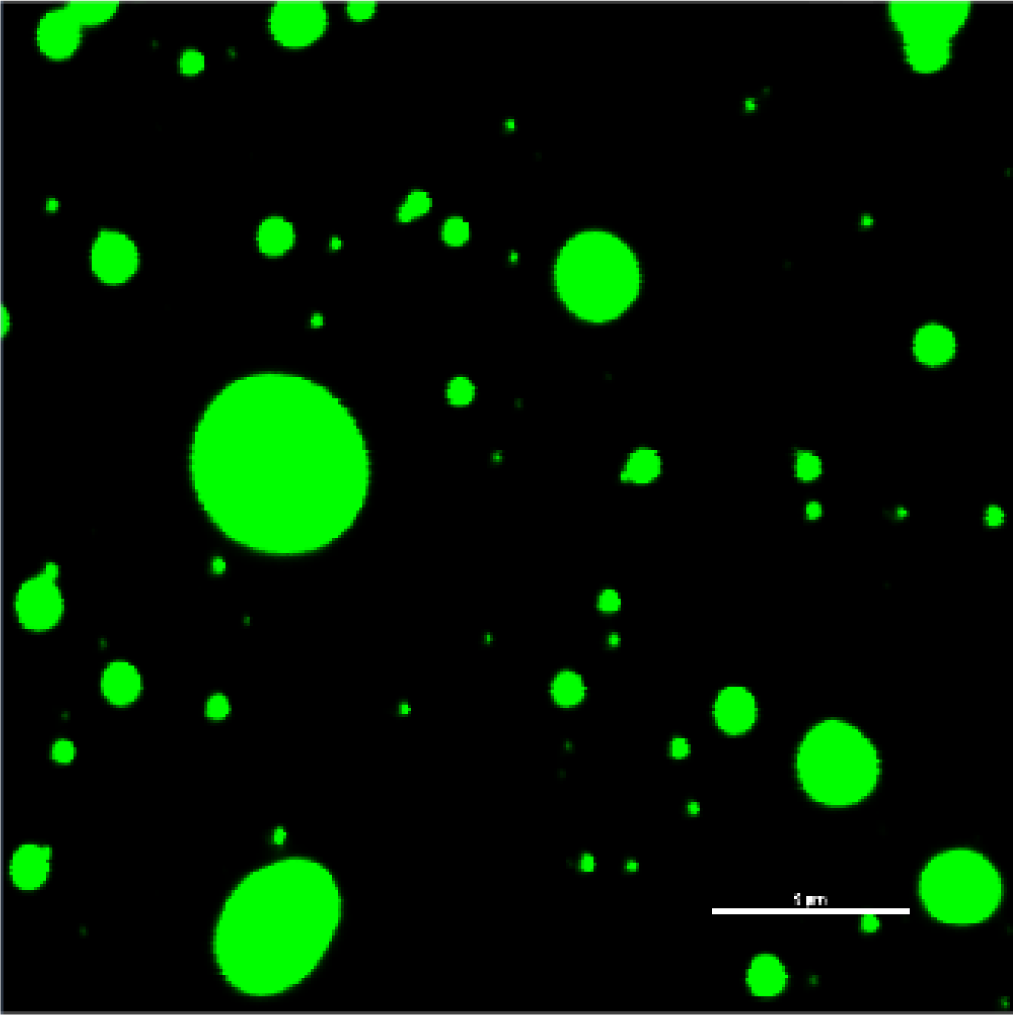

Supplement: Supplementary file 25 — Figure Source Data for Expanded View and Appendix [file 44318_2024_212_MOESM25_ESM.zip › Source Data for Expanded View and Appendix/Figure EV4/4N/IDR-WT.tif]

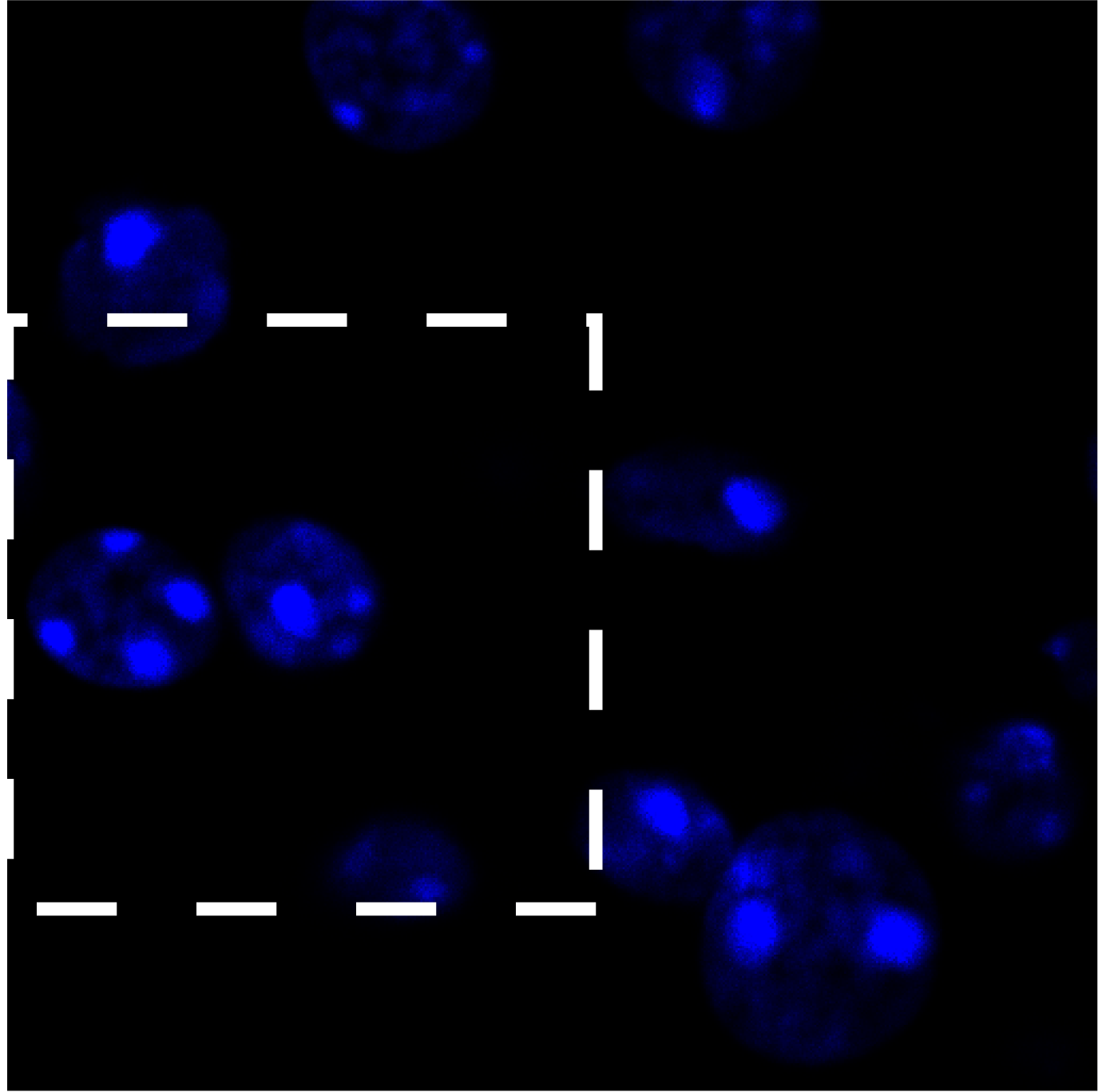

Supplement: Supplementary file 25 — Figure Source Data for Expanded View and Appendix [file 44318_2024_212_MOESM25_ESM.zip › Source Data for Expanded View and Appendix/Figure EV4/4P/KO-DAPI.tif]

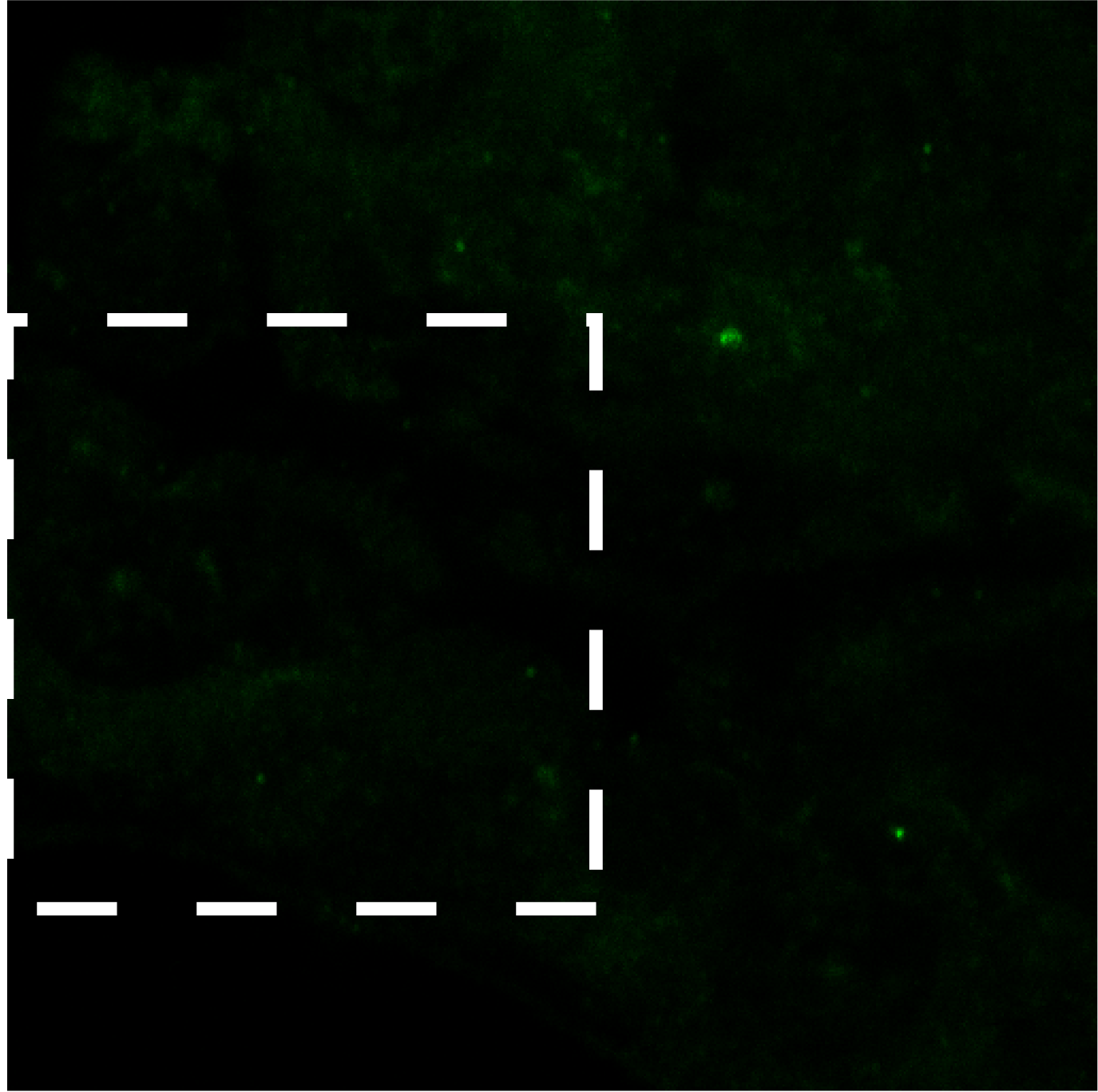

Supplement: Supplementary file 25 — Figure Source Data for Expanded View and Appendix [file 44318_2024_212_MOESM25_ESM.zip › Source Data for Expanded View and Appendix/Figure EV4/4P/KO-IRTKS.tif]

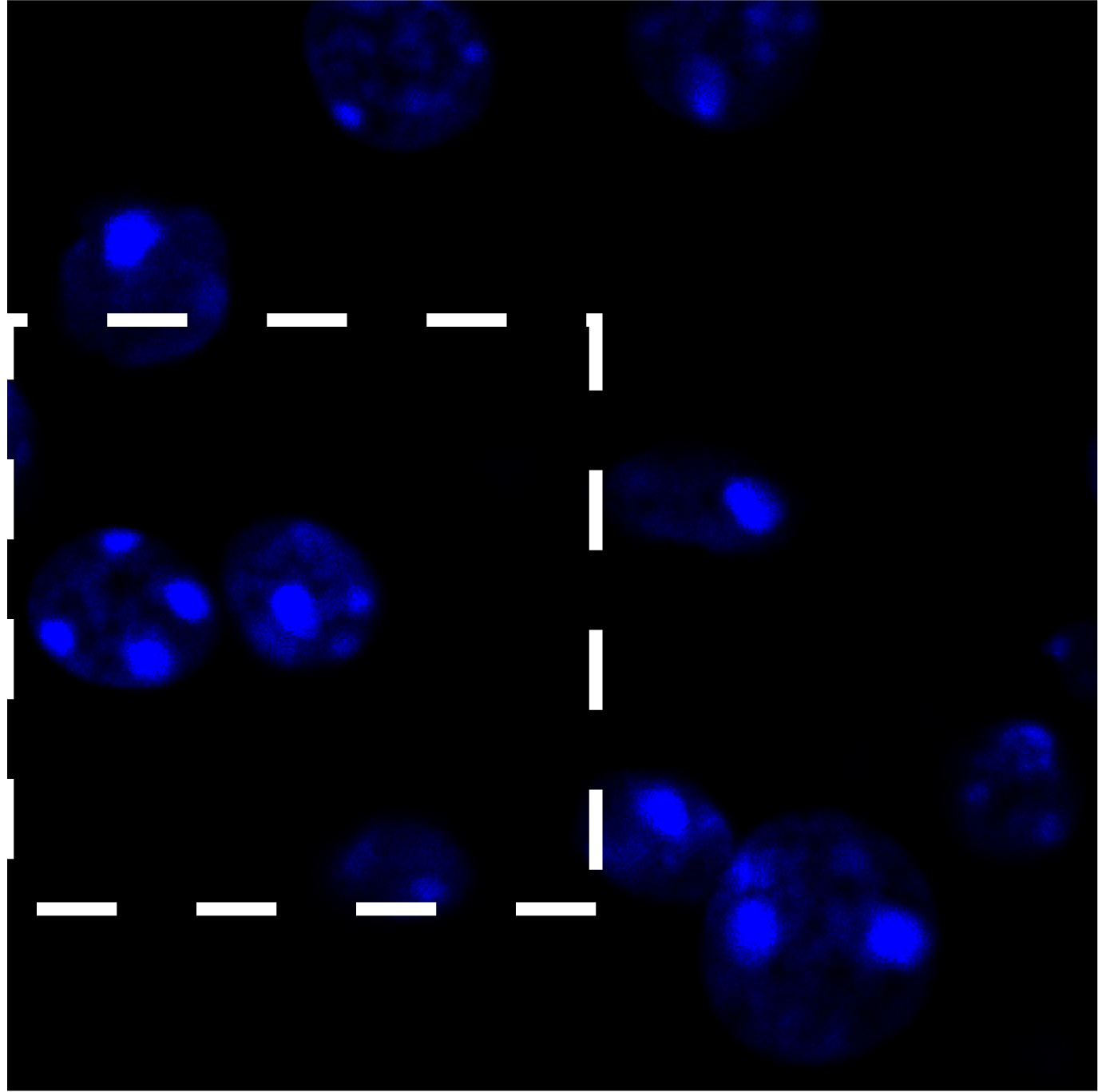

Supplement: Supplementary file 25 — Figure Source Data for Expanded View and Appendix [file 44318_2024_212_MOESM25_ESM.zip › Source Data for Expanded View and Appendix/Figure EV4/4P/KO-Merge.tif]

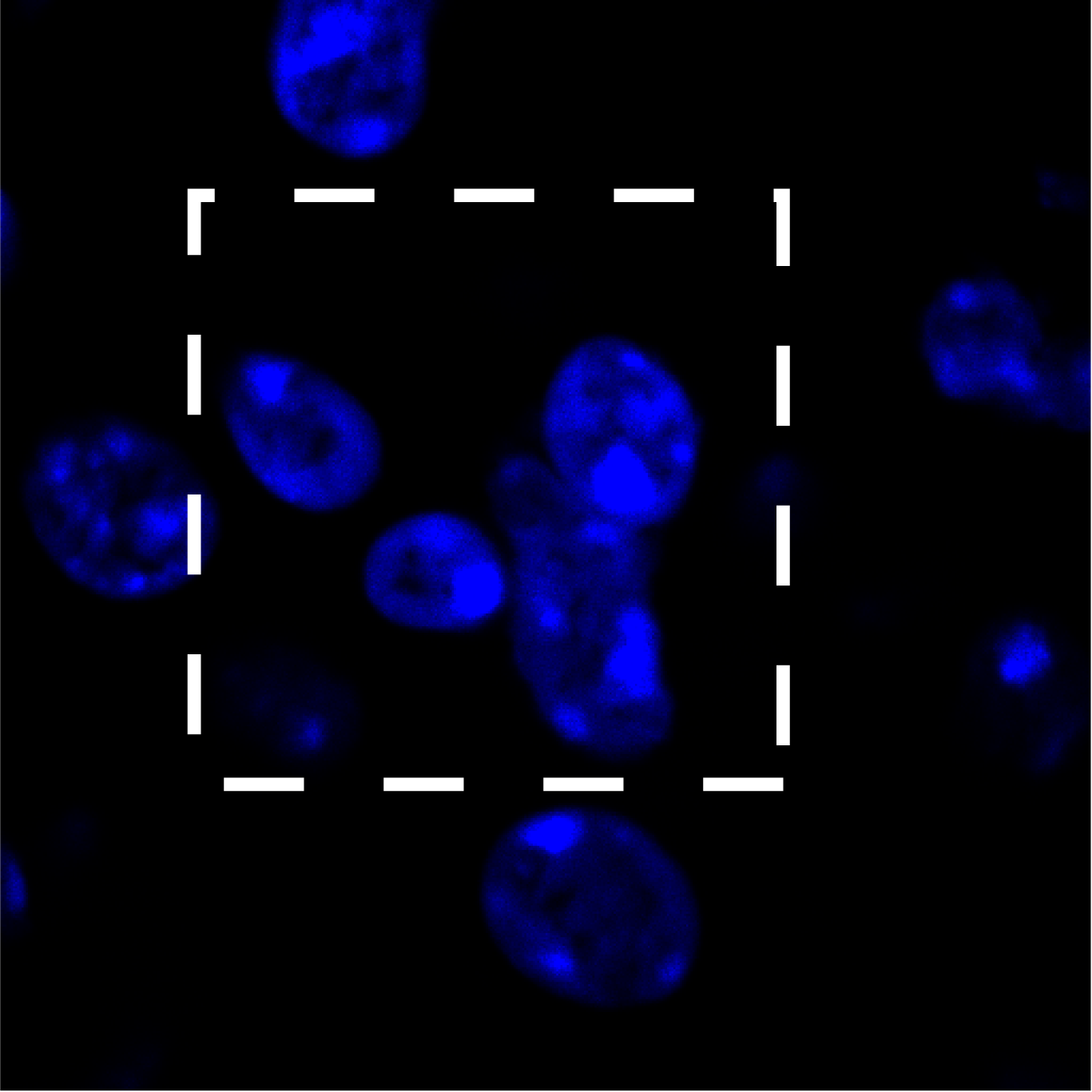

Supplement: Supplementary file 25 — Figure Source Data for Expanded View and Appendix [file 44318_2024_212_MOESM25_ESM.zip › Source Data for Expanded View and Appendix/Figure EV4/4P/WT-DAPI.tif]

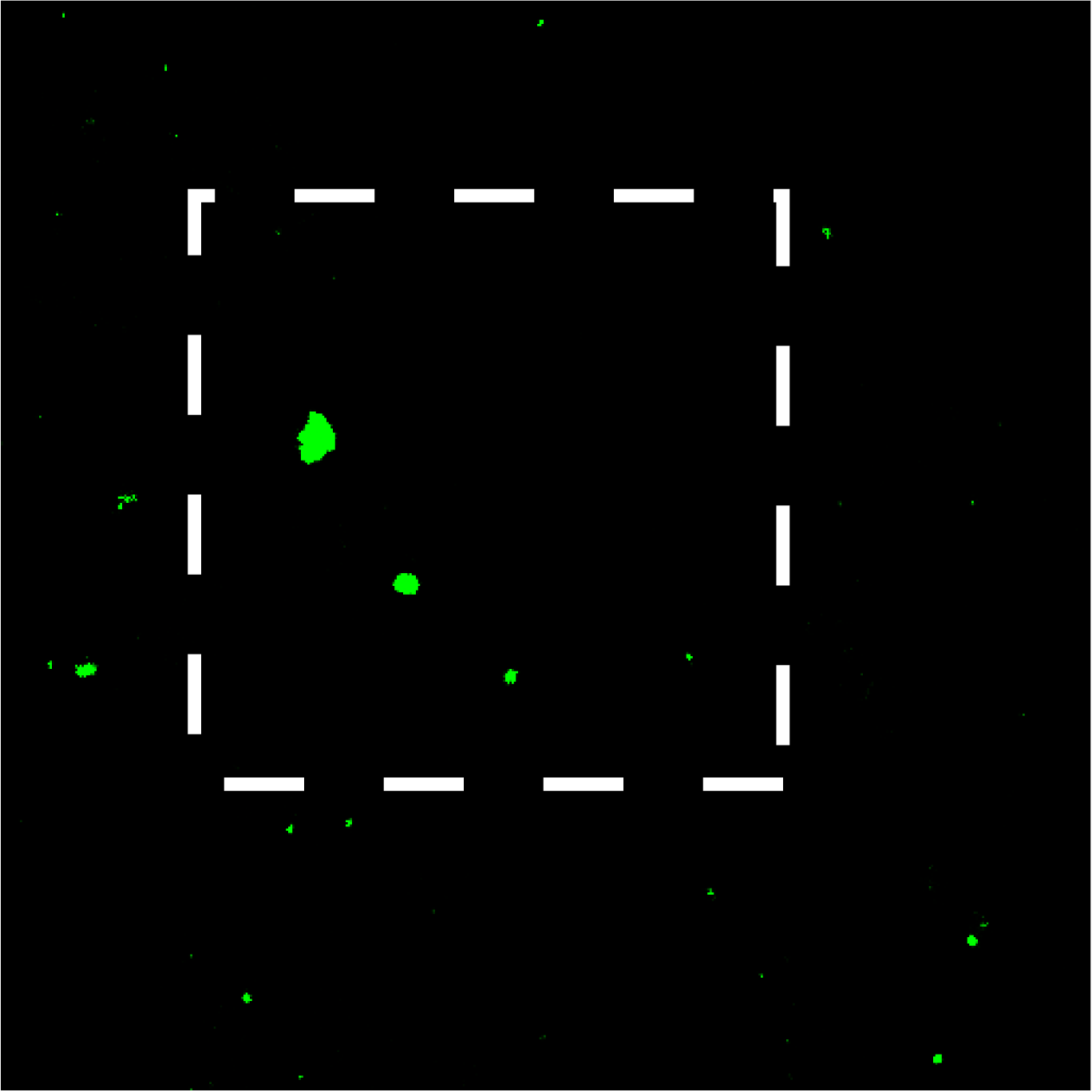

Supplement: Supplementary file 25 — Figure Source Data for Expanded View and Appendix [file 44318_2024_212_MOESM25_ESM.zip › Source Data for Expanded View and Appendix/Figure EV4/4P/WT-IRTKS.tif]

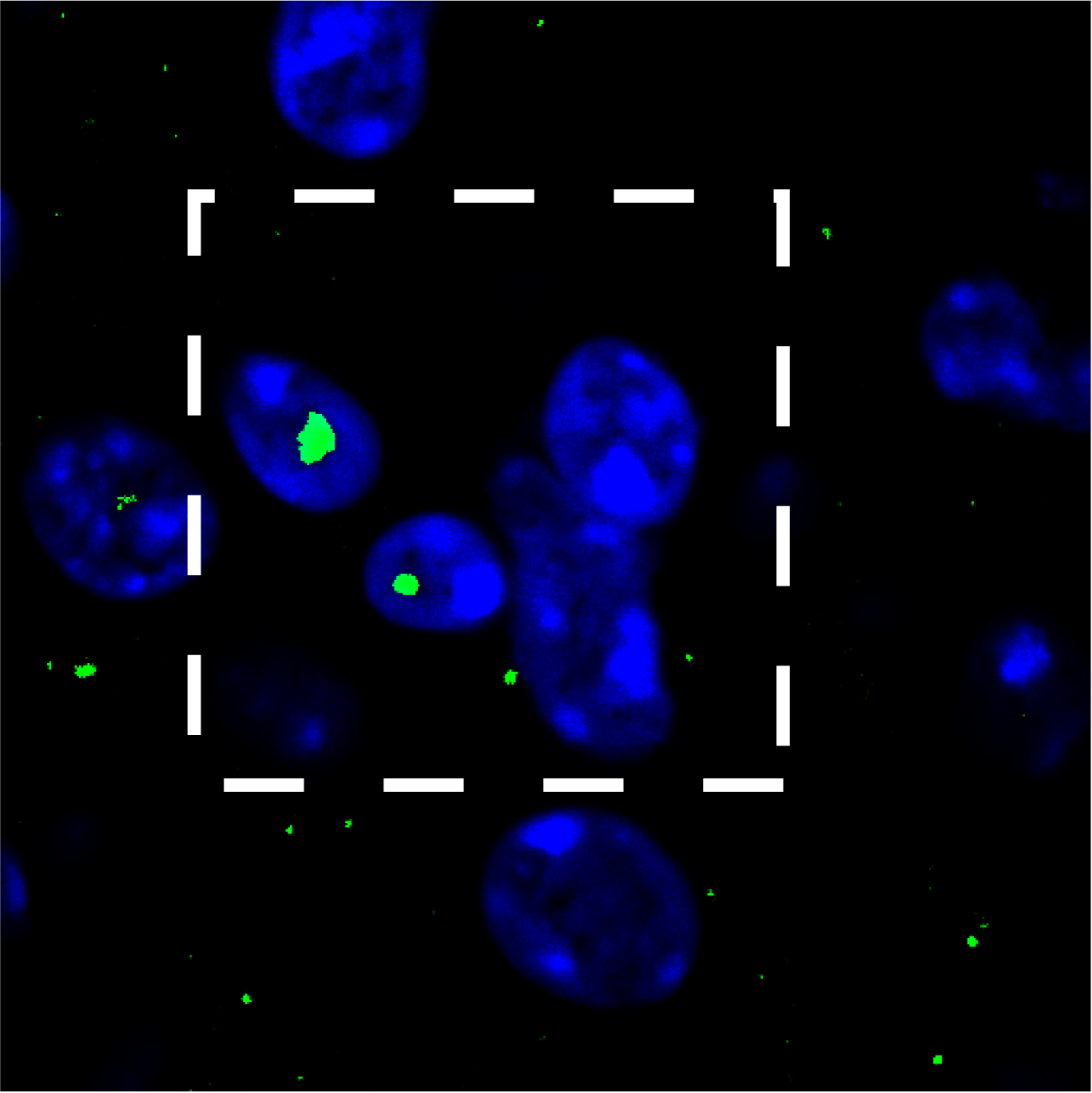

Supplement: Supplementary file 25 — Figure Source Data for Expanded View and Appendix [file 44318_2024_212_MOESM25_ESM.zip › Source Data for Expanded View and Appendix/Figure EV4/4P/WT-Merge.tif]

**Fig. EV4B**

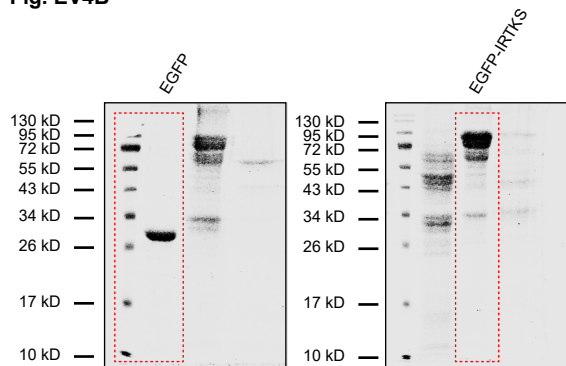

**Fig. EV4E**

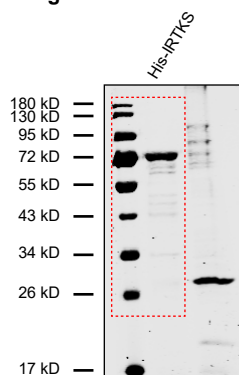

**Fig. EV4J**

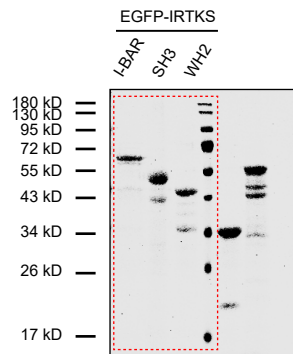

**Fig. EV4T**

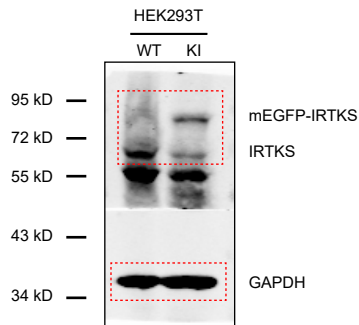

Supplement: Supplementary file 25 — Figure Source Data for Expanded View and Appendix [file 44318_2024_212_MOESM25_ESM.zip › Source Data for Expanded View and Appendix/Figure EV4/Source DataFig. EV4.pdf]

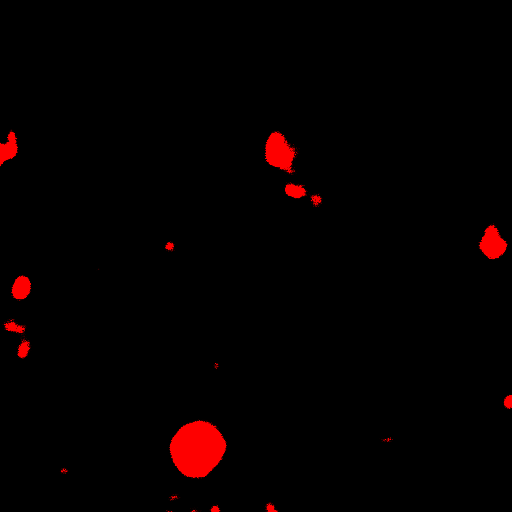

Supplement: Supplementary file 25 — Figure Source Data for Expanded View and Appendix [file 44318_2024_212_MOESM25_ESM.zip › Source Data for Expanded View and Appendix/Figure EV5/5A/0 irtks 20 hp1a 500x 2.tif]

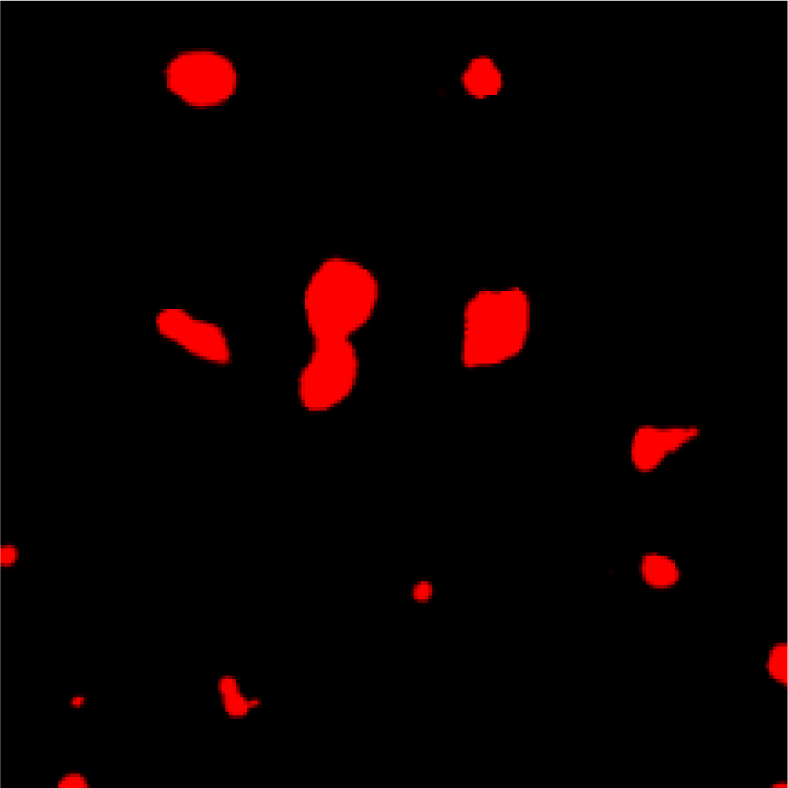

Supplement: Supplementary file 25 — Figure Source Data for Expanded View and Appendix [file 44318_2024_212_MOESM25_ESM.zip › Source Data for Expanded View and Appendix/Figure EV5/5A/0 irtks 40 hp1a 500x 2.tif]

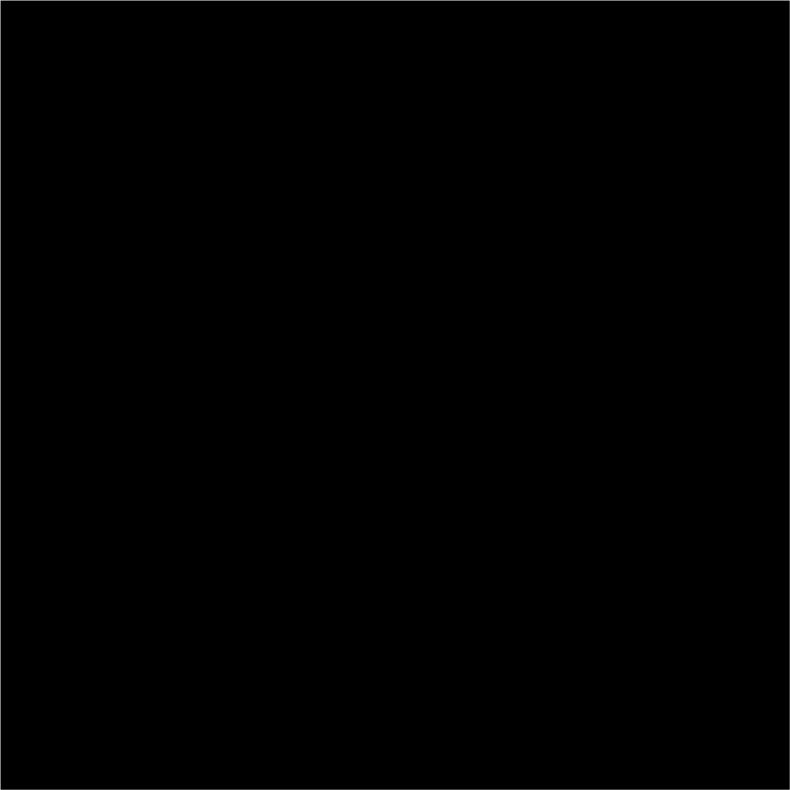

Supplement: Supplementary file 25 — Figure Source Data for Expanded View and Appendix [file 44318_2024_212_MOESM25_ESM.zip › Source Data for Expanded View and Appendix/Figure EV5/5A/0 irtks 0 hp1a 500x 2.tif]

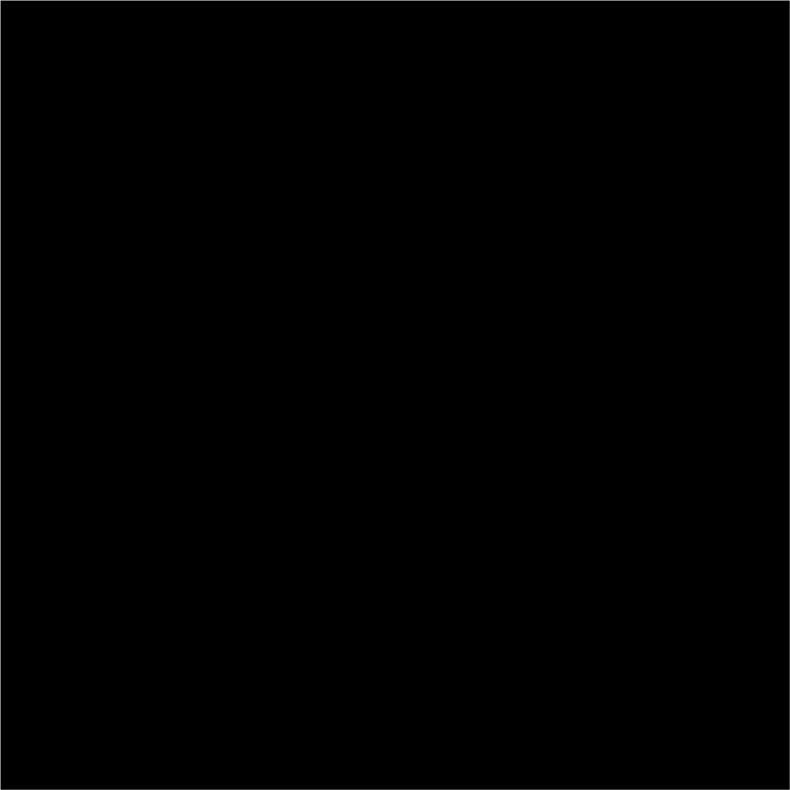

Supplement: Supplementary file 25 — Figure Source Data for Expanded View and Appendix [file 44318_2024_212_MOESM25_ESM.zip › Source Data for Expanded View and Appendix/Figure EV5/5A/0 irtks 0 hp1a 500x 2c1.tif]

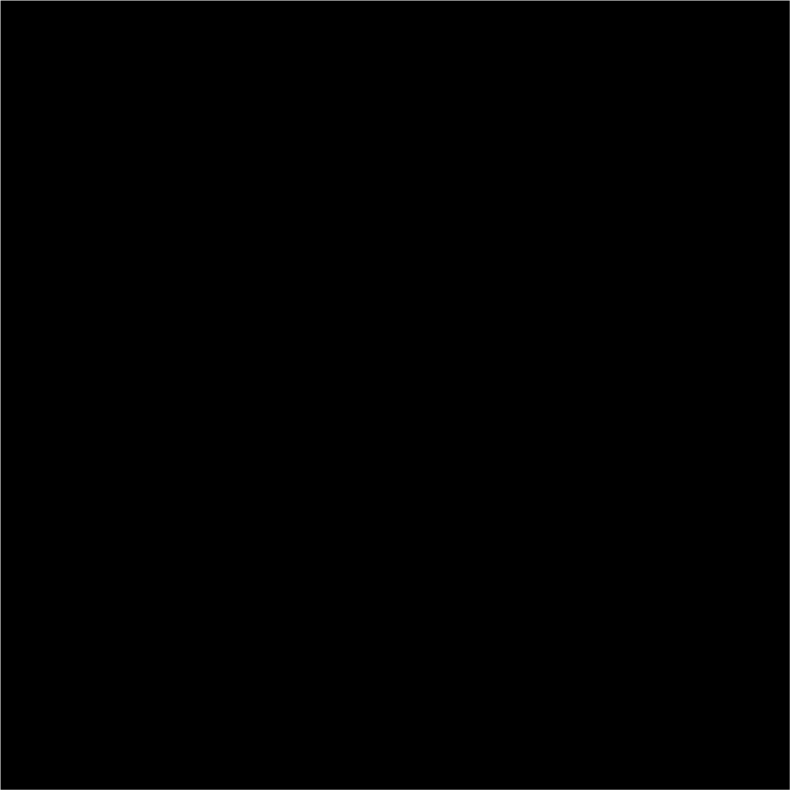

Supplement: Supplementary file 25 — Figure Source Data for Expanded View and Appendix [file 44318_2024_212_MOESM25_ESM.zip › Source Data for Expanded View and Appendix/Figure EV5/5A/0 irtks 0 hp1a 500x 2c2.tif]

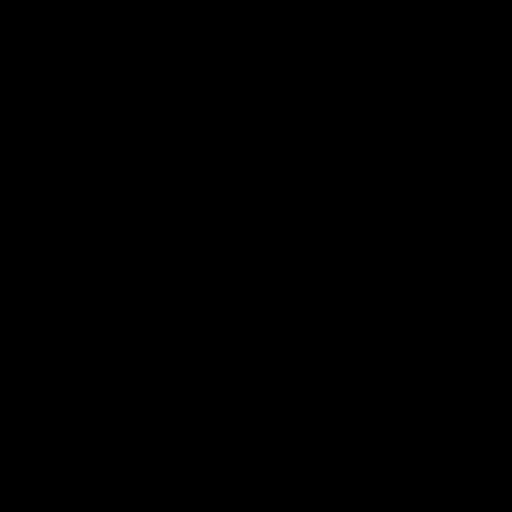

Supplement: Supplementary file 25 — Figure Source Data for Expanded View and Appendix [file 44318_2024_212_MOESM25_ESM.zip › Source Data for Expanded View and Appendix/Figure EV5/5A/0 irtks 20 hp1a 500x 2c1.tif]

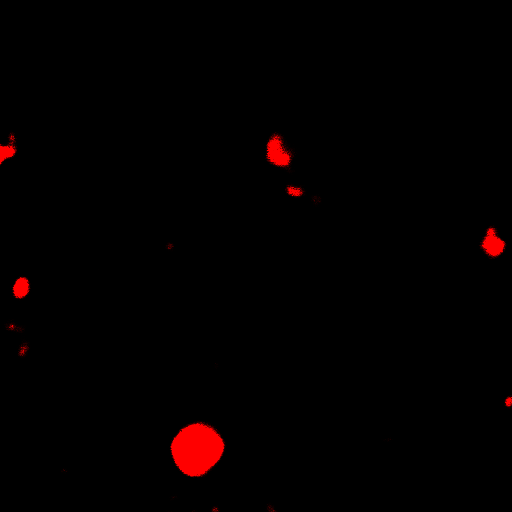

Supplement: Supplementary file 25 — Figure Source Data for Expanded View and Appendix [file 44318_2024_212_MOESM25_ESM.zip › Source Data for Expanded View and Appendix/Figure EV5/5A/0 irtks 20 hp1a 500x 2c2.tif]

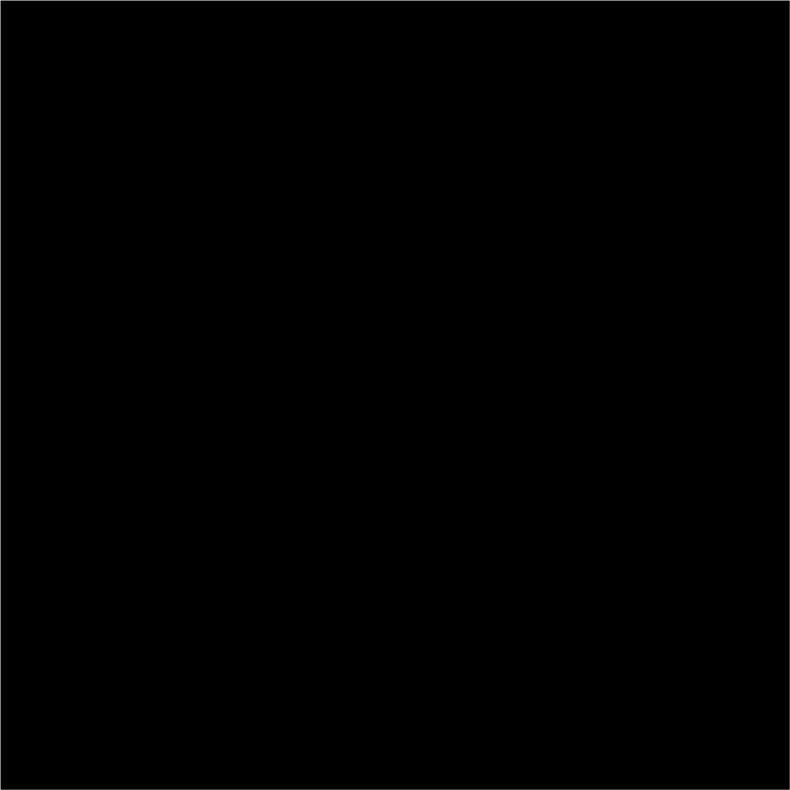

Supplement: Supplementary file 25 — Figure Source Data for Expanded View and Appendix [file 44318_2024_212_MOESM25_ESM.zip › Source Data for Expanded View and Appendix/Figure EV5/5A/0 irtks 40 hp1a 500x 2c1.tif]

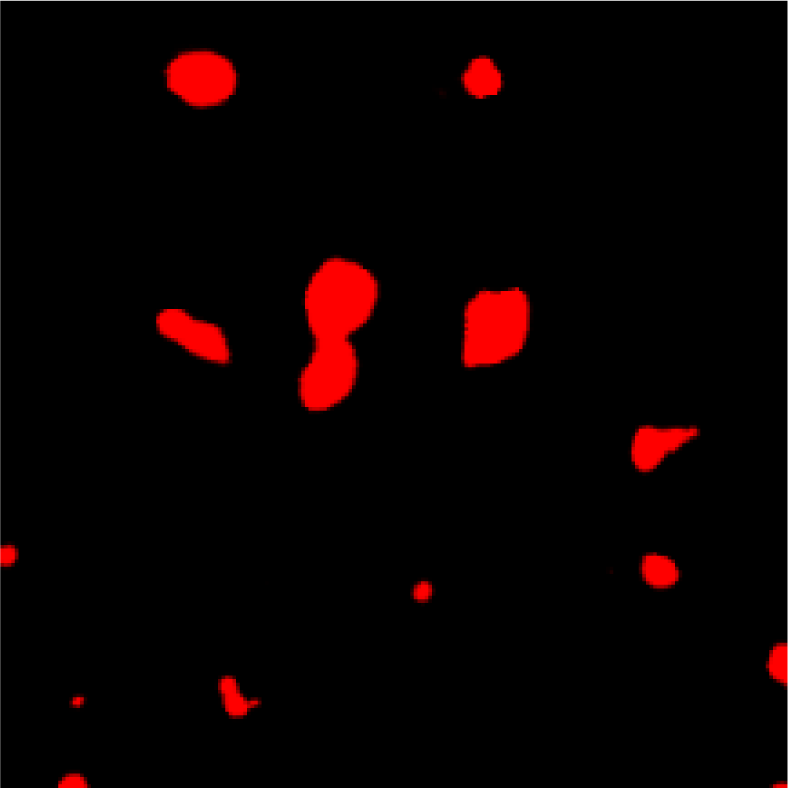

Supplement: Supplementary file 25 — Figure Source Data for Expanded View and Appendix [file 44318_2024_212_MOESM25_ESM.zip › Source Data for Expanded View and Appendix/Figure EV5/5A/0 irtks 40 hp1a 500x 2c2.tif]

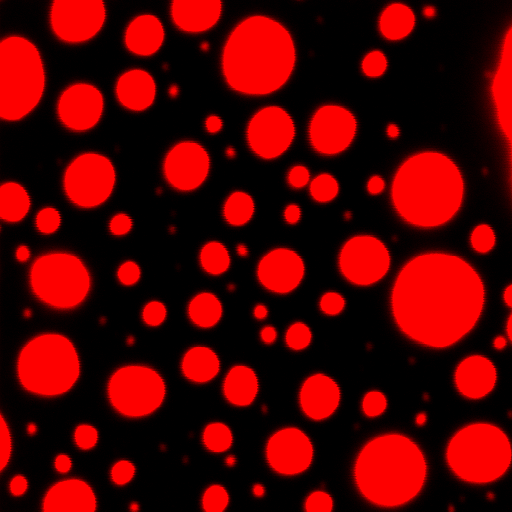

Supplement: Supplementary file 25 — Figure Source Data for Expanded View and Appendix [file 44318_2024_212_MOESM25_ESM.zip › Source Data for Expanded View and Appendix/Figure EV5/5A/0 irtks 80 hp1a 500x 1.tif]

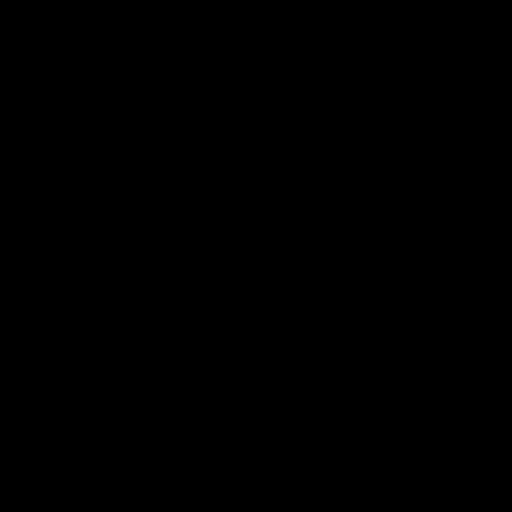

Supplement: Supplementary file 25 — Figure Source Data for Expanded View and Appendix [file 44318_2024_212_MOESM25_ESM.zip › Source Data for Expanded View and Appendix/Figure EV5/5A/0 irtks 80 hp1a 500x 1c1.tif]

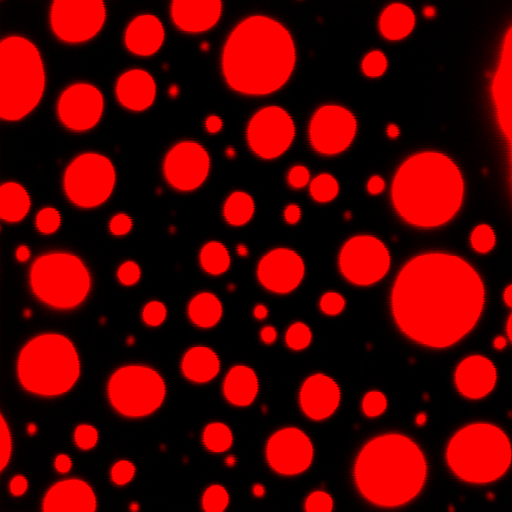

Supplement: Supplementary file 25 — Figure Source Data for Expanded View and Appendix [file 44318_2024_212_MOESM25_ESM.zip › Source Data for Expanded View and Appendix/Figure EV5/5A/0 irtks 80 hp1a 500x 1c2.tif]

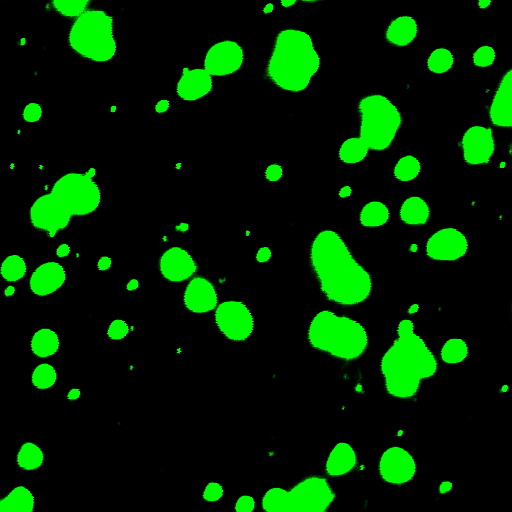

Supplement: Supplementary file 25 — Figure Source Data for Expanded View and Appendix [file 44318_2024_212_MOESM25_ESM.zip › Source Data for Expanded View and Appendix/Figure EV5/5A/10 irtks 0 hp1a 500x 4.tif]

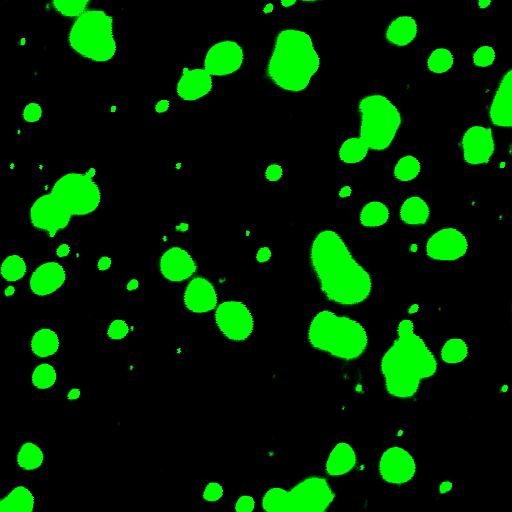

Supplement: Supplementary file 25 — Figure Source Data for Expanded View and Appendix [file 44318_2024_212_MOESM25_ESM.zip › Source Data for Expanded View and Appendix/Figure EV5/5A/10 irtks 0 hp1a 500x 4c1.tif]

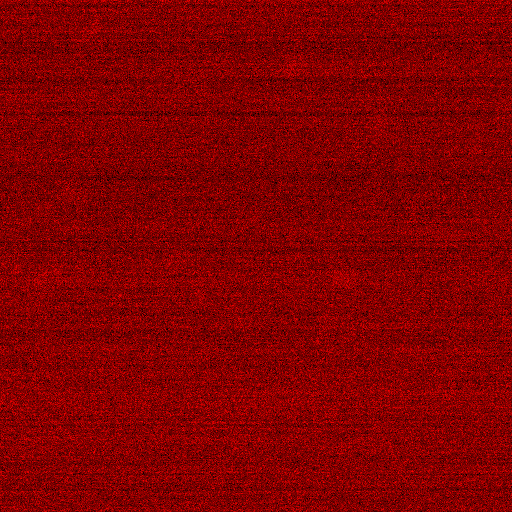

Supplement: Supplementary file 25 — Figure Source Data for Expanded View and Appendix [file 44318_2024_212_MOESM25_ESM.zip › Source Data for Expanded View and Appendix/Figure EV5/5A/10 irtks 0 hp1a 500x 4c2.tif]

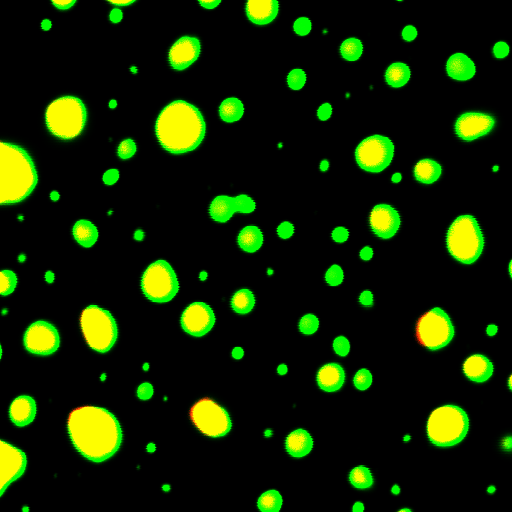

Supplement: Supplementary file 25 — Figure Source Data for Expanded View and Appendix [file 44318_2024_212_MOESM25_ESM.zip › Source Data for Expanded View and Appendix/Figure EV5/5A/10 irtks 20 hp1a 500x 2.tif]

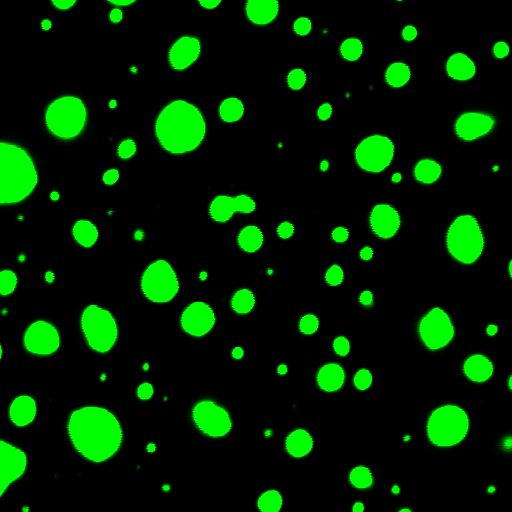

Supplement: Supplementary file 25 — Figure Source Data for Expanded View and Appendix [file 44318_2024_212_MOESM25_ESM.zip › Source Data for Expanded View and Appendix/Figure EV5/5A/10 irtks 20 hp1a 500x 2c1.tif]

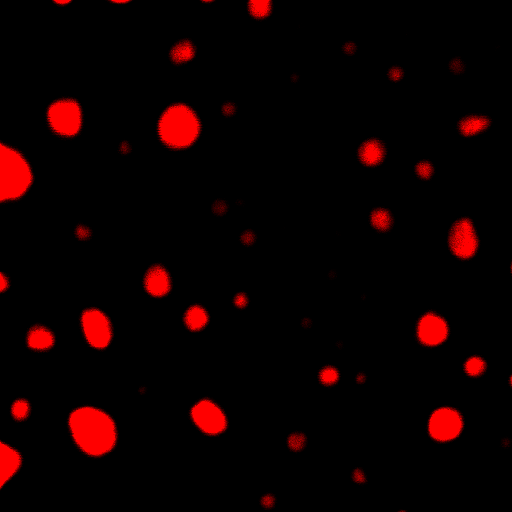

Supplement: Supplementary file 25 — Figure Source Data for Expanded View and Appendix [file 44318_2024_212_MOESM25_ESM.zip › Source Data for Expanded View and Appendix/Figure EV5/5A/10 irtks 20 hp1a 500x 2c2.tif]

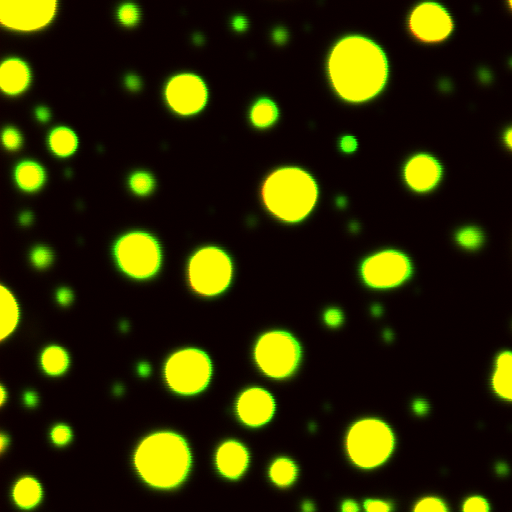

Supplement: Supplementary file 25 — Figure Source Data for Expanded View and Appendix [file 44318_2024_212_MOESM25_ESM.zip › Source Data for Expanded View and Appendix/Figure EV5/5A/10 irtks 40 hp1a 500x 3.tif]

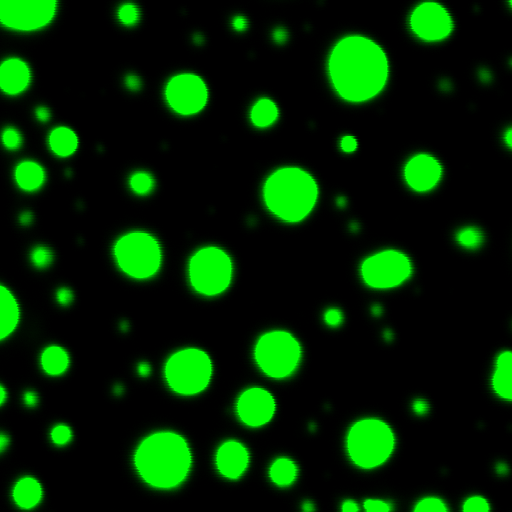

Supplement: Supplementary file 25 — Figure Source Data for Expanded View and Appendix [file 44318_2024_212_MOESM25_ESM.zip › Source Data for Expanded View and Appendix/Figure EV5/5A/10 irtks 40 hp1a 500x 3c1.tif]

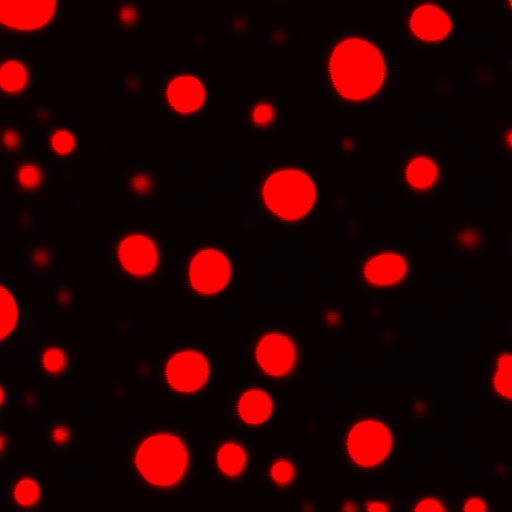

Supplement: Supplementary file 25 — Figure Source Data for Expanded View and Appendix [file 44318_2024_212_MOESM25_ESM.zip › Source Data for Expanded View and Appendix/Figure EV5/5A/10 irtks 40 hp1a 500x 3c2.tif]

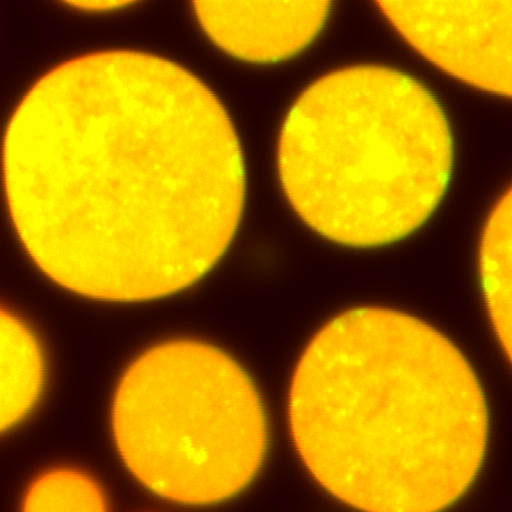

Supplement: Supplementary file 25 — Figure Source Data for Expanded View and Appendix [file 44318_2024_212_MOESM25_ESM.zip › Source Data for Expanded View and Appendix/Figure EV5/5A/10 irtks 80 hp1a 500x 2.tif]

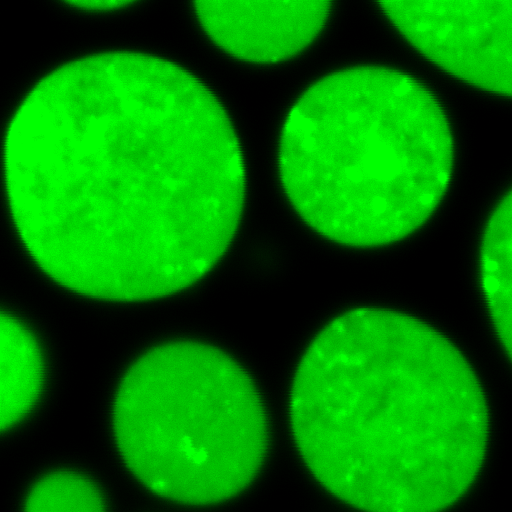

Supplement: Supplementary file 25 — Figure Source Data for Expanded View and Appendix [file 44318_2024_212_MOESM25_ESM.zip › Source Data for Expanded View and Appendix/Figure EV5/5A/10 irtks 80 hp1a 500x 2c1.tif]

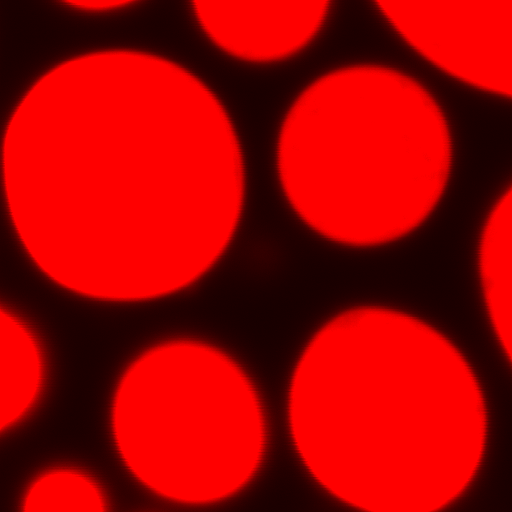

Supplement: Supplementary file 25 — Figure Source Data for Expanded View and Appendix [file 44318_2024_212_MOESM25_ESM.zip › Source Data for Expanded View and Appendix/Figure EV5/5A/10 irtks 80 hp1a 500x 2c2.tif]

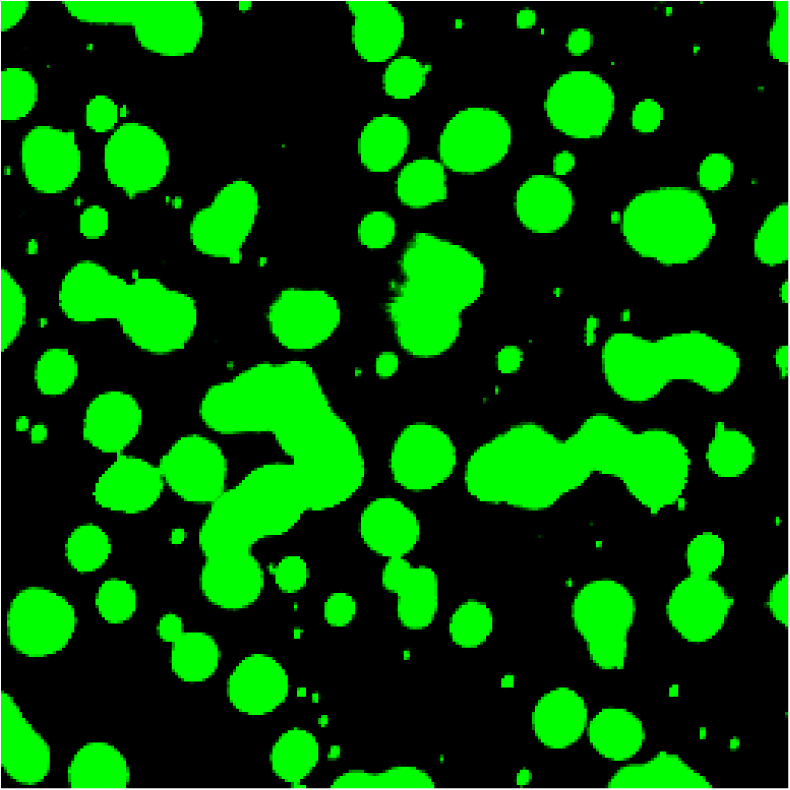

Supplement: Supplementary file 25 — Figure Source Data for Expanded View and Appendix [file 44318_2024_212_MOESM25_ESM.zip › Source Data for Expanded View and Appendix/Figure EV5/5A/20 irtks 0 hp1a 500x 2c.tif]

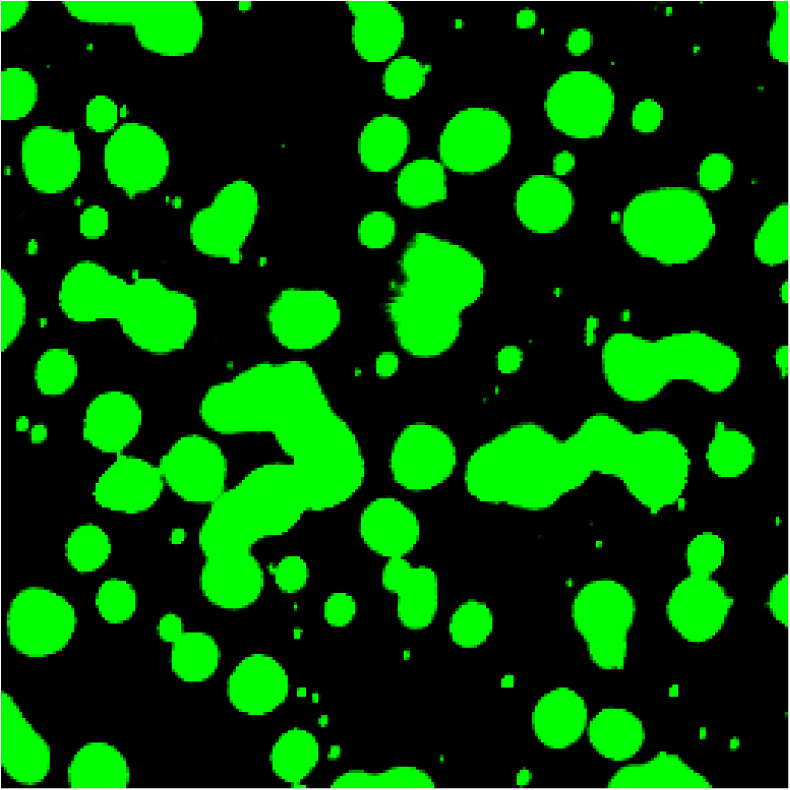

Supplement: Supplementary file 25 — Figure Source Data for Expanded View and Appendix [file 44318_2024_212_MOESM25_ESM.zip › Source Data for Expanded View and Appendix/Figure EV5/5A/20 irtks 0 hp1a 500x 2c1.tif]

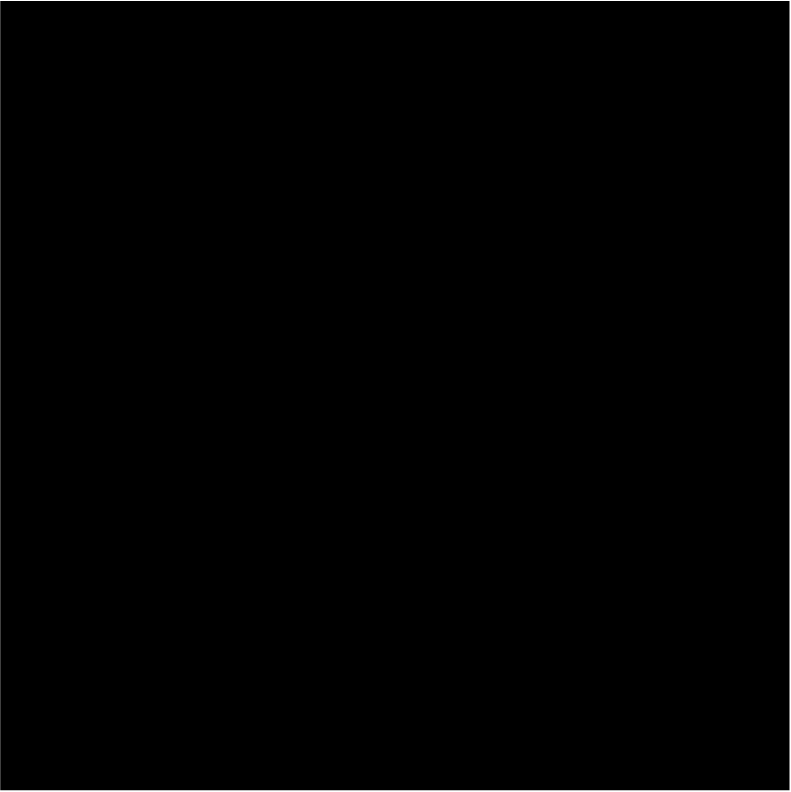

Supplement: Supplementary file 25 — Figure Source Data for Expanded View and Appendix [file 44318_2024_212_MOESM25_ESM.zip › Source Data for Expanded View and Appendix/Figure EV5/5A/20 irtks 0 hp1a 500x 2c2.tif]
