# Supplementary material for: Heterochromatin formation and remodeling by IRTKS condensates counteract cellular senescence
Source: EMBO J. 2024 Aug 27;43(20):7. doi: 10.1038/s44318-024-00212-3 (PMC11480336; doi:10.1038/s44318-024-00212-3)
Supplement: Supplementary file 26 — Expanded View Figures [file 44318_2024_212_MOESM26_ESM.pdf]

Expanded View Figures

Figure EV1. IRTKS regulates heterochromatin formation.

(**A**, **B**) Quantification of electron-dense regions (EDRs) around the nucleolus of the livers (**A**, ***$p = 2.72 \times 10^{-10}$) and kidneys (**B**, ***$p = 2.73 \times 10^{-9}$) from WT and *Irtks* KO mice. $n = 10$ cells analyzed for each condition. (**C–E**) Representative images (**C**) and quantification of EDRs at the nuclear periphery (**D**, ***$p = 3.09 \times 10^{-9}$) and around the nucleolus (**E**, ***$p = 5.49 \times 10^{-11}$) of the stomach tissues of WT and *Irtks* KO mice. Red arrows indicate the electron-dense heterochromatin regions. Nu, nucleolus. $n = 10$ cells analyzed for each condition. Scale bar, 1 µm. (**F**) Quantification of EDRs around the nucleolus in MEF cells. $n = 10$ cells analyzed for each condition. ***$p = 1.22 \times 10^{-13}$, *$p = 0.0134$. (**G**, **H**) Electron microscopy images (**G**) and quantification of EDRs at the nuclear periphery (**H**, ***$p = 2.14 \times 10^{-8}$) and around the nucleolus (**I**), ***$p = 1.19 \times 10^{-10}$) in WT and Irtks-KO SK-Hep-1 cells. Red arrows indicate the electron-dense heterochromatin regions. Nu, nucleolus. $n = 10$ cells analyzed for each condition. Scale bar, 1 µm. (**J–O**) Electron microscopy images and quantification of the electron-dense heterochromatin regions at the nuclear periphery and around the nucleolus in MEFs (**J–L**, respectively) and SK-Hep-1 cells (**M–O**, respectively) that were transfected with empty vector or Flag-IRTKS construct. Red arrows indicate the electron-dense heterochromatin regions. Nu, nucleolus. $n = 10$ cells analyzed for each condition (**K**, ***$p = 1.39 \times 10^{-9}$; **L**, *$p = 0.0370$; **N**, ***$p = 5.68 \times 10^{-6}$; **O**, ***$p = 6.90 \times 10^{-8}$). Scale bar, 1 µm. (**P**, **Q**) Representative confocal images (**P**) and line scan analysis (**Q**) of HP1α foci (red) and nuclei (DAPI, blue) in the stomach tissues of WT and *Irtks* KO mice. Quantification of lines scanned across HP1α foci and nuclei at the position depicted by the white arrow. Scale bar, 5 µm. (**R**, **S**) Western blotting analyses of HP1α expression in the livers (**R**) and kidneys (**S**) of WT and Irtks-KO mice. GAPDH was used as the loading control. (**T**) Representative confocal images and line scan analysis (right) on H3K9me3 (green) and nuclei (DAPI, blue) in the livers of WT and *Irtks* KO mice. Quantification of lines scanned across H3K9me3 foci and nuclei at the position depicted by the white arrow. Scale bar, 5 µm. (**U**) Representative confocal microscopy and line scan analysis of SK-Hep-1 cells showing the location of EGFP-HP1α foci. Nuclei were labeled with Hoechst 33342 (blue). Quantification of lines scanned across HP1α foci and nuclei at the position depicted by the white arrow. Scale bar, 5 µm. (**V**) Live-cell images and fluorescence recovery curves of FRAP experiments of EGFP-HP1α in SK-Hep-1 cells. Red arrow indicates the bleached point, which is boxed and amplified in the images on the right. $n = 8$ biological replicates for the FRAP curve construction. ***$p = 3.10 \times 10^{-9}$. Scale bar, 5 µm. (**W**) Representative confocal microscopy and line scan analysis of MEF cells showing the location of EGFP-HP1α foci. Nuclei were labeled with Hoechst 33342 (blue). Quantification of lines scanned across HP1α foci and nuclei at the position depicted by the white arrow. Scale bar, 5 µm. (**X**) Live-cell images and fluorescence recovery curves of FRAP experiments of EGFP-HP1α in MEF cells. Red arrow indicates the bleached point, which is boxed and amplified in the images on the right. $n = 8$ biological replicates for the FRAP curve construction. ***$p = 1.03 \times 10^{-6}$. Scale bar, 5 µm. Data are presented as the mean ± SD. Figure EV1V and y were tested by two-way ANOVA. The remaining plots were tested by two-tailed unpaired Student's t test. Source data are available online for this figure.

 © The Author(s)

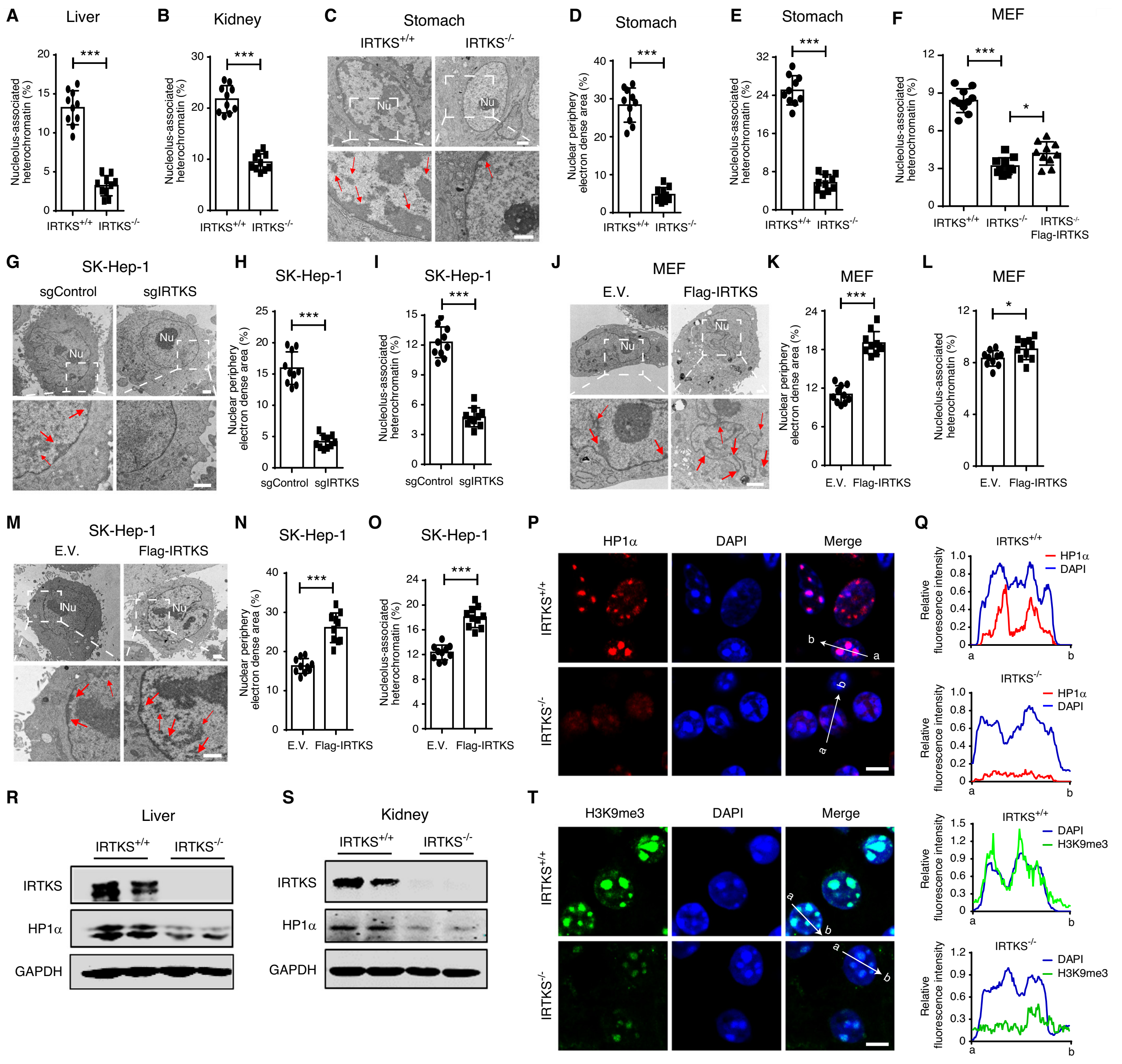

A Liver
Nucleolus-associated heterochromatin (%)
IRTKS+/+ IRTKS-/-
B Kidney
C Stomach
D Stomach
Nuclear periphery electron dense area (%)
E Stomach
F MEF
IRTKS-/- Flag-IRTKS
G SK-Hep-1
sgControl sgIRTKS
H SK-Hep-1
I SK-Hep-1
J MEF
E.V. Flag-IRTKS
K MEF
L MEF
M SK-Hep-1
N SK-Hep-1
O SK-Hep-1
P HP1α DAPI Merge
Q
Relative fluorescence intensity
R Liver
IRTKS
HP1α
GAPDH
S Kidney
T H3K9me3 DAPI Merge

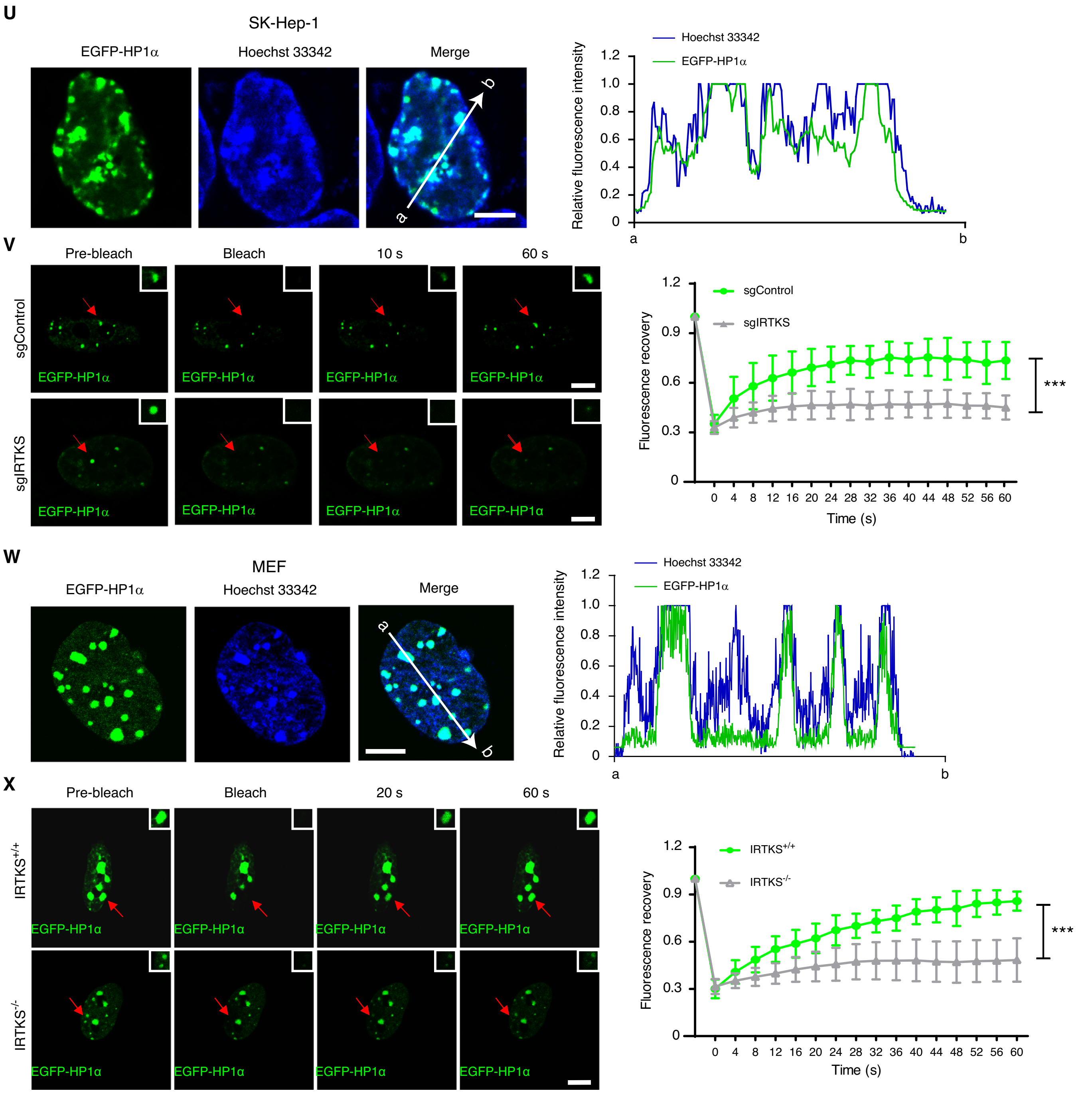
U
SK-Hep-1
EGFP-HP1α
Hoechst 33342
Merge
a
b
Hoechst 33342
EGFP-HP1α
Relative fluorescence intensity
V
Pre-bleach
Bleach
10 s
60 s
sgControl
sgIRTKS
EGFP-HP1α
Fluorescence recovery
Time (s)
***
W
MEF
EGFP-HP1α
Hoechst 33342
Merge
X
Pre-bleach
Bleach
20 s
60 s
IRTKS+/+
IRTKS-/-
EGFP-HP1α
Fluorescence recovery
Time (s)
***

 © The Author(s)

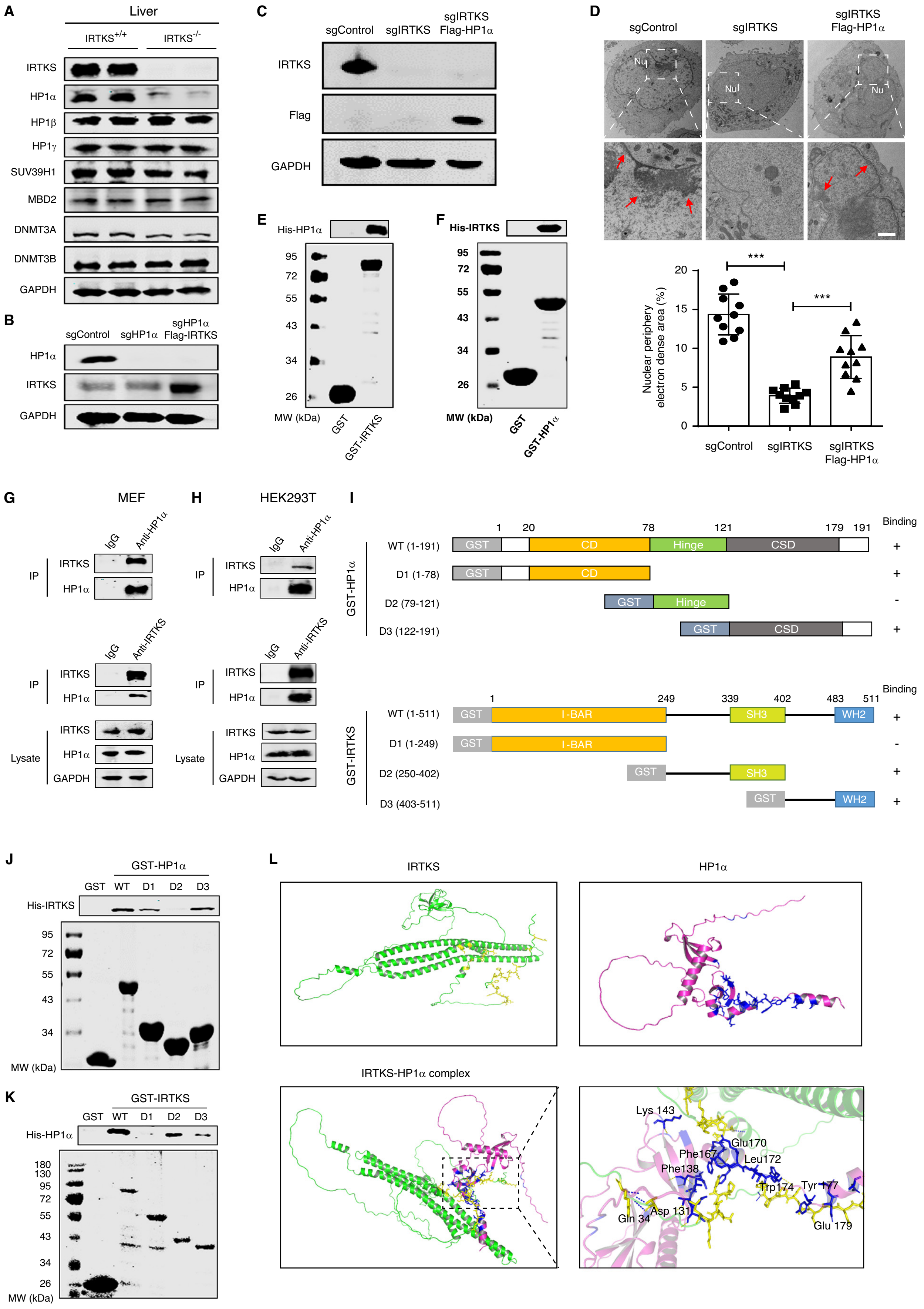
A
Liver
IRTKS+/+
IRTKS-/-
IRTKS
HP1α
HP1β
HP1γ
SUV39H1
MBD2
DNMT3A
DNMT3B
GAPDH
B
sgControl
sgHP1α
sgHP1α Flag-IRTKS
C
sgControl
sgIRTKS
sgIRTKS Flag-HP1α
Flag
D
Nu
Nuclear periphery electron dense area (%)
***
E
His-HP1α
MW (kDa)
GST
GST-IRTKS
F
His-IRTKS
GST-HP1α
G
MEF
IgG
Anti-HP1α
Anti-IRTKS
IP
Lysate
H
HEK293T
I
GST-HP1α
Binding
WT (1-191)
D1 (1-78)
D2 (79-121)
D3 (122-191)
CD
Hinge
CSD
GST-IRTKS
WT (1-511)
D1 (1-249)
D2 (250-402)
D3 (403-511)
I-BAR
SH3
WH2
J
K
L
IRTKS-HP1α complex
Lys 143
Glu170
Phe167
Leu172
Phe138
Trp174
Tyr 177
Gln 34
Asp 131
Glu 179

Figure EV2. IRTKS directly associates with HP1α.

(**A**) Western blotting analysis of several important epigenetic factors associated with heterochromatin formation in liver tissues from WT and *Irtks* KO mice. GAPDH was used as the loading control. (**B**) Western blotting analysis of HP1α and IRTKS expression in SK-Hep-1 cells treated with CRISPR/Cas9 single-guide RNA (sgRNA) lentivirus (sgHP1α), to knock out HP1α, coupled with the Flag-IRTKS construct. GAPDH was used as the loading control. (**C**) Western blotting analysis of HP1α and IRTKS expression in these SK-Hep-1 cells treated with CRISPR/Cas9 single-guide RNA (sgRNA) lentivirus (sgIRTKS) to knock out IRTKS, and then coupled with the Flag-HP1α construct. GAPDH was used as the loading control. (**D**) Electron microscopy images and quantification (bottom) of the electron-dense heterochromatin regions in SK-Hep-1 cells that were genetically engineered with CRISPR/Cas9 single-guide RNA (sgRNA) lentivirus (sgIRTKS) to knock out IRTKS, and then transfected with the Flag-HP1α construct. Red arrows indicate the electron-dense heterochromatin regions. Nu, nucleolus. $n = 10$ cells analyzed for each condition. ***$p = 7.52 \times 10^{-11}$ (sgControl *vs* sgIRTKS) and 4×10^{-5} (sgIRTKS *vs* sgIRTKS-Flag-HP1α). Scale bar, 1 μm. (**E**, **F**) IRTKS and HP1α reciprocally interact directly in a GST pull-down assay. Equivalent amounts of His-HP1α were incubated with either GST (negative control) or GST-IRTKS. After GST pulldown, HP1α was detected by western blotting. The mirror experiment was performed using His-IRTKS and GST-HP1α. (**G**, **H**) The reciprocal interaction between IRTKS and HP1α was detected by co-immunoprecipitation (co-IP) with anti-IRTKS and anti-HP1α antibodies in MEFs (**G**) and HEK293T cells (**H**). The immunoglobulin G (IgG) group was the negative control. (**I**) Schematic summaries of the interactions between diverse IRTKS and HP1α truncations. (**J**) Direct interaction between full-length or truncated GST-HP1α proteins and His-IRTKS revealed by GST pulldown assay. Full-length and truncated GST-HP1α proteins were visualized by Coomassie blue staining. (**K**) GST pulldown assays were performed with recombinant His-HP1α and full-length or truncated GST-IRTKS. The pulldown samples were analyzed by western blotting. (**L**) A 3D structural model of the IRTKS-HP1α complex was constructed using the Z-DOCK server. The 3D structures of IRTKS and HP1α were predicted by AlphaFold algorithms (upper panel). The interaction between IRTKS and HP1α is depicted by the yellow and blue colors, respectively. Details of the key residues of HP1α that interact with IRTKS are also shown in the lower panel. Data are presented as the mean ± SD. Figure EV2D was tested by one-way ANOVA followed by Tukey's post hoc test. Source data are available online for this figure.

 © The Author(s)

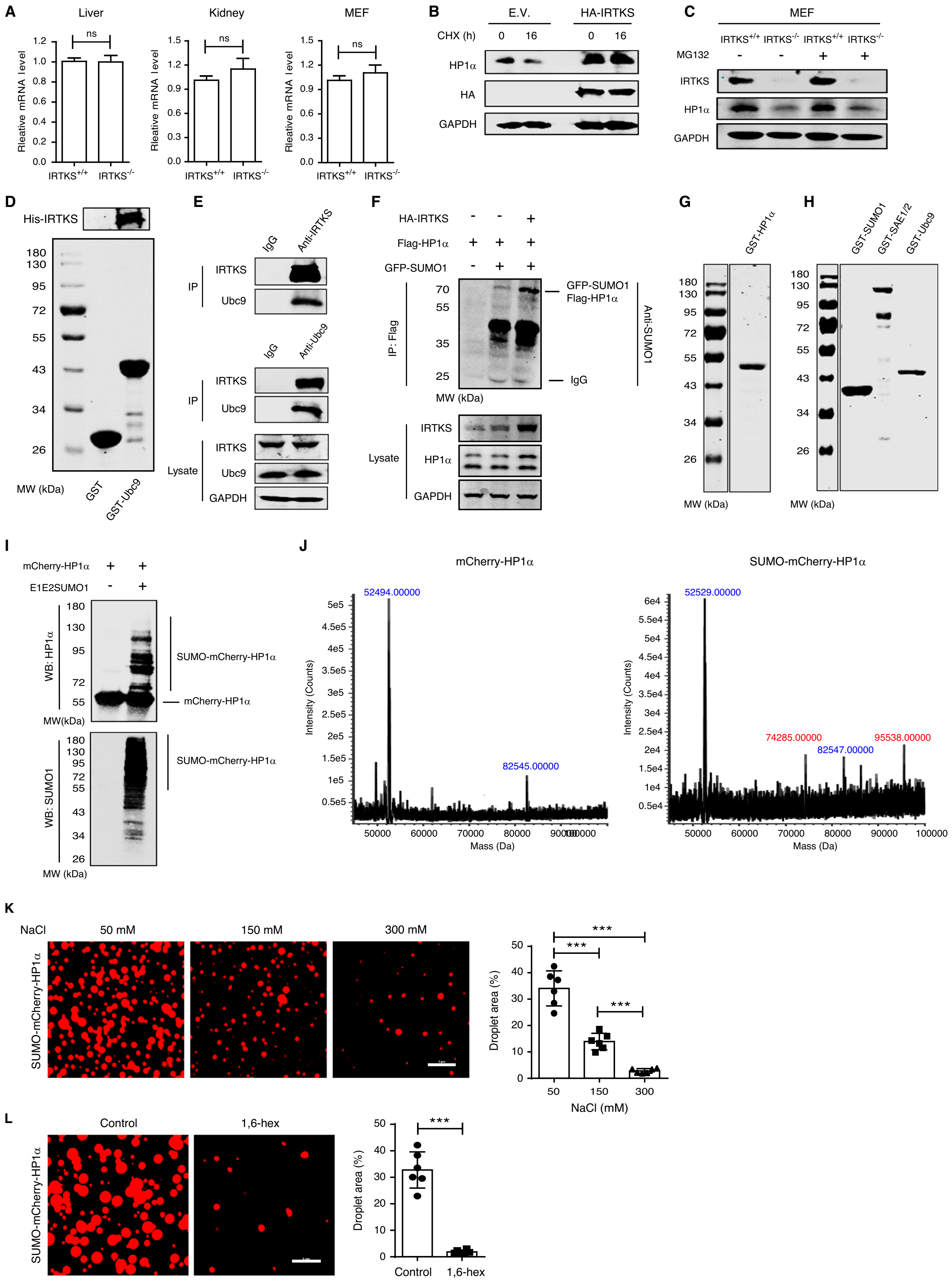

A
Liver
Kidney
MEF
ns
Rleative mRNA level
IRTKS+/+
IRTKS-/-
B
E.V.
HA-IRTKS
CHX (h)
0
16
HP1α
HA
GAPDH
C
MEF
MG132
IRTKS
HP1α
GAPDH
D
His-IRTKS
MW (kDa)
GST
GST-Ubc9
E
IgG
Anti-IRTKS
Anti-Ubc9
IP
Ubc9
Lysate
F
HA-IRTKS
Flag-HP1α
GFP-SUMO1
IP: Flag
GFP-SUMO1 Flag-HP1α
IgG
Anti-SUMO1
G
GST-HP1α
H
GST-SUMO1
GST-SAE1/2
GST-Ubc9
I
mCherry-HP1α
E1E2SUMO1
WB: HP1α
WB: SUMO1
SUMO-mCherry-HP1α
MW(kDa)
J
mCherry-HP1α
SUMO-mCherry-HP1α
52494.00000
82545.00000
52529.00000
74285.00000
95538.00000
82547.00000
Intensity (Counts)
Mass (Da)
K
NaCl
50 mM
150 mM
300 mM
SUMO-mCherry-HP1α
Droplet area (%)
NaCl (mM)
***
L
Control
1,6-hex

Figure EV3. SUMOylated HP1α follows the principle of phase separation.

(**A**) RT–qPCR showing RNA levels of HP1α in the livers, kidneys, and MEFs of WT and Irtks-KO mice. Relative RNA levels are normalized to GAPDH. $n = 3$ biological replicates. ns = 0.9397 (liver), 0.3482 (kidney), and 0.4195 (MEF). (**B**) Western blotting analysis showed the stability of HP1α in HEK293T cells that were transfected with empty vector or Flag-IRTKS construct after treatment with the protein synthesis inhibitor cycloheximide (CHX, 100 μg/ml) at the indicated time. (**C**) The HP1α level was not restored by MG132 (10 μM, 9 h), a proteasome inhibitor, in MEFs. (**D**) IRTKS can directly interact with Ubc9 in a GST pull-down assay in vitro. (**E**) The reciprocal interaction between IRTKS and Ubc9 was detected by co-immunoprecipitation (co-IP) with anti-IRTKS or anti-Ubc9 antibodies in HEK293T cells. The immunoglobulin G (IgG) group was the negative control. (**F**) The SUMO-1-mediated SUMOylation of HP1α was obviously enhanced by the co-expressed IRTKS in NIH3T3 cells, as detected by immunoprecipitation assay. NIH3T3 cells were transfected with HA-IRTKS, Flag-HP1α, and GFP-SUMO1, and then were immunoprecipitated with an anti-Flag antibody for the SUMOylation assay, followed by western blotting with the indicated antibodies. (**G**, **H**) Coomassie blue-stained images of purified GST-HP1α (**G**), GST-SUMO1, SAE1/2, and Ubc9 (**H**). (**I**) Western blotting analysis showing that mCherry-HP1α protein was SUMOylated by coexpression with E1E2SUMO1. (**J**) Results of Acquity UPLC I-class/VION IMS QTOF analysis for mCherry-HP1α and SUMO-mcherry-HP1α. The observed masses of mCherry-HP1α and SUMO-mCherry-HP1α proteins are also shown. The observed masses of SUMO-mCherry-HP1α proteins are increased compared with that of unmodified mCherry-HP1α. (**K**) Representative images and quantification of droplet formation at various salt concentrations. SUMO-mCherry-HP1α was added to droplet formation buffer to achieve a 20 μM protein concentration with a final NaCl concentration as indicated ($n = 8$ fields for each group were quantified). ***$p = 6.6 \times 10^{-7}$ (50 mM *vs* 150 mM), 2.03×10^{-9} (50 mM *vs* 300 mM), and 4.21×10^{-4} (150 mM *vs* 300 mM). Scale bar, 5 μm. (**L**) The droplet formation ability of SUMO-mCherry-HP1α was significantly depressed by 5% 1,6-hexanediol treatment. $n = 8$ fields for each group were quantified. ***$p = 9.36 \times 10^{-5}$. Scale bar, 5 μm. Data are presented as the mean ± SD or mean ± SEM. Figure EV3K was tested by one-way ANOVA followed by Tukey's post hoc test. The remaining plots were tested by two-tailed Student's t test. Source data are available online for this figure.

 © The Author(s)

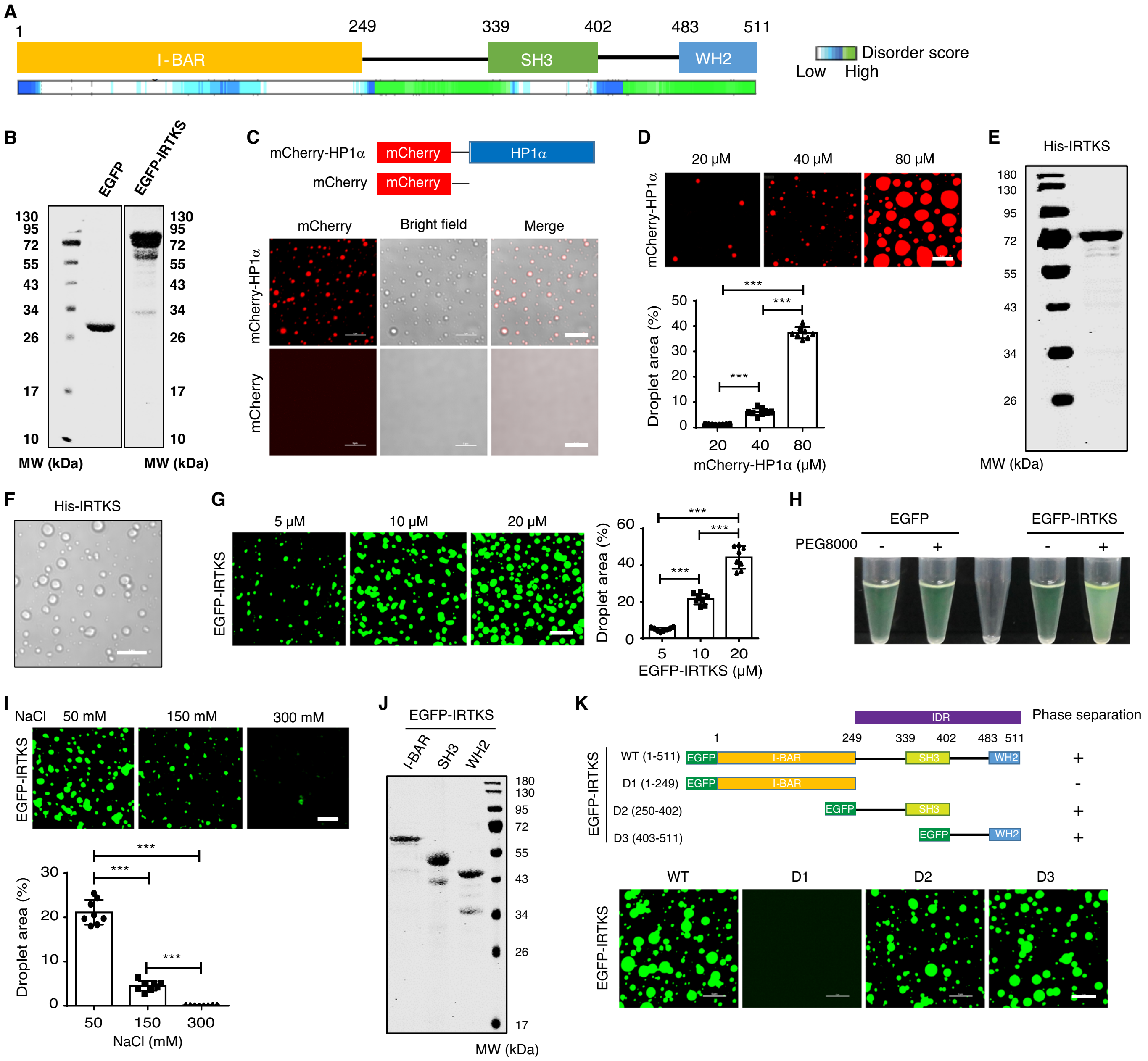
A
1
249
339
402
483
511
I-BAR
SH3
WH2
Disorder score
Low High
B
EGFP
EGFP-IRTKS
MW (kDa)
C
mCherry-HP1α
mCherry
HP1α
Bright field
Merge
D
20 μM
40 μM
80 μM
Droplet area (%)
mCherry-HP1α (μM)
E
His-IRTKS
F
G
5 μM
10 μM
20 μM
EGFP-IRTKS
EGFP-IRTKS (μM)
H
EGFP
PEG8000
I
NaCl 50 mM
150 mM
300 mM
NaCl (mM)
J
I-BAR
SH3
WH2
K
IDR
Phase separation
WT (1-511)
D1 (1-249)
D2 (250-402)
D3 (403-511)
WT
D1
D2
D3

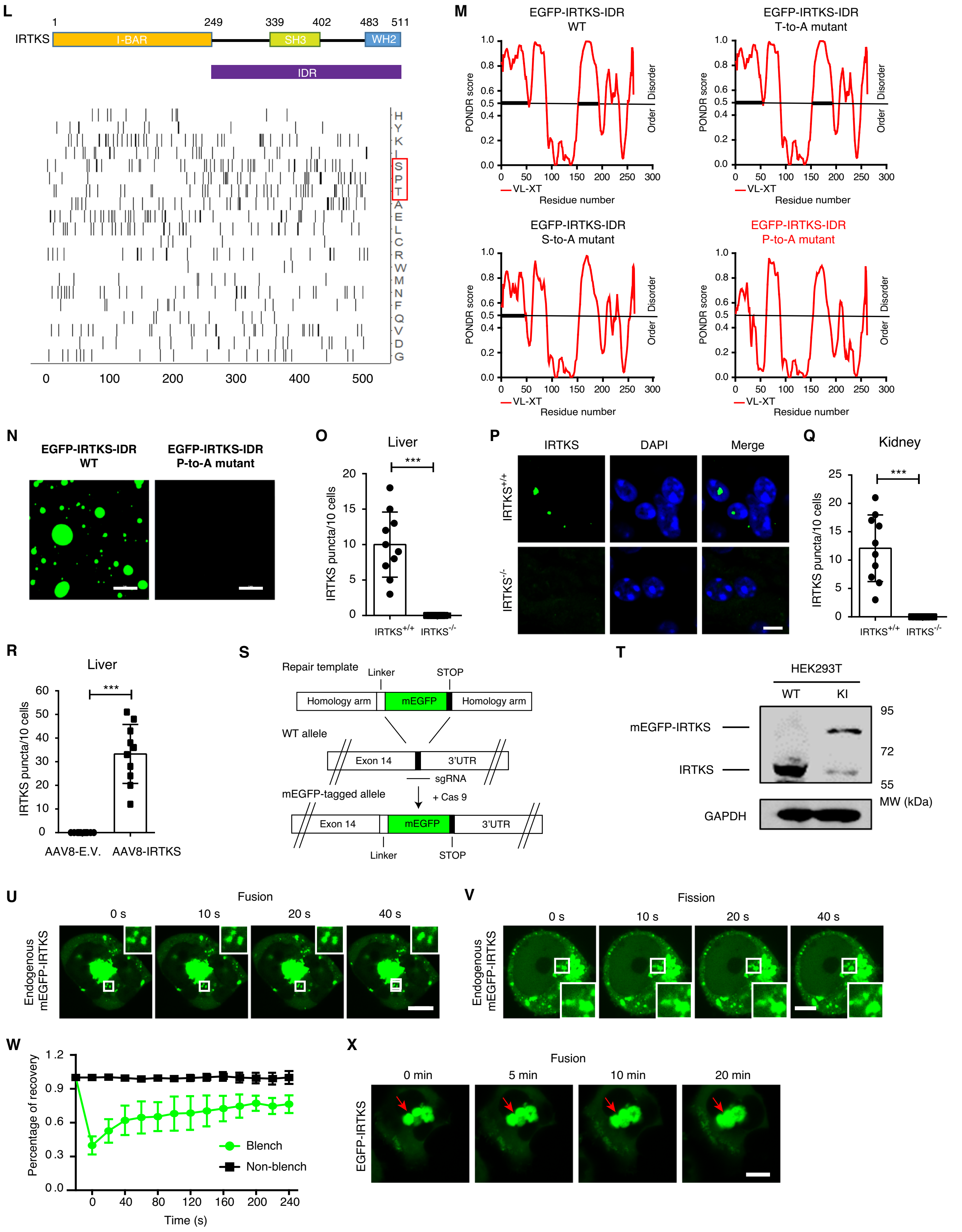
L
IRTKS
1
249
339
402
483
511
I-BAR
SH3
WH2
IDR
H Y K I S P T A E L C R W M N F Q V D G
0 100 200 300 400 500
M
EGFP-IRTKS-IDR WT
EGFP-IRTKS-IDR T-to-A mutant
EGFP-IRTKS-IDR S-to-A mutant
EGFP-IRTKS-IDR P-to-A mutant
PONDR score
Disorder
Order
VL-XT
Residue number
N
EGFP-IRTKS-IDR WT
EGFP-IRTKS-IDR P-to-A mutant
O
Liver
IRTKS puncta/10 cells
***
IRTKS+/+
IRTKS-/-
P
IRTKS
DAPI
Merge
IRTKS+/+
IRTKS-/-
Q
Kidney
IRTKS puncta/10 cells
***
IRTKS+/+
IRTKS-/-
R
Liver
IRTKS puncta/10 cells
***
AAV8-E.V.
AAV8-IRTKS
S
Repair template
Linker
STOP
Homology arm
mEGFP
Homology arm
WT allele
Exon 14
3'UTR
sgRNA
+ Cas 9
mEGFP-tagged allele
Exon 14
mEGFP
3'UTR
Linker
STOP
T
HEK293T
WT
KI
mEGFP-IRTKS
IRTKS
GAPDH
95
72
55
MW (kDa)
U
Fusion
0 s
10 s
20 s
40 s
Endogenous mEGFP-IRTKS
V
Fission
0 s
10 s
20 s
40 s
Endogenous mEGFP-IRTKS
W
Percentage of recovery
Blench
Non-blench
Time (s)
X
Fusion
0 min
5 min
10 min
20 min
EGFP-IRTKS

 © The Author(s)

Figure EV4. IRTKS possesses phase-separated properties.

(**A**) Schematic diagram of IRTKS domains and intrinsically disordered region (IDR) prediction by the D^2P^2 algorithm. (**B**) Coomassie blue staining image of purified EGFP and EGFP-IRTKS proteins. (**C**) Representative images of droplet formation showing that mCherry-HP1α has liquid-like properties. mCherry and mCherry-HP1α were added to droplet formation buffer to 40 μM. Scale bar, 5 μm. (**D**) In vitro droplet assay of mCherry-HP1α at the indicated concentrations ($n = 8$ fields for each group were quantified). ***$p = 1.55 \times 10^{-6}$ (20 μM *vs* 40 μM), 6.48×10^{-23} (20 μM *vs* 80 μM), and 1.42×10^{-21} (40 μM *vs* 80 μM). Scale bar, 5 μm. (**E**) Coomassie blue staining image of purified His-IRTKS. (**F**) Representative DIC images of droplet formation showing that His-IRTKS has liquid-like properties. His-IRTKS was added to droplet formation buffer to 20 μM. Scale bar, 5 μm. (**G**) Representative images and quantification of droplet formation at various protein concentrations. EGFP-IRTKS was added to droplet formation buffer to final concentrations as indicated ($n = 8$ fields for each group were quantified). ***$p = 4.13 \times 10^{-8}$ (5 μM *vs* 10 μM), 4.29×10^{-15} (5 μM *vs* 20 μM) and 1.55×10^{-10} (10 μM vs 20 μM). Scale bar, 5 μm. (**H**) Visualization of turbidity associated with droplet formation. Tubes containing EGFP (left pair) and EGFP-IRTKS (right pair) in the presence (+) or absence (–) of PEG-8000 are shown. Blank tubes are included between pairs for contrast. (**I**) Representative images and quantification of droplet formation at various salt concentrations. EGFP-IRTKS was added to droplet formation buffer to achieve a 10 μM protein concentration with a final NaCl concentration as indicated ($n = 8$ fields for each group were quantified). ***$p = 7.87 \times 10^{-15}$ (50 mM *vs* 150 mM), 6.92×10^{-17} (50 mM *vs* 300 mM), and 4.65×10^{-5} (150 mM *vs* 300 mM). Scale bar, 5 μm. (**J**) Coomassie blue staining image of purified EGFP-IRTKS truncations (I-BAR, SH3, and WH2). (**K**) A schematic summary of the droplet formation ability of full-length or truncated EGFP-IRTKS and representative images of droplet formation of various EGFP-IRTKS truncations. Scale bar, 5 μm. (**L**) Heatmap analyzing the amino acid composition and position of IRTKS. Each row represents information for a single amino acid. The length of the row corresponds to the length of the IRTKS protein. The purple bar represents the IDR of IRTKS shown in Extended Data Fig. 4a. (**M**) Predictions of IDRs of IRTKS with mutation of all prolines (P), serines (S) or threonines (T) to alanine (A) using the PONDR algorithm. (**N**) Mutating all prolines to alanine (P to A) disrupts phase separation. Representative images of droplet formation by wild-type IRTKS-IDR or the IRTKS-IDR P-to-A mutant fused to EGFP. Scale bar, 5 μm. (**O**) Quantification of IRTKS puncta number per 10 cells was analyzed in the livers from WT and *Irtks* KO mice. $n = 10$ for each group. ***$p = 7.21 \times 10^{-5}$. (**P, Q**) Immunofluorescent staining (**P**) of IRTKS (green) and DAPI (blue) and quantification (**Q**, ***$p = 1.1 \times 10^{-4}$) of IRTKS puncta number per 10 cells in kidney sections of WT and *Irtks* KO mice. Scale bar, 5 μm. $n = 10$ for each group. (**R**) Quantification of IRTKS puncta number per 10 cells was analyzed in the livers of these mice infected with AAV8-IRTKS and empty vector AAV8 (AAV-E.V.) as control. ***$p = 1.43 \times 10^{-5}$. $n = 10$ for each group. (**S**) Schematic of the strategy used to generate endogenously mEGFP-tagged IRTKS HEK293T cells. (**T**) Western blotting analysis of HEK293T cells with mEGFP knock-in at the endogenous IRTKS locus. (**U**) Time-lapse fluorescence images showing that the endogenous mEGFP-tagged IRTKS puncta rapidly fused in HEK293T cell. The fused puncta are boxed and enlarged in the images on the right. Scale bar, 5 μm. (**V**) Live-cell imaging of endogenous mEGFP-tagged IRTKS puncta in HEK293T cells. The white box indicated the fission events of IRTKS puncta and enlarged in the images on the right. Scale bar, 5 μm. (**W**) The fluorescence recovery curves of endogenous mEGFP-tagged IRTKS puncta shown by FRAP experiments ($n = 8$ biological replicates for the FRAP curve construction). (**X**) Fusion events of droplets over time indicate liquid-like material properties of EGFP-IRTKS in HEK293T cells. Red arrows indicate fusion events. Scale bar, 10 μm. Data are presented as the mean ± SD. Figures EV4O, Q and R were tested by two-tailed Student's t test. The remaining plots were tested by one-way ANOVA followed by Tukey's post hoc test. Source data are available online for this figure.

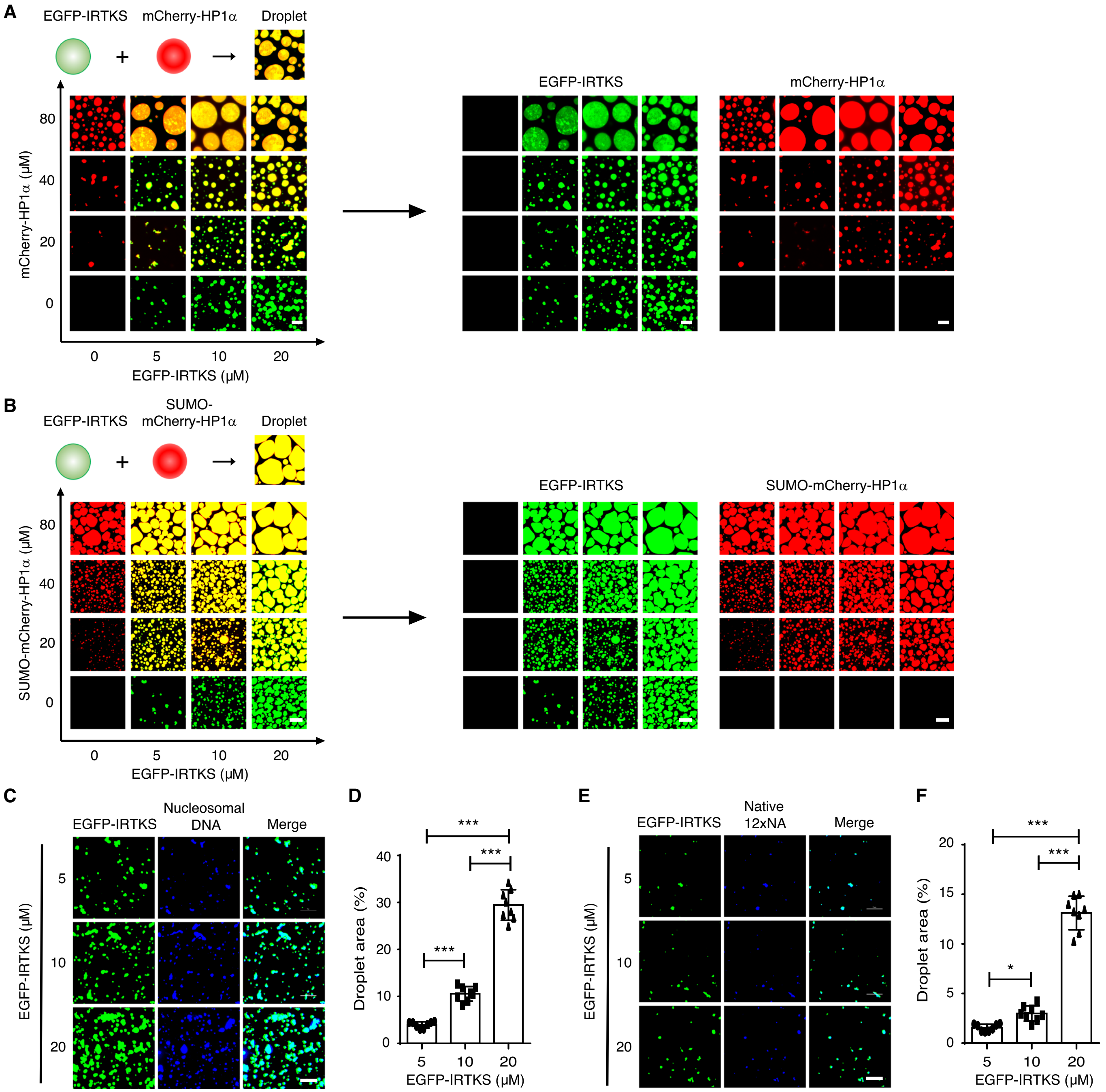
A
EGFP-IRTKS
mCherry-HP1α
Droplet
mCherry-HP1α (μM)
80
40
20
0
0
5
10
20
EGFP-IRTKS (μM)
EGFP-IRTKS
mCherry-HP1α
B
EGFP-IRTKS
SUMO-mCherry-HP1α
Droplet
SUMO-mCherry-HP1α (μM)
80
40
20
0
0
5
10
20
EGFP-IRTKS (μM)
EGFP-IRTKS
SUMO-mCherry-HP1α
C
EGFP-IRTKS
Nucleosomal DNA
Merge
EGFP-IRTKS (μM)
5
10
20
D
Droplet area (%)
***
***
***
EGFP-IRTKS (μM)
E
EGFP-IRTKS
Native 12xNA
Merge
EGFP-IRTKS (μM)
5
10
20
F
Droplet area (%)
***
***
*
EGFP-IRTKS (μM)

 © The Author(s)

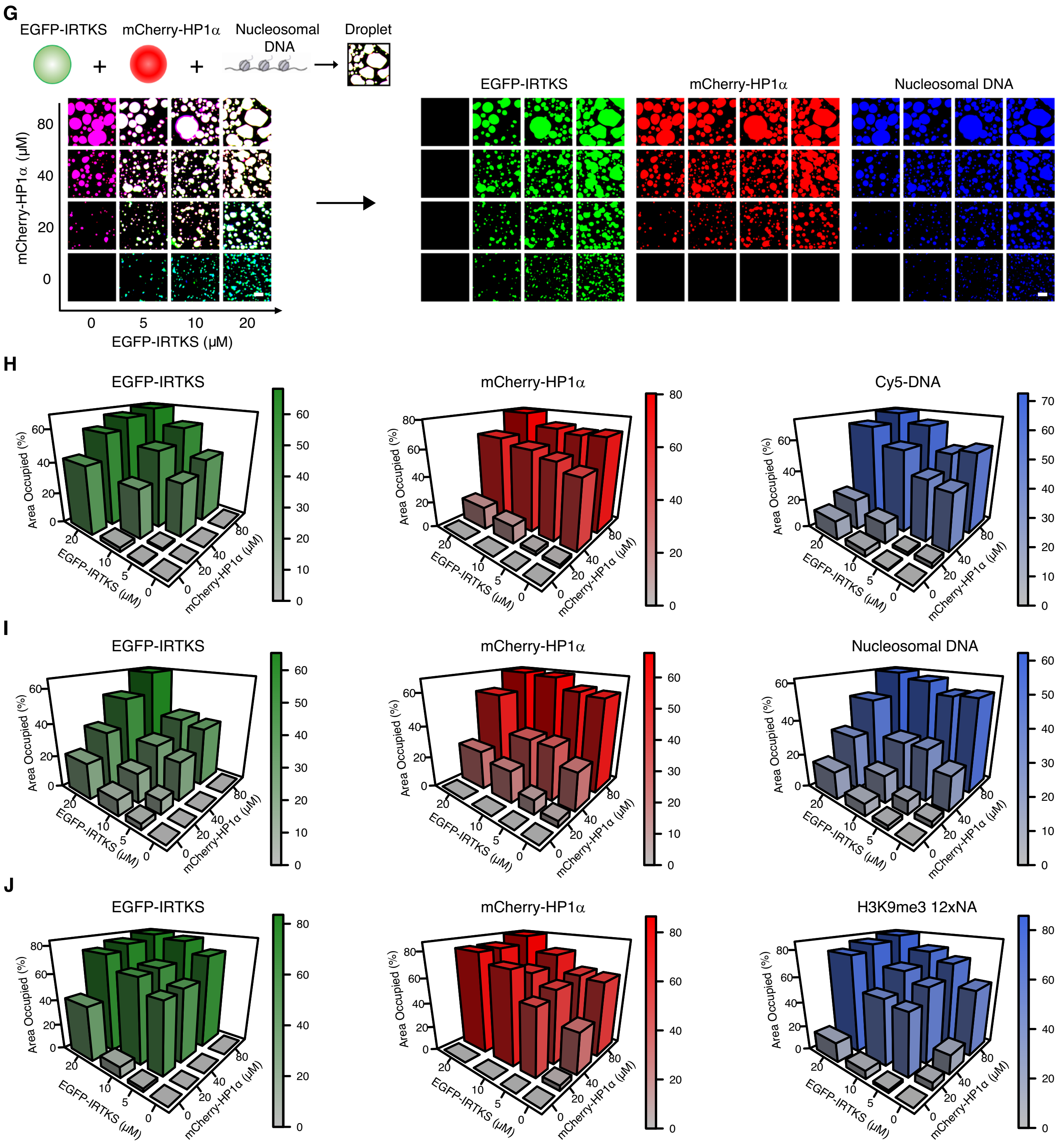
G
EGFP-IRTKS
mCherry-HP1α
Nucleosomal DNA
Droplet
mCherry-HP1α (μM)
EGFP-IRTKS (μM)
Nucleosomal DNA
H
EGFP-IRTKS
mCherry-HP1α
Cy5-DNA
Area Occupied (%)
I
EGFP-IRTKS
mCherry-HP1α
Nucleosomal DNA
J
EGFP-IRTKS
mCherry-HP1α
H3K9me3 12xNA

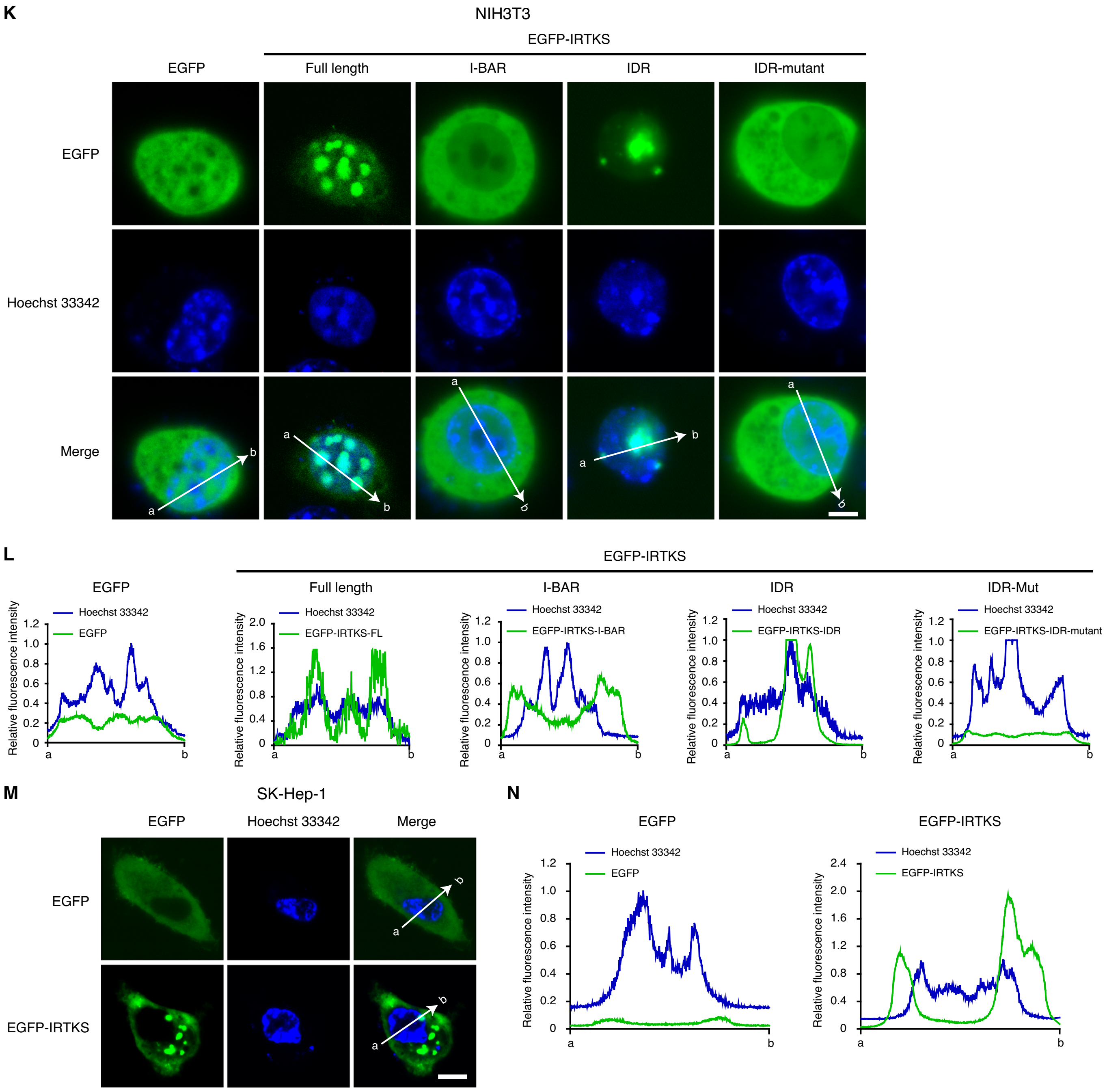
K
NIH3T3
EGFP-IRTKS
EGFP
Full length
I-BAR
IDR
IDR-mutant
EGFP
Hoechst 33342
Merge
L
EGFP-IRTKS
EGFP
Full length
I-BAR
IDR
IDR-Mut
Hoechst 33342
EGFP
EGFP-IRTKS-FL
EGFP-IRTKS-I-BAR
EGFP-IRTKS-IDR
EGFP-IRTKS-IDR-mutant
Relative fluorescence intensity
M
SK-Hep-1
EGFP
Hoechst 33342
Merge
EGFP
EGFP-IRTKS
N
EGFP
EGFP-IRTKS
Hoechst 33342
EGFP
EGFP-IRTKS
Relative fluorescence intensity

 © The Author(s)

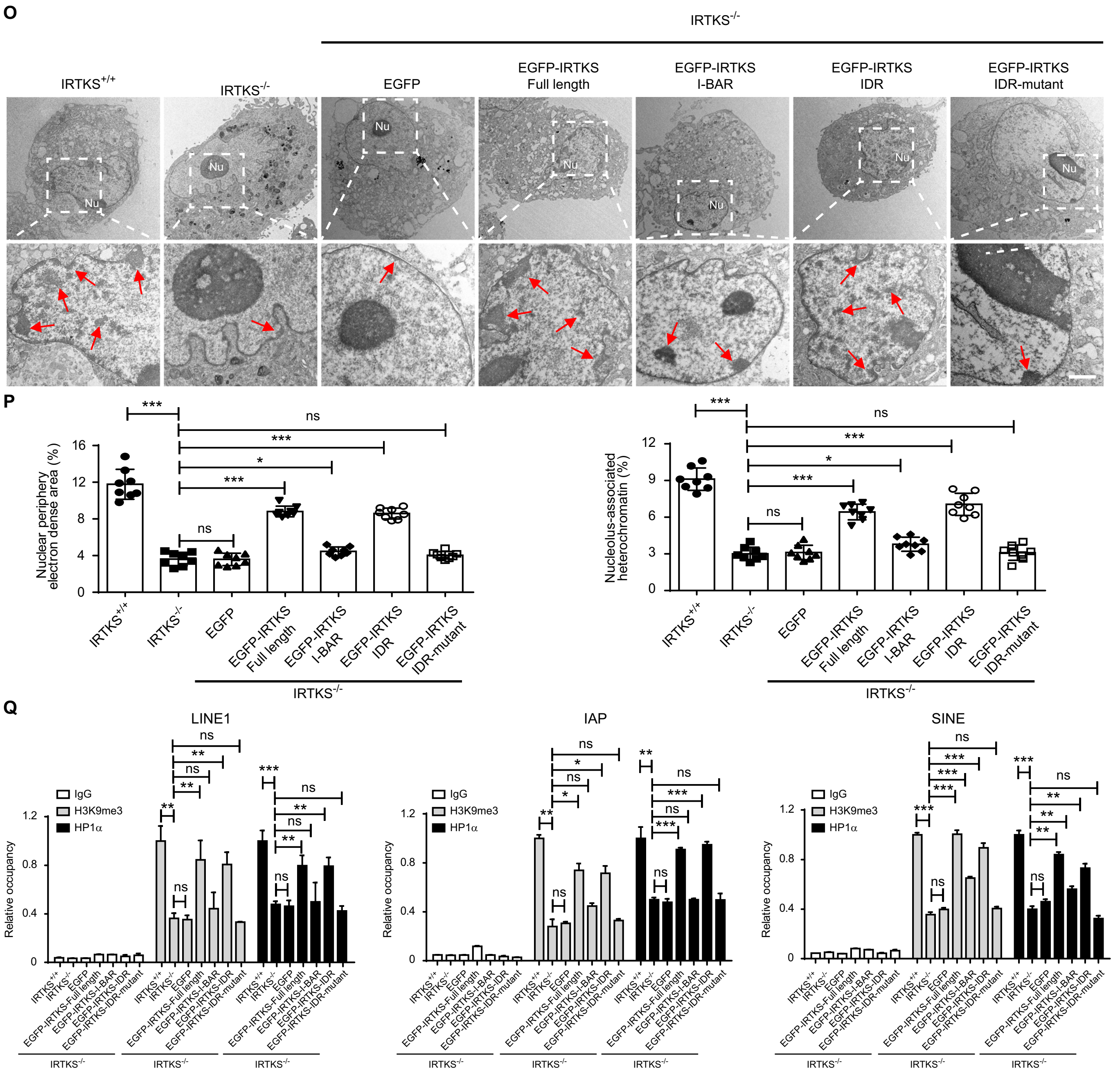
O
IRTKS-/-
IRTKS+/+
IRTKS-/-
EGFP
EGFP-IRTKS Full length
EGFP-IRTKS I-BAR
EGFP-IRTKS IDR
EGFP-IRTKS IDR-mutant
Nu
P
Nuclear periphery electron dense area (%)
Nucleolus-associated heterochromatin (%)
***
ns
*
IRTKS+/+
IRTKS-/-
EGFP
EGFP-IRTKS Full length
EGFP-IRTKS I-BAR
EGFP-IRTKS IDR
EGFP-IRTKS IDR-mutant
IRTKS-/-
Q
LINE1
IAP
SINE
IgG
H3K9me3
HP1α
Relative occupancy
**
ns
***
IRTKS-/-

Figure EV5. Liquid–liquid phase separation of IRTKS and HP1α droplets with diverse DNA-containing substrates.

(**A**) Representative images of droplet formation at different concentrations of IRTKS and HP1α protein. Concentrations of IRTKS and HP1α are indicated at the bottom and left of the images, respectively. Scale bars, 5 μm. (**B**) Representative images of droplet formation at various concentrations of EGFP-IRTKS and SUMO-mCherry-HP1α protein. Concentrations of EGFP-IRTKS and SUMO-mCherry-HP1α are indicated at the bottom and left of the images, respectively. Scale bars, 10 μm. (**C**, **D**) In vitro phase separation assay of EGFP-IRTKS protein at various concentrations mixed with nucleosomal DNA. A total of 6 nM nucleosomal DNA for the droplet assay was stained using DAPI ($n = 8$ fields for each group were quantified). ***$p = 3.3 \times 10^{-6}$ (5 μM *vs* 10 μM), 8.34×10^{-17} (5 μM *vs* 20 μM), and 3.42×10^{-14} (10 μM *vs* 20 μM). Scale bar, 5 μm. (**E**, **F**) Droplet formation of various concentrations of EGFP-IRTKS protein mixed with reconstituted native 12× nucleosomal arrays (NA). A total of 330 nM reconstituted native 12× NA for the droplet assay was stained using DAPI ($n = 8$ fields for each group were quantified). *$p = 0.0162$ (5 μM *vs* 10 μM), ***$p = 1.22 \times 10^{-15}$ (5 μM *vs* 20 μM), and 1.71×10^{-14} (10 μM *vs* 20 μM). Scale bar, 5 μm. (**G**) Liquid–liquid phase separation assay with nucleosomal DNA to examine the ability of IRTKS to form condensates with HP1α and nucleosomal DNA stained using DAPI. Scale bar, 5 μm. (**H–J**) A phase diagram of IRTKS and HP1α mixed with Cy5-labeled DNA oligos (**H**), nucleosomal DNA (**I**), and reconstituted H3K9me3 12× NA (**J**). $n = 8$ fields for each group were quantified. (**K**, **L**) Representative images (**K**) and line scan analysis (**L**) of EGFP or EGFP-IRTKS with different truncations (full-length, I-BAR, IDR and IDR-mutant) in NIH3T3 cells. Nuclei were labeled with Hoechst 33342. Quantification of lines scanned across EGFP or EGFP-IRTKS with different truncations and nuclei at the position depicted by the white arrow. Scale bar, 2 μm. (**M**, **N**) Live-cell images (**M**) and line scan analysis (**N**) of EGFP or EGFP-IRTKS-expressing SK-Hep-1 cells. Nuclei were labeled with Hoechst 33342. Quantification of lines scanned across EGFP or EGFP-IRTKS and nuclei at the position depicted by the white arrow. Scale bar, 5 μm. (**O**, **P**) Electron microscopy images (**O**) and quantification of the electron-dense heterochromatin regions (**P**) in MEF cells overexpressed EGFP or EGFP-IRTKS with different truncations (full-length, I-BAR, IDR, and IDR-mutant). Red arrows indicate the electron-dense heterochromatin regions. Nu, nucleolus. $n = 8$ cells analyzed for each condition. Scale bar, 1 μm. (**Q**) Enrichment of H3K9me3 and HP1α within the regions of repetitive sequences (LINE1, IAP, and SINE) in the MEFs of WT and *Irtks* KO mice as measured by ChIP-qPCR. $n = 3$ technical replicates in independent experiments. The *p* values of Fig. EV5P and Q were provided in Source data. Data are presented as the mean ± SD or mean ± SEM and the *p* value of one-way ANOVA followed by Tukey's post hoc test. Source data are available online for this figure.

 © The Author(s)
